# Development of a prognostic risk model for colorectal cancer based on microsatellite stability-associated genes

**DOI:** 10.1186/s12885-025-14918-y

**Published:** 2025-10-01

**Authors:** Xuefeng Zheng, Yunduan He, Zhan Tuo, Kuikui Zhu, Hong Ge, Xu Wang

**Affiliations:** Department of Radiation Oncology, The Affiliated Cancer Hospital of Zhengzhou University & Henan Cancer Hospital, Zhengzhou, China

**Keywords:** Colorectal cancer, Microsatellite, Prognostic genes, Risk score

## Abstract

**Background:**

Colorectal cancer (CRC) patients have a high recurrence rate, impacting survival. Microsatellite instability (MSI) is strongly linked to CRC development, making the MSI-related prognostic genes crucial for diagnosis and treatment.

**Methods:**

This study used CRC datasets, including TCGA-CRC, GSE17537, GSE39582, and GSE18088. We analyzed differential expression between CRC and control samples, and between MSS and MSI-H samples. Key genes were identified through a co-expression network and used to develop a prognostic risk model. The model's performance was validated in GSE17537, and independent prognostic factors were identified to construct a survival nomogram. We also explored pathways linked to the risk groups and their association with the tumor immune microenvironment, and predicted potential therapeutic agents for CRC.

**Results:**

We identified 11 prognostic genes (*CHGB*, *FABP4*, *PLIN4*, *PLIN1*, *RPRM*, *C7*, *AQP8*, *C2CD4A*, *APLP1*, *ADH1B*, and *CD36*) and developed a CRC risk model that showed significant survival differences in the TCGA-CRC cohort and GSE17537, with AUCs over 0.6 at 3, 5, and 7 years. Independent prognostic factors included risk score, age, tumor stage, and pathological N, and a nomogram was created for survival prediction. The identified genes may influence CRC through various pathways and are linked to immune responses. Bleomycin emerged as a potential treatment, with *CHGB* and *RPRM* regulated by non-coding RNAs and transcription factors, possibly affecting CRC development.

**Conclusions:**

Our analysis of microsatellite stability-associated genes in CRC highlights their impact on TIME, clinicopathological features, and prognosis, providing new insights into predicting prognosis and developing personalized treatments.

**Supplementary Information:**

The online version contains supplementary material available at 10.1186/s12885-025-14918-y.

## Background

The International Agency for Research on Cancer (IARC) has documented that Colorectal Cancer (CRC) is the third most diagnosed and second most deadly among all cancer groups globally [1, 2]. This underscores the substantial risk CRC poses to human health, given its status as one of the most lethal malignant tumors [1, 3–5]. Accurate prognostic assessment is essential for the selection of tumor treatments, as it facilitates the development of more personalized therapeutic strategies and patient care. Currently, the American Joint Committee on Cancer (AJCC) staging system's TNM staging guidelines are the most frequently employed prognostic system in clinical settings. Although most research affirms that CRC prognosis is primarily determined by stage, some studies have indicated that stage IIIa patients may experience more favorable outcomes than select stage II patients [6, 7]. Survival heterogeneity is evident among patients who share the same stage and prognosis, and this variability may be attributed to various factors such as patient age, tumor size, gross type, histological type and grade, growth pattern, tumor interstitial response, lymph node immune response, venous and perineural infiltration, tumor bud and micropapilla, DNA content, and ploidy analysis [8, 9]. Consequently, it is imperative to develop a more precise prognostic prediction model that can aid in clinical decision-making and enhance the practice of precision medicine.

In recent times, an increasing number of molecular markers have been employed to forecast the prognosis of CRC patients. The mutations in *KRAS*, *BRAF*, *VEGF*, and *PIK3CA* genes have been identified as adverse predictors of overall survival (OS) [10–12]. The PETACC-3 study [13], which analyzed *BRAF* and *KRAS* mutations in 1,404 colon cancer patients, demonstrating that patients with the *BRAF* mutation had inferior OS compared to those with the wild type, and the contrast was more pronounced when stratified by microsatellite instability (MSI). Nazemalhosseini ME et al. reported that *CIMP*-high is an independent prognostic factor for reduced colon cancer-specific mortality [14]. Nonetheless, the majority of *PI3K* inhibitors have demonstrated significant toxicity or deleterious side effects in vitro, thereby limiting their effectiveness for clinical translation. In CRC patients with *BRAF* gene mutations, only those with Class I mutations have shown sensitivity to *BRAF* and *MEK* inhibitors, accounting for approximately 10% of patients. While those with Class II/III mutations exhibit resistance to *BRAF* inhibitors. Moreover, the multigene combination test offers better precision and dimensionality compared to these markers. In 2015, the CRC Subtyping Consortium utilized six subtyping systems to identify four consensus molecular subtypes (*CMS1*, *CMS2*, *CMS3*, and *CMS4*) for colon cancer, resulting in the most comprehensive classification system to date [15]. *CMS1* tumors are characterized by MSI and immune features, while *CMS2*, *CMS3*, and *CMS4* exhibit canonical, metabolic, and mesenchymal phenotypes, respectively [16, 17]. Notably, *CMS4* demonstrated the poorest relapse-free survival, with a 60% survival rate at 5 years, compared to 75% for *CMS1* and 73% for *CMS2/3* [16, 18, 19]. The *CMS* classification system offers medical oncologists the opportunity to individualize treatment plans. Nevertheless, the utilization of *CMS* molecular typing in clinical practice has not been widely implemented due to the limited integration of gene sequencing in routine applications.

Numerous studies have shown that high microsatellite instability (MSI-H) status is present in various tumors, including colorectal cancer (CRC), endometrial cancer, gastric cancer, and head and neck squamous cell carcinoma. The clinical efficacy of immune checkpoint blockade is directly related to the MSI status, particularly in CRC [20]. The main treatment methods for tumors involve surgery, chemotherapy, and radiotherapy, and CRC treatment is now entering the era of immunotherapy [21–23]. The findings of the phase II KEYNOTE-016 study indicate that pembrolizumab, a monoclonal anti-PD-1 antibody, yielded an objective response rate of 40% in patients with progressive DNA mismatch repair-deficient (dMMR) metastatic CRC, compared to0% in patients with MMR-proficient (pMMR) CRC [20, 24]. Similarly, the NICHE and CheckMate142 studies demonstrated that microsatellite stable (MSS)*/*pMMR tumors exhibited minimal pathological remission with *CTLA-4/PD-1* dual antibody immunotherapy [24]. Although MSI is commonly assessed in the diagnosis and treatment of CRC, its prognostic value remains a topic of debate. Notably, approximately 10% of stage III CRC patients with MSI-H or dMMR. While MSI has been linked to improved OS [25], conflicting data exist regarding the relationship between MMR status and prognosis.

The aforementioned evidence suggests that the dichotomy between MSS and MSI is imprecise. The objective response rate (ORR) of pembrolizumab and nivolumab, which serves as a predictor of immunotherapy efficacy, typically falls below 50% (28%−52%) for MSI CRC patients [26], highlighting their inherent heterogeneity. Furthermore, the MSI-based prognostic assessment of CRC varies across different tumor stages. To address these issues, we obtained CRC related data from the University of California Santa Cruz (UCSC) Xena database. Differential expression analysis was conducted to identify differentially expressed genes (DEGs) between the MSI-H and MSS groups, as well as between the CRC and control groups. The module exhibiting the strongest correlation with CRC was identified using weighted gene co-expression network analysis (WGCNA), and a prognostic risk model was constructed through univariate and multivariate Cox analyses. Independent prognostic factors significantly associated with survival were obtained through independent prognostic analysis. Subsequently, potential therapeutic agents for CRC were predicted. The study successfully developed a prognostic risk model for CRC related to MSI, which holds significant implications for the management and prognosis of CRC.

## Methods

### Acquisition of data

The TCGA-CRC cohort, namely the colon and rectal cancer related data (638 CRC samples, and 51 control samples), was obtained via UCSC Xena database (https://xenabrowser.net/datapages/). The 638 CRC samples served as the training set, including both MSS and MSI-H samples. The GSE17537, GSE18088, GSE106584 and GSE39582 were acquired via GEO query package (v2.62.2) [27], which included 55 CRC, 53 CRC, 154 CRC (Two samples with a survival time of less than 60 days were excluded), and 585 samples (566 CRC and 19 non-tumor samples) respectively, and considered as validation sets. The data obtained from the GEO database typically undergoes a series of standardization processes. Initially, background correction is performed to eliminate background noise. Subsequently, normalization is conducted to ensure comparability of expression values across different samples. Finally, a logarithmic transformation, often employing a log2 scale, is applied to mitigate the skewness in the data distribution. Clinical data processing included recoding blank entries,"not reported","Tis","NX"and"MX"were recoded as missing values (NA). The missing data rates for all variables were < 12% (maximum: Pathologic M at 11.7%), primarily due to: unreported metastasis status (11.7%), incomplete staging (TumourStage, 3.5%), incomplete lymph node assessment (Pathologic N, 0.7%), and missing primary tumor assessment (Pathologic T, 0.5%). A deletion method was employed for data processing, followed by complete case analysis after ensuring data consistency. The Kolmogorov–Smirnov test (*P* = 0.999) and Fisher's exact test (*P* = 1.0) indicated no significant differences (*P* > 0.05) in risk score distributions or survival status before and after the removal of missing values. The flowchart of this study is shown in Figure S1.

### Construction of gene co-expression network

To screen for CRC-related key module genes, gene co-expression networks were constructed using the WGCNA package (v1.70–3) [28]. In the TCGA-CRC cohort, all samples were hierarchically clustered, and outlier samples were excluded. The optimal soft threshold was selected to construct the scale-free network based on the R2 exceeding 0.85 and the connectivity tending to 0. Then, co-expression network construction and module detection were performed based on gene FPKM and sample information, and module clustering dendrograms were drawn. Pearson correlation was employed to screen the modules with the strongest correlation with CRC, which contained the genes as key module genes.

### Differential expression analysis, enrichment analysis and construction of protein–protein interaction (PPI) network

The differential expression analysis on ‘MSI-H vs. MSS’ and ‘CRC vs. Control’ groups in TCGA-CRC cohort was conducted to identify DEGs1 and DEGs2 by DESeq2 package (v1.34.0) [29] setting |log_2_FC|> 0.5 and adj.*P* < 0.05, respectively. The intersection of DEGs1, DEGs2, and key module genes was then taken to obtain intersecting genes. Enrichment analysis was performed using clusterProfiler (v4.2.2) [30] to probe pathways and functions related to intersected genes, including gene ontology (GO) and kyoto encyclopedia of genes and genomes (KEGG) (*p*.adj < 0.05). Further, these genes were entered into the STRING database (http://string-db.org) to retrieve potential interactions among the encoded proteins, and a PPI network was constructed.

### Construction, assessment and verification of prognostic risk model

In the training set, univariate Cox analysis was performed using survival package (v3.3–1) [31] to screen for survival-related genes in intersected genes (*P* < 0.05). To enhance the robustness of the model and prevent overfitting, the Cox proportional hazards model was constructed using the coxph function in the"survival"R package (v 3.8.3) [32], and determined the optimal regularization parameter λ through tenfold cross-validation to minimize partial likelihood bias. This method compresses the coefficients of non-information features to zero, thereby selecting the most predictive genes. Then the proportional hazards (PH) assumption test was implemented (*P* > 0.05). Genes that passed the PH assumption test were included in the multifactorial Cox analysis for further screening (P < 0.05), and the genes obtained from the screening were named prognostic genes. Then, we calculated the risk score for each sample in the training set, CRC samples were divided into high/low risk groups according to the median risk score. The risk scoring formula is as follows:

$$Risk score=\left(\beta i\times Expi\right)$$

Among them, βi is the regression coefficient of each gene obtained by multivariate Cox regression, and Expi is the expression value of the corresponding gene (standardized). The specific β values and weight proportions of the 11 genes are presented in Table S3. The survival difference between the high- and low- risk groups were compared by Kaplan–Meier (K-M) survival curves using survminer (v0.4.9) [33] (*P* < 0.05). In order to evaluate prediction accuracy of the prognostic risk model, we calculated the false- and true-positive values of CRC samples in the training set, and plotted the receiver operating characteristic (ROC) curves generated by survivalROC (v1.0.3) [34]. Time-dependent ROC curves of the model at 3-year, 5-year and 7-year survival time points were calculated, and the corresponding AUC values were obtained. Furthermore, the results were validated by the same method in the GSE17537 and GSE106584 validation sets.

### Survival analysis and prognostic genes expression analysis

This study performed survival analysis on MSI-H and MSS samples in training set and compared the survival difference between these two groups (*P* < 0.05). The expression differences of prognostic genes were then analyzed between CRC and control samples, as well as between MSI-H and MSS samples. Additionally, the expression of prognostic genes between CRC and control samples, and between MSI-H and MSS samples, was validated separately in the GSE39582 and GSE18088 validation sets (*P* < 0.05). To investigate the surface distribution of pre- and post-gene coding albumen in both tumor and normal tissues, the Human Protein Atlas (HPA) database (https://www.proteinatlas.org) should be utilized for both the target and control groups in the context of gene-encoding protein immunohistochemical analysis. This study aims to verify whether the protein expression levels of key genes correlate with prognosis and to evaluate their potential as clinical biomarkers.

### Independent prognostic analysis

Risk scores were incorporated into clinical characteristics for univariate Cox analyses (*P* < 0.05) to screen for factors associated with survival. These factors were then subjected to PH assumption testing (*P* > 0.05) followed by inclusion in multivariate Cox analyses (*P* < 0.05) to identify independent prognostic factors for CRC. To elucidate the direct association between genes and clinical indicators and enhance the clinical relevance of genes, 11 prognostic genes were jointly analyzed with clinical characteristics (age, gender, disease stage, TNM stage, etc.). To assess the 3-, 5-, and 7-year survivability of CRC patients, nomograms containing independent prognostic factors were constructed using the rms package (v6.2–0) [35]. Subsequently, the predictive ability of the model was validated by calibration curves and ROC curves.

### Gene set enrichment analysis (GSEA)

To detect the pathways associated with all genes in the risk group, GSEA was conducted using clusterProfiler (v4.2.2) [30]. Pathways with a *p*.adj less than 0.05 were considered significantly enriched, and the top 5 most significant pathways were selected for display.

### Tumor immune microenvironment (TIME)

In the training set, we utilized the cell type identification by estimating relative subsets of RNA transcripts (CIBERSORT) algorithm to calculate the immune cell infiltration abundance of tumor samples. The immune cell infiltration and 9 immune checkpoints (*CD160, CD244, CTLA-4, KLRG1, LAG-3, PD-1, TIGIT, TIM-3, VISTA*) were compared between high- and low- risk groups to identify significantly different immune cells and immune checkpoints via Wilcoxon. The significantly different immune cells and immune checkpoints were then separately correlated with the risk score. We obtained the infiltration abundance data for M2 macrophages and regulatory T cells from the previously calculated immune cell infiltration dataset. Subsequently, we performed a correlation analysis with the associated risk score utilizing the corrplot package (v 0.95) [36]. The linear relationship between the risk score and the abundance of each immunosuppressive cell infiltration was evaluated by using the Pearson correlation coefficient calculation. To provide a more intuitive visualization of these associations, scatter plots were generated to depict the relationship between the risk score and the infiltration abundance of M2 macrophages and regulatory T (Treg) cells. Finally, the tumor immune dysfunction and exclusion (TIDE) value for each CRC sample was calculated, and the samples were divided into responder and non-responder groups based on the TIDE value. The Wilcoxon method was used to test the difference of risk scores between responder and non-responder groups. ROC curves were drawn to determine the efficacy of the risk score in predicting response to immunotherapy.

### Somatic mutation analysis

A total of 556 CRC samples in the TCGA-CRC cohort had somatic mutation information. We used the maftools package (v2.10.5) [37] to analyze and visualize the somatic mutation in these 556 CRC patients. Mutation categories, mutation frequencies, and other related data were then counted, and the samples with tumor mutational burden (TMB) information were identified. The Wilcoxon test was used to compare difference in TMB value between high- and low- risk groups.

### Drug sensitivity analysis and molecular network

Drug sensitivity data were obtained from the CellMiner database (https://discover.nci.nih.gov/cellminer/CellMiner), and drugs that met clinical trial validation and FDA standard were screened for 50% inhibitory concentration (IC_50_) calculation using pRRophetic (v0.5) [38]. The correlation between drug sensitivity and risk score was analyzed, and the differences of top 5 drugs with the strongest correlation were compared between high- and low- risk groups. In addition, to explore the molecular mechanisms involving prognostic genes in CRC, we constructed lncRNA-miRNA-mRNA network and TF-mRNA-miRNA network. First, we predicted the upstream miRNAs of prognostic genes using the miRDB (http://mirdb.org/miRDB/) and miranda databases. The co-predicted miRNAs were regarded as target miRNAs. We then predicted the upstream lncRNAs of target miRNAs using the Starbase database (https://starbase.sysu.edu.cn/index.phpStarBase), and constructed the lncRNA-miRNA-mRNA network using Cytoscape software. Moreover, transcription factors (TF) targeting prognostic genes were identified using the hTFtarget database (http://bioinfo.life.hust.edu.cn/hTFtarget#!/target) and combined with the target miRNAs to construct the TF-mRNA-miRNA regulatory network.

### Cell culture

The CRC cell lines SW480 and HCT-116 were obtained from the The CellBank of Type Culture Collection of Chinese Academy of Sciences (https://cellbank.org.cn) and cultured according to ATCC guidelines. SW480 and HCT-116 were rapidly thawed in a water bath maintained at 37 °C and subsequently transferred to cell culture bottles containing Dulbecco's Modified Eagle Medium (DMEM) or RPMI-1640 supplemented with 10% fetal bovine serum (FBS) and 1% penicillin–streptomycin. The cultures were incubated at 37 °C in a 5% CO2 atmosphere, with medium changes occurring every 2 to 3 days. When cell confluence reached 80–90%, the cells were rinsed with phosphate-buffered saline (PBS), treated with trypsin for detachment, and then reseeded in fresh medium at a dilution ratio of 1:3 to 1:5. For cryopreservation, the cells were suspended in a solution of 90% FBS and 10% dimethyl sulfoxide (DMSO), frozen at −80 °C, and subsequently stored in liquid nitrogen.

### Quantitative real-time PCR

Total RNA was extracted from cultured CRC cells (SW480 and HCT-116) using the Total RNA Kit (Omega Bio-Tek, USA) per the manufacturer's instructions. RNA concentration was measured with a Nanodrop ND-2000 spectrophotometer (Nano Drop Technologies, DE, USA). cDNA synthesis followed the HiScript III RT SuperMix protocol (Vazyme Biotech, China). Real-time PCR was conducted in triplicate using AceQ qPCR SYBR Green Master Mix (Vazyme Biotech, China) according to the manufacturer's guidelines. All data were normalized to the control using GAPDH as internal control.

The following primer pairs were used: RPRM, 5’-GTGTGGTGCAGATCGCAGT-3’ (Forward) and 5’-ATCATGCCTTCGGACTTGATG-3’ (Reverse); CD36, 5’- GGCTGTGACCGGAACTGTG-3’ (Forward) and 5’- AGGTCTCCAACTGGCATTAGAA-3’ (Reverse); APLP1, 5’-GCTGCCACTATTGCTGCTG-3’ (Forward) and 5’- GCTCCGGGTACATCTGTCTG-3’(Reverse); C7, 5’- AATGGCTGTACCAAGACTCAGA-3’ (Forward) and 5’-GCTGATGCACTGACCTGAAAA-3’ (Reverse); GAPDH, 5’-GGAGCGAGATCCCTCCAAAAT-3’ (Forward) and 5’-GGCTGTTGTCATACTTCTCATGG-3’(Reverse). All data were normalized to the control using GAPDH as internal control.

### Statistical analysis

All statistical analyses were conducted using R software (v4.1.0) (https://www.r-project.org/). In our study, differential comparisons between two groups were implemented using Wilcoxon's test. Differential expression analyses were performed using DESeq2 package. Gene co-expression networks were constructed using the WGCNA package. Enrichment analysis was performed using the clusterProfiler package. K-M survival curve construction was carried out using the survminer package. ROC curves were established with the survivalROC package. A nomogram was constructed using the rms package. Somatic mutations were performed with the maftools package. Drug IC50 values were calculated using the pRRophetic package. A p-value less than 0.05 was considered statistically significant. The R packages used in our study included “GEOquery”, “enrichplot”, “forestplot”, “ggplot2”, “pheatmap”, “rms” and “stats”.

## Results

### Certification of 1,752 DEGs1, 9,071 DEGs2 and 1,094 key module genes

**Fig. 1:**
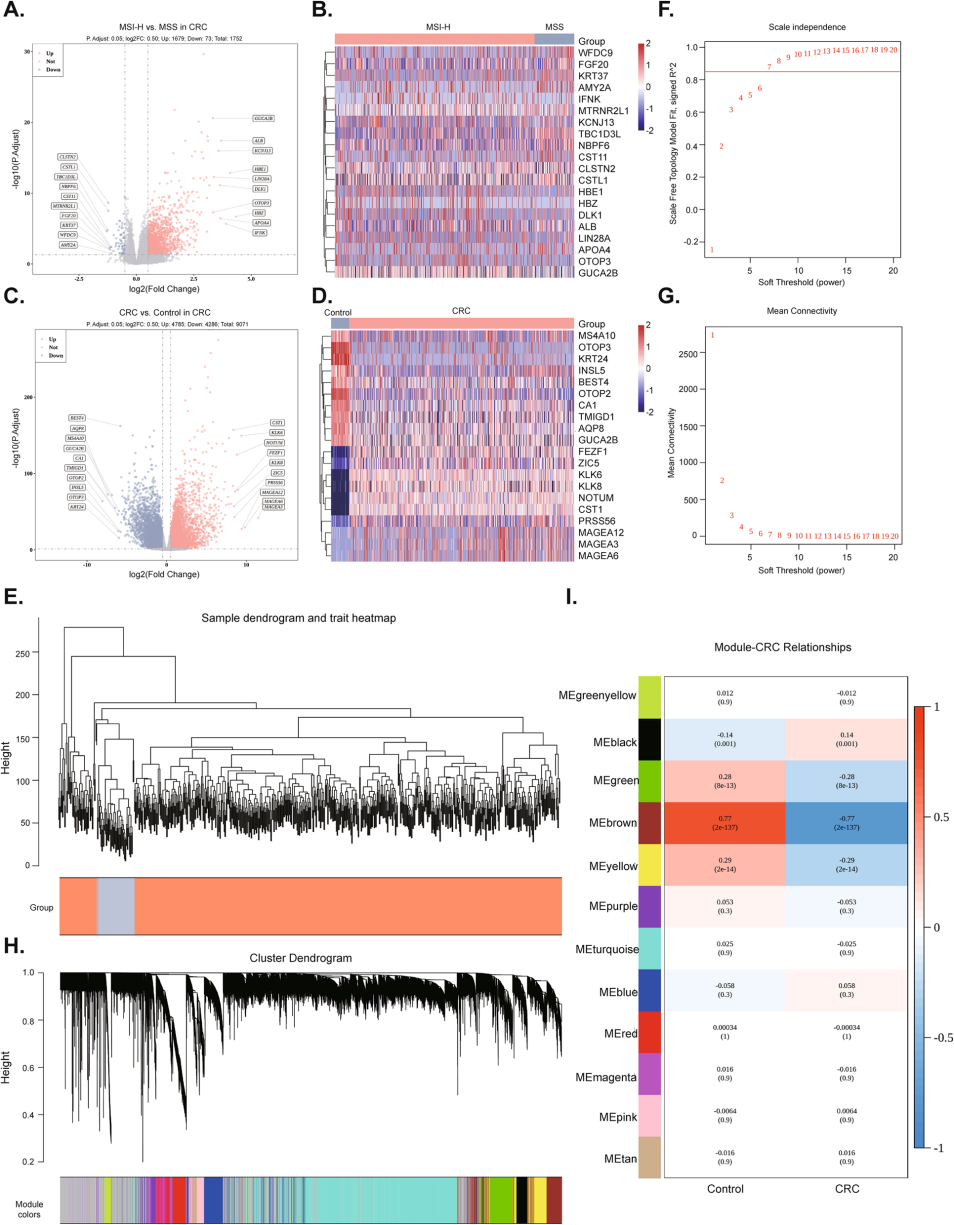
The DEGs were filtered and selected using the TCGA database, and the core gene module was screened through WGCNA. **A** Volcano plot of DEGs1. **B** Heatmap of the top 10 up- and down-regulated DEGs1. The color scale range in the heatmap is standardized based on the Z-score. The expression value of each gene is converted into a Z-score, and the calculation formula is $\text{z}=\frac{\text{x}-\upmu }{\upsigma }$ here, x represents the original expression value, μ is the mean of the gene in all samples, and σ is the standard deviation of the gene in all samples. A positive value indicates a value above the sample mean, while a negative value indicates a value below the sample mean. **C** Volcano plot of DEGs2. **D** Heatmap of the top 10 up- and down-regulated DEGs2. **E** Clustering dendrogram of samples to detect outliers. Abscissa: Sample, Ordinate: Height of clustering tree; CRC in orange, Control in grey. **F**, **G** Screening of the optimal soft-threshold values. **H** The clustering dendrograms and modules identified by WGCNA. Abscissa: Gene, Ordinate: Height of clustering tree. **I** Heatmap of correlations between modules and traits. The numbers in each square indicated the Pearson correlation coefficient (up) and *p* value (down); Red squares represent positive correlation, blue squares represent negative correlation

There were 1,752 DEGs1 and 9,071 DEGs2 in the MSI-H vs. MSS group and CRC vs. Control group, respectively (Fig. 1A, Fig. 1C, Table S1, Table S2). The heatmap showed the top 10 upregulated and downregulated DEGs1 and DEGs2 (Fig. 1B, D). In Figure S2 and Fig. 1E, the overall clustering of the dataset samples was good, and there was no need to eliminate samples. Based on the position of the red line in Fig. 1F and Fig. 1G, the power threshold was determined to be 7. At this point, the vertical coordinate R^2 was around 0.85, and the mean value of the adjacency function gradually approached 0, indicating that the network showed a flat trend. After constructing the co-expression matrix, we found that the most genes converged in the cyan module (Fig. 1H). The Brown module had the highest correlation with CRC patients, so we considered it the key module, containing 1,094 key module genes (Fig. 1I).

### The 253 intersected genes involved in multiple pathways and functions

**Fig. 2:**
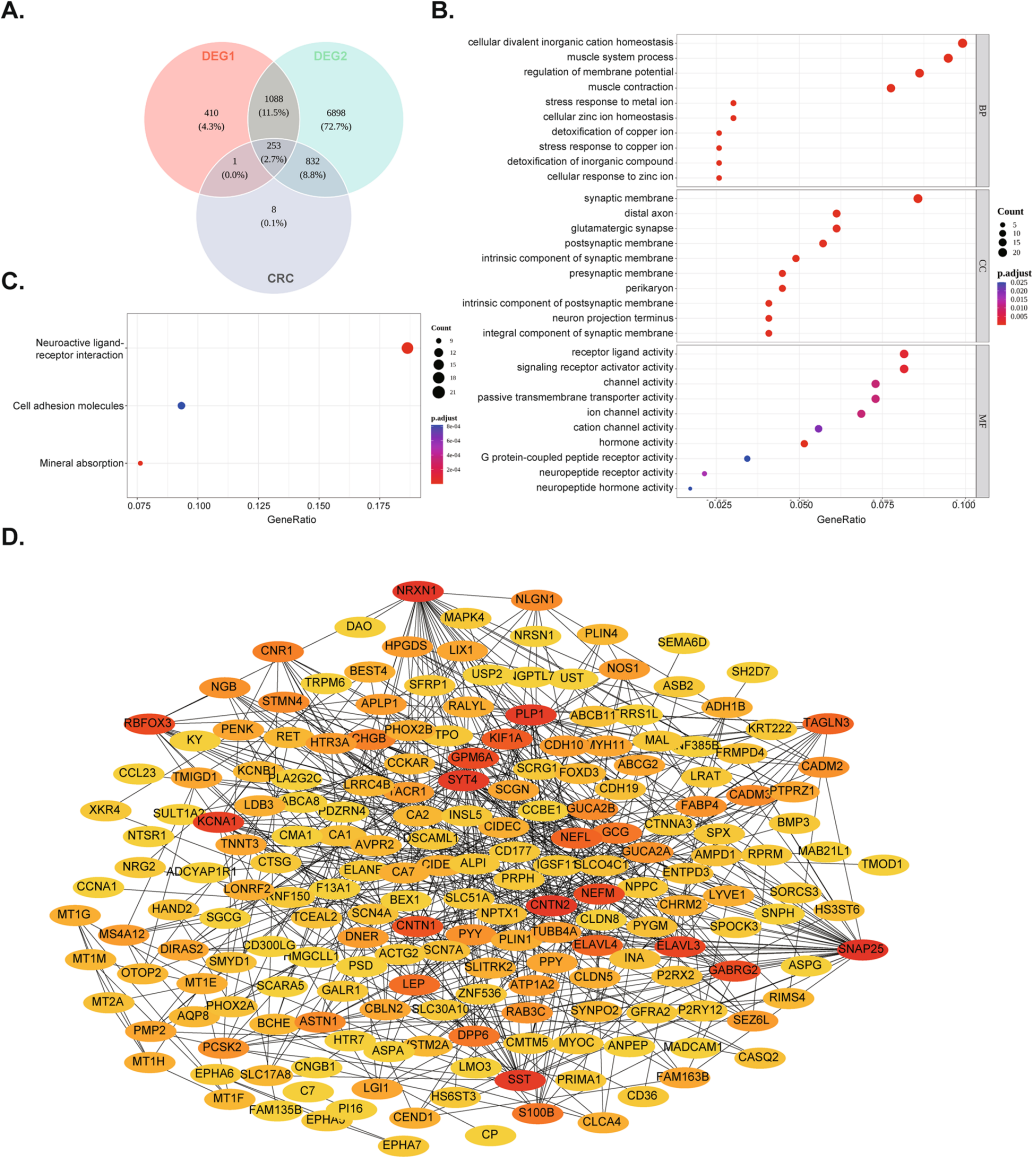
Functional enrichment analysis and construction of the PPI network. **A** The intersection genes between DEGs1 and DEGs2. **B** Bubble plots of GO enrichment analysis. **C** Bubble plots of KEGG enrichment analysis. **D** PPI network

The 253 intersected genes were obtained by taking the intersection of DEGs1, DEGs2 and key module genes (Fig. 2A). The intersected genes were involved in 226 entries, including regulation of membrane potential, presynaptic membrane, hormone activity, and more (Fig. 2B). The intersected genes were enriched to 171 KEGG pathways, such as the cAMP signaling pathway, cholesterol metabolism, and renin secretion (Fig. 2C). The PPI network consisted of 146 nodes and 504 edges, with *CNTN2* showing the strongest interaction (interacting with 24 genes) (Fig. 2D). This gene encodes a contactin family protein, part of the immunoglobulin superfamily, crucial for positioning potassium channels on nerve fibers. Mutations can cause nerve cell dysfunction and have been linked to epilepsy.

### Development of a prognostic risk model for effective prediction of CRC

**Fig. 3:**
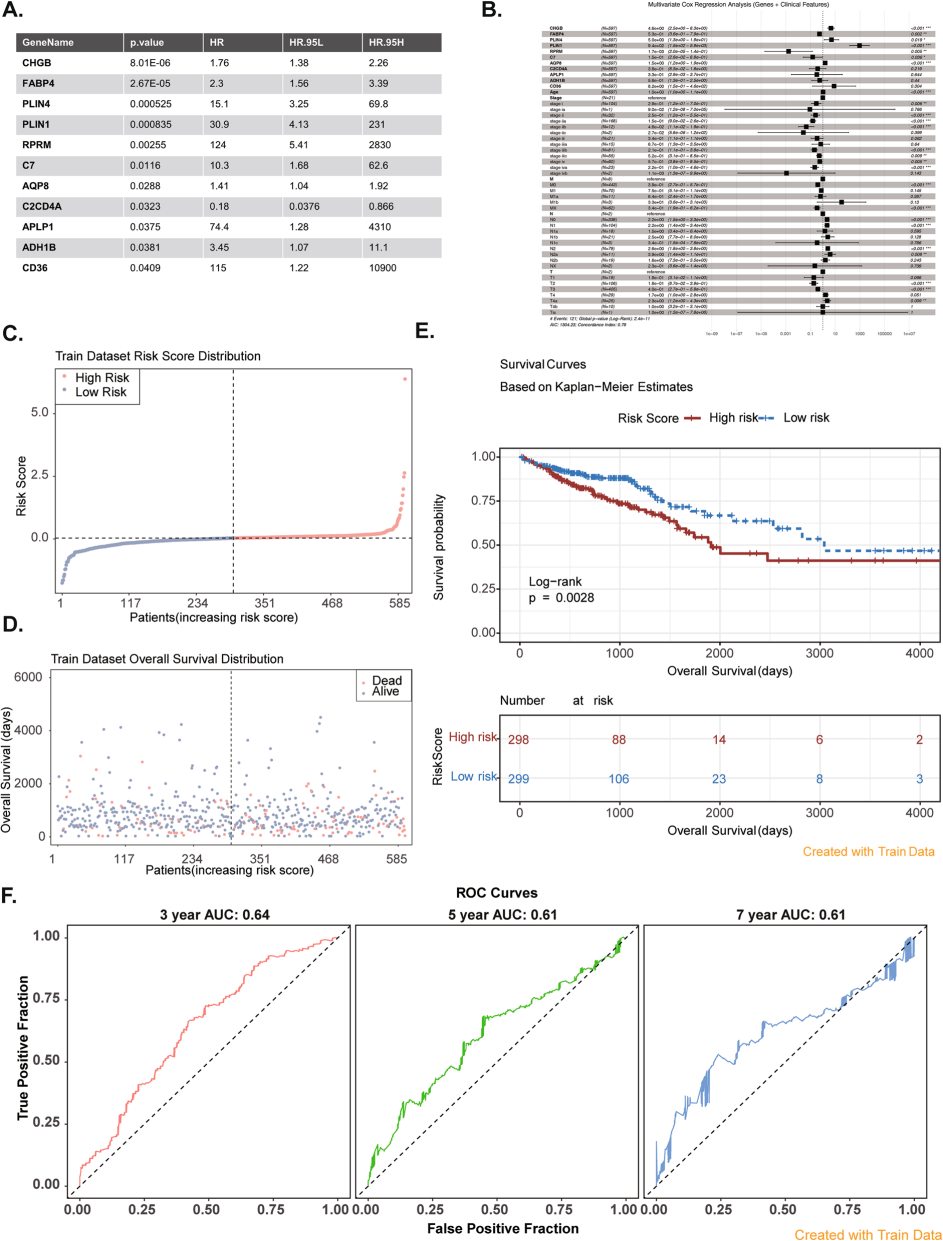
Establishment of prognostic risk model. **A** Univariate Cox regression analysis. **B** Multivariate Cox regression analysis. **C** Risk score distribution plot. **D** Overall survival distribution of train dataset. **E** Kaplan–Meier survival analyses of patients in high and low risk groups. Left: Survival curve plots probability of survival versus overall survival; Right: Risk list chart. **F** ROC curve for the 3-, 5-, and 7-years

The 11 prognostic genes, namely *CHGB*, *FABP4*, *PLIN4*, *PLIN1*, *RPRM*, *C7*, *AQP8*, *C2CD4A*, *APLP1*, *ADH1B*, and *CD36*, were identified through univariate and multivariate Cox analyses. Through tenfold cross-validation, the optimized model retained only one significant prognostic gene (FABP4) (Figure S3). A combined analysis of prognostic genes and clinical features (Age, gender, disease stage, TNM stage, etc.) revealed significant factors including AGE, N1, N2, T2, T3, MX, etc. Finally, the model as a whole is evaluated from the following aspects: The Concordance Index = 0.79 indicates that the model has excellent predictive discrimination. In the global Log-Rank test, *p* = 2.4e⁻^11^, which indicates a highly significant model. AIC = 1304.23, which indicates that the model has a good fit (Fig. 3A, B). Figure 3C shows the risk score distribution of the samples. As seen in Fig. 3D, the samples with higher overall survival (OS) were primarily clustered in the regions with lower risk scores, while the death samples were clustered in the opposite region. The K-M curve showed that the survival of the samples in the high-risk group was substantially lower than that in the low-risk group (Fig. 3E). The AUC values for 3- year (AUC = 0.64), 5-year (AUC = 0.61), and 7-year (AUC = 0.61) were all greater than 0.6, suggesting that the model had the decent predictive performance for CRC (Fig. 3F). The results from the GSE17537 and GSE106584 validation sets were consistent with those from the training set (Figs. S4, and S5). The C-index for the training set was 0.5946932, while it was 0.6136364 for the GSE17537 validation set and 0.5729508 for the GSE106584 validation set.

### Comparative gene expression analysis in CRC vs. control and MSI-H vs. MSS: insights from training and validation sets

**Fig. 4:**
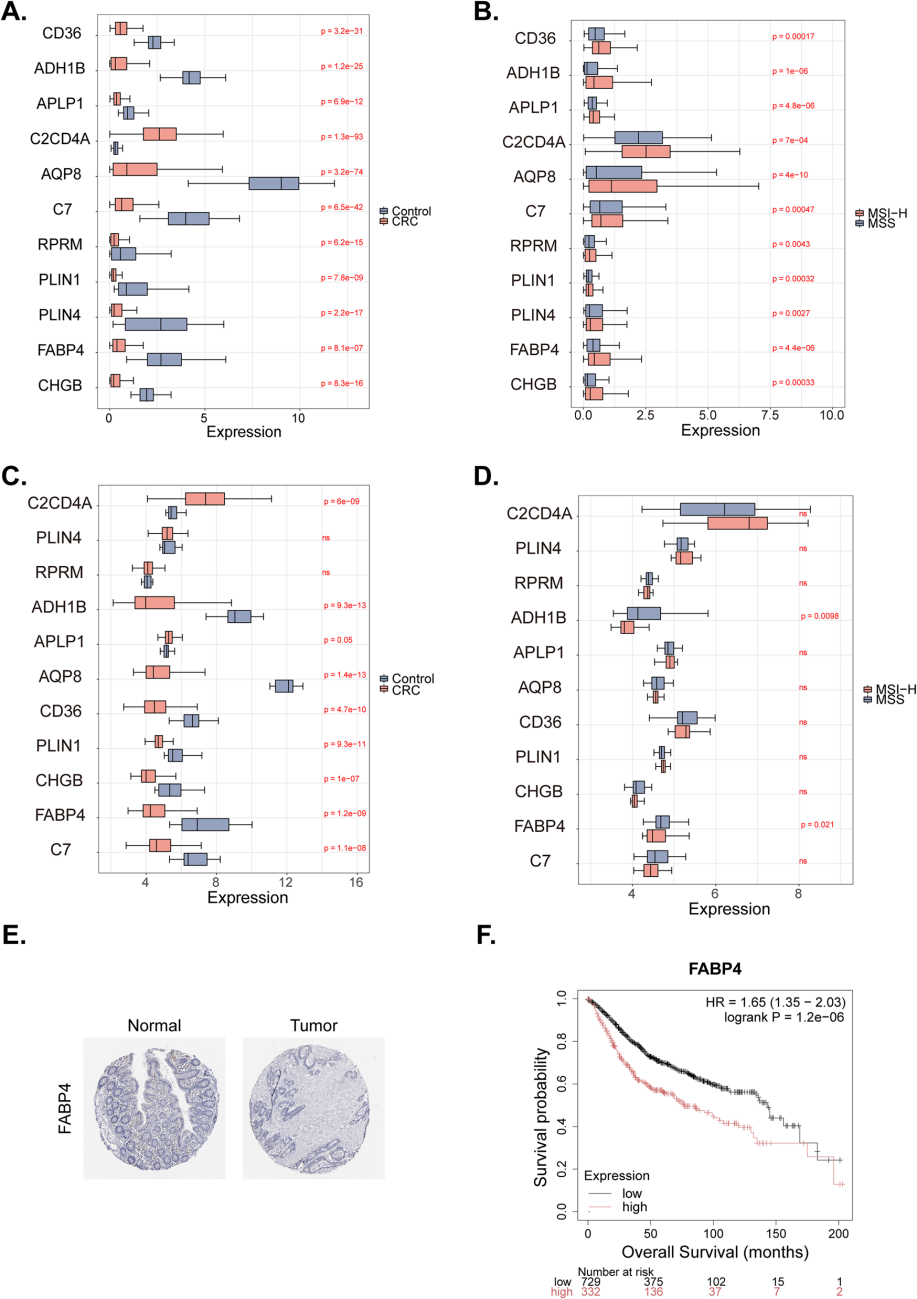
Expression of prognostic genes. **A** The expression of prognostic genes in the training set (CRC vs. Control). **B** The expression of prognostic genes in the training set (MSI-H vs. MSS). **C** The expression of prognostic genes in the validation set (CRC vs. Control). **D** The expression of prognostic genes in the validation set (MSI-H vs. MSS). **E** Immunohistochemical analysis of FABP4 proteins (tumor tissues and normal tissues). **F** Kaplan–Meier analysis of the relationship between the overall survival of CRC patients and FABP4 expression

In the training set, only *C2CD4A* was more highly expressed in CRC samples than in control samples, and all 11 prognostic genes were more highly expressed in MSI-H samples (Fig. 4A, B). In the GSE39582 validation set, *C2CD4A*, *PLIN4*, *RPRM*, and *APLP1* were more highly expressed in the CRC samples than in the control samples, while the remaining six genes showed the opposite pattern (Fig. 4C). In the GSE18088 validation set, the expression of *C2CD4A*, *APLP1*, *CD36*, and *PLIN1* was higher in MSI-H samples than in the other samples, while the remaining seven genes showed the opposite trend (Fig. 4D). We also selected four high-weighted genes (RPRM, CD36, C7, and APLP1) from the prognostic model for RT-qPCR experiments (Figure S6). The results showed that, except for C7, the other genes were highly expressed in MSI-H cells compared to MSS cell lines. Immunohistochemical analysis of prognostic gene-encoded proteins (excluding RPRM/C2CD4A due to unavailable HPA data) demonstrated significant downregulation of 9 proteins in colorectal cancer (CRC) tissues versus normal controls (*p* < 0.05), aligning with transcriptome differential results. As exemplified by FABP4 (Fig. 4E), strong protein staining in normal tissues contrasted with marked reduction in CRC, while survival analysis from the Kaplan–Meier Plotter revealed prolonged survival in the low-expression group (n = 729) versus rapid decline in the high-expression cohort (*n* = 332; *p* = 1.2e − 6)—validating its high-risk role in the prognostic model (univariate Cox HR = 2.3, *p* = 2.67e − 5) and confirming biomarker robustness (Fig. 4F).

### Construction of an effective nomogram for predicting patient survival rates

**Fig. 5:**
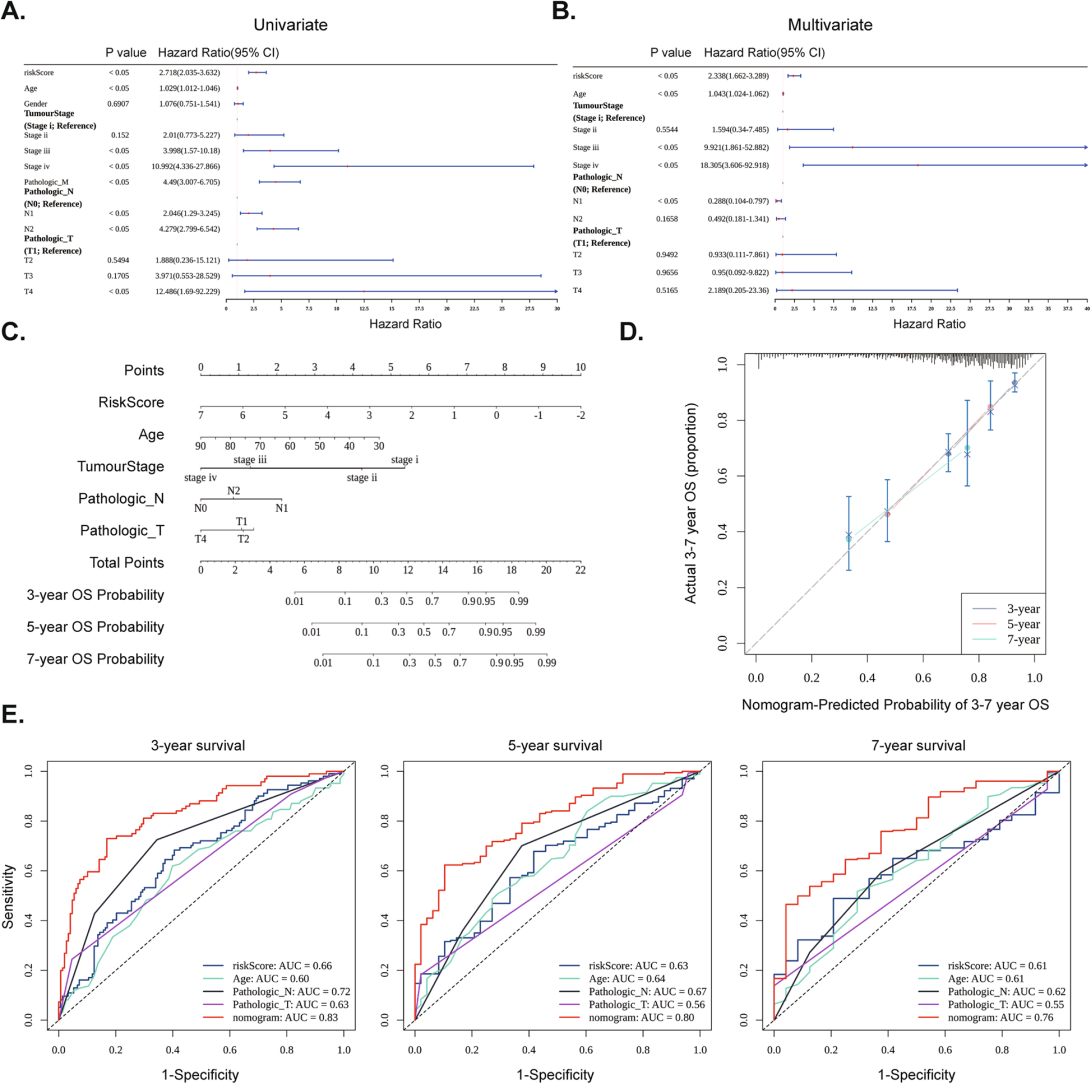
Independent prognostic factors and Nomogram construction. **A** Forest map based on the results of univariate Cox regression analysis. **B** Forest map based on the results of Multivariate cox regression analysis. **C** Nomogram. **D** Calibration curve for the nomogram. **E** ROC curve for the 3-, 5-, and 7-years

We found that Risk score, Age, Tumor Stage (Stage III, Stage IV) and Pathologic N (N1) were significantly related to survival (Fig. 5A, B). The nomogram suggested that the independent prognostic model had a good ability to predict CRC, and the calibration curve further validated the results (Fig. 5C, D). The AUC values of the nomogram at 3 years (AUC = 0.83), 5 years (AUC = 0.8), and 7 years (AUC = 0.76) were all greater than the AUC values of other single factors, illustrating the accuracy and excellent performance of the model's prediction (Fig. 5E).

### Genes in risk groups linked to multiple pathways

All genes in the two risk groups were involved in 35 pathways. The top five pathways in terms of significance were Antigen processing and presentation, Graft-versus-host disease, IL-17 signaling pathway, Inflammatory bowel disease and Natural killer cell mediated cytotoxicity. The enrichment scores of these five pathways were all less than 0, indicating that these genes were predominantly enriched at the bottom of the gene set and downregulated in the high-risk group (Figure S7). This suggests that the related genes may influence CRC patients through these pathways.

### The strong correlation between risk score and TIME of the CRC

**Fig. 6:**
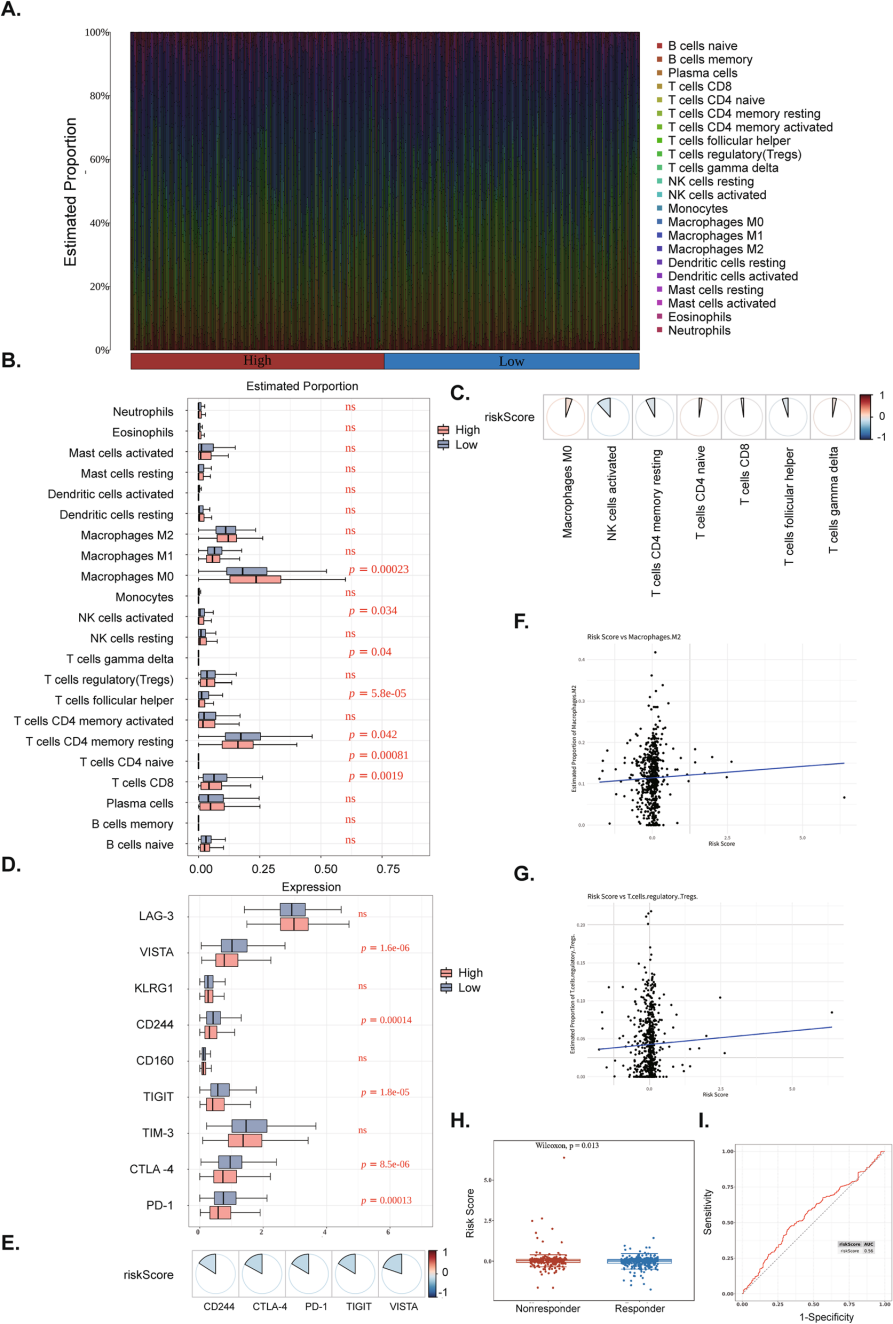
Immune microenvironment analysis. **A** The percentage abundance of immune cells in tumor samples. **B** Immune cell abundance differences between the high- and low-risk groups. **C** Heatmaps of the correlation diagrams of 7 immune cells and risk score. Pie chart size represents the magnitude of the correlation coefficient; Red is positive correlation and blue is negative correlation. **D** Immune checkpoint expression analysis between the high- and low-risk groups. **E** Heatmaps of the correlation diagrams of 5 immune checkpoints and risk score. Pie chart size represents the magnitude of the correlation coefficient; Red is positive correlation and blue is negative correlation. **F** Correlation analysis chart between risk score and M2-type macrophage infiltration. **G** Correlation analysis chart of risk score and regulatory T cell (Treg) infiltration. **H** Differences in risk score between the responder and non-responder groups. **I** ROC curve: the role of risk score in the prediction of immunotherapy response

Figure 6A illustrates the infiltration abundance of immune cells in tumor samples. Seven immune cells, including M0 macrophages, follicular helper T cells, and CD8 T cells, showed significant difference in infiltration abundance percentage between risk groups (Fig. 6B). The correlation heatmap indicated that only activated NK cells had a significant negative correlation with the risk score (Fig. 6C). Five immune checkpoints-*CD244*, *PD-1*, *CTLA-4*, *TIGIT*, and *VISTA*- differed notably between risk groups and all negatively correlated with the risk score (Fig. 6D, E). As shown in Figs. 6F and 6G, the blue line represents the linear regression fitting line, which elucidates the correlation trend between the risk score and the abundance of infiltration of two types of immunosuppressive cells. The study identified a positive correlation between the risk score and the degree of infiltration of these immunosuppressive cells. This finding suggests that a high-risk score may be associated with an increased presence of immunosuppressive cells within the tumor microenvironment, potentially contributing to the elevated expression of immune checkpoints. Figure S8 shows the response ratio between risk groups. The box plot demonstrated a significant difference in risk scores between the responder and non-responder groups (Fig. 6H). The AUC of risk score was 0.56, suggesting its efficacy in predicting response to immunotherapy (Fig. 6I).

### Comparison of somatic mutation and TMB in risk model

**Fig. 7:**
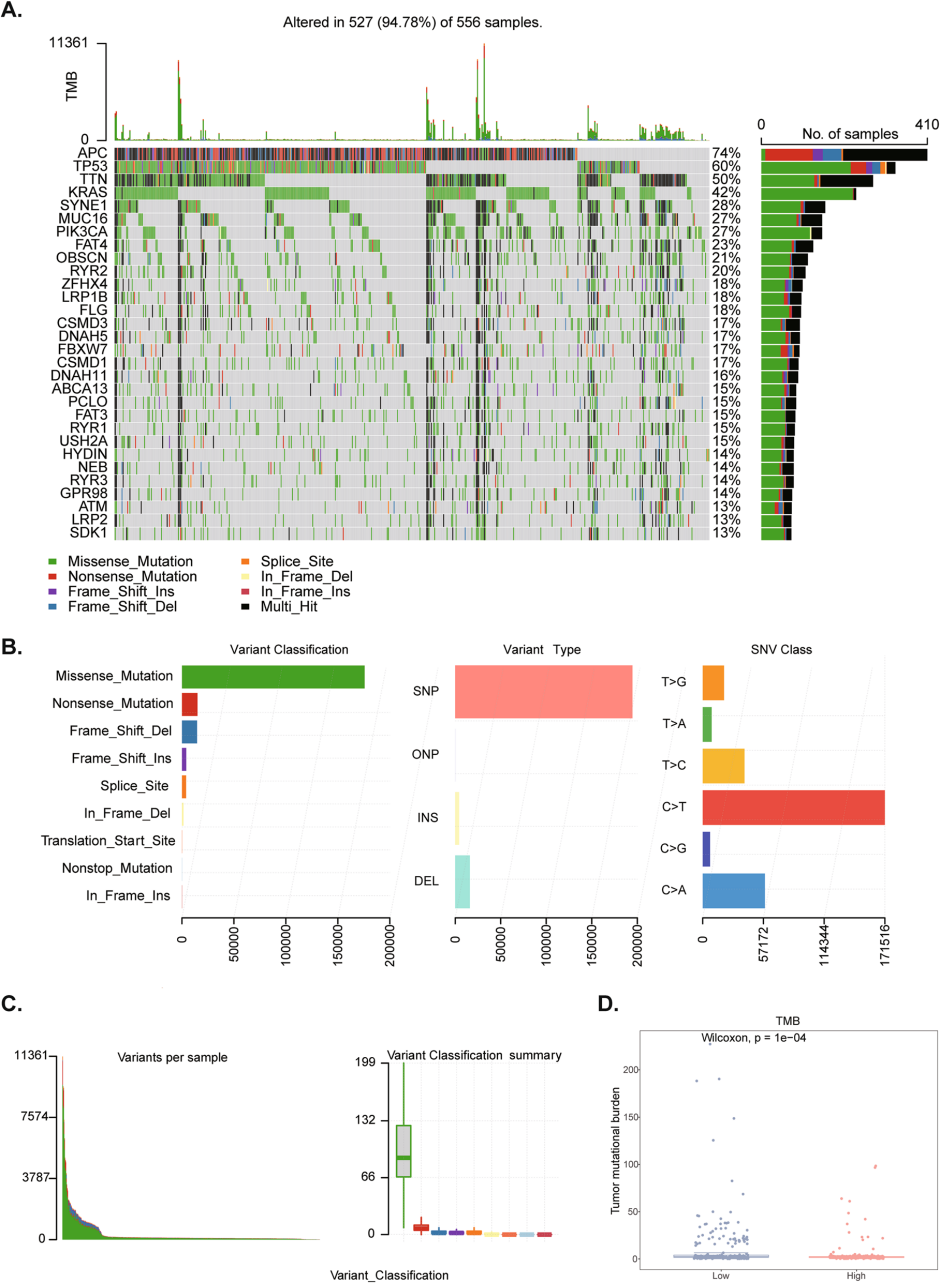
Somatic cell mutation analysis. **A** Waterfall chart of somatic mutations of CRC samples. **B**, **C** Statistical map of gene mutation types. **D** Comparison of TMB between risk groups

The waterfall plot showed the top 30 mutated genes in the CRC samples, with APC having the highest mutation frequency. Besides, we found that among the mutation types, nonsense mutation occurred mainly in APC, while missense mutation occurred mainly in the other 29 genes (Fig. 7A). Missense mutations were the most frequent, and the most common single nucleotide mutation was a C to T transition (Fig. 7B, C). There were 512 samples with TMB information, and the TMB value differed significantly between high- and low-risk groups (Fig. 7D).

### Prediction of potential therapeutic drugs and regulatory role

The drug sensitivity of Bleomycin, Gemcitabine, Doxorubicin, Etoposide and Cisplatin was positively correlated with risk score, with Bleomycin showing the strongest correlation (Figure S9A-E). The sensitivity of these five drugs differed significantly between the high- and low-risk groups (Figure S9F). Additionally, a total of 17 target miRNAs were predicted based on the miRDB and miranda databases, and 30 lncRNAs were predicted using the Starbase database. A lncRNA-miRNA-mRNA network was then constructed, including 3 prognostic genes (*C7, CHGB, RPRM*), 12 miRNAs, and 30 lncRNAs (Figure S10A). Furthermore, 19 TFs were predicted using the hTFtarget database, and a TF-mRNA-miRNA network, including 2 prognostic genes (*CHGB, RPRM*), 19 TFs, and 17 miRNAs, was constructed (Figure S10B). It suggests that *CHGB* and *RPRM* are regulated by non-coding RNAs and TFs, which may influence the occurrence and development of CRC.

## Discussion

Currently, adjuvant chemotherapy is exclusively recommended for non-metastatic CRC patients with a high risk of recurrence, leading to overtreatment of a significant number of low-risk patients [24, 39]. Therefore, there is an urgent need for a more precise prognostic system to effectively strategize the treatment and follow-up care of CRC patients. Despite the effectiveness of MSI as a prognostic indicator of immune response, its ability to predict the prognosis of CRC patients is limited. Furthermore, the applicability of microsatellite (MS) status in stage III CRC remains a contentious issue.

In this study, a total of 11 prognostic genes (*CHGB*, *FABP4*, *PLIN4*, *PLIN1*, *RPRM*, *C7*, *AQP8*, *C2CD4A*, *APLP1*, *ADH1B*, and *CD36*) were identified through univariate and multivariate Cox regression analyses. The prognostic risk model was evaluated and verified using KM and ROC curves, while the calibration curve and ROC curve were utilized to assess the prognostic model. The C-index and time-dependent ROC analysis serve as comprehensive indicators for assessing model performance, facilitating an initial evaluation of the model's predictive capabilities [40, 41]. Nevertheless, the model’s AUC value in this study is moderate, potentially due to factors such as a limited sample size, significant data noise, or an incomplete capture of all pertinent features. Additionally, the performance of the model may be influenced by intrinsic factors associated with colorectal cancer, such as MSI status and immune checkpoint expression. Despite these limitations, the novel potential biomarkers identified in this study offer valuable directions for future research.

Complement component 7 (*C7*) is a crucial constituent of the innate immune system, belonging to the complement system, which comprises natural complement components, complement control proteins, and complement receptors [42, 43]. Encoded by the *C7* gene, *C7* is a 93 kDa serum glycoprotein that interacts with other terminal complement components (*C5b*, *C6*, *C8*, and *C9*) to form a membrane attack complex (MAC), the cytolytic effector unit of the complement system. Its pivotal role in coordinating innate and adaptive immune responses underscores its significance in immunology. Recent findings suggest that *C7* plays a role in the progression of various malignancies [42–44]. Specifically, *C7* expression is elevated in normal tissues but significantly diminished in carcinoma tissues of human esophageal, colonic, and renal cancers, as well as non-small cell lung cancer [42, 45]. Furthermore, *C7* mRNA expression levels show a gradual decline from normal to benign, borderline, and malignant ovarian tissues, and *C7* expression is inversely correlated with tumor grade in ovarian cancer patients [42]. Conversely, some researchers argue that the presence of *C7* may contribute to cancer progression [22, 42]. This assertion is supported by a study that observed increased *C7* expression in ovarian cancer and a subsequent reduction in ovarian cell proliferation following C7 suppression. Additionally, maintaining of stemness in liver tumor-initiating cells required a significant upregulation of *C7* protein [44]. However, the current investigation revealed a significant downregulation and selective expression of *C7* in CRC tissues. Notably, low *C7* expression was associated with shorter disease-free survival (DFS) and OS, indicating its potential as a prognostic indicator. Importantly, multivariate Cox analysis demonstrated that *C7* may serve as a positive prognostic indicator for OS.

Prior research has indicated that *C2CD4A*, a gene that encodes a nuclear protein induced by *IL-1β*, exhibits increased expression in CRC tissues compared to adjacent normal colorectal tissues. In vitro experiments demonstrated that the suppression of *C2CD4A* significantly stimulated cell apoptosis and inhibited proliferation, while in vivo experiments showed that it reduced tumorigenicity. Conversely, overexpression of *C2CD4A* resulted in the opposite effects. *C2CD4A* fundamentally impeded the *p53* signaling pathway by interacting with *p53* and augmenting its ubiquitination and degradation [46]. In contrast, our study demonstrated that CRC patients with high C2CD4A expression exhibited longer OS. Additional investigations are necessary to elucidate the precise association between *C2CD4A* and CRC prognosis.

*PLIN1* and *PLIN4* are integral members of the Perilipin (*PLIN*) family, which play a crucial role in the formation and degradation of lipid droplets (*LDs*). The accumulation of *LDs* appears to be a prevalent characteristic in various cancers, including CRC [47, 48]. In CRC, *LDs* are believed to function as protective organelles against the toxicity resulting from an excess of reactive oxygen species and free lipids, as well as energy suppliers and sites for the synthesis of inflammatory mediators. As a result, *LDs* promote the survival, aggressiveness, and treatment resistance of cancer stem cells [48]. Nonetheless, the current body of literature on the correlation between the *PLIN* family and tumors is limited, and there is no conclusive evidence to support the notion that the *PLIN* family plays a role in tumorigenesis and progression via modulation of *LD* metabolism. Our findings indicate a significant association between high levels of *PLIN1* or *PLIN4* and poor prognosis in CRC, highlighting the predictive role of lipid droplets in CRC prognosis.

The maintenance of cellular homeostasis necessitates the regulation of lipid metabolism, including lipid uptake, synthesis, and hydrolysis. One of the most notable metabolic changes in cancer is the dysregulation of lipid metabolism. Cancer cells exploit lipid metabolism to acquire energy, components for biological membranes, and signaling molecules that are indispensable for proliferation, survival, invasion, metastasis, and adaptation to the tumor microenvironment and cancer treatment [49, 50]. Research has demonstrated that established *FA* protein transporters present in the plasma membrane, such as *CD36* (*FA* translocator), *SLC27* (*FA* transporter family), and *FABPs* (plasma membrane *FA* binding proteins), among others, exhibit up-regulation in cancer. Furthermore, the heightened expression of *CD36* has been linked to unfavorable prognoses in metastatic breast cancer, ovarian cancer, gastric cancer, prostate cancer, and oral squamous cell carcinoma [51, 52]. Fatty acid Binding protein 4 (*FABP4*), a constituent of the *FABP* family, is predominantly expressed in adipocytes and has the potential to be secreted into the bloodstream. It is hypothesized that *FABP4* concentrations may serve as a valuable circulating Biomarker for certain metabolic disorders, including obesity, metabolic syndrome, and type 2 diabetes. *FABP4* is implicated in the regulation of gene expression, cell proliferation, differentiation, and signal transduction in the context of tumor metastasis [53, 54]. An investigation into ovarian cancer revealed that the absence of *FABP4* is linked to the metastasis of ovarian cancer, and targeting *FABP4* in ovarian cancer cells can impede their capacity to adapt and colonize lipid-rich tumor microenvironments [55]. Studies have discovered that the upregulation of *FABP4* in CRC tissues could stimulate tumor growth by activating the fatty acid oxidation pathway [54, 56]. Our investigation demonstrated that both *CD36* and *FABP4* could serve as unfavorable prognostic markers for both DFS and OS. This evidence suggests that lipid transporters may exert a crucial influence on the prognosis of CRC. Further research will be conducted to elucidate the role of lipid metabolism in CRC.

*AQP8*, a member of the Aquaporins (AQPs) family, functions as an integral membrane protein that forms channels to facilitate the transmembrane diffusion of water and various small solutes. *AQPs* are involved in cellular trafficking and numerous physiological processes [57]. The available evidence suggests that *AQP8* plays a role in tumorigenesis, specifically in tumor cell migration, angiogenesis, and tumor growth. Notably, reduced expression of *AQP8* has been associated with increased resistance to apoptosis in hepatocellular carcinoma, ovarian cancer, and breast cancer [58]. Previous studies have demonstrated reduced *AQP8* expression levels in human CRC [58]; however, there is a dearth of literature on the relationship between *AQP8* and the prognosis of CRC. The present study utilized univariate and multivariate Cox analysis to demonstrate a negative correlation between AQP8 expression and CRC prognosis, indicating its negative predictive value. However, further investigations are warranted to expand upon these findings.

Limited research has been conducted on the prognostic significance of several genes, namely *APLP1*, *ADH1B*, *CHGB*, and *RPRM* in CRC. *APLP1*, a member of the APP-related protein family, is a neuron-specific, membrane-bound protein that participates in various cellular processes such as apoptosis, cell adhesion, endocytosis, cell signaling, and neurite extension. Several studies have indicated that the overexpression of *APLP1* is significantly linked to a lower OS rate in the early stages of clear cell renal cell carcinoma, thus suggesting its potential as a biomarker for the aggressiveness and prognosis of the disease [59]. Reprimo (*RPRM*), a constituent of the *RPRM* gene family, functions as a tumor-suppressor gene that modulates the *p53*-mediated cell cycle arrest at G2/M. The epigenetic silencing of the *RPRM* gene through promoter methylation is responsible for the loss of *RPRM* expression, which in turn promotes tumor cell proliferation and migration in various cancers such as stomach cancer, breast cancer, pituitary adenocarcinoma, and CRC [60, 61]. Consequently, RPRM is anticipated to play a role in the response to DNA damage repair pathways. The ADH1B protein is involved in the primary pathway of ethanol metabolism, while the polymorphic site rs3811802, located in the promoter region of the *ADH1B* gene, has been identified as a risk factor for CRC in the Chinese Han population [62, 63]. To identify pulmonary tumors with neuroendocrine differentiation, it is recommended to utilize Chromogranin (*CHGB*), Synaptophysin, and *CD56* as markers [64]. Furthermore, although only a small percentage of patients with pancreatic neuroendocrine tumors exhibit elevated serum levels of pancreastatin and *CHGB*, ranging from 17 to 57%, their clinical significance remains uncertain. This study found that all four genes were significantly associated with a negative prognosis in CRC patients, serving as reliable indicators of poor prognosis (with the exception of *APLP1* gene, which demonstrated a HR < 1 in the multivariate COX analysis).

Remarkably, the prognostic risk model genes identified in this investigation did not include DNA mismatch repair genes. Furthermore, all genes in the high- and low-risk group related to 35 pathways, such as antigen processing and presentation, the *IL-17* signaling pathway, inflammatory bowel disease, natural killer cell mediated cytotoxicity, and others. These findings suggest that dMMR systems are not entirely interchangeable with MS in forecasting the prognosis of CRC.

The risk score exhibits a positive correlation with M0 macrophages, naive CD4 T cells, and gamma delta T cells, while other immune cells show negative correlations with the risk score, consistent with prior research. Five immune checkpoints, like *CTLA-4*, *TIGIT*, and *VISTA*, exhibited significant distinctions between high and low expression cohorts, and they were inversely associated with risk score. This implies that immune response is linked to prognosis, whereby a heightened response corresponds to a more favorable prognosis. Research indicates that within the tumor microenvironment of high-risk colorectal cancer, continuous antigen stimulation induces the differentiation of CD8⁺ T cells into a terminally exhausted (Tex) state [65]. Initially, these cells exhibit high expression levels of inhibitory receptors, including PD-1 and CTLA-4; however, their effector functions, such as IFN-γ secretion, progressively deteriorate due to mitochondrial dysfunction and a decline in oxidative phosphorylation (OXPHOS) capacity [66, 67]. This process is further characterized by epigenetic remodeling, including the upregulation of Eomes and downregulation of TCF7, leading Tex cells to experience metabolic collapse. Consequently, these cells develop a phenotype marked by low expression of immune checkpoints and functional paralysis [68]. This phenomenon may elucidate the observed negative correlation between risk scores and immune checkpoint expression. Nevertheless, the contribution of immune response to prognosis is relatively limited. The association between high TMB and a suboptimal immune response contradicts previous understanding, necessitating further investigation into the connection between immune response and prognosis.

This study has several limitations. Although the prognostic gene expression of *CHGB*, *FABP4*, *PLIN4*, *PLIN1*, *RPRM*, *C7*, *AQP8*, *C2CD4A*, *APLP1*, *ADH1B*, and *CD36* shows potential in predicting CRC prognosis, further research is needed to elucidate the regulatory mechanisms underlying the prognostic risk model. A significant limitation of this study is the lack of experimental validation, particularly concerning drug sensitivity. Due to existing constraints, such validations were not conducted; however, future investigations will prioritize the assessment of predicted drug sensitivities in CRC cell lines and animal models. Moreover, the relatively small sample size, despite using publicly available datasets, may not fully capture the heterogeneity of CRC. Expanding the sample size through additional cohorts or prospective studies will be essential to improve the robustness and generalizability of our results.

## Conclusion

This study developed a prognostic risk model based on microsatellite stability-associated genes that effectively predicts survival in CRC patients. We identified 11 key genes linked to the tumor immune microenvironment, clinicopathological features, and patient outcomes. The model demonstrated strong predictive performance, providing valuable insights for prognosis prediction and personalized treatment strategies in CRC. However, there are limitations such as a small sample size and suboptimal model performance (e.g., AUC value). Future studies need to expand the sample size, optimize data collection strategies, further validate these findings, and explore their clinical application.

## Supplementary Information

Supplementary Material 1. Figure S1 The flow chart of this study

Supplementary Material 2. Figure S2 Sample clustering to detect outliers

Supplementary Material 3. Figure S3 Cross-validate the error curve

Supplementary Material 4. Figure S4 Validation of the established prognostic risk model using GSE17537 dataset. (A) Risk score distribution of GSE17537dataset. (B) Overall survival distribution of GSE17537dataset. (C) Kaplan–Meier survival analyses of patients in high and low risk groups based on GSE17537. Upper: Survival curve plots probability of survival versus overall survival; Bottom: Risk list chart. (D) Heatmap of expression profiles of genes in the prognostic model. (E) ROC curve for the 3-, 5-, and 7-years

Supplementary Material 5. Figure S5 Validation of the established prognostic risk model using GSE106584 dataset. (A) Risk score distribution of GSE106584 dataset. (B) Overall survival distribution of GSE106584 dataset. (C) Kaplan–Meier survival analyses of patients in high and low risk groups based on GSE106584. Upper: Survival curve plots probability of survival versus overall survival; Bottom: Risk list chart. (D) Heatmap of expression profiles of genes in the prognostic model. (E) ROC curve for the 3-, 5-, and 7-years

Supplementary Material 6. Figure S6 The differential expression of multiple prognostic genes in MSI-H and MSS colorectal cancer cells was analyzed using qPCR. Statistical significance is indicated at *p < 0.05 vs. MSS, ****p < 0.0001 vs. MSS, respectively

Supplementary Material 7. Figure S7 Results of enrichment analysis of GSEA. The curves represented the connecting lines of the enrichment scores of each gene inside the pathway, different colours indicate different pathways. The figure showed the enriched pathways for the top 5

Supplementary Material 8. Figure S8 Immune response in high and low risk groups

Supplementary Material 9. Figure S9 Drug sensitivity analysis. (A-E) Correlation analysis between drug sensitivities and risk scores. A: Cisplatin; B: Etoposide; C: Doxorubicin; D: Gemcitabine; E: Bleomycin. (F) Variations in drug sensitivity between risk groups

Supplementary Material 10. Figure S10 Molecular network. (A) LncRNA-miRNA-mRNA network. Orange denotes lncRNAs, blue denotes miRNAs, and green denotes prognostic genes. (B) TF-mRNA-miRNA network. Blue represents transcription factors (TF), green represents miRNAs, and pink represents prognostic genes

Supplementary Material 11.

## Data Availability

Publicly available datasets were analyzed in this study, these can be found in The Cancer Genome Atlas (https://portal.gdc.cancer.gov/) database (TCGA-CRC cohort) and the Gene Expression Omnibus (https://www.ncbi.nlm.nih.gov/geo/) database (accession numbers: GSE17537, GSE18088 and GSE39582).

## Abbreviations

CRC: Colorectal cancer
UCSC: University of California Santa Cruz
MSS: Microsatellite stable
MSI: Microsatellite instability
MSI-H: High microsatellite instability
DEGs: Differentially expressed genes
WGCNA: Weighted gene co-expression network analysis
IARC: The International Agency for Research on Cancer
AJCC: The American Joint Committee on Cancer
OS: Overall survival
MMR: Mismatch repair;
dMMR: MMR-deficient
pMMR: MMR-proficient
ORR: Objective response rate
GO: Gene ontology
KEGG: Kyoto encyclopedia of genes and genomes
PPI: Protein-protein interaction
ROC: Receiver operating characteristic
GSEA: Gene set enrichment analysis
CIBERSORT: Cell type identification by estimating relative subsets of RNA transcripts
TIDE: Tumor immune dysfunction and exclusion
TMB: Tumor mutational burden
IC50: 50% Inhibitory concentration
K-M: Kaplan-Meier
*C7*: Complement component 7
*PLIN*: Perilipin
*LDs*: Lipid droplets
*FABPs*: Fatty acid binding proteins
*AQPs*: Aquaporins
*RPRM*: Reprimo
*CHGB*: Chromogranin
*CD244*: Also known as *2B4\NAIL\Nmrk\NKR2B4\SLAMF4*
*PD-1*: Programmed cell death 1
*CTLA-4*: Cytotoxic T-lymphocyte associated protein 4
*TIGIT*: T cell immunoreceptor with Ig and *ITIM* domains
*VISTA*: V-set immunoregulatory receptor
