# Supplementary figures and images for: Development of a prognostic risk model for colorectal cancer based on microsatellite stability-associated genes
Source: BMC Cancer. 2025 Oct 1;25:1490. doi: 10.1186/s12885-025-14918-y (PMC12487216; doi:10.1186/s12885-025-14918-y)

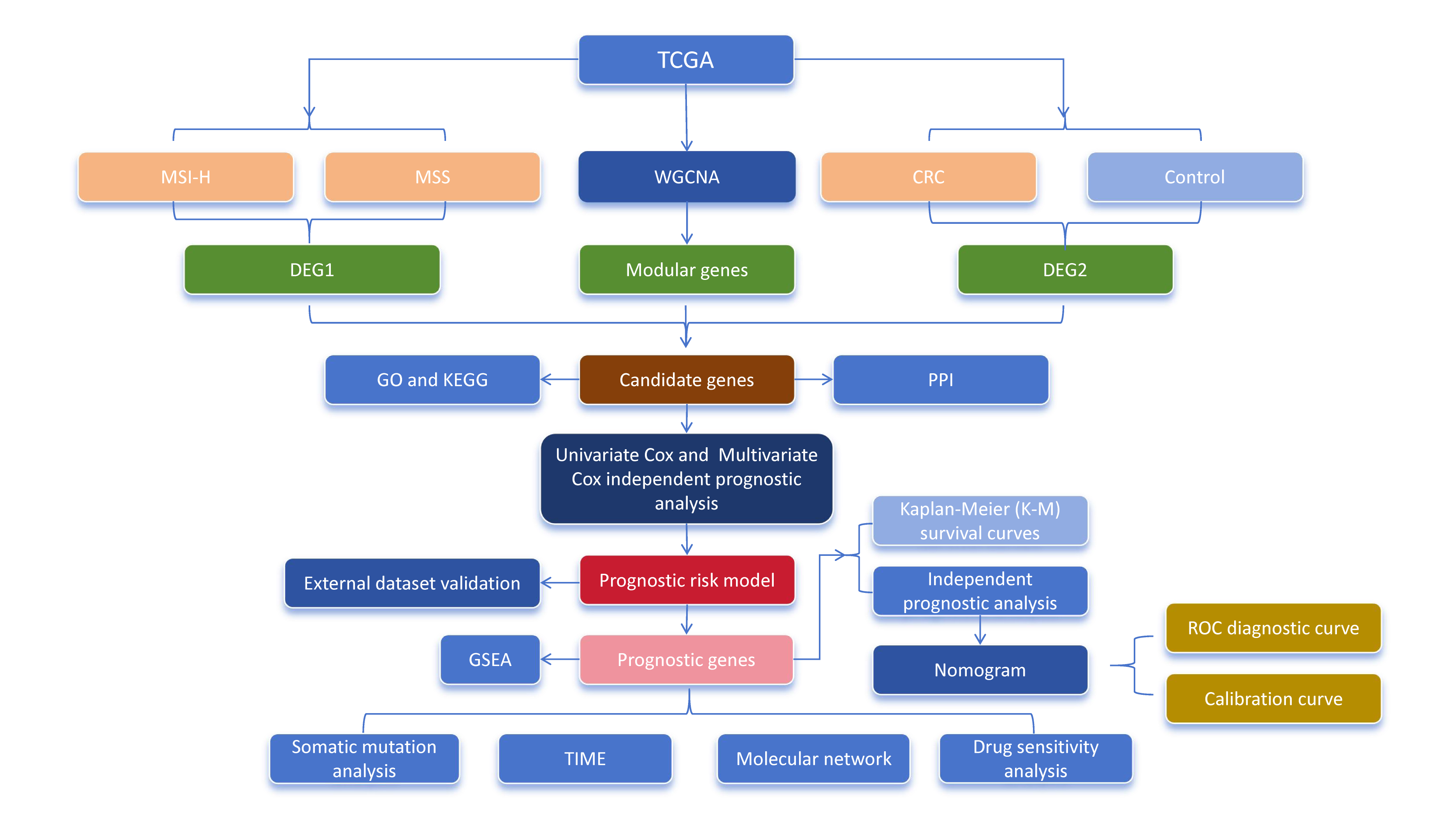

Supplement: Supplementary file 1 — Supplementary Material 1. Figure S1 The flow chart of this study [file 12885_2025_14918_MOESM1_ESM.tif]

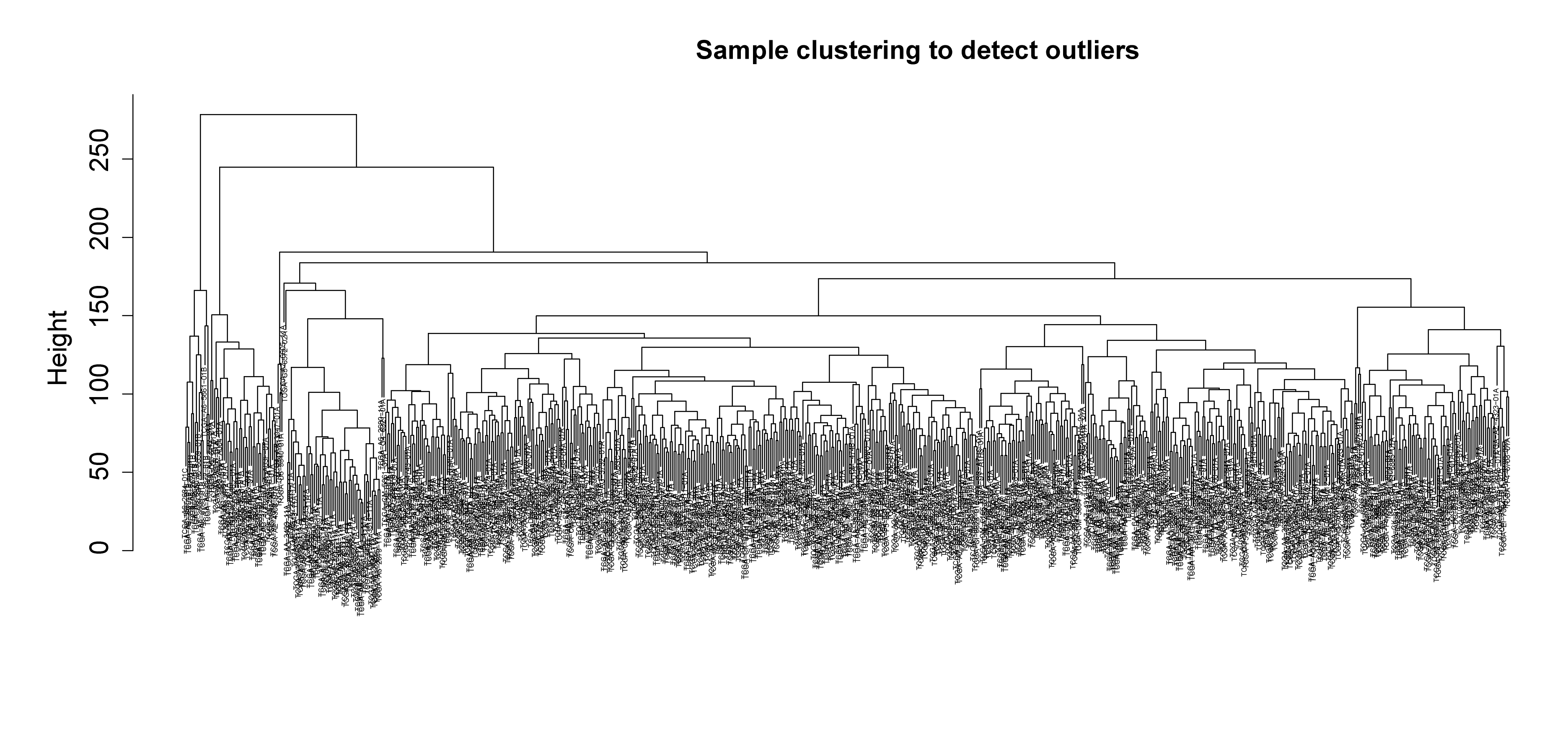

Supplement: Supplementary file 2 — Supplementary Material 2. Figure S2 Sample clustering to detect outliers [file 12885_2025_14918_MOESM2_ESM.tif]

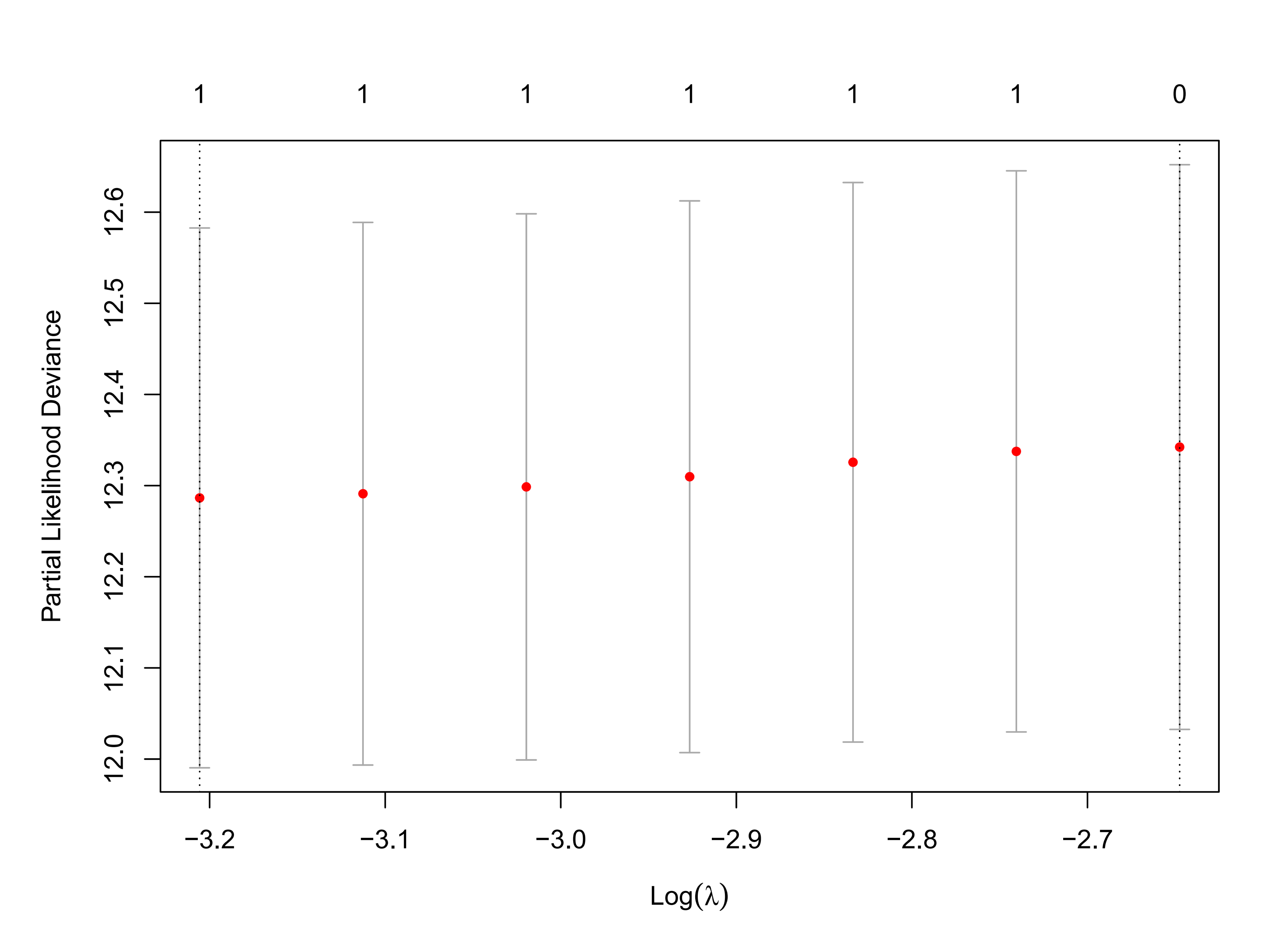

Supplement: Supplementary file 3 — Supplementary Material 3. Figure S3 Cross-validate the error curve [file 12885_2025_14918_MOESM3_ESM.tif]

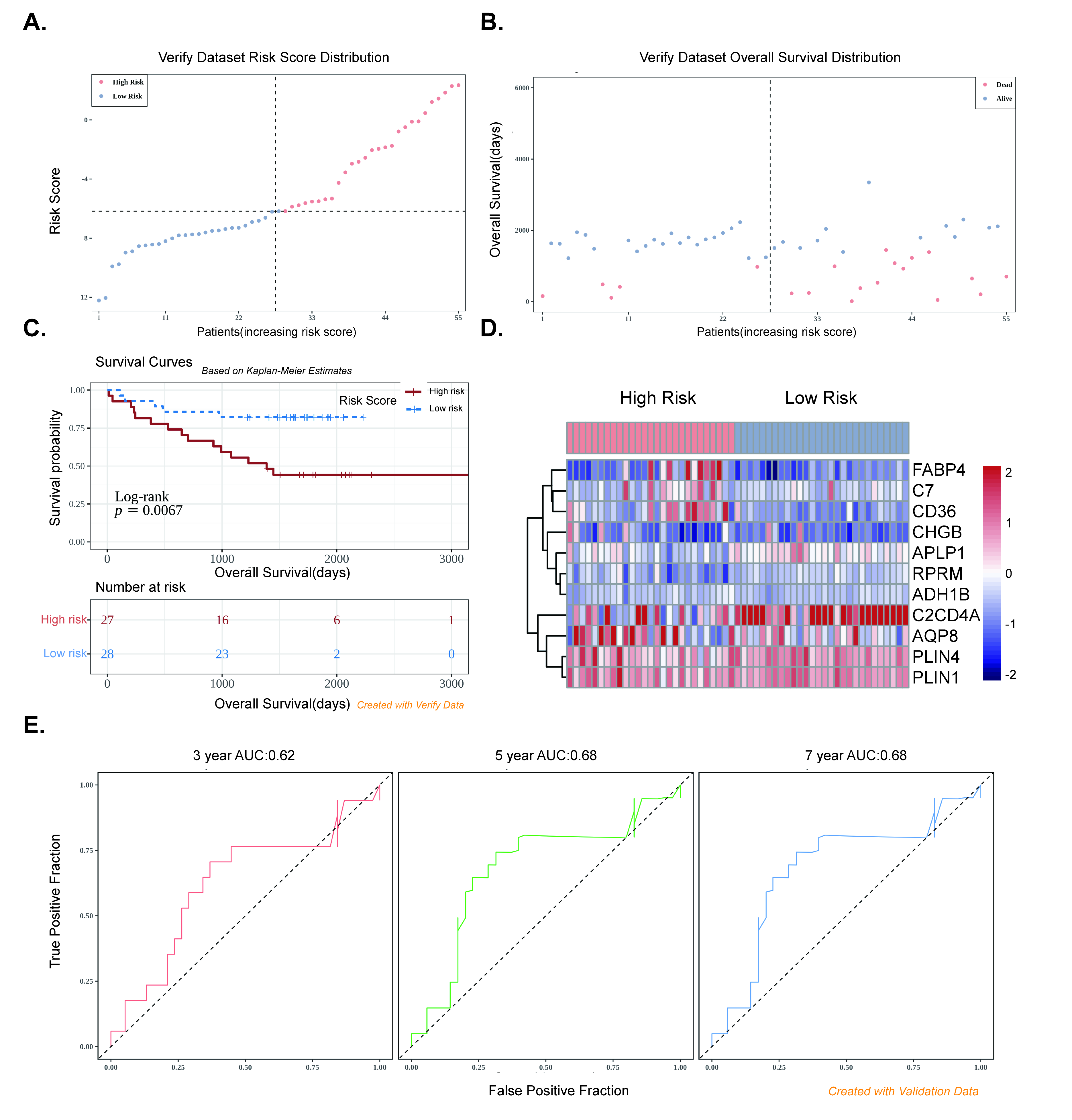

Supplement: Supplementary file 4 — Supplementary Material 4. Figure S4 Validation of the established prognostic risk model using GSE17537 dataset. (A) Risk score distribution of GSE17537dataset. (B) Overall survival distribution of GSE17537dataset. (C) Kaplan–Meier survival analyses of patients in high and low risk groups based on GSE17537. Upper: Survival curve plots probability of survival versus overall survival; Bottom: Risk list chart. (D) Heatmap of expression profiles of genes in the prognostic model. (E) ROC curve for the 3-, 5-, and 7-years [file 12885_2025_14918_MOESM4_ESM.tif]

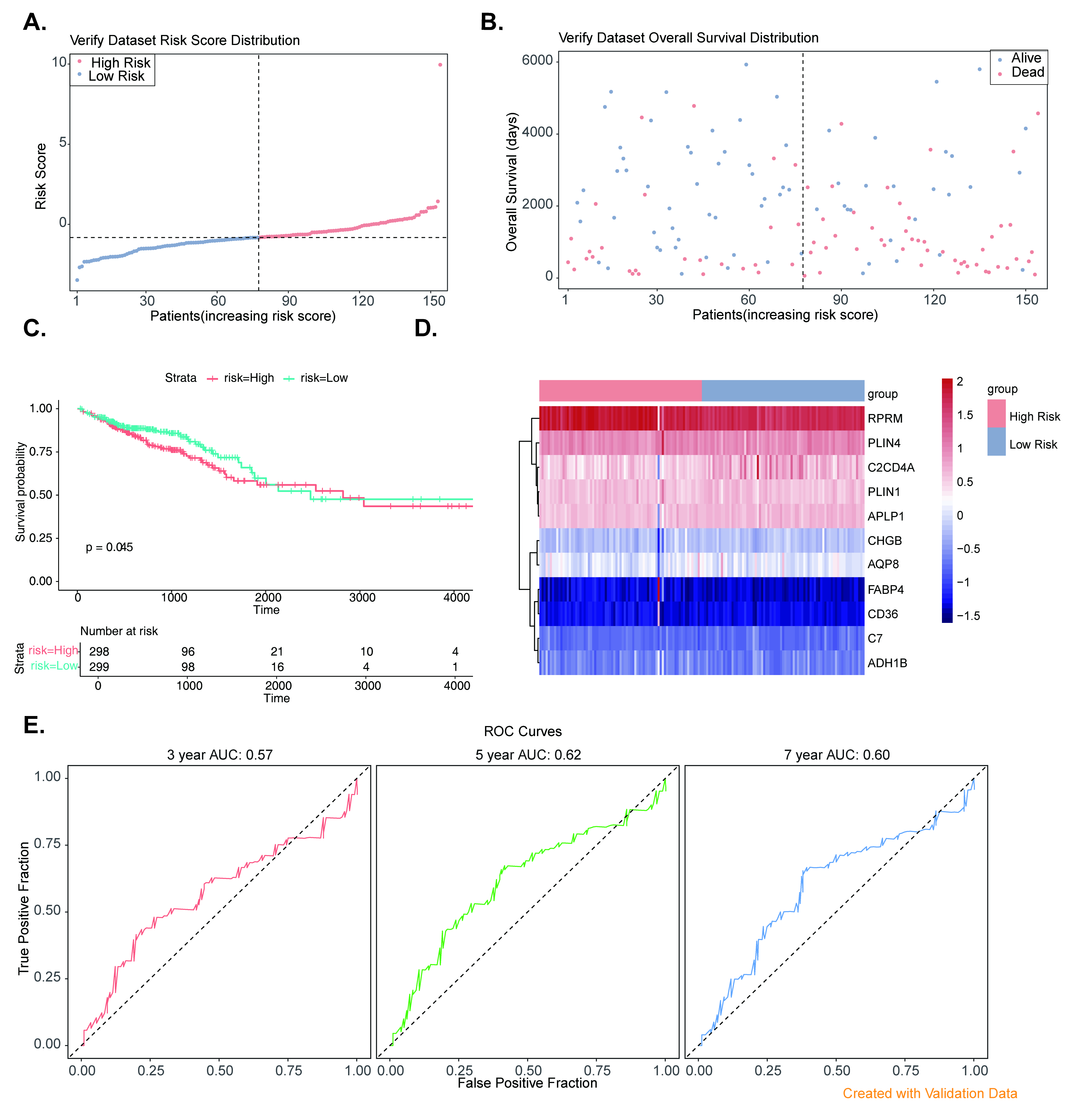

Supplement: Supplementary file 5 — Supplementary Material 5. Figure S5 Validation of the established prognostic risk model using GSE106584 dataset. (A) Risk score distribution of GSE106584 dataset. (B) Overall survival distribution of GSE106584 dataset. (C) Kaplan–Meier survival analyses of patients in high and low risk groups based on GSE106584. Upper: Survival curve plots probability of survival versus overall survival; Bottom: Risk list chart. (D) Heatmap of expression profiles of genes in the prognostic model. (E) ROC curve for the 3-, 5-, and 7-years [file 12885_2025_14918_MOESM5_ESM.tif]

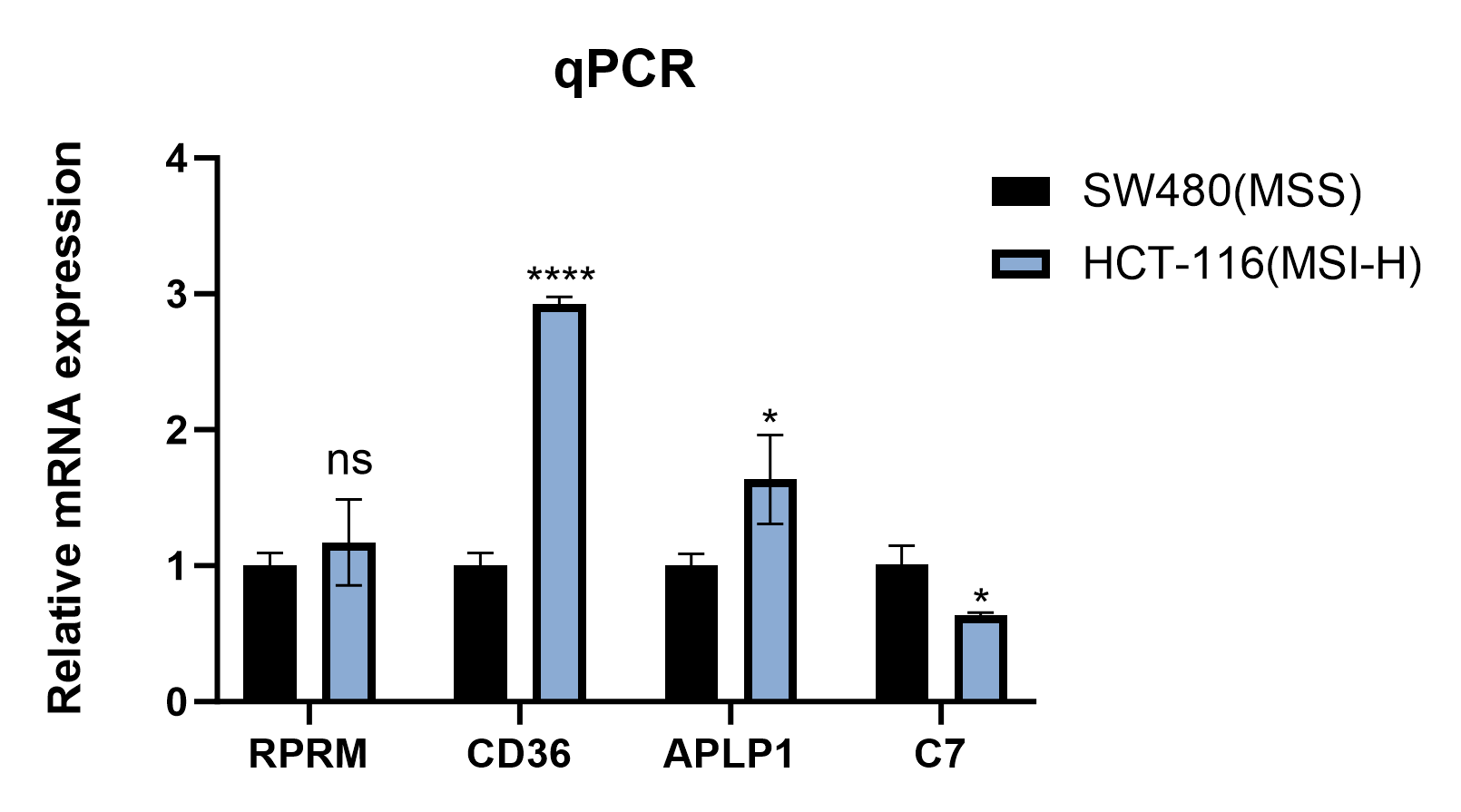

Supplement: Supplementary file 6 — Supplementary Material 6. Figure S6 The differential expression of multiple prognostic genes in MSI-H and MSS colorectal cancer cells was analyzed using qPCR. Statistical significance is indicated at *p < 0.05 vs. MSS, ****p < 0.0001 vs. MSS, respectively [file 12885_2025_14918_MOESM6_ESM.tif]

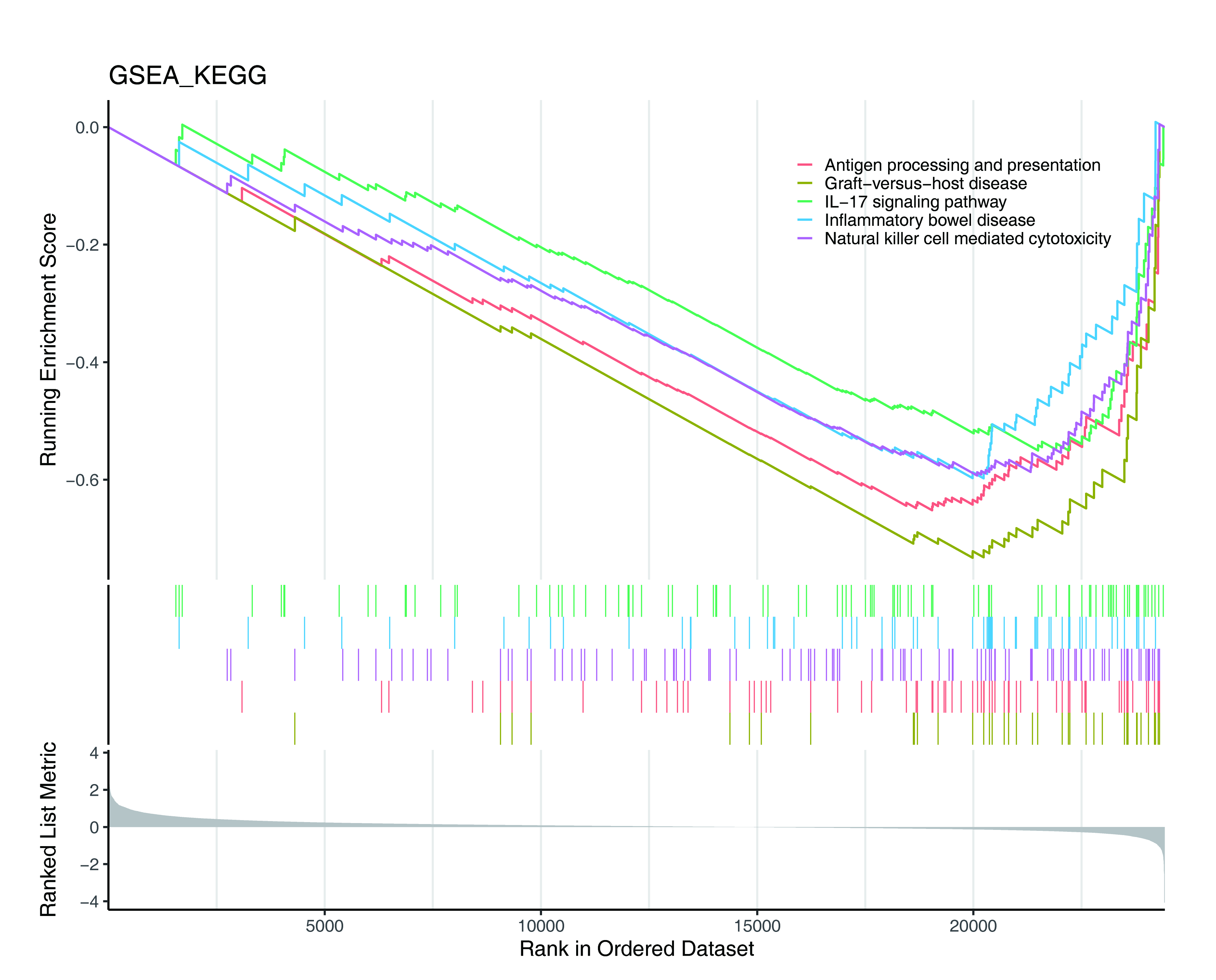

Supplement: Supplementary file 7 — Supplementary Material 7. Figure S7 Results of enrichment analysis of GSEA. The curves represented the connecting lines of the enrichment scores of each gene inside the pathway, different colours indicate different pathways. The figure showed the enriched pathways for the top 5 [file 12885_2025_14918_MOESM7_ESM.tif]

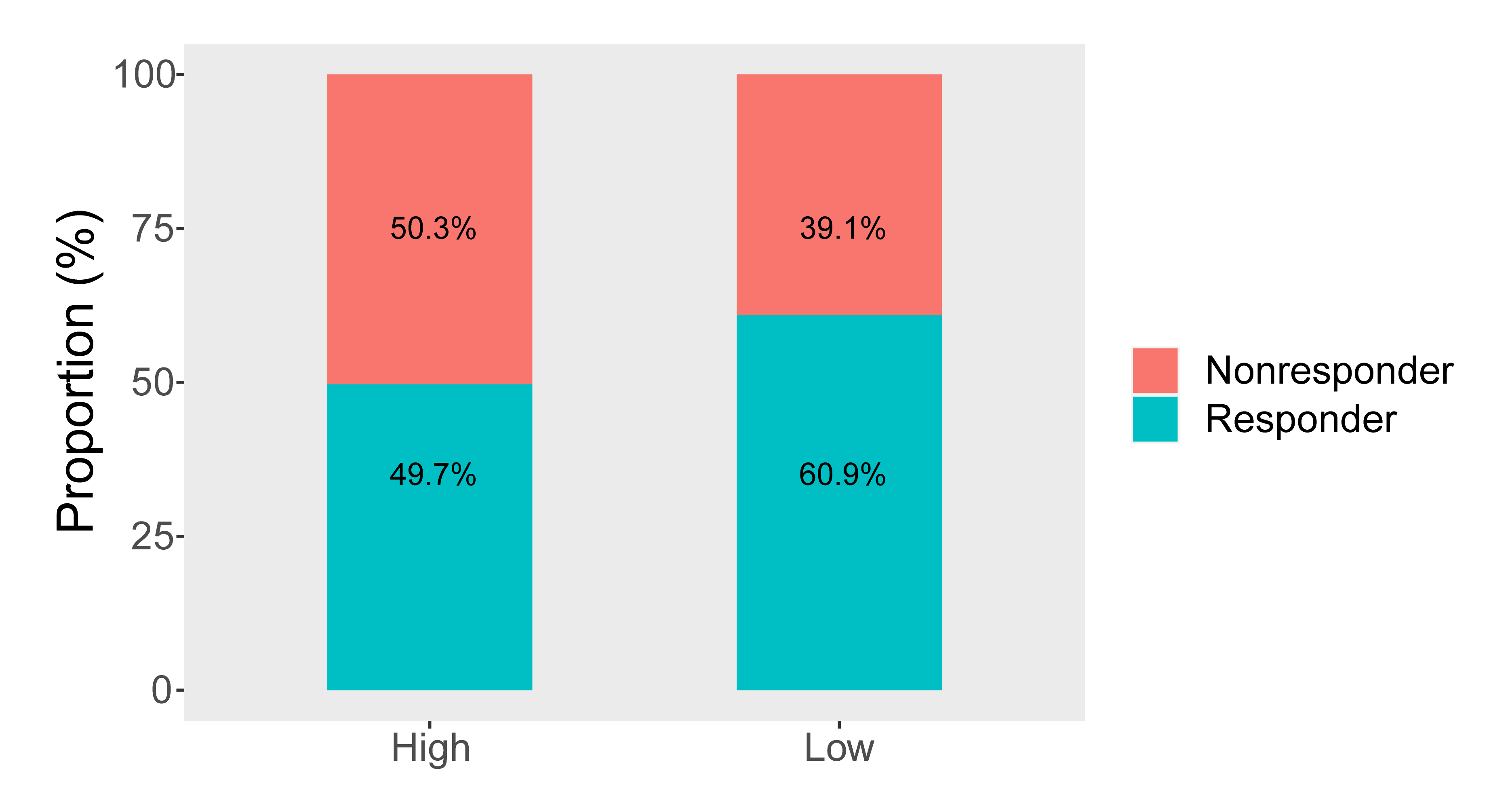

Supplement: Supplementary file 8 — Supplementary Material 8. Figure S8 Immune response in high and low risk groups [file 12885_2025_14918_MOESM8_ESM.tif]

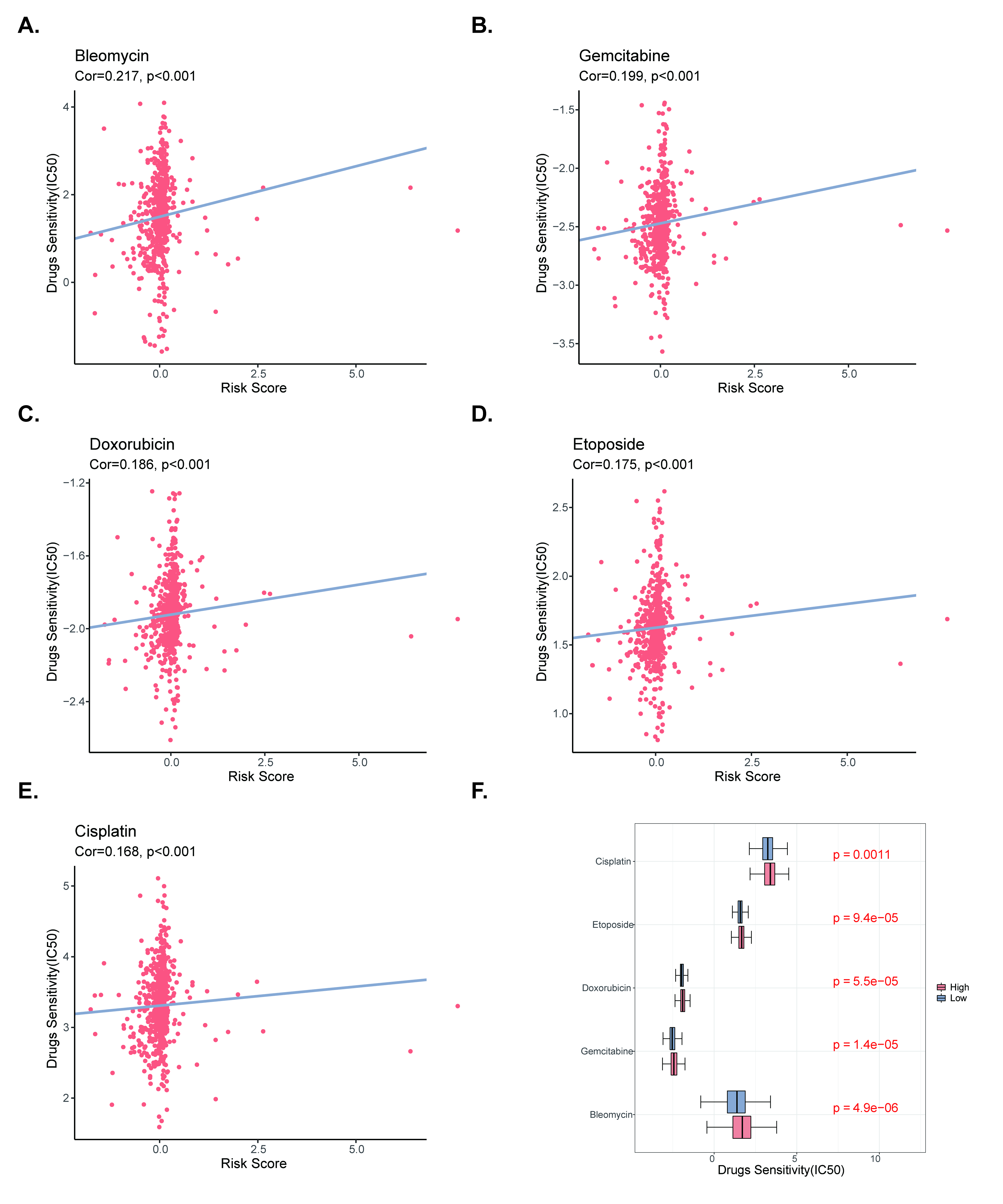

Supplement: Supplementary file 9 — Supplementary Material 9. Figure S9 Drug sensitivity analysis. (A-E) Correlation analysis between drug sensitivities and risk scores. A: Cisplatin; B: Etoposide; C: Doxorubicin; D: Gemcitabine; E: Bleomycin. (F) Variations in drug sensitivity between risk groups [file 12885_2025_14918_MOESM9_ESM.tif]

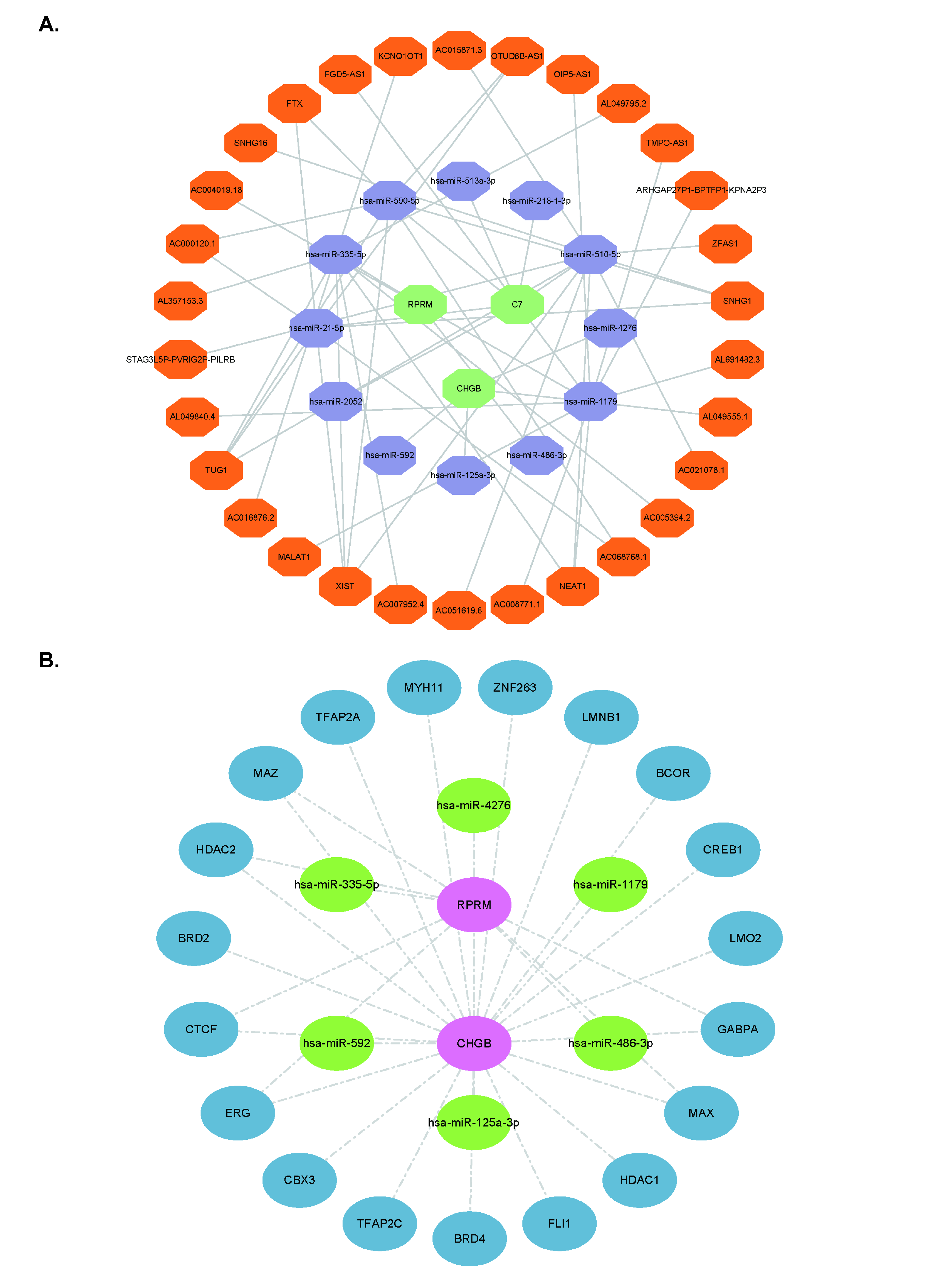

Supplement: Supplementary file 10 — Supplementary Material 10. Figure S10 Molecular network. (A) LncRNA-miRNA-mRNA network. Orange denotes lncRNAs, blue denotes miRNAs, and green denotes prognostic genes. (B) TF-mRNA-miRNA network. Blue represents transcription factors (TF), green represents miRNAs, and pink represents prognostic genes [file 12885_2025_14918_MOESM10_ESM.tif]
