# Supplementary material for: Development of a prognostic risk model for colorectal cancer based on microsatellite stability-associated genes
Source: BMC Cancer. 2025 Oct 1;25:1490. doi: 10.1186/s12885-025-14918-y (PMC12487216; doi:10.1186/s12885-025-14918-y)
Supplement: Supplementary file 11 — Supplementary Material 11. [file 12885_2025_14918_MOESM11_ESM.zip › Table S1.pdf]

GeneSymbol	baseMean	log2FoldChar	lfcSE	stat	pvalue	padj	change	Symbols
CA7	207.617063	2.93138169	0.23890364	12.2701423	1.31E-34	2.25E-30	Up	CA7
TRAPPC5	104.807281	1.66092929	0.15610788	10.6396247	1.95E-26	1.67E-22	Up	TRAPPC5
GUCA2B	218.09175	3.3326254	0.32215637	10.3447448	4.42E-25	2.53E-21	Up	GUCA2B
TRPV6	40.441406	2.71210999	0.26565973	10.2089616	1.81E-24	7.75E-21	Up	TRPV6
MUC6	220.445705	2.84323902	0.28898685	9.83864484	7.67E-23	2.63E-19	Up	MUC6
AHSG	31.1414377	3.09778016	0.31962644	9.69187692	3.26E-22	9.33E-19	Up	AHSG
ALB	45.4888128	3.69599255	0.38756088	9.536547	1.48E-21	3.62E-18	Up	ALB
BEST4	173.383812	2.13155975	0.22404544	9.5139617	1.84E-21	3.94E-18	Up	BEST4
CLDN18	259.468092	3.11711785	0.32811916	9.49995678	2.10E-21	4.00E-18	Up	CLDN18
KCNJ13	8.99731153	3.57965037	0.39159842	9.14112571	6.18E-20	1.06E-16	Up	KCNJ13
CD177	1561.8861	2.55728632	0.2805183	9.11629054	7.77E-20	1.21E-16	Up	CD177
CCL25	44.0650556	2.84497132	0.3133718	9.07858122	1.10E-19	1.57E-16	Up	CCL25
NACA2	33.9685912	1.60981782	0.1781778	9.03489537	1.64E-19	2.17E-16	Up	NACA2
MUC5AC	1366.5691	2.41898515	0.27109723	8.922943	4.54E-19	5.56E-16	Up	MUC5AC
CA1	1681.40748	2.92037025	0.33038462	8.83930445	9.63E-19	1.10E-15	Up	CA1
MTRNR2L3	11.372343	1.70431284	0.204553	8.33188855	7.96E-17	8.53E-14	Up	MTRNR2L3
TMEM160	565.646082	1.2697018	0.15388893	8.25076752	1.57E-16	1.35E-13	Up	TMEM160
ZNF460	73.9649584	1.25512309	0.15191128	8.26221123	1.43E-16	1.35E-13	Up	ZNF460
HBE1	15.0118107	4.08217242	0.49409504	8.26191737	1.43E-16	1.35E-13	Up	HBE1
C4orf48	661.958583	1.36140232	0.16497517	8.25216477	1.56E-16	1.35E-13	Up	C4orf48
LIN28A	3.55234959	3.3817089	0.41847889	8.08095447	6.43E-16	5.25E-13	Up	LIN28A
FOXI3	6.85379865	3.04859257	0.37813106	8.06226437	7.49E-16	5.84E-13	Up	FOXI3
TMIGD1	243.611027	2.79075618	0.34766208	8.02720895	9.97E-16	7.44E-13	Up	TMIGD1
NOXO1	2.47736266	2.48469667	0.30982656	8.01963744	1.06E-15	7.58E-13	Up	NOXO1
DRD5	7.98544069	2.94077338	0.36979467	7.95244933	1.83E-15	1.25E-12	Up	DRD5
NXPH4	210.262987	1.86525524	0.2358258	7.90946208	2.59E-15	1.71E-12	Up	NXPH4
GPR25	13.9760728	1.51966186	0.19289563	7.87815597	3.32E-15	2.11E-12	Up	GPR25
GPR18	34.4022476	1.35212728	0.17359832	7.78882681	6.76E-15	4.14E-12	Up	GPR18
SOCS1	278.227806	1.0117483	0.12999903	7.7827371	7.10E-15	4.20E-12	Up	SOCS1
FEZF2	2.43878773	3.17447761	0.41202931	7.70449462	1.31E-14	7.51E-12	Up	FEZF2
DLK1	5.55528642	3.6446585	0.47344865	7.69810724	1.38E-14	7.64E-12	Up	DLK1
KCNQ2	15.2211295	1.95076344	0.25581058	7.62581228	2.43E-14	1.30E-11	Up	KCNQ2
REG1B	1329.88262	2.46150111	0.33116715	7.43280583	1.06E-13	5.53E-11	Up	REG1B
RELL1	149.554414	0.78936684	0.10635881	7.42173454	1.16E-13	5.83E-11	Up	RELL1
SLC6A19	152.785878	2.24328515	0.3029833	7.40398936	1.32E-13	6.48E-11	Up	SLC6A19
POU5F2	4.68988539	2.62849986	0.35571649	7.38931123	1.48E-13	7.03E-11	Up	POU5F2
MTTP	225.394726	2.06408417	0.27974488	7.37845195	1.60E-13	7.42E-11	Up	MTTP
NMUR2	19.1360485	2.41465588	0.32746366	7.37381337	1.66E-13	7.49E-11	Up	NMUR2
APOA1	27.4677709	1.76461463	0.24080194	7.32807471	2.33E-13	1.03E-10	Up	APOA1
GPR22	5.27033845	2.07664857	0.28464696	7.29552349	2.98E-13	1.28E-10	Up	GPR22
PYY	228.681682	2.27981929	0.31541703	7.22795248	4.90E-13	2.05E-10	Up	PYY
ABCG2	223.803837	1.41147297	0.19626258	7.19175796	6.40E-13	2.61E-10	Up	ABCG2
ERVH48-1	7.19021897	1.45303179	0.20288211	7.16195146	7.95E-13	3.17E-10	Up	ERVH48-1
GTF2H4	55.9732062	-0.51573265	0.07205323	-7.15766189	8.21E-13	3.20E-10	Down	GTF2H4
SLC51A	242.782903	1.20926664	0.16914344	7.14935572	8.72E-13	3.32E-10	Up	SLC51A
HCN2	31.2203618	1.13276743	0.15921331	7.11477855	1.12E-12	4.01E-10	Up	HCN2
NHLH2	4.90238497	2.70811176	0.38053601	7.11657157	1.11E-12	4.01E-10	Up	NHLH2
SMIM18	3.50141175	2.24929349	0.315988	7.11828771	1.09E-12	4.01E-10	Up	SMIM18
DPPA4	3.69953895	1.9592018	0.27587396	7.10180051	1.23E-12	4.22E-10	Up	DPPA4
GLTPD2	38.7582692	0.98433643	0.13855304	7.10440173	1.21E-12	4.22E-10	Up	GLTPD2
OTOP2	97.0595205	3.06954416	0.43339028	7.08263259	1.41E-12	4.76E-10	Up	OTOP2
VNN1	383.28092	1.41209002	0.19971327	7.07058681	1.54E-12	4.92E-10	Up	VNN1
KLHL11	77.4343734	0.6989033	0.10000823	6.98845772	2.78E-12	8.36E-10	Up	KLHL11
APOB	56.3399888	2.16783121	0.31112139	6.96779874	3.22E-12	9.52E-10	Up	APOB
PLA2G2F	41.2462328	1.84502194	0.26530506	6.95434131	3.54E-12	1.02E-09	Up	PLA2G2F
GPR82	29.170591	1.08725293	0.15638537	6.95239539	3.59E-12	1.02E-09	Up	GPR82
MUC16	41.300325	1.86170835	0.26783898	6.95084908	3.63E-12	1.02E-09	Up	MUC16

ZNF771	214.834028	0.69701403	0.1006387	6.92590491	4.33E-12	1.20E-09	Up	ZNF771
CASKIN1	11.8453822	1.21819465	0.17647366	6.90298272	5.09E-12	1.36E-09	Up	CASKIN1
MS4A10	5.51510387	2.47770451	0.35890604	6.90349075	5.07E-12	1.36E-09	Up	MS4A10
IDO1	543.883156	1.2557787	0.18260494	6.87702472	6.11E-12	1.61E-09	Up	IDO1
ANXA10	26.2133522	2.20099283	0.32219707	6.83120065	8.42E-12	2.19E-09	Up	ANXA10
CLSTN2	157.124168	-1.22287091	0.17909876	-6.82791395	8.62E-12	2.21E-09	Down	CLSTN2
RAB19	79.9251424	0.60834584	0.0897488	6.77831694	1.22E-11	3.07E-09	Up	RAB19
GJD3	4.59689732	1.65225929	0.24421553	6.76557833	1.33E-11	3.30E-09	Up	GJD3
C9orf131	3.35827873	1.96327498	0.29079933	6.75130491	1.47E-11	3.59E-09	Up	C9orf131
CDH16	57.0050515	1.68185464	0.24992038	6.72956167	1.70E-11	4.11E-09	Up	CDH16
TF	43.4324004	1.47915141	0.22066657	6.70310587	2.04E-11	4.81E-09	Up	TF
CSNK1A1L	1.58767064	1.89198976	0.28227622	6.70261831	2.05E-11	4.81E-09	Up	CSNK1A1L
SLC30A10	44.080179	1.98259243	0.29754144	6.66324804	2.68E-11	6.21E-09	Up	SLC30A10
CAMK2N2	15.745656	1.16557556	0.17526369	6.65041092	2.92E-11	6.68E-09	Up	CAMK2N2
GNLY	229.761989	1.02051831	0.15361526	6.64333959	3.07E-11	6.92E-09	Up	GNLY
BTBD18	4.37850694	1.66280774	0.25057417	6.63599017	3.22E-11	7.18E-09	Up	BTBD18
PTGER1	27.1036039	1.14677143	0.17328899	6.61768207	3.65E-11	8.02E-09	Up	PTGER1
MS4A12	1083.0777	2.04815131	0.31006108	6.60563825	3.96E-11	8.49E-09	Up	MS4A12
C2CD4C	33.7743159	1.03820448	0.15715994	6.60603764	3.95E-11	8.49E-09	Up	C2CD4C
TPGS1	249.194222	0.88385409	0.13422088	6.58507128	4.55E-11	9.63E-09	Up	TPGS1
LY6D	29.63543	1.89498794	0.28796849	6.58053914	4.69E-11	9.81E-09	Up	LY6D
PLLP	572.388115	0.75975348	0.11558738	6.57297937	4.93E-11	1.02E-08	Up	PLLP
CYBA	8890.95351	0.67512039	0.10325714	6.53824393	6.22E-11	1.24E-08	Up	CYBA
ANGPTL3	7.96904915	1.80619606	0.27626425	6.53792905	6.24E-11	1.24E-08	Up	ANGPTL3
S100A9	1256.71119	0.97575486	0.14973084	6.51672596	7.19E-11	1.42E-08	Up	S100A9
CEBPE	10.2510111	1.37501945	0.21120714	6.5102886	7.50E-11	1.46E-08	Up	CEBPE
NAT8L	38.6394995	1.20251035	0.18477951	6.50781218	7.63E-11	1.47E-08	Up	NAT8L
PHGR1	8742.00505	1.04633112	0.16152054	6.47800665	9.29E-11	1.77E-08	Up	PHGR1
TNNT1	129.164053	1.54664886	0.2394037	6.46042168	1.04E-10	1.97E-08	Up	TNNT1
CCDC85B	1376.99803	0.84023759	0.13024641	6.45113814	1.11E-10	2.07E-08	Up	CCDC85B
PABPC3	86.4771298	0.89328408	0.1389862	6.42714213	1.30E-10	2.40E-08	Up	PABPC3
C15orf61	330.146678	0.52363053	0.08155944	6.4202319	1.36E-10	2.48E-08	Up	C15orf61
EPHA8	8.45855229	2.20924022	0.34493227	6.40485216	1.51E-10	2.69E-08	Up	EPHA8
ACSM6	2.50969179	2.46997276	0.38649874	6.39063594	1.65E-10	2.92E-08	Up	ACSM6
NDUFA4L2	698.552433	1.00033565	0.15657249	6.3889618	1.67E-10	2.92E-08	Up	NDUFA4L2
CSTL1	6.06533123	-1.22161009	0.19149821	-6.37922465	1.78E-10	3.08E-08	Down	CSTL1
POMK	84.0205391	0.73808043	0.11589721	6.36840543	1.91E-10	3.24E-08	Up	POMK
BAAT	21.9436841	1.50072876	0.23571562	6.36669208	1.93E-10	3.25E-08	Up	BAAT
ORM1	66.1768787	2.28831895	0.36061334	6.34563026	2.22E-10	3.69E-08	Up	ORM1
AQP5	71.4760619	1.7526416	0.27683269	6.33105002	2.43E-10	4.02E-08	Up	AQP5
IGLL1	6.39655328	1.53281027	0.24247022	6.32164328	2.59E-10	4.23E-08	Up	IGLL1
ANKUB1	3.00921801	1.9216859	0.30513758	6.29776876	3.02E-10	4.84E-08	Up	ANKUB1
OTOP3	5.548014	3.34328593	0.53145704	6.29079243	3.16E-10	4.94E-08	Up	OTOP3
GPR52	2.97051244	2.8280741	0.44951259	6.29142354	3.15E-10	4.94E-08	Up	GPR52
HBA1	24.0853007	1.58843184	0.25251817	6.29036649	3.17E-10	4.94E-08	Up	HBA1
ANGPTL7	17.8407282	1.97512737	0.31411674	6.28787683	3.22E-10	4.97E-08	Up	ANGPTL7
PDXP	75.8973578	0.70480358	0.11214031	6.28501548	3.28E-10	5.02E-08	Up	PDXP
HOXC10	10.5806061	1.69897321	0.27040317	6.2831113	3.32E-10	5.04E-08	Up	HOXC10
TAS2R30	2.60408749	2.65117523	0.42252612	6.2745831	3.51E-10	5.28E-08	Up	TAS2R30
XKR7	7.9498313	1.79054659	0.28593387	6.26210039	3.80E-10	5.67E-08	Up	XKR7
HBQ1	5.80666709	1.77630911	0.2837659	6.25976939	3.86E-10	5.70E-08	Up	HBQ1
AQP8	1123.48107	1.95612882	0.3128109	6.25339091	4.02E-10	5.89E-08	Up	AQP8
TYMP	2077.82697	0.76634382	0.12262757	6.24936014	4.12E-10	5.99E-08	Up	TYMP
TDRD1	11.4774329	1.4344544	0.23039895	6.22595893	4.79E-10	6.90E-08	Up	TDRD1
CLDN6	7.10530129	1.44136129	0.23173542	6.2198574	4.98E-10	7.11E-08	Up	CLDN6
ERVFRD-1	6.24543753	1.65424731	0.26631646	6.21158503	5.25E-10	7.44E-08	Up	ERVFRD-1
GBP4	1378.0875	0.91782667	0.14784628	6.20797926	5.37E-10	7.55E-08	Up	GBP4
NPIPA1	87.0153592	-0.55021268	0.08879747	-6.19626499	5.78E-10	7.93E-08	Down	NPIPA1

GZMA	220.92956	0.89767608	0.14536785	6.17520353	6.61E-10	9.00E-08	Up	GZMA
BATF3	64.3645049	0.6171702	0.09997586	6.17319195	6.69E-10	9.04E-08	Up	BATF3
PDF	185.077026	0.73032635	0.11843885	6.16627357	6.99E-10	9.37E-08	Up	PDF
CARD9	147.899175	0.83136729	0.13504464	6.15624052	7.45E-10	9.91E-08	Up	CARD9
MLN	2.01237187	2.75896091	0.44919651	6.14199099	8.15E-10	1.08E-07	Up	MLN
PGLYRP2	2.90621127	1.4538817	0.23754761	6.12038025	9.34E-10	1.20E-07	Up	PGLYRP2
CCR10	26.4702277	0.85476467	0.13964546	6.12096272	9.30E-10	1.20E-07	Up	CCR10
UNC13A	45.2127165	0.91748634	0.15024898	6.10643956	1.02E-09	1.28E-07	Up	UNC13A
TRIM40	98.8760884	1.0504111	0.17204153	6.10556691	1.02E-09	1.28E-07	Up	TRIM40
MT1M	266.179251	1.4267584	0.23369372	6.1052493	1.03E-09	1.28E-07	Up	MT1M
CEBPD	1551.57178	0.56717483	0.09289028	6.10585754	1.02E-09	1.28E-07	Up	CEBPD
HLA-DMB	989.543354	0.75101918	0.12296002	6.10783251	1.01E-09	1.28E-07	Up	HLA-DMB
PYGM	61.0245135	1.09878175	0.18007782	6.10170521	1.05E-09	1.30E-07	Up	PYGM
FEM1A	117.169103	0.57782714	0.09486192	6.09124449	1.12E-09	1.36E-07	Up	FEM1A
RHOF	218.112475	0.76059455	0.12529267	6.07054318	1.27E-09	1.53E-07	Up	RHOF
TCP11L2	179.838661	0.58126505	0.09641482	6.0287937	1.65E-09	1.95E-07	Up	TCP11L2
CALB1	103.761459	1.71938628	0.2854012	6.02445348	1.70E-09	1.99E-07	Up	CALB1
HBZ	2.83093848	4.33566615	0.72003913	6.02143131	1.73E-09	2.02E-07	Up	HBZ
ZNF683	41.0067485	1.14817737	0.19148617	5.99613737	2.02E-09	2.34E-07	Up	ZNF683
OLIG1	3.05067655	1.3147745	0.21945852	5.99099312	2.09E-09	2.39E-07	Up	OLIG1
LGALS12	9.65983928	1.25563376	0.20962583	5.98988104	2.10E-09	2.39E-07	Up	LGALS12
CFAP46	12.1551938	1.27853368	0.21442119	5.96272087	2.48E-09	2.80E-07	Up	CFAP46
SST	33.368897	1.754052	0.29432077	5.95966094	2.53E-09	2.82E-07	Up	SST
CTXN1	151.025326	0.98489929	0.16524072	5.96039087	2.52E-09	2.82E-07	Up	CTXN1
SAA1	386.91518	1.25496429	0.21063479	5.9580104	2.55E-09	2.83E-07	Up	SAA1
ERVW-1	9.9128298	1.35263042	0.22743416	5.94734934	2.73E-09	3.00E-07	Up	ERVW-1
BARX1	15.7362335	1.42417522	0.23994028	5.93554028	2.93E-09	3.20E-07	Up	BARX1
PRSS1	32.6787765	1.53139189	0.25851796	5.92373509	3.15E-09	3.42E-07	Up	PRSS1
APOA4	10.3279339	3.65602343	0.61901688	5.90617731	3.50E-09	3.75E-07	Up	APOA4
STAR	4.144712	0.94750203	0.16082991	5.89132972	3.83E-09	4.08E-07	Up	STAR
OMG	11.4948271	0.99590283	0.16946955	5.87658858	4.19E-09	4.35E-07	Up	OMG
CTU1	306.655155	0.74628307	0.1269909	5.8766657	4.19E-09	4.35E-07	Up	CTU1
ACKR4	28.1477206	1.01680786	0.1731597	5.8720816	4.30E-09	4.45E-07	Up	ACKR4
TMEM238	874.230388	0.87725665	0.1502061	5.8403529	5.21E-09	5.32E-07	Up	TMEM238
ADCY8	1.84747398	2.38868747	0.40970558	5.83025363	5.53E-09	5.62E-07	Up	ADCY8
CLCA4	2475.92204	1.8076699	0.31022607	5.82694388	5.65E-09	5.64E-07	Up	CLCA4
IL17C	13.7938271	1.27113657	0.21815911	5.82664894	5.66E-09	5.64E-07	Up	IL17C
FSD2	3.08250601	1.40300732	0.24077957	5.82693683	5.65E-09	5.64E-07	Up	FSD2
SCRG1	24.3193385	1.48822498	0.25566064	5.82109551	5.85E-09	5.80E-07	Up	SCRG1
TAS2R43	2.0025358	2.22007752	0.38164806	5.81708057	5.99E-09	5.90E-07	Up	TAS2R43
GBA3	77.5191771	1.47881323	0.2545668	5.80913615	6.28E-09	6.16E-07	Up	GBA3
HLA-DRA	14810.622	0.75182544	0.12961091	5.80063382	6.61E-09	6.44E-07	Up	HLA-DRA
CST6	24.7534208	1.25212622	0.21602263	5.79627348	6.78E-09	6.57E-07	Up	CST6
TMEM121	40.5631966	0.81528141	0.14098012	5.78295303	7.34E-09	6.96E-07	Up	TMEM121
HES4	321.758441	0.85591421	0.14836896	5.76882268	7.98E-09	7.52E-07	Up	HES4
TMEM151A	30.2738607	1.14161552	0.19821957	5.75934815	8.44E-09	7.87E-07	Up	TMEM151A
PTGES3L-AA	9.7275259	-0.74814294	0.13004115	-5.75312467	8.76E-09	8.12E-07	Down	PTGES3L-AARSD1
ISG20	783.750013	0.57673087	0.10032631	5.74855055	9.00E-09	8.30E-07	Up	ISG20
PZP	7.0361764	1.30622952	0.22727007	5.74747713	9.06E-09	8.31E-07	Up	PZP
PILRB	247.454899	-0.71262326	0.1241852	-5.73839133	9.56E-09	8.68E-07	Down	PILRB
ABI3	319.686912	0.53122988	0.09264406	5.73409515	9.80E-09	8.85E-07	Up	ABI3
TFF2	201.242543	1.32155848	0.23054153	5.73240972	9.90E-09	8.89E-07	Up	TFF2
HCAR2	73.6446839	1.21501809	0.21207998	5.72905615	1.01E-08	9.02E-07	Up	HCAR2
SPIN2A	7.08377433	1.08755565	0.1899502	5.72547769	1.03E-08	9.17E-07	Up	SPIN2A
RARRES1	983.743133	0.87570714	0.15311829	5.71915451	1.07E-08	9.42E-07	Up	RARRES1
RAB33A	23.3788886	0.58618827	0.10273072	5.70606576	1.16E-08	1.01E-06	Up	RAB33A
HOXC4	29.6492375	1.10586884	0.1939755	5.70107486	1.19E-08	1.03E-06	Up	HOXC4
ZNF467	101.096093	0.59643893	0.10464828	5.69946247	1.20E-08	1.04E-06	Up	ZNF467

VPREB1	1.16712776	2.51871809	0.44209401	5.69724539	1.22E-08	1.04E-06	Up	VPREB1
IL12RB1	163.914317	0.66230258	0.11637811	5.69095508	1.26E-08	1.08E-06	Up	IL12RB1
SLC14A1	223.136941	1.35014971	0.23741265	5.68693246	1.29E-08	1.10E-06	Up	SLC14A1
MEP1B	57.699152	1.33407444	0.23515291	5.67322096	1.40E-08	1.18E-06	Up	MEP1B
HLA-G	309.456697	0.74640697	0.13170308	5.6673462	1.45E-08	1.22E-06	Up	HLA-G
ITGB7	191.623711	0.71866771	0.12695349	5.66087379	1.51E-08	1.26E-06	Up	ITGB7
KRT2	3.33321606	1.38567695	0.24524791	5.65010697	1.60E-08	1.33E-06	Up	KRT2
UPK1B	3.47972126	1.88667313	0.33429696	5.64370406	1.66E-08	1.37E-06	Up	UPK1B
RBFOX3	41.9481108	1.22688903	0.21751566	5.64046308	1.70E-08	1.39E-06	Up	RBFOX3
UNC5A	31.0874492	1.13621048	0.20151714	5.63828222	1.72E-08	1.40E-06	Up	UNC5A
ODF3B	317.97631	0.63590945	0.11281242	5.63687438	1.73E-08	1.40E-06	Up	ODF3B
SERPINB2	25.2890661	1.53629533	0.27253553	5.63704598	1.73E-08	1.40E-06	Up	SERPINB2
SLC9A3	1658.4601	1.20379889	0.21397554	5.62587139	1.85E-08	1.47E-06	Up	SLC9A3
POU3F2	4.43123449	1.0838172	0.1927338	5.62338947	1.87E-08	1.48E-06	Up	POU3F2
TAS2R13	2.08535318	2.35607841	0.4193001	5.61907425	1.92E-08	1.51E-06	Up	TAS2R13
SKOR2	0.94533908	2.46096781	0.43804532	5.61806664	1.93E-08	1.51E-06	Up	SKOR2
C1QL1	33.2484842	0.85196406	0.15170941	5.61576292	1.96E-08	1.53E-06	Up	C1QL1
PDC	2.98396966	1.75695702	0.31344355	5.60533788	2.08E-08	1.61E-06	Up	PDC
NME2	32.9881596	-0.61514605	0.10976742	-5.60408577	2.09E-08	1.62E-06	Down	NME2
SCARA5	336.746736	1.2865712	0.22975802	5.59967921	2.15E-08	1.64E-06	Up	SCARA5
S100B	115.80316	0.80172711	0.14368928	5.57958902	2.41E-08	1.81E-06	Up	S100B
CIITA	738.413818	0.77349621	0.13861709	5.58009281	2.40E-08	1.81E-06	Up	CIITA
ADAT3	282.249072	0.67713266	0.121413	5.57710191	2.45E-08	1.82E-06	Up	ADAT3
C8G	77.8144751	0.90546404	0.16252548	5.57121299	2.53E-08	1.86E-06	Up	C8G
SPTA1	2.92616046	1.38807965	0.24946555	5.56421383	2.63E-08	1.93E-06	Up	SPTA1
CD160	22.1733935	0.84465511	0.15226587	5.54723877	2.90E-08	2.12E-06	Up	CD160
TSPYL6	2.25962567	2.64577838	0.47717815	5.54463437	2.95E-08	2.14E-06	Up	TSPYL6
IFNK	2.36650796	3.90080125	0.703809	5.54241457	2.98E-08	2.15E-06	Up	IFNK
PLP1	69.9772197	1.50056016	0.27081178	5.54097067	3.01E-08	2.16E-06	Up	PLP1
ATP1A3	151.161096	0.93816271	0.16936982	5.53913729	3.04E-08	2.17E-06	Up	ATP1A3
LRRC36	127.008108	-0.84416037	0.15251615	-5.53489188	3.11E-08	2.22E-06	Down	LRRC36
EGFL8	46.2683475	-0.51055638	0.09234037	-5.52907034	3.22E-08	2.28E-06	Down	EGFL8
IL1RL1	41.8208104	0.82434487	0.14920641	5.52486241	3.30E-08	2.31E-06	Up	IL1RL1
GPR34	154.290871	0.75253441	0.13617452	5.5262496	3.27E-08	2.31E-06	Up	GPR34
SLC7A9	32.6989302	1.28238139	0.23217341	5.52337751	3.33E-08	2.32E-06	Up	SLC7A9
UBE2L6	2766.92013	0.54421299	0.0985466	5.52239222	3.34E-08	2.32E-06	Up	UBE2L6
NUP210L	5.01389184	1.16869736	0.21180785	5.51772451	3.43E-08	2.38E-06	Up	NUP210L
MESP1	122.118883	0.9093997	0.16499121	5.51180693	3.55E-08	2.43E-06	Up	MESP1
TEX101	8.05295513	1.29624396	0.23556749	5.50264377	3.74E-08	2.53E-06	Up	TEX101
SERPINB7	22.9350029	1.48924436	0.27065281	5.50241598	3.75E-08	2.53E-06	Up	SERPINB7
P2RY6	148.997388	0.6249952	0.11357748	5.50280904	3.74E-08	2.53E-06	Up	P2RY6
HLA-DMA	1693.92707	0.63504353	0.11564317	5.49140537	3.99E-08	2.68E-06	Up	HLA-DMA
SEZ6	9.6098827	1.29640827	0.23651436	5.4813089	4.22E-08	2.83E-06	Up	SEZ6
UBASH3B	236.815884	0.64651502	0.11804583	5.47681377	4.33E-08	2.89E-06	Up	UBASH3B
CD3D	184.761189	0.64654416	0.11815749	5.47188464	4.45E-08	2.96E-06	Up	CD3D
ENPP7	3.34637674	1.34760867	0.24635168	5.47026375	4.49E-08	2.98E-06	Up	ENPP7
SNPH	179.327235	0.70674421	0.12929391	5.46618339	4.60E-08	3.03E-06	Up	SNPH
ALX4	2.8148975	1.30323515	0.23854373	5.46329665	4.67E-08	3.04E-06	Up	ALX4
SYT16	2.89370243	1.46991689	0.26899078	5.46456231	4.64E-08	3.04E-06	Up	SYT16
FAM83A	68.8851146	1.08708525	0.19898195	5.46323535	4.68E-08	3.04E-06	Up	FAM83A
S100A12	12.9677168	1.13780433	0.20826104	5.46335659	4.67E-08	3.04E-06	Up	S100A12
HOMER2	74.3368937	0.96671324	0.17697989	5.46227741	4.70E-08	3.04E-06	Up	HOMER2
OASL	555.04638	0.68502209	0.12577103	5.44658067	5.13E-08	3.30E-06	Up	OASL
PSMB9	3036.25175	0.57439818	0.10546983	5.44608999	5.15E-08	3.30E-06	Up	PSMB9
SLC15A3	792.374181	0.54811036	0.1006719	5.44452195	5.19E-08	3.31E-06	Up	SLC15A3
VAMP5	506.13265	0.58410545	0.10731416	5.44294881	5.24E-08	3.33E-06	Up	VAMP5
SLC14A2	15.4977725	1.39720519	0.25676163	5.44164332	5.28E-08	3.34E-06	Up	SLC14A2
C22orf15	4.94781164	0.81187347	0.14932503	5.43695518	5.42E-08	3.42E-06	Up	C22orf15

GBP5	425.095086	0.83480606	0.15357027	5.43598754	5.45E-08	3.42E-06	Up	GBP5
DCT	11.7658387	1.46939141	0.27074005	5.42731459	5.72E-08	3.57E-06	Up	DCT
SLC37A2	386.584299	0.81802205	0.15073642	5.4268374	5.74E-08	3.57E-06	Up	SLC37A2
HSF4	373.164761	-0.62934148	0.11602116	-5.42436787	5.82E-08	3.60E-06	Down	HSF4
RPE65	4.48544178	1.46138598	0.26948378	5.42290877	5.86E-08	3.62E-06	Up	RPE65
NKAIN1	9.93965472	0.95220232	0.17610721	5.40694698	6.41E-08	3.89E-06	Up	NKAIN1
CD244	41.9461781	0.74084526	0.13717702	5.4006512	6.64E-08	4.00E-06	Up	CD244
FAM171A2	37.3250101	0.64197087	0.11888916	5.39974253	6.67E-08	4.00E-06	Up	FAM171A2
TAS2R3	3.01202965	1.98091633	0.36738687	5.39190839	6.97E-08	4.17E-06	Up	TAS2R3
RILP	438.122261	0.51128736	0.09488068	5.38874069	7.10E-08	4.21E-06	Up	RILP
MAPK12	197.456892	0.67512397	0.12528362	5.38876473	7.09E-08	4.21E-06	Up	MAPK12
CD52	388.774629	0.6461789	0.12009333	5.38063917	7.42E-08	4.38E-06	Up	CD52
ANK1	57.9534631	0.7067004	0.1314154	5.37760701	7.55E-08	4.39E-06	Up	ANK1
CLEC4A	85.2903545	0.57677069	0.10724423	5.37810479	7.53E-08	4.39E-06	Up	CLEC4A
INSL6	1.65850108	2.52392474	0.46921284	5.37906155	7.49E-08	4.39E-06	Up	INSL6
CELF3	75.3419388	1.06116121	0.19729552	5.37853675	7.51E-08	4.39E-06	Up	CELF3
GRIN3A	39.5371476	0.66101923	0.12303818	5.37247222	7.77E-08	4.50E-06	Up	GRIN3A
SECTM1	1236.5937	0.86875139	0.16183686	5.36806889	7.96E-08	4.58E-06	Up	SECTM1
KCNK12	12.3205354	0.83581029	0.15574882	5.36639872	8.03E-08	4.59E-06	Up	KCNK12
KCNG2	7.10352324	0.83762449	0.15614175	5.3645132	8.12E-08	4.63E-06	Up	KCNG2
AMN	2155.73318	0.73210712	0.13653542	5.36203069	8.23E-08	4.67E-06	Up	AMN
VAX1	6.208064	2.35114622	0.43974789	5.34657755	8.96E-08	5.07E-06	Up	VAX1
CD7	271.238859	0.75622748	0.14145379	5.34610982	8.99E-08	5.07E-06	Up	CD7
HBG2	3.37335598	1.58082257	0.29607595	5.33924686	9.33E-08	5.25E-06	Up	HBG2
ASPG	88.8384187	1.04273074	0.19541892	5.33587398	9.51E-08	5.31E-06	Up	ASPG
ENDOG	377.028437	0.60336407	0.11307068	5.33616724	9.49E-08	5.31E-06	Up	ENDOG
INSL4	2.53936094	2.52310629	0.47324274	5.33152662	9.74E-08	5.42E-06	Up	INSL4
TTC36	5.22527668	0.93784823	0.17600536	5.32852088	9.90E-08	5.50E-06	Up	TTC36
PCP2	11.8873222	-0.74186336	0.13925629	-5.32732395	9.97E-08	5.52E-06	Down	PCP2
DAO	8.60464639	1.58528588	0.2976487	5.32602996	1.00E-07	5.54E-06	Up	DAO
PSAPL1	4.24166612	1.93808694	0.36491699	5.3110351	1.09E-07	5.97E-06	Up	PSAPL1
PRR7	424.917099	0.75377101	0.14237706	5.2941885	1.20E-07	6.49E-06	Up	PRR7
VNN2	101.867694	0.91409625	0.17285995	5.28807434	1.24E-07	6.69E-06	Up	VNN2
ITIH2	12.3959014	1.09826448	0.20790824	5.28244807	1.27E-07	6.88E-06	Up	ITIH2
TRPM1	1.90196012	1.95862451	0.37082487	5.28180463	1.28E-07	6.88E-06	Up	TRPM1
LAG3	148.588659	0.6933657	0.13128945	5.28119896	1.28E-07	6.88E-06	Up	LAG3
ULBP2	65.6807663	0.89983628	0.1706816	5.272017	1.35E-07	7.21E-06	Up	ULBP2
ATP6V1B1	19.1961833	0.92834459	0.17621846	5.26814623	1.38E-07	7.32E-06	Up	ATP6V1B1
ALDOB	1270.64975	1.00851373	0.1916628	5.26191707	1.43E-07	7.48E-06	Up	ALDOB
NPTX1	56.986314	1.22273311	0.23240467	5.26122428	1.43E-07	7.48E-06	Up	NPTX1
VSTM1	5.45681018	1.1429422	0.21753002	5.2541814	1.49E-07	7.68E-06	Up	VSTM1
UBE2NL	2.27045514	1.16592819	0.22188873	5.2545625	1.48E-07	7.68E-06	Up	UBE2NL
RPL39	4500.40772	-0.59819889	0.11390183	-5.2518813	1.51E-07	7.76E-06	Down	RPL39
EMX1	38.5961297	1.32774599	0.25285655	5.25098506	1.51E-07	7.77E-06	Up	EMX1
SOX18	253.347861	0.57580294	0.10977139	5.24547356	1.56E-07	7.98E-06	Up	SOX18
PPBP	190.594274	1.47913268	0.28219582	5.24151166	1.59E-07	8.11E-06	Up	PPBP
TRIM72	153.4558	1.46409764	0.27940321	5.24008886	1.60E-07	8.12E-06	Up	TRIM72
GABRP	231.748061	1.18955736	0.22703566	5.23951764	1.61E-07	8.12E-06	Up	GABRP
CNTN2	18.1886142	1.31159438	0.25051769	5.23553601	1.65E-07	8.28E-06	Up	CNTN2
B3GAT1	19.8168794	0.74310347	0.14210238	5.22935277	1.70E-07	8.53E-06	Up	B3GAT1
PBOV1	2.66167209	2.1169758	0.40488156	5.22862988	1.71E-07	8.54E-06	Up	PBOV1
KIR3DL1	1.98862068	1.63802888	0.31333898	5.22765752	1.72E-07	8.56E-06	Up	KIR3DL1
EIF3CL	9.19543477	-0.64231959	0.12291107	-5.225889	1.73E-07	8.62E-06	Down	EIF3CL
FBXL15	742.099303	0.51198818	0.09815927	5.21589205	1.83E-07	8.90E-06	Up	FBXL15
GJA3	20.6145073	1.052432	0.20174653	5.21660512	1.82E-07	8.90E-06	Up	GJA3
AIF1L	256.117938	0.67654501	0.12968254	5.21693214	1.82E-07	8.90E-06	Up	AIF1L
TPSAB1	273.404944	0.80900038	0.15511107	5.21561991	1.83E-07	8.90E-06	Up	TPSAB1
KIF1A	62.1257988	1.20112287	0.23053279	5.21020404	1.89E-07	9.12E-06	Up	KIF1A

C1QB	3198.03462	0.65871094	0.12643139	5.21002701	1.89E-07	9.12E-06	Up	C1QB
EPHB6	98.1691821	0.75759795	0.14543023	5.20935676	1.89E-07	9.13E-06	Up	EPHB6
TGFBR3L	8.95387023	0.9502966	0.18246502	5.20810305	1.91E-07	9.17E-06	Up	TGFBR3L
MAS1	1.29243698	1.77146275	0.34034252	5.20494095	1.94E-07	9.29E-06	Up	MAS1
PLET1	1.21262455	2.46939742	0.47451434	5.20405222	1.95E-07	9.29E-06	Up	PLET1
SAA2-SAA4	17.2702536	1.47244754	0.28294259	5.20405057	1.95E-07	9.29E-06	Up	SAA2-SAA4
IFIT1B	0.92064606	2.17920293	0.41902606	5.20063822	1.99E-07	9.43E-06	Up	IFIT1B
KLF1	4.94556237	0.72713987	0.14005479	5.1918242	2.08E-07	9.84E-06	Up	KLF1
SLC6A12	51.2190077	0.71717626	0.13821638	5.18879339	2.12E-07	9.95E-06	Up	SLC6A12
GPC3	290.719407	0.92362125	0.17800205	5.18882366	2.12E-07	9.95E-06	Up	GPC3
NKG7	187.053828	0.72161126	0.1392123	5.18353103	2.18E-07	1.02E-05	Up	NKG7
APOBEC3G	266.79131	0.59217968	0.11439073	5.17681517	2.26E-07	1.05E-05	Up	APOBEC3G
CLC	73.6942094	1.3562645	0.26209192	5.17476647	2.28E-07	1.05E-05	Up	CLC
MYO1G	302.844651	0.56570067	0.10932581	5.17444776	2.29E-07	1.05E-05	Up	MYO1G
CLEC4D	7.5080186	1.12957117	0.21872899	5.16424995	2.41E-07	1.10E-05	Up	CLEC4D
CXCL10	896.32575	0.90489199	0.17520528	5.16475302	2.41E-07	1.10E-05	Up	CXCL10
HLA-DPA1	5192.85307	0.68467295	0.13273431	5.15822131	2.49E-07	1.13E-05	Up	HLA-DPA1
C12orf40	2.02250116	2.27002766	0.44021872	5.1565905	2.51E-07	1.13E-05	Up	C12orf40
TAS2R50	2.22570756	2.21902687	0.43052725	5.1542077	2.55E-07	1.14E-05	Up	TAS2R50
RERGL	12.9824151	1.61238381	0.31297061	5.15186966	2.58E-07	1.15E-05	Up	RERGL
IGSF6	327.764366	0.58399522	0.11362051	5.13987499	2.75E-07	1.22E-05	Up	IGSF6
ANPEP	6323.84801	1.15259136	0.22433421	5.13783152	2.78E-07	1.23E-05	Up	ANPEP
ACMSD	5.0196715	1.2351206	0.24094082	5.12624061	2.96E-07	1.30E-05	Up	ACMSD
HMSD	10.5208606	0.99006303	0.19334084	5.12081698	3.04E-07	1.34E-05	Up	HMSD
NKX2-5	2.22343283	1.81000154	0.35383628	5.1153645	3.13E-07	1.37E-05	Up	NKX2-5
ANO2	20.1340591	0.58233973	0.11398143	5.10907556	3.24E-07	1.41E-05	Up	ANO2
MROH2A	2.15438853	1.45590164	0.28494485	5.10941553	3.23E-07	1.41E-05	Up	MROH2A
NRXN1	29.2080991	1.47973729	0.2896924	5.10796033	3.26E-07	1.41E-05	Up	NRXN1
SERPINA5	51.8590853	0.79489043	0.15566591	5.10638727	3.28E-07	1.42E-05	Up	SERPINA5
PEX5L	4.41280981	1.05564024	0.20688207	5.10261823	3.35E-07	1.44E-05	Up	PEX5L
PCDHGA8	5.56447497	1.0873057	0.21330435	5.0974379	3.44E-07	1.48E-05	Up	PCDHGA8
KRT14	11.6578682	1.54886107	0.30391965	5.09628476	3.46E-07	1.49E-05	Up	KRT14
CLDN10	21.0484072	1.56546771	0.30722315	5.0955395	3.48E-07	1.49E-05	Up	CLDN10
HOXD3	14.6083167	0.77736107	0.15267803	5.0915056	3.55E-07	1.52E-05	Up	HOXD3
BCL2A1	163.341417	0.73410663	0.14430854	5.08706296	3.64E-07	1.54E-05	Up	BCL2A1
NKX6-3	13.8285353	2.04155812	0.40132656	5.08702467	3.64E-07	1.54E-05	Up	NKX6-3
TMIGD2	25.4147135	0.69170056	0.13601474	5.0854823	3.67E-07	1.54E-05	Up	TMIGD2
WFDC5	1.25829644	1.3544901	0.26636658	5.08506028	3.68E-07	1.54E-05	Up	WFDC5
TBC1D3L	7.98192502	-1.25265504	0.24645213	-5.08275183	3.72E-07	1.56E-05	Down	TBC1D3L
RHAG	2.58382796	2.69267262	0.53045279	5.07617769	3.85E-07	1.60E-05	Up	RHAG
BMP3	197.176111	1.38465342	0.27304072	5.07123411	3.95E-07	1.63E-05	Up	BMP3
MT1H	203.652408	1.07761158	0.212474	5.07173381	3.94E-07	1.63E-05	Up	MT1H
HSD17B3	18.3374102	-0.77944195	0.1538576	-5.0659958	4.06E-07	1.67E-05	Down	HSD17B3
NTNG1	10.9840961	1.26592227	0.24993296	5.06504738	4.08E-07	1.67E-05	Up	NTNG1
RPS5	41175.3282	0.51478677	0.10188868	5.05244315	4.36E-07	1.77E-05	Up	RPS5
SH2B2	194.004174	0.50564676	0.10010323	5.05125322	4.39E-07	1.78E-05	Up	SH2B2
TRPC5	1.37364672	2.1764307	0.43132712	5.04589349	4.51E-07	1.82E-05	Up	TRPC5
C17orf78	34.6973732	1.49718665	0.2967581	5.04514168	4.53E-07	1.82E-05	Up	C17orf78
CD69	136.53893	0.70598923	0.1399606	5.04419986	4.55E-07	1.83E-05	Up	CD69
B3GNT4	45.8100169	0.59514834	0.11800348	5.04348109	4.57E-07	1.83E-05	Up	B3GNT4
TUBB4A	24.3871401	1.01573983	0.20148852	5.04117957	4.63E-07	1.85E-05	Up	TUBB4A
KYNU	171.577404	0.68731458	0.13632918	5.04158097	4.62E-07	1.85E-05	Up	KYNU
SUCNR1	58.4416921	0.76837119	0.15244224	5.04040884	4.65E-07	1.85E-05	Up	SUCNR1
PRSS48	3.2039208	1.56568846	0.31159127	5.02481499	5.04E-07	1.99E-05	Up	PRSS48
MAL	30.6391027	0.97749403	0.1946562	5.02164338	5.12E-07	2.02E-05	Up	MAL
KMO	36.1510381	0.63371627	0.1262145	5.02094677	5.14E-07	2.02E-05	Up	KMO
EBI3	59.7345728	0.60339452	0.12026056	5.01739315	5.24E-07	2.05E-05	Up	EBI3
PHYHIPL	87.9484884	1.25786381	0.25072375	5.01693127	5.25E-07	2.05E-05	Up	PHYHIPL

TNFRSF18	102.874336	0.61972738	0.12370075	5.0098917	5.45E-07	2.12E-05	Up	TNFRSF18
KLHDC7B	53.1351687	0.66448228	0.13287953	5.00063674	5.71E-07	2.22E-05	Up	KLHDC7B
GCG	88.4520584	1.68494557	0.33699049	4.99997956	5.73E-07	2.22E-05	Up	GCG
HLA-DRB1	8343.91196	0.62748517	0.12552041	4.99906868	5.76E-07	2.23E-05	Up	HLA-DRB1
MYOC	20.4109124	2.01643259	0.40370059	4.99487152	5.89E-07	2.26E-05	Up	MYOC
AFP	11.7917837	1.30852703	0.26251014	4.98467241	6.21E-07	2.36E-05	Up	AFP
NBPF6	6.45713437	-1.29495305	0.2598545	-4.98337736	6.25E-07	2.37E-05	Down	NBPF6
TACR2	194.295909	0.99098471	0.19896095	4.98080005	6.33E-07	2.38E-05	Up	TACR2
MT2A	2648.84216	0.69320883	0.13917559	4.98082196	6.33E-07	2.38E-05	Up	MT2A
TBR1	1.0719262	1.79770214	0.36097452	4.98013588	6.35E-07	2.39E-05	Up	TBR1
ATP8B3	37.6075808	0.64324207	0.12933369	4.97350756	6.58E-07	2.46E-05	Up	ATP8B3
KRT7	318.544134	0.87795362	0.17667406	4.96934087	6.72E-07	2.50E-05	Up	KRT7
IGF2	25446.297	1.43393713	0.28870248	4.96683344	6.81E-07	2.53E-05	Up	IGF2
CD274	155.423001	0.66696067	0.13433805	4.96479339	6.88E-07	2.54E-05	Up	CD274
NPIPB5	173.248661	0.72837834	0.14670647	4.96486857	6.87E-07	2.54E-05	Up	NPIPB5
EVA1B	327.643	0.55567087	0.11199432	4.96159885	6.99E-07	2.58E-05	Up	EVA1B
REG1A	10622.3029	1.50282347	0.30304833	4.95902247	7.08E-07	2.59E-05	Up	REG1A
MCEMP1	46.6447176	0.96887168	0.19535948	4.95943002	7.07E-07	2.59E-05	Up	MCEMP1
MROH9	1.52728449	2.54324848	0.51379912	4.94988873	7.43E-07	2.70E-05	Up	MROH9
LRRC26	343.810255	1.1997043	0.24239876	4.9493004	7.45E-07	2.71E-05	Up	LRRC26
GP2	96.9627256	1.4629079	0.29566832	4.94780058	7.51E-07	2.72E-05	Up	GP2
DNASE1L3	174.234658	1.03466082	0.20924512	4.94473106	7.62E-07	2.75E-05	Up	DNASE1L3
PRIMA1	70.1875986	1.27932371	0.25877635	4.9437428	7.66E-07	2.76E-05	Up	PRIMA1
PRB4	1.34760802	3.07164067	0.62163981	4.94119037	7.76E-07	2.79E-05	Up	PRB4
NR1H4	62.3292228	1.26772267	0.25682299	4.93617283	7.97E-07	2.84E-05	Up	NR1H4
AP3B2	22.171267	0.9587584	0.19421673	4.93653857	7.95E-07	2.84E-05	Up	AP3B2
HMX3	7.59369272	1.53063885	0.31006165	4.93656291	7.95E-07	2.84E-05	Up	HMX3
MAP3K19	2.73803191	1.14297656	0.23159895	4.93515438	8.01E-07	2.84E-05	Up	MAP3K19
TRIB2	780.679077	0.54653257	0.11091951	4.92729	8.34E-07	2.92E-05	Up	TRIB2
NR5A1	2.83433785	1.47054883	0.29838325	4.92838934	8.29E-07	2.92E-05	Up	NR5A1
KCTD4	9.23208188	1.35925406	0.27584802	4.92754681	8.33E-07	2.92E-05	Up	KCTD4
OLIG2	2.49019736	1.19419344	0.24233801	4.92780079	8.32E-07	2.92E-05	Up	OLIG2
HOXC6	58.9453217	1.22036762	0.24778209	4.92516487	8.43E-07	2.94E-05	Up	HOXC6
TAS2R31	4.91554422	1.14730468	0.23302392	4.92354905	8.50E-07	2.96E-05	Up	TAS2R31
PAPOLB	0.83553089	2.0128407	0.40909172	4.92026748	8.64E-07	3.00E-05	Up	PAPOLB
CXCR6	165.669347	0.57511939	0.11698645	4.91611951	8.83E-07	3.05E-05	Up	CXCR6
BSN	44.3682725	0.71063136	0.14456299	4.91572125	8.85E-07	3.05E-05	Up	BSN
PRB2	2.00992629	1.6060717	0.32689295	4.91314273	8.96E-07	3.08E-05	Up	PRB2
MEGF11	38.1140166	0.93152228	0.18986465	4.906244	9.28E-07	3.19E-05	Up	MEGF11
CCDC141	12.4182844	0.92437837	0.18875383	4.89726946	9.72E-07	3.31E-05	Up	CCDC141
APOA2	29.5593803	1.72462453	0.35219253	4.89682317	9.74E-07	3.32E-05	Up	APOA2
SYNM	1398.31881	1.06401345	0.21764134	4.88883886	1.01E-06	3.43E-05	Up	SYNM
ADH1B	523.589584	1.33159121	0.27244258	4.88760317	1.02E-06	3.45E-05	Up	ADH1B
TAS2R19	5.90405015	1.04602036	0.21445949	4.87747298	1.07E-06	3.61E-05	Up	TAS2R19
CD74	39179.118	0.56110418	0.11504977	4.87705628	1.08E-06	3.61E-05	Up	CD74
MAMDC2	100.974406	1.18596194	0.24330917	4.87430029	1.09E-06	3.66E-05	Up	MAMDC2
PDZRN4	53.2438878	1.13973057	0.23403343	4.86994766	1.12E-06	3.73E-05	Up	PDZRN4
ACY3	433.796405	0.56786042	0.11663804	4.86856974	1.12E-06	3.74E-05	Up	ACY3
RPL3L	8.58358417	-0.53182297	0.10941922	-4.86041646	1.17E-06	3.89E-05	Down	RPL3L
GUCA2A	1747.49547	1.24263989	0.25577573	4.85831822	1.18E-06	3.92E-05	Up	GUCA2A
LINGO3	16.6835443	0.80896971	0.1667526	4.8513169	1.23E-06	4.05E-05	Up	LINGO3
ARHGEF35	220.295619	-0.56008664	0.11547735	-4.85018594	1.23E-06	4.07E-05	Down	ARHGEF35
FOXD3	6.76537757	1.30608278	0.26951866	4.84598271	1.26E-06	4.13E-05	Up	FOXD3
SLC5A4	6.59111645	0.79022386	0.16313701	4.84392754	1.27E-06	4.17E-05	Up	SLC5A4
TAS2R46	1.49580352	1.90663617	0.39380683	4.84155183	1.29E-06	4.21E-05	Up	TAS2R46
CCL1	0.85999805	1.38766971	0.28671015	4.8399741	1.30E-06	4.23E-05	Up	CCL1
CD109	542.054802	0.82201115	0.16989008	4.83848825	1.31E-06	4.26E-05	Up	CD109
ELOVL3	8.43786956	0.79301319	0.16401638	4.83496344	1.33E-06	4.32E-05	Up	ELOVL3

GDF10	35.2131358	1.25343791	0.25923846	4.83507698	1.33E-06	4.32E-05	Up	GDF10
MAPK8IP2	158.639299	0.80641734	0.16686171	4.83284825	1.35E-06	4.36E-05	Up	MAPK8IP2
SDF2L1	1528.81935	0.51937986	0.1075677	4.82840003	1.38E-06	4.44E-05	Up	SDF2L1
S1PR4	90.5245332	0.59924741	0.12413161	4.82751654	1.38E-06	4.44E-05	Up	S1PR4
NOS2	2179.05937	0.84286412	0.1747437	4.82343058	1.41E-06	4.52E-05	Up	NOS2
LRRN3	26.2346603	0.83877074	0.17412657	4.8170175	1.46E-06	4.65E-05	Up	LRRN3
C11orf16	3.54321495	0.81573943	0.16935438	4.81676007	1.46E-06	4.65E-05	Up	C11orf16
ESR1	32.9903879	0.75857263	0.15755616	4.81461731	1.47E-06	4.69E-05	Up	ESR1
BEGAIN	45.6750538	0.61836713	0.12855976	4.80995855	1.51E-06	4.80E-05	Up	BEGAIN
KISS1R	11.5953631	1.03733325	0.21574318	4.80818553	1.52E-06	4.83E-05	Up	KISS1R
CFD	1117.64103	0.85096206	0.17702133	4.8071158	1.53E-06	4.85E-05	Up	CFD
PITX1	1765.30353	0.5159053	0.10734749	4.80593709	1.54E-06	4.87E-05	Up	PITX1
B4GALNT2	670.865728	1.41703339	0.29509417	4.80197018	1.57E-06	4.95E-05	Up	B4GALNT2
CD48	320.898366	0.62266399	0.12976211	4.79850375	1.60E-06	4.99E-05	Up	CD48
NCR1	5.03692153	0.86477049	0.18030095	4.79626133	1.62E-06	5.04E-05	Up	NCR1
FOXL2NB	2.5799161	1.85395954	0.38740005	4.78564614	1.70E-06	5.31E-05	Up	FOXL2NB
KIR2DL4	15.1625176	0.95306965	0.199347	4.78095799	1.74E-06	5.41E-05	Up	KIR2DL4
ENAM	11.6132512	1.10966186	0.23218529	4.77920829	1.76E-06	5.45E-05	Up	ENAM
RTBDN	8.36210017	1.01264828	0.21198299	4.77702623	1.78E-06	5.48E-05	Up	RTBDN
CMPK2	403.886721	0.54005411	0.11315875	4.7725352	1.82E-06	5.59E-05	Up	CMPK2
NPW	66.0293011	0.93335003	0.19564568	4.7706141	1.84E-06	5.64E-05	Up	NPW
ARC	33.9540468	0.88882797	0.18662362	4.76267674	1.91E-06	5.82E-05	Up	ARC
CA4	1597.15808	1.40232716	0.29448263	4.76200297	1.92E-06	5.83E-05	Up	CA4
CYSLTR2	27.5370789	0.66643155	0.13999169	4.76050781	1.93E-06	5.86E-05	Up	CYSLTR2
SLC13A5	10.3227386	0.9971263	0.20975629	4.75373737	2.00E-06	6.04E-05	Up	SLC13A5
KCNRG	17.0881831	0.62075182	0.13057592	4.75395317	1.99E-06	6.04E-05	Up	KCNRG
CIDEC	108.404996	0.86217035	0.1814346	4.75196211	2.01E-06	6.08E-05	Up	CIDEC
CCL22	168.460431	0.60746199	0.12785524	4.75117007	2.02E-06	6.10E-05	Up	CCL22
CDH20	1.96115612	1.10795261	0.23340776	4.74685431	2.07E-06	6.21E-05	Up	CDH20
CLDN5	349.654175	0.66299511	0.13968929	4.7462129	2.07E-06	6.22E-05	Up	CLDN5
NKX3-1	46.7659049	0.70857776	0.14933271	4.74496026	2.09E-06	6.24E-05	Up	NKX3-1
C1QTNF9B	2.69674337	-0.65255794	0.13757269	-4.74336838	2.10E-06	6.28E-05	Down	C1QTNF9B
IGFALS	81.8696849	1.14401713	0.24134817	4.74011112	2.14E-06	6.34E-05	Up	IGFALS
NUDT8	587.299261	0.55933352	0.11799787	4.74020014	2.14E-06	6.34E-05	Up	NUDT8
USH1G	4.15103474	1.23344483	0.26076853	4.73003713	2.24E-06	6.59E-05	Up	USH1G
PMEL	163.844173	0.53432762	0.11296812	4.72989724	2.25E-06	6.59E-05	Up	PMEL
IL4I1	313.117818	0.61029327	0.12910029	4.72728024	2.28E-06	6.65E-05	Up	IL4I1
RASGRP1	119.417385	0.60488541	0.12814098	4.72046826	2.35E-06	6.82E-05	Up	RASGRP1
SLA2	69.6523581	0.52399028	0.11103064	4.71933045	2.37E-06	6.83E-05	Up	SLA2
GATA5	10.2467755	1.35744867	0.28762699	4.71947599	2.36E-06	6.83E-05	Up	GATA5
IL18RAP	33.1244031	0.60674427	0.12866864	4.71555678	2.41E-06	6.94E-05	Up	IL18RAP
IL31RA	3.98186188	0.86162389	0.18286868	4.71170824	2.46E-06	7.03E-05	Up	IL31RA
HLA-DOA	515.344376	0.68590262	0.14579503	4.70456775	2.54E-06	7.24E-05	Up	HLA-DOA
TKTL1	8.63654749	1.26011463	0.26820164	4.69838527	2.62E-06	7.41E-05	Up	TKTL1
CACNA1E	32.2785725	0.85960832	0.18309539	4.69486608	2.67E-06	7.50E-05	Up	CACNA1E
C1QC	3194.12023	0.59167894	0.12606311	4.69351367	2.69E-06	7.53E-05	Up	C1QC
PLA2G2A	9007.72458	1.03134909	0.21978454	4.69254614	2.70E-06	7.55E-05	Up	PLA2G2A
ZBTB20	30.7295515	0.62210001	0.13267019	4.68907164	2.74E-06	7.63E-05	Up	ZBTB20
RPL36A-HNF	2.30401774	-0.6641226	0.14170777	-4.68656459	2.78E-06	7.71E-05	Down	RPL36A-HNRNPH2
SIGLEC12	71.6132563	0.747567	0.15953554	4.68589645	2.79E-06	7.72E-05	Up	SIGLEC12
COMTD1	1328.5046	0.5564304	0.11875674	4.6854637	2.79E-06	7.73E-05	Up	COMTD1
TNFSF9	316.194565	0.81882225	0.1748595	4.68274387	2.83E-06	7.82E-05	Up	TNFSF9
CFAP61	6.0117852	0.66202631	0.14144017	4.68061009	2.86E-06	7.88E-05	Up	CFAP61
FLG2	1.35932237	2.62126688	0.56035225	4.67789128	2.90E-06	7.95E-05	Up	FLG2
ZDHHC22	2.46545828	1.54111764	0.32953461	4.67664886	2.92E-06	7.98E-05	Up	ZDHHC22
CDH4	25.266431	-0.69866209	0.14943458	-4.6753711	2.93E-06	8.00E-05	Down	CDH4
CCDC169	8.04832107	1.23403952	0.26396483	4.67501499	2.94E-06	8.00E-05	Up	CCDC169
CRHBP	5.93193872	0.8108836	0.17353198	4.67281929	2.97E-06	8.05E-05	Up	CRHBP

EEF1G	18.9511405	-0.66237663	0.14183163	-4.67016164	3.01E-06	8.14E-05	Down	EEF1G
DIRAS2	6.49549716	1.06248113	0.22771906	4.66575414	3.07E-06	8.25E-05	Up	DIRAS2
LRP1B	5.09393965	1.1126213	0.23846484	4.6657666	3.07E-06	8.25E-05	Up	LRP1B
LST1	211.487344	0.50402994	0.10808187	4.66340874	3.11E-06	8.34E-05	Up	LST1
LEP	7.59903303	1.32576474	0.2844613	4.66061548	3.15E-06	8.42E-05	Up	LEP
POMC	14.8819992	0.66894061	0.14363162	4.65733527	3.20E-06	8.48E-05	Up	POMC
CYP4F22	5.41275376	0.67523159	0.14496475	4.65790189	3.19E-06	8.48E-05	Up	CYP4F22
GATA1	4.16398048	0.83609523	0.17959655	4.6554081	3.23E-06	8.53E-05	Up	GATA1
NAT16	8.0903934	-0.98456954	0.21151857	-4.6547664	3.24E-06	8.55E-05	Down	NAT16
HLA-DPB1	4042.31629	0.57722182	0.12424721	4.64575268	3.39E-06	8.88E-05	Up	HLA-DPB1
CAPN14	15.7513241	0.98017445	0.21114858	4.6421077	3.45E-06	8.99E-05	Up	CAPN14
RNF165	13.7945901	0.86171042	0.18570143	4.64030034	3.48E-06	9.06E-05	Up	RNF165
SEMG2	2.69122874	2.23246578	0.48149754	4.63650506	3.54E-06	9.21E-05	Up	SEMG2
INSL5	44.4878737	1.83725668	0.3967817	4.63039667	3.65E-06	9.43E-05	Up	INSL5
ZNF483	9.90768588	0.86514655	0.18685756	4.62997882	3.66E-06	9.43E-05	Up	ZNF483
KLRC1	11.951383	0.80296241	0.17350465	4.62790144	3.69E-06	9.51E-05	Up	KLRC1
TYROBP	1024.61621	0.55306783	0.11959076	4.62467045	3.75E-06	9.65E-05	Up	TYROBP
DMRT1	4.67977868	1.81572696	0.39329747	4.61667588	3.90E-06	9.97E-05	Up	DMRT1
TIFAB	23.5842955	0.69682219	0.15100014	4.61471213	3.94E-06	0.0001	Up	TIFAB
CLIC3	275.979148	0.748004	0.16223663	4.61057417	4.02E-06	0.000102	Up	CLIC3
ICOS	52.6876503	0.58417923	0.12677807	4.60788871	4.07E-06	0.000103	Up	ICOS
CHD5	22.7500281	0.68133641	0.14791597	4.60623953	4.10E-06	0.000104	Up	CHD5
ROBO3	110.434311	0.523344	0.1137074	4.60255002	4.17E-06	0.000105	Up	ROBO3
CD300LG	7.80876489	1.34270829	0.2918976	4.59992914	4.23E-06	0.000106	Up	CD300LG
TLX3	3.36684189	1.7648126	0.38390869	4.59695923	4.29E-06	0.000108	Up	TLX3
SMC1B	12.3672435	0.8331902	0.18132043	4.59512577	4.32E-06	0.000108	Up	SMC1B
ATP8B4	87.4783027	0.53830881	0.11718853	4.59352818	4.36E-06	0.000109	Up	ATP8B4
FABP4	93.4423048	1.09067275	0.23760006	4.5903724	4.42E-06	0.00011	Up	FABP4
GZMM	38.8298857	0.6572213	0.1431706	4.59047678	4.42E-06	0.00011	Up	GZMM
DOK2	224.124583	0.51690675	0.11267563	4.5875647	4.48E-06	0.000111	Up	DOK2
NOX5	6.19940002	0.79888193	0.17414259	4.58751602	4.49E-06	0.000111	Up	NOX5
INA	20.7578637	0.99280982	0.21650858	4.58554493	4.53E-06	0.000112	Up	INA
CD226	59.4694391	0.62902292	0.13728702	4.58180905	4.61E-06	0.000113	Up	CD226
APLP1	57.6607192	0.60611441	0.13248304	4.57503398	4.76E-06	0.000117	Up	APLP1
ZNF750	28.4085125	1.0252024	0.22424194	4.57185831	4.83E-06	0.000118	Up	ZNF750
PLEKHG4B	13.1239519	0.84605599	0.18528011	4.56636156	4.96E-06	0.000121	Up	PLEKHG4B
CYP2C18	116.042686	0.76272254	0.167173	4.5624744	5.06E-06	0.000123	Up	CYP2C18
SLC25A27	200.654308	-0.52208911	0.11445037	-4.56170752	5.07E-06	0.000123	Down	SLC25A27
ACTBL2	2.72199706	1.34622884	0.29510403	4.56187894	5.07E-06	0.000123	Up	ACTBL2
EVI2A	160.27637	0.64854228	0.14220104	4.56074217	5.10E-06	0.000124	Up	EVI2A
TSPAN32	26.6743081	0.73885068	0.16225057	4.55376329	5.27E-06	0.000127	Up	TSPAN32
ADAM20	2.40393422	1.13054058	0.24836533	4.55192595	5.32E-06	0.000128	Up	ADAM20
GALR1	3.39090003	1.46471939	0.32176603	4.55212567	5.31E-06	0.000128	Up	GALR1
CD3G	102.881993	0.54619096	0.1201334	4.54653714	5.45E-06	0.00013	Up	CD3G
OR1L8	1.07680559	1.2642491	0.27813505	4.54545054	5.48E-06	0.000131	Up	OR1L8
SERPINA3	4.25485353	1.14021554	0.25098707	4.54292542	5.55E-06	0.000132	Up	SERPINA3
GALR2	10.4520229	0.71605622	0.15783401	4.53676768	5.71E-06	0.000136	Up	GALR2
VSIG1	35.036318	0.90117374	0.19876839	4.53378797	5.79E-06	0.000137	Up	VSIG1
MYH1	1.86634873	1.50668098	0.33235418	4.53335954	5.81E-06	0.000137	Up	MYH1
TNFSF14	59.1006077	0.59022793	0.13022786	4.53227076	5.84E-06	0.000137	Up	TNFSF14
TLL2	35.6494191	0.59783578	0.13192706	4.53156294	5.85E-06	0.000138	Up	TLL2
PCSK2	16.8867813	1.45014085	0.32036386	4.5265432	6.00E-06	0.00014	Up	PCSK2
PTGS2	568.874045	0.78140956	0.17264375	4.52613866	6.01E-06	0.00014	Up	PTGS2
CLEC12B	1.76480836	1.10766172	0.24502161	4.52066947	6.16E-06	0.000143	Up	CLEC12B
SAA2	104.830736	0.93398196	0.20667728	4.51903556	6.21E-06	0.000144	Up	SAA2
IL17REL	13.5528302	0.95846105	0.21209761	4.51896212	6.21E-06	0.000144	Up	IL17REL
HEMGN	1.69161963	1.67516252	0.37126257	4.51206944	6.42E-06	0.000149	Up	HEMGN
TNFRSF9	102.873927	0.53528082	0.11877175	4.50680262	6.58E-06	0.000152	Up	TNFRSF9

ALPL	171.070891	0.52193806	0.11582512	4.5062598	6.60E-06	0.000152	Up	ALPL
LTA	20.0735695	0.54218528	0.12035436	4.50490776	6.64E-06	0.000153	Up	LTA
CSF2	23.8769423	0.87983792	0.1954883	4.50071913	6.77E-06	0.000156	Up	CSF2
CNR1	42.7125873	1.0688267	0.23751105	4.50011366	6.79E-06	0.000156	Up	CNR1
CLEC10A	130.421139	0.75693598	0.16822797	4.49946572	6.81E-06	0.000156	Up	CLEC10A
KLF2	742.868159	0.52068676	0.11579052	4.49679951	6.90E-06	0.000157	Up	KLF2
IDO2	4.5987077	0.72393603	0.16116591	4.49186816	7.06E-06	0.00016	Up	IDO2
XKR4	13.6612354	1.22231901	0.27216531	4.49109049	7.09E-06	0.000161	Up	XKR4
MSH4	10.4238582	0.91829708	0.20448544	4.49076997	7.10E-06	0.000161	Up	MSH4
GPR141	14.2858783	0.71241393	0.15868742	4.48941648	7.14E-06	0.000161	Up	GPR141
PRDM8	118.337438	0.68399702	0.15244681	4.48679128	7.23E-06	0.000163	Up	PRDM8
TMEM145	26.6409008	0.70628837	0.15744206	4.48602078	7.26E-06	0.000163	Up	TMEM145
CD3E	388.966931	0.52527997	0.11716628	4.48320096	7.35E-06	0.000165	Up	CD3E
ANKRD45	9.85968597	0.92610517	0.20673311	4.47971391	7.47E-06	0.000167	Up	ANKRD45
AVPR2	24.2770763	0.72776751	0.1625038	4.47846465	7.52E-06	0.000168	Up	AVPR2
PDE1B	68.0169749	0.5735657	0.12813141	4.47638624	7.59E-06	0.000169	Up	PDE1B
APOL1	3857.97839	0.54611861	0.12203259	4.47518664	7.63E-06	0.00017	Up	APOL1
SIT1	104.608931	0.61317636	0.13702575	4.47489872	7.64E-06	0.00017	Up	SIT1
TBC1D10C	202.099417	0.51068904	0.11412286	4.4749058	7.64E-06	0.00017	Up	TBC1D10C
TCF23	6.0295702	1.09700545	0.24553878	4.46774819	7.90E-06	0.000175	Up	TCF23
SYT8	76.91747	0.73764522	0.16527777	4.46306366	8.08E-06	0.000178	Up	SYT8
CA2	4959.66433	1.01637988	0.22776387	4.4624281	8.10E-06	0.000178	Up	CA2
TM4SF4	91.3479609	1.00533166	0.22528818	4.46242519	8.10E-06	0.000178	Up	TM4SF4
TAGLN3	12.3580978	1.09890847	0.2465812	4.45657842	8.33E-06	0.000183	Up	TAGLN3
FTCD	7.41871631	1.05361083	0.23642039	4.45651418	8.33E-06	0.000183	Up	FTCD
GPT	577.394562	0.78433948	0.1761054	4.45380706	8.44E-06	0.000185	Up	GPT
CCR3	15.6425091	0.73390052	0.16482727	4.4525431	8.49E-06	0.000186	Up	CCR3
CASP1	1614.73757	0.53913984	0.12113776	4.45063397	8.56E-06	0.000187	Up	CASP1
GCNT2	111.9993	0.79231029	0.1780625	4.44961895	8.60E-06	0.000188	Up	GCNT2
DUSP9	29.0118888	0.98958793	0.22256681	4.44625107	8.74E-06	0.00019	Up	DUSP9
CC2D2B	2.98468544	1.05755338	0.23798159	4.44384544	8.84E-06	0.000191	Up	CC2D2B
FCER1G	700.514184	0.50479377	0.11360892	4.4432584	8.86E-06	0.000192	Up	FCER1G
ATP4A	1.47958189	1.13530655	0.25561208	4.44152143	8.93E-06	0.000193	Up	ATP4A
CRP	4.23877561	2.37166232	0.53398761	4.4414183	8.94E-06	0.000193	Up	CRP
UNC80	4.15328442	0.99336181	0.22365153	4.44156053	8.93E-06	0.000193	Up	UNC80
IGDCC3	7.2016951	1.14150775	0.25703146	4.44112084	8.95E-06	0.000193	Up	IGDCC3
SAPCD1	29.0229292	-0.55547253	0.12509476	-4.44041402	8.98E-06	0.000193	Down	SAPCD1
TAF1L	2.04773496	0.84196927	0.18970125	4.4383961	9.06E-06	0.000194	Up	TAF1L
SLC7A3	2.90999247	0.99764485	0.22477388	4.43843757	9.06E-06	0.000194	Up	SLC7A3
KCNJ6	7.60201508	0.82770617	0.18664693	4.43460908	9.22E-06	0.000197	Up	KCNJ6
CXCL8	3542.73406	0.86699254	0.19561927	4.43204058	9.33E-06	0.000199	Up	CXCL8
HLA-DQB1	2954.31317	0.61922743	0.13989408	4.4264021	9.58E-06	0.000204	Up	HLA-DQB1
CD300LF	99.8114347	0.52262869	0.11812829	4.4242465	9.68E-06	0.000205	Up	CD300LF
ZBED2	35.1567413	0.78846221	0.17826832	4.42289577	9.74E-06	0.000206	Up	ZBED2
NEK5	116.322775	-0.57215747	0.1293751	-4.42246995	9.76E-06	0.000206	Down	NEK5
SLC25A48	37.7288768	0.71477605	0.1616467	4.42184134	9.79E-06	0.000206	Up	SLC25A48
AIPL1	0.72443464	1.79166963	0.40541187	4.41938125	9.90E-06	0.000208	Up	AIPL1
MS4A6A	723.813601	0.52522406	0.11885755	4.41893742	9.92E-06	0.000209	Up	MS4A6A
CD53	928.057561	0.52298884	0.11838938	4.41753152	9.98E-06	0.000209	Up	CD53
NRTN	70.4747892	0.50197995	0.11367632	4.41587107	1.01E-05	0.000211	Up	NRTN
MYH8	1.27471287	1.5062977	0.34134699	4.41280498	1.02E-05	0.000213	Up	MYH8
FNDC9	4.53698315	0.9756646	0.22113717	4.4120336	1.02E-05	0.000214	Up	FNDC9
LRRC4B	44.1685158	0.52085715	0.11822713	4.40556388	1.06E-05	0.000219	Up	LRRC4B
WDR64	1.73597764	1.03080817	0.2339592	4.40593136	1.05E-05	0.000219	Up	WDR64
CDC42EP5	2327.01188	0.53072555	0.1204605	4.40580548	1.05E-05	0.000219	Up	CDC42EP5
CFAP54	11.5698748	0.72818581	0.16530998	4.40497192	1.06E-05	0.000219	Up	CFAP54
FHL5	32.9644979	0.56458942	0.12821844	4.40334038	1.07E-05	0.00022	Up	FHL5
GSDMC	13.7652961	0.70042514	0.15908003	4.40297343	1.07E-05	0.00022	Up	GSDMC

RSPO4	16.5183134	1.23541803	0.28061916	4.40247208	1.07E-05	0.000221	Up	RSPO4
CRYBA2	20.2903666	0.98905799	0.22479957	4.39973257	1.08E-05	0.000223	Up	CRYBA2
MILR1	63.7048748	0.51455902	0.11710132	4.39413513	1.11E-05	0.000228	Up	MILR1
FAM162B	15.6627608	0.51110344	0.11632562	4.39373075	1.11E-05	0.000228	Up	FAM162B
FOLR2	260.110585	0.68970996	0.15706323	4.39128842	1.13E-05	0.00023	Up	FOLR2
SHISA8	2.60724377	0.98415096	0.22421135	4.38938967	1.14E-05	0.000232	Up	SHISA8
PDE10A	137.080327	0.68452323	0.15598386	4.38842336	1.14E-05	0.000233	Up	PDE10A
ISYNA1	645.306287	0.56359237	0.1284402	4.38797481	1.14E-05	0.000233	Up	ISYNA1
ADCYAP1R1	19.5580016	1.1341034	0.25867708	4.38424391	1.16E-05	0.000237	Up	ADCYAP1R1
KCNJ16	8.44224711	1.07518316	0.24531183	4.38292421	1.17E-05	0.000237	Up	KCNJ16
HMGN5	257.732329	-0.511276	0.11666966	-4.38225312	1.17E-05	0.000237	Down	HMGN5
VPREB3	29.328273	0.8072496	0.18443936	4.37677513	1.20E-05	0.000242	Up	VPREB3
CXCL5	941.767689	1.08121268	0.2472319	4.37327341	1.22E-05	0.000246	Up	CXCL5
DTHD1	10.0286829	0.89168122	0.20388981	4.37334867	1.22E-05	0.000246	Up	DTHD1
ITGA2B	13.1247997	0.57712474	0.13200781	4.37189842	1.23E-05	0.000247	Up	ITGA2B
SLC35G2	67.9203226	0.50577155	0.11568701	4.37189591	1.23E-05	0.000247	Up	SLC35G2
FAM183A	11.5574083	-0.8757965	0.20034323	-4.37148044	1.23E-05	0.000247	Down	FAM183A
MT1E	1698.04125	0.76807975	0.17573584	4.37064947	1.24E-05	0.000247	Up	MT1E
TRIM7	242.872475	0.84649876	0.19369563	4.37025224	1.24E-05	0.000248	Up	TRIM7
HCAR3	31.1893434	1.06440775	0.24362912	4.36896765	1.25E-05	0.000249	Up	HCAR3
PTPRU	623.217055	0.69202023	0.15859834	4.36335096	1.28E-05	0.000254	Up	PTPRU
FOLH1	58.0790017	0.53197566	0.12217988	4.35403667	1.34E-05	0.000264	Up	FOLH1
NFATC1	246.765401	0.51189655	0.11756207	4.35426607	1.34E-05	0.000264	Up	NFATC1
PRSS56	48.7098705	1.67659123	0.38508661	4.353803	1.34E-05	0.000264	Up	PRSS56
IGFBP1	23.858399	1.16888125	0.26851855	4.35307456	1.34E-05	0.000264	Up	IGFBP1
CEND1	12.6060009	0.66643944	0.15311977	4.35240631	1.35E-05	0.000264	Up	CEND1
LYPD5	147.928568	0.53023929	0.12188229	4.35042128	1.36E-05	0.000266	Up	LYPD5
GPM6A	55.2346865	1.21982186	0.28061818	4.34690954	1.38E-05	0.000269	Up	GPM6A
RAET1E	22.4750915	0.6026859	0.13881837	4.34154289	1.41E-05	0.000275	Up	RAET1E
C4orf45	2.18877592	1.44713958	0.33335356	4.34115534	1.42E-05	0.000275	Up	C4orf45
ART4	10.7914631	0.97301778	0.22423012	4.33937137	1.43E-05	0.000277	Up	ART4
AIM2	88.7950048	0.80130247	0.1848402	4.33510929	1.46E-05	0.000282	Up	AIM2
SEMG1	40.2190741	1.29924638	0.29973511	4.33464861	1.46E-05	0.000282	Up	SEMG1
FMO1	29.4030443	0.60744753	0.14032006	4.32901411	1.50E-05	0.000289	Up	FMO1
MAMLD1	103.690994	0.54987065	0.12711796	4.32567257	1.52E-05	0.000292	Up	MAMLD1
RIMS2	5.66839129	1.08841931	0.25197854	4.3194921	1.56E-05	0.0003	Up	RIMS2
MAPK4	23.951182	1.06690613	0.24706085	4.31839417	1.57E-05	0.000301	Up	MAPK4
GLB1L3	4.11199196	0.98666744	0.22852004	4.31764085	1.58E-05	0.000302	Up	GLB1L3
LBP	23.9859062	0.95098633	0.2203734	4.3153409	1.59E-05	0.000304	Up	LBP
C11orf21	25.365335	0.71497084	0.16574237	4.313748	1.61E-05	0.000306	Up	C11orf21
GABRG3	1.82650856	1.6162536	0.37479097	4.31241337	1.61E-05	0.000307	Up	GABRG3
MDK	5553.02562	0.50124601	0.11631237	4.30948162	1.64E-05	0.000309	Up	MDK
ERICH3	2.56008457	1.29615306	0.30080804	4.30890434	1.64E-05	0.00031	Up	ERICH3
SLC7A10	8.01708762	1.07394987	0.24927243	4.30833798	1.64E-05	0.00031	Up	SLC7A10
CLEC2B	150.485022	0.52005737	0.12074783	4.30697071	1.66E-05	0.000311	Up	CLEC2B
CGA	1.93349163	1.30940233	0.30401726	4.30699994	1.65E-05	0.000311	Up	CGA
MS4A18	0.86197781	1.83011244	0.4251868	4.30425502	1.68E-05	0.000314	Up	MS4A18
IL2RA	144.503461	0.52725382	0.12251823	4.30347244	1.68E-05	0.000315	Up	IL2RA
DPYD	358.861897	0.59668782	0.13876535	4.29997699	1.71E-05	0.000319	Up	DPYD
ALPP	22.2095785	1.18010903	0.27455265	4.29829774	1.72E-05	0.00032	Up	ALPP
POTEF	7.8190808	0.797231	0.1854772	4.29826943	1.72E-05	0.00032	Up	POTEF
CPS1	244.674931	0.99573432	0.23196273	4.29264793	1.77E-05	0.000326	Up	CPS1
TNNT3	4.52652056	1.04778644	0.24429136	4.28908514	1.79E-05	0.000331	Up	TNNT3
LIPF	1.10055915	2.49033	0.58075861	4.28806388	1.80E-05	0.000332	Up	LIPF
CEP85L	94.7197467	0.54696863	0.12756882	4.28763583	1.81E-05	0.000333	Up	CEP85L
TMPRSS15	1.81824459	1.81513736	0.42342315	4.28681649	1.81E-05	0.000334	Up	TMPRSS15
FAM216B	5.95215555	1.07162399	0.25002925	4.28599458	1.82E-05	0.000334	Up	FAM216B
MMP25	124.391433	0.51090474	0.11921359	4.285625	1.82E-05	0.000335	Up	MMP25

IFIT3	868.90833	0.51270039	0.1197165	4.28262082	1.85E-05	0.000338	Up	IFIT3
REG3A	3434.26894	1.36797587	0.31946425	4.28209374	1.85E-05	0.000339	Up	REG3A
SCRT2	0.85799979	1.84437975	0.43084346	4.28085823	1.86E-05	0.00034	Up	SCRT2
TNNI2	15.7928846	0.58303838	0.13620427	4.28061761	1.86E-05	0.00034	Up	TNNI2
IL1RAPL1	2.36924938	1.0891799	0.25455156	4.27881847	1.88E-05	0.000342	Up	IL1RAPL1
MDGA2	0.9511751	1.68964346	0.39504844	4.27705384	1.89E-05	0.000345	Up	MDGA2
ISG15	1822.56358	0.55798109	0.13048834	4.27609923	1.90E-05	0.000345	Up	ISG15
ANGPTL1	114.72777	1.05097378	0.24616758	4.26934284	1.96E-05	0.000354	Up	ANGPTL1
ALDH1A2	117.451169	0.99304293	0.23258691	4.26955633	1.96E-05	0.000354	Up	ALDH1A2
ACTG2	6763.7331	0.89156032	0.20899274	4.26598711	1.99E-05	0.000359	Up	ACTG2
CD37	380.234599	0.53725957	0.12597406	4.26484293	2.00E-05	0.000359	Up	CD37
TMEM132E	12.061815	0.82770075	0.19406019	4.26517546	2.00E-05	0.000359	Up	TMEM132E
EPPIN	1.77449703	1.60766827	0.37766005	4.25691901	2.07E-05	0.00037	Up	EPPIN
TPSD1	52.3695556	1.03140762	0.24238126	4.25531082	2.09E-05	0.000372	Up	TPSD1
HSD17B2	895.093282	0.80982724	0.19062594	4.24825315	2.15E-05	0.000383	Up	HSD17B2
SIGLEC15	77.0313476	0.73730892	0.17356181	4.24810586	2.16E-05	0.000383	Up	SIGLEC15
RD3	1.69656963	1.10667497	0.26051503	4.24802739	2.16E-05	0.000383	Up	RD3
CTLA4	73.2783697	0.53114163	0.12505054	4.24741553	2.16E-05	0.000383	Up	CTLA4
WFDC10B	11.0100791	-0.70629325	0.16642231	-4.24398171	2.20E-05	0.000388	Down	WFDC10B
KRT24	8.10344749	2.59543531	0.6116721	4.24318079	2.20E-05	0.000389	Up	KRT24
ATP2B3	3.55344469	1.1478043	0.2706903	4.24028598	2.23E-05	0.000393	Up	ATP2B3
HLA-DRB5	2787.3386	0.64729386	0.15265725	4.24017756	2.23E-05	0.000393	Up	HLA-DRB5
PLAC8	2187.70058	0.75396612	0.17786992	4.23886233	2.25E-05	0.000393	Up	PLAC8
STX11	85.6392212	0.5071421	0.11967531	4.23765033	2.26E-05	0.000395	Up	STX11
TMEM169	15.9898356	0.52635468	0.12424312	4.23648942	2.27E-05	0.000397	Up	TMEM169
GPER1	122.519583	0.65374537	0.15458059	4.22915558	2.35E-05	0.000408	Up	GPER1
CTCFL	2.64714734	1.08619762	0.25685385	4.22885468	2.35E-05	0.000408	Up	CTCFL
CD80	32.3762633	0.5309891	0.12572324	4.22347613	2.41E-05	0.000417	Up	CD80
DLX1	10.3488935	0.98078729	0.23227267	4.22256858	2.42E-05	0.000418	Up	DLX1
MT1G	1914.26912	0.80025843	0.18961302	4.22048254	2.44E-05	0.000421	Up	MT1G
HTR6	2.13084562	0.80608404	0.19099707	4.22040012	2.44E-05	0.000421	Up	HTR6
SCN2A	16.0451556	0.91272458	0.21641466	4.21748041	2.47E-05	0.000425	Up	SCN2A
TXNDC2	1.17463378	1.01901204	0.24160021	4.21776132	2.47E-05	0.000425	Up	TXNDC2
KCNK7	23.5720989	0.53594341	0.12710733	4.21646352	2.48E-05	0.000426	Up	KCNK7
MROH2B	1.60769429	1.84591263	0.43815542	4.21291745	2.52E-05	0.000432	Up	MROH2B
CEACAM7	12490.8161	0.99112649	0.2354079	4.21025163	2.55E-05	0.000436	Up	CEACAM7
WNT10B	26.7505655	0.63097039	0.14998407	4.20691605	2.59E-05	0.000442	Up	WNT10B
CNTNAP4	1.85665082	1.8219991	0.43315969	4.20629879	2.60E-05	0.000443	Up	CNTNAP4
HOXC8	10.8442302	1.13844895	0.27074287	4.20490835	2.61E-05	0.000444	Up	HOXC8
BIRC3	1516.00371	0.50956186	0.12119172	4.20459321	2.62E-05	0.000445	Up	BIRC3
CLDN8	165.417731	1.70761205	0.40663523	4.19937068	2.68E-05	0.000453	Up	CLDN8
CD5L	1.30136416	1.6404269	0.39071964	4.19847565	2.69E-05	0.000455	Up	CD5L
CLEC1B	2.1016685	1.1411889	0.27201298	4.1953473	2.72E-05	0.00046	Up	CLEC1B
LRP2	7.41138976	1.1649163	0.27770285	4.19483011	2.73E-05	0.000461	Up	LRP2
KCNJ9	4.62990604	0.86146685	0.2053913	4.1942713	2.74E-05	0.000462	Up	KCNJ9
DHH	9.44409553	0.54472784	0.12991937	4.19281476	2.76E-05	0.000464	Up	DHH
LDHAL6B	4.47188589	0.77322873	0.18445227	4.19202623	2.76E-05	0.000465	Up	LDHAL6B
PDE4B	523.178425	0.51281674	0.12239187	4.18995744	2.79E-05	0.000469	Up	PDE4B
MIA	36.8062154	0.96364533	0.23009277	4.1880731	2.81E-05	0.000472	Up	MIA
HIGD1C	0.79921117	1.92301734	0.45943432	4.18561972	2.84E-05	0.000476	Up	HIGD1C
METRN	727.300129	0.54707735	0.13074523	4.18430072	2.86E-05	0.000478	Up	METRN
FPR2	62.1873456	0.8177424	0.19590145	4.17425399	2.99E-05	0.000495	Up	FPR2
TCTE1	2.54047822	0.92351734	0.22127644	4.17359087	3.00E-05	0.000495	Up	TCTE1
SLC34A2	15.3308089	1.05822613	0.25369188	4.17130474	3.03E-05	0.000498	Up	SLC34A2
C19orf18	12.1299993	-0.66003505	0.15828008	-4.17004498	3.05E-05	0.0005	Down	C19orf18
NTRK1	13.6186035	0.57158534	0.13706967	4.17003528	3.05E-05	0.0005	Up	NTRK1
RPEL1	3.16596728	0.72950119	0.17508417	4.1665741	3.09E-05	0.000507	Up	RPEL1
KLHL14	17.0976093	0.91042926	0.21871752	4.16258032	3.15E-05	0.000515	Up	KLHL14

KIF4B	3.39659004	0.68788127	0.16531517	4.16102925	3.17E-05	0.000517	Up	KIF4B
LGSN	13.5863943	1.46538254	0.35228841	4.15961041	3.19E-05	0.00052	Up	LGSN
RGS18	44.5450368	0.56812968	0.13662541	4.15830161	3.21E-05	0.000522	Up	RGS18
NBEAL1	439.653223	0.53056631	0.12762077	4.15736665	3.22E-05	0.000524	Up	NBEAL1
KCNB1	26.3289339	0.99186405	0.23865812	4.15600382	3.24E-05	0.000527	Up	KCNB1
MAFA	3.59394037	0.8075491	0.19439534	4.15415875	3.26E-05	0.00053	Up	MAFA
AVPR1A	58.6509906	0.65206465	0.15705132	4.15192074	3.30E-05	0.000532	Up	AVPR1A
ZBED6	123.851988	0.62741133	0.15112541	4.1515939	3.30E-05	0.000532	Up	ZBED6
MYH11	15493.6582	0.95165959	0.22929067	4.15045054	3.32E-05	0.000534	Up	MYH11
UGT2B17	2317.45077	1.22681487	0.29576159	4.1479858	3.35E-05	0.000539	Up	UGT2B17
ERVV-2	1.85254361	1.78514351	0.43034747	4.1481446	3.35E-05	0.000539	Up	ERVV-2
ADAM21	4.15156106	0.85696992	0.20667597	4.14644203	3.38E-05	0.000541	Up	ADAM21
ACTN3	2.60238749	0.66353543	0.16001841	4.14661927	3.37E-05	0.000541	Up	ACTN3
SOAT2	7.78498007	1.12978065	0.27261709	4.14420328	3.41E-05	0.000545	Up	SOAT2
LRRC71	2.96450345	0.82655443	0.19946216	4.14391602	3.41E-05	0.000545	Up	LRRC71
CD79B	123.550358	0.62661501	0.15149171	4.13629911	3.53E-05	0.000561	Up	CD79B
LHX2	6.41550302	0.64859772	0.15683979	4.13541565	3.54E-05	0.000562	Up	LHX2
ATP1A2	106.067681	1.00108938	0.24233884	4.13094899	3.61E-05	0.00057	Up	ATP1A2
SCIMP	97.1435135	0.51206768	0.12394475	4.13141891	3.61E-05	0.00057	Up	SCIMP
LILRA5	84.9780975	0.53986389	0.130687	4.13096857	3.61E-05	0.00057	Up	LILRA5
M1AP	12.2585697	0.81839283	0.19819292	4.12927379	3.64E-05	0.000573	Up	M1AP
MMP12	2145.27157	0.65739365	0.15925234	4.12799991	3.66E-05	0.000575	Up	MMP12
PLEK	638.955434	0.58240762	0.14112586	4.12686671	3.68E-05	0.000578	Up	PLEK
LCT	8.73117493	0.89884628	0.21806578	4.12190434	3.76E-05	0.000587	Up	LCT
FGF23	3.62693887	1.58138569	0.38366325	4.1218065	3.76E-05	0.000587	Up	FGF23
TMEM163	62.208757	0.56305537	0.1365983	4.12197947	3.76E-05	0.000587	Up	TMEM163
MT1F	476.0844	0.63448465	0.15393162	4.1218604	3.76E-05	0.000587	Up	MT1F
KCNA7	1.45498886	1.25796881	0.30532215	4.12013615	3.79E-05	0.00059	Up	KCNA7
PCDHGB2	52.4074108	0.65713666	0.15968313	4.11525402	3.87E-05	0.000599	Up	PCDHGB2
AMER2	1.84639794	1.45523494	0.35367352	4.11462788	3.88E-05	0.0006	Up	AMER2
PEG10	251.236747	0.95203014	0.23175648	4.10789001	3.99E-05	0.000614	Up	PEG10
FPR1	231.444146	0.64259105	0.15661947	4.10288109	4.08E-05	0.000627	Up	FPR1
IL1RN	666.931662	0.61566652	0.1500895	4.10199597	4.10E-05	0.000627	Up	IL1RN
RADIL	30.65167	0.67870355	0.16547943	4.10143767	4.11E-05	0.000627	Up	RADIL
ZMAT1	108.94746	-0.65617256	0.15998142	-4.10155475	4.10E-05	0.000627	Down	ZMAT1
MAGEA1	3.27048627	2.55339227	0.622843	4.09957609	4.14E-05	0.000631	Up	MAGEA1
ST18	27.4959939	0.76131328	0.18574598	4.09867965	4.16E-05	0.000631	Up	ST18
HCST	73.8363625	0.50985693	0.12442815	4.09760102	4.17E-05	0.000633	Up	HCST
FBXO39	6.21210972	0.74843606	0.18265391	4.09756395	4.18E-05	0.000633	Up	FBXO39
SNAP25	26.7721902	0.87693763	0.21402762	4.09731056	4.18E-05	0.000633	Up	SNAP25
BLK	40.6012796	0.82520964	0.20146876	4.0959682	4.20E-05	0.000635	Up	BLK
MRAP	1.95007788	1.11010305	0.27124412	4.09263449	4.26E-05	0.000643	Up	MRAP
UGT1A4	0.80734572	2.21248945	0.54106214	4.08915962	4.33E-05	0.000653	Up	UGT1A4
ASB11	1.22822776	1.28075569	0.31345312	4.08595611	4.39E-05	0.000661	Up	ASB11
VSIG4	434.280481	0.60697839	0.14864333	4.08345529	4.44E-05	0.000666	Up	VSIG4
XG	25.6344951	0.78479904	0.19238885	4.07923351	4.52E-05	0.000677	Up	XG
VAX2	7.9041291	0.76932074	0.18865497	4.07792461	4.54E-05	0.00068	Up	VAX2
CCL17	14.2438654	0.64786156	0.15889499	4.07729375	4.56E-05	0.000681	Up	CCL17
EFNA2	375.520855	0.51468348	0.12629922	4.07511215	4.60E-05	0.000686	Up	EFNA2
CYBB	1274.41871	0.52014969	0.12768727	4.07362226	4.63E-05	0.000689	Up	CYBB
PCDHGB4	21.0867254	0.79609952	0.19541907	4.07380683	4.63E-05	0.000689	Up	PCDHGB4
CCBE1	64.9478672	0.90849735	0.22309348	4.07227207	4.66E-05	0.000692	Up	CCBE1
IGSF11	8.23130967	0.98381105	0.24189003	4.06718308	4.76E-05	0.000704	Up	IGSF11
IKZF1	333.955721	0.52467905	0.12901098	4.06693341	4.76E-05	0.000704	Up	IKZF1
CADM2	13.999134	1.23807732	0.30450604	4.06585476	4.79E-05	0.000707	Up	CADM2
C5orf58	8.02708911	0.77204313	0.19014085	4.06037481	4.90E-05	0.000723	Up	C5orf58
WFDC8	0.85376099	1.98759725	0.48964485	4.05926305	4.92E-05	0.000724	Up	WFDC8
ZNF488	117.739099	0.62936849	0.1550377	4.05945447	4.92E-05	0.000724	Up	ZNF488

GAPT	32.7232523	0.62458598	0.15394172	4.05728869	4.96E-05	0.00073	Up	GAPT
TLR8	82.6597544	0.64270209	0.15873056	4.04901291	5.14E-05	0.000754	Up	TLR8
KLRD1	105.818502	0.64643193	0.15978685	4.045589	5.22E-05	0.000764	Up	KLRD1
INSC	59.592156	0.58459882	0.14458003	4.04342714	5.27E-05	0.000769	Up	INSC
PCDHA11	4.62839475	1.18596063	0.2933107	4.04335953	5.27E-05	0.000769	Up	PCDHA11
MTRNR2L8	40.7194604	0.74804685	0.18502023	4.04305434	5.28E-05	0.00077	Up	MTRNR2L8
BFSP2	5.09197345	0.73703677	0.18238282	4.04115247	5.32E-05	0.000775	Up	BFSP2
CITED4	346.088849	0.55243799	0.13689759	4.03541069	5.45E-05	0.000793	Up	CITED4
WNK3	11.8883105	0.56374934	0.1397226	4.03477573	5.47E-05	0.000795	Up	WNK3
NECAB1	35.1218231	0.73951015	0.18334204	4.03350012	5.50E-05	0.000798	Up	NECAB1
IL5RA	8.27813565	0.7347266	0.18239038	4.02831887	5.62E-05	0.000812	Up	IL5RA
WFDC2	1926.45791	0.77955958	0.19362849	4.02605833	5.67E-05	0.000818	Up	WFDC2
ESRRB	3.44949064	0.75191451	0.1868074	4.02507885	5.70E-05	0.00082	Up	ESRRB
IGSF21	33.5709646	0.52070322	0.12944045	4.02272423	5.75E-05	0.000824	Up	IGSF21
GTSF1	16.4590981	0.84434229	0.20987704	4.02303322	5.75E-05	0.000824	Up	GTSF1
SRRM3	71.4522818	0.54701264	0.13597539	4.02287975	5.75E-05	0.000824	Up	SRRM3
ALPI	162.36369	0.8774086	0.21814441	4.02214564	5.77E-05	0.000825	Up	ALPI
ARRDC5	4.25888452	0.63276138	0.15733739	4.02168473	5.78E-05	0.000826	Up	ARRDC5
MYL3	9.73484062	0.86700784	0.21565922	4.02026789	5.81E-05	0.000829	Up	MYL3
SPRR3	18.7048662	1.51597771	0.3772022	4.01900547	5.84E-05	0.000833	Up	SPRR3
CHRNB2	16.1782909	0.71827852	0.17883043	4.01653399	5.91E-05	0.000839	Up	CHRNB2
GMNC	1.00895775	1.84124015	0.45845578	4.01617826	5.91E-05	0.00084	Up	GMNC
NCF1	76.7665867	0.53020116	0.13207575	4.01437164	5.96E-05	0.000845	Up	NCF1
LRRC73	41.6943184	0.54192864	0.13507903	4.01193756	6.02E-05	0.000853	Up	LRRC73
SPIB	201.492509	0.90959495	0.22675635	4.01133185	6.04E-05	0.000855	Up	SPIB
TBX21	22.8787727	0.56201903	0.14028199	4.00635204	6.17E-05	0.000871	Up	TBX21
CCL13	101.307622	0.82109165	0.20521366	4.00115493	6.30E-05	0.000888	Up	CCL13
MT1HL1	0.46152062	1.49635824	0.37435172	3.99719874	6.41E-05	0.000901	Up	MT1HL1
PPP1R27	1.97399941	0.8182496	0.20475099	3.99631572	6.43E-05	0.000902	Up	PPP1R27
HDC	40.9089082	0.59356399	0.14872784	3.99094064	6.58E-05	0.000919	Up	HDC
CPA3	304.112031	0.63655657	0.15963862	3.98748489	6.68E-05	0.000929	Up	CPA3
MGAT5B	9.0250257	0.67415313	0.16908808	3.98699377	6.69E-05	0.00093	Up	MGAT5B
HOXD1	22.3509258	0.71086833	0.17834329	3.98595492	6.72E-05	0.000933	Up	HOXD1
GDF3	2.00146164	0.8365072	0.20994311	3.98444706	6.76E-05	0.000937	Up	GDF3
MS4A7	394.043126	0.52962888	0.13297315	3.98297599	6.81E-05	0.00094	Up	MS4A7
LRRC74B	1.64694466	1.15117258	0.28900909	3.98317078	6.80E-05	0.00094	Up	LRRC74B
C11orf86	187.984395	0.97244429	0.24424551	3.98142138	6.85E-05	0.000944	Up	C11orf86
LCK	511.634663	0.52053971	0.13074429	3.981357	6.85E-05	0.000944	Up	LCK
CYP4A22	1.5589242	1.78484328	0.44872756	3.97756556	6.96E-05	0.000957	Up	CYP4A22
DEFB4A	2.03100374	1.69361033	0.42624167	3.97335703	7.09E-05	0.000972	Up	DEFB4A
CLEC5A	105.579536	0.69349279	0.17459925	3.97191154	7.13E-05	0.000974	Up	CLEC5A
HPCAL4	4.6494962	0.75713573	0.19066762	3.97097179	7.16E-05	0.000976	Up	HPCAL4
BPIFB1	3.83292941	1.5946627	0.40163745	3.97040336	7.18E-05	0.000977	Up	BPIFB1
PM20D1	4.94756846	0.80757473	0.20346581	3.96909308	7.21E-05	0.00098	Up	PM20D1
SRSF12	11.2135523	0.68783489	0.17340311	3.96668134	7.29E-05	0.000986	Up	SRSF12
KY	7.46196743	0.70257812	0.17711903	3.96670036	7.29E-05	0.000986	Up	KY
CD209	260.232019	0.59029247	0.14882438	3.96636938	7.30E-05	0.000986	Up	CD209
GNRHR	2.7276418	0.96105229	0.24237148	3.96520364	7.33E-05	0.000988	Up	GNRHR
CYP1B1	226.605133	0.75602818	0.19066016	3.96531799	7.33E-05	0.000988	Up	CYP1B1
FUT9	2.20962136	2.07032468	0.52209701	3.96540231	7.33E-05	0.000988	Up	FUT9
CTSW	103.785345	0.58770949	0.14822531	3.964974	7.34E-05	0.000988	Up	CTSW
HEATR9	2.97159067	0.86797983	0.21904108	3.962635	7.41E-05	0.000995	Up	HEATR9
AMZ1	16.8617247	0.62780037	0.15846179	3.96184059	7.44E-05	0.000998	Up	AMZ1
ITPKA	536.608826	0.50056516	0.12637594	3.96092123	7.47E-05	0.001001	Up	ITPKA
RYR1	35.6702666	0.55990945	0.14145292	3.95827418	7.55E-05	0.001009	Up	RYR1
C14orf180	4.26261032	1.6020861	0.40504655	3.95531349	7.64E-05	0.00102	Up	C14orf180
CMTM5	4.33045395	1.20180971	0.30386643	3.95505913	7.65E-05	0.00102	Up	CMTM5
SUMO4	4.31522065	0.75699194	0.19141651	3.95468465	7.66E-05	0.001021	Up	SUMO4

PPP1R1A	42.7115832	0.92054944	0.23286898	3.95307883	7.72E-05	0.001025	Up	PPP1R1A
ROPN1L	5.12606768	0.69211484	0.17508011	3.95313222	7.71E-05	0.001025	Up	ROPN1L
S100A8	194.137579	0.63725528	0.16121956	3.95271685	7.73E-05	0.001026	Up	S100A8
NBPF4	12.3240372	-0.8754523	0.22180546	-3.94693766	7.92E-05	0.001047	Down	NBPF4
UTS2B	4.61453831	0.7577652	0.19207402	3.94517291	7.97E-05	0.001054	Up	UTS2B
DPP6	26.5763179	0.96728764	0.24531311	3.9430736	8.04E-05	0.001062	Up	DPP6
AKR1B15	15.4751583	0.91579804	0.23246914	3.93943927	8.17E-05	0.001075	Up	AKR1B15
NPPC	4.0346262	0.86711862	0.22025088	3.93695873	8.25E-05	0.001083	Up	NPPC
FCRL6	16.457005	0.60789194	0.15441044	3.93685775	8.26E-05	0.001083	Up	FCRL6
FAM135B	11.9547258	1.05657154	0.26864508	3.93296437	8.39E-05	0.001096	Up	FAM135B
NPIPB15	121.205087	-0.77943721	0.19818251	-3.93292637	8.39E-05	0.001096	Down	NPIPB15
C1QL4	3.20936983	0.88681758	0.22551287	3.93244779	8.41E-05	0.001098	Up	C1QL4
DMBT1	12635.3835	0.93897082	0.23882667	3.93159955	8.44E-05	0.0011	Up	DMBT1
CR1L	2.81289206	0.64468725	0.16397938	3.93151424	8.44E-05	0.0011	Up	CR1L
INSM1	58.2120958	0.92267044	0.23474165	3.93057835	8.47E-05	0.001102	Up	INSM1
CD38	199.583773	0.58427243	0.14866905	3.93002065	8.49E-05	0.001103	Up	CD38
IGFL2	85.7513183	0.84586721	0.21528033	3.92914312	8.52E-05	0.001105	Up	IGFL2
RHBG	5.21087269	0.86722497	0.22117407	3.92100658	8.82E-05	0.001141	Up	RHBG
ERICH2	30.0332158	-0.61274576	0.15630813	-3.92011438	8.85E-05	0.001144	Down	ERICH2
PDCD1LG2	67.8677954	0.54679941	0.13950857	3.91946824	8.87E-05	0.001145	Up	PDCD1LG2
SLC2A5	215.755666	0.54698814	0.13980199	3.91259197	9.13E-05	0.001172	Up	SLC2A5
PI16	95.6285934	1.03381357	0.26439987	3.91003815	9.23E-05	0.001182	Up	PI16
TCEAL2	26.7959409	1.20253951	0.30762504	3.909108	9.26E-05	0.001186	Up	TCEAL2
GRM1	4.64789162	0.90180883	0.23084417	3.90656966	9.36E-05	0.001196	Up	GRM1
THEMIS	44.6412123	0.5604685	0.14348374	3.90614635	9.38E-05	0.001197	Up	THEMIS
HSD3B2	25.7807082	1.329682	0.3409051	3.9004462	9.60E-05	0.001224	Up	HSD3B2
PRRT4	9.62122524	0.79114731	0.20289469	3.8993003	9.65E-05	0.001229	Up	PRRT4
SP140	99.7964087	0.53723926	0.13779195	3.89891631	9.66E-05	0.00123	Up	SP140
CAPN11	15.3604393	0.50446557	0.12944661	3.89709368	9.74E-05	0.001237	Up	CAPN11
KCND3	202.217279	0.59268353	0.15210017	3.89666572	9.75E-05	0.001238	Up	KCND3
HRK	8.15788614	1.01462772	0.2606132	3.89323222	9.89E-05	0.001255	Up	HRK
NAIP	21.8275506	0.51043679	0.13124091	3.88931153	0.000101	0.001269	Up	NAIP
IL1B	805.440266	0.62712845	0.16132211	3.88743018	0.000101	0.001275	Up	IL1B
PVALB	2.11526874	0.82146748	0.21150775	3.88386477	0.000103	0.001291	Up	PVALB
CDKN2A	329.304071	0.66068153	0.17013594	3.88325681	0.000103	0.001292	Up	CDKN2A
KRT84	2.0237338	1.40582939	0.36246547	3.87851948	0.000105	0.001314	Up	KRT84
KLHL6	224.59337	0.51522523	0.13287112	3.8776313	0.000105	0.001317	Up	KLHL6
DSCAML1	29.1410393	0.76156438	0.19643694	3.87688978	0.000106	0.001319	Up	DSCAML1
SH2D1B	14.8113863	0.58819173	0.15174713	3.87613077	0.000106	0.001322	Up	SH2D1B
NTSR1	107.479617	0.88399641	0.22817645	3.87417897	0.000107	0.001332	Up	NTSR1
SLC6A16	16.5004038	0.57902226	0.1495471	3.8718388	0.000108	0.001339	Up	SLC6A16
NRSN1	3.99902011	1.12250164	0.28990726	3.87193354	0.000108	0.001339	Up	NRSN1
CDH19	35.233723	1.1268795	0.29114148	3.87055634	0.000109	0.001342	Up	CDH19
TFEC	118.930443	0.57187907	0.14792013	3.86613423	0.000111	0.00136	Up	TFEC
CFAP221	4.06434964	0.8751873	0.22639946	3.86567744	0.000111	0.001361	Up	CFAP221
NKX6-2	1.04250237	1.31615001	0.34078513	3.862111	0.000112	0.001378	Up	NKX6-2
RAB44	8.99969389	0.56829114	0.14716532	3.86158335	0.000113	0.00138	Up	RAB44
COLGALT2	83.0268096	0.80637114	0.20884849	3.86103418	0.000113	0.001382	Up	COLGALT2
OAS2	1411.62667	0.5320535	0.13786864	3.85913353	0.000114	0.001391	Up	OAS2
CXCL9	1206.38847	0.68016525	0.17632309	3.85749393	0.000115	0.001399	Up	CXCL9
CAV3	1.93076205	1.25876504	0.32637874	3.85676171	0.000115	0.001402	Up	CAV3
NTSR2	0.67894401	1.56335878	0.40573174	3.85318333	0.000117	0.001421	Up	NTSR2
FMO2	38.2797157	0.8221059	0.21339417	3.85252274	0.000117	0.001423	Up	FMO2
HAPLN2	6.80916044	0.78831516	0.20462286	3.85252728	0.000117	0.001423	Up	HAPLN2
ENTPD3	45.4032182	0.69596878	0.18073589	3.85075024	0.000118	0.001432	Up	ENTPD3
FBXO15	5.00845723	0.55241236	0.14346896	3.85039649	0.000118	0.001433	Up	FBXO15
EMX2	5.74217881	1.15620165	0.30036535	3.84931767	0.000118	0.001437	Up	EMX2
SLC26A3	14451.4323	1.00670422	0.26154002	3.8491403	0.000119	0.001437	Up	SLC26A3

IGFL4	80.6226219	-0.68964222	0.1791821	-3.84883444	0.000119	0.001438	Down	IGFL4
MTRNR2L5	0.73223491	1.62381197	0.42192322	3.84859586	0.000119	0.001438	Up	MTRNR2L5
PROX2	13.8287798	0.61237793	0.15921691	3.84618651	0.00012	0.001447	Up	PROX2
SLC35F1	24.772949	0.54523613	0.14175498	3.84632777	0.00012	0.001447	Up	SLC35F1
KRT16	60.0948973	0.93817745	0.24398286	3.84525972	0.00012	0.001451	Up	KRT16
RBP3	5.20709925	1.12546353	0.29271501	3.84491219	0.000121	0.001452	Up	RBP3
FGB	42.287696	1.15015053	0.29917238	3.84444093	0.000121	0.001453	Up	FGB
ANKRD63	0.76074714	1.41074127	0.36712111	3.84271356	0.000122	0.001462	Up	ANKRD63
TFPI2	118.951558	0.70808756	0.18434375	3.84112597	0.000122	0.001466	Up	TFPI2
CSF3R	330.046212	0.56558172	0.14723956	3.84123485	0.000122	0.001466	Up	CSF3R
PRH2	3.44201392	0.89398249	0.23279043	3.84028883	0.000123	0.00147	Up	PRH2
HS3ST4	8.41148306	1.23922244	0.32276209	3.83942994	0.000123	0.001474	Up	HS3ST4
NGB	4.89969053	1.4817504	0.38607288	3.83800694	0.000124	0.001479	Up	NGB
C2CD4B	290.551266	0.67796712	0.17681253	3.83438384	0.000126	0.001495	Up	C2CD4B
FBN3	4.33633245	0.95603541	0.24951889	3.83151521	0.000127	0.001511	Up	FBN3
NETO1	2.286716	0.99440205	0.25958195	3.83078275	0.000128	0.001512	Up	NETO1
TMPRSS11A	0.81341583	2.28616078	0.596749	3.83102573	0.000128	0.001512	Up	TMPRSS11A
TMEFF1	0.9011458	1.001498	0.26143349	3.83079459	0.000128	0.001512	Up	TMEFF1
DNAI1	1.82091678	0.97907002	0.25559942	3.83048606	0.000128	0.001513	Up	DNAI1
CLEC7A	192.033606	0.50241186	0.13133515	3.82541802	0.000131	0.001541	Up	CLEC7A
TAT	10.7066249	0.75270338	0.19680345	3.82464532	0.000131	0.001545	Up	TAT
FCAR	18.5350715	0.76842382	0.20099442	3.82311014	0.000132	0.00155	Up	FCAR
MSR1	565.172394	0.57345793	0.1500809	3.82099218	0.000133	0.00156	Up	MSR1
CADM3	92.1841796	0.974048	0.25498948	3.81995369	0.000133	0.001565	Up	CADM3
MATN4	6.2720103	0.57357727	0.15016876	3.81955132	0.000134	0.001566	Up	MATN4
MAGEC1	2.72856733	2.33208077	0.61062852	3.81914812	0.000134	0.001566	Up	MAGEC1
CAPN12	215.227191	-0.51449998	0.13471291	-3.81923283	0.000134	0.001566	Down	CAPN12
CD8A	258.818439	0.54023327	0.14147176	3.81866514	0.000134	0.001568	Up	CD8A
WDR38	2.31197236	0.89629282	0.23473413	3.8183319	0.000134	0.001569	Up	WDR38
LINGO1	193.858671	0.51357999	0.13451195	3.8180994	0.000134	0.001569	Up	LINGO1
RNF113B	1.56113855	1.5965689	0.4182338	3.8174076	0.000135	0.001572	Up	RNF113B
PLD4	60.9320396	0.51332307	0.1345363	3.81549866	0.000136	0.001581	Up	PLD4
TGM6	2.42448306	1.96926359	0.51671544	3.81111815	0.000138	0.001605	Up	TGM6
GALNTL6	32.6143322	0.85040845	0.22315725	3.81080354	0.000139	0.001605	Up	GALNTL6
CYP3A4	29.5908221	0.75129798	0.19716939	3.81041897	0.000139	0.001607	Up	CYP3A4
ESPNL	43.9082868	0.67900294	0.17831939	3.80779094	0.00014	0.001616	Up	ESPNL
LRRTM2	14.9278057	0.60107185	0.15785358	3.80778087	0.00014	0.001616	Up	LRRTM2
GRID2	1.51893689	1.46677456	0.38521369	3.80769064	0.00014	0.001616	Up	GRID2
NCR3	13.3605725	0.54907422	0.14426666	3.80596738	0.000141	0.001625	Up	NCR3
HPGDS	37.7187602	0.61857367	0.16268353	3.80231273	0.000143	0.001646	Up	HPGDS
PSCA	65.1151524	0.95107173	0.25032429	3.79935862	0.000145	0.001661	Up	PSCA
OR10A2	1.02719773	1.8844149	0.49615754	3.79801724	0.000146	0.001667	Up	OR10A2
SLC1A7	280.74694	0.74665399	0.19678946	3.79417676	0.000148	0.001687	Up	SLC1A7
SLC10A1	6.06639325	0.73604188	0.19407381	3.79258729	0.000149	0.001695	Up	SLC10A1
GPHA2	1.05315831	1.20336123	0.31740579	3.79123907	0.00015	0.001703	Up	GPHA2
PIK3CG	120.633282	0.52537439	0.1385945	3.79073047	0.00015	0.001705	Up	PIK3CG
RETNLB	643.700999	-0.88085278	0.23237918	-3.79058387	0.00015	0.001705	Down	RETNLB
COL6A6	11.9166198	0.81749349	0.21578682	3.78843108	0.000152	0.001717	Up	COL6A6
CCL28	1170.69944	0.5567511	0.14704468	3.78627165	0.000153	0.001728	Up	CCL28
VSTM2L	163.777864	0.80117269	0.21164207	3.78550777	0.000153	0.001732	Up	VSTM2L
GFI1B	15.9633752	0.76935475	0.20332583	3.78385149	0.000154	0.00174	Up	GFI1B
UST	113.867228	0.59264446	0.15669546	3.78214186	0.000155	0.001748	Up	UST
ZNF648	2.17539577	0.86820877	0.22956582	3.78196011	0.000156	0.001748	Up	ZNF648
ABCA12	74.1381806	0.95262002	0.25197461	3.780619	0.000156	0.001756	Up	ABCA12
IFNG	12.235574	0.73183183	0.19358956	3.78032695	0.000157	0.001757	Up	IFNG
MADCAM1	59.3306517	0.60102806	0.15914899	3.77651192	0.000159	0.00178	Up	MADCAM1
CAMK2B	17.6650471	0.7396575	0.1962526	3.76890549	0.000164	0.00183	Up	CAMK2B
TRIML2	0.79633663	1.59388732	0.42307192	3.76741455	0.000165	0.00184	Up	TRIML2

ASB2	218.513556	0.6170542	0.16380212	3.76707082	0.000165	0.001841	Up	ASB2
CEACAM20	3.18834806	1.00113808	0.26608537	3.7624694	0.000168	0.001868	Up	CEACAM20
CCK	9.76972338	1.2747633	0.33905862	3.75971363	0.00017	0.001877	Up	CCK
HYAL4	2.94453912	0.92040464	0.2449983	3.7567797	0.000172	0.001891	Up	HYAL4
CSAG1	23.236985	1.79284557	0.47720722	3.75695398	0.000172	0.001891	Up	CSAG1
CD36	360.869314	0.60771213	0.16184254	3.75495927	0.000173	0.001902	Up	CD36
LKAAEAR1	1.46198662	1.15688254	0.30815205	3.7542588	0.000174	0.001906	Up	LKAAEAR1
RTL1	1.17299496	1.76397746	0.4704006	3.7499473	0.000177	0.001935	Up	RTL1
STXBP5L	13.3966875	0.89893114	0.24006795	3.7444862	0.000181	0.001971	Up	STXBP5L
SMYD1	20.9846382	1.0543853	0.28232128	3.73469998	0.000188	0.002034	Up	SMYD1
ONECUT2	181.368296	0.6176604	0.16540775	3.73416841	0.000188	0.002037	Up	ONECUT2
CASQ2	101.585666	1.01023814	0.27055808	3.73390487	0.000189	0.002038	Up	CASQ2
FCER2	23.6831496	0.86756177	0.23240634	3.73295223	0.000189	0.002043	Up	FCER2
HOXC5	1.27332627	1.27135552	0.34075094	3.7310404	0.000191	0.002056	Up	HOXC5
TEX11	23.9765489	0.70948151	0.19020438	3.73010078	0.000191	0.002062	Up	TEX11
LRRTM3	1.10887142	1.54406986	0.41395702	3.73002456	0.000191	0.002062	Up	LRRTM3
CYP2C9	29.3747995	0.81885362	0.21962482	3.72842026	0.000193	0.002074	Up	CYP2C9
NLRP4	2.26716609	1.33722766	0.35877437	3.72721068	0.000194	0.002082	Up	NLRP4
KIR3DL2	1.86962914	1.01255405	0.27190412	3.72393785	0.000196	0.002105	Up	KIR3DL2
MAGEA6	109.899917	2.12390552	0.57075029	3.72125178	0.000198	0.002127	Up	MAGEA6
MS4A4A	254.68326	0.50440517	0.13571073	3.71676717	0.000202	0.002161	Up	MS4A4A
IL1A	92.5975441	0.66487752	0.17898377	3.71473643	0.000203	0.002175	Up	IL1A
DPYSL5	7.33624325	0.92381788	0.24875446	3.71377409	0.000204	0.002182	Up	DPYSL5
C9orf24	45.7195833	0.58845229	0.15868169	3.70838171	0.000209	0.002224	Up	C9orf24
SI	647.859931	0.90974366	0.24542995	3.7067344	0.00021	0.002236	Up	SI
KCNV2	4.45735903	0.72446877	0.19568834	3.70215604	0.000214	0.002271	Up	KCNV2
TPO	25.1179856	0.66437475	0.17950499	3.70114927	0.000215	0.002278	Up	TPO
GBP7	5.17067043	0.82131416	0.22204328	3.69889216	0.000217	0.00229	Up	GBP7
ZFR2	2.64226094	0.701944	0.189833	3.69769218	0.000218	0.002298	Up	ZFR2
AQP9	194.223587	0.70458619	0.19060238	3.69662843	0.000218	0.002304	Up	AQP9
ZAR1	2.37331363	1.01831629	0.27551993	3.69598048	0.000219	0.002308	Up	ZAR1
PPM1E	13.529226	0.62108108	0.16825687	3.69126724	0.000223	0.002344	Up	PPM1E
ERBB4	2.88605841	1.04988375	0.28448768	3.69043658	0.000224	0.00235	Up	ERBB4
ABCB11	11.2140182	0.93038547	0.25247948	3.68499438	0.000229	0.002391	Up	ABCB11
CHRNA2	1.30240431	1.36408879	0.37017308	3.68500272	0.000229	0.002391	Up	CHRNA2
SLITRK4	12.4315989	0.79917982	0.21710441	3.68108512	0.000232	0.002422	Up	SLITRK4
PMP2	5.87880491	1.20951518	0.32868855	3.67982147	0.000233	0.002429	Up	PMP2
S100G	1.39676437	1.62682473	0.4425373	3.67613021	0.000237	0.002454	Up	S100G
CEACAM3	33.261136	0.51082064	0.13896363	3.67593043	0.000237	0.002455	Up	CEACAM3
DNER	29.5424882	0.77589793	0.21131018	3.67184358	0.000241	0.002488	Up	DNER
SOD3	3760.1222	0.56387678	0.15360337	3.67099234	0.000242	0.002495	Up	SOD3
CD200R1	39.6829036	0.53167722	0.14494742	3.66806956	0.000244	0.002521	Up	CD200R1
ADRA1A	3.75780724	0.83183591	0.22689332	3.66619829	0.000246	0.002536	Up	ADRA1A
FGF4	3.21296955	0.85832783	0.23431407	3.66315101	0.000249	0.002558	Up	FGF4
STAC	26.2370366	0.65750391	0.17948679	3.66324402	0.000249	0.002558	Up	STAC
TDRP	94.1715428	0.51284278	0.14005055	3.66184062	0.00025	0.002566	Up	TDRP
CD1B	10.3092796	0.62768185	0.17147222	3.66054537	0.000252	0.002577	Up	CD1B
OR2D2	0.9842982	2.29475916	0.62706696	3.65951214	0.000253	0.00258	Up	OR2D2
GALNT14	30.9030733	0.71232807	0.19471829	3.65824945	0.000254	0.00259	Up	GALNT14
RPSAP58	440.099482	-0.51741005	0.14145527	-3.65776432	0.000254	0.002593	Down	RPSAP58
PROZ	12.0846104	-0.52721268	0.14415192	-3.65734062	0.000255	0.002594	Down	PROZ
GCNT3	4305.6041	0.60457916	0.16540543	3.65513486	0.000257	0.002614	Up	GCNT3
RAB27B	481.402891	0.55661213	0.15236596	3.65312658	0.000259	0.00263	Up	RAB27B
CHST8	2.90329401	0.7810537	0.21386598	3.65207084	0.00026	0.002636	Up	CHST8
FAM217A	3.6446671	0.83107851	0.22763309	3.65095651	0.000261	0.002643	Up	FAM217A
WNT16	8.11361522	0.75017001	0.20551725	3.65015589	0.000262	0.002648	Up	WNT16
LMO1	1.08726785	1.22086269	0.33462394	3.64846187	0.000264	0.002662	Up	LMO1
IL1RL2	86.9383001	-0.50222544	0.13768221	-3.64771497	0.000265	0.002666	Down	IL1RL2

NLRP7	8.23872959	0.70342582	0.19284622	3.64759975	0.000265	0.002666	Up	NLRP7
C19orf84	3.22836542	0.71044177	0.19495345	3.64416103	0.000268	0.002694	Up	C19orf84
LYPD1	21.7512934	0.50088473	0.13753188	3.64195359	0.000271	0.00271	Up	LYPD1
PROK2	23.5634028	0.80615353	0.22134386	3.64208667	0.00027	0.00271	Up	PROK2
CHST13	102.960344	0.66489834	0.18268879	3.63951367	0.000273	0.002729	Up	CHST13
SLAMF7	561.710111	0.53558397	0.14721356	3.63814292	0.000275	0.00274	Up	SLAMF7
ANKRD1	5.04594423	0.84217364	0.23171872	3.63446524	0.000279	0.002777	Up	ANKRD1
NUGGC	37.8058276	0.64531313	0.17775321	3.63038799	0.000283	0.002816	Up	NUGGC
CXCR1	47.4766405	0.69087842	0.19044658	3.62767565	0.000286	0.002839	Up	CXCR1
GCGR	4.70040981	0.7014252	0.19337783	3.62722648	0.000286	0.002842	Up	GCGR
F2	22.7852647	0.83365722	0.22985445	3.62689181	0.000287	0.002844	Up	F2
DLX2	6.79427562	0.772556	0.21304321	3.62628774	0.000288	0.002845	Up	DLX2
TAS2R60	1.07935027	1.42343202	0.39253591	3.62624661	0.000288	0.002845	Up	TAS2R60
CCL3L3	84.7102879	0.62133766	0.17134468	3.6262443	0.000288	0.002845	Up	CCL3L3
DIO3	183.750704	0.62950363	0.17362854	3.62557702	0.000288	0.002851	Up	DIO3
MAGEA10	2.02543867	1.67432013	0.46183942	3.62532958	0.000289	0.002852	Up	MAGEA10
P2RY10	42.2721378	0.52936812	0.14609388	3.62347915	0.000291	0.002869	Up	P2RY10
CST2	63.5061722	0.74903191	0.20698775	3.61872573	0.000296	0.002915	Up	CST2
POU5F1B	288.035543	-0.60931277	0.16837161	-3.61885703	0.000296	0.002915	Down	POU5F1B
LDHAL6A	5.5594165	0.61817452	0.17094773	3.61616102	0.000299	0.002939	Up	LDHAL6A
FCGR2B	133.703474	0.5418537	0.1498714	3.61545768	0.0003	0.002946	Up	FCGR2B
P2RY13	93.9989147	0.5176265	0.1431835	3.61512667	0.0003	0.002948	Up	P2RY13
HDAC9	181.96245	0.50602008	0.13998362	3.614852	0.000301	0.002949	Up	HDAC9
BTBD17	1.62269563	0.95643097	0.26490248	3.61050221	0.000306	0.00299	Up	BTBD17
TCL1A	30.6968475	1.00179962	0.27765984	3.60801049	0.000309	0.003012	Up	TCL1A
KCNH3	20.5013885	0.55090638	0.15274305	3.60675264	0.00031	0.003022	Up	KCNH3
LMO3	137.756233	0.74871571	0.20765988	3.60549039	0.000312	0.00303	Up	LMO3
MTNR1A	14.8787399	0.64684501	0.17954265	3.60273734	0.000315	0.003057	Up	MTNR1A
USH2A	11.9335072	0.59641742	0.16560847	3.60137033	0.000317	0.003068	Up	USH2A
SPRR1A	16.7334457	1.31951655	0.36655018	3.59982509	0.000318	0.003083	Up	SPRR1A
CTAGE8	4.30543864	-0.75223567	0.20909163	-3.59763645	0.000321	0.003102	Down	CTAGE8
PLIN1	57.9059257	0.70055601	0.19476016	3.59701912	0.000322	0.003104	Up	PLIN1
CCDC175	13.7926494	-0.59743337	0.16611751	-3.59645026	0.000323	0.003107	Down	CCDC175
G6PC2	1.42198746	1.07985173	0.30050435	3.59346457	0.000326	0.003134	Up	G6PC2
GRIN2A	10.3169974	0.8704776	0.24223134	3.59357961	0.000326	0.003134	Up	GRIN2A
EDN2	45.866106	0.61757053	0.17187817	3.59307137	0.000327	0.003137	Up	EDN2
MPPED1	1.32904254	1.06914155	0.29774196	3.59083263	0.00033	0.003156	Up	MPPED1
CHGB	115.74878	0.81366551	0.22661364	3.5905408	0.00033	0.003157	Up	CHGB
FBXO40	0.89889615	1.4854927	0.41375302	3.59028849	0.00033	0.003158	Up	FBXO40
CCR2	82.2346185	0.55925719	0.15588207	3.58769419	0.000334	0.003181	Up	CCR2
SYT5	15.0301308	0.61759567	0.1721735	3.58705422	0.000334	0.003187	Up	SYT5
NXPH1	3.96504629	-0.90676	0.25280203	-3.58683833	0.000335	0.003188	Down	NXPH1
EFCAB1	4.44907393	0.60139626	0.16769521	3.58624597	0.000335	0.003192	Up	EFCAB1
GDAP1L1	8.53088321	0.69533305	0.19396433	3.58485009	0.000337	0.003205	Up	GDAP1L1
LHCGR	1.12938236	1.96568306	0.54905897	3.58009458	0.000343	0.003252	Up	LHCGR
TFAP2B	0.7972001	1.80909622	0.50560472	3.57808414	0.000346	0.003269	Up	TFAP2B
SLC1A2	13.2291116	0.56486691	0.15786735	3.57811105	0.000346	0.003269	Up	SLC1A2
NPAS3	18.4035376	0.63687601	0.17805697	3.57681026	0.000348	0.003282	Up	NPAS3
UGT3A2	3.46084356	0.82176087	0.22980715	3.5758717	0.000349	0.003292	Up	UGT3A2
SPTBN4	25.5608099	0.55715458	0.15585631	3.5747965	0.000351	0.003303	Up	SPTBN4
A2ML1	9.00256619	0.87368682	0.24462075	3.57159737	0.000355	0.003337	Up	A2ML1
ACSM5	9.48042193	0.68905163	0.19294421	3.57124812	0.000355	0.003339	Up	ACSM5
SPDYE1	9.61783046	0.51132425	0.14321042	3.57044016	0.000356	0.003348	Up	SPDYE1
FASLG	28.7809456	0.51460164	0.14420988	3.5684216	0.000359	0.003365	Up	FASLG
NEUROG3	28.4775119	0.9894625	0.27769096	3.56317862	0.000366	0.003416	Up	NEUROG3
CCDC42	1.04170156	0.92203128	0.25881064	3.56257094	0.000367	0.00342	Up	CCDC42
TMEM37	806.297225	0.52319758	0.1468594	3.56257466	0.000367	0.00342	Up	TMEM37
SLC12A3	4.45469286	0.66152585	0.18580961	3.56023491	0.000371	0.003441	Up	SLC12A3

SYN3	104.405461	-0.74776587	0.21004303	-3.56006034	0.000371	0.003442	Down	SYN3
CCDC38	2.83484904	0.7149043	0.20092877	3.55799866	0.000374	0.003465	Up	CCDC38
AMPD1	22.8368713	0.82566543	0.23207101	3.55781382	0.000374	0.003466	Up	AMPD1
PCDH15	2.06984279	1.10239062	0.31003174	3.55573478	0.000377	0.003489	Up	PCDH15
ATP13A5	1.17875651	1.16310146	0.32719615	3.55475286	0.000378	0.003501	Up	ATP13A5
CNR2	13.2933095	0.67342742	0.1896189	3.55147832	0.000383	0.003541	Up	CNR2
MYPN	17.2368657	0.69273864	0.19511654	3.55038407	0.000385	0.003551	Up	MYPN
RGS7	6.2599953	0.89350567	0.25194753	3.54639584	0.000391	0.003586	Up	RGS7
HTR7	10.8574576	0.58195927	0.16412354	3.54586096	0.000391	0.003592	Up	HTR7
GFY	1.54551679	1.25903074	0.35522476	3.54432144	0.000394	0.003611	Up	GFY
NTF4	1.820285	0.98145519	0.27698033	3.54341114	0.000395	0.003621	Up	NTF4
VSTM2A	22.5172548	1.13898809	0.32152567	3.54244839	0.000396	0.00363	Up	VSTM2A
MRC1	592.752222	0.54814723	0.15474199	3.54233034	0.000397	0.00363	Up	MRC1
TENM1	13.2505488	0.77438997	0.21862621	3.54207283	0.000397	0.003632	Up	TENM1
HOXC12	7.18910317	1.68691504	0.47627924	3.54186137	0.000397	0.003633	Up	HOXC12
LCE1E	1.96585899	1.60379468	0.45292868	3.5409431	0.000399	0.003642	Up	LCE1E
P2RY12	13.2215462	0.68597576	0.19387709	3.53819913	0.000403	0.003672	Up	P2RY12
SPATA31E1	0.78003705	1.2537567	0.35437898	3.53789803	0.000403	0.003674	Up	SPATA31E1
SLC26A9	30.1285353	1.01894599	0.28859133	3.53075749	0.000414	0.003753	Up	SLC26A9
ORM2	11.9983515	0.74025611	0.20971033	3.52989822	0.000416	0.003763	Up	ORM2
ODF3	1.01564871	1.66542456	0.47182764	3.5297308	0.000416	0.003764	Up	ODF3
ANKS1B	13.3808348	0.72092315	0.20425464	3.52953135	0.000416	0.003764	Up	ANKS1B
ATP6V0D2	49.2576718	0.51933614	0.1472514	3.5268672	0.000421	0.003795	Up	ATP6V0D2
LRCOL1	2.01933527	0.75677273	0.21466493	3.52536739	0.000423	0.003812	Up	LRCOL1
SCHIP1	18.0210094	0.53468787	0.15174934	3.52349379	0.000426	0.003833	Up	SCHIP1
TSGA10IP	1.09747265	0.70365854	0.19979349	3.52192925	0.000428	0.003852	Up	TSGA10IP
DYNLRB2	2.89943775	-0.6052177	0.17194131	-3.51990864	0.000432	0.003879	Down	DYNLRB2
GABRA4	31.9280411	0.88930175	0.25266597	3.51967363	0.000432	0.003881	Up	GABRA4
TRNP1	295.968627	0.56854947	0.16156586	3.51899516	0.000433	0.003884	Up	TRNP1
SUSD4	18.7879308	0.58162201	0.16543884	3.51563148	0.000439	0.003925	Up	SUSD4
COL6A5	9.12099472	0.86552132	0.24621202	3.51534952	0.000439	0.003926	Up	COL6A5
CYP1A1	9.42666288	1.21795803	0.34649047	3.51512708	0.00044	0.003927	Up	CYP1A1
NTS	48.6497447	0.99528269	0.28417465	3.50236267	0.000461	0.004086	Up	NTS
C7	601.953154	0.80222249	0.22924601	3.4993956	0.000466	0.004123	Up	C7
DSPP	0.8821008	1.98454931	0.56729847	3.49824549	0.000468	0.004131	Up	DSPP
MTRNR2L1	345.238226	-1.11397768	0.31841999	-3.49845395	0.000468	0.004131	Down	MTRNR2L1
FAM163B	12.0075035	0.75869313	0.21707452	3.49508144	0.000474	0.004173	Up	FAM163B
CST11	0.62181841	-1.09036533	0.31200263	-3.49473119	0.000475	0.004177	Down	CST11
RRAD	120.203469	0.50751945	0.14523415	3.49449123	0.000475	0.004178	Up	RRAD
FAM189A2	43.6817888	0.56852616	0.16304575	3.48691185	0.000489	0.004272	Up	FAM189A2
CHRNA4	1.16458125	1.22409121	0.35110448	3.48640161	0.00049	0.004278	Up	CHRNA4
CEACAM4	11.7521175	0.50801251	0.14574655	3.48558867	0.000491	0.004285	Up	CEACAM4
SLC8A3	7.42196522	0.59009686	0.16932229	3.4850512	0.000492	0.004291	Up	SLC8A3
UNC13C	2.0351323	0.97874835	0.28093792	3.48385983	0.000494	0.004301	Up	UNC13C
BPIFB2	4.67801378	1.54825417	0.44445043	3.48352497	0.000495	0.004305	Up	BPIFB2
ELAVL4	18.5849807	0.67450063	0.19367951	3.48256054	0.000497	0.004311	Up	ELAVL4
HNRNPCL1	1.12018484	0.72192771	0.20729609	3.48259203	0.000497	0.004311	Up	HNRNPCL1
TSHZ2	223.478758	0.54546274	0.15680203	3.47867149	0.000504	0.00437	Up	TSHZ2
CTSE	1865.18	0.87061516	0.25046967	3.47593042	0.000509	0.004402	Up	CTSE
DKK1	57.8500869	0.83049181	0.23903993	3.47428074	0.000512	0.00442	Up	DKK1
BEND4	8.66064688	0.67894904	0.19548104	3.47322188	0.000514	0.004431	Up	BEND4
TREM1	147.40965	0.59464224	0.17125259	3.47231088	0.000516	0.004441	Up	TREM1
ASB15	0.77861822	1.52645209	0.43986387	3.47028296	0.00052	0.004466	Up	ASB15
CT83	6.91819897	2.50445303	0.72203921	3.46858313	0.000523	0.004488	Up	CT83
GCSAML	10.3465406	0.63951688	0.1843876	3.46832906	0.000524	0.004488	Up	GCSAML
RIMS4	11.3786782	0.96321158	0.2778486	3.46667778	0.000527	0.004508	Up	RIMS4
NDP	27.288219	0.69253633	0.1997794	3.46650518	0.000527	0.004509	Up	NDP
DRGX	16.3445667	1.06970267	0.30905411	3.46121481	0.000538	0.004587	Up	DRGX

SPSB4	9.56645447	0.59899823	0.17308506	3.46071587	0.000539	0.004593	Up	SPSB4
NRG1	98.9041516	0.5194469	0.15036675	3.45453305	0.000551	0.004688	Up	NRG1
PRODH2	1.09584234	1.766132	0.51136825	3.45373807	0.000553	0.0047	Up	PRODH2
NTN3	2.56320114	0.65507111	0.18974136	3.45244239	0.000556	0.004713	Up	NTN3
XCL2	8.14071082	0.60467691	0.17517195	3.45190484	0.000557	0.004718	Up	XCL2
DGKB	20.8154509	0.8079577	0.23409079	3.45147153	0.000558	0.004723	Up	DGKB
MGAM	44.5874057	0.56744541	0.16461279	3.44715266	0.000567	0.004787	Up	MGAM
FRRS1L	12.088302	0.83244127	0.24161024	3.44538905	0.00057	0.004809	Up	FRRS1L
IRX6	1.81764803	1.18182276	0.34314765	3.44406487	0.000573	0.004823	Up	IRX6
CYTL1	19.8848558	0.52268255	0.15180545	3.44310806	0.000575	0.004838	Up	CYTL1
PI3	3281.62654	0.65582846	0.1905102	3.44248481	0.000576	0.004847	Up	PI3
BPIFB4	0.8483133	1.85820332	0.54024626	3.43954869	0.000583	0.00489	Up	BPIFB4
FGF20	33.2516902	-1.16943788	0.34028921	-3.43659991	0.000589	0.004936	Down	FGF20
ADAMTS20	2.73317917	1.97294575	0.57492001	3.43168739	0.0006	0.00501	Up	ADAMTS20
LIX1	5.20931773	0.96233467	0.28047053	3.43114361	0.000601	0.005017	Up	LIX1
BAALC	23.8059973	0.53923881	0.15731354	3.42779647	0.000609	0.005062	Up	BAALC
ENHO	23.7954747	0.56299791	0.16426734	3.42732712	0.00061	0.005066	Up	ENHO
HEPACAM	2.09573387	1.12322292	0.32782559	3.42628197	0.000612	0.005078	Up	HEPACAM
BPIFA2	0.64023099	1.71587263	0.5013542	3.42247583	0.000621	0.005135	Up	BPIFA2
AMBN	0.91880434	1.45965402	0.42653982	3.42208149	0.000621	0.00514	Up	AMBN
PRR18	3.84878878	0.9154735	0.26764732	3.42044717	0.000625	0.005168	Up	PRR18
GALR3	1.54589531	0.85203489	0.24925638	3.41830727	0.00063	0.005195	Up	GALR3
C3orf49	7.57829093	0.51303986	0.15044108	3.41023778	0.000649	0.005325	Up	C3orf49
PCDHA9	1.34439162	1.06174037	0.31138035	3.40978602	0.00065	0.005331	Up	PCDHA9
NELL1	13.2404585	0.67827789	0.19898373	3.40871023	0.000653	0.005347	Up	NELL1
UTS2R	6.73895076	0.87924147	0.25809576	3.40664829	0.000658	0.005382	Up	UTS2R
KIR2DS4	2.02047902	1.03658499	0.3043448	3.40595597	0.000659	0.005393	Up	KIR2DS4
TEKT2	3.93046067	0.62585996	0.18379559	3.40519567	0.000661	0.005402	Up	TEKT2
CXCR2	67.1698625	0.57004028	0.16756452	3.40191518	0.000669	0.00544	Up	CXCR2
BTNL2	0.43521808	1.16132205	0.34142831	3.40136426	0.000671	0.005449	Up	BTNL2
LIPN	2.45873323	0.91791388	0.27007605	3.3987237	0.000677	0.005499	Up	LIPN
LDHD	546.424937	0.54577914	0.16072154	3.39580582	0.000684	0.005539	Up	LDHD
MUC19	2.15902187	1.10100269	0.32428472	3.39517285	0.000686	0.005547	Up	MUC19
LHX9	2.51329706	1.1896217	0.35043675	3.39468304	0.000687	0.005549	Up	LHX9
GUCY1A2	106.933272	0.5169887	0.1523972	3.3923767	0.000693	0.005585	Up	GUCY1A2
KCNK15	54.7139726	0.6627902	0.19540761	3.39183417	0.000694	0.005594	Up	KCNK15
DDX25	2.72963605	0.7744071	0.22836812	3.39104724	0.000696	0.005602	Up	DDX25
C2CD4A	868.779856	0.54779475	0.16155199	3.39082627	0.000697	0.005604	Up	C2CD4A
CLEC9A	8.41348384	0.61007741	0.17994754	3.39030702	0.000698	0.005612	Up	CLEC9A
ANKRD7	1.08234055	0.87003123	0.25687416	3.38699389	0.000707	0.005664	Up	ANKRD7
GPR151	0.75326881	1.5336004	0.45338309	3.38257079	0.000718	0.005729	Up	GPR151
TRPM6	529.369946	0.70496017	0.20846384	3.38169036	0.00072	0.005735	Up	TRPM6
KCNS2	4.57502538	0.75613462	0.22360208	3.38160821	0.000721	0.005735	Up	KCNS2
ATCAY	7.90169575	0.76559578	0.22637677	3.38195374	0.00072	0.005735	Up	ATCAY
RPS4Y2	0.59675281	1.16622611	0.34502661	3.38010487	0.000725	0.005752	Up	RPS4Y2
ZBTB9	1.16698005	-0.51922073	0.15366253	-3.37896768	0.000728	0.005765	Down	ZBTB9
ARPP21	2.54192606	1.1405968	0.33767016	3.377843	0.000731	0.005786	Up	ARPP21
EBF2	24.7665869	0.57295207	0.16973522	3.37556374	0.000737	0.005818	Up	EBF2
CLDN20	11.1075549	0.60885515	0.18041955	3.37466281	0.000739	0.005834	Up	CLDN20
POLR2F	3.86000017	0.53415537	0.15831077	3.37409376	0.000741	0.005841	Up	POLR2F
HP	17.4420858	0.92456831	0.27406614	3.37352255	0.000742	0.005847	Up	HP
HOXC13	3.81496903	1.5101341	0.44767691	3.3732678	0.000743	0.005848	Up	HOXC13
GZMH	58.6943655	0.51658149	0.15315924	3.37283913	0.000744	0.005852	Up	GZMH
SPRR2A	17.048716	1.13533261	0.33712012	3.36773913	0.000758	0.005936	Up	SPRR2A
NOL4	13.0227896	0.8672387	0.25764333	3.36604372	0.000763	0.005967	Up	NOL4
PPP1R17	0.64254096	1.461775	0.43438108	3.36519033	0.000765	0.005983	Up	PPP1R17
SLC35G6	1.72558975	0.7927016	0.2355816	3.36487062	0.000766	0.005985	Up	SLC35G6
KCNA1	4.3847066	1.14605977	0.34064746	3.36435735	0.000767	0.005993	Up	KCNA1

ACTL8	49.7181686	0.67203676	0.19988297	3.3621511	0.000773	0.006022	Up	ACTL8
DMRT3	7.98848172	0.86468358	0.25737797	3.35958655	0.000781	0.006073	Up	DMRT3
GALNT8	373.465196	-0.6245983	0.18593919	-3.35915358	0.000782	0.006079	Down	GALNT8
GALNT9	34.9783216	0.74649183	0.22223359	3.35904147	0.000782	0.006079	Up	GALNT9
TP63	15.0789575	0.60290484	0.17955444	3.35778288	0.000786	0.006099	Up	TP63
DNAH8	7.39308602	0.71462059	0.21318153	3.35216938	0.000802	0.006201	Up	DNAH8
GPR21	0.50300117	1.26581612	0.37785602	3.34999588	0.000808	0.006242	Up	GPR21
ZSCAN10	1.47729936	0.92915361	0.27753408	3.34789013	0.000814	0.00628	Up	ZSCAN10
ADD2	29.3980346	0.63871221	0.19084636	3.34673509	0.000818	0.006299	Up	ADD2
PRSS58	0.64659067	1.29174664	0.38598817	3.34659646	0.000818	0.006299	Up	PRSS58
C1orf94	1.24616309	1.11695532	0.33401417	3.34403571	0.000826	0.006346	Up	C1orf94
C4orf51	0.64035416	1.14333041	0.34205454	3.34253833	0.00083	0.006366	Up	C4orf51
ZNF705A	0.95566673	0.97368128	0.2915099	3.34013107	0.000837	0.006404	Up	ZNF705A
MRGPRE	1.41327948	0.95095162	0.28483656	3.3385869	0.000842	0.006432	Up	MRGPRE
RAB40AL	2.11617017	0.90786853	0.27205461	3.33708198	0.000847	0.00646	Up	RAB40AL
PTPRQ	1.22657882	1.3712614	0.41101509	3.33627995	0.000849	0.006473	Up	PTPRQ
TEKT1	1.5025146	1.01175563	0.30328133	3.33603004	0.00085	0.006476	Up	TEKT1
LHX1	2.80723729	1.34081075	0.4020467	3.33496271	0.000853	0.006498	Up	LHX1
PKD2L2	4.69321608	0.69158151	0.20738914	3.33470461	0.000854	0.006501	Up	PKD2L2
TSHR	8.78646225	0.64588414	0.19376126	3.33340187	0.000858	0.006515	Up	TSHR
HS6ST3	6.4623891	0.86736596	0.26018677	3.33362823	0.000857	0.006515	Up	HS6ST3
OR2AG2	1.84686538	0.69794613	0.2093751	3.33347243	0.000858	0.006515	Up	OR2AG2
LILRB5	135.754181	0.51000988	0.15307803	3.33169872	0.000863	0.006546	Up	LILRB5
IL1R2	466.400916	0.5509702	0.16538377	3.33146469	0.000864	0.006549	Up	IL1R2
HCN4	2.92049789	0.68977282	0.20711542	3.33037893	0.000867	0.006568	Up	HCN4
CHRM5	5.865962	0.5705941	0.17139195	3.32917684	0.000871	0.006585	Up	CHRM5
NXF3	67.2061691	0.90137461	0.27083762	3.32809977	0.000874	0.006605	Up	NXF3
KCNJ4	4.84778384	0.76840947	0.23109532	3.32507587	0.000884	0.006671	Up	KCNJ4
TRAPPC3L	1.61254083	0.61768906	0.18582249	3.32408133	0.000887	0.006687	Up	TRAPPC3L
OSTN	0.67706426	1.62762533	0.48990057	3.32235853	0.000893	0.006714	Up	OSTN
SSTR3	6.97664439	0.62042534	0.18675938	3.32205712	0.000894	0.006717	Up	SSTR3
CR1	74.9411818	0.57983381	0.17460079	3.32091178	0.000897	0.006742	Up	CR1
XIRP2	0.76141682	1.65636002	0.49890519	3.31998959	0.0009	0.006758	Up	XIRP2
GRM5	0.59155156	1.25648091	0.37904687	3.31484312	0.000917	0.006863	Up	GRM5
MORN5	10.500008	1.20059609	0.36236181	3.31325225	0.000922	0.006887	Up	MORN5
ALLC	1.15336488	0.99931809	0.30163571	3.31299659	0.000923	0.00689	Up	ALLC
CLEC6A	4.61063395	0.73350034	0.22155393	3.31070795	0.000931	0.006932	Up	CLEC6A
RNF148	3.80100149	0.55194854	0.16678452	3.30935108	0.000935	0.006956	Up	RNF148
ALK	5.75082412	0.7966053	0.24076711	3.30861343	0.000938	0.006971	Up	ALK
MS4A2	49.7008161	0.56631216	0.17133036	3.30538131	0.000948	0.00703	Up	MS4A2
CRISP3	1.14379398	1.72323452	0.52201101	3.30114596	0.000963	0.007098	Up	CRISP3
CYP2A6	2.49917861	0.74186337	0.224765	3.30061785	0.000965	0.007106	Up	CYP2A6
RNF175	10.9135455	0.52693598	0.15966011	3.30036081	0.000966	0.007109	Up	RNF175
PIWIL1	245.181552	0.80463079	0.24392162	3.29872684	0.000971	0.007141	Up	PIWIL1
CYP7B1	42.108888	0.50288531	0.1524475	3.29874422	0.000971	0.007141	Up	CYP7B1
FANCD2OS	2.57645405	0.60029941	0.18218755	3.29495305	0.000984	0.007215	Up	FANCD2OS
LGALS7	1.00100474	1.3352346	0.40530314	3.29440974	0.000986	0.007221	Up	LGALS7
CDHR4	2.60313448	0.76852875	0.23331448	3.29396077	0.000988	0.007229	Up	CDHR4
MMP23B	7.30534968	0.5009684	0.15214281	3.292751	0.000992	0.007251	Up	MMP23B
IL19	2.9974019	0.83904693	0.25483864	3.29246352	0.000993	0.007256	Up	IL19
TBPL2	1.38710057	0.80488358	0.24450378	3.29190651	0.000995	0.007264	Up	TBPL2
CRB1	1.82012606	0.85126234	0.25864863	3.29119213	0.000998	0.007279	Up	CRB1
DCDC2B	7.33773179	0.55878842	0.1699109	3.28871434	0.001006	0.007331	Up	DCDC2B
ATP4B	1.3887176	0.77779637	0.2366666	3.28646451	0.001015	0.007368	Up	ATP4B
DUSP4	1605.36749	0.57258574	0.17431664	3.28474515	0.001021	0.007405	Up	DUSP4
CNNM1	23.9728	0.55564511	0.16920234	3.28390914	0.001024	0.007415	Up	CNNM1
MICU3	28.7017663	0.52154366	0.15882061	3.28385389	0.001024	0.007415	Up	MICU3
DSC3	495.298412	-0.78680842	0.23970672	-3.28237952	0.001029	0.007447	Down	DSC3

SCGB3A2	1.61190564	1.1002506	0.33526565	3.28172785	0.001032	0.007458	Up	SCGB3A2
TMOD1	78.0500075	0.57676648	0.17586366	3.27962281	0.001039	0.007505	Up	TMOD1
NCR2	1.30966432	0.95741294	0.29210255	3.27766033	0.001047	0.007541	Up	NCR2
TMEM132D	6.98623149	0.65461461	0.19973719	3.27737974	0.001048	0.007545	Up	TMEM132D
THRSP	5.08872285	0.7681776	0.23464689	3.27375997	0.001061	0.007632	Up	THRSP
SLITRK2	4.65734222	0.81208297	0.24821935	3.27163441	0.001069	0.007675	Up	SLITRK2
LGI1	7.37336596	1.19562702	0.36561357	3.2701932	0.001075	0.007689	Up	LGI1
CELF5	55.737385	-0.53775524	0.16445946	-3.26983464	0.001076	0.007691	Down	CELF5
PATE4	0.59049915	2.0272678	0.62031294	3.26813723	0.001083	0.007712	Up	PATE4
CLPSL2	1.93878598	0.86055086	0.26343414	3.2666641	0.001088	0.007743	Up	CLPSL2
PAQR9	0.68599898	1.66323309	0.50961827	3.26368415	0.0011	0.007795	Up	PAQR9
ATP1A4	2.11068402	0.61967352	0.18988299	3.26344938	0.001101	0.007799	Up	ATP1A4
CYP7A1	1.36343013	1.1640559	0.35692136	3.26137916	0.001109	0.007833	Up	CYP7A1
SBK3	2.77176035	0.55471185	0.17009091	3.26126681	0.001109	0.007833	Up	SBK3
CDK15	7.80743067	0.56760071	0.17408751	3.26043328	0.001112	0.00785	Up	CDK15
SERPINA9	4.35176592	1.21931594	0.37404052	3.25984989	0.001115	0.007853	Up	SERPINA9
F11	5.75657667	0.88890947	0.27277097	3.25881255	0.001119	0.007869	Up	F11
CASP14	0.78819591	1.51235349	0.46412766	3.25848602	0.00112	0.007871	Up	CASP14
NKAIN4	16.9343383	0.54027203	0.16582065	3.25817091	0.001121	0.007877	Up	NKAIN4
RETN	6.65069508	0.68575886	0.2105619	3.25680403	0.001127	0.007902	Up	RETN
RGS20	7.62923835	0.63206576	0.19408188	3.25669642	0.001127	0.007902	Up	RGS20
ANKRD55	4.52810149	0.51293006	0.15749195	3.25686537	0.001126	0.007902	Up	ANKRD55
CATSPERD	0.85634781	1.09682973	0.33694595	3.25520973	0.001133	0.007938	Up	CATSPERD
LRRC18	3.91595059	0.57389859	0.17634611	3.25438748	0.001136	0.007947	Up	LRRC18
SYNPO2	2604.66207	0.68031171	0.20913975	3.25290479	0.001142	0.007975	Up	SYNPO2
FGFBP2	10.1535274	0.62205424	0.19128707	3.25194092	0.001146	0.007999	Up	FGFBP2
SPESP1	26.1307996	0.66356332	0.2041483	3.25039848	0.001152	0.008031	Up	SPESP1
ECEL1	30.2420926	0.66200419	0.20376148	3.24891732	0.001158	0.008062	Up	ECEL1
CDH10	1.72212539	1.21859352	0.37513728	3.24839354	0.001161	0.008073	Up	CDH10
ST8SIA3	3.58423557	1.02554348	0.3157803	3.24764872	0.001164	0.008088	Up	ST8SIA3
COL26A1	7.2067362	0.55562229	0.17112331	3.24691181	0.001167	0.008102	Up	COL26A1
FRMPD4	4.70745017	1.06051536	0.32669824	3.24616184	0.00117	0.008117	Up	FRMPD4
OVCH1	2.26520236	0.86794433	0.26766113	3.24269843	0.001184	0.008213	Up	OVCH1
GJB6	8.12911816	0.78918193	0.24340303	3.24228474	0.001186	0.008221	Up	GJB6
GLT1D1	16.7318844	0.52121317	0.16076512	3.24207876	0.001187	0.008221	Up	GLT1D1
SYT4	11.8018814	0.96805744	0.29868309	3.24108549	0.001191	0.008234	Up	SYT4
SEMA6D	357.773478	0.50019477	0.15440347	3.23953062	0.001197	0.008268	Up	SEMA6D
KCNJ1	1.27981457	0.74349652	0.22981113	3.23525023	0.001215	0.008376	Up	KCNJ1
TLR10	35.0046504	0.56541213	0.1750406	3.230177	0.001237	0.008492	Up	TLR10
PGC	56.1266846	0.71234711	0.22073017	3.22723038	0.00125	0.008549	Up	PGC
MAGEB2	4.39034603	1.93129955	0.59881405	3.22520747	0.001259	0.008589	Up	MAGEB2
SLC16A12	5.02530741	0.62133209	0.19266067	3.2250074	0.00126	0.008589	Up	SLC16A12
C2orf66	6.70470372	-0.60792315	0.18852483	-3.22463175	0.001261	0.00859	Down	C2orf66
TAS2R38	11.3566937	-0.51592482	0.15999236	-3.22468402	0.001261	0.00859	Down	TAS2R38
SH2D7	28.5200412	0.65334287	0.20277892	3.22194675	0.001273	0.008657	Up	SH2D7
PSD	296.988259	0.55021883	0.17086471	3.22020178	0.001281	0.008703	Up	PSD
WNT1	1.65989476	0.69861081	0.21724449	3.21578146	0.001301	0.008803	Up	WNT1
LRAT	7.17285936	0.739534	0.23004908	3.21467922	0.001306	0.008826	Up	LRAT
IL36B	3.83632728	1.20624245	0.37530136	3.21406365	0.001309	0.008838	Up	IL36B
KRT37	2.80553191	-1.15521055	0.35950171	-3.21336593	0.001312	0.008849	Down	KRT37
ZNF80	2.96213142	0.63353716	0.19725254	3.21180728	0.001319	0.008891	Up	ZNF80
GSG1L	3.59971643	0.82431298	0.25670109	3.2111783	0.001322	0.008903	Up	GSG1L
SYT9	2.29181016	0.87016447	0.27118615	3.20873493	0.001333	0.008972	Up	SYT9
MORC1	0.88967037	1.27818741	0.39845824	3.20783283	0.001337	0.008988	Up	MORC1
GRIK1	9.5105337	0.57782663	0.18033888	3.2041157	0.001355	0.009096	Up	GRIK1
OR6A2	0.83539438	1.69298236	0.52844503	3.20370573	0.001357	0.009105	Up	OR6A2
NEFM	8.45312553	0.73333088	0.2291156	3.20070258	0.001371	0.009188	Up	NEFM
APOBEC2	5.1800304	0.57692766	0.18030183	3.19978811	0.001375	0.009201	Up	APOBEC2

PCDHA8	1.16743772	1.10228633	0.34448665	3.19979404	0.001375	0.009201	Up	PCDHA8
CD22	129.431621	0.60058918	0.18776172	3.19867748	0.001381	0.009226	Up	CD22
FCGR2C	39.4618617	0.50308447	0.15731223	3.19799983	0.001384	0.00924	Up	FCGR2C
MS4A3	1.0091775	0.90155745	0.28197474	3.19729866	0.001387	0.009256	Up	MS4A3
TDRD9	13.0141061	0.5394139	0.16889953	3.19369688	0.001405	0.00935	Up	TDRD9
RGS8	2.16172722	0.65352134	0.20465882	3.19322337	0.001407	0.009355	Up	RGS8
AMHR2	2.07705628	0.66873443	0.2094475	3.19284988	0.001409	0.009359	Up	AMHR2
LYPD2	1.60255033	1.38484149	0.43378941	3.19242803	0.001411	0.009366	Up	LYPD2
CYP19A1	19.0904701	0.52103943	0.16330249	3.1906399	0.00142	0.009413	Up	CYP19A1
USP2	178.802932	0.571328	0.17913834	3.18931166	0.001426	0.009433	Up	USP2
R3HDML	64.5278467	-0.50633922	0.15878717	-3.18879181	0.001429	0.009441	Down	R3HDML
ZFP42	1.49627487	1.31148154	0.41146561	3.18734179	0.001436	0.009476	Up	ZFP42
PAX5	123.454033	0.70593663	0.22161627	3.18540068	0.001446	0.009526	Up	PAX5
C10orf90	1.93238491	0.80106623	0.25156433	3.18433945	0.001451	0.009543	Up	C10orf90
PYDC1	2.99462019	1.14841033	0.36064123	3.18435674	0.001451	0.009543	Up	PYDC1
SFRP1	239.48443	0.79513887	0.24974742	3.18377208	0.001454	0.009554	Up	SFRP1
SPATA22	1.09576532	0.98612926	0.30984803	3.1826223	0.001459	0.009574	Up	SPATA22
NRG2	10.0911337	0.59384579	0.18673706	3.18011751	0.001472	0.009628	Up	NRG2
SLC30A8	2.67829181	0.85490143	0.26892046	3.17901218	0.001478	0.009656	Up	SLC30A8
PITX3	0.83259486	0.84214509	0.26511454	3.17653302	0.00149	0.009718	Up	PITX3
GP9	4.19039996	0.73162821	0.23034045	3.17629061	0.001492	0.009722	Up	GP9
ANXA8	2.84610035	0.90186319	0.28397578	3.17584544	0.001494	0.009733	Up	ANXA8
NKX6-1	2.24949249	1.17770047	0.37107541	3.17374968	0.001505	0.009782	Up	NKX6-1
RUNDC3A	40.6509507	0.54993316	0.17330673	3.17317827	0.001508	0.00979	Up	RUNDC3A
CBLN2	16.725294	0.57874098	0.18250211	3.17114679	0.001518	0.00984	Up	CBLN2
COL20A1	2.93221574	0.83739017	0.26445064	3.16652736	0.001543	0.009971	Up	COL20A1
KCNS1	8.46848368	0.61476062	0.19417124	3.16607462	0.001545	0.009983	Up	KCNS1
LTK	245.35351	0.54531237	0.17248421	3.16152055	0.001569	0.010114	Up	LTK
OR52W1	0.9508714	0.96248326	0.30478364	3.15792292	0.001589	0.010205	Up	OR52W1
HRNR	5.29672322	0.707118	0.22403901	3.15622715	0.001598	0.010256	Up	HRNR
PCSK1N	247.273066	0.81873371	0.25943442	3.15584074	0.0016	0.010257	Up	PCSK1N
HORMAD1	13.9469453	0.76643597	0.24299772	3.15408707	0.00161	0.01029	Up	HORMAD1
TMEM72	98.3603619	0.70936592	0.2249668	3.15320265	0.001615	0.010317	Up	TMEM72
GABRR3	0.45710947	1.40559473	0.44582523	3.15279317	0.001617	0.010328	Up	GABRR3
CNDP1	12.6393447	0.7559736	0.23981015	3.15238362	0.001619	0.010332	Up	CNDP1
IRF4	209.748785	0.51824547	0.16451653	3.15011181	0.001632	0.010395	Up	IRF4
IRX4	0.79627087	1.19091056	0.3783547	3.14760345	0.001646	0.010474	Up	IRX4
KDM4E	0.67601389	1.02513212	0.32579008	3.14660326	0.001652	0.010502	Up	KDM4E
CACNG7	1.91590943	0.79452698	0.25264265	3.14486487	0.001662	0.010549	Up	CACNG7
RANBP3L	6.32069736	0.64480753	0.20508706	3.1440674	0.001666	0.010571	Up	RANBP3L
SAG	3.12950848	0.62401979	0.19855809	3.14275676	0.001674	0.010602	Up	SAG
REC114	1.30890752	0.95084141	0.30298865	3.13820805	0.0017	0.01074	Up	REC114
PLA2G2C	2.83697967	0.78983797	0.2517379	3.137541	0.001704	0.01076	Up	PLA2G2C
ABCA6	61.3939753	0.57229323	0.1824626	3.13649616	0.00171	0.010795	Up	ABCA6
DACH2	2.75388062	0.76516218	0.24402036	3.13564888	0.001715	0.010818	Up	DACH2
GABBR2	4.97194946	0.53195363	0.16970102	3.13465193	0.001721	0.010851	Up	GABBR2
DPEP3	3.81146304	0.67313774	0.21480045	3.13378176	0.001726	0.010867	Up	DPEP3
FAT3	19.0387766	0.50918316	0.1625925	3.1316522	0.001738	0.010926	Up	FAT3
PWP2	62.1491174	-0.54858947	0.17525847	-3.13017376	0.001747	0.010964	Down	PWP2
SUPT20HL1	0.77297687	0.86415566	0.27619824	3.12875152	0.001756	0.011002	Up	SUPT20HL1
SUPT20HL2	0.36456672	1.3691562	0.4377991	3.12736186	0.001764	0.011039	Up	SUPT20HL2
SCN1A	1.99504261	1.10584371	0.3539161	3.12459282	0.001781	0.011098	Up	SCN1A
C6orf118	0.68178692	1.47645792	0.4727103	3.12338854	0.001788	0.011132	Up	C6orf118
ELMOD1	4.03555546	0.59472291	0.19046383	3.12249799	0.001793	0.011153	Up	ELMOD1
KRT6A	133.657859	0.88454684	0.28332266	3.12204765	0.001796	0.011166	Up	KRT6A
LONRF2	45.9922033	0.8329818	0.26694152	3.12046549	0.001806	0.011214	Up	LONRF2
RGSL1	0.97266313	1.21657415	0.39008631	3.11873069	0.001816	0.011268	Up	RGSL1
CIDEA	4.73519978	1.60455756	0.51469872	3.11746949	0.001824	0.011296	Up	CIDEA

FAM180B	3.30296367	0.96965461	0.31121957	3.11566083	0.001835	0.011345	Up	FAM180B
HGFAC	3.69072816	0.63587042	0.20410199	3.11545434	0.001837	0.011349	Up	HGFAC
CCL23	15.5197989	0.5954339	0.19116337	3.11479079	0.001841	0.011367	Up	CCL23
GRM4	23.0159928	0.70092484	0.22524649	3.11181254	0.001859	0.011457	Up	GRM4
BEX1	16.698587	0.7108462	0.22874279	3.1076223	0.001886	0.011592	Up	BEX1
IGF2BP1	140.159411	0.88173813	0.2838735	3.10609524	0.001896	0.011639	Up	IGF2BP1
CAMP	2.95929908	0.62292733	0.20058304	3.10558328	0.001899	0.011653	Up	CAMP
HTN1	0.87411165	2.13399163	0.68738438	3.10450993	0.001906	0.011677	Up	HTN1
LRTM1	0.54938542	1.02726692	0.33088294	3.10462343	0.001905	0.011677	Up	LRTM1
PRDM7	1.2283179	1.07339621	0.34579741	3.10411875	0.001908	0.011684	Up	PRDM7
SLITRK5	17.2627844	0.72006437	0.23205691	3.10296458	0.001916	0.011713	Up	SLITRK5
LCN6	3.29210587	0.70122242	0.22599822	3.10277852	0.001917	0.011716	Up	LCN6
STAP1	19.5156879	0.55380642	0.17855952	3.10152278	0.001925	0.011761	Up	STAP1
ZBBX	0.71541162	1.59735298	0.51522493	3.10030218	0.001933	0.011792	Up	ZBBX
APOD	590.344459	0.54434021	0.1755853	3.10014686	0.001934	0.011792	Up	APOD
HOXD12	6.09022829	0.82020102	0.26484222	3.09694204	0.001955	0.011886	Up	HOXD12
GBP6	3.72416826	0.65912754	0.21322595	3.09121628	0.001993	0.012045	Up	GBP6
CR2	187.083567	0.7406412	0.23963693	3.09068057	0.001997	0.012055	Up	CR2
ACOT12	0.59116064	1.30422122	0.42197579	3.09074893	0.001997	0.012055	Up	ACOT12
TMEM229A	6.19670459	1.04604096	0.33849433	3.09027614	0.002	0.012062	Up	TMEM229A
CCDC177	1.02105016	0.91203582	0.29517177	3.08984773	0.002003	0.012066	Up	CCDC177
UGT2B10	10.4219507	0.75515301	0.24447763	3.088843	0.002009	0.012099	Up	UGT2B10
PPY	2.45816945	1.09202759	0.35384203	3.0862009	0.002027	0.012185	Up	PPY
CXCL13	243.395702	0.61355898	0.19881585	3.08606672	0.002028	0.012186	Up	CXCL13
SPATA21	2.50447057	0.77546491	0.25146129	3.08383418	0.002044	0.012248	Up	SPATA21
PCP4L1	21.3378896	0.72843558	0.23635076	3.08201077	0.002056	0.012293	Up	PCP4L1
ADH1A	2.36365277	0.86759874	0.28164927	3.08042243	0.002067	0.012333	Up	ADH1A
NUTM1	3.60746365	0.66725911	0.21690651	3.07625212	0.002096	0.012481	Up	NUTM1
MAB21L1	6.5686863	0.71719513	0.23358189	3.07042264	0.002138	0.012675	Up	MAB21L1
KCNQ5	11.1520373	0.65563728	0.21370825	3.06790808	0.002156	0.012768	Up	KCNQ5
LEFTY2	44.7941904	0.66485561	0.2167697	3.06710575	0.002161	0.012781	Up	LEFTY2
TGM4	2.33053281	0.52265475	0.17047446	3.06588297	0.00217	0.012816	Up	TGM4
HTR3A	13.2430786	0.72778835	0.23744698	3.06505631	0.002176	0.012842	Up	HTR3A
SPOCK3	5.40277346	1.00074589	0.32671429	3.06306125	0.002191	0.012906	Up	SPOCK3
NCAN	2.14069418	0.71681036	0.23411548	3.06178109	0.0022	0.012943	Up	NCAN
CDK5R2	15.2916006	0.61248057	0.20009688	3.06092021	0.002207	0.012967	Up	CDK5R2
ART1	0.9155313	0.97857678	0.31977378	3.06021587	0.002212	0.012993	Up	ART1
PNLIPRP3	0.76273424	1.91670969	0.62637514	3.0600028	0.002213	0.012994	Up	PNLIPRP3
NAA11	1.30055441	1.36167001	0.44535674	3.05748151	0.002232	0.013059	Up	NAA11
RNF17	1.60620657	0.97112554	0.31773164	3.05643326	0.00224	0.013087	Up	RNF17
TM4SF20	461.553357	0.63246382	0.2071437	3.0532611	0.002264	0.013203	Up	TM4SF20
PRKACG	1.05485293	0.83521555	0.27370045	3.0515681	0.002276	0.01326	Up	PRKACG
DLL3	21.3341892	0.611305	0.20035758	3.05107005	0.00228	0.013267	Up	DLL3
PCDH9	36.9247094	0.6143755	0.20140793	3.05040379	0.002285	0.013276	Up	PCDH9
PRDM14	0.68932821	1.40336207	0.46023742	3.0492133	0.002294	0.013324	Up	PRDM14
DNTT	0.71183884	1.35033853	0.44289167	3.04891381	0.002297	0.013328	Up	DNTT
SCGN	59.5751655	0.80024409	0.26256465	3.04779825	0.002305	0.01336	Up	SCGN
OR1J1	1.0886069	1.17943455	0.38697459	3.0478346	0.002305	0.01336	Up	OR1J1
ZDHHC11B	174.417619	-0.52444757	0.17207175	-3.0478424	0.002305	0.01336	Down	ZDHHC11B
NYAP2	1.51950699	0.89344292	0.29329747	3.04620063	0.002318	0.013413	Up	NYAP2
SLC6A15	7.16636475	0.8728734	0.28664273	3.04516146	0.002326	0.013454	Up	SLC6A15
REG3G	4.74193192	1.19226734	0.39165037	3.04421347	0.002333	0.013488	Up	REG3G
PTGDR	128.771949	0.67767064	0.2227662	3.04207127	0.00235	0.013557	Up	PTGDR
KLHL10	1.82422527	0.59816469	0.1966427	3.04188601	0.002351	0.013561	Up	KLHL10
B3GALT5	1451.85353	0.5823761	0.19152932	3.04066287	0.002361	0.013602	Up	B3GALT5
FLG	8.23900293	0.61098299	0.20095624	3.04037825	0.002363	0.01361	Up	FLG
SCN7A	79.8911938	0.84366939	0.27761754	3.03896283	0.002374	0.013661	Up	SCN7A
FOXN4	1.91469331	0.92520025	0.30498128	3.0336296	0.002416	0.013867	Up	FOXN4

TACR1	19.9346957	0.63042068	0.20788855	3.03249352	0.002425	0.013906	Up	TACR1
KCNF1	26.2347981	0.58103407	0.19174324	3.03027147	0.002443	0.013985	Up	KCNF1
VTN	13.6712958	0.51654333	0.17048523	3.02984212	0.002447	0.014	Up	VTN
KCNC1	4.76875982	0.52665235	0.17394904	3.02762445	0.002465	0.01408	Up	KCNC1
AADACL2	2.20553787	1.42712331	0.47160108	3.02612392	0.002477	0.014141	Up	AADACL2
SYT6	3.38721215	0.67916049	0.22445376	3.02583696	0.002479	0.014143	Up	SYT6
GJB7	11.565679	0.98391055	0.32517598	3.02577869	0.00248	0.014143	Up	GJB7
IQCF1	0.86967197	0.84022151	0.27775645	3.02502967	0.002486	0.014172	Up	IQCF1
LYVE1	195.199765	0.60821049	0.20121006	3.02276383	0.002505	0.014254	Up	LYVE1
GPR15	58.7623849	0.74046863	0.24499197	3.02241996	0.002508	0.014263	Up	GPR15
WFDC12	2.97172502	0.55350567	0.18336844	3.01854391	0.00254	0.014401	Up	WFDC12
MYH7	4.20788136	0.54063119	0.17917282	3.01737282	0.00255	0.014436	Up	MYH7
TBL1Y	3.030638	1.37127778	0.45446215	3.01736411	0.00255	0.014436	Up	TBL1Y
ELAVL3	4.06858803	0.75508129	0.25036461	3.01592663	0.002562	0.01448	Up	ELAVL3
HMGCLL1	5.88793673	0.5750629	0.19074705	3.01479307	0.002572	0.014513	Up	HMGCLL1
CHRM4	10.7456596	0.56687866	0.1880357	3.01473947	0.002572	0.014513	Up	CHRM4
SCGB2A1	30.7225441	0.66212921	0.21990704	3.01095056	0.002604	0.014667	Up	SCGB2A1
SORCS3	2.98191221	0.95830101	0.3184332	3.00942554	0.002617	0.014726	Up	SORCS3
EPHA6	5.50396122	0.87835272	0.29206899	3.00734673	0.002635	0.014798	Up	EPHA6
WFDC9	0.64742577	-1.00017792	0.33276933	-3.00561929	0.00265	0.014858	Down	WFDC9
GABRQ	5.54851284	0.54906337	0.18305012	2.99952481	0.002704	0.015094	Up	GABRQ
DCDC1	3.87931455	0.71080172	0.23701675	2.99895145	0.002709	0.015113	Up	DCDC1
PLIN4	389.32432	0.75285836	0.25109012	2.99835911	0.002714	0.015132	Up	PLIN4
KCNK2	10.7724058	0.68857454	0.22988352	2.99531932	0.002742	0.015239	Up	KCNK2
FAM81B	1.35139087	1.0315643	0.34494324	2.99053342	0.002785	0.01546	Up	FAM81B
LSMEM2	2.76184693	0.54733563	0.18319281	2.98775723	0.00281	0.015566	Up	LSMEM2
EPGN	2.52712514	0.82676693	0.27694094	2.98535463	0.002832	0.015659	Up	EPGN
CCL18	738.177993	0.58198252	0.19503226	2.98403201	0.002845	0.015721	Up	CCL18
SULT1E1	25.0794108	0.76183237	0.2555408	2.98125531	0.002871	0.015824	Up	SULT1E1
CCDC160	2.21609237	0.90111271	0.30241592	2.97971321	0.002885	0.015871	Up	CCDC160
SVOP	7.9264726	0.60747924	0.20391951	2.97901484	0.002892	0.015892	Up	SVOP
MUC17	3022.4497	0.7473214	0.25090261	2.97853175	0.002896	0.015904	Up	MUC17
RET	129.250566	0.53055744	0.17821843	2.9770066	0.002911	0.015973	Up	RET
ZNF536	21.3061795	0.55965832	0.18810863	2.97518679	0.002928	0.016037	Up	ZNF536
OSM	208.135531	0.50872663	0.17107821	2.97364943	0.002943	0.016097	Up	OSM
BTNL8	540.472501	0.57425352	0.19326459	2.97133342	0.002965	0.016183	Up	BTNL8
FGF14	17.0264633	0.6152755	0.20708776	2.97108582	0.002967	0.016187	Up	FGF14
SLC12A1	2.40411821	0.95372491	0.32113501	2.96985653	0.002979	0.016237	Up	SLC12A1
ATP12A	29.5134048	0.88145361	0.29710527	2.9668057	0.003009	0.016366	Up	ATP12A
ASPA	30.4678214	0.57227794	0.19291452	2.96648448	0.003012	0.016372	Up	ASPA
BOD1L2	0.46317313	1.17153681	0.39493631	2.96639427	0.003013	0.016372	Up	BOD1L2
ARX	21.0470036	0.74735287	0.25220055	2.96332761	0.003043	0.0165	Up	ARX
VWCE	56.3814749	0.50347574	0.16993104	2.96282396	0.003048	0.016516	Up	VWCE
VWC2L	0.53372662	1.49402379	0.50468242	2.9603246	0.003073	0.016617	Up	VWC2L
AKAP14	3.02652097	0.64698325	0.21868277	2.95854698	0.003091	0.0167	Up	AKAP14
CRYBA1	1.779977	0.73123993	0.24725572	2.95742371	0.003102	0.01675	Up	CRYBA1
LIPC	23.7688594	0.56933698	0.19262607	2.95565911	0.00312	0.01682	Up	LIPC
PAX4	9.49361998	0.9469616	0.32048996	2.95473091	0.003129	0.016855	Up	PAX4
AFF2	9.18844719	0.55174636	0.1867521	2.9544319	0.003132	0.016866	Up	AFF2
LIPJ	1.42708996	0.66184676	0.22415553	2.95262297	0.003151	0.016944	Up	LIPJ
LRRN4	13.0478326	0.50719016	0.17192057	2.95014236	0.003176	0.017048	Up	LRRN4
PNPLA3	49.7219136	0.62690536	0.21256162	2.94928773	0.003185	0.01709	Up	PNPLA3
TTC29	7.27799345	1.27851408	0.43413511	2.94496816	0.00323	0.017276	Up	TTC29
CDH12	0.77327157	1.26238567	0.42864463	2.94506357	0.003229	0.017276	Up	CDH12
MIA2	5.07816614	0.586521	0.19949057	2.94009392	0.003281	0.017469	Up	MIA2
KCNA3	25.7027475	0.50591469	0.1723489	2.93540997	0.003331	0.017707	Up	KCNA3
BARX2	265.536275	0.50508295	0.17213695	2.93419248	0.003344	0.017771	Up	BARX2
SCN4A	10.6266743	0.5287989	0.18027888	2.93322707	0.003355	0.017812	Up	SCN4A

ALOXE3	9.51536334	0.56984974	0.19429447	2.93291797	0.003358	0.017822	Up	ALOXE3
CALCB	22.0737475	0.92740097	0.31668459	2.92846892	0.003406	0.018026	Up	CALCB
GFRA2	41.5008882	0.53626719	0.18319186	2.92735275	0.003419	0.018072	Up	GFRA2
ARSF	0.7527008	0.73648984	0.25178595	2.92506328	0.003444	0.018177	Up	ARSF
CHST9	3.98137142	0.98110239	0.33542318	2.92496896	0.003445	0.018177	Up	CHST9
SERPINB3	5.65553933	1.35664616	0.46414552	2.92288967	0.003468	0.018282	Up	SERPINB3
KLK5	4.75574266	1.13853454	0.3897348	2.92130585	0.003486	0.01837	Up	KLK5
OPN4	2.41150802	0.60766272	0.20806546	2.92053617	0.003494	0.018409	Up	OPN4
GABRG2	4.48496437	1.0455622	0.35809594	2.9197823	0.003503	0.018443	Up	GABRG2
LHFPL4	24.593858	0.8669167	0.29694755	2.91942701	0.003507	0.018458	Up	LHFPL4
SLC39A12	0.71161842	0.86910171	0.29780858	2.91832327	0.003519	0.018507	Up	SLC39A12
MARCO	188.832593	0.66934352	0.22940331	2.91775876	0.003526	0.018523	Up	MARCO
CCL8	72.6240873	0.55596638	0.19068154	2.91568017	0.003549	0.018616	Up	CCL8
CYP2C19	2.36724642	0.79223862	0.27171233	2.91572566	0.003549	0.018616	Up	CYP2C19
ABRA	1.94670207	0.71816292	0.24719082	2.90529774	0.003669	0.019084	Up	ABRA
ANP32D	1.64818398	0.64743254	0.22286215	2.90508071	0.003672	0.019091	Up	ANP32D
TECTB	2.66982021	0.63340132	0.21808045	2.90443875	0.003679	0.019119	Up	TECTB
POU3F3	6.82192514	0.73065386	0.25179616	2.90176727	0.003711	0.019259	Up	POU3F3
HS3ST6	3.77696113	0.92945167	0.3205376	2.89966503	0.003736	0.019354	Up	HS3ST6
TRIM71	26.2233342	0.95518384	0.32946043	2.89923687	0.003741	0.019369	Up	TRIM71
PAX1	0.90324729	1.14475939	0.39504966	2.89776071	0.003758	0.019437	Up	PAX1
MAB21L2	462.976045	0.52996061	0.18290369	2.89748457	0.003762	0.019445	Up	MAB21L2
LHB	3.1650982	0.54985579	0.18983964	2.89642237	0.003774	0.019502	Up	LHB
OMD	49.0727298	0.74990833	0.25896085	2.89583671	0.003781	0.019521	Up	OMD
MEOX2	44.7069975	0.5700798	0.19690828	2.89515412	0.00379	0.019546	Up	MEOX2
VRTN	11.6377449	0.6544431	0.22615376	2.89379714	0.003806	0.019613	Up	VRTN
SEL1L2	1.21911404	0.79865574	0.27626117	2.89094463	0.003841	0.019756	Up	SEL1L2
IGSF1	27.9122274	0.60846834	0.21049128	2.89070564	0.003844	0.019765	Up	IGSF1
UNC45B	2.06425091	0.52291557	0.18104226	2.88836197	0.003873	0.019847	Up	UNC45B
SEZ6L	10.6817619	0.62264384	0.21561211	2.88779619	0.00388	0.019872	Up	SEZ6L
VTCN1	14.508144	0.79778396	0.27629735	2.88741083	0.003884	0.019884	Up	VTCN1
EPHA7	87.1212194	0.59896946	0.20747342	2.88696959	0.00389	0.0199	Up	EPHA7
OR10A5	0.8405296	2.63248739	0.91368173	2.8811864	0.003962	0.020214	Up	OR10A5
IL5	2.93770127	0.56758527	0.19703185	2.88067774	0.003968	0.020235	Up	IL5
MYH2	2.22438748	1.12930535	0.39217613	2.87958714	0.003982	0.020293	Up	MYH2
TTC24	4.91285595	0.56696078	0.19698745	2.87815686	0.004	0.02036	Up	TTC24
ANKRD34C	0.77929009	1.09514178	0.38051088	2.87808268	0.004001	0.02036	Up	ANKRD34C
PRPH	39.2794124	0.63650763	0.22130376	2.8761718	0.004025	0.020459	Up	PRPH
FOXL2	2.488725	0.93169121	0.32405855	2.87507063	0.004039	0.020506	Up	FOXL2
GSG1	1.40211044	0.89584814	0.31169926	2.87407848	0.004052	0.020553	Up	GSG1
F13A1	654.80884	0.51018722	0.17765964	2.87171142	0.004083	0.02068	Up	F13A1
C22orf42	0.47261825	0.91970032	0.32026188	2.87171337	0.004083	0.02068	Up	C22orf42
OR51B4	0.75123357	1.72613794	0.60122691	2.87102576	0.004091	0.020703	Up	OR51B4
IFI44L	437.425878	0.50092906	0.17465174	2.86815957	0.004129	0.020848	Up	IFI44L
CLDN19	1.689349	0.85888735	0.29967478	2.86606485	0.004156	0.020926	Up	CLDN19
KLRC4	1.79952828	0.66000188	0.23031094	2.86569919	0.004161	0.02094	Up	KLRC4
RFX4	2.83437832	0.55860269	0.19494704	2.8654074	0.004165	0.020951	Up	RFX4
VWA5B2	35.0090037	0.50890776	0.17777199	2.86269934	0.0042	0.021087	Up	VWA5B2
RAD21L1	0.96466841	1.08351309	0.37878062	2.86052935	0.004229	0.021189	Up	RAD21L1
NDUFA7	1.60530111	0.52948966	0.18525897	2.85810532	0.004262	0.021301	Up	NDUFA7
HTR3B	0.53222904	1.31629701	0.46079055	2.85660594	0.004282	0.021371	Up	HTR3B
P2RX2	4.70639884	0.74810801	0.26195362	2.85587962	0.004292	0.021414	Up	P2RX2
RPRM	17.6784739	0.58885082	0.20624614	2.85508768	0.004302	0.021461	Up	RPRM
WDR49	1.74171071	0.81435744	0.28542194	2.85317042	0.004329	0.021565	Up	WDR49
GDPD2	143.882067	0.51774993	0.18160643	2.85094488	0.004359	0.021695	Up	GDPD2
SYNDIG1L	3.43447431	-0.58838235	0.2065488	-2.84863595	0.004391	0.021812	Down	SYNDIG1L
RPS4Y1	3407.61853	0.91663755	0.32187708	2.84778754	0.004402	0.021858	Up	RPS4Y1
GPR42	0.73809129	0.72435683	0.25445904	2.84665394	0.004418	0.021907	Up	GPR42

ZG16	5482.3422	0.88415444	0.3106593	2.84605817	0.004426	0.021939	Up	ZG16
GH1	0.57618198	0.83416004	0.29314204	2.8455831	0.004433	0.021959	Up	GH1
WNT7B	47.5535288	0.61821651	0.21743661	2.84320343	0.004466	0.022104	Up	WNT7B
RSPO2	32.9147472	0.64836629	0.22821504	2.84103222	0.004497	0.02223	Up	RSPO2
GABRA5	0.75188264	1.16259463	0.40930113	2.84043835	0.004505	0.022265	Up	GABRA5
TNFRSF17	77.8093072	0.60024698	0.2113371	2.84023481	0.004508	0.022273	Up	TNFRSF17
CLRN1	0.61748808	1.50169849	0.52950781	2.83602709	0.004568	0.022497	Up	CLRN1
FAM170B	0.2942666	0.96390426	0.33990607	2.835796	0.004571	0.022507	Up	FAM170B
TSPAN19	0.49629064	1.35795783	0.47923476	2.8335963	0.004603	0.022604	Up	TSPAN19
HRH4	4.46457669	0.51732421	0.18262442	2.83272204	0.004615	0.022647	Up	HRH4
ASTN1	8.65388235	0.78427421	0.27686322	2.83271359	0.004615	0.022647	Up	ASTN1
MEI4	1.31792944	0.9677216	0.34188367	2.83055815	0.004647	0.022761	Up	MEI4
LMX1B	8.66314409	0.70743581	0.25002936	2.82941095	0.004663	0.022797	Up	LMX1B
CXCL11	523.091326	0.53990003	0.19083457	2.8291521	0.004667	0.022809	Up	CXCL11
AQP12B	9.12169991	-0.52261683	0.18486248	-2.82705735	0.004698	0.022946	Down	AQP12B
SLC36A3	0.57978888	1.20878697	0.42811992	2.82347751	0.004751	0.023137	Up	SLC36A3
NMRK2	1.34310353	1.02744914	0.36396402	2.8229415	0.004759	0.023163	Up	NMRK2
VSX2	0.44747079	0.94234219	0.33422614	2.81947487	0.00481	0.023355	Up	VSX2
IL22	3.06056654	0.80977226	0.28729774	2.81858203	0.004824	0.023414	Up	IL22
RGS22	6.60043857	0.58478609	0.20751059	2.8181023	0.004831	0.023442	Up	RGS22
LGALS7B	5.41516303	0.63505214	0.22550886	2.81608505	0.004861	0.023556	Up	LGALS7B
GKN2	0.51918862	1.04707164	0.3718538	2.81581538	0.004865	0.023563	Up	GKN2
KCNA4	0.68340607	1.25393466	0.44665933	2.80736252	0.004995	0.024095	Up	KCNA4
SERPINB10	0.42790152	1.06017714	0.37788994	2.80551827	0.005024	0.02422	Up	SERPINB10
SIX2	49.5862569	0.65182221	0.23234675	2.80538554	0.005026	0.024223	Up	SIX2
FCRLA	42.7718277	0.5827737	0.20812169	2.80015845	0.005108	0.024515	Up	FCRLA
EPHA5	3.88533366	0.68704379	0.24534714	2.80029268	0.005106	0.024515	Up	EPHA5
SLC4A1	2.14932799	0.7001868	0.25006528	2.80001602	0.00511	0.024519	Up	SLC4A1
ST8SIA5	4.66328496	0.50225427	0.17939191	2.79975985	0.005114	0.024532	Up	ST8SIA5
PPP1R42	0.81443599	1.01908502	0.36428346	2.7975056	0.00515	0.024642	Up	PPP1R42
IGSF5	4.61260554	0.50955052	0.18228576	2.7953391	0.005185	0.024759	Up	IGSF5
LGALS9C	165.157789	0.55701296	0.1993222	2.79453545	0.005197	0.024797	Up	LGALS9C
TFF1	4671.26953	0.57626834	0.20627314	2.79371489	0.005211	0.024846	Up	TFF1
RIMBP3B	0.44779544	0.81269704	0.29095175	2.79323643	0.005218	0.024865	Up	RIMBP3B
FDCSP	68.8952106	0.74634867	0.26734626	2.791693	0.005243	0.024957	Up	FDCSP
NOS1	31.05498	0.69689158	0.24991026	2.78856735	0.005294	0.025115	Up	NOS1
RNF150	160.114775	0.51477507	0.18466229	2.78765677	0.005309	0.025172	Up	RNF150
SLCO4C1	27.4771291	0.61943761	0.22242665	2.78490734	0.005354	0.025351	Up	SLCO4C1
GRIA3	12.472323	0.51248438	0.18412192	2.78339681	0.005379	0.025463	Up	GRIA3
SULT1A2	98.0096874	0.51430744	0.18481732	2.78278805	0.005389	0.025496	Up	SULT1A2
GLP1R	1.93725309	0.74699694	0.26855641	2.78152715	0.00541	0.025574	Up	GLP1R
ZNF385B	14.3750423	0.55128544	0.19824005	2.78089846	0.005421	0.025596	Up	ZNF385B
TTR	29.7900059	0.67483994	0.24297885	2.77736083	0.00548	0.025833	Up	TTR
GPR88	5.32674639	0.5595161	0.2016565	2.77459982	0.005527	0.025997	Up	GPR88
LRRC53	1.41673184	1.05957424	0.38202824	2.77354954	0.005545	0.026063	Up	LRRC53
CAPSL	0.95013091	0.91126689	0.32882974	2.77124232	0.005584	0.026208	Up	CAPSL
KCNH6	22.684645	0.53692022	0.19377053	2.77090748	0.00559	0.026214	Up	KCNH6
ATP2B2	4.37070816	0.53909084	0.19462528	2.76989111	0.005608	0.02628	Up	ATP2B2
LHFPL5	1.92383747	0.60664893	0.21902074	2.76982415	0.005609	0.02628	Up	LHFPL5
XKR3	0.41406094	1.03962725	0.3756706	2.76739048	0.005651	0.026424	Up	XKR3
DHRS9	904.360716	0.60103868	0.21725877	2.76646449	0.005667	0.02648	Up	DHRS9
RTP5	1.69332322	0.63493538	0.22958101	2.76562673	0.005681	0.026526	Up	RTP5
RAB3C	30.3529271	0.52544056	0.1900069	2.76537621	0.005686	0.026536	Up	RAB3C
MIOX	12.4904559	0.58936352	0.21313387	2.76522698	0.005688	0.026537	Up	MIOX
LY6G6F	8.8999417	-0.60166479	0.21763051	-2.76461596	0.005699	0.026566	Down	LY6G6F
DRD1	20.3297794	-0.53654315	0.1941211	-2.76396097	0.00571	0.026598	Down	DRD1
CNGB3	5.12598855	0.61427527	0.22232264	2.76299016	0.005727	0.026662	Up	CNGB3
TEPP	1.47065628	0.55095535	0.19953109	2.76125065	0.005758	0.026761	Up	TEPP

LCN15	1028.29569	0.85932922	0.3112861	2.76057693	0.00577	0.026797	Up	LCN15
SLC17A8	5.66162645	0.98978748	0.35856043	2.76044819	0.005772	0.026797	Up	SLC17A8
MGAT4C	4.60656276	0.84870555	0.30748684	2.76013619	0.005778	0.026814	Up	MGAT4C
OGN	219.843237	0.72869718	0.26457323	2.75423623	0.005883	0.027216	Up	OGN
CACNA1B	26.1048978	0.67680836	0.24582826	2.75317556	0.005902	0.027282	Up	CACNA1B
LDB3	83.9799046	0.56033502	0.20355171	2.75278946	0.005909	0.027299	Up	LDB3
BHLHA9	6.88939067	0.92621957	0.33681809	2.74991041	0.005961	0.027452	Up	BHLHA9
LMOD2	0.83869793	0.85140782	0.3096793	2.74932108	0.005972	0.027486	Up	LMOD2
ZNF728	1.30356591	0.85702185	0.31216427	2.74541945	0.006043	0.027763	Up	ZNF728
SPRR2D	20.2922696	0.90728597	0.33124923	2.73898286	0.006163	0.028214	Up	SPRR2D
SLC6A11	1.54877463	0.65230256	0.2382101	2.73834968	0.006175	0.028258	Up	SLC6A11
LY6G6D	348.095777	-0.7299734	0.26676664	-2.73637442	0.006212	0.028369	Down	LY6G6D
FSHR	0.6485469	1.8155494	0.66377937	2.73516996	0.006235	0.028452	Up	FSHR
IGLL5	1751.95177	0.54980527	0.20106109	2.73451849	0.006247	0.028501	Up	IGLL5
MYBPHL	19.4688055	0.85055029	0.31108134	2.73417323	0.006254	0.028523	Up	MYBPHL
KIAA0408	2.1099564	0.86229639	0.31544501	2.73358704	0.006265	0.028567	Up	KIAA0408
FCRL2	34.1256488	0.52902315	0.19357238	2.73294751	0.006277	0.028599	Up	FCRL2
SLC38A3	44.5838568	-0.63781376	0.23353542	-2.73112213	0.006312	0.028722	Down	SLC38A3
ZPLD1	2.11242664	0.80493613	0.29482003	2.73026265	0.006328	0.02878	Up	ZPLD1
CPNE6	32.2816841	0.57604929	0.21099716	2.73012815	0.006331	0.028781	Up	CPNE6
MTRNR2L7	0.47383918	1.20743673	0.44231046	2.72983985	0.006337	0.028794	Up	MTRNR2L7
ASB18	0.88708864	0.91109066	0.33423821	2.72587221	0.006413	0.029065	Up	ASB18
STMN4	5.18075158	0.80881281	0.29673051	2.72574875	0.006416	0.029068	Up	STMN4
SCN10A	0.84382872	1.27163534	0.46726883	2.7214213	0.0065	0.029351	Up	SCN10A
CCKAR	1.13609014	1.13042426	0.41553007	2.72043911	0.00652	0.029407	Up	CCKAR
CDKL4	2.22435499	0.61476693	0.2260073	2.7201198	0.006526	0.02942	Up	CDKL4
IL37	15.5203032	0.55911543	0.20563364	2.71898813	0.006548	0.029498	Up	IL37
DHRS7C	1.79414268	1.10830626	0.40769037	2.71849995	0.006558	0.02952	Up	DHRS7C
C17orf99	9.62481221	0.520169	0.19134557	2.71847945	0.006558	0.02952	Up	C17orf99
NTRK3	26.555232	0.51463939	0.18949585	2.71583458	0.006611	0.029673	Up	NTRK3
CLEC17A	10.9006087	0.57493564	0.21180966	2.71439763	0.00664	0.029746	Up	CLEC17A
DNAI2	2.19299925	0.55011558	0.20272669	2.71358244	0.006656	0.029811	Up	DNAI2
ZFHX4	97.1010479	0.52820323	0.19484979	2.71082266	0.006712	0.02999	Up	ZFHX4
PKD1L2	9.18448826	0.52362479	0.19345475	2.70670422	0.006795	0.030278	Up	PKD1L2
LIPK	0.59020858	1.06105862	0.39286457	2.70082542	0.006917	0.030667	Up	LIPK
KRT81	18.556599	0.51414032	0.19045984	2.69946835	0.006945	0.030768	Up	KRT81
KCNC2	2.89058921	1.00815511	0.37366646	2.69800803	0.006976	0.030888	Up	KCNC2
TRPM3	11.9451575	0.60926158	0.2258496	2.69764291	0.006983	0.030907	Up	TRPM3
NCCRP1	17.6092769	0.52096187	0.1931444	2.69726618	0.006991	0.030909	Up	NCCRP1
CEACAM18	22.253717	0.79224675	0.29397555	2.69494091	0.00704	0.031077	Up	CEACAM18
INSRR	3.7823402	0.51903094	0.19279155	2.69218717	0.007099	0.031286	Up	INSRR
PSG4	0.99662438	1.17490398	0.43647238	2.69181746	0.007106	0.031297	Up	PSG4
SPAG6	1.61410859	0.71855482	0.26712076	2.68999996	0.007145	0.031444	Up	SPAG6
PHOX2B	8.55818465	0.9058565	0.33697099	2.68823287	0.007183	0.031587	Up	PHOX2B
NT5C1A	0.36993634	0.83011039	0.30894139	2.68695102	0.007211	0.031684	Up	NT5C1A
GAL3ST3	0.63466621	1.00837214	0.37540797	2.68607016	0.00723	0.031721	Up	GAL3ST3
SLCO6A1	0.47999416	1.76817545	0.65832636	2.68586457	0.007234	0.031721	Up	SLCO6A1
SPX	2.91193238	0.6164155	0.2295904	2.68484874	0.007256	0.031762	Up	SPX
A4GNT	0.81165155	0.78704213	0.29347877	2.68176852	0.007323	0.031982	Up	A4GNT
GPR87	1.6473603	0.960313	0.35808456	2.68180509	0.007323	0.031982	Up	GPR87
HRH3	1.06284803	0.88227861	0.32901833	2.6815485	0.007328	0.031995	Up	HRH3
ANKRD18B	10.571247	-0.73753599	0.27518379	-2.68015784	0.007359	0.032071	Down	ANKRD18B
ADAM29	0.72089722	0.95177154	0.35555344	2.67687341	0.007431	0.032305	Up	ADAM29
GAST	3.09658587	0.75668443	0.28275987	2.67606727	0.007449	0.032352	Up	GAST
KRT76	0.41709694	1.51504915	0.56615141	2.67604941	0.00745	0.032352	Up	KRT76
MC5R	0.92532864	0.72769572	0.27218859	2.67349823	0.007506	0.032525	Up	MC5R
PI15	139.460964	0.51150789	0.19137414	2.67281619	0.007522	0.032566	Up	PI15
CHRM2	69.2699111	0.87904981	0.32890888	2.67262413	0.007526	0.032569	Up	CHRM2

PRSS21	90.442201	0.75349704	0.28198705	2.67209802	0.007538	0.032595	Up	PRSS21
AMY2A	0.50224293	-1.09532012	0.40991011	-2.67209833	0.007538	0.032595	Down	AMY2A
FOXE1	0.7649936	1.25721243	0.4708101	2.67031749	0.007578	0.032719	Up	FOXE1
AVPR1B	1.48859129	0.90006943	0.33748446	2.66699517	0.007653	0.032994	Up	AVPR1B
TRPC5OS	0.38135168	0.99711059	0.37422292	2.66448296	0.007711	0.033167	Up	TRPC5OS
TRPV5	1.27850594	0.75089131	0.2819834	2.66289187	0.007747	0.033292	Up	TRPV5
PCDHB1	1.16952553	0.93021128	0.34959467	2.66082797	0.007795	0.03347	Up	PCDHB1
CALN1	2.90733197	0.64653716	0.24323834	2.6580397	0.00786	0.033664	Up	CALN1
SPDYE4	0.35149845	0.89256651	0.33591432	2.6571255	0.007881	0.033739	Up	SPDYE4
CPLX2	162.464097	0.56631726	0.21316882	2.65666089	0.007892	0.033768	Up	CPLX2
PGPEP1L	1.37017735	0.91294999	0.34417099	2.65260589	0.007987	0.0341	Up	PGPEP1L
MAEL	12.1476413	0.57391178	0.21642547	2.65177557	0.008007	0.034167	Up	MAEL
MS4A1	130.345885	0.62481245	0.23568744	2.65102145	0.008025	0.034209	Up	MS4A1
ANKRD30A	0.65946851	1.50331048	0.56749633	2.6490224	0.008072	0.034387	Up	ANKRD30A
SLC9C2	1.3722008	0.97026815	0.3666283	2.64646279	0.008134	0.034571	Up	SLC9C2
ANKRD62	0.87676074	0.77246613	0.29187182	2.64659371	0.008131	0.034571	Up	ANKRD62
INSM2	0.58983227	0.9295835	0.3514074	2.64531568	0.008161	0.034671	Up	INSM2
IL36RN	5.25073705	0.7037997	0.26677449	2.63818216	0.008335	0.035252	Up	IL36RN
CNGB1	5.01794919	0.59075383	0.22411934	2.63588963	0.008392	0.035447	Up	CNGB1
GLRA3	2.16894885	0.68906848	0.26143548	2.6357114	0.008396	0.035453	Up	GLRA3
MT4	4.94941468	1.4493968	0.549977	2.6353771	0.008404	0.035466	Up	MT4
BBOX1	4.54535819	0.61393442	0.23317665	2.63291556	0.008466	0.035645	Up	BBOX1
PTPRZ1	23.0811328	0.66390459	0.25221889	2.63225557	0.008482	0.035671	Up	PTPRZ1
TMEM82	108.427593	0.50201119	0.19093765	2.62918915	0.008559	0.035888	Up	TMEM82
CP	136.124081	0.51534179	0.19613231	2.62752115	0.008601	0.035988	Up	CP
SGCG	4.31478794	0.76532749	0.29125558	2.62768352	0.008597	0.035988	Up	SGCG
FFAR3	2.56898011	0.50849659	0.19357652	2.62685055	0.008618	0.036039	Up	FFAR3
IL2	0.94878377	0.68638428	0.26136675	2.62613468	0.008636	0.036071	Up	IL2
CCDC70	0.78885147	0.82439595	0.31393022	2.62604843	0.008638	0.036071	Up	CCDC70
GRPR	30.5912998	-0.60408674	0.23029559	-2.62309301	0.008714	0.036263	Down	GRPR
TREML4	2.71313324	0.8070746	0.3076591	2.6232756	0.008709	0.036263	Up	TREML4
MYH13	4.45655793	0.50378782	0.19223638	2.62066846	0.008776	0.036424	Up	MYH13
DEFB124	1.01792958	0.71301852	0.27232741	2.61824002	0.008838	0.036569	Up	DEFB124
HAND2	125.174962	0.58997583	0.22548798	2.61644031	0.008885	0.036709	Up	HAND2
CCNA1	2.90761843	0.51577451	0.19732057	2.6138913	0.008952	0.036913	Up	CCNA1
SLC6A5	0.55417392	1.77257886	0.67869438	2.61174823	0.009008	0.037101	Up	SLC6A5
CRYGN	2.32776223	0.50259759	0.19261445	2.60934522	0.009072	0.037326	Up	CRYGN
ABCA8	164.266776	0.61228805	0.2346458	2.60941406	0.00907	0.037326	Up	ABCA8
CPB1	7.61429053	0.83215233	0.31900339	2.60860027	0.009091	0.037382	Up	CPB1
SMLR1	4.26663481	0.5455727	0.20914442	2.60859309	0.009092	0.037382	Up	SMLR1
SPIC	0.66812172	0.7227866	0.27737089	2.60584875	0.009165	0.037636	Up	SPIC
DCX	3.70818569	0.5957533	0.22873025	2.60461085	0.009198	0.037728	Up	DCX
TMPRSS11D	1.91815925	1.12170081	0.43084093	2.60351499	0.009227	0.037822	Up	TMPRSS11D
PRSS55	0.97131551	1.10406761	0.42419378	2.60274349	0.009248	0.03788	Up	PRSS55
AQP4	1.79830154	0.83925759	0.32249088	2.60242269	0.009257	0.037907	Up	AQP4
CPLX4	0.44802526	0.90449644	0.3477368	2.60109494	0.009293	0.037999	Up	CPLX4
CYP26A1	12.857432	0.59441937	0.22862425	2.59998397	0.009323	0.038095	Up	CYP26A1
FGG	10.2389748	1.10814994	0.42630584	2.59942471	0.009338	0.038139	Up	FGG
GJB5	144.431343	0.65206178	0.25093458	2.598533	0.009362	0.038193	Up	GJB5
PNCK	89.5386981	0.54369346	0.20928844	2.59781891	0.009382	0.038263	Up	PNCK
UGT1A5	0.60974024	1.33287335	0.51313957	2.59748699	0.009391	0.038273	Up	UGT1A5
PNMA5	9.47917366	0.82852468	0.31898881	2.59734719	0.009395	0.038279	Up	PNMA5
PAEP	11.3935581	0.59412373	0.22922157	2.59191901	0.009544	0.038733	Up	PAEP
KRTAP5-5	13.1191665	-0.51117857	0.19725472	-2.59146426	0.009557	0.038756	Down	KRTAP5-5
ASB5	11.6656804	1.02299811	0.39486028	2.59078507	0.009576	0.038805	Up	ASB5
PENK	21.8589536	0.74900046	0.2892958	2.58904713	0.009624	0.038918	Up	PENK
CALHM1	2.52491828	0.53839234	0.20808187	2.58740631	0.00967	0.039052	Up	CALHM1
KBTBD13	0.36060344	0.82803268	0.32002718	2.58738237	0.009671	0.039052	Up	KBTBD13

LRTM2	1.67344571	0.64057378	0.24759985	2.58713313	0.009678	0.039064	Up	LRTM2
PRTN3	0.662054	0.82167026	0.31760149	2.58711087	0.009678	0.039064	Up	PRTN3
GNGT1	12.8325075	-0.65904894	0.2552581	-2.58189236	0.009826	0.039521	Down	GNGT1
ERVV-1	2.35930087	0.79457449	0.30799951	2.57979145	0.009886	0.039714	Up	ERVV-1
DRC7	2.56579958	0.54339697	0.21078297	2.57799281	0.009938	0.039871	Up	DRC7
FOXB1	2.14940988	0.61754638	0.23968296	2.57651345	0.00998	0.04001	Up	FOXB1
TGM5	1.09395641	0.70936437	0.27541456	2.57562403	0.010006	0.04008	Up	TGM5
SLC6A14	550.483199	0.51311619	0.19923552	2.57542531	0.010012	0.040089	Up	SLC6A14
AKR1C8P	3.82464821	0.70956776	0.27574432	2.57328151	0.010074	0.040291	Up	AKR1C8P
CMA1	13.2748374	0.75324174	0.29277636	2.57275465	0.010089	0.040315	Up	CMA1
SCGB3A1	3.32130205	0.5152359	0.20028609	2.57249963	0.010097	0.040335	Up	SCGB3A1
SLC32A1	1.27676428	1.11826889	0.43491258	2.57124983	0.010133	0.040453	Up	SLC32A1
UPK1A	9.12190928	0.63076667	0.24561785	2.56808158	0.010226	0.040748	Up	UPK1A
SERPINI2	0.55432416	1.09028908	0.42468912	2.567264	0.01025	0.040797	Up	SERPINI2
PRMT8	1.99900577	0.64727816	0.2521772	2.5667592	0.010265	0.040822	Up	PRMT8
OR13A1	1.99452055	0.55038513	0.2146154	2.56451837	0.010332	0.041017	Up	OR13A1
SCEL	82.7819379	0.5625432	0.21945704	2.56334086	0.010367	0.041109	Up	SCEL
CTSG	48.0230875	0.59540245	0.23243706	2.56156416	0.01042	0.041291	Up	CTSG
UCP1	0.64864341	0.85402806	0.3335712	2.56025715	0.010459	0.041427	Up	UCP1
CTNNA3	15.3956158	0.6340959	0.24773356	2.55958819	0.01048	0.041498	Up	CTNNA3
C17orf64	0.93448182	0.60921149	0.23810303	2.55860453	0.010509	0.041596	Up	C17orf64
REP15	65.5575135	-0.54503529	0.21309667	-2.55769038	0.010537	0.041677	Down	REP15
CXCR5	5.6893196	0.61748519	0.24172264	2.55451951	0.010633	0.041971	Up	CXCR5
GABRB2	64.5919478	-0.59722551	0.23465793	-2.54508986	0.010925	0.042787	Down	GABRB2
DAZL	3.58926824	0.58562799	0.23024891	2.54345609	0.010976	0.042914	Up	DAZL
KRT222	2.5642062	0.64957291	0.25541105	2.54324516	0.010983	0.042925	Up	KRT222
CSMD1	6.39551114	0.61735607	0.24285851	2.54204004	0.011021	0.043025	Up	CSMD1
SNTG1	0.47401696	1.07308796	0.42228634	2.54113824	0.011049	0.043079	Up	SNTG1
MAS1L	1.20260558	1.06512668	0.41919551	2.54088287	0.011057	0.043099	Up	MAS1L
CNTN1	65.7136364	0.57476581	0.22628465	2.54001238	0.011085	0.043167	Up	CNTN1
RBM46	0.44498415	1.12792669	0.44405144	2.54008116	0.011083	0.043167	Up	RBM46
NEFL	41.5142232	0.64652206	0.25471229	2.53824442	0.011141	0.043317	Up	NEFL
AADACL4	0.26420461	1.15817052	0.45643396	2.53743281	0.011167	0.043398	Up	AADACL4
TNFRSF13B	23.0765672	0.53673319	0.21153928	2.53727439	0.011172	0.043407	Up	TNFRSF13B
CGB7	2.25607133	0.63694488	0.25110455	2.53657243	0.011194	0.043475	Up	CGB7
TACR3	0.79285063	0.94777114	0.37384272	2.53521358	0.011238	0.043594	Up	TACR3
COL25A1	4.64460395	0.51440717	0.20301001	2.53390045	0.01128	0.043709	Up	COL25A1
CDH18	0.81166241	1.07838109	0.42561035	2.53372855	0.011286	0.04372	Up	CDH18
KIAA1549L	155.103741	0.50481036	0.19940141	2.53162876	0.011353	0.043825	Up	KIAA1549L
SAA4	4.32107726	0.71889096	0.28400861	2.53122945	0.011366	0.043855	Up	SAA4
CCDC166	0.78466271	0.78336089	0.30987596	2.52798213	0.011472	0.044153	Up	CCDC166
TRPM8	6.17728425	0.55453263	0.21976647	2.52328129	0.011627	0.044558	Up	TRPM8
C1QTNF8	1.82089033	0.67178754	0.26644064	2.52134035	0.011691	0.044715	Up	C1QTNF8
RALYL	2.04645859	0.91965647	0.36517771	2.51838063	0.01179	0.045002	Up	RALYL
PRPS1L1	0.39645069	1.07279046	0.42656353	2.51496059	0.011905	0.04532	Up	PRPS1L1
GCM2	0.66833113	1.37572588	0.54782372	2.51125649	0.01203	0.045727	Up	GCM2
ELANE	4.37278474	0.57261082	0.22843642	2.50665292	0.012188	0.046173	Up	ELANE
ASCL4	0.49862135	0.8408631	0.33561338	2.50545168	0.01223	0.046249	Up	ASCL4
GALNTL5	0.51526976	1.3246238	0.52884596	2.50474411	0.012254	0.046331	Up	GALNTL5
MT3	62.9877135	0.5669272	0.22637994	2.50431736	0.012269	0.046377	Up	MT3
PRR27	0.86942014	1.13195924	0.45208243	2.503878	0.012284	0.046404	Up	PRR27
PCDHGB1	11.3569929	0.51278812	0.20481339	2.50368451	0.012291	0.046409	Up	PCDHGB1
SIRPD	1.41160772	0.57854911	0.23112493	2.50318778	0.012308	0.046454	Up	SIRPD
C6	15.1903484	0.51950214	0.20764148	2.50191893	0.012352	0.046565	Up	C6
PAX2	4.30701949	0.65517174	0.2618968	2.50164085	0.012362	0.046585	Up	PAX2
PDYN	0.38132762	1.27430917	0.51108309	2.49335027	0.012654	0.047437	Up	PDYN
SEC14L4	24.2991381	0.67659813	0.27182168	2.48912493	0.012806	0.047816	Up	SEC14L4
SULT1C3	20.6567844	0.6944515	0.27916567	2.48759634	0.012861	0.047981	Up	SULT1C3

KRT17	816.0109	0.51091965	0.20564334	2.48449399	0.012974	0.048296	Up	KRT17
OR2D3	0.64337483	2.27692384	0.91669254	2.48384682	0.012997	0.048373	Up	OR2D3
NLGN1	12.1056593	0.6565952	0.26452183	2.48219667	0.013058	0.048534	Up	NLGN1
PHOX2A	4.43452971	0.68307344	0.27533845	2.48085014	0.013107	0.04864	Up	PHOX2A
CLEC4G	9.91896285	0.55013814	0.22181763	2.48013716	0.013133	0.048651	Up	CLEC4G
B3GNT7	2174.71848	0.50382353	0.20330797	2.47812981	0.013207	0.048874	Up	B3GNT7
BCHE	59.5257371	0.6081299	0.24556419	2.47646001	0.013269	0.049014	Up	BCHE
GRIA1	6.06450851	0.56091893	0.22653791	2.47604886	0.013285	0.04906	Up	GRIA1
SLC7A14	9.27971529	0.74614983	0.30136066	2.47593639	0.013289	0.049065	Up	SLC7A14
CACNG6	1.51913886	0.70249802	0.28388971	2.47454557	0.013341	0.049243	Up	CACNG6
ABCB5	7.22546725	0.73574595	0.29752171	2.47291516	0.013402	0.049407	Up	ABCB5
KIR2DL1	1.17719436	0.80214388	0.32436496	2.47296716	0.0134	0.049407	Up	KIR2DL1
SLC22A8	0.45957659	1.26147389	0.51020869	2.4724665	0.013418	0.049448	Up	SLC22A8
