# Supplementary material for: Development of a prognostic risk model for colorectal cancer based on microsatellite stability-associated genes
Source: BMC Cancer. 2025 Oct 1;25:1490. doi: 10.1186/s12885-025-14918-y (PMC12487216; doi:10.1186/s12885-025-14918-y)
Supplement: Supplementary file 11 — Supplementary Material 11. [file 12885_2025_14918_MOESM11_ESM.zip › Table S2.pdf]

GeneSymbol	baseMean	log2FoldChar	lfcSE	stat	pvalue	padj	change	Symbols
CDH3	3190.49975	5.76614607	0.13130025	43.9157264	0	0	Up	CDH3
KRT80	1387.39473	6.67058615	0.1607116	41.5065625	0	0	Up	KRT80
ETV4	3964.78726	5.30112295	0.13170811	40.249024	0	0	Up	ETV4
FOXQ1	1533.06096	6.48725299	0.18168118	35.7067977	3.10E-279	1.38E-275	Up	FOXQ1
ESM1	242.263823	5.5466515	0.16036843	34.5869289	3.97E-262	1.42E-258	Up	ESM1
CLDN1	3318.59798	4.8365493	0.14496548	33.3634543	4.65E-244	1.38E-240	Up	CLDN1
CEMIP	4846.66668	5.2038162	0.16065726	32.390793	3.70E-230	9.43E-227	Up	CEMIP
GRIN2D	1368.31966	5.03652035	0.16051703	31.3768598	4.19E-216	9.34E-213	Up	GRIN2D
AJUBA	828.279846	2.97324727	0.09497124	31.3068164	3.77E-215	7.47E-212	Up	AJUBA
WNT2	309.814807	5.47537718	0.17684409	30.9616075	1.77E-210	3.16E-207	Up	WNT2
MTHFD1L	1693.84209	2.36271656	0.07837615	30.1458617	1.22E-199	1.97E-196	Up	MTHFD1L
NFE2L3	4510.47795	2.66301446	0.09164054	29.0593482	1.17E-185	1.74E-182	Up	NFE2L3
CPNE7	1118.60465	5.83137156	0.20900477	27.900663	2.62E-171	3.60E-168	Up	CPNE7
BEST4	266.475677	-5.80682286	0.21149529	-27.4560384	5.88E-166	7.50E-163	Down	BEST4
SIM2	536.235746	4.52219282	0.16493306	27.4183534	1.66E-165	1.97E-162	Up	SIM2
CST1	979.862637	8.38400445	0.3102596	27.0225465	8.03E-161	8.96E-158	Up	CST1
PDX1	650.19381	4.7420543	0.17584767	26.9668299	3.62E-160	3.80E-157	Up	PDX1
GLTP	1413.23735	-1.59789927	0.05932488	-26.9347259	8.61E-160	8.54E-157	Down	GLTP
TRIB3	2927.86778	3.86480117	0.14354267	26.9244063	1.14E-159	1.07E-156	Up	TRIB3
EPHX4	256.998468	4.43137449	0.16524374	26.8172005	2.04E-158	1.82E-155	Up	EPHX4
CLEC3B	193.72616	-4.24366409	0.16076707	-26.3963515	1.51E-153	1.28E-150	Down	CLEC3B
KLK6	928.160403	9.38571369	0.3560089	26.3637055	3.57E-153	2.90E-150	Up	KLK6
UGP2	4037.62278	-2.13251579	0.08135497	-26.2124843	1.91E-151	1.49E-148	Down	UGP2
SLCO4A1	2625.36967	3.51159027	0.13405165	26.1958004	2.97E-151	2.21E-148	Up	SLCO4A1
COL10A1	871.782163	7.82802538	0.30031703	26.0658725	8.89E-150	6.35E-147	Up	COL10A1
LRP8	791.222794	2.88653334	0.11196672	25.7802785	1.48E-146	1.01E-143	Up	LRP8
INHBA	1383.44676	5.24322387	0.20587578	25.4679004	4.47E-143	2.96E-140	Up	INHBA
COL11A1	1340.0353	6.28304549	0.24717488	25.4194336	1.54E-142	9.80E-140	Up	COL11A1
MMP7	2280.67259	7.0435693	0.2771977	25.4099129	1.96E-142	1.21E-139	Up	MMP7
APPL2	2229.87698	-1.81775343	0.07238081	-25.1137466	3.52E-139	2.09E-136	Down	APPL2
PEX26	2637.58644	-1.72478925	0.0687137	-25.1010959	4.84E-139	2.79E-136	Down	PEX26
PHLPP2	829.991013	-2.58665634	0.10348191	-24.9962168	6.72E-138	3.75E-135	Down	PHLPP2
OTX1	99.9335812	5.69750466	0.22872212	24.9101601	5.77E-137	3.12E-134	Up	OTX1
SPTBN2	1339.46222	3.08350906	0.12455367	24.7564694	2.64E-135	1.39E-132	Up	SPTBN2
MDFI	508.857015	3.65267559	0.14780339	24.7130711	7.74E-135	3.95E-132	Up	MDFI
ENC1	5621.00695	2.01391304	0.08208659	24.534009	6.41E-133	3.18E-130	Up	ENC1
KRT23	2528.699	7.36672661	0.30075465	24.4941399	1.71E-132	8.23E-130	Up	KRT23
SALL4	131.154849	4.99350123	0.20438284	24.4320958	7.80E-132	3.66E-129	Up	SALL4
DDN	114.552367	4.55294115	0.18691679	24.3581177	4.76E-131	2.18E-128	Up	DDN
SLC25A34	82.4621198	-3.91607688	0.16130168	-24.2779675	3.35E-130	1.49E-127	Down	SLC25A34
TRIP13	931.910111	2.22299872	0.09189707	24.1900926	2.83E-129	1.23E-126	Up	TRIP13
GTF2IRD1	2541.60129	2.22782097	0.09248167	24.0893243	3.23E-128	1.37E-125	Up	GTF2IRD1
NOTUM	2825.64982	8.84588794	0.36757181	24.0657409	5.71E-128	2.37E-125	Up	NOTUM
PDCD4	3648.4412	-2.0232107	0.08610223	-23.4977734	4.30E-122	1.74E-119	Down	PDCD4
PLEKHN1	149.856383	3.47887411	0.14852252	23.42321	2.48E-121	9.83E-119	Up	PLEKHN1
CGREF1	938.392171	3.46838596	0.14808106	23.4222115	2.54E-121	9.85E-119	Up	CGREF1
CBX2	545.447744	3.64803014	0.15600229	23.3844655	6.15E-121	2.34E-118	Up	CBX2
KIAA1549	955.998741	2.30445144	0.09878617	23.3276716	2.32E-120	8.64E-118	Up	KIAA1549
FUT1	309.382793	2.88241723	0.12414873	23.2174515	3.03E-119	1.11E-116	Up	FUT1
VWA2	803.417547	4.04885181	0.17591187	23.0163654	3.20E-117	1.14E-114	Up	VWA2
PPM1H	1577.85791	2.28632241	0.09949972	22.9781794	7.71E-117	2.70E-114	Up	PPM1H
SLC51A	282.767019	-4.02882321	0.1756748	-22.9334162	2.16E-116	7.40E-114	Down	SLC51A
TESC	1972.74028	4.64338119	0.20332129	22.8376538	1.94E-115	6.53E-113	Up	TESC
TEX11	25.9751437	-4.11714552	0.18063364	-22.7927954	5.41E-115	1.79E-112	Down	TEX11
ETFDH	731.623764	-1.86525339	0.08190898	-22.7722705	8.64E-115	2.80E-112	Down	ETFDH
RETSAT	4347.7974	-1.89789642	0.08365427	-22.6873815	5.97E-114	1.90E-111	Down	RETSAT
CIPC	1019.81645	-1.33614668	0.05905427	-22.6257418	2.42E-113	7.57E-111	Down	CIPC

SLC7A5	6858.65528	3.07063998	0.13599779	22.5786022	7.03E-113	2.16E-110	Up		SLC7A5
STRA6	461.544211	5.79221147	0.25663473	22.5698657	8.57E-113	2.59E-110	Up		STRA6
LEMD1	136.428833	6.19614139	0.27595347	22.4535734	1.18E-111	3.51E-109	Up		LEMD1
SLC36A1	998.478415	-1.95573801	0.08820387	-22.1729276	6.27E-109	1.83E-106	Down		SLC36A1
DDX31	739.292099	1.1474226	0.05175832	22.168855	6.86E-109	1.98E-106	Up		DDX31
SLC22A5	927.041034	-1.79136318	0.08094575	-22.1304169	1.61E-108	4.56E-106	Down		SLC22A5
PRSS22	868.701384	3.66399332	0.16686921	21.9572756	7.38E-107	2.06E-104	Up		PRSS22
PRKG2	65.7278761	-3.97106257	0.18088792	-21.9531656	8.08E-107	2.22E-104	Down		PRKG2
TEAD4	1021.83588	2.33972682	0.10667957	21.9322857	1.28E-106	3.46E-104	Up		TEAD4
CSRP1	10059.6734	-2.32516991	0.10662073	-21.8078586	1.95E-105	5.20E-103	Down		CSRP1
SLC6A6	4194.70102	2.88840649	0.13300399	21.7166899	1.43E-104	3.74E-102	Up		SLC6A6
EVA1A	318.754062	3.80216381	0.17538278	21.6792313	3.22E-104	8.33E-102	Up		EVA1A
MMP11	4137.40022	4.4608687	0.20606556	21.647813	6.37E-104	1.62E-101	Up		MMP11
SH3TC2	382.536588	3.29380709	0.15253302	21.5940597	2.04E-103	5.13E-101	Up		SH3TC2
ABCG2	308.191055	-4.66682535	0.21616321	-21.5893601	2.26E-103	5.60E-101	Down		ABCG2
TGFBI	32555.2526	3.29972034	0.15327828	21.527645	8.58E-103	2.10E-100	Up		TGFBI
NEBL	3293.25238	2.30900119	0.1073886	21.5013618	1.51E-102	3.65E-100	Up		NEBL
IGFL4	80.6226219	5.63077285	0.26214777	21.4793847	2.43E-102	5.77E-100	Up		IGFL4
RHPN1	995.867603	3.37930933	0.15774555	21.4225328	8.24E-102	1.93E-99	Up		RHPN1
NKD2	940.514475	4.41608456	0.20680713	21.3536379	3.61E-101	8.36E-99	Up		NKD2
CBX8	537.767755	1.73872481	0.08187655	21.2359322	4.45E-100	1.02E-97	Up		CBX8
POU5F1B	288.035543	4.70907088	0.2219525	21.2165708	6.71E-100	1.52E-97	Up		POU5F1B
USP2	178.802932	-3.80557857	0.1804774	-21.0861777	1.07E-98	2.38E-96	Down		USP2
IL6R	588.071036	-2.73804383	0.12989234	-21.0793332	1.23E-98	2.71E-96	Down		IL6R
GLP2R	61.2623588	-3.93506158	0.1869142	-21.0527691	2.16E-98	4.69E-96	Down		GLP2R
CA7	350.955454	-5.67942437	0.26999632	-21.0351914	3.13E-98	6.72E-96	Down		CA7
CNNM2	629.395634	-1.6226114	0.07731648	-20.9866167	8.69E-98	1.85E-95	Down		CNNM2
DPEP1	9401.54338	5.47619153	0.26178534	20.9186337	3.62E-97	7.61E-95	Up		DPEP1
PUS7	1128.90954	1.67959025	0.08038149	20.8952366	5.92E-97	1.23E-94	Up		PUS7
SMPDL3A	881.879111	-2.64139337	0.12660585	-20.8631219	1.16E-96	2.38E-94	Down		SMPDL3A
ORC6	540.52167	1.98865144	0.09539479	20.8465406	1.64E-96	3.32E-94	Up		ORC6
POLR1B	1452.60542	1.20875519	0.05798929	20.8444551	1.71E-96	3.43E-94	Up		POLR1B
TRIM29	3107.56644	4.0344699	0.19382581	20.8149256	3.17E-96	6.29E-94	Up		TRIM29
AUNIP	187.207003	2.1802663	0.10484412	20.7953125	4.77E-96	9.34E-94	Up		AUNIP
DKC1	5137.39026	1.48107397	0.07122292	20.7949066	4.81E-96	9.34E-94	Up		DKC1
ENDOD1	1950.87872	-1.69841842	0.08181386	-20.7595446	1.01E-95	1.93E-93	Down		ENDOD1
CCDC69	909.29651	-3.01674281	0.14563499	-20.714409	2.57E-95	4.88E-93	Down		CCDC69
OSBPL3	1426.52223	1.83630568	0.08919098	20.5884673	3.48E-94	6.54E-92	Up		OSBPL3
SPPL2A	2257.71789	-1.62522516	0.07911301	-20.5430842	8.87E-94	1.65E-91	Down		SPPL2A
C2CD4A	868.779856	4.29792248	0.20940681	20.5242729	1.31E-93	2.40E-91	Up		C2CD4A
MSX2	462.705241	4.66548942	0.22743774	20.5132593	1.64E-93	2.99E-91	Up		MSX2
CLDN2	5379.10196	5.60551981	0.27380054	20.473005	3.75E-93	6.76E-91	Up		CLDN2
FUCA1	3216.9923	-1.7862636	0.0874082	-20.4358805	8.02E-93	1.43E-90	Down		FUCA1
PRDX6	6943.79695	-1.4541006	0.0712757	-20.4010709	1.64E-92	2.89E-90	Down		PRDX6
ACAN	335.889067	3.24387772	0.15942352	20.3475485	4.88E-92	8.54E-90	Up		ACAN
SFTA2	107.430747	7.35183	0.36139146	20.3431202	5.34E-92	9.26E-90	Up		SFTA2
APLN	331.595563	3.24583475	0.15969441	20.3252868	7.68E-92	1.32E-89	Up		APLN
CA2	5107.69578	-4.78480251	0.23547706	-20.3196124	8.63E-92	1.47E-89	Down		CA2
HILPDA	1119.66664	2.35615153	0.11627124	20.2642675	2.66E-91	4.48E-89	Up		HILPDA
FAM107A	156.252525	-3.4850096	0.17256703	-20.1951065	1.08E-90	1.80E-88	Down		FAM107A
MXI1	1572.9698	-1.66562748	0.08248031	-20.1942428	1.10E-90	1.82E-88	Down		MXI1
ATG9B	203.755683	3.57949659	0.1773714	20.1807986	1.44E-90	2.36E-88	Up		ATG9B
CDKN2B	837.686259	-3.179104	0.15790551	-20.1329512	3.80E-90	6.16E-88	Down		CDKN2B
CEP72	370.625391	2.01834158	0.10029651	20.123747	4.57E-90	7.35E-88	Up		CEP72
TMEM132A	1430.98342	2.57899785	0.12820298	20.1165197	5.29E-90	8.43E-88	Up		TMEM132A
ASCL2	4367.51614	4.12673168	0.20517559	20.1131711	5.66E-90	8.94E-88	Up		ASCL2
PKIB	635.627238	-3.94590716	0.19635222	-20.0960657	7.99E-90	1.25E-87	Down		PKIB
FJX1	179.641246	2.90994205	0.14486707	20.0869813	9.59E-90	1.49E-87	Up		FJX1

C2orf88	564.930641	-3.5965251	0.17920842	-20.0689515	1.38E-89	2.12E-87	Down	C2orf88
MAPK15	242.504676	4.28662272	0.21439103	19.9944124	6.16E-89	9.40E-87	Up	MAPK15
XRCC2	310.049777	2.10842787	0.10581846	19.924953	2.47E-88	3.74E-86	Up	XRCC2
GCNT2	113.802741	-3.71286213	0.18637357	-19.9216128	2.64E-88	3.96E-86	Down	GCNT2
GRHL3	186.497435	3.97503508	0.19954765	19.92023	2.72E-88	4.04E-86	Up	GRHL3
PRR7	424.917099	3.69485148	0.18556872	19.9109604	3.27E-88	4.82E-86	Up	PRR7
EGFL6	122.571431	3.48240557	0.17501545	19.8977036	4.26E-88	6.23E-86	Up	EGFL6
HJURP	865.849264	1.68639415	0.08476919	19.8939504	4.59E-88	6.66E-86	Up	HJURP
SLC39A10	1359.84257	1.9271077	0.09688861	19.8899294	4.97E-88	7.16E-86	Up	SLC39A10
SULT2B1	924.427922	3.49100303	0.17552118	19.8893544	5.03E-88	7.18E-86	Up	SULT2B1
CLDN23	855.771104	-2.92323248	0.14749748	-19.8188645	2.05E-87	2.90E-85	Down	CLDN23
CDC25B	5657.51802	2.23419716	0.11275976	19.8137803	2.26E-87	3.18E-85	Up	CDC25B
TFAP4	1016.82881	1.47980178	0.07473145	19.801593	2.88E-87	4.02E-85	Up	TFAP4
PTGS1	825.530728	-2.93053196	0.14808027	-19.7901579	3.62E-87	5.01E-85	Down	PTGS1
ANLN	1902.05899	2.03353418	0.10279778	19.7818885	4.26E-87	5.85E-85	Up	ANLN
MCIDAS	35.6960171	4.01429718	0.20318598	19.7567622	7.02E-87	9.56E-85	Up	MCIDAS
KLK7	181.649844	8.32751393	0.42248815	19.7106452	1.75E-86	2.36E-84	Up	KLK7
PDK4	1010.09781	-3.32731174	0.16920792	-19.6640426	4.38E-86	5.88E-84	Down	PDK4
CASP7	2511.65759	-1.69593181	0.08627036	-19.6583376	4.91E-86	6.53E-84	Down	CASP7
SPATA12	38.4237135	3.19977401	0.16282645	19.6514386	5.62E-86	7.43E-84	Up	SPATA12
CPT2	1235.16154	-1.42707168	0.07272671	-19.6223864	9.96E-86	1.31E-83	Down	CPT2
FAM47E-STE	134.108883	-1.84833332	0.09427954	-19.6048199	1.41E-85	1.83E-83	Down	FAM47E-STBD1
CTHRC1	1027.37956	3.76853282	0.19224067	19.6032022	1.45E-85	1.88E-83	Up	CTHRC1
FABP6	339.533579	4.65035029	0.23726152	19.6001031	1.54E-85	1.98E-83	Up	FABP6
TTC26	319.576629	1.65493021	0.08447351	19.5911156	1.84E-85	2.35E-83	Up	TTC26
CCNO	343.318903	3.07687323	0.15725709	19.5658786	3.02E-85	3.82E-83	Up	CCNO
ASPA	32.6399076	-3.93498076	0.20127709	-19.550068	4.12E-85	5.18E-83	Down	ASPA
SPIB	221.284018	-4.71309785	0.24156315	-19.5108315	8.88E-85	1.11E-82	Down	SPIB
MIER3	1372.04116	-1.9506603	0.10004193	-19.4984281	1.13E-84	1.40E-82	Down	MIER3
NAAA	1271.81494	-2.05918361	0.10561134	-19.4977511	1.15E-84	1.41E-82	Down	NAAA
SKA3	895.001255	2.09030864	0.10725792	19.4886173	1.37E-84	1.68E-82	Up	SKA3
CST2	63.5061722	6.36934544	0.32692702	19.4824689	1.55E-84	1.88E-82	Up	CST2
ZNF575	139.023324	-1.99035404	0.10216718	-19.4813452	1.58E-84	1.91E-82	Down	ZNF575
TPX2	4456.75006	1.87646564	0.09635958	19.4735771	1.84E-84	2.20E-82	Up	TPX2
MRGBP	1440.86868	1.65773565	0.08526013	19.4432695	3.32E-84	3.95E-82	Up	MRGBP
TP53INP2	1930.32267	-2.5219067	0.12974209	-19.4378462	3.69E-84	4.37E-82	Down	TP53INP2
PLCD1	778.943529	-2.16332426	0.11143307	-19.4136648	5.92E-84	6.95E-82	Down	PLCD1
SLC4A11	298.742114	4.12380171	0.2125577	19.4008575	7.59E-84	8.85E-82	Up	SLC4A11
RIPK2	934.917076	1.54021752	0.07954051	19.3639388	1.56E-83	1.80E-81	Up	RIPK2
HTR1D	307.682996	4.13935569	0.21387155	19.3544004	1.87E-83	2.15E-81	Up	HTR1D
PYGM	74.9750537	-3.98589208	0.20602227	-19.3468995	2.16E-83	2.48E-81	Down	PYGM
SGK1	1908.83109	-2.77287625	0.14333511	-19.3454083	2.23E-83	2.53E-81	Down	SGK1
KLHL35	234.486904	3.92364939	0.20288085	19.3396732	2.49E-83	2.81E-81	Up	KLHL35
SLC51B	340.607111	-4.00122559	0.20706885	-19.3231647	3.43E-83	3.85E-81	Down	SLC51B
AHCYL2	3939.76343	-2.69524674	0.13951971	-19.3180361	3.79E-83	4.20E-81	Down	AHCYL2
SULT1A2	98.0096874	-3.74624273	0.19392159	-19.3183373	3.77E-83	4.20E-81	Down	SULT1A2
ENTPD5	2611.85358	-2.5139038	0.1301903	-19.3094549	4.47E-83	4.93E-81	Down	ENTPD5
SLC25A23	2754.08856	-1.72514383	0.08943263	-19.2898693	6.53E-83	7.15E-81	Down	SLC25A23
URB2	888.97791	1.24971794	0.06480935	19.2829897	7.46E-83	8.12E-81	Up	URB2
GAD1	116.904449	5.30152377	0.27505785	19.2742139	8.84E-83	9.56E-81	Up	GAD1
RUVBL1	2454.08801	1.37191519	0.07119986	19.268511	9.87E-83	1.06E-80	Up	RUVBL1
GTPBP4	2395.06055	1.08242323	0.05620745	19.2576462	1.22E-82	1.30E-80	Up	GTPBP4
UBE2C	3033.52754	2.13012748	0.11064337	19.2521924	1.35E-82	1.44E-80	Up	UBE2C
ZC3HAV1L	279.286452	2.1743176	0.11299144	19.2432056	1.61E-82	1.70E-80	Up	ZC3HAV1L
TCN1	789.187596	7.19209406	0.37428297	19.2156594	2.74E-82	2.87E-80	Up	TCN1
XPO5	2689.47234	1.272887	0.06629989	19.1989301	3.78E-82	3.94E-80	Up	XPO5
MPZ	59.099316	-2.89346361	0.1509908	-19.1631786	7.51E-82	7.80E-80	Down	MPZ
PLCE1	1065.66621	-2.03097172	0.10599296	-19.1613825	7.78E-82	8.02E-80	Down	PLCE1

TMEM253	60.8983082	-2.97976086	0.15554306	-19.1571438	8.44E-82	8.65E-80	Down	TMEM253
COMP	742.524907	6.00541154	0.31388101	19.1327648	1.35E-81	1.37E-79	Up	COMP
KLK8	92.9173859	8.93961308	0.46844296	19.0836746	3.45E-81	3.50E-79	Up	KLK8
ENPP6	14.9072549	-3.4169459	0.17905684	-19.0830236	3.49E-81	3.52E-79	Down	ENPP6
POPDC2	140.237426	-3.591234	0.18820898	-19.0810977	3.63E-81	3.64E-79	Down	POPDC2
PRR36	315.040084	4.07493237	0.21357145	19.0799486	3.71E-81	3.70E-79	Up	PRR36
S100A11	14616.4635	1.90693105	0.1001388	19.0428786	7.53E-81	7.46E-79	Up	S100A11
TNS4	4487.79443	3.41017788	0.17956556	18.991269	2.01E-80	1.99E-78	Up	TNS4
ADAMTS12	743.446971	3.36962145	0.17744639	18.9895184	2.08E-80	2.04E-78	Up	ADAMTS12
MEP1B	129.163663	-5.54099619	0.29180701	-18.9885642	2.12E-80	2.07E-78	Down	MEP1B
LYVE1	221.092009	-4.20117111	0.22153504	-18.9639122	3.39E-80	3.29E-78	Down	LYVE1
LRRC1	1473.83601	-1.19401636	0.06297039	-18.9615528	3.55E-80	3.42E-78	Down	LRRC1
TAT	11.0199551	-3.728049	0.196617	-18.9609702	3.58E-80	3.44E-78	Down	TAT
ADAMDEC1	1476.47356	-3.63113356	0.19155652	-18.9559383	3.94E-80	3.76E-78	Down	ADAMDEC1
SCARA5	361.600277	-4.6955293	0.24791271	-18.9402522	5.31E-80	5.04E-78	Down	SCARA5
CITED2	1034.77117	-1.74158243	0.09200874	-18.9284446	6.65E-80	6.28E-78	Down	CITED2
SMKR1	62.9862728	3.99886201	0.2113738	18.9184377	8.04E-80	7.55E-78	Up	SMKR1
TMCC3	1018.32525	-2.38688213	0.12652083	-18.8655272	2.19E-79	2.05E-77	Down	TMCC3
FEZF1	83.3680566	8.61289198	0.45701844	18.8458305	3.18E-79	2.96E-77	Up	FEZF1
TLX1	127.97808	4.12892415	0.21927169	18.8301742	4.27E-79	3.95E-77	Up	TLX1
SOX9	8398.66758	1.92266852	0.10211204	18.8290097	4.37E-79	4.02E-77	Up	SOX9
CSE1L	6240.8431	1.56904774	0.08335032	18.8247355	4.74E-79	4.33E-77	Up	CSE1L
GNA11	4966.19255	-1.67672154	0.08921383	-18.7944138	8.39E-79	7.64E-77	Down	GNA11
GFRA2	41.5008882	-3.60624968	0.19190235	-18.7921077	8.76E-79	7.90E-77	Down	GFRA2
PERM1	53.8152658	3.15640768	0.1679649	18.7920667	8.77E-79	7.90E-77	Up	PERM1
NOP2	2310.57555	1.43965275	0.07664736	18.7828099	1.04E-78	9.36E-77	Up	NOP2
UGDH	2752.00068	-1.82718508	0.09743112	-18.7536085	1.81E-78	1.61E-76	Down	UGDH
PSAT1	2054.84085	2.73087899	0.14565173	18.7493751	1.96E-78	1.74E-76	Up	PSAT1
TEF	684.579074	-1.78970897	0.09559181	-18.7224099	3.25E-78	2.87E-76	Down	TEF
EPM2A	143.713251	-1.76195312	0.09413248	-18.7178015	3.54E-78	3.12E-76	Down	EPM2A
SMOX	874.966434	2.34776193	0.12566638	18.6824981	6.87E-78	5.98E-76	Up	SMOX
PLAC9	81.9819148	-2.57057939	0.13759163	-18.6826732	6.85E-78	5.98E-76	Down	PLAC9
TNXB	522.584104	-4.03548366	0.21609895	-18.67424	8.02E-78	6.95E-76	Down	TNXB
KAT2A	3094.26623	1.59631488	0.08550937	18.6683034	8.97E-78	7.73E-76	Up	KAT2A
PABPC1L	976.174934	3.17208158	0.16993357	18.6665981	9.26E-78	7.94E-76	Up	PABPC1L
BYSL	1169.43936	1.56075485	0.08372253	18.6419927	1.47E-77	1.25E-75	Up	BYSL
TROAP	898.481688	1.95244811	0.10474258	18.640444	1.51E-77	1.28E-75	Up	TROAP
CA9	1672.13554	5.52205542	0.29651166	18.6234007	2.08E-77	1.76E-75	Up	CA9
KLK10	1661.49907	5.05249714	0.27198781	18.5761899	5.01E-77	4.22E-75	Up	KLK10
NAALADL1	257.586016	-3.21515945	0.1734156	-18.5401972	9.79E-77	8.20E-75	Down	NAALADL1
MET	5525.02427	1.79802695	0.09700921	18.5346	1.09E-76	9.06E-75	Up	MET
TOMM34	3250.65725	1.83380829	0.09904546	18.5148144	1.57E-76	1.30E-74	Up	TOMM34
HAGHL	409.242448	3.62383225	0.19608278	18.4811345	2.93E-76	2.42E-74	Up	HAGHL
DHDDS	1356.37326	-1.2042823	0.0652039	-18.4694833	3.64E-76	2.99E-74	Down	DHDDS
SVIL	2990.32602	-2.12693778	0.11516782	-18.4681601	3.73E-76	3.05E-74	Down	SVIL
RNF183	178.685791	3.38240799	0.18317145	18.4658037	3.89E-76	3.17E-74	Up	RNF183
DBNDD1	833.833328	2.48098082	0.13446646	18.4505547	5.16E-76	4.19E-74	Up	DBNDD1
ZZEF1	2572.99456	-1.70946722	0.09272646	-18.4355922	6.81E-76	5.50E-74	Down	ZZEF1
IQGAP3	2360.38028	1.9403406	0.10537171	18.4142467	1.01E-75	8.12E-74	Up	IQGAP3
GRIK3	45.9956553	-3.74741219	0.20391783	-18.3770694	2.01E-75	1.60E-73	Down	GRIK3
CCND1	7841.62674	1.65876227	0.09027794	18.3739486	2.12E-75	1.69E-73	Up	CCND1
MMP3	1630.79171	4.84166116	0.2635683	18.3696639	2.30E-75	1.82E-73	Up	MMP3
CCDC78	122.787994	3.65579012	0.19909617	18.361931	2.65E-75	2.09E-73	Up	CCDC78
CCNYL1	838.751853	-1.41542426	0.07710266	-18.3576576	2.87E-75	2.25E-73	Down	CCNYL1
RFC3	1256.38344	1.80623445	0.09839467	18.3570359	2.90E-75	2.27E-73	Up	RFC3
CNNM4	3058.35399	-1.6098759	0.08770821	-18.3549061	3.02E-75	2.35E-73	Down	CNNM4
FHL1	1463.39168	-3.39746897	0.18518835	-18.3460191	3.55E-75	2.76E-73	Down	FHL1
MS4A10	26.2673071	-7.38669379	0.40272813	-18.3416385	3.85E-75	2.97E-73	Down	MS4A10

GPD1L	2062.01472	-1.44248943	0.07864842	-18.3409844	3.90E-75	3.00E-73	Down	GPD1L
ANKRD13B	628.626198	2.42266947	0.13212122	18.3367169	4.21E-75	3.23E-73	Up	ANKRD13B
PTPN21	584.077818	-1.49916923	0.08179749	-18.3278156	4.96E-75	3.79E-73	Down	PTPN21
FAM89A	394.119259	1.77524152	0.09686368	18.3272155	5.02E-75	3.81E-73	Up	FAM89A
ATP11A	3271.27933	2.23198236	0.12179929	18.3250851	5.22E-75	3.95E-73	Up	ATP11A
SNTB1	2185.45591	2.26617962	0.12373288	18.3150966	6.27E-75	4.72E-73	Up	SNTB1
CCT6A	9635.19105	1.14251072	0.06247592	18.287218	1.05E-74	7.84E-73	Up	CCT6A
PDCD2L	382.787759	1.52665441	0.08350929	18.2812534	1.17E-74	8.71E-73	Up	PDCD2L
CDC14A	248.66342	-1.93178192	0.10570758	-18.2747728	1.31E-74	9.77E-73	Down	CDC14A
TMEM100	52.365577	-3.86982569	0.21189181	-18.2632149	1.62E-74	1.20E-72	Down	TMEM100
MLXIPL	1542.89605	3.26934406	0.17916082	18.2480969	2.14E-74	1.58E-72	Up	MLXIPL
AQP8	3084.60946	-6.86512517	0.3766711	-18.2257817	3.22E-74	2.37E-72	Down	AQP8
PACSIN2	3403.52339	-1.14907294	0.06316029	-18.1929658	5.87E-74	4.29E-72	Down	PACSIN2
QSOX2	1133.09938	1.28268616	0.07052076	18.1887741	6.33E-74	4.61E-72	Up	QSOX2
MEX3A	555.5928	2.90175805	0.15954332	18.1879003	6.44E-74	4.67E-72	Up	MEX3A
S100A2	321.091886	4.08430839	0.22477065	18.1710041	8.76E-74	6.33E-72	Up	S100A2
TNFRSF12A	1939.40936	2.24165931	0.12341128	18.1641358	9.93E-74	7.14E-72	Up	TNFRSF12A
GABRD	52.9072261	2.9355283	0.16177135	18.1461564	1.38E-73	9.87E-72	Up	GABRD
ACOX1	3904.54779	-1.49626769	0.0824847	-18.1399411	1.54E-73	1.10E-71	Down	ACOX1
CMTM5	4.43227868	-4.79480894	0.26442517	-18.1329522	1.75E-73	1.24E-71	Down	CMTM5
CEL	1384.40295	6.13223788	0.33844613	18.1188003	2.26E-73	1.60E-71	Up	CEL
TDP2	3695.30372	-1.88638088	0.1041344	-18.1148671	2.43E-73	1.72E-71	Down	TDP2
SOX4	5433.78526	1.72116623	0.09509255	18.0999064	3.19E-73	2.24E-71	Up	SOX4
CELSR3	793.444612	2.75633619	0.15273165	18.0469221	8.34E-73	5.84E-71	Up	CELSR3
PLP1	108.912751	-5.5400353	0.30741734	-18.0212192	1.33E-72	9.26E-71	Down	PLP1
AP3S2	875.307696	-1.16533388	0.0646679	-18.0202825	1.35E-72	9.38E-71	Down	AP3S2
TGIF2	1406.11799	1.89689636	0.10540315	17.9965815	2.07E-72	1.43E-70	Up	TGIF2
ADD1	6164.75281	-0.83193253	0.04625663	-17.9851518	2.55E-72	1.75E-70	Down	ADD1
RXRG	10.0120664	-3.98842013	0.22195122	-17.969805	3.36E-72	2.31E-70	Down	RXRG
PDE2A	160.070889	-2.7083412	0.15088556	-17.9496386	4.83E-72	3.30E-70	Down	PDE2A
STC2	953.070882	3.55928017	0.19853127	17.9280582	7.12E-72	4.85E-70	Up	STC2
GSTM5	57.008835	-2.99783817	0.16725734	-17.923507	7.73E-72	5.25E-70	Down	GSTM5
CBX7	720.984132	-1.8437922	0.10290703	-17.917067	8.68E-72	5.87E-70	Down	CBX7
DKK4	108.612744	7.72072751	0.4309534	17.9154578	8.93E-72	6.02E-70	Up	DKK4
DSCC1	504.793764	1.8673812	0.10440539	17.8858696	1.52E-71	1.02E-69	Up	DSCC1
BTBD16	43.229073	3.54513015	0.19829486	17.8780739	1.75E-71	1.17E-69	Up	BTBD16
KIF18B	802.807623	1.82538369	0.10220642	17.859776	2.43E-71	1.62E-69	Up	KIF18B
HIGD1A	2271.39731	-1.8711686	0.10483369	-17.8489243	2.95E-71	1.95E-69	Down	HIGD1A
FAM151A	59.151814	-2.33216543	0.13067009	-17.8477368	3.01E-71	1.99E-69	Down	FAM151A
NKRF	611.756364	1.41797774	0.07945821	17.8455784	3.13E-71	2.06E-69	Up	NKRF
HPGDS	37.7187602	-3.12810327	0.17530495	-17.8437822	3.23E-71	2.12E-69	Down	HPGDS
MYOM1	116.431937	-3.32064435	0.18610995	-17.842379	3.31E-71	2.17E-69	Down	MYOM1
TRPM6	549.820652	-4.11742773	0.23085839	-17.8352962	3.76E-71	2.45E-69	Down	TRPM6
SPECC1L	927.751235	-1.06200038	0.05959355	-17.8207259	4.88E-71	3.17E-69	Down	SPECC1L
KRT17	862.216192	4.99233115	0.28026719	17.8127565	5.63E-71	3.64E-69	Up	KRT17
IL11	166.666059	4.6665884	0.26209524	17.804934	6.47E-71	4.17E-69	Up	IL11
RBFOX3	75.5920223	-4.84388244	0.27222914	-17.7934018	7.95E-71	5.10E-69	Down	RBFOX3
TMEM37	806.297225	-2.90574145	0.16331628	-17.7921114	8.14E-71	5.20E-69	Down	TMEM37
KRT6B	433.27246	5.918598	0.33293233	17.7771803	1.06E-70	6.77E-69	Up	KRT6B
RNF185	913.979275	-0.86001399	0.04838034	-17.7761051	1.08E-70	6.87E-69	Down	RNF185
DHRS7C	2.05456379	-5.32602459	0.29976029	-17.7676124	1.26E-70	7.97E-69	Down	DHRS7C
RDH5	81.4683275	-2.24898901	0.1265891	-17.7660559	1.29E-70	8.17E-69	Down	RDH5
FXYD5	4091.37347	2.13146136	0.12001719	17.7596346	1.45E-70	9.12E-69	Up	FXYD5
MYLK	5862.51568	-3.4538178	0.19450753	-17.7567303	1.53E-70	9.57E-69	Down	MYLK
METTL7A	1962.94972	-2.47132922	0.1393229	-17.7381408	2.13E-70	1.33E-68	Down	METTL7A
TG	356.221085	4.11349911	0.23234401	17.7043475	3.88E-70	2.41E-68	Up	TG
ABHD3	1311.84036	-1.92709699	0.10888475	-17.6985024	4.31E-70	2.67E-68	Down	ABHD3
LRRC8E	37.3114399	2.43034667	0.13734504	17.6951911	4.57E-70	2.82E-68	Up	LRRC8E

CFL2	528.156043	-2.73197517	0.154431	-17.6905877	4.96E-70	3.05E-68	Down	CFL2
WDR43	2484.04575	1.1646704	0.06584994	17.6867348	5.31E-70	3.25E-68	Up	WDR43
PAQR4	1126.84867	1.85023498	0.10461334	17.6864152	5.34E-70	3.26E-68	Up	PAQR4
TACSTD2	1536.94438	5.01431106	0.2838257	17.6668676	7.55E-70	4.60E-68	Up	TACSTD2
ASB8	873.532668	-0.78137572	0.0442377	-17.6631197	8.07E-70	4.90E-68	Down	ASB8
KLC3	35.5466642	5.91256929	0.33497507	17.6507742	1.00E-69	6.07E-68	Up	KLC3
DPP6	48.7047494	-5.16993516	0.29304303	-17.6422387	1.17E-69	7.04E-68	Down	DPP6
PPAT	867.383663	1.53116141	0.08682029	17.635986	1.30E-69	7.84E-68	Up	PPAT
MACC1	2387.10352	2.30464694	0.13077803	17.6225852	1.65E-69	9.90E-68	Up	MACC1
COL7A1	1293.69443	3.26700931	0.18549583	17.6123062	1.98E-69	1.18E-67	Up	COL7A1
RAP1A	2121.11627	-1.28961745	0.07326081	-17.6031013	2.33E-69	1.39E-67	Down	RAP1A
GDF15	6534.76139	3.00071791	0.17055883	17.5934475	2.77E-69	1.64E-67	Up	GDF15
FBLIM1	3186.81743	-1.27365209	0.07256163	-17.5526947	5.67E-69	3.35E-67	Down	FBLIM1
RMDN2	199.94182	-1.92458871	0.1097158	-17.5415819	6.90E-69	4.05E-67	Down	RMDN2
ATP6V1G2	14.2603916	-2.48332163	0.14156667	-17.541711	6.88E-69	4.05E-67	Down	ATP6V1G2
SLCO1B3	138.139325	6.53127764	0.37284312	17.5174952	1.05E-68	6.16E-67	Up	SLCO1B3
RTKN	2712.43303	1.24455887	0.0711153	17.5005783	1.42E-68	8.27E-67	Up	RTKN
GUCA2A	2028.62336	-5.07209255	0.28989491	-17.4963146	1.53E-68	8.88E-67	Down	GUCA2A
CYP27B1	140.23326	1.96084809	0.11215037	17.4840988	1.89E-68	1.10E-66	Up	CYP27B1
MAMDC2	114.884089	-4.74863984	0.27164816	-17.4808466	2.00E-68	1.16E-66	Down	MAMDC2
CMSS1	935.864822	1.43572563	0.08218509	17.4694171	2.45E-68	1.41E-66	Up	CMSS1
NR3C2	958.904118	-2.73330465	0.15664726	-17.448787	3.52E-68	2.02E-66	Down	NR3C2
CBX4	1422.22698	1.6120418	0.0923902	17.4481908	3.55E-68	2.03E-66	Up	CBX4
SEC14L2	264.924271	2.10277456	0.12054819	17.4434347	3.86E-68	2.20E-66	Up	SEC14L2
METTL1	788.114863	1.38180709	0.07924149	17.4379251	4.25E-68	2.42E-66	Up	METTL1
TRANK1	1296.32978	-2.09246076	0.12002591	-17.4334088	4.60E-68	2.61E-66	Down	TRANK1
NKX2-3	166.988203	-2.81368769	0.16144141	-17.4285374	5.01E-68	2.83E-66	Down	NKX2-3
MYL9	10213.182	-3.29588033	0.18920917	-17.4192419	5.90E-68	3.32E-66	Down	MYL9
AZGP1	2628.27628	3.25773195	0.18713853	17.4081308	7.16E-68	4.02E-66	Up	AZGP1
SCGN	63.1472392	-4.89319696	0.28119856	-17.4012166	8.08E-68	4.52E-66	Down	SCGN
SEMA6D	357.773478	-3.00313894	0.17276665	-17.3826308	1.12E-67	6.23E-66	Down	SEMA6D
TMEM220	144.430593	-2.94483372	0.16946541	-17.377196	1.23E-67	6.83E-66	Down	TMEM220
TCF7	1915.31776	1.9850548	0.11426141	17.3729237	1.32E-67	7.33E-66	Up	TCF7
NPFFR1	140.090283	3.36928166	0.19395468	17.3714894	1.36E-67	7.50E-66	Up	NPFFR1
ARNTL2	903.933556	2.22911611	0.12832486	17.3708824	1.37E-67	7.55E-66	Up	ARNTL2
HADHB	3806.58257	-0.99149623	0.05707923	-17.3705246	1.38E-67	7.58E-66	Down	HADHB
SLC26A2	8251.36395	-4.05164975	0.23337763	-17.3609172	1.63E-67	8.93E-66	Down	SLC26A2
STK31	116.457923	3.76072013	0.21670899	17.3537802	1.85E-67	1.01E-65	Up	STK31
ADAM12	768.761435	4.06411789	0.23436911	17.3406724	2.32E-67	1.26E-65	Up	ADAM12
MSX1	252.003075	3.40722445	0.19658832	17.3317747	2.71E-67	1.47E-65	Up	MSX1
HSPB8	560.077702	-3.77345633	0.21787376	-17.3194618	3.35E-67	1.81E-65	Down	HSPB8
ULBP1	30.088158	3.86960362	0.22345576	17.3170907	3.50E-67	1.88E-65	Up	ULBP1
PTN	119.723781	-2.77598112	0.1603052	-17.3168497	3.51E-67	1.89E-65	Down	PTN
RBM28	1350.28835	1.12747748	0.06521474	17.2886901	5.72E-67	3.07E-65	Up	RBM28
MYC	9948.68059	1.95719961	0.1132653	17.2797812	6.68E-67	3.57E-65	Up	MYC
FBXO41	733.453371	2.05261237	0.11879233	17.278998	6.77E-67	3.61E-65	Up	FBXO41
LITAF	5443.35393	-1.24941586	0.07231106	-17.2783509	6.85E-67	3.64E-65	Down	LITAF
ULBP2	65.6807663	4.15661819	0.24057975	17.2775063	6.95E-67	3.68E-65	Up	ULBP2
RNF43	9485.90501	2.32456096	0.13458183	17.2724727	7.58E-67	4.00E-65	Up	RNF43
SERPINA4	48.9516075	6.88448231	0.39946768	17.2341408	1.47E-66	7.75E-65	Up	SERPINA4
TTLL4	1189.98114	1.17205227	0.06800913	17.2337478	1.48E-66	7.78E-65	Up	TTLL4
MMP1	3828.73921	4.54536916	0.26409622	17.2110347	2.19E-66	1.15E-64	Up	MMP1
OTOP2	255.934253	-8.06277252	0.46858801	-17.2065277	2.37E-66	1.24E-64	Down	OTOP2
DPT	568.214517	-4.13802394	0.2405286	-17.2038748	2.48E-66	1.29E-64	Down	DPT
GRAMD1A	2967.40299	1.62530577	0.09452086	17.195207	2.88E-66	1.50E-64	Up	GRAMD1A
EZH2	1225.26153	1.28139514	0.0746203	17.1722062	4.29E-66	2.22E-64	Up	EZH2
FBXL22	72.9104019	-2.45456006	0.14293986	-17.1719775	4.31E-66	2.22E-64	Down	FBXL22
KLF4	3474.82394	-2.47606006	0.1442378	-17.1665135	4.73E-66	2.43E-64	Down	KLF4

PSD	324.459247	-3.42353637	0.19946273	-17.1637899	4.96E-66	2.54E-64	Down	PSD
OXTR	78.919325	2.80454551	0.16340272	17.1633951	4.99E-66	2.55E-64	Up	OXTR
EME1	154.423968	1.70986515	0.09963831	17.1607197	5.23E-66	2.66E-64	Up	EME1
VSNL1	1011.98455	2.84127129	0.16561215	17.1561766	5.65E-66	2.87E-64	Up	VSNL1
ZMYND19	980.408934	1.44416866	0.08419841	17.1519716	6.08E-66	3.08E-64	Up	ZMYND19
WNT3	123.435615	2.43200019	0.14179547	17.1514655	6.13E-66	3.10E-64	Up	WNT3
BMP3	223.334547	-5.21477889	0.3041773	-17.1438791	6.98E-66	3.52E-64	Down	BMP3
ATP8B1	6456.08785	-1.68071326	0.09810136	-17.1324162	8.50E-66	4.26E-64	Down	ATP8B1
C1orf115	1229.96994	-2.35098755	0.1372233	-17.1325687	8.48E-66	4.26E-64	Down	C1orf115
RRP12	2118.39144	1.38429225	0.08081208	17.1297679	8.90E-66	4.45E-64	Up	RRP12
XPOT	3808.11261	1.33196031	0.07776384	17.128273	9.13E-66	4.55E-64	Up	XPOT
SCD	17409.2915	2.30093321	0.13436139	17.1249581	9.67E-66	4.81E-64	Up	SCD
TMEM41A	1410.94236	1.04442605	0.06104679	17.1086144	1.28E-65	6.35E-64	Up	TMEM41A
TMEM127	3939.13188	-0.89897545	0.05254583	-17.1084082	1.28E-65	6.35E-64	Down	TMEM127
ABCA8	167.360629	-4.44981151	0.26027865	-17.0963371	1.58E-65	7.79E-64	Down	ABCA8
CBFB	2181.74781	1.3069672	0.07650127	17.0842553	1.94E-65	9.56E-64	Up	CBFB
MCM10	459.932733	1.69452081	0.09921864	17.0786537	2.14E-65	1.05E-63	Up	MCM10
DUSP14	527.540779	1.63711831	0.0959873	17.055572	3.18E-65	1.55E-63	Up	DUSP14
DDIAS	331.441286	1.90188179	0.11157126	17.0463408	3.72E-65	1.81E-63	Up	DDIAS
NCAPG2	1440.54294	1.29025792	0.07575058	17.0329772	4.68E-65	2.27E-63	Up	NCAPG2
ASCL5	55.919369	4.11792878	0.24193132	17.0210651	5.73E-65	2.78E-63	Up	ASCL5
DCUN1D5	949.500682	1.39811274	0.08217273	17.014316	6.43E-65	3.11E-63	Up	DCUN1D5
RBM47	5530.13548	-1.26335241	0.07431246	-17.0005473	8.14E-65	3.92E-63	Down	RBM47
MAL	31.2512633	-3.64413151	0.21439089	-16.9976045	8.55E-65	4.11E-63	Down	MAL
TESK2	272.843488	-1.37480795	0.08093875	-16.9857809	1.05E-64	5.02E-63	Down	TESK2
TMEM72	102.91768	-4.2600405	0.25087698	-16.9805958	1.14E-64	5.47E-63	Down	TMEM72
KIF20A	888.70724	1.52539753	0.08985585	16.9760523	1.24E-64	5.89E-63	Up	KIF20A
ADHFE1	27.1003934	-2.77963017	0.16397627	-16.9514173	1.88E-64	8.94E-63	Down	ADHFE1
SAPCD2	2623.14358	2.03726007	0.12026788	16.9393534	2.31E-64	1.09E-62	Up	SAPCD2
B3GNTL1	259.679883	1.56837012	0.09267856	16.9226857	3.06E-64	1.45E-62	Up	B3GNTL1
DDX55	825.638612	1.08817127	0.06431307	16.9199078	3.21E-64	1.52E-62	Up	DDX55
SLC17A7	14.9805341	-2.97368602	0.17604082	-16.8920255	5.15E-64	2.43E-62	Down	SLC17A7
DDX56	3260.26301	1.0963054	0.0649728	16.8732974	7.07E-64	3.32E-62	Up	DDX56
ANK3	858.850288	-1.72974981	0.10252163	-16.872047	7.22E-64	3.38E-62	Down	ANK3
NPTX1	125.21894	-5.10867341	0.3027928	-16.871846	7.25E-64	3.39E-62	Down	NPTX1
LDB3	108.769172	-4.14147576	0.24555856	-16.8655317	8.07E-64	3.76E-62	Down	LDB3
SLC4A4	1154.90438	-4.60013702	0.27290758	-16.8560254	9.48E-64	4.40E-62	Down	SLC4A4
SP5	501.710713	3.71710419	0.22062499	16.8480647	1.08E-63	5.02E-62	Up	SP5
RIOK3	2664.88503	-1.55141032	0.09212034	-16.8411259	1.22E-63	5.61E-62	Down	RIOK3
STIM1	1921.57107	-1.06038453	0.06296366	-16.8412148	1.22E-63	5.61E-62	Down	STIM1
SH3BGR	79.8372644	-2.13236003	0.12661535	-16.8412442	1.22E-63	5.61E-62	Down	SH3BGR
SLC20A1	2322.55779	-1.47691191	0.08782009	-16.8174728	1.82E-63	8.34E-62	Down	SLC20A1
PYCR1	4338.88588	1.79772398	0.10691173	16.815031	1.89E-63	8.67E-62	Up	PYCR1
GRIN2B	259.611383	3.34815497	0.1991258	16.81427	1.92E-63	8.76E-62	Up	GRIN2B
ATP1A2	183.608676	-5.03530352	0.29977452	-16.7969699	2.57E-63	1.17E-61	Down	ATP1A2
FNBP1	1331.43254	-1.92153429	0.11440768	-16.7955002	2.63E-63	1.20E-61	Down	FNBP1
NEGR1	203.442973	-3.08326972	0.18374649	-16.7800198	3.42E-63	1.55E-61	Down	NEGR1
CHP1	5715.44814	-1.3997572	0.08351196	-16.7611589	4.69E-63	2.12E-61	Down	CHP1
ECT2	2986.10214	1.76745319	0.10550462	16.7523774	5.44E-63	2.45E-61	Up	ECT2
NAT10	3209.83329	1.09254653	0.06524507	16.7452735	6.13E-63	2.76E-61	Up	NAT10
ANAPC7	1843.3477	0.93243465	0.05570104	16.739986	6.70E-63	3.00E-61	Up	ANAPC7
CELSR1	1464.47694	2.2837608	0.13649424	16.7315542	7.72E-63	3.45E-61	Up	CELSR1
MTM1	776.39475	-1.38794281	0.08297062	-16.7281241	8.18E-63	3.65E-61	Down	MTM1
EXOSC2	1141.11582	1.11496525	0.06668855	16.7189916	9.53E-63	4.24E-61	Up	EXOSC2
CD163L1	290.331955	-2.86949324	0.17168246	-16.7139568	1.04E-62	4.60E-61	Down	CD163L1
C6orf15	109.291292	8.07078977	0.48287963	16.713875	1.04E-62	4.60E-61	Up	C6orf15
PXMP2	574.570096	-1.54024035	0.09231966	-16.6837738	1.72E-62	7.60E-61	Down	PXMP2
ACTR3B	371.216168	1.28840443	0.07728589	16.6706295	2.14E-62	9.44E-61	Up	ACTR3B

KANK2	2269.51028	-1.94093285	0.1165915	-16.6472922	3.17E-62	1.39E-60	Down	KANK2
SLC2A4	176.61248	-2.76347285	0.16616752	-16.6306439	4.18E-62	1.83E-60	Down	SLC2A4
SOX14	57.6840424	7.76038526	0.46673001	16.6271401	4.43E-62	1.94E-60	Up	SOX14
LIFR	176.079001	-3.28893632	0.19797648	-16.6127626	5.63E-62	2.46E-60	Down	LIFR
SHISA2	135.473759	3.14772971	0.1897034	16.5929006	7.84E-62	3.41E-60	Up	SHISA2
HPSE2	34.3789143	-3.36768598	0.20299594	-16.5899182	8.24E-62	3.58E-60	Down	HPSE2
GNAO1	219.936178	-3.55912115	0.21467	-16.5794991	9.80E-62	4.25E-60	Down	GNAO1
ZIC2	224.86358	6.09790014	0.36817321	16.562585	1.30E-61	5.61E-60	Up	ZIC2
TMEM151B	8.81940143	-3.29707431	0.19909705	-16.5601365	1.35E-61	5.83E-60	Down	TMEM151B
HADHA	8739.91933	-0.91677718	0.05536303	-16.5593739	1.37E-61	5.89E-60	Down	HADHA
TIMP1	10084.3949	2.01466738	0.12173912	16.5490551	1.63E-61	6.98E-60	Up	TIMP1
GJB4	144.28068	3.78525541	0.22882733	16.5419727	1.83E-61	7.83E-60	Up	GJB4
WNT7B	47.5535288	5.42587211	0.32801044	16.5417666	1.84E-61	7.84E-60	Up	WNT7B
MT1F	476.0844	-2.9225961	0.17670872	-16.5390602	1.92E-61	8.18E-60	Down	MT1F
HSP90AB1	50804.3695	1.08426867	0.06557675	16.5343467	2.08E-61	8.82E-60	Up	HSP90AB1
CAD	3574.24218	1.44658806	0.08754115	16.5246632	2.44E-61	1.03E-59	Up	CAD
WDR75	1587.64934	1.08715449	0.06579112	16.5243354	2.45E-61	1.04E-59	Up	WDR75
TEX10	1190.56168	1.011594	0.06122333	16.523016	2.51E-61	1.06E-59	Up	TEX10
GRHL1	208.5633	2.37117962	0.14359202	16.5133101	2.94E-61	1.24E-59	Up	GRHL1
KRT75	59.0745258	7.4513158	0.45157302	16.5007995	3.62E-61	1.52E-59	Up	KRT75
GNG7	126.507634	-2.64353795	0.16021791	-16.4996403	3.69E-61	1.55E-59	Down	GNG7
UCN2	21.3937925	4.70118958	0.28509754	16.4897584	4.35E-61	1.82E-59	Up	UCN2
GDPD5	1341.66136	2.86452225	0.17375808	16.4856919	4.65E-61	1.94E-59	Up	GDPD5
PKP1	434.189043	4.03848233	0.24498339	16.484719	4.72E-61	1.97E-59	Up	PKP1
RFWD3	1964.85926	0.99787371	0.06053995	16.4828964	4.87E-61	2.02E-59	Up	RFWD3
STRIP2	248.542452	2.41559199	0.14655631	16.4823473	4.91E-61	2.03E-59	Up	STRIP2
NKD1	3360.88865	4.09263544	0.24853424	16.4670889	6.32E-61	2.61E-59	Up	NKD1
CDKL1	68.2672344	-1.77830414	0.10809046	-16.4519991	8.11E-61	3.34E-59	Down	CDKL1
SLC25A20	797.731943	-1.49390452	0.09081274	-16.4503844	8.33E-61	3.43E-59	Down	SLC25A20
ZSWIM4	665.007454	1.47370196	0.08967981	16.4329291	1.11E-60	4.56E-59	Up	ZSWIM4
ZNF473	471.782888	1.02226554	0.06223821	16.4250476	1.27E-60	5.18E-59	Up	ZNF473
ZER1	2001.41492	-1.2111755	0.07375913	-16.4206852	1.36E-60	5.55E-59	Down	ZER1
TSPAN7	708.32883	-2.75231269	0.16766068	-16.4159701	1.47E-60	5.99E-59	Down	TSPAN7
CALM1	15373.6716	-0.9024807	0.05500968	-16.4058539	1.74E-60	7.06E-59	Down	CALM1
CALCOCO2	3469.39347	-0.9427614	0.05755027	-16.3815291	2.59E-60	1.05E-58	Down	CALCOCO2
CADM3	97.5486419	-4.68359871	0.28594557	-16.3793367	2.69E-60	1.09E-58	Down	CADM3
ZNF692	830.341081	1.6445851	0.10041083	16.3785624	2.72E-60	1.10E-58	Up	ZNF692
RNF152	324.332458	-2.82578066	0.17256666	-16.3750097	2.88E-60	1.16E-58	Down	RNF152
RAB27A	967.502207	-1.66373171	0.10162497	-16.3712891	3.07E-60	1.23E-58	Down	RAB27A
AKR1C4	44.3416842	6.01987504	0.36794636	16.360741	3.65E-60	1.46E-58	Up	AKR1C4
PHLDA1	5301.78488	2.49318026	0.15249647	16.3491017	4.41E-60	1.77E-58	Up	PHLDA1
POLR2D	1592.34703	0.79533593	0.04867901	16.3383742	5.26E-60	2.10E-58	Up	POLR2D
SF3B3	7928.15209	0.93457186	0.05722229	16.3323041	5.81E-60	2.32E-58	Up	SF3B3
NAP1L2	23.7234476	-3.72558028	0.22816088	-16.328743	6.16E-60	2.45E-58	Down	NAP1L2
AURKA	1757.97273	1.54863048	0.09487225	16.3233233	6.74E-60	2.67E-58	Up	AURKA
ACADS	2222.2699	-2.09604389	0.12849331	-16.3124749	8.05E-60	3.18E-58	Down	ACADS
INAFM2	201.62807	-1.61980895	0.09935027	-16.3040209	9.24E-60	3.65E-58	Down	INAFM2
UGT1A8	62.4821574	-4.23613781	0.25996843	-16.2948163	1.07E-59	4.23E-58	Down	UGT1A8
RECQL4	1629.48597	2.3012017	0.14133218	16.28222	1.32E-59	5.19E-58	Up	RECQL4
CAB39	3621.74381	-0.94856063	0.05830159	-16.2698921	1.61E-59	6.33E-58	Down	CAB39
ZIC5	92.1876968	8.47136642	0.52077311	16.2669046	1.70E-59	6.63E-58	Up	ZIC5
LRRN2	217.030599	-3.49009208	0.21460402	-16.2629389	1.81E-59	7.06E-58	Down	LRRN2
FGF19	116.814159	6.56602615	0.40378804	16.2610715	1.86E-59	7.26E-58	Up	FGF19
NR5A2	477.709023	-2.26363758	0.1392593	-16.2548401	2.06E-59	8.03E-58	Down	NR5A2
TMEM236	557.547612	-3.57731438	0.22012653	-16.2511733	2.19E-59	8.50E-58	Down	TMEM236
ARL14	367.868114	-2.48799908	0.15310013	-16.2507972	2.20E-59	8.54E-58	Down	ARL14
TNNI3	57.8131654	4.86835763	0.29983335	16.2368784	2.77E-59	1.07E-57	Up	TNNI3
RAE1	2215.02577	1.37099028	0.08448938	16.2267755	3.26E-59	1.26E-57	Up	RAE1

RCL1	534.814762	1.04463283	0.0643837	16.2251144	3.35E-59	1.29E-57	Up	RCL1
CDK2	1359.71497	1.00213794	0.06178999	16.2184523	3.73E-59	1.43E-57	Up	CDK2
NTN1	245.745454	-2.78083103	0.1714644	-16.2181248	3.75E-59	1.44E-57	Down	NTN1
SYNM	1989.63001	-4.41359266	0.2723139	-16.2077394	4.45E-59	1.70E-57	Down	SYNM
TPM1	12719.6761	-1.7315718	0.10685218	-16.2053015	4.63E-59	1.76E-57	Down	TPM1
ALPI	204.145975	-4.28241256	0.26431876	-16.2016972	4.91E-59	1.87E-57	Down	ALPI
TOP1MT	2437.54212	1.8647007	0.11516337	16.1917871	5.76E-59	2.19E-57	Up	TOP1MT
RBPMS2	147.40127	-3.44491712	0.21277878	-16.1901346	5.92E-59	2.24E-57	Down	RBPMS2
PPP1R12B	2751.56621	-2.76524324	0.17084009	-16.1861491	6.32E-59	2.39E-57	Down	PPP1R12B
NME1	2709.15018	1.70653263	0.1054938	16.1766153	7.37E-59	2.78E-57	Up	NME1
TRAK2	1698.95701	-1.07647456	0.06654588	-16.176427	7.40E-59	2.78E-57	Down	TRAK2
ATIC	4864.65508	1.06175968	0.06564509	16.1742428	7.66E-59	2.88E-57	Up	ATIC
E2F1	1187.32523	1.8927128	0.11714762	16.1566477	1.02E-58	3.82E-57	Up	E2F1
GLIPR2	752.725323	-2.01160749	0.12454796	-16.1512675	1.11E-58	4.16E-57	Down	GLIPR2
ST6GALNAC	1908.36809	-2.61495915	0.16190711	-16.1509836	1.12E-58	4.17E-57	Down	ST6GALNAC6
CDCA5	1591.7988	1.50116215	0.09296024	16.1484326	1.16E-58	4.34E-57	Up	CDCA5
JOSD1	2896.00884	-0.95613371	0.05922886	-16.1430381	1.27E-58	4.73E-57	Down	JOSD1
CEACAM1	9775.92355	-2.22851294	0.13805818	-16.1418393	1.30E-58	4.81E-57	Down	CEACAM1
IRX5	44.4928433	4.20830671	0.26074913	16.1392934	1.35E-58	5.00E-57	Up	IRX5
RHBDF2	1684.76814	1.55737899	0.09655246	16.1298736	1.57E-58	5.81E-57	Up	RHBDF2
DHRS11	1698.02219	-2.19899323	0.13635595	-16.1268599	1.65E-58	6.09E-57	Down	DHRS11
DIAPH3	653.572952	1.61970055	0.10044106	16.1258801	1.68E-58	6.18E-57	Up	DIAPH3
INPP5A	1037.0474	-0.94904939	0.05885741	-16.1245529	1.71E-58	6.30E-57	Down	INPP5A
FTSJ1	1696.72247	1.22005312	0.07567143	16.1230356	1.76E-58	6.44E-57	Up	FTSJ1
PRPF4	1677.48421	0.85388521	0.05297468	16.1187413	1.88E-58	6.89E-57	Up	PRPF4
C5orf34	140.729732	1.51824334	0.09423892	16.1105774	2.15E-58	7.85E-57	Up	C5orf34
KCNN3	151.961894	-2.44081314	0.15157954	-16.1025242	2.45E-58	8.92E-57	Down	KCNN3
NBPF1	456.457838	-1.52751567	0.09487791	-16.099803	2.56E-58	9.30E-57	Down	NBPF1
SMYD5	1436.9344	1.10331444	0.06853053	16.0996047	2.57E-58	9.31E-57	Up	SMYD5
SLC12A8	787.757104	1.16163402	0.0721658	16.0967396	2.69E-58	9.73E-57	Up	SLC12A8
WDR62	580.427833	1.86732528	0.11602173	16.0946167	2.78E-58	1.01E-56	Up	WDR62
KCNJ14	88.758805	2.01614302	0.12534324	16.084976	3.25E-58	1.17E-56	Up	KCNJ14
KIF2C	1288.02462	1.4370947	0.08937013	16.0802572	3.51E-58	1.26E-56	Up	KIF2C
POP1	534.332377	1.45530868	0.09051172	16.0786763	3.60E-58	1.29E-56	Up	POP1
SUCLG2	4334.59853	-1.33586243	0.08312557	-16.0704148	4.11E-58	1.47E-56	Down	SUCLG2
PUS1	1676.63221	1.6026517	0.09975149	16.0664434	4.39E-58	1.57E-56	Up	PUS1
TMEM97	2513.69573	1.57099331	0.09784339	16.0562029	5.17E-58	1.85E-56	Up	TMEM97
CXCL12	1078.95291	-2.70831247	0.16870249	-16.0537785	5.38E-58	1.92E-56	Down	CXCL12
TFR2	166.272881	2.83548153	0.1766717	16.049438	5.77E-58	2.05E-56	Up	TFR2
PDE8A	1404.34673	-1.15270797	0.0718268	-16.0484377	5.86E-58	2.08E-56	Down	PDE8A
NXPH4	210.262987	5.3463709	0.33318677	16.0461682	6.08E-58	2.15E-56	Up	NXPH4
E2F7	277.603735	1.87762884	0.11708411	16.0365806	7.10E-58	2.51E-56	Up	E2F7
DNAJB5	225.090349	-2.55549761	0.15939715	-16.0322668	7.61E-58	2.68E-56	Down	DNAJB5
MMP13	73.6596269	5.58934546	0.34895431	16.0174135	9.66E-58	3.40E-56	Up	MMP13
GLS2	36.8872635	2.52832833	0.15791009	16.011189	1.07E-57	3.75E-56	Up	GLS2
NCAM1	202.347859	-2.99407707	0.18700297	-16.010853	1.07E-57	3.76E-56	Down	NCAM1
SMIM14	2586.10642	-1.81221991	0.11332637	-15.9911579	1.47E-57	5.15E-56	Down	SMIM14
ATP2B4	2494.01555	-2.27350322	0.1422193	-15.9858976	1.60E-57	5.60E-56	Down	ATP2B4
POLR1C	1433.35338	1.31330608	0.08220711	15.9755786	1.89E-57	6.59E-56	Up	POLR1C
MARVELD3	1352.3647	-1.23928285	0.0775813	-15.9739891	1.94E-57	6.75E-56	Down	MARVELD3
ELANE	4.37278474	-3.56112321	0.22319603	-15.9551369	2.62E-57	9.11E-56	Down	ELANE
DMBX1	30.3491095	6.22613868	0.39025638	15.9539701	2.67E-57	9.26E-56	Up	DMBX1
MYH11	22381.5938	-4.56646701	0.28623681	-15.9534582	2.70E-57	9.32E-56	Down	MYH11
BUB1	1332.25481	1.360624	0.08532511	15.9463491	3.02E-57	1.04E-55	Up	BUB1
UHRF1	865.37015	1.89230485	0.11868601	15.9437907	3.15E-57	1.08E-55	Up	UHRF1
CA1	3377.16493	-6.48117354	0.40651614	-15.9432132	3.18E-57	1.09E-55	Down	CA1
SMPD1	1113.21311	-1.77466339	0.11132079	-15.9418871	3.24E-57	1.11E-55	Down	SMPD1
CXCL3	1614.95361	2.94532421	0.1849008	15.9292132	3.97E-57	1.36E-55	Up	CXCL3

HAUS5	746.108312	1.21637844	0.07647989	15.904552	5.89E-57	2.01E-55	Up	HAUS5
CPM	739.545594	-2.5593345	0.16095385	-15.9010459	6.23E-57	2.13E-55	Down	CPM
ANK2	233.376907	-3.22008989	0.20256357	-15.8966883	6.68E-57	2.28E-55	Down	ANK2
SLC5A6	4141.65161	1.88635444	0.11866575	15.8963676	6.71E-57	2.28E-55	Up	SLC5A6
CMTM8	546.700293	1.35191143	0.08506341	15.8929847	7.09E-57	2.40E-55	Up	CMTM8
PAH	126.317385	5.51709137	0.3471893	15.8907299	7.35E-57	2.49E-55	Up	PAH
PAICS	7026.87334	1.25128813	0.07874725	15.8899272	7.44E-57	2.52E-55	Up	PAICS
FAM180B	3.47564068	-4.57013494	0.28763773	-15.8885099	7.61E-57	2.57E-55	Down	FAM180B
NOM1	1091.0813	0.91548696	0.05763986	15.8828802	8.33E-57	2.80E-55	Up	NOM1
KCNA5	17.9347049	-2.66016503	0.16758028	-15.873974	9.60E-57	3.23E-55	Down	KCNA5
CAP2	192.132182	-2.41606492	0.15230491	-15.863342	1.14E-56	3.81E-55	Down	CAP2
GALNT6	2740.75862	1.89323529	0.11936556	15.8608165	1.18E-56	3.96E-55	Up	GALNT6
MFSD12	2300.58513	1.6356901	0.10312962	15.8605272	1.19E-56	3.97E-55	Up	MFSD12
LMOD1	1505.62042	-3.64780174	0.23002873	-15.8580265	1.24E-56	4.13E-55	Down	LMOD1
WDR12	1632.81261	1.02419512	0.06458857	15.8572199	1.25E-56	4.17E-55	Up	WDR12
CNN1	3610.54989	-4.13910623	0.26103481	-15.8565296	1.27E-56	4.21E-55	Down	CNN1
CORIN	85.585325	3.42535101	0.21636849	15.8310993	1.90E-56	6.30E-55	Up	CORIN
DHRS2	109.487559	3.8797688	0.24509881	15.829407	1.95E-56	6.46E-55	Up	DHRS2
FAM227A	83.3360406	2.35619832	0.14894059	15.8197196	2.28E-56	7.52E-55	Up	FAM227A
CD44	11623.9932	1.32724468	0.08393568	15.8126407	2.55E-56	8.40E-55	Up	CD44
TRIM27	3015.60423	0.9134063	0.05777742	15.8090536	2.69E-56	8.87E-55	Up	TRIM27
PDZD4	66.7590247	-3.23929128	0.20496993	-15.8037387	2.93E-56	9.64E-55	Down	PDZD4
AFF3	66.8201359	-3.12679226	0.19804593	-15.7882176	3.75E-56	1.23E-54	Down	AFF3
ABI3BP	345.17951	-3.19980311	0.20277063	-15.7804071	4.24E-56	1.39E-54	Down	ABI3BP
CCNF	1177.37929	1.4774096	0.09370078	15.7673142	5.22E-56	1.71E-54	Up	CCNF
FLNC	2708.58337	-3.4497423	0.21909019	-15.7457635	7.34E-56	2.40E-54	Down	FLNC
SH2D7	29.2451535	-3.62814783	0.23045115	-15.7436745	7.59E-56	2.47E-54	Down	SH2D7
PBX4	128.242993	2.04334924	0.12981854	15.7400415	8.04E-56	2.61E-54	Up	PBX4
GBA3	101.67644	-4.79631911	0.30495369	-15.7280244	9.72E-56	3.15E-54	Down	GBA3
MYBL2	4144.29762	2.15690414	0.13724562	15.7156506	1.18E-55	3.83E-54	Up	MYBL2
C1orf210	899.614178	-1.55727701	0.09910708	-15.7130756	1.23E-55	3.98E-54	Down	C1orf210
MT1M	269.61622	-4.28312053	0.27267964	-15.7075188	1.34E-55	4.34E-54	Down	MT1M
RERGL	17.3848624	-5.28738641	0.33665918	-15.7054573	1.39E-55	4.47E-54	Down	RERGL
C1orf109	639.092635	0.93451765	0.05954812	15.6934873	1.68E-55	5.39E-54	Up	C1orf109
ANKRD13A	1429.91556	-0.97195132	0.06194386	-15.6908415	1.75E-55	5.61E-54	Down	ANKRD13A
NCOA4	6040.52404	-1.03083045	0.06575464	-15.6769234	2.18E-55	6.97E-54	Down	NCOA4
ACACB	776.11595	-1.8080548	0.11535519	-15.6738059	2.28E-55	7.31E-54	Down	ACACB
WDR4	820.503791	1.5787759	0.10074608	15.6708426	2.39E-55	7.64E-54	Up	WDR4
PDE9A	1004.62297	-2.58617044	0.16509441	-15.6647974	2.63E-55	8.39E-54	Down	PDE9A
ACSL6	819.116243	4.31824346	0.27568659	15.6635962	2.68E-55	8.53E-54	Up	ACSL6
KCNE2	4.80586718	-2.74741143	0.17552874	-15.6522024	3.21E-55	1.02E-53	Down	KCNE2
PALB2	635.183031	0.91139763	0.05823932	15.6491795	3.37E-55	1.07E-53	Up	PALB2
JAM2	211.441141	-2.52552143	0.16141237	-15.6463931	3.52E-55	1.11E-53	Down	JAM2
SRPX2	1097.00471	3.08495993	0.19722311	15.6419801	3.77E-55	1.19E-53	Up	SRPX2
FAM161B	121.641045	-1.40010651	0.08954493	-15.635798	4.15E-55	1.31E-53	Down	FAM161B
PADI2	3182.80983	-3.25837018	0.20842168	-15.6335474	4.30E-55	1.35E-53	Down	PADI2
ZNF121	1455.80947	1.67623549	0.10723002	15.6321476	4.40E-55	1.38E-53	Up	ZNF121
ASAP3	480.41995	-1.84202451	0.11783867	-15.6317489	4.42E-55	1.39E-53	Down	ASAP3
PINK1	864.01924	-1.47914861	0.09463628	-15.6298268	4.56E-55	1.43E-53	Down	PINK1
NOB1	2801.42894	1.20062791	0.07682641	15.6278013	4.71E-55	1.47E-53	Up	NOB1
BVES	96.0140951	-2.89490271	0.18526434	-15.625796	4.86E-55	1.52E-53	Down	BVES
SHCBP1	485.077359	1.35024377	0.08641953	15.6242898	4.97E-55	1.55E-53	Up	SHCBP1
SRPX	233.864925	-2.76933252	0.17727395	-15.6217684	5.18E-55	1.61E-53	Down	SRPX
FANCA	899.094685	1.4243629	0.09121204	15.6159518	5.67E-55	1.76E-53	Up	FANCA
PRIMA1	85.4174899	-4.74156889	0.30371131	-15.6120918	6.02E-55	1.87E-53	Down	PRIMA1
CKAP2	1905.28911	1.51827381	0.09726562	15.6095634	6.27E-55	1.94E-53	Up	CKAP2
EML1	351.840766	-2.39101812	0.15327351	-15.5996828	7.32E-55	2.26E-53	Down	EML1
PTPRH	1804.91866	-2.04958942	0.13141262	-15.5965955	7.68E-55	2.37E-53	Down	PTPRH

CDCA3	881.779424	1.54533445	0.09909707	15.5941486	7.98E-55	2.45E-53	Up	CDCA3
CDCA7	5154.36632	1.6863258	0.10823986	15.5795272	1.00E-54	3.08E-53	Up	CDCA7
FGL2	902.727147	-2.73155328	0.17534602	-15.5780741	1.03E-54	3.15E-53	Down	FGL2
TACR2	250.96144	-3.90758524	0.251215	-15.5547447	1.48E-54	4.51E-53	Down	TACR2
P2RY1	344.112241	-2.53971203	0.16327509	-15.5548036	1.48E-54	4.51E-53	Down	P2RY1
SCN9A	99.0962721	-3.49487473	0.22474691	-15.5502685	1.58E-54	4.83E-53	Down	SCN9A
AMPD1	23.4533016	-3.9960783	0.25718777	-15.5375906	1.93E-54	5.88E-53	Down	AMPD1
PBLD	895.588151	-2.23795969	0.14404973	-15.5360219	1.98E-54	6.02E-53	Down	PBLD
NELFCD	5204.26413	1.47581643	0.09503968	15.528424	2.23E-54	6.76E-53	Up	NELFCD
VEGFA	6024.01179	1.53959284	0.09916871	15.5249869	2.35E-54	7.12E-53	Up	VEGFA
PLEKHG4	1459.91566	2.12322949	0.13683969	15.5161816	2.70E-54	8.16E-53	Up	PLEKHG4
CAND2	52.3039881	-2.24795634	0.14488656	-15.5152855	2.73E-54	8.26E-53	Down	CAND2
LYAR	1036.05184	1.34954779	0.08698665	15.5144235	2.77E-54	8.35E-53	Up	LYAR
UNC5C	120.123827	-2.28044964	0.14706584	-15.506318	3.14E-54	9.46E-53	Down	UNC5C
RAD54B	137.531519	1.83663548	0.11846434	15.5036985	3.27E-54	9.84E-53	Up	RAD54B
ASB2	218.513556	-2.97533934	0.19196698	-15.4992248	3.51E-54	1.05E-52	Down	ASB2
KIF26B	489.763551	3.15543575	0.20362195	15.4965406	3.66E-54	1.10E-52	Up	KIF26B
NMB	241.368978	1.90081298	0.12268864	15.4929821	3.87E-54	1.16E-52	Up	NMB
LRRC36	127.008108	3.24336663	0.20938554	15.4899267	4.06E-54	1.21E-52	Up	LRRC36
CST4	17.125552	6.49679421	0.41953705	15.4856267	4.34E-54	1.29E-52	Up	CST4
DSG3	470.399599	5.36986	0.34679704	15.4841574	4.44E-54	1.32E-52	Up	DSG3
NOP56	5519.98546	1.40978859	0.09105743	15.4824106	4.56E-54	1.35E-52	Up	NOP56
C4orf19	655.922338	-1.66552067	0.10763609	-15.4736268	5.23E-54	1.55E-52	Down	C4orf19
P2RY14	103.459736	-2.66964973	0.17255151	-15.4716106	5.39E-54	1.60E-52	Down	P2RY14
YTHDF1	3326.01906	1.0548391	0.06818165	15.4710109	5.44E-54	1.61E-52	Up	YTHDF1
TXNRD3	286.110285	1.17486019	0.07596192	15.4664363	5.85E-54	1.72E-52	Up	TXNRD3
ACTG2	9504.7759	-4.1457566	0.26811258	-15.4627458	6.19E-54	1.82E-52	Down	ACTG2
CARD14	161.728033	2.38368799	0.15417196	15.4612292	6.34E-54	1.86E-52	Up	CARD14
PAPSS2	3451.68741	-2.00302749	0.12959771	-15.4557322	6.90E-54	2.03E-52	Down	PAPSS2
DGAT2	1134.32256	1.93300741	0.12508851	15.4531171	7.19E-54	2.11E-52	Up	DGAT2
RPGRIP1L	330.451649	1.27789538	0.08272236	15.4480041	7.78E-54	2.28E-52	Up	RPGRIP1L
NEK2	912.542769	1.40085888	0.09074407	15.4374703	9.16E-54	2.68E-52	Up	NEK2
CCL23	15.5197989	-3.27601548	0.21226667	-15.4334894	9.75E-54	2.84E-52	Down	CCL23
CNN2	6623.78844	1.15267057	0.07469147	15.4324267	9.91E-54	2.88E-52	Up	CNN2
CCDC86	1319.28185	1.27270596	0.08248044	15.4303978	1.02E-53	2.97E-52	Up	CCDC86
GTSE1	709.950645	1.33890681	0.08677663	15.4293471	1.04E-53	3.02E-52	Up	GTSE1
EXO1	477.096724	1.52343501	0.09877365	15.4234959	1.14E-53	3.30E-52	Up	EXO1
ZBTB7B	3128.12666	-1.39514252	0.09045976	-15.4227968	1.15E-53	3.33E-52	Down	ZBTB7B
CEP55	1322.38834	1.5481848	0.10044729	15.4129078	1.34E-53	3.87E-52	Up	CEP55
CAMK2G	1805.2881	-0.95433837	0.06192186	-15.4119791	1.36E-53	3.92E-52	Down	CAMK2G
CENPI	288.184767	1.56710584	0.10172805	15.4048553	1.52E-53	4.37E-52	Up	CENPI
PCSK5	550.970436	-2.43000739	0.15776872	-15.4023394	1.58E-53	4.54E-52	Down	PCSK5
UTP14A	1542.13251	1.36335808	0.08851711	15.4021977	1.58E-53	4.54E-52	Up	UTP14A
EMILIN3	14.6294378	-2.57149972	0.16695949	-15.4019379	1.59E-53	4.55E-52	Down	EMILIN3
IBSP	34.2232053	5.91089304	0.38393723	15.3954673	1.76E-53	5.02E-52	Up	IBSP
TMPRSS13	383.332507	3.39525102	0.22064033	15.3881704	1.97E-53	5.61E-52	Up	TMPRSS13
WDR77	1929.77419	1.38332645	0.08993432	15.3815185	2.18E-53	6.21E-52	Up	WDR77
ADCY9	936.363837	-1.44715715	0.09412601	-15.3746782	2.42E-53	6.89E-52	Down	ADCY9
GUCA2B	343.83796	-5.98724714	0.38945917	-15.3732344	2.47E-53	7.03E-52	Down	GUCA2B
TTI1	1561.57875	1.23665451	0.08048937	15.3641966	2.85E-53	8.07E-52	Up	TTI1
TPM2	6431.00889	-2.69381839	0.17545168	-15.35362	3.35E-53	9.49E-52	Down	TPM2
BRCA2	583.368467	2.03722324	0.13269455	15.3527274	3.40E-53	9.60E-52	Up	BRCA2
ABHD5	466.345035	-1.13132932	0.07371776	-15.346768	3.72E-53	1.05E-51	Down	ABHD5
C8orf88	21.6907736	-3.22563593	0.21020311	-15.3453291	3.81E-53	1.07E-51	Down	C8orf88
NEDD4L	2330.85122	-1.29811399	0.08459472	-15.3450953	3.82E-53	1.08E-51	Down	NEDD4L
MTBP	263.100209	1.71876104	0.11206129	15.3376875	4.28E-53	1.20E-51	Up	MTBP
ADAT2	567.470785	1.55258402	0.10122866	15.3373957	4.30E-53	1.21E-51	Up	ADAT2
TNFSF15	579.251117	1.80418249	0.11767286	15.3321885	4.66E-53	1.31E-51	Up	TNFSF15

SLC30A10	70.5352522	-5.42396563	0.35382722	-15.3294189	4.86E-53	1.36E-51	Down	SLC30A10
MOB3B	1169.57743	-1.49497249	0.09752811	-15.3286316	4.92E-53	1.37E-51	Down	MOB3B
CENPA	395.567809	1.43171456	0.09340564	15.327924	4.98E-53	1.39E-51	Up	CENPA
IMPDH1	3398.10439	1.64665832	0.10745517	15.3241424	5.27E-53	1.47E-51	Up	IMPDH1
ATAD2	1777.10496	1.57789326	0.10298897	15.3209932	5.54E-53	1.54E-51	Up	ATAD2
UGT1A10	246.648486	-3.57755798	0.23361792	-15.3137137	6.19E-53	1.72E-51	Down	UGT1A10
PGM5	743.963846	-3.60645146	0.23581144	-15.2937932	8.41E-53	2.33E-51	Down	PGM5
TTYH3	6801.06726	1.62906523	0.10656601	15.2869128	9.35E-53	2.59E-51	Up	TTYH3
GNG2	354.678213	-2.04621542	0.13391093	-15.280421	1.03E-52	2.85E-51	Down	GNG2
LDHD	546.424937	-2.89582631	0.18954752	-15.2775742	1.08E-52	2.98E-51	Down	LDHD
NOP58	3226.59688	1.20895875	0.0791687	15.2706647	1.20E-52	3.30E-51	Up	NOP58
UBE2S	1373.7634	1.88314235	0.1233505	15.2665973	1.28E-52	3.51E-51	Up	UBE2S
PJA2	2546.10344	-1.40535063	0.09216447	-15.24829	1.69E-52	4.64E-51	Down	PJA2
DIXDC1	545.904536	-1.75428361	0.11505759	-15.2470052	1.72E-52	4.72E-51	Down	DIXDC1
NKPD1	29.8375477	3.30626761	0.2169735	15.2381174	1.97E-52	5.41E-51	Up	NKPD1
SPTBN5	254.421031	3.13110032	0.20549961	15.236527	2.02E-52	5.53E-51	Up	SPTBN5
TMEM131	2799.3399	-0.99079806	0.06504568	-15.2323417	2.16E-52	5.89E-51	Down	TMEM131
SLC22A11	101.155323	4.68146674	0.30735414	15.2315069	2.18E-52	5.95E-51	Up	SLC22A11
AXIN2	5227.03452	2.50908556	0.16473839	15.2307275	2.21E-52	6.02E-51	Up	AXIN2
ARHGEF25	267.487711	-2.49129898	0.16362693	-15.225483	2.40E-52	6.51E-51	Down	ARHGEF25
LMTK3	260.293291	2.24728852	0.14776187	15.2088532	3.09E-52	8.38E-51	Up	LMTK3
CNST	868.39426	-0.96369392	0.06340123	-15.1999254	3.54E-52	9.59E-51	Down	CNST
NOLC1	5703.96296	1.04939344	0.06908965	15.1888658	4.19E-52	1.13E-50	Up	NOLC1
GNL3	3825.2018	1.09500961	0.0721605	15.1746408	5.21E-52	1.41E-50	Up	GNL3
FMO4	129.098752	-1.70842386	0.11260352	-15.1720289	5.42E-52	1.46E-50	Down	FMO4
METTL24	16.9892952	-3.46685769	0.22855071	-15.1688776	5.68E-52	1.53E-50	Down	METTL24
PPP2R3A	302.432992	-2.01667483	0.13305404	-15.1568108	6.83E-52	1.84E-50	Down	PPP2R3A
CXCL1	2799.95267	2.92194701	0.19280492	15.1549398	7.03E-52	1.89E-50	Up	CXCL1
VSTM2A	23.2632824	-5.15254063	0.34017736	-15.1466299	7.98E-52	2.14E-50	Down	VSTM2A
LZTS3	1419.73923	2.27120223	0.15000376	15.140969	8.69E-52	2.33E-50	Up	LZTS3
CHAF1B	563.985885	1.3223342	0.08735018	15.1383106	9.05E-52	2.42E-50	Up	CHAF1B
CNGB1	5.30849665	-3.56592488	0.23561562	-15.1345009	9.59E-52	2.56E-50	Down	CNGB1
EIF3B	11392.8948	1.18338856	0.07820978	15.1309537	1.01E-51	2.70E-50	Up	EIF3B
KIF23	929.981271	1.31780446	0.08713	15.1245784	1.12E-51	2.97E-50	Up	KIF23
SLC7A11	844.81758	2.57803491	0.17047059	15.1230478	1.14E-51	3.03E-50	Up	SLC7A11
SGCG	4.43237126	-4.33630608	0.28677481	-15.1209449	1.18E-51	3.12E-50	Down	SGCG
HSPD1	22221.048	1.27223168	0.08414738	15.1190878	1.21E-51	3.21E-50	Up	HSPD1
RAD54L	437.362875	1.47447297	0.09758478	15.1096608	1.40E-51	3.70E-50	Up	RAD54L
SHH	943.961412	2.19856319	0.14551352	15.1089955	1.41E-51	3.73E-50	Up	SHH
KIAA0513	599.88645	-1.9399457	0.12841288	-15.1070957	1.45E-51	3.83E-50	Down	KIAA0513
SCN7A	126.40194	-5.11222964	0.33856958	-15.099495	1.63E-51	4.30E-50	Down	SCN7A
MAB21L2	515.327482	-3.39561096	0.22491529	-15.0972885	1.69E-51	4.43E-50	Down	MAB21L2
MTHFD2	3128.99464	1.37053832	0.09081722	15.0911725	1.85E-51	4.86E-50	Up	MTHFD2
CCDC113	377.874416	1.79217852	0.11892152	15.0702619	2.54E-51	6.66E-50	Up	CCDC113
SLC5A11	16.0902534	-3.49079958	0.23171387	-15.06513	2.75E-51	7.19E-50	Down	SLC5A11
ELAVL4	18.940155	-3.32557828	0.22082524	-15.0597741	2.98E-51	7.78E-50	Down	ELAVL4
CENPN	623.891537	1.2879319	0.08553171	15.0579472	3.06E-51	7.99E-50	Up	CENPN
FANCI	1734.61102	1.30872473	0.08693317	15.0543777	3.23E-51	8.42E-50	Up	FANCI
PKNOX2	24.8278953	-2.71708009	0.18049707	-15.05332	3.28E-51	8.54E-50	Down	PKNOX2
FGFBP2	10.1535274	-3.18240241	0.21145034	-15.0503537	3.43E-51	8.92E-50	Down	FGFBP2
NONO	14851.5906	0.90493265	0.06014233	15.0465175	3.64E-51	9.44E-50	Up	NONO
PLXNA1	2949.22068	1.36883055	0.09097608	15.0460485	3.66E-51	9.49E-50	Up	PLXNA1
WDR74	1291.52405	1.23645556	0.08223111	15.0363472	4.24E-51	1.10E-49	Up	WDR74
CDC45	749.589731	1.67352053	0.11133316	15.0316444	4.56E-51	1.18E-49	Up	CDC45
JADE3	795.128059	1.37467338	0.0914677	15.0290579	4.74E-51	1.22E-49	Up	JADE3
UBFD1	2132.83906	0.7642455	0.05085577	15.0277044	4.83E-51	1.25E-49	Up	UBFD1
JPH1	1048.9947	1.60753695	0.10699056	15.0250351	5.03E-51	1.29E-49	Up	JPH1
TLR3	384.504562	-2.04702409	0.1363454	-15.0135174	5.99E-51	1.54E-49	Down	TLR3

SYNPO2	2954.94918	-3.84472743	0.25624195	-15.0042858	6.88E-51	1.76E-49	Down	SYNPO2
GPT2	2751.71537	1.68937795	0.11261939	15.0007734	7.26E-51	1.86E-49	Up	GPT2
TCF21	167.108374	-2.24340188	0.14956566	-14.9994453	7.40E-51	1.89E-49	Down	TCF21
MELK	1059.75609	1.42910763	0.09528646	14.9980137	7.56E-51	1.93E-49	Up	MELK
MT1G	1914.26912	-3.37954927	0.22535455	-14.9965876	7.73E-51	1.97E-49	Down	MT1G
WT1	72.5334051	4.92398378	0.328486	14.9899351	8.54E-51	2.18E-49	Up	WT1
SLC17A4	276.704149	-3.28356413	0.21919266	-14.9802649	9.88E-51	2.51E-49	Down	SLC17A4
C1orf112	369.692663	1.21846687	0.08135192	14.9777281	1.03E-50	2.61E-49	Up	C1orf112
OPN3	267.948699	-1.57450923	0.10513216	-14.9764758	1.05E-50	2.65E-49	Down	OPN3
SRI	6552.2321	-1.54056318	0.10291347	-14.9694992	1.16E-50	2.94E-49	Down	SRI
ANKZF1	1538.57298	1.02543236	0.06852831	14.9636314	1.27E-50	3.21E-49	Up	ANKZF1
GINS1	1115.07577	1.67733347	0.11227542	14.9394536	1.82E-50	4.60E-49	Up	GINS1
CHPF	5900.3701	1.78428266	0.11943394	14.9394946	1.82E-50	4.60E-49	Up	CHPF
PLCD4	111.899306	-1.58640698	0.10619184	-14.9390667	1.84E-50	4.62E-49	Down	PLCD4
WSCD1	323.994338	-2.98303986	0.19971663	-14.9363622	1.91E-50	4.80E-49	Down	WSCD1
MT1X	816.886116	-2.38820626	0.15990224	-14.9354147	1.94E-50	4.87E-49	Down	MT1X
SORD	1799.38066	1.39131575	0.09316605	14.9337209	1.99E-50	4.98E-49	Up	SORD
CREB3L3	118.649325	-4.19139526	0.2808864	-14.9220297	2.37E-50	5.93E-49	Down	CREB3L3
LPAR1	361.915202	-2.19821957	0.14731456	-14.9219432	2.37E-50	5.93E-49	Down	LPAR1
RNFT2	146.854841	1.98708399	0.13317721	14.9206011	2.42E-50	6.04E-49	Up	RNFT2
SIAE	3435.56736	-1.55965206	0.10453287	-14.9202066	2.44E-50	6.07E-49	Down	SIAE
HHLA2	1558.50738	-2.75727776	0.18504871	-14.9002813	3.28E-50	8.17E-49	Down	HHLA2
SYNPO	2490.40344	-1.62179296	0.10887838	-14.8954546	3.53E-50	8.77E-49	Down	SYNPO
GTF3A	6901.80414	1.8453583	0.12388938	14.8952093	3.54E-50	8.79E-49	Up	GTF3A
BCAR3	685.878691	-1.41554803	0.09511921	-14.8818309	4.32E-50	1.07E-48	Down	BCAR3
HLF	110.697354	-2.61533386	0.17575423	-14.8806315	4.40E-50	1.09E-48	Down	HLF
EPYC	45.5441051	6.86393519	0.46142578	14.8754914	4.75E-50	1.18E-48	Up	EPYC
BOP1	4654.10071	2.01153812	0.13532983	14.8639664	5.65E-50	1.39E-48	Up	BOP1
PAFAH2	1075.17067	-1.18080601	0.07946337	-14.8597518	6.01E-50	1.48E-48	Down	PAFAH2
CCT3	12678.042	0.97129955	0.06541944	14.8472618	7.25E-50	1.78E-48	Up	CCT3
SETD6	541.188028	1.0563915	0.07115895	14.8455188	7.44E-50	1.83E-48	Up	SETD6
CKAP2L	530.21001	1.44933659	0.09765018	14.842129	7.82E-50	1.92E-48	Up	CKAP2L
CXCL5	1014.22187	5.11485562	0.34464945	14.8407481	7.99E-50	1.96E-48	Up	CXCL5
RFC4	808.508728	1.17530842	0.07920258	14.8392693	8.16E-50	2.00E-48	Up	RFC4
KCNMB1	333.019794	-3.30507162	0.22273212	-14.8387743	8.22E-50	2.01E-48	Down	KCNMB1
TTC22	1232.73936	-1.5508017	0.10451323	-14.8383288	8.28E-50	2.02E-48	Down	TTC22
TRAP1	4438.38705	1.3277903	0.08949837	14.8359165	8.58E-50	2.09E-48	Up	TRAP1
TRIM40	98.8760884	-3.05423893	0.20588424	-14.8347386	8.73E-50	2.13E-48	Down	TRIM40
CPEB1	19.8756355	-3.31573503	0.22362207	-14.8274053	9.74E-50	2.37E-48	Down	CPEB1
RGS16	648.664022	2.0753319	0.14004679	14.8188468	1.11E-49	2.69E-48	Up	RGS16
ITM2C	21481.4053	-2.21572948	0.14958526	-14.8124856	1.22E-49	2.95E-48	Down	ITM2C
SLC9A9	122.311481	-2.20633686	0.14903655	-14.8039985	1.38E-49	3.34E-48	Down	SLC9A9
MYEOV	1086.08871	3.0990818	0.20936422	14.8023466	1.41E-49	3.42E-48	Up	MYEOV
NCAPH	876.408535	1.2137095	0.08200251	14.8008816	1.45E-49	3.49E-48	Up	NCAPH
IER5L	566.489284	2.06685213	0.13966516	14.7986231	1.50E-49	3.61E-48	Up	IER5L
SECTM1	1236.5937	-2.88337985	0.19487649	-14.7959348	1.56E-49	3.75E-48	Down	SECTM1
RNF150	175.941439	-3.3536531	0.22674696	-14.7902889	1.69E-49	4.07E-48	Down	RNF150
PPRC1	2239.98737	1.05624982	0.07145769	14.7814713	1.93E-49	4.63E-48	Up	PPRC1
HSD17B2	895.093282	-3.36334104	0.22760183	-14.7773026	2.05E-49	4.92E-48	Down	HSD17B2
KIF4A	1185.88892	1.30421757	0.08826562	14.7760536	2.09E-49	5.01E-48	Up	KIF4A
EN2	64.1940634	6.24953593	0.42322127	14.7665924	2.41E-49	5.76E-48	Up	EN2
HAND2	142.985021	-4.04394927	0.27388148	-14.7653255	2.45E-49	5.86E-48	Down	HAND2
NTMT1	1028.23051	1.20913752	0.08189434	14.7646049	2.48E-49	5.91E-48	Up	NTMT1
PDZRN4	71.0815374	-4.29966912	0.29121862	-14.7644031	2.49E-49	5.92E-48	Down	PDZRN4
LY6G6D	348.095777	5.40740479	0.36657405	14.7511935	3.02E-49	7.19E-48	Up	LY6G6D
GPM6B	161.80368	-2.66919806	0.18095487	-14.7506287	3.05E-49	7.24E-48	Down	GPM6B
XKRX	232.746558	2.526212	0.17131166	14.7462935	3.25E-49	7.71E-48	Up	XKRX
TBC1D24	541.460546	1.01430523	0.06883129	14.7361056	3.78E-49	8.96E-48	Up	TBC1D24

PRDM12	11.026426	2.79263761	0.18951758	14.7355073	3.81E-49	9.03E-48	Up	PRDM12
KPNA2	4485.52831	1.18982742	0.0807485	14.7349779	3.84E-49	9.08E-48	Up	KPNA2
RHEBL1	70.2902113	1.6991276	0.1153292	14.7328486	3.97E-49	9.36E-48	Up	RHEBL1
ACAA2	4406.64218	-1.64075745	0.11137109	-14.7323456	4.00E-49	9.42E-48	Down	ACAA2
GREM2	254.233186	-3.7539917	0.25485984	-14.7296321	4.16E-49	9.79E-48	Down	GREM2
GDPD2	143.882067	-3.17196863	0.21536652	-14.7282344	4.25E-49	9.98E-48	Down	GDPD2
DCAF13	2340.73347	1.2964282	0.08809069	14.7169719	5.02E-49	1.18E-47	Up	DCAF13
LRRC19	1028.05278	-2.79670194	0.1900388	-14.7164787	5.05E-49	1.18E-47	Down	LRRC19
CEACAM7	12681.1249	-4.12755123	0.28054225	-14.7127615	5.34E-49	1.25E-47	Down	CEACAM7
RUNX1	2635.33583	1.44386283	0.09816217	14.7089542	5.65E-49	1.32E-47	Up	RUNX1
SCN11A	12.2139559	-3.41313694	0.23209256	-14.7059303	5.91E-49	1.38E-47	Down	SCN11A
CTPS1	1533.85553	1.12170148	0.07628343	14.704393	6.04E-49	1.41E-47	Up	CTPS1
PRMT3	895.409529	1.23729912	0.08419225	14.6961168	6.83E-49	1.59E-47	Up	PRMT3
EIF2S2	5032.17197	1.12967997	0.07690093	14.6900692	7.46E-49	1.74E-47	Up	EIF2S2
SLC35D3	247.053821	4.37619367	0.29792095	14.6891101	7.57E-49	1.76E-47	Up	SLC35D3
CYSRT1	47.9508688	2.68536528	0.18290859	14.6814605	8.47E-49	1.97E-47	Up	CYSRT1
RRP9	1671.54862	1.47054646	0.10020388	14.6755448	9.25E-49	2.14E-47	Up	RRP9
ULBP3	51.2940166	1.96522808	0.13393042	14.673501	9.53E-49	2.21E-47	Up	ULBP3
GMPS	2714.61818	0.86615993	0.05902987	14.6732475	9.57E-49	2.21E-47	Up	GMPS
PKMYT1	1226.50188	1.97810061	0.13484302	14.6696556	1.01E-48	2.33E-47	Up	PKMYT1
ARMC10	1584.08766	1.01313392	0.06906853	14.6685311	1.03E-48	2.36E-47	Up	ARMC10
CD177	2166.74635	-5.17089052	0.35254022	-14.6675192	1.04E-48	2.40E-47	Down	CD177
TDGF1	972.619883	3.02392835	0.20617985	14.6664594	1.06E-48	2.43E-47	Up	TDGF1
LDLRAD3	532.166678	1.9316311	0.13171584	14.6651389	1.08E-48	2.48E-47	Up	LDLRAD3
PYY	311.665261	-5.60234041	0.38202279	-14.6649376	1.08E-48	2.48E-47	Down	PYY
DHX34	1365.03145	1.41297922	0.09635172	14.664805	1.08E-48	2.48E-47	Up	DHX34
CCDC150	95.8844819	2.0466924	0.13957602	14.663639	1.10E-48	2.52E-47	Up	CCDC150
ATP6V1C2	298.688583	2.38432065	0.16263666	14.6604137	1.16E-48	2.64E-47	Up	ATP6V1C2
SLC2A13	526.880833	-1.69682533	0.11574369	-14.6601978	1.16E-48	2.65E-47	Down	SLC2A13
F2RL1	3373.09253	-1.30263638	0.08886122	-14.6592228	1.18E-48	2.68E-47	Down	F2RL1
TICRR	481.929972	1.58210212	0.10792815	14.6588464	1.18E-48	2.69E-47	Up	TICRR
BEND5	24.0740449	-2.57404177	0.17560644	-14.658015	1.20E-48	2.72E-47	Down	BEND5
AEN	1608.37944	1.33493005	0.09107246	14.65789	1.20E-48	2.72E-47	Up	AEN
EDIL3	934.721731	-2.13926236	0.14595948	-14.6565501	1.22E-48	2.77E-47	Down	EDIL3
NUF2	550.898672	1.63417265	0.11150125	14.6560929	1.23E-48	2.79E-47	Up	NUF2
SCLY	186.163669	1.48835332	0.10157214	14.6531643	1.29E-48	2.91E-47	Up	SCLY
GLDN	96.2484841	-2.81778129	0.19238796	-14.6463493	1.42E-48	3.21E-47	Down	GLDN
ERCC6L	307.455054	1.69809477	0.11595142	14.6448815	1.45E-48	3.28E-47	Up	ERCC6L
OLR1	196.838993	3.74866821	0.2559808	14.6443332	1.46E-48	3.30E-47	Up	OLR1
HOMER1	304.808627	1.925669	0.13150431	14.6433909	1.48E-48	3.34E-47	Up	HOMER1
RCAN2	315.749393	-1.91300952	0.13064471	-14.6428392	1.50E-48	3.36E-47	Down	RCAN2
ARHGAP44	772.325296	-2.19185626	0.14973544	-14.6381929	1.60E-48	3.60E-47	Down	ARHGAP44
TMIGD1	384.718596	-6.11613681	0.41786039	-14.636795	1.64E-48	3.67E-47	Down	TMIGD1
ADAMTSL3	129.199718	-3.30091536	0.22557564	-14.6332973	1.72E-48	3.86E-47	Down	ADAMTSL3
RRS1	1618.96022	1.38938451	0.09496321	14.6307661	1.79E-48	4.00E-47	Up	RRS1
CNTN4	99.7790158	-2.45158471	0.16760095	-14.6275109	1.88E-48	4.19E-47	Down	CNTN4
TMEM171	654.443812	-2.27997855	0.15589654	-14.624946	1.95E-48	4.34E-47	Down	TMEM171
CLEC5A	108.519836	3.55308806	0.24301005	14.6211571	2.06E-48	4.59E-47	Up	CLEC5A
ARRDC4	1089.01887	-1.68805479	0.11546148	-14.6200682	2.09E-48	4.65E-47	Down	ARRDC4
RGS9	41.7081794	-2.28067287	0.15604835	-14.6151682	2.25E-48	5.00E-47	Down	RGS9
MT1H	234.024925	-3.85535171	0.26387451	-14.6105499	2.41E-48	5.34E-47	Down	MT1H
HSD11B2	5497.08061	-2.22164336	0.15206446	-14.6098791	2.43E-48	5.39E-47	Down	HSD11B2
NANOS3	69.7009183	3.89900835	0.26704179	14.6007422	2.78E-48	6.15E-47	Up	NANOS3
NUFIP1	522.812305	1.37300224	0.09404905	14.598789	2.86E-48	6.32E-47	Up	NUFIP1
TNFSF10	2503.92279	-1.50190461	0.10289007	-14.5971769	2.93E-48	6.47E-47	Down	TNFSF10
TRMT1	1747.53811	1.43667776	0.09847472	14.5893047	3.29E-48	7.25E-47	Up	TRMT1
SLC35G1	387.032221	-1.37438995	0.09425996	-14.5808452	3.72E-48	8.19E-47	Down	SLC35G1
SHMT2	6151.84363	1.47398099	0.10112124	14.5763734	3.97E-48	8.74E-47	Up	SHMT2

ENOPH1	1912.74672	0.95508313	0.06554244	14.5719794	4.23E-48	9.31E-47	Up	ENOPH1
ASPHD1	316.042533	2.42392079	0.16635067	14.5711517	4.29E-48	9.41E-47	Up	ASPHD1
TMEM74B	254.975138	2.98912746	0.20521046	14.5661553	4.61E-48	1.01E-46	Up	TMEM74B
GSN	13440.1421	-1.8760572	0.12897657	-14.5457213	6.22E-48	1.36E-46	Down	GSN
TAGLN	11116.0121	-2.99036866	0.20558573	-14.5456043	6.23E-48	1.36E-46	Down	TAGLN
GINM1	1094.92202	-1.0526575	0.07237321	-14.5448498	6.30E-48	1.38E-46	Down	GINM1
DNAH2	178.683741	2.85075098	0.19600245	14.5444661	6.33E-48	1.38E-46	Up	DNAH2
STPG1	352.753559	-1.09067794	0.07505333	-14.5320388	7.59E-48	1.65E-46	Down	STPG1
GNG3	11.8817981	-2.24297485	0.15435944	-14.5308566	7.72E-48	1.68E-46	Down	GNG3
TARBP1	1811.97981	1.30687916	0.0899736	14.5251399	8.40E-48	1.83E-46	Up	TARBP1
EIF4E3	838.546375	-1.8556542	0.12779194	-14.5209012	8.93E-48	1.94E-46	Down	EIF4E3
HPGD	1527.83914	-2.85138745	0.19638942	-14.5190485	9.18E-48	1.99E-46	Down	HPGD
FBXO32	1684.92928	-2.19684955	0.15130938	-14.5189247	9.19E-48	1.99E-46	Down	FBXO32
ELF5	112.262389	7.3563747	0.50682098	14.51474	9.77E-48	2.11E-46	Up	ELF5
MRPL17	2125.0339	1.1960092	0.08240625	14.5135735	9.94E-48	2.15E-46	Up	MRPL17
FXYD1	17.0968469	-3.1023916	0.21375821	-14.5135555	9.94E-48	2.15E-46	Down	FXYD1
ZNF239	237.421939	1.75702979	0.12107868	14.5114718	1.02E-47	2.21E-46	Up	ZNF239
PEX11A	470.612689	-1.2425472	0.08563992	-14.5089715	1.06E-47	2.29E-46	Down	PEX11A
NUP43	1519.55966	0.89827776	0.06193443	14.5036898	1.15E-47	2.47E-46	Up	NUP43
CLIC5	2153.63457	-2.07581663	0.14315557	-14.5004252	1.20E-47	2.59E-46	Down	CLIC5
TBC1D30	551.564064	1.31744923	0.09085725	14.5002101	1.21E-47	2.59E-46	Up	TBC1D30
PLS1	6917.87692	-1.44037156	0.09936906	-14.4951709	1.30E-47	2.78E-46	Down	PLS1
PIRT	24.1291489	-4.16865973	0.28766664	-14.4912864	1.38E-47	2.94E-46	Down	PIRT
SLC26A3	14950.4108	-4.54087026	0.3134763	-14.4855296	1.50E-47	3.20E-46	Down	SLC26A3
ZPR1	1929.71224	0.83083227	0.05737531	14.4806578	1.61E-47	3.43E-46	Up	ZPR1
NUDCD1	1176.69196	1.36788925	0.094509	14.4736397	1.78E-47	3.79E-46	Up	NUDCD1
NLE1	1258.0626	1.30783319	0.09038687	14.4692822	1.89E-47	4.03E-46	Up	NLE1
CSNK1E	4131.98355	0.9671135	0.06684743	14.4674755	1.94E-47	4.14E-46	Up	CSNK1E
FXYD6	473.643536	-2.67717155	0.18519769	-14.4557506	2.31E-47	4.90E-46	Down	FXYD6
CHP2	1641.51999	-3.88653211	0.26887146	-14.4549818	2.33E-47	4.95E-46	Down	CHP2
C15orf48	5212.61321	-2.46764869	0.17081695	-14.4461584	2.65E-47	5.62E-46	Down	C15orf48
IL23A	107.441822	2.87489778	0.19909016	14.4401805	2.89E-47	6.12E-46	Up	IL23A
STMN4	5.58847821	-4.42614039	0.30659123	-14.4366176	3.04E-47	6.44E-46	Down	STMN4
C5orf46	18.6640794	6.16708754	0.42725531	14.4341976	3.15E-47	6.66E-46	Up	C5orf46
PPA1	11245.0755	1.17559772	0.08145516	14.4324521	3.23E-47	6.82E-46	Up	PPA1
LIF	1179.18411	1.83136755	0.12691494	14.4298815	3.36E-47	7.07E-46	Up	LIF
PLCL2	283.618417	-2.41492941	0.16742753	-14.4237296	3.67E-47	7.72E-46	Down	PLCL2
KIF14	394.308618	1.7734456	0.1229546	14.4235805	3.68E-47	7.73E-46	Up	KIF14
CDK5RAP1	1336.57082	1.09852296	0.07616739	14.4224838	3.74E-47	7.84E-46	Up	CDK5RAP1
THUMPD2	379.513481	0.98127247	0.06806208	14.4173148	4.03E-47	8.45E-46	Up	THUMPD2
EGLN1	1980.88628	-0.89397803	0.06202705	-14.4127112	4.30E-47	9.02E-46	Down	EGLN1
SHROOM4	914.535215	2.02258689	0.14039948	14.4059428	4.75E-47	9.93E-46	Up	SHROOM4
RNF112	25.1215239	-2.39745265	0.1665663	-14.3933835	5.69E-47	1.19E-45	Down	RNF112
ARL4D	109.862008	-2.33898319	0.16251382	-14.3925191	5.77E-47	1.20E-45	Down	ARL4D
SCP2	4451.98217	-1.1303501	0.07854086	-14.3918736	5.82E-47	1.21E-45	Down	SCP2
MFAP2	665.116884	2.39828673	0.16665372	14.3908384	5.91E-47	1.23E-45	Up	MFAP2
PCSK9	2405.64909	2.72178472	0.18914012	14.3903085	5.95E-47	1.24E-45	Up	PCSK9
S100B	115.80316	-2.50580778	0.17424294	-14.3811148	6.80E-47	1.41E-45	Down	S100B
MCM3	5640.83264	1.0555536	0.07344246	14.3725255	7.70E-47	1.60E-45	Up	MCM3
FLVCR2	232.584164	-1.95527442	0.13613551	-14.3627064	8.87E-47	1.84E-45	Down	FLVCR2
SPATA33	396.801251	1.30821702	0.0910908	14.3616809	9.00E-47	1.86E-45	Up	SPATA33
SDCBP2	2220.90064	-2.35550246	0.16405663	-14.3578619	9.51E-47	1.97E-45	Down	SDCBP2
OTUB2	259.615092	1.93356196	0.13468663	14.3560047	9.77E-47	2.02E-45	Up	OTUB2
NOL6	2662.76302	1.113746	0.07762474	14.3478221	1.10E-46	2.27E-45	Up	NOL6
CHTF18	1160.553	1.87706035	0.1308464	14.3455257	1.14E-46	2.34E-45	Up	CHTF18
PPBP	230.140932	5.87697814	0.40977848	14.3418417	1.20E-46	2.47E-45	Up	PPBP
HSD17B11	3863.40197	-1.33263385	0.09298628	-14.3315098	1.39E-46	2.86E-45	Down	HSD17B11
TNS1	4326.82142	-2.82629383	0.19722148	-14.3305576	1.41E-46	2.90E-45	Down	TNS1

CPT1A	5279.06136	-1.19522015	0.08344708	-14.3230912	1.57E-46	3.22E-45	Down	CPT1A
PRPH	39.2794124	-3.71220073	0.25920508	-14.3214812	1.61E-46	3.29E-45	Down	PRPH
ARHGEF18	363.850532	-1.30392517	0.09104894	-14.3211465	1.61E-46	3.30E-45	Down	ARHGEF18
C16orf89	51.3287239	-3.57396357	0.2496943	-14.3133566	1.81E-46	3.69E-45	Down	C16orf89
GART	3477.76897	0.94207318	0.06582247	14.3123339	1.83E-46	3.74E-45	Up	GART
NFXL1	555.64545	0.99008056	0.06918391	14.3108503	1.87E-46	3.81E-45	Up	NFXL1
SSTR2	46.8285453	-2.39649476	0.16745956	-14.3108869	1.87E-46	3.81E-45	Down	SSTR2
SLCO2A1	1006.11969	-2.07814069	0.14524405	-14.3079228	1.95E-46	3.97E-45	Down	SLCO2A1
BCHE	74.6922445	-4.30473578	0.30091673	-14.3054053	2.02E-46	4.12E-45	Down	BCHE
CDCA4	962.781345	1.40672911	0.09834703	14.303728	2.07E-46	4.21E-45	Up	CDCA4
MOBP	5.58233332	-2.94831999	0.20623692	-14.2957913	2.32E-46	4.71E-45	Down	MOBP
TM6SF2	48.5684919	-3.02893801	0.21187556	-14.2958345	2.32E-46	4.71E-45	Down	TM6SF2
DAND5	7.65166345	-3.1005336	0.21691569	-14.2937266	2.39E-46	4.84E-45	Down	DAND5
SLC13A3	322.584412	4.24809345	0.29741868	14.2832101	2.78E-46	5.63E-45	Up	SLC13A3
RCN1	5109.01188	1.23505384	0.08648661	14.280289	2.90E-46	5.86E-45	Up	RCN1
LGALS4	46883.1017	-1.85193964	0.12978499	-14.2692894	3.40E-46	6.86E-45	Down	LGALS4
NCAPD3	1700.34652	1.03426492	0.07249611	14.2664893	3.54E-46	7.13E-45	Up	NCAPD3
PRSS33	282.770632	4.7276651	0.33144555	14.263776	3.68E-46	7.40E-45	Up	PRSS33
AMPD2	1929.07038	1.10172182	0.07726893	14.2582781	3.98E-46	8.00E-45	Up	AMPD2
ZNF74	653.548118	1.26079754	0.08849423	14.2472287	4.66E-46	9.36E-45	Up	ZNF74
MALL	470.52969	-2.28308926	0.16025675	-14.2464467	4.72E-46	9.46E-45	Down	MALL
RACGAP1	2226.15509	1.06422572	0.07471142	14.2444851	4.85E-46	9.72E-45	Up	RACGAP1
CDC6	1353.74671	1.65723467	0.11635098	14.2434098	4.93E-46	9.86E-45	Up	CDC6
LILRB5	135.754181	-2.62672446	0.18453564	-14.234239	5.62E-46	1.12E-44	Down	LILRB5
TAMM41	432.575522	1.00956394	0.07094122	14.2309922	5.88E-46	1.17E-44	Up	TAMM41
SST	69.6410546	-5.33556694	0.37498405	-14.2287836	6.07E-46	1.21E-44	Down	SST
FGFRL1	3331.51745	2.48709687	0.17482374	14.2263109	6.29E-46	1.25E-44	Up	FGFRL1
MTFR2	287.40565	1.39045285	0.09776312	14.2226733	6.63E-46	1.32E-44	Up	MTFR2
LY6G6F	8.8999417	5.67587515	0.39919093	14.2184471	7.04E-46	1.40E-44	Up	LY6G6F
TMEM211	142.102884	3.97116024	0.27931855	14.2173163	7.15E-46	1.42E-44	Up	TMEM211
SULT1A1	826.710391	-2.12825749	0.14976856	-14.2103088	7.91E-46	1.57E-44	Down	SULT1A1
GFRA1	158.42359	-3.14658097	0.22147983	-14.2070769	8.28E-46	1.64E-44	Down	GFRA1
RHOU	1225.36524	-1.47407912	0.10377023	-14.2052215	8.50E-46	1.68E-44	Down	RHOU
DDX10	1195.79151	1.02593985	0.0722272	14.204343	8.61E-46	1.70E-44	Up	DDX10
FILIP1	124.015977	-2.67692057	0.18847209	-14.2032732	8.74E-46	1.73E-44	Down	FILIP1
SLC35D1	2296.52959	-1.29729153	0.09135583	-14.2004236	9.11E-46	1.80E-44	Down	SLC35D1
ADCY5	242.376805	-2.89683137	0.20400074	-14.2001022	9.15E-46	1.80E-44	Down	ADCY5
DACT3	190.406366	-2.57156539	0.18117082	-14.1941477	9.96E-46	1.96E-44	Down	DACT3
AMH	126.92795	4.17233008	0.2939687	14.1931099	1.01E-45	1.99E-44	Up	AMH
GBX2	12.3299233	5.96534818	0.42031424	14.1925912	1.02E-45	2.00E-44	Up	GBX2
SNAI1	227.761961	1.76580967	0.12443712	14.1903774	1.05E-45	2.06E-44	Up	SNAI1
MORC2	1666.05278	0.82571923	0.05819114	14.1897765	1.06E-45	2.08E-44	Up	MORC2
SLC22A3	776.263546	1.97973762	0.13958129	14.183403	1.16E-45	2.27E-44	Up	SLC22A3
MYOCD	232.613436	-3.07069644	0.21652707	-14.181582	1.19E-45	2.33E-44	Down	MYOCD
EXOSC7	1338.21255	1.08701042	0.07666812	14.1781277	1.25E-45	2.44E-44	Up	EXOSC7
NUP107	1627.94962	0.92462104	0.06521797	14.177397	1.26E-45	2.47E-44	Up	NUP107
FANCB	78.8530152	1.92560421	0.13583113	14.1764576	1.28E-45	2.50E-44	Up	FANCB
ADH1C	1791.55118	-3.99232652	0.28163472	-14.1755483	1.30E-45	2.53E-44	Down	ADH1C
PLN	430.168769	-3.63307635	0.25629901	-14.175148	1.31E-45	2.54E-44	Down	PLN
EPB41L3	713.865533	-2.6911876	0.18988789	-14.1725075	1.36E-45	2.63E-44	Down	EPB41L3
RPS6KL1	366.470398	1.48413457	0.10477359	14.1651596	1.51E-45	2.92E-44	Up	RPS6KL1
ADCYAP1R1	23.544259	-4.32569865	0.30578037	-14.1464237	1.96E-45	3.81E-44	Down	ADCYAP1R1
WASL	3094.26742	-1.05308085	0.07445276	-14.1442827	2.03E-45	3.92E-44	Down	WASL
PLAU	2335.57203	2.06924329	0.14631037	14.1428341	2.07E-45	4.00E-44	Up	PLAU
HEATR1	1779.75195	1.08270211	0.07656151	14.1415989	2.10E-45	4.06E-44	Up	HEATR1
HMGCLL1	5.88793673	-2.89643147	0.20483048	-14.1406273	2.13E-45	4.12E-44	Down	HMGCLL1
DAAM2	315.543893	-1.91703323	0.13563617	-14.1336436	2.36E-45	4.54E-44	Down	DAAM2
PMAIP1	469.634028	1.95450529	0.13831851	14.1304683	2.46E-45	4.75E-44	Up	PMAIP1

CACNG8	36.5267222	3.59872515	0.25469972	14.129286	2.51E-45	4.82E-44	Up	CACNG8
ANKS6	1549.50526	1.07997115	0.07646843	14.1230977	2.74E-45	5.26E-44	Up	ANKS6
UBE2T	899.172158	1.44529122	0.10239389	14.1150139	3.07E-45	5.89E-44	Up	UBE2T
TMEM59	10176.9429	-1.0807201	0.07657927	-14.1124367	3.18E-45	6.10E-44	Down	TMEM59
SSBP2	225.385823	-2.05393231	0.14556186	-14.110374	3.28E-45	6.28E-44	Down	SSBP2
ITM2A	287.158142	-2.3163336	0.16418399	-14.1081574	3.38E-45	6.47E-44	Down	ITM2A
KCNMA1	728.093146	-3.18655965	0.22588077	-14.1072639	3.43E-45	6.55E-44	Down	KCNMA1
KRT6A	133.352311	5.63254051	0.39929936	14.1060595	3.49E-45	6.65E-44	Up	KRT6A
CCNE1	465.264942	1.65013026	0.11700755	14.1027679	3.65E-45	6.96E-44	Up	CCNE1
SMTN	4495.49937	-1.65322756	0.11723157	-14.1022389	3.68E-45	7.01E-44	Down	SMTN
MROH6	1190.23907	2.36437397	0.16769436	14.0993049	3.84E-45	7.30E-44	Up	MROH6
RIPPLY3	47.862496	3.79162347	0.26892566	14.0991509	3.84E-45	7.31E-44	Up	RIPPLY3
NRG2	10.0911337	-2.99106184	0.21221077	-14.0947694	4.09E-45	7.76E-44	Down	NRG2
KCNIP4	66.5044089	-2.31957706	0.16456971	-14.0947995	4.09E-45	7.76E-44	Down	KCNIP4
HM13	9094.16922	1.29010957	0.0915945	14.0850114	4.70E-45	8.90E-44	Up	HM13
REEP2	66.6714006	-2.50276172	0.17771783	-14.0827835	4.85E-45	9.17E-44	Down	REEP2
ABCB11	19.5303066	-4.54183971	0.32257173	-14.0800921	5.03E-45	9.52E-44	Down	ABCB11
MXD1	2502.97191	-1.74410417	0.12387465	-14.079589	5.07E-45	9.58E-44	Down	MXD1
PRSS56	57.2375044	8.53529679	0.60624707	14.0789082	5.12E-45	9.66E-44	Up	PRSS56
ARID3A	1722.62751	2.27252776	0.16142241	14.0781433	5.18E-45	9.75E-44	Up	ARID3A
TMEM249	22.6266529	3.86804898	0.27476588	14.0776177	5.21E-45	9.81E-44	Up	TMEM249
CCL14	29.7735473	-2.24938296	0.15979399	-14.0767681	5.28E-45	9.92E-44	Down	CCL14
CHMP1B	2175.41191	-0.99773386	0.07088848	-14.0746959	5.43E-45	1.02E-43	Down	CHMP1B
BGN	8874.09785	2.55409156	0.1814765	14.0739516	5.49E-45	1.03E-43	Up	BGN
DSN1	1107.81799	1.22387928	0.08696142	14.0738187	5.50E-45	1.03E-43	Up	DSN1
MGLL	6171.27609	-1.40954789	0.10017286	-14.0711558	5.71E-45	1.07E-43	Down	MGLL
EPHB4	5439.55353	1.22780535	0.08727438	14.0683362	5.95E-45	1.11E-43	Up	EPHB4
JPH2	271.454224	-3.14514339	0.22356561	-14.0680999	5.97E-45	1.11E-43	Down	JPH2
CPED1	380.730287	-2.45334931	0.17444811	-14.0634902	6.37E-45	1.19E-43	Down	CPED1
NEFM	9.26032916	-3.6702535	0.26103947	-14.0601478	6.67E-45	1.24E-43	Down	NEFM
TCF7L1	217.908416	-1.53304579	0.10904134	-14.0593079	6.75E-45	1.26E-43	Down	TCF7L1
HAPLN1	110.484012	-2.47711745	0.17620462	-14.0581871	6.86E-45	1.28E-43	Down	HAPLN1
PSRC1	468.035126	1.33244176	0.09482167	14.0520809	7.48E-45	1.39E-43	Up	PSRC1
ABCD3	2447.72364	-1.07408484	0.07646032	-14.0476117	7.97E-45	1.48E-43	Down	ABCD3
CENPH	524.048957	1.31649648	0.09371763	14.0474791	7.98E-45	1.48E-43	Up	CENPH
NAT9	1250.20476	1.17251469	0.08348463	14.0446768	8.30E-45	1.54E-43	Up	NAT9
ATP1B2	35.8331729	-2.40174341	0.17102732	-14.0430393	8.50E-45	1.57E-43	Down	ATP1B2
CLMN	1958.03294	-1.3269917	0.09451662	-14.0397713	8.90E-45	1.65E-43	Down	CLMN
STX12	1585.39042	-1.03924449	0.07405308	-14.0337783	9.69E-45	1.79E-43	Down	STX12
ZDHHC9	5159.51702	1.2377868	0.08820111	14.0336872	9.70E-45	1.79E-43	Up	ZDHHC9
RAB36	392.177035	1.83673126	0.13088691	14.0329636	9.80E-45	1.81E-43	Up	RAB36
CLEC9A	8.41348384	-2.81979455	0.20096733	-14.0311091	1.01E-44	1.85E-43	Down	CLEC9A
PDCD6IP	6427.16319	-0.81776464	0.05828919	-14.0294401	1.03E-44	1.89E-43	Down	PDCD6IP
TCP11L1	253.117597	-1.13182367	0.08069956	-14.0251525	1.09E-44	2.01E-43	Down	TCP11L1
TUBB4A	24.8889906	-3.37381963	0.24061902	-14.0214173	1.15E-44	2.12E-43	Down	TUBB4A
KCNH8	178.501369	2.54674988	0.18165079	14.020032	1.18E-44	2.16E-43	Up	KCNH8
SCN2B	20.566173	-3.79029974	0.27035347	-14.0197931	1.18E-44	2.16E-43	Down	SCN2B
CCDC68	489.480491	-2.15530687	0.1537733	-14.016132	1.24E-44	2.27E-43	Down	CCDC68
CENPP	205.021916	1.06557145	0.07602951	14.0152354	1.26E-44	2.30E-43	Up	CENPP
TSHZ1	975.174922	-1.26651069	0.09039052	-14.0115439	1.32E-44	2.42E-43	Down	TSHZ1
CBLN2	16.725294	-2.94626064	0.21038625	-14.0040554	1.47E-44	2.69E-43	Down	CBLN2
RBKS	168.894044	-1.26010456	0.08998319	-14.0037775	1.48E-44	2.69E-43	Down	RBKS
NPTN	3566.5293	-0.86588034	0.06184064	-14.0018006	1.52E-44	2.77E-43	Down	NPTN
PGC	56.1266846	4.4121653	0.31514567	14.0003994	1.55E-44	2.82E-43	Up	PGC
SLMAP	2326.79992	-1.26469167	0.09036452	-13.9954457	1.66E-44	3.02E-43	Down	SLMAP
FAP	441.203279	3.06596792	0.21907489	13.9950675	1.67E-44	3.03E-43	Up	FAP
RPP40	305.082208	1.28166965	0.09162017	13.988947	1.82E-44	3.30E-43	Up	RPP40
DSC2	4018.65271	-1.57785023	0.11281785	-13.9858202	1.90E-44	3.45E-43	Down	DSC2

RNASEH2A	1562.12031	1.50652545	0.1077499	13.981688	2.02E-44	3.65E-43	Up	RNASEH2A
TRPV3	70.2681298	-2.92395437	0.20932033	-13.9688023	2.42E-44	4.37E-43	Down	TRPV3
INTS7	1139.4794	0.85150268	0.06098693	13.9620516	2.66E-44	4.80E-43	Up	INTS7
TSPAN1	14060.9822	-2.28082803	0.16337266	-13.9608921	2.70E-44	4.87E-43	Down	TSPAN1
ARHGEF37	462.565066	-1.58950225	0.11387131	-13.9587592	2.78E-44	5.02E-43	Down	ARHGEF37
GABRE	1243.43733	2.44839	0.1754116	13.9579712	2.81E-44	5.07E-43	Up	GABRE
GOLGA7B	328.730679	2.53073427	0.18138085	13.9525994	3.03E-44	5.46E-43	Up	GOLGA7B
RANBP1	3421.53957	1.08453941	0.07775134	13.9488199	3.20E-44	5.75E-43	Up	RANBP1
SLC17A8	7.7531825	-5.30856662	0.38064963	-13.9460705	3.32E-44	5.97E-43	Down	SLC17A8
RRM2	3856.78156	1.36927982	0.09819519	13.9444693	3.40E-44	6.10E-43	Up	RRM2
DLX4	28.547323	2.47789218	0.17769964	13.944272	3.41E-44	6.11E-43	Up	DLX4
RAN	13033.8268	1.0141562	0.07273415	13.9433295	3.45E-44	6.18E-43	Up	RAN
LPCAT3	755.718008	-1.19369399	0.08563199	-13.9398134	3.63E-44	6.49E-43	Down	LPCAT3
PDZD7	55.2570731	2.19195194	0.15733004	13.9321894	4.04E-44	7.21E-43	Up	PDZD7
TMEM185B	940.401656	0.88693308	0.06366471	13.9313147	4.09E-44	7.29E-43	Up	TMEM185B
PALD1	1609.49417	1.94785356	0.13986337	13.9268313	4.35E-44	7.76E-43	Up	PALD1
DBF4B	363.310419	1.20052831	0.08620554	13.9263476	4.38E-44	7.80E-43	Up	DBF4B
EXOSC3	794.257265	0.91730505	0.06586883	13.9262385	4.39E-44	7.81E-43	Up	EXOSC3
CPOX	1047.25087	0.84288954	0.06055065	13.9204055	4.76E-44	8.46E-43	Up	CPOX
UBE3D	218.223557	1.28779054	0.0925175	13.9194259	4.83E-44	8.57E-43	Up	UBE3D
PSPH	897.587863	1.4106088	0.10134652	13.9186703	4.88E-44	8.66E-43	Up	PSPH
CCT2	7685.57194	0.98180938	0.07060465	13.905733	5.85E-44	1.04E-42	Up	CCT2
GDNF	42.1545143	-2.29125116	0.16482623	-13.9010105	6.25E-44	1.11E-42	Down	GDNF
TIGD1	178.425412	1.49659669	0.10767048	13.8997859	6.35E-44	1.12E-42	Up	TIGD1
MSH5	174.289687	2.02692932	0.14583991	13.898317	6.48E-44	1.15E-42	Up	MSH5
MS4A12	1401.09596	-5.32912745	0.38349813	-13.8960978	6.69E-44	1.18E-42	Down	MS4A12
GRIK5	52.608688	-2.93668331	0.21147126	-13.8869146	7.60E-44	1.34E-42	Down	GRIK5
CSNK2A2	2872.64656	0.80663452	0.05808958	13.8860442	7.70E-44	1.36E-42	Up	CSNK2A2
C7orf31	191.170844	-1.66161758	0.11967693	-13.8841928	7.90E-44	1.39E-42	Down	C7orf31
RNASEL	460.834145	-1.26928175	0.09142786	-13.8828769	8.04E-44	1.41E-42	Down	RNASEL
LRP11	2328.6797	1.14738777	0.0826667	13.879686	8.41E-44	1.48E-42	Up	LRP11
EDN3	392.970346	-3.18675542	0.2297212	-13.8722738	9.33E-44	1.64E-42	Down	EDN3
PDE6A	63.2407559	-3.34438112	0.24108401	-13.8722645	9.33E-44	1.64E-42	Down	PDE6A
PKHD1L1	19.6825725	-3.5564411	0.25644949	-13.8679984	9.90E-44	1.73E-42	Down	PKHD1L1
TMPRSS3	496.33953	2.90767055	0.20972644	13.8641108	1.05E-43	1.83E-42	Up	TMPRSS3
CHEK1	986.413756	1.17889838	0.08503595	13.8635293	1.05E-43	1.84E-42	Up	CHEK1
BCL2L2	1349.39299	-0.95662671	0.06901542	-13.8610579	1.09E-43	1.90E-42	Down	BCL2L2
CCNB1	2968.54759	1.45205763	0.10476874	13.8596454	1.11E-43	1.94E-42	Up	CCNB1
CCBE1	74.821052	-3.8447248	0.27742164	-13.8587776	1.13E-43	1.96E-42	Down	CCBE1
XPNPEP3	781.31652	1.12803763	0.08141279	13.8557794	1.17E-43	2.04E-42	Up	XPNPEP3
RCC2	6792.13432	0.84814631	0.06122336	13.8533112	1.21E-43	2.11E-42	Up	RCC2
CDK4	3745.19579	1.21857122	0.08797556	13.8512476	1.25E-43	2.17E-42	Up	CDK4
CA14	25.3615251	-2.1642802	0.15626466	-13.8500941	1.27E-43	2.21E-42	Down	CA14
KAT2B	440.174425	-1.73963446	0.12562562	-13.847768	1.31E-43	2.28E-42	Down	KAT2B
ANO10	1578.59175	-1.05976089	0.07653889	-13.8460446	1.34E-43	2.33E-42	Down	ANO10
ILK	440.32223	-1.35285016	0.09777088	-13.8369442	1.53E-43	2.64E-42	Down	ILK
PRR11	831.624182	1.29825841	0.09383194	13.8359968	1.55E-43	2.67E-42	Up	PRR11
CAV1	1754.31715	-2.14797439	0.15533179	-13.8282989	1.72E-43	2.97E-42	Down	CAV1
PPP1R14A	283.337698	-2.49403172	0.18036393	-13.8277743	1.73E-43	2.99E-42	Down	PPP1R14A
JAG2	1628.17672	2.04726429	0.14807281	13.8260653	1.77E-43	3.06E-42	Up	JAG2
LMO3	176.977008	-3.72583245	0.26948872	-13.8255597	1.79E-43	3.08E-42	Down	LMO3
E2F3	989.94632	0.94210183	0.06815526	13.8228769	1.86E-43	3.19E-42	Up	E2F3
AHCY	17979.1041	1.69105534	0.1224023	13.8155514	2.05E-43	3.53E-42	Up	AHCY
RSPO2	49.9256373	-4.12166212	0.29837436	-13.8137276	2.11E-43	3.62E-42	Down	RSPO2
MFSD11	719.869646	-0.83349473	0.06034783	-13.8115123	2.17E-43	3.73E-42	Down	MFSD11
LPO	30.3152121	4.83774304	0.35029873	13.8103355	2.21E-43	3.79E-42	Up	LPO
MAGEA3	163.21105	11.5131937	0.83368892	13.8099397	2.22E-43	3.80E-42	Up	MAGEA3
ALDH3B2	87.1715538	3.79010424	0.27454159	13.80521	2.37E-43	4.06E-42	Up	ALDH3B2

AOC3	1089.9299	-2.75233397	0.19939445	-13.8034631	2.43E-43	4.15E-42	Down	AOC3
RYR3	48.888635	-2.32591194	0.16856584	-13.7982403	2.61E-43	4.46E-42	Down	RYR3
PIF1	269.641437	1.65354412	0.1198855	13.7926946	2.82E-43	4.81E-42	Up	PIF1
HABP2	67.078309	6.37832768	0.46249022	13.7912704	2.88E-43	4.90E-42	Up	HABP2
ZNF695	58.4835221	2.03034929	0.14726858	13.7867108	3.06E-43	5.22E-42	Up	ZNF695
CHI3L1	1067.00011	2.9155788	0.21151516	13.7842544	3.17E-43	5.39E-42	Up	CHI3L1
NUP85	1702.36089	0.84556106	0.06135911	13.7805299	3.34E-43	5.67E-42	Up	NUP85
PAFAH1B3	1733.90026	1.65603177	0.12017692	13.7799491	3.37E-43	5.71E-42	Up	PAFAH1B3
FOXF2	253.950683	-2.17831828	0.15810807	-13.7774009	3.49E-43	5.91E-42	Down	FOXF2
TADA2B	1039.4878	-0.7013524	0.05091524	-13.7749006	3.61E-43	6.12E-42	Down	TADA2B
CGN	4444.43132	-1.35739957	0.09854732	-13.7740887	3.65E-43	6.18E-42	Down	CGN
URB1	1831.92024	1.03155879	0.07489694	13.7730427	3.70E-43	6.26E-42	Up	URB1
KISS1	31.7667453	3.46479282	0.25158746	13.771723	3.77E-43	6.37E-42	Up	KISS1
SCAMP2	5665.60659	-0.98810618	0.07175053	-13.7714129	3.79E-43	6.39E-42	Down	SCAMP2
SUV39H2	489.664143	1.09726509	0.07971079	13.7655787	4.11E-43	6.93E-42	Up	SUV39H2
PDE7B	56.7609253	-2.1928162	0.15931138	-13.7643412	4.18E-43	7.04E-42	Down	PDE7B
PAQR5	778.44404	-1.86762977	0.1357059	-13.7623329	4.29E-43	7.23E-42	Down	PAQR5
ALKBH2	557.479985	1.220263	0.08870404	13.7565668	4.65E-43	7.82E-42	Up	ALKBH2
LGR5	2653.37323	2.89529406	0.21049563	13.7546519	4.78E-43	8.03E-42	Up	LGR5
SND1	12249.2842	0.99435211	0.07231409	13.750461	5.06E-43	8.50E-42	Up	SND1
PNO1	1140.07024	1.01187973	0.07361023	13.7464544	5.35E-43	8.97E-42	Up	PNO1
DDX21	5917.59975	1.1378116	0.08279144	13.7431063	5.60E-43	9.39E-42	Up	DDX21
SLC22A23	1413.58285	-1.46382406	0.10652332	-13.7418181	5.70E-43	9.55E-42	Down	SLC22A23
ACTR5	601.288993	1.09382825	0.07965939	13.7313154	6.59E-43	1.10E-41	Up	ACTR5
KIF18A	327.204331	1.33776714	0.09747632	13.7240212	7.29E-43	1.22E-41	Up	KIF18A
PANO1	18.9544043	1.91746356	0.13973575	13.722069	7.49E-43	1.25E-41	Up	PANO1
CPSF3	1812.31191	0.79751584	0.05813265	13.7188967	7.82E-43	1.31E-41	Up	CPSF3
HOXB8	1199.41791	2.91702959	0.21269456	13.7146413	8.30E-43	1.38E-41	Up	HOXB8
ITGA8	175.231421	-1.85861109	0.13553818	-13.7128228	8.51E-43	1.42E-41	Down	ITGA8
WDR3	1711.58914	0.93019432	0.067838	13.7119941	8.61E-43	1.43E-41	Up	WDR3
SP8	32.1792989	5.82570457	0.42490747	13.7105252	8.78E-43	1.46E-41	Up	SP8
DNMT3B	225.570875	1.71566457	0.1251615	13.7076064	9.14E-43	1.52E-41	Up	DNMT3B
TRAIP	342.503005	1.26243604	0.09209945	13.7073143	9.18E-43	1.52E-41	Up	TRAIP
FAM135B	12.3892378	-4.15359131	0.30302276	-13.7071924	9.19E-43	1.52E-41	Down	FAM135B
MMP10	144.078669	3.46365724	0.25274155	13.7043443	9.56E-43	1.58E-41	Up	MMP10
DISP2	212.68571	-2.23671645	0.16326651	-13.699787	1.02E-42	1.68E-41	Down	DISP2
EAF2	99.2779166	-1.27405299	0.09300848	-13.6982451	1.04E-42	1.72E-41	Down	EAF2
KRT16	60.0948973	4.79188073	0.34994074	13.6934063	1.11E-42	1.84E-41	Up	KRT16
PLSCR4	579.177233	-1.62199011	0.11846023	-13.6922753	1.13E-42	1.86E-41	Down	PLSCR4
NOP14	2500.99189	0.84372201	0.06162388	13.6914781	1.14E-42	1.88E-41	Up	NOP14
RCAN1	1358.80627	-1.27585867	0.09321992	-13.686546	1.22E-42	2.01E-41	Down	RCAN1
SLC17A9	1928.95196	2.41029546	0.17612669	13.68501	1.25E-42	2.05E-41	Up	SLC17A9
TMEM140	519.994729	-1.20390386	0.08798774	-13.6826315	1.29E-42	2.12E-41	Down	TMEM140
TMOD1	78.0500075	-2.92376228	0.21369219	-13.6821205	1.30E-42	2.13E-41	Down	TMOD1
TNFSF9	316.194565	3.26978296	0.23904904	13.6782935	1.37E-42	2.24E-41	Up	TNFSF9
SNX1	2735.87246	-0.63864414	0.04669336	-13.6774089	1.39E-42	2.27E-41	Down	SNX1
ODF2	1671.17482	0.83409155	0.06099865	13.6739347	1.45E-42	2.38E-41	Up	ODF2
MAOA	4831.38717	-1.99880818	0.14627957	-13.6643013	1.66E-42	2.71E-41	Down	MAOA
IGFL1	17.9926811	7.07671837	0.51798262	13.6620769	1.71E-42	2.80E-41	Up	IGFL1
SPP1	5329.76027	3.85063467	0.28187989	13.6605512	1.75E-42	2.85E-41	Up	SPP1
PPP2R3B	603.463365	1.68756472	0.12355339	13.6585874	1.79E-42	2.93E-41	Up	PPP2R3B
E2F5	628.70538	1.41239627	0.10341155	13.6580122	1.81E-42	2.95E-41	Up	E2F5
CA12	4946.43268	-2.29803417	0.16828311	-13.6557628	1.87E-42	3.04E-41	Down	CA12
MRGPRF	430.790238	-2.60143999	0.19051737	-13.654608	1.90E-42	3.08E-41	Down	MRGPRF
C1QTNF9	5.31056003	-2.74865507	0.20131097	-13.6537767	1.92E-42	3.12E-41	Down	C1QTNF9
RUNDC3B	72.0980375	-2.45611473	0.1799078	-13.6520751	1.96E-42	3.19E-41	Down	RUNDC3B
RAVER2	1535.25368	-1.11190772	0.08145878	-13.649943	2.02E-42	3.28E-41	Down	RAVER2
PIANP	25.2281039	-2.4176737	0.17719497	-13.6441439	2.19E-42	3.55E-41	Down	PIANP

SCUBE2	185.750744	-2.4949878	0.18287193	-13.643361	2.21E-42	3.58E-41	Down	SCUBE2
CSTF1	1631.5996	0.9018533	0.06611414	13.6408545	2.29E-42	3.70E-41	Up	CSTF1
PRKCB	253.622673	-2.46629584	0.18085297	-13.6370219	2.41E-42	3.90E-41	Down	PRKCB
NOX4	88.4909714	2.93113544	0.21513306	13.6247558	2.85E-42	4.61E-41	Up	NOX4
C1orf105	13.5810231	4.24431081	0.31160992	13.6205894	3.02E-42	4.87E-41	Up	C1orf105
SIX1	30.3332904	3.66143114	0.26907751	13.6073474	3.62E-42	5.84E-41	Up	SIX1
PLAC8	2187.70058	-2.97741663	0.21883415	-13.6058134	3.70E-42	5.96E-41	Down	PLAC8
AMELX	13.1570172	5.53605354	0.40710124	13.5987144	4.08E-42	6.56E-41	Up	AMELX
LGI1	8.45208321	-5.16283004	0.37972738	-13.5961491	4.22E-42	6.79E-41	Down	LGI1
TMEM30B	2293.4972	-1.11878694	0.08229455	-13.5949087	4.29E-42	6.90E-41	Down	TMEM30B
TRIM72	155.578211	5.37667077	0.39557701	13.5919697	4.47E-42	7.17E-41	Up	TRIM72
ZNF485	146.753795	1.1450386	0.0842716	13.5874791	4.75E-42	7.62E-41	Up	ZNF485
MPP2	67.7374971	-2.11829569	0.15592928	-13.584977	4.92E-42	7.88E-41	Down	MPP2
CPNE5	234.45816	-1.95262557	0.14382587	-13.576317	5.53E-42	8.86E-41	Down	CPNE5
SLC9A3	1684.06404	-3.58918588	0.26454261	-13.5675153	6.24E-42	9.98E-41	Down	SLC9A3
POLD2	5450.67366	1.31863472	0.09719409	13.5670252	6.28E-42	1.00E-40	Up	POLD2
LEF1	512.2354	2.11885089	0.15617958	13.5667598	6.30E-42	1.01E-40	Up	LEF1
C18orf32	195.125415	-0.99147921	0.07308891	-13.5653858	6.42E-42	1.02E-40	Down	C18orf32
RAD18	866.165533	1.01494614	0.07482397	13.5644525	6.51E-42	1.04E-40	Up	RAD18
C7	626.647154	-3.81672682	0.28138398	-13.5641225	6.54E-42	1.04E-40	Down	C7
P2RX2	5.16818687	-3.89945081	0.28750064	-13.5632769	6.61E-42	1.05E-40	Down	P2RX2
NRXN1	40.0435919	-4.78766169	0.35300786	-13.5624788	6.68E-42	1.06E-40	Down	NRXN1
CNR1	43.5780975	-3.85593381	0.28435963	-13.5600604	6.91E-42	1.10E-40	Down	CNR1
IL10RB	1751.92149	-1.08448976	0.07997683	-13.5600488	6.91E-42	1.10E-40	Down	IL10RB
TRIM59	369.980197	1.29333012	0.09538302	13.5593331	6.98E-42	1.11E-40	Up	TRIM59
NDRG2	2722.93078	-1.39956592	0.1032353	-13.5570474	7.20E-42	1.14E-40	Down	NDRG2
NOP16	1271.20495	1.12374393	0.08289466	13.5562889	7.27E-42	1.15E-40	Up	NOP16
SLC6A20	993.779444	3.30645604	0.24395698	13.5534391	7.56E-42	1.20E-40	Up	SLC6A20
SORBS1	2285.96699	-2.27803817	0.16809998	-13.5516859	7.74E-42	1.22E-40	Down	SORBS1
MCM7	8692.10856	1.29583021	0.09562774	13.5507771	7.84E-42	1.24E-40	Up	MCM7
TRAF5	1950.87256	1.51461868	0.11180211	13.5473175	8.22E-42	1.30E-40	Up	TRAF5
NR2C2AP	954.433941	1.11985583	0.08267084	13.5459598	8.37E-42	1.32E-40	Up	NR2C2AP
SLC9A1	2528.14731	-1.38477102	0.10227316	-13.5399258	9.09E-42	1.43E-40	Down	SLC9A1
RRP1	1612.36965	1.21417723	0.08968089	13.5388629	9.22E-42	1.45E-40	Up	RRP1
TOM1L2	1735.16749	-1.16504048	0.08605612	-13.5381479	9.31E-42	1.46E-40	Down	TOM1L2
SLC7A6	778.631089	1.21421382	0.08972132	13.5331697	9.96E-42	1.56E-40	Up	SLC7A6
VAT1L	90.9209461	-2.08927772	0.15440941	-13.5307667	1.03E-41	1.61E-40	Down	VAT1L
MPPE1	425.110751	-1.05772129	0.07818675	-13.5281393	1.07E-41	1.67E-40	Down	MPPE1
VPS4B	2082.0606	-1.06873665	0.07900675	-13.527157	1.08E-41	1.69E-40	Down	VPS4B
SCNN1B	613.100675	-3.91631704	0.28953509	-13.5262261	1.10E-41	1.71E-40	Down	SCNN1B
ZNF821	80.9505347	-0.98686755	0.07296054	-13.5260447	1.10E-41	1.72E-40	Down	ZNF821
UNC5CL	571.406154	2.15679789	0.15949997	13.5222467	1.16E-41	1.80E-40	Up	UNC5CL
ARMCX1	154.174026	-1.99656277	0.14779477	-13.5090221	1.38E-41	2.16E-40	Down	ARMCX1
CLCA4	3790.00389	-5.34397614	0.39566981	-13.5061509	1.44E-41	2.24E-40	Down	CLCA4
COL1A1	87428.5079	2.66352019	0.19721883	13.5054048	1.45E-41	2.26E-40	Up	COL1A1
SFRP1	249.518934	-4.10873568	0.30430057	-13.5022281	1.52E-41	2.36E-40	Down	SFRP1
TCN2	843.451548	-1.73966017	0.12888344	-13.4979341	1.61E-41	2.50E-40	Down	TCN2
NACAD	58.0700499	-2.13529244	0.15827218	-13.4912681	1.76E-41	2.73E-40	Down	NACAD
FMO5	705.197235	-2.02267829	0.14993236	-13.4906057	1.78E-41	2.76E-40	Down	FMO5
CLEC10A	130.421139	-2.78772064	0.20667383	-13.488503	1.83E-41	2.83E-40	Down	CLEC10A
DUSP4	1605.36749	3.18605147	0.23623383	13.4868553	1.87E-41	2.90E-40	Up	DUSP4
NIFK	1803.84537	0.99232078	0.07358361	13.4856231	1.90E-41	2.94E-40	Up	NIFK
NEURL1B	2113.72473	-1.43211841	0.10620207	-13.4848447	1.92E-41	2.97E-40	Down	NEURL1B
CYP2D7	56.4144633	2.6879782	0.19935071	13.4836648	1.95E-41	3.02E-40	Up	CYP2D7
DTL	800.241713	1.27697726	0.09473369	13.479653	2.06E-41	3.18E-40	Up	DTL
STOX2	38.6704751	-2.44549508	0.18142364	-13.4794728	2.07E-41	3.19E-40	Down	STOX2
SAMD10	571.987245	1.57481177	0.11684455	13.4778371	2.11E-41	3.25E-40	Up	SAMD10
SCN4B	81.8550185	-2.15687848	0.16009281	-13.4726759	2.27E-41	3.49E-40	Down	SCN4B

PTGDR2	76.6032912	-2.44187576	0.18124836	-13.4725399	2.27E-41	3.49E-40	Down	PTGDR2
CDHR5	5575.8661	-2.29233209	0.17014989	-13.4724279	2.27E-41	3.49E-40	Down	CDHR5
OVGP1	206.90116	1.64771753	0.12233985	13.4683628	2.40E-41	3.69E-40	Up	OVGP1
TCEAL7	55.6055762	-2.28830526	0.16991276	-13.4675307	2.43E-41	3.73E-40	Down	TCEAL7
SMYD2	1458.07039	0.85353175	0.06340527	13.4615265	2.63E-41	4.04E-40	Up	SMYD2
PEG3	64.428753	-2.74064257	0.2036225	-13.4594288	2.71E-41	4.15E-40	Down	PEG3
C8orf74	8.50540562	5.98722672	0.44486121	13.4586397	2.74E-41	4.19E-40	Up	C8orf74
CHAD	59.12671	-2.71644278	0.20190326	-13.4541796	2.91E-41	4.45E-40	Down	CHAD
BTNL8	540.472501	-3.18908672	0.23703988	-13.4537984	2.92E-41	4.47E-40	Down	BTNL8
UCN	30.3453488	2.37025779	0.17626938	13.4467925	3.22E-41	4.91E-40	Up	UCN
ZNF200	442.805526	0.84012634	0.06249244	13.4436473	3.35E-41	5.12E-40	Up	ZNF200
HSPB6	546.711758	-3.50922308	0.26105399	-13.4425187	3.41E-41	5.19E-40	Down	HSPB6
CDK1	2104.35923	1.45869523	0.10857362	13.4350802	3.77E-41	5.74E-40	Up	CDK1
BMP2	692.185124	-1.90909733	0.1421803	-13.4272988	4.18E-41	6.37E-40	Down	BMP2
ACKR2	39.8538622	-2.30169592	0.17153325	-13.4183655	4.72E-41	7.18E-40	Down	ACKR2
SLC3A2	6652.12066	1.15443603	0.08606724	13.4131868	5.06E-41	7.69E-40	Up	SLC3A2
MAD2L1	1681.48847	1.39493001	0.10399905	13.4129116	5.08E-41	7.71E-40	Up	MAD2L1
PRC1	2020.56068	1.1580414	0.08636409	13.4088302	5.37E-41	8.14E-40	Up	PRC1
TXNDC11	1947.75882	-0.75672298	0.05643502	-13.4087495	5.37E-41	8.14E-40	Down	TXNDC11
PNPT1	1617.2491	0.99378838	0.0741178	13.4082284	5.41E-41	8.19E-40	Up	PNPT1
TOP2A	5617.04524	1.38451946	0.10326141	13.4079078	5.44E-41	8.22E-40	Up	TOP2A
LIMA1	5805.74936	-1.4405001	0.10744101	-13.4073584	5.48E-41	8.27E-40	Down	LIMA1
RPN2	18504.5763	0.99946574	0.07455804	13.4052036	5.64E-41	8.51E-40	Up	RPN2
CAPN2	8413.82727	-1.16889711	0.08721933	-13.4018125	5.90E-41	8.90E-40	Down	CAPN2
PIWIL1	245.181552	4.52032032	0.33743944	13.3959455	6.39E-41	9.63E-40	Up	PIWIL1
TBC1D1	1950.36202	-1.12159079	0.08373774	-13.3940895	6.55E-41	9.86E-40	Down	TBC1D1
TFAP2A	218.089984	3.23252144	0.24150663	13.3848145	7.42E-41	1.12E-39	Up	TFAP2A
RPUSD4	1184.18934	0.85027285	0.063538	13.3821162	7.69E-41	1.16E-39	Up	RPUSD4
NANP	431.252998	1.26924295	0.0948659	13.3793382	7.99E-41	1.20E-39	Up	NANP
CLIP3	427.852828	-2.00316335	0.14973468	-13.3780856	8.12E-41	1.22E-39	Down	CLIP3
LAMA1	160.83272	-2.59269381	0.19383992	-13.3754376	8.42E-41	1.26E-39	Down	LAMA1
ZC3H8	506.172639	1.09668274	0.08200239	13.3737894	8.60E-41	1.29E-39	Up	ZC3H8
CPNE1	8131.3419	1.82877831	0.1367554	13.3726219	8.74E-41	1.31E-39	Up	CPNE1
PI16	105.289186	-4.33769927	0.32443716	-13.3699211	9.06E-41	1.36E-39	Down	PI16
SORCS1	40.2999975	-3.8073225	0.28480593	-13.3681295	9.28E-41	1.39E-39	Down	SORCS1
ASB7	600.712635	-0.7958703	0.0595573	-13.3631029	9.93E-41	1.48E-39	Down	ASB7
TGIF1	3109.96076	1.04217003	0.07799453	13.362092	1.01E-40	1.50E-39	Up	TGIF1
TMEM63A	7200.98707	1.33340922	0.09979109	13.3620072	1.01E-40	1.50E-39	Up	TMEM63A
BFSP1	104.838409	1.8552399	0.138923	13.3544476	1.12E-40	1.66E-39	Up	BFSP1
HMGA1	17598.801	1.46461503	0.10970687	13.3502579	1.18E-40	1.76E-39	Up	HMGA1
GALNT12	1647.92564	-1.45415538	0.10897789	-13.3435822	1.29E-40	1.92E-39	Down	GALNT12
CFAP45	33.8150844	2.12868594	0.15956053	13.3409303	1.34E-40	1.99E-39	Up	CFAP45
GNA13	2443.5159	-1.06858627	0.08014584	-13.3330228	1.49E-40	2.21E-39	Down	GNA13
TMEM82	108.427593	-3.10215704	0.23270525	-13.3308422	1.53E-40	2.27E-39	Down	TMEM82
CYB561A3	1309.13642	-0.91313602	0.06850177	-13.3301079	1.55E-40	2.29E-39	Down	CYB561A3
TAF1D	2534.45662	1.28636147	0.09652261	13.3270475	1.61E-40	2.39E-39	Up	TAF1D
ACAT1	2053.09741	-1.29590946	0.09725098	-13.3254124	1.65E-40	2.44E-39	Down	ACAT1
MSRB3	843.056554	-2.53197278	0.19006449	-13.321651	1.73E-40	2.56E-39	Down	MSRB3
TSACC	19.5201593	1.98386381	0.1490177	13.3129404	1.95E-40	2.88E-39	Up	TSACC
KRT20	13361.6652	-2.71171147	0.20370474	-13.3119703	1.97E-40	2.91E-39	Down	KRT20
FANCD2	895.455537	1.11451099	0.08373964	13.3092403	2.05E-40	3.02E-39	Up	FANCD2
EPAS1	5091.40169	-1.19616144	0.08990309	-13.3050097	2.16E-40	3.19E-39	Down	EPAS1
SCIN	710.613911	-2.59907373	0.1953548	-13.304376	2.18E-40	3.21E-39	Down	SCIN
PTP4A3	2347.5576	2.36538846	0.17780611	13.3031902	2.22E-40	3.26E-39	Up	PTP4A3
HSF4	373.164761	2.09377215	0.15746157	13.297036	2.41E-40	3.54E-39	Up	HSF4
SPARCL1	3026.85591	-2.34376742	0.17633646	-13.2914512	2.59E-40	3.81E-39	Down	SPARCL1
DSG4	38.6901171	2.21428323	0.16663312	13.2883739	2.70E-40	3.97E-39	Up	DSG4
VAMP2	1222.59429	-1.25674325	0.09461625	-13.2825305	2.92E-40	4.29E-39	Down	VAMP2

PLEKHB2	5691.85587	-0.73093158	0.05503015	-13.2823851	2.93E-40	4.29E-39	Down	PLEKHB2
DNAJC28	23.2646384	-1.53082494	0.11525472	-13.2821023	2.94E-40	4.30E-39	Down	DNAJC28
PLK1	2465.29105	1.46731174	0.11049189	13.2798138	3.03E-40	4.43E-39	Up	PLK1
PGM1	2600.37171	-1.42282519	0.10714913	-13.2789239	3.07E-40	4.48E-39	Down	PGM1
SULT1B1	2588.17426	-2.67054202	0.20124264	-13.2702596	3.44E-40	5.03E-39	Down	SULT1B1
NUP93	2034.07008	0.78960802	0.05952291	13.2656142	3.66E-40	5.35E-39	Up	NUP93
SEMA6A	1041.07337	-2.33385225	0.17600733	-13.2599715	3.95E-40	5.76E-39	Down	SEMA6A
ZNF263	1238.98591	0.71181091	0.05368145	13.2599041	3.95E-40	5.76E-39	Up	ZNF263
NUP155	1660.42666	1.01018894	0.07619621	13.2577329	4.07E-40	5.92E-39	Up	NUP155
B3GALT4	407.363748	-1.45917214	0.11013934	-13.2484193	4.61E-40	6.70E-39	Down	B3GALT4
GTPBP3	803.06399	1.08733242	0.08208068	13.2471176	4.69E-40	6.81E-39	Up	GTPBP3
NIT2	1743.83767	0.95558442	0.07215003	13.2444071	4.86E-40	7.06E-39	Up	NIT2
DUSP9	29.0118888	4.38296238	0.33100296	13.2414596	5.06E-40	7.34E-39	Up	DUSP9
LYNX1	126.668949	-2.60261901	0.19660499	-13.2378072	5.31E-40	7.69E-39	Down	LYNX1
BIRC5	2457.05476	1.42809694	0.10788939	13.2366763	5.39E-40	7.80E-39	Up	BIRC5
CNGA3	19.7180926	-3.80980763	0.28795078	-13.23076	5.83E-40	8.44E-39	Down	CNGA3
ANXA3	3418.08241	1.61087254	0.1217908	13.2265533	6.17E-40	8.92E-39	Up	ANXA3
PCID2	2039.36445	1.07952039	0.08162273	13.2257318	6.23E-40	9.01E-39	Up	PCID2
RIPK1	1441.04895	-0.69074166	0.05223201	-13.2244892	6.34E-40	9.15E-39	Down	RIPK1
EXOSC5	1391.99936	1.47651229	0.11165279	13.2241409	6.37E-40	9.18E-39	Up	EXOSC5
KRT7	341.591706	3.29889229	0.24952656	13.220606	6.67E-40	9.62E-39	Up	KRT7
ABCC2	246.085086	3.50183343	0.26492565	13.2181741	6.89E-40	9.93E-39	Up	ABCC2
CPNE8	235.107137	-2.09431256	0.15844802	-13.217663	6.94E-40	9.99E-39	Down	CPNE8
SLIT3	616.276065	-2.52133799	0.19077239	-13.2164724	7.05E-40	1.01E-38	Down	SLIT3
GPR3	54.5651811	2.18238716	0.16516626	13.2132744	7.36E-40	1.06E-38	Up	GPR3
PSEN1	3409.025	-0.76185689	0.05766022	-13.2128689	7.40E-40	1.06E-38	Down	PSEN1
RAET1L	47.3216402	3.25241261	0.24623547	13.2085462	7.83E-40	1.12E-38	Up	RAET1L
SMYD1	33.9576009	-4.73294237	0.35837915	-13.2065226	8.05E-40	1.15E-38	Down	SMYD1
CRADD	283.306316	-0.95370103	0.07225001	-13.2000131	8.77E-40	1.26E-38	Down	CRADD
ADAMTS2	1591.46521	2.39972524	0.18180185	13.1996744	8.81E-40	1.26E-38	Up	ADAMTS2
SPDYC	25.2675134	3.6559234	0.27697177	13.1996246	8.82E-40	1.26E-38	Up	SPDYC
KLF9	927.182167	-1.89063014	0.14325473	-13.1976802	9.05E-40	1.29E-38	Down	KLF9
TMEM9B	2183.53641	-0.85879555	0.0650805	-13.1958971	9.26E-40	1.32E-38	Down	TMEM9B
CEBPB	2270.80324	1.59745249	0.12115203	13.1855195	1.06E-39	1.52E-38	Up	CEBPB
WDR90	1796.40921	1.67637228	0.12714161	13.1850794	1.07E-39	1.52E-38	Up	WDR90
COPS7B	1351.72241	0.76260014	0.05785708	13.1807576	1.13E-39	1.61E-38	Up	COPS7B
ITGA7	580.197259	-1.96294569	0.14898867	-13.1751344	1.22E-39	1.74E-38	Down	ITGA7
TLCD2	670.082468	-1.45231295	0.11023729	-13.1744249	1.23E-39	1.75E-38	Down	TLCD2
CRYAB	480.138751	-2.44352777	0.18554378	-13.1695485	1.31E-39	1.87E-38	Down	CRYAB
DNAAF5	2445.71047	1.04312341	0.07921886	13.1676149	1.35E-39	1.91E-38	Up	DNAAF5
CLDN16	32.8594906	2.90130417	0.22035998	13.1662025	1.37E-39	1.95E-38	Up	CLDN16
STX1A	401.177509	1.88371375	0.14320311	13.1541397	1.61E-39	2.28E-38	Up	STX1A
JPH4	15.3922441	-2.55511626	0.1943291	-13.1483975	1.74E-39	2.46E-38	Down	JPH4
FAM162A	2521.39517	-1.11408962	0.0847422	-13.1468102	1.78E-39	2.51E-38	Down	FAM162A
BRCA1	936.797316	1.22083606	0.09292313	13.1381288	1.99E-39	2.82E-38	Up	BRCA1
ARHGAP20	41.1732464	-2.56257646	0.19506204	-13.1372383	2.01E-39	2.85E-38	Down	ARHGAP20
AGAP3	2964.57346	1.11565109	0.08494732	13.1334474	2.12E-39	2.99E-38	Up	AGAP3
MCM4	5072.54313	1.19591992	0.09105972	13.1333585	2.12E-39	2.99E-38	Up	MCM4
LIMS2	915.321167	-2.39300855	0.18236471	-13.1221032	2.46E-39	3.47E-38	Down	LIMS2
TNFRSF10B	3012.04355	1.24885201	0.09518768	13.1198914	2.53E-39	3.57E-38	Up	TNFRSF10B
PPARGC1B	682.805022	-1.3196478	0.10067361	-13.1081799	2.96E-39	4.16E-38	Down	PPARGC1B
NUP205	2748.29223	0.8187528	0.06246523	13.107336	2.99E-39	4.20E-38	Up	NUP205
SNAP29	1188.39973	-0.5992016	0.04571841	-13.1063513	3.03E-39	4.26E-38	Down	SNAP29
TNFRSF13B	23.0765672	-3.28844064	0.25092756	-13.1051392	3.08E-39	4.32E-38	Down	TNFRSF13B
DPF3	69.7881634	-2.31733113	0.17683462	-13.1045101	3.10E-39	4.35E-38	Down	DPF3
AKAP6	122.576935	-2.22188301	0.1695734	-13.1027802	3.17E-39	4.45E-38	Down	AKAP6
TMEFF2	6.14462554	-4.04026159	0.30842024	-13.0998586	3.30E-39	4.62E-38	Down	TMEFF2
CTNND2	12.2218871	-3.36164096	0.25662871	-13.0992395	3.33E-39	4.65E-38	Down	CTNND2

VTI1B	2099.27059	-0.73557003	0.05615658	-13.0985553	3.36E-39	4.69E-38	Down	VTI1B
CPEB3	187.691145	-1.32426858	0.10111986	-13.0960285	3.47E-39	4.85E-38	Down	CPEB3
NUP62CL	173.226605	1.55415356	0.11875421	13.0871449	3.90E-39	5.45E-38	Up	NUP62CL
IPO5	8634.91894	1.02158303	0.07806886	13.0856654	3.98E-39	5.55E-38	Up	IPO5
EBPL	1649.18942	1.16113454	0.08874612	13.0837783	4.08E-39	5.68E-38	Up	EBPL
LY6G6C	50.8477774	3.27314223	0.2502185	13.0811362	4.22E-39	5.88E-38	Up	LY6G6C
FOLR1	223.301782	4.37169325	0.33422463	13.0801048	4.28E-39	5.96E-38	Up	FOLR1
ITIH5	800.60646	-2.07588603	0.15871516	-13.0793181	4.32E-39	6.01E-38	Down	ITIH5
IGSF3	975.922206	-1.21872114	0.09318061	-13.0791289	4.33E-39	6.02E-38	Down	IGSF3
TIMELESS	1736.78281	1.01361593	0.07751251	13.0768039	4.47E-39	6.21E-38	Up	TIMELESS
FLNA	30811.2236	-2.23232952	0.17072875	-13.0752996	4.56E-39	6.32E-38	Down	FLNA
MCM2	3381.35901	1.31583879	0.10064441	13.0741363	4.63E-39	6.42E-38	Up	MCM2
TKT	15813.0532	1.17427721	0.08981859	13.0738774	4.64E-39	6.43E-38	Up	TKT
ARHGEF26	208.799431	-1.89660297	0.14525615	-13.0569547	5.80E-39	8.02E-38	Down	ARHGEF26
PSMD14	2942.78979	0.85369286	0.06538215	13.0569716	5.80E-39	8.02E-38	Up	PSMD14
SHB	835.792744	1.19467925	0.09152839	13.0525543	6.15E-39	8.49E-38	Up	SHB
MAGEB17	86.7172808	5.44331697	0.41712786	13.0495165	6.40E-39	8.83E-38	Up	MAGEB17
GPBP1L1	4472.73277	-0.68509737	0.05250766	-13.0475699	6.56E-39	9.06E-38	Down	GPBP1L1
RPL7L1	4239.24477	0.75483007	0.05789659	13.0375559	7.48E-39	1.03E-37	Up	RPL7L1
ANGPTL1	131.030202	-4.03631165	0.30960571	-13.0369419	7.54E-39	1.04E-37	Down	ANGPTL1
PRR19	48.843197	1.569671	0.12041882	13.0350969	7.73E-39	1.06E-37	Up	PRR19
FIGNL1	653.946083	1.16042137	0.08903006	13.0340405	7.83E-39	1.08E-37	Up	FIGNL1
TAF1A	205.436042	1.23235228	0.09458068	13.0296411	8.30E-39	1.14E-37	Up	TAF1A
PPEF1	14.8029107	2.79354294	0.21444563	13.0268121	8.61E-39	1.18E-37	Up	PPEF1
SALL2	82.4171391	-2.27855848	0.17491663	-13.02654	8.64E-39	1.19E-37	Down	SALL2
MAB21L1	6.5686863	-3.41891644	0.26255508	-13.0217113	9.21E-39	1.26E-37	Down	MAB21L1
P2RX1	85.6568863	-2.17167589	0.16680477	-13.0192671	9.51E-39	1.30E-37	Down	P2RX1
DNASE1L2	41.4176579	2.53506131	0.19477673	13.0152164	1.00E-38	1.37E-37	Up	DNASE1L2
RELL2	128.776656	2.12396428	0.16322312	13.0126435	1.04E-38	1.42E-37	Up	RELL2
LGR4	2598.90018	-1.26269401	0.09707004	-13.0080708	1.10E-38	1.51E-37	Down	LGR4
PTK7	3406.10331	2.09698009	0.16125248	13.0043275	1.16E-38	1.58E-37	Up	PTK7
DSC3	495.298412	4.30345907	0.33096231	13.0028677	1.18E-38	1.61E-37	Up	DSC3
GRINA	8461.16915	1.48411439	0.11419049	12.9968303	1.28E-38	1.74E-37	Up	GRINA
SLC23A1	117.778759	-2.73092403	0.21012349	-12.9967572	1.28E-38	1.74E-37	Down	SLC23A1
ARHGEF39	544.257796	1.24610396	0.09588352	12.9960186	1.29E-38	1.76E-37	Up	ARHGEF39
BMP7	1484.90052	3.19209262	0.24565457	12.9942328	1.32E-38	1.80E-37	Up	BMP7
OGFOD1	1397.54867	0.6049259	0.04656256	12.9916808	1.36E-38	1.86E-37	Up	OGFOD1
UBAP2L	5266.72777	0.68820548	0.05297826	12.9903386	1.39E-38	1.89E-37	Up	UBAP2L
UNC93A	156.530586	3.88509542	0.29908281	12.9900327	1.39E-38	1.89E-37	Up	UNC93A
SLCO4C1	28.4781915	-3.49567098	0.26931368	-12.9799234	1.59E-38	2.16E-37	Down	SLCO4C1
NOC3L	733.358816	1.08736791	0.08379713	12.9761954	1.67E-38	2.26E-37	Up	NOC3L
CDC25C	333.313015	1.20371468	0.09279526	12.9717259	1.77E-38	2.40E-37	Up	CDC25C
FBXO8	570.319864	-0.80787889	0.06228038	-12.971644	1.77E-38	2.40E-37	Down	FBXO8
CMTM7	728.644272	1.47400266	0.1136693	12.9674652	1.87E-38	2.53E-37	Up	CMTM7
DENND1C	1015.48134	-1.06712527	0.08230356	-12.9657245	1.91E-38	2.59E-37	Down	DENND1C
ANKRD35	37.2340606	-1.93351041	0.14912877	-12.9653752	1.92E-38	2.60E-37	Down	ANKRD35
IFITM1	10188.2792	1.8730505	0.14449729	12.96253	2.00E-38	2.69E-37	Up	IFITM1
HSPH1	7083.95405	1.61477308	0.12460896	12.9587238	2.10E-38	2.83E-37	Up	HSPH1
CNTN2	18.8571507	-3.84873318	0.29709787	-12.9544288	2.22E-38	2.99E-37	Down	CNTN2
FGA	37.0274989	5.00146047	0.38623346	12.9493196	2.37E-38	3.19E-37	Up	FGA
C1QTNF2	47.2394962	-1.69704643	0.13105758	-12.9488611	2.38E-38	3.21E-37	Down	C1QTNF2
SLC16A12	5.02530741	-2.75950755	0.21317917	-12.9445461	2.52E-38	3.39E-37	Down	SLC16A12
STYK1	386.128893	-1.72485072	0.13325308	-12.9441714	2.53E-38	3.41E-37	Down	STYK1
NT5DC2	2568.9745	1.7095161	0.13207369	12.9436532	2.55E-38	3.43E-37	Up	NT5DC2
EXOSC8	1256.60188	1.119892	0.08652471	12.9430307	2.57E-38	3.45E-37	Up	EXOSC8
PDE5A	872.225285	-1.95462559	0.1510227	-12.9425945	2.59E-38	3.47E-37	Down	PDE5A
MCM6	2522.15392	0.93334428	0.07212141	12.941293	2.63E-38	3.53E-37	Up	MCM6
WBP1L	1797.90298	-0.74853811	0.0578511	-12.939046	2.71E-38	3.63E-37	Down	WBP1L

CAPN10	897.194988	0.95747755	0.07403314	12.9330944	2.93E-38	3.92E-37	Up	CAPN10
MYO1A	3148.40611	-1.92309903	0.14872375	-12.9306789	3.02E-38	4.04E-37	Down	MYO1A
CASD1	733.883341	-1.3353061	0.10332533	-12.9233183	3.32E-38	4.44E-37	Down	CASD1
ADAMTS1	904.78667	-2.02741217	0.15691345	-12.920576	3.45E-38	4.60E-37	Down	ADAMTS1
CKS2	1979.04755	1.44312212	0.11170406	12.9191552	3.51E-38	4.68E-37	Up	CKS2
SLAMF9	17.1249684	3.64122858	0.28208753	12.9081516	4.05E-38	5.40E-37	Up	SLAMF9
CLPB	676.00113	1.12409336	0.08709454	12.9065879	4.13E-38	5.50E-37	Up	CLPB
CA4	1660.03362	-4.70170614	0.36432108	-12.9053914	4.20E-38	5.59E-37	Down	CA4
IDH3A	1170.87638	-0.93303014	0.07231064	-12.9030814	4.32E-38	5.75E-37	Down	IDH3A
PERP	16368.7532	1.26400186	0.09800123	12.897816	4.63E-38	6.15E-37	Up	PERP
SUGCT	108.092392	-1.98906846	0.1542227	-12.8973781	4.66E-38	6.18E-37	Down	SUGCT
P2RX4	1500.69667	-1.27008432	0.09849669	-12.8946908	4.82E-38	6.40E-37	Down	P2RX4
CHODL	23.5829055	-3.10476595	0.2408481	-12.8909715	5.06E-38	6.71E-37	Down	CHODL
EVPL	4300.31481	1.77738846	0.13794732	12.8845448	5.50E-38	7.29E-37	Up	EVPL
HS6ST2	534.578125	3.3927425	0.26341198	12.8799855	5.83E-38	7.72E-37	Up	HS6ST2
EPB41L4B	3750.29899	-1.29774209	0.10077528	-12.8775839	6.02E-38	7.96E-37	Down	EPB41L4B
TMPRSS2	7596.61482	-1.37830767	0.10706032	-12.8741222	6.30E-38	8.32E-37	Down	TMPRSS2
CHST4	113.966908	5.12337166	0.39797508	12.8735992	6.34E-38	8.37E-37	Up	CHST4
S100P	8494.62978	2.30997604	0.17944707	12.8727432	6.41E-38	8.46E-37	Up	S100P
DONSON	488.626766	0.95576584	0.07425691	12.8710695	6.55E-38	8.64E-37	Up	DONSON
RCSD1	305.093932	-1.72414888	0.13396409	-12.8702313	6.62E-38	8.73E-37	Down	RCSD1
ANPEP	7878.7168	-3.79092947	0.29456754	-12.8694747	6.69E-38	8.81E-37	Down	ANPEP
SMIM6	115.55203	-1.77682091	0.13807546	-12.8684769	6.77E-38	8.91E-37	Down	SMIM6
MPC1	1269.18779	-1.23054464	0.09563634	-12.8669145	6.91E-38	9.09E-37	Down	MPC1
DNA2	405.166011	1.3487412	0.1048265	12.8664143	6.96E-38	9.14E-37	Up	DNA2
HSD3B2	66.430848	-5.72814167	0.44526566	-12.8645484	7.13E-38	9.36E-37	Down	HSD3B2
PDCD5	2390.71549	1.06086042	0.08247316	12.8630996	7.26E-38	9.52E-37	Up	PDCD5
SMARCC1	6382.14988	0.73490528	0.05713269	12.8631312	7.26E-38	9.52E-37	Up	SMARCC1
RNF11	1825.47921	-0.98705475	0.07673762	-12.8627236	7.30E-38	9.56E-37	Down	RNF11
SPEG	318.921408	-3.00760564	0.23386468	-12.8604528	7.51E-38	9.84E-37	Down	SPEG
SETBP1	212.831105	-2.16439491	0.16831438	-12.8592393	7.63E-38	9.99E-37	Down	SETBP1
PRKACB	1936.58284	-1.9939779	0.15507872	-12.8578433	7.77E-38	1.02E-36	Down	PRKACB
MMP28	406.297524	-2.66401177	0.20725211	-12.8539668	8.17E-38	1.07E-36	Down	MMP28
ADAMTSL1	163.552328	-2.11555237	0.16461545	-12.8514813	8.44E-38	1.10E-36	Down	ADAMTSL1
LBX2	41.7076209	1.7779069	0.1383614	12.8497321	8.63E-38	1.13E-36	Up	LBX2
ZIK1	31.7312007	-1.76906563	0.13770886	-12.8464184	9.01E-38	1.17E-36	Down	ZIK1
SPOCD1	93.7076512	2.17139331	0.16908763	12.8418225	9.56E-38	1.25E-36	Up	SPOCD1
PDRG1	1315.71553	1.4030741	0.10928225	12.8389931	9.92E-38	1.29E-36	Up	PDRG1
ATP6V1E2	173.274998	1.12041116	0.0872754	12.8376517	1.01E-37	1.31E-36	Up	ATP6V1E2
ACADM	1390.2491	-1.32793698	0.1034674	-12.8343516	1.05E-37	1.37E-36	Down	ACADM
POLR1E	929.599836	0.86421645	0.06735438	12.830888	1.10E-37	1.43E-36	Up	POLR1E
DNAJC2	1405.24886	1.14912565	0.08959401	12.8259204	1.17E-37	1.52E-36	Up	DNAJC2
CAMKV	45.9120082	4.39945884	0.34301456	12.8258663	1.17E-37	1.52E-36	Up	CAMKV
KIFC1	1730.22328	1.22031818	0.09519205	12.8195394	1.27E-37	1.65E-36	Up	KIFC1
PTGER4	946.413916	-1.55932243	0.12165463	-12.817617	1.31E-37	1.69E-36	Down	PTGER4
MND1	218.034317	1.41003607	0.11001099	12.8172296	1.31E-37	1.70E-36	Up	MND1
HGH1	1843.40633	1.39276241	0.10870233	12.8126266	1.39E-37	1.80E-36	Up	HGH1
BCAS1	1823.89482	-2.48431694	0.19394652	-12.8092885	1.45E-37	1.88E-36	Down	BCAS1
PTK2B	1951.74753	-1.25415542	0.0979479	-12.804311	1.55E-37	2.00E-36	Down	PTK2B
STIL	821.738777	1.1983311	0.09360093	12.802555	1.59E-37	2.05E-36	Up	STIL
AHNAK	18444.0416	-1.72777801	0.13495539	-12.8025861	1.59E-37	2.05E-36	Down	AHNAK
SIGMAR1	5337.91769	1.29760248	0.10135906	12.8020372	1.60E-37	2.06E-36	Up	SIGMAR1
BTBD3	1403.22491	-1.14063561	0.08910077	-12.8016354	1.61E-37	2.07E-36	Down	BTBD3
KIF24	302.923378	1.09074059	0.08520294	12.8016783	1.60E-37	2.07E-36	Up	KIF24
ZNF280C	215.047148	1.34212759	0.10486937	12.7980897	1.68E-37	2.16E-36	Up	ZNF280C
SSTR5	62.6645559	4.30331892	0.3363175	12.7954059	1.74E-37	2.23E-36	Up	SSTR5
GLYATL1	87.2840302	3.21880808	0.25158366	12.7941859	1.77E-37	2.27E-36	Up	GLYATL1
RAB9B	45.5767943	-2.32902639	0.18204095	-12.7939697	1.77E-37	2.27E-36	Down	RAB9B

SPC25	394.797331	1.16340957	0.09096019	12.790316	1.86E-37	2.38E-36	Up	SPC25
SERPINB5	2302.58799	3.10979318	0.24317641	12.788219	1.91E-37	2.44E-36	Up	SERPINB5
ASB9	301.873203	2.1415939	0.16759537	12.7783598	2.17E-37	2.77E-36	Up	ASB9
PDCD11	2512.30779	0.94676713	0.07410674	12.7757222	2.24E-37	2.87E-36	Up	PDCD11
ZBTB12	345.026712	1.53606623	0.12023613	12.7754126	2.25E-37	2.88E-36	Up	ZBTB12
PAMR1	200.132915	-1.69356766	0.13263956	-12.7681937	2.47E-37	3.15E-36	Down	PAMR1
CDKN2A	339.371183	3.01018893	0.23578621	12.7666032	2.52E-37	3.22E-36	Up	CDKN2A
GIMAP7	184.796198	-1.7832935	0.13969939	-12.76522	2.56E-37	3.27E-36	Down	GIMAP7
NOVA1	40.901856	-3.10888614	0.24357588	-12.7635224	2.62E-37	3.34E-36	Down	NOVA1
RBM20	23.1604698	-2.81233285	0.22049647	-12.7545481	2.94E-37	3.75E-36	Down	RBM20
C20orf144	14.2645309	2.19767945	0.17232137	12.7533772	2.99E-37	3.80E-36	Up	C20orf144
PASK	637.675524	1.07668817	0.08444313	12.750453	3.10E-37	3.94E-36	Up	PASK
CCNB1IP1	1842.40692	1.12065244	0.08789248	12.7502659	3.11E-37	3.95E-36	Up	CCNB1IP1
MUSK	10.4367195	-3.57123748	0.28009863	-12.7499285	3.12E-37	3.96E-36	Down	MUSK
TBX15	31.8184388	3.09825143	0.24312521	12.7434395	3.39E-37	4.30E-36	Up	TBX15
ELAVL3	4.06858803	-3.45371732	0.27106797	-12.7411487	3.49E-37	4.43E-36	Down	ELAVL3
TH	48.5792411	3.43598488	0.26976382	12.7370113	3.68E-37	4.67E-36	Up	TH
GNB1L	381.968138	1.56659152	0.12300176	12.7363347	3.71E-37	4.70E-36	Up	GNB1L
CAPN5	6008.91509	-1.36663241	0.10730495	-12.735968	3.73E-37	4.72E-36	Down	CAPN5
GDPD3	413.578014	-2.08600697	0.16391812	-12.7259086	4.25E-37	5.37E-36	Down	GDPD3
CEND1	12.6060009	-2.32369204	0.18263246	-12.7233245	4.39E-37	5.55E-36	Down	CEND1
FGB	46.0190496	5.72064271	0.44972732	12.7202473	4.56E-37	5.76E-36	Up	FGB
MGAT4A	2211.14747	-1.25521493	0.09875121	-12.7108816	5.15E-37	6.49E-36	Down	MGAT4A
MIER1	1570.56987	-1.26377051	0.09943402	-12.7096394	5.23E-37	6.59E-36	Down	MIER1
HELLS	914.955524	1.51612044	0.1193508	12.7030602	5.69E-37	7.17E-36	Up	HELLS
ABCC1	2972.46911	1.04767894	0.08247579	12.7028666	5.70E-37	7.18E-36	Up	ABCC1
POLD4	916.700424	-1.08468195	0.08539052	-12.7026035	5.72E-37	7.20E-36	Down	POLD4
RP9	338.578448	0.9217007	0.07256318	12.7020443	5.76E-37	7.24E-36	Up	RP9
ARHGAP17	2180.3808	-0.70523723	0.05552408	-12.7014672	5.80E-37	7.29E-36	Down	ARHGAP17
P3H1	944.075881	1.20787579	0.0951153	12.6990693	5.98E-37	7.51E-36	Up	P3H1
ONECUT3	97.3745822	6.37055935	0.50174588	12.6967845	6.16E-37	7.73E-36	Up	ONECUT3
JAM3	517.141097	-1.57456855	0.12406126	-12.6918636	6.56E-37	8.23E-36	Down	JAM3
GSDMC	13.7652961	3.09528976	0.24427888	12.6711313	8.55E-37	1.07E-35	Up	GSDMC
ZNF536	21.3061795	-2.87121591	0.22662689	-12.6693522	8.74E-37	1.09E-35	Down	ZNF536
ZSWIM6	645.464656	-1.03929721	0.08205213	-12.6663035	9.09E-37	1.14E-35	Down	ZSWIM6
NECAB1	38.7191052	-2.96620591	0.23423203	-12.6635365	9.42E-37	1.18E-35	Down	NECAB1
SLC6A19	295.382225	-5.15305016	0.40692486	-12.6633949	9.43E-37	1.18E-35	Down	SLC6A19
ACBD7	36.1225224	2.05439311	0.16223116	12.6633696	9.44E-37	1.18E-35	Up	ACBD7
MUC6	229.46692	5.24428046	0.41413372	12.6632539	9.45E-37	1.18E-35	Up	MUC6
TCF24	8.58727561	4.52760839	0.35766041	12.6589587	9.98E-37	1.24E-35	Up	TCF24
PABPC1	68653.5241	1.03103809	0.08146846	12.6556722	1.04E-36	1.30E-35	Up	PABPC1
MARCKS	6326.60097	-1.16895556	0.0923907	-12.6523081	1.09E-36	1.35E-35	Down	MARCKS
PCP4L1	26.9611444	-3.81420326	0.30148245	-12.6514936	1.10E-36	1.37E-35	Down	PCP4L1
IPO4	235.996277	1.31063013	0.1036059	12.6501502	1.12E-36	1.39E-35	Up	IPO4
MTERF3	677.176283	1.10827035	0.08764746	12.644637	1.20E-36	1.49E-35	Up	MTERF3
MRPL3	4083.30054	0.77995674	0.06168846	12.6434793	1.22E-36	1.51E-35	Up	MRPL3
ASIC1	624.59004	1.73662289	0.1373653	12.64237	1.23E-36	1.53E-35	Up	ASIC1
POLR1A	2925.62524	0.84359608	0.06674648	12.6388106	1.29E-36	1.60E-35	Up	POLR1A
DMRTA2	58.8567786	5.18283202	0.41021201	12.6345205	1.36E-36	1.69E-35	Up	DMRTA2
ASGR1	165.989853	2.6518915	0.20997026	12.6298432	1.45E-36	1.79E-35	Up	ASGR1
MEST	2616.35735	1.03936199	0.08231186	12.6271238	1.50E-36	1.85E-35	Up	MEST
ZDHHC7	2438.31729	-0.65876379	0.0521839	-12.6238888	1.56E-36	1.93E-35	Down	ZDHHC7
CES2	8637.2525	-2.00453078	0.15879295	-12.6235503	1.57E-36	1.94E-35	Down	CES2
APELA	11.8630255	2.99694494	0.23742983	12.6224448	1.59E-36	1.96E-35	Up	APELA
TTLL6	89.3836777	-2.24643722	0.1780724	-12.6153027	1.74E-36	2.15E-35	Down	TTLL6
TNFRSF17	77.8093072	-3.27650315	0.25974078	-12.614512	1.76E-36	2.17E-35	Down	TNFRSF17
NIPAL1	977.316545	-1.32749206	0.10531665	-12.6047693	1.99E-36	2.45E-35	Down	NIPAL1
E2F6	374.542048	0.72838895	0.05778657	12.6048145	1.99E-36	2.45E-35	Up	E2F6

KHDRBS2	3.21907039	-4.25136329	0.33731828	-12.603418	2.02E-36	2.49E-35	Down	KHDRBS2
LYPD8	2060.24762	-3.59006329	0.2848716	-12.6023909	2.05E-36	2.52E-35	Down	LYPD8
SOS2	1240.8721	-1.04769594	0.08313518	-12.6023175	2.05E-36	2.52E-35	Down	SOS2
SERINC1	4421.99296	-0.94728044	0.07518486	-12.5993509	2.13E-36	2.61E-35	Down	SERINC1
PALLD	4551.66172	-1.66737596	0.13233774	-12.599399	2.13E-36	2.61E-35	Down	PALLD
SPRR2D	21.5400318	6.71533462	0.53302633	12.5985044	2.15E-36	2.64E-35	Up	SPRR2D
GPT	577.394562	-2.79104993	0.22158729	-12.5957133	2.23E-36	2.73E-35	Down	GPT
TONSL	1387.0722	1.43502074	0.11394225	12.594281	2.27E-36	2.78E-35	Up	TONSL
PTTG1	1430.26216	1.31369489	0.10431191	12.5939109	2.28E-36	2.79E-35	Up	PTTG1
TMEM161A	1717.17508	1.35563906	0.1076552	12.5924162	2.32E-36	2.84E-35	Up	TMEM161A
ALDH4A1	1014.48075	1.88919593	0.15002634	12.5924285	2.32E-36	2.84E-35	Up	ALDH4A1
CXCL8	3542.73406	3.37973328	0.268401	12.5921041	2.33E-36	2.85E-35	Up	CXCL8
DCLK1	89.111676	-2.82138525	0.22408209	-12.5908558	2.37E-36	2.89E-35	Down	DCLK1
YEATS2	1493.62909	0.79364498	0.06303348	12.5908482	2.37E-36	2.89E-35	Up	YEATS2
MMRN1	175.618689	-3.22923133	0.25651303	-12.5889564	2.43E-36	2.96E-35	Down	MMRN1
SUSD6	2409.02392	-1.01091673	0.08034164	-12.5827253	2.63E-36	3.20E-35	Down	SUSD6
GNPDA1	2295.80768	0.95031169	0.07553064	12.5818035	2.66E-36	3.24E-35	Up	GNPDA1
ZP3	477.078796	1.8566272	0.14758442	12.5801032	2.72E-36	3.30E-35	Up	ZP3
GRPEL2	869.03603	1.0547786	0.08386136	12.5776468	2.80E-36	3.41E-35	Up	GRPEL2
CCDC13	14.1328346	-1.78505819	0.14197273	-12.5732467	2.96E-36	3.60E-35	Down	CCDC13
SPRY4	1845.91764	1.05947645	0.08430917	12.5665622	3.22E-36	3.91E-35	Up	SPRY4
SHE	124.960485	-1.81366344	0.14433265	-12.5658571	3.25E-36	3.95E-35	Down	SHE
HTR7	10.8574576	-2.43872039	0.19408276	-12.565363	3.27E-36	3.97E-35	Down	HTR7
MPEG1	957.850516	-2.05562212	0.16361818	-12.563531	3.35E-36	4.06E-35	Down	MPEG1
CKAP5	4469.19375	0.784388	0.06243729	12.5628127	3.38E-36	4.09E-35	Up	CKAP5
DDX11	1104.89641	1.24512602	0.09911652	12.5622453	3.41E-36	4.12E-35	Up	DDX11
HMOX1	1085.59401	-1.57995235	0.12578662	-12.560576	3.48E-36	4.21E-35	Down	HMOX1
FOXM1	2175.03258	1.34555975	0.10713546	12.5594247	3.53E-36	4.26E-35	Up	FOXM1
CTSS	8634.95038	-1.17806241	0.09383731	-12.5543076	3.76E-36	4.55E-35	Down	CTSS
CCNDBP1	1291.50029	-0.89775513	0.0715184	-12.5527858	3.84E-36	4.63E-35	Down	CCNDBP1
ARPIN	1499.41707	-0.95039263	0.07575685	-12.5453034	4.22E-36	5.09E-35	Down	ARPIN
TUBAL3	298.030179	-2.52991501	0.20171216	-12.5422037	4.39E-36	5.29E-35	Down	TUBAL3
BTD	547.920013	-0.96363083	0.07686081	-12.5373495	4.66E-36	5.62E-35	Down	BTD
CPA4	12.4147605	3.71974272	0.29675966	12.5345295	4.83E-36	5.81E-35	Up	CPA4
ELAVL1	3323.3031	0.54312191	0.04334557	12.5300439	5.11E-36	6.15E-35	Up	ELAVL1
FRMPD4	6.8995878	-4.73110021	0.37764627	-12.5278617	5.26E-36	6.32E-35	Down	FRMPD4
NPRL3	1836.82021	1.14814271	0.09165583	12.5266738	5.34E-36	6.41E-35	Up	NPRL3
MAGI3	877.161683	-1.10587262	0.08828325	-12.5264151	5.35E-36	6.42E-35	Down	MAGI3
SRM	3199.20704	1.49915005	0.11969749	12.5244907	5.48E-36	6.58E-35	Up	SRM
NUTF2	4470.54248	1.05594963	0.08431596	12.5237222	5.54E-36	6.64E-35	Up	NUTF2
C12orf73	292.960813	0.95027461	0.07589138	12.5215096	5.69E-36	6.82E-35	Up	C12orf73
MYADML2	89.0899256	3.28567909	0.26241425	12.5209629	5.73E-36	6.86E-35	Up	MYADML2
ANGPTL5	2.21859326	-3.51628815	0.28088963	-12.5183979	5.92E-36	7.08E-35	Down	ANGPTL5
CLDN14	52.770605	3.17520101	0.25365159	12.5179621	5.95E-36	7.12E-35	Up	CLDN14
MYOT	15.0104741	-3.72658733	0.29782803	-12.5125475	6.37E-36	7.61E-35	Down	MYOT
DAO	15.168815	-4.7308266	0.37810684	-12.5118778	6.43E-36	7.67E-35	Down	DAO
CADM2	16.7993192	-4.5208899	0.36153302	-12.5047774	7.03E-36	8.39E-35	Down	CADM2
FOXN3	1596.43451	-1.26354431	0.10105456	-12.5035859	7.14E-36	8.51E-35	Down	FOXN3
PAEP	12.3854402	5.12648924	0.41005314	12.5020119	7.28E-36	8.67E-35	Up	PAEP
KLHL17	282.575552	1.7347341	0.13876276	12.5014382	7.33E-36	8.73E-35	Up	KLHL17
NEK5	116.322775	2.21997559	0.17761581	12.4987502	7.58E-36	9.02E-35	Up	NEK5
GIMAP1	122.069556	-1.66770459	0.13348034	-12.4940094	8.05E-36	9.57E-35	Down	GIMAP1
ACP6	897.289552	0.96425328	0.0772123	12.4883381	8.64E-36	1.03E-34	Up	ACP6
ABCA9	47.5426252	-2.94723689	0.23604925	-12.4856861	8.94E-36	1.06E-34	Down	ABCA9
CYBRD1	1729.2987	-2.08437858	0.16695632	-12.484574	9.06E-36	1.08E-34	Down	CYBRD1
PAG1	1108.86133	-1.69515318	0.13578966	-12.483669	9.17E-36	1.09E-34	Down	PAG1
TGS1	1259.59012	0.987094	0.07907991	12.4822345	9.33E-36	1.11E-34	Up	TGS1
KIF13B	3050.45757	-1.29716003	0.10392573	-12.481606	9.41E-36	1.11E-34	Down	KIF13B

ASNS	2053.26124	1.41906821	0.11374309	12.4760832	1.01E-35	1.19E-34	Up	ASNS
TMPRSS5	72.2023195	3.36761436	0.26992676	12.4760298	1.01E-35	1.19E-34	Up	TMPRSS5
ANKS1B	13.3808348	-3.05653779	0.24500182	-12.4755717	1.01E-35	1.20E-34	Down	ANKS1B
ZNF202	486.393388	0.74697818	0.05987706	12.4751988	1.02E-35	1.20E-34	Up	ZNF202
MRPL35	1766.86531	-0.77745774	0.06232443	-12.4743661	1.03E-35	1.22E-34	Down	MRPL35
DESI1	2116.21947	0.81331766	0.06526288	12.4621782	1.20E-35	1.42E-34	Up	DESI1
DACH1	1245.90689	2.18667953	0.17550764	12.4591697	1.25E-35	1.47E-34	Up	DACH1
CROT	497.200823	-1.24537102	0.09997282	-12.4570966	1.28E-35	1.51E-34	Down	CROT
MOB3A	1287.50436	-0.85525471	0.06870284	-12.4486072	1.42E-35	1.68E-34	Down	MOB3A
CTNND1	13237.0899	-0.73929079	0.05941299	-12.443251	1.52E-35	1.79E-34	Down	CTNND1
PLEKHO1	917.687501	-1.62988378	0.13098677	-12.4431174	1.52E-35	1.79E-34	Down	PLEKHO1
RBL1	688.507131	1.31112231	0.105406	12.4387821	1.61E-35	1.89E-34	Up	RBL1
CLCNKB	5.84258122	-3.20037262	0.25729148	-12.4387042	1.61E-35	1.89E-34	Down	CLCNKB
TMEM255A	37.2361491	-2.41580142	0.19422624	-12.4380793	1.62E-35	1.90E-34	Down	TMEM255A
CTDSP2	7899.17409	-0.74655632	0.06003006	-12.4363743	1.66E-35	1.94E-34	Down	CTDSP2
VAPA	3805.74668	-0.93226954	0.07499863	-12.4304884	1.79E-35	2.09E-34	Down	VAPA
CXCL2	908.638004	2.36909035	0.19061006	12.4289892	1.82E-35	2.13E-34	Up	CXCL2
SLC11A1	323.416037	2.41566554	0.19439129	12.4268201	1.87E-35	2.19E-34	Up	SLC11A1
MTFR1L	1112.51765	-0.72650422	0.05853204	-12.412077	2.25E-35	2.63E-34	Down	MTFR1L
SNRPF	2292.56088	1.15448782	0.0930133	12.4120724	2.25E-35	2.63E-34	Up	SNRPF
PLA2G2C	2.91334821	-3.36048442	0.27102228	-12.3992921	2.64E-35	3.08E-34	Down	PLA2G2C
SCML1	1067.64421	1.59399568	0.12856475	12.398388	2.67E-35	3.11E-34	Up	SCML1
P2RY12	13.2215462	-2.86743773	0.23129232	-12.3974617	2.70E-35	3.15E-34	Down	P2RY12
PNCK	102.24949	-3.36128026	0.27118859	-12.3946227	2.79E-35	3.26E-34	Down	PNCK
CTNNA3	17.7807279	-3.79510468	0.3062187	-12.3934452	2.84E-35	3.30E-34	Down	CTNNA3
GAB1	926.217068	-1.1696883	0.09440667	-12.3898899	2.96E-35	3.45E-34	Down	GAB1
DUS4L	305.698916	1.04927663	0.08469721	12.388562	3.01E-35	3.51E-34	Up	DUS4L
GPM6A	80.4916432	-4.50235598	0.36345449	-12.3876746	3.05E-35	3.54E-34	Down	GPM6A
CSF2	25.0544449	3.62037099	0.29234929	12.3837172	3.20E-35	3.72E-34	Up	CSF2
PHOX2B	9.34601847	-4.65285968	0.37587241	-12.3788275	3.40E-35	3.95E-34	Down	PHOX2B
ZADH2	1386.90067	-0.87309026	0.07054091	-12.3770767	3.48E-35	4.04E-34	Down	ZADH2
SUV39H1	716.825927	1.23750905	0.10000583	12.3743686	3.60E-35	4.17E-34	Up	SUV39H1
TCFL5	718.6316	1.35225675	0.10931664	12.3700906	3.79E-35	4.40E-34	Up	TCFL5
EAPP	1282.14486	-0.92412096	0.07473084	-12.3659923	3.99E-35	4.62E-34	Down	EAPP
RRP1B	2043.16914	0.811745	0.06564628	12.3654375	4.02E-35	4.65E-34	Up	RRP1B
KCTD9	876.915843	-1.35288532	0.10942031	-12.3641153	4.09E-35	4.73E-34	Down	KCTD9
TCOF1	3230.25596	1.12383145	0.09091167	12.3617951	4.21E-35	4.86E-34	Up	TCOF1
LAS1L	2139.14996	1.0639751	0.08608113	12.3601439	4.29E-35	4.96E-34	Up	LAS1L
CHL1	139.342352	-2.62562265	0.21250887	-12.3553555	4.56E-35	5.26E-34	Down	CHL1
TRMT11	539.909439	0.91244042	0.07386032	12.353595	4.66E-35	5.37E-34	Up	TRMT11
SLC41A2	704.834866	-1.55534799	0.12590332	-12.3535103	4.66E-35	5.38E-34	Down	SLC41A2
PPM1A	2095.24442	-0.91852545	0.07436346	-12.3518384	4.76E-35	5.49E-34	Down	PPM1A
GJB5	144.431343	4.31608255	0.34953411	12.3481012	4.99E-35	5.74E-34	Up	GJB5
NCAPG	782.485772	1.18587899	0.09607043	12.3438504	5.26E-35	6.05E-34	Up	NCAPG
FAM214A	596.736786	-1.24110593	0.10056163	-12.3417441	5.40E-35	6.21E-34	Down	FAM214A
PCSK2	24.6189889	-4.87296271	0.39488162	-12.3403128	5.49E-35	6.31E-34	Down	PCSK2
SLC26A9	35.3110179	5.50601026	0.4462124	12.3394381	5.55E-35	6.38E-34	Up	SLC26A9
STC1	506.846522	2.03161204	0.16466367	12.3379494	5.66E-35	6.49E-34	Up	STC1
SQLE	3304.45841	1.53522818	0.124457	12.3354101	5.84E-35	6.70E-34	Up	SQLE
SDHD	2701.02142	-1.13791488	0.09225787	-12.3340684	5.94E-35	6.81E-34	Down	SDHD
ILF3	10713.5194	0.67106779	0.05441568	12.3322503	6.07E-35	6.95E-34	Up	ILF3
EMP1	4608.01229	-1.80445889	0.14632081	-12.3322092	6.08E-35	6.95E-34	Down	EMP1
SGCE	251.850701	-1.86697679	0.15139597	-12.3317472	6.11E-35	6.99E-34	Down	SGCE
P2RY4	6.13559767	-3.4033895	0.2759887	-12.3316261	6.12E-35	7.00E-34	Down	P2RY4
PLCG2	404.131161	-1.83070008	0.14846927	-12.3304985	6.21E-35	7.09E-34	Down	PLCG2
CHCHD6	558.943462	1.20971602	0.09813002	12.327685	6.43E-35	7.34E-34	Up	CHCHD6
AP1S3	311.312955	1.3898466	0.11274314	12.3275491	6.44E-35	7.35E-34	Up	AP1S3
EFNA3	754.371852	1.78075695	0.14445559	12.327366	6.45E-35	7.36E-34	Up	EFNA3

NGB	6.32991346	-5.13613917	0.41674275	-12.3244835	6.69E-35	7.62E-34	Down	NGB
IL20RA	1010.12981	1.3641388	0.11070252	12.3225629	6.85E-35	7.80E-34	Up	IL20RA
XDH	1075.36591	-2.34001318	0.18993681	-12.319956	7.07E-35	8.05E-34	Down	XDH
CENPF	2353.0175	1.41940161	0.11527	12.3137125	7.64E-35	8.69E-34	Up	CENPF
GJC3	22.4389987	2.63891667	0.21433393	12.3121743	7.79E-35	8.85E-34	Up	GJC3
PLCD3	3920.85596	-1.55449987	0.12631529	-12.3065057	8.36E-35	9.49E-34	Down	PLCD3
TIGD4	32.6435075	1.63678571	0.13304583	12.3024204	8.79E-35	9.98E-34	Up	TIGD4
RP1	8.3951414	5.46107153	0.44397757	12.3003321	9.02E-35	1.02E-33	Up	RP1
CYP2W1	2212.12118	4.06156682	0.33031904	12.2958908	9.53E-35	1.08E-33	Up	CYP2W1
PITX1	1765.30353	1.78199461	0.14494101	12.2946195	9.68E-35	1.10E-33	Up	PITX1
GPX3	1342.56829	-2.27452815	0.18503667	-12.2923102	9.96E-35	1.13E-33	Down	GPX3
CCT4	7682.74692	0.87376066	0.07108261	12.2921852	9.98E-35	1.13E-33	Up	CCT4
PVR	2488.62714	0.95126577	0.07739534	12.2909956	1.01E-34	1.15E-33	Up	PVR
MORC4	1225.26647	1.34946924	0.10982888	12.2870167	1.06E-34	1.20E-33	Up	MORC4
BCL2L11	889.659917	-1.00040597	0.08142858	-12.285686	1.08E-34	1.22E-33	Down	BCL2L11
ADORA3	92.0593453	-1.99056473	0.16203227	-12.2849897	1.09E-34	1.23E-33	Down	ADORA3
OSBPL1A	726.959647	-1.64421046	0.13384598	-12.2843469	1.10E-34	1.24E-33	Down	OSBPL1A
PNPLA3	49.7219136	3.69479294	0.3008299	12.2820005	1.13E-34	1.28E-33	Up	PNPLA3
HERPUD1	3790.5427	-0.85313427	0.06946919	-12.2807581	1.15E-34	1.29E-33	Down	HERPUD1
ETHE1	5416.29921	-1.55853599	0.12691409	-12.2802439	1.16E-34	1.30E-33	Down	ETHE1
RGP1	1641.65959	-0.88874292	0.0723791	-12.2789987	1.17E-34	1.32E-33	Down	RGP1
GNG12	4904.38375	-1.10692215	0.09015583	-12.2778766	1.19E-34	1.34E-33	Down	GNG12
CDCA8	1565.28146	1.07032956	0.08724311	12.2683568	1.34E-34	1.51E-33	Up	CDCA8
KCNB1	32.5357898	-3.75993798	0.30648776	-12.2678243	1.35E-34	1.51E-33	Down	KCNB1
TMEM108	21.2458566	-1.90234227	0.15508568	-12.2663952	1.37E-34	1.54E-33	Down	TMEM108
NDN	355.835448	-1.78967955	0.145981	-12.2596746	1.49E-34	1.67E-33	Down	NDN
EDN2	49.7407381	-2.73539396	0.22313415	-12.2589661	1.50E-34	1.69E-33	Down	EDN2
SORBS2	1010.49991	-2.04560543	0.16686925	-12.2587319	1.51E-34	1.69E-33	Down	SORBS2
BTNL3	608.366815	-2.65509516	0.21661492	-12.257213	1.54E-34	1.72E-33	Down	BTNL3
ADTRP	744.659704	-3.07255866	0.2507161	-12.255131	1.58E-34	1.76E-33	Down	ADTRP
NIM1K	9.76899592	-1.45950789	0.1191726	-12.2470089	1.74E-34	1.95E-33	Down	NIM1K
PAFAH1B1	3427.62395	-0.74162417	0.06055955	-12.2461958	1.76E-34	1.97E-33	Down	PAFAH1B1
ARRB1	1308.52356	-1.0807787	0.08826479	-12.2447321	1.79E-34	2.00E-33	Down	ARRB1
FAM72A	44.8920521	1.51338406	0.12361606	12.2426164	1.84E-34	2.05E-33	Up	FAM72A
SLC12A2	17946.3697	1.47217796	0.12027096	12.2405109	1.89E-34	2.11E-33	Up	SLC12A2
CD1D	98.7113421	-1.67809689	0.13710784	-12.2392485	1.92E-34	2.14E-33	Down	CD1D
GPR19	38.2866126	1.55057404	0.12671727	12.2364851	1.98E-34	2.21E-33	Up	GPR19
NPM3	1447.77722	1.51388361	0.12375072	12.2333321	2.06E-34	2.30E-33	Up	NPM3
GPR157	926.323026	1.31375926	0.1073939	12.2330907	2.07E-34	2.30E-33	Up	GPR157
VDR	3671.08265	-1.09302797	0.08938349	-12.2285221	2.19E-34	2.43E-33	Down	VDR
CYS1	94.7493212	-2.19205941	0.1792749	-12.2273639	2.22E-34	2.47E-33	Down	CYS1
AGT	987.069964	2.14943282	0.17579544	12.2268973	2.23E-34	2.48E-33	Up	AGT
NBEAL2	5293.95947	1.35546163	0.11088092	12.2244803	2.30E-34	2.55E-33	Up	NBEAL2
ITPKB	955.222904	-1.02905271	0.08418663	-12.2234689	2.33E-34	2.58E-33	Down	ITPKB
DNASE1L3	174.234658	-3.2241952	0.26382387	-12.2210139	2.40E-34	2.66E-33	Down	DNASE1L3
JAZF1	238.16289	-1.76913037	0.14477247	-12.2200747	2.43E-34	2.69E-33	Down	JAZF1
MAP6	60.6435058	-2.23474158	0.18291846	-12.2171463	2.52E-34	2.79E-33	Down	MAP6
DNAH5	55.8179118	2.42341469	0.1984778	12.2100036	2.75E-34	3.04E-33	Up	DNAH5
CBX3	7844.02996	1.0817582	0.08864796	12.2028545	3.00E-34	3.32E-33	Up	CBX3
RCC1	2915.65333	1.04500815	0.08563959	12.2023958	3.02E-34	3.34E-33	Up	RCC1
NPAS4	4.25672279	-2.8172267	0.23088031	-12.202109	3.03E-34	3.34E-33	Down	NPAS4
BRIX1	1386.37945	1.1529395	0.09450636	12.1995966	3.12E-34	3.45E-33	Up	BRIX1
KNTC1	1073.4544	1.21204692	0.09940075	12.1935393	3.36E-34	3.71E-33	Up	KNTC1
HES6	2356.56148	1.86307869	0.15287976	12.1865617	3.67E-34	4.04E-33	Up	HES6
B3GNT7	2174.71848	-3.1232543	0.25628591	-12.1866015	3.66E-34	4.04E-33	Down	B3GNT7
TPD52L2	5913.47114	0.93629478	0.07685985	12.1818446	3.88E-34	4.28E-33	Up	TPD52L2
NXT1	1038.76543	1.43161735	0.11754273	12.1795483	3.99E-34	4.40E-33	Up	NXT1
RASL12	257.899008	-1.68893869	0.13867742	-12.1789016	4.03E-34	4.43E-33	Down	RASL12

EPHA7	132.143478	-3.54535204	0.29112665	-12.1780403	4.07E-34	4.47E-33	Down	EPHA7
IPO9	2780.26204	0.62384644	0.0512371	12.1756785	4.19E-34	4.60E-33	Up	IPO9
RNPS1	4934.45905	0.67137126	0.05515585	12.1722578	4.37E-34	4.79E-33	Up	RNPS1
VCL	6189.99154	-1.1129396	0.09144201	-12.1709875	4.44E-34	4.87E-33	Down	VCL
MT2A	2648.84216	-2.16404516	0.17784406	-12.1682172	4.59E-34	5.03E-33	Down	MT2A
NPSR1	193.038147	5.71397425	0.46958259	12.1681986	4.59E-34	5.03E-33	Up	NPSR1
POLQ	427.403729	1.42814396	0.11736784	12.168103	4.60E-34	5.03E-33	Up	POLQ
XKR4	15.7036935	-4.03013359	0.33120963	-12.1679238	4.61E-34	5.04E-33	Down	XKR4
ZFP69B	81.2762738	1.29248642	0.10626672	12.1626643	4.91E-34	5.37E-33	Up	ZFP69B
TXNIP	16202.1379	-1.55459246	0.12785389	-12.1591335	5.13E-34	5.61E-33	Down	TXNIP
BRPF3	2071.11237	-0.87337211	0.07186847	-12.1523675	5.57E-34	6.09E-33	Down	BRPF3
NUP37	1139.93293	0.91010527	0.07489463	12.1518097	5.61E-34	6.12E-33	Up	NUP37
RBMX	6737.39739	0.61097192	0.05028541	12.1500832	5.73E-34	6.25E-33	Up	RBMX
AQP5	87.7163429	4.98512828	0.4103949	12.1471498	5.94E-34	6.48E-33	Up	AQP5
TTK	725.714733	1.24483203	0.10249618	12.1451559	6.09E-34	6.63E-33	Up	TTK
SPDL1	484.88839	1.01743012	0.08379651	12.141677	6.35E-34	6.92E-33	Up	SPDL1
DHCR7	3831.99776	1.35641677	0.11178196	12.1344872	6.93E-34	7.55E-33	Up	DHCR7
TAGLN3	14.4075921	-3.71822617	0.30642439	-12.1342371	6.96E-34	7.56E-33	Down	TAGLN3
CHGA	1178.62274	-4.41841632	0.36413429	-12.1340299	6.97E-34	7.58E-33	Down	CHGA
MUL1	1185.42504	-0.74633966	0.06152565	-12.1305447	7.28E-34	7.90E-33	Down	MUL1
SLC22A4	104.87801	-1.59387567	0.13142972	-12.1272091	7.58E-34	8.23E-33	Down	SLC22A4
HERC3	796.88851	-1.04950351	0.08654404	-12.1268146	7.62E-34	8.26E-33	Down	HERC3
NDE1	1309.62696	0.78303407	0.06457249	12.1264354	7.65E-34	8.30E-33	Up	NDE1
C9orf43	23.7463784	1.50272798	0.12396357	12.1223359	8.04E-34	8.72E-33	Up	C9orf43
NUDT5	2396.99585	0.7724454	0.06372338	12.1218529	8.09E-34	8.76E-33	Up	NUDT5
IL16	311.87788	-1.81919854	0.15009438	-12.120364	8.24E-34	8.92E-33	Down	IL16
SIGLEC11	10.2272577	-2.22342096	0.18351629	-12.1156596	8.73E-34	9.44E-33	Down	SIGLEC11
CDC25A	741.251766	1.19853349	0.09894442	12.1131994	8.99E-34	9.72E-33	Up	CDC25A
KRTAP5-1	36.6826711	2.19951474	0.181592	12.1123992	9.08E-34	9.81E-33	Up	KRTAP5-1
MAPK4	27.606924	-3.76562563	0.31095222	-12.1099816	9.35E-34	1.01E-32	Down	MAPK4
TSEN2	631.499919	1.1897468	0.09828973	12.1044871	1.00E-33	1.08E-32	Up	TSEN2
LRRC66	271.945155	-1.81699598	0.15014233	-12.1018233	1.03E-33	1.11E-32	Down	LRRC66
SLC16A9	472.613787	-2.35256517	0.19441018	-12.1010391	1.04E-33	1.12E-32	Down	SLC16A9
ACVRL1	2474.93277	-1.52352741	0.12593321	-12.0978998	1.08E-33	1.17E-32	Down	ACVRL1
RIMKLA	445.185819	-1.51149665	0.12497161	-12.09472	1.13E-33	1.21E-32	Down	RIMKLA
GALNT16	53.0026296	-2.66255592	0.22026099	-12.0881864	1.22E-33	1.31E-32	Down	GALNT16
CRY2	933.488648	-0.83375244	0.06898253	-12.0864296	1.25E-33	1.34E-32	Down	CRY2
TRAF3IP2	1326.69002	-0.87939301	0.07276827	-12.0848412	1.27E-33	1.36E-32	Down	TRAF3IP2
FEZ1	175.408634	-1.6438292	0.13604005	-12.0834212	1.29E-33	1.39E-32	Down	FEZ1
BAALC	23.8059973	-2.34856727	0.19436906	-12.0830306	1.30E-33	1.39E-32	Down	BAALC
ATXN7L2	219.488897	1.05271617	0.08712768	12.0824539	1.31E-33	1.40E-32	Up	ATXN7L2
LRRN4CL	100.109161	-1.85866476	0.1538397	-12.0818276	1.32E-33	1.41E-32	Down	LRRN4CL
FRRS1L	12.3658956	-3.4932216	0.28914931	-12.0810304	1.33E-33	1.43E-32	Down	FRRS1L
TBC1D16	2917.321	1.16541015	0.09646772	12.0808296	1.33E-33	1.43E-32	Up	TBC1D16
CDT1	1887.63579	1.60116514	0.13255434	12.0793111	1.36E-33	1.45E-32	Up	CDT1
ATP6V0D2	49.2576718	-2.22653392	0.18434978	-12.0777685	1.38E-33	1.48E-32	Down	ATP6V0D2
FYCO1	1568.22126	-1.03768029	0.08592336	-12.0768122	1.40E-33	1.50E-32	Down	FYCO1
C11orf86	187.984395	-3.68245629	0.30500087	-12.0735928	1.46E-33	1.56E-32	Down	C11orf86
SESN2	863.855076	-1.11007261	0.09194406	-12.0733475	1.46E-33	1.56E-32	Down	SESN2
RPF2	1249.64108	0.95533617	0.07913996	12.0714763	1.49E-33	1.59E-32	Up	RPF2
LOXL2	2156.6764	1.65952975	0.13750734	12.0686627	1.55E-33	1.65E-32	Up	LOXL2
SIDT2	1513.03721	-0.97281247	0.08060861	-12.0683441	1.55E-33	1.65E-32	Down	SIDT2
ECE1	5340.08884	-1.06305314	0.08809337	-12.0673454	1.57E-33	1.67E-32	Down	ECE1
FERMT1	9737.12719	1.46046036	0.12105633	12.0643042	1.63E-33	1.73E-32	Up	FERMT1
TNNT1	129.164053	4.07302491	0.33772202	12.0602884	1.71E-33	1.82E-32	Up	TNNT1
BUB1B	922.661706	1.17268978	0.09725253	12.0581932	1.76E-33	1.87E-32	Up	BUB1B
DHRS9	904.360716	-3.3077694	0.27432938	-12.0576564	1.77E-33	1.88E-32	Down	DHRS9
SYNE3	230.724456	-1.64452588	0.13639529	-12.0570581	1.78E-33	1.89E-32	Down	SYNE3

PRKCG	81.7143809	3.68382388	0.30569224	12.0507601	1.92E-33	2.04E-32	Up	PRKCG
SET	11990.4244	0.75460205	0.06264078	12.0464993	2.02E-33	2.15E-32	Up	SET
TYRO3	498.107578	1.74450735	0.14492395	12.0373988	2.26E-33	2.39E-32	Up	TYRO3
ENHO	23.7954747	-2.43545946	0.20234525	-12.0361581	2.29E-33	2.43E-32	Down	ENHO
PLEKHA7	1351.10629	-0.95118287	0.07903758	-12.034564	2.34E-33	2.48E-32	Down	PLEKHA7
SLC7A1	5457.22036	1.15755989	0.09618939	12.0341748	2.35E-33	2.49E-32	Up	SLC7A1
CD209	260.232019	-2.27734719	0.18924443	-12.0338927	2.36E-33	2.49E-32	Down	CD209
MAD2L2	855.334006	1.29227125	0.10741585	12.030545	2.46E-33	2.59E-32	Up	MAD2L2
PLOD3	7343.16285	1.29391665	0.10755935	12.0297925	2.48E-33	2.62E-32	Up	PLOD3
DYRK2	2471.6309	-0.96148935	0.07996189	-12.0243454	2.65E-33	2.79E-32	Down	DYRK2
CASQ2	147.105386	-4.29881118	0.35764216	-12.019867	2.79E-33	2.95E-32	Down	CASQ2
AKR1B10	854.377992	-3.07785918	0.25607329	-12.0194463	2.81E-33	2.96E-32	Down	AKR1B10
AOC1	9081.70216	-1.7710836	0.14735221	-12.0193898	2.81E-33	2.96E-32	Down	AOC1
NCOA1	1381.60781	-0.90033332	0.07492726	-12.0160989	2.92E-33	3.08E-32	Down	NCOA1
GRB7	2009.374	1.70289629	0.14173479	12.0146665	2.98E-33	3.13E-32	Up	GRB7
GPSM2	2282.44631	1.28030332	0.1066093	12.0093018	3.18E-33	3.34E-32	Up	GPSM2
ATAT1	540.651766	1.10053214	0.09166145	12.0064884	3.29E-33	3.45E-32	Up	ATAT1
C9orf116	74.5615989	1.77361223	0.14778267	12.00149	3.49E-33	3.67E-32	Up	C9orf116
GEMIN5	1049.42055	0.7491507	0.0624255	12.0007169	3.52E-33	3.70E-32	Up	GEMIN5
KLHDC8B	326.553915	-1.48690337	0.12390396	-12.0004504	3.53E-33	3.71E-32	Down	KLHDC8B
MOCS1	353.159108	-1.61278198	0.13439681	-12.0001507	3.55E-33	3.72E-32	Down	MOCS1
PADI3	92.2092572	4.57701864	0.3814585	11.9987329	3.61E-33	3.78E-32	Up	PADI3
LMNB2	6218.01185	1.10649562	0.09222418	11.9978911	3.64E-33	3.82E-32	Up	LMNB2
AFMID	1013.20319	0.91306905	0.07611289	11.996247	3.72E-33	3.89E-32	Up	AFMID
IFRD2	3118.76327	1.21373555	0.10118841	11.9948075	3.78E-33	3.96E-32	Up	IFRD2
NEIL3	157.449247	1.27743467	0.10654506	11.9896193	4.03E-33	4.21E-32	Up	NEIL3
IRAK2	483.724307	1.621939	0.13532525	11.9854871	4.23E-33	4.42E-32	Up	IRAK2
COLGALT1	5358.21159	0.87056848	0.07263801	11.9850266	4.26E-33	4.45E-32	Up	COLGALT1
ADAMTS6	87.0386284	2.17156426	0.18121231	11.9835363	4.33E-33	4.52E-32	Up	ADAMTS6
EIF4EBP1	2237.85715	1.53055589	0.12773223	11.9825346	4.39E-33	4.58E-32	Up	EIF4EBP1
SAP30L	1289.03307	-0.68779834	0.0574037	-11.9817774	4.43E-33	4.62E-32	Down	SAP30L
DBF4	742.557048	1.09032995	0.09103179	11.977464	4.66E-33	4.86E-32	Up	DBF4
CCDC152	59.6626649	-2.35245076	0.19653572	-11.9695837	5.13E-33	5.34E-32	Down	CCDC152
DUSP26	13.4952767	-2.6963283	0.22527606	-11.968996	5.16E-33	5.37E-32	Down	DUSP26
IGF2BP1	159.070338	4.84270001	0.4046287	11.9682565	5.21E-33	5.42E-32	Up	IGF2BP1
NPC1L1	222.093835	3.99301605	0.33364811	11.9677466	5.24E-33	5.45E-32	Up	NPC1L1
ZNF132	61.8422434	-1.14706763	0.09585318	-11.9669237	5.30E-33	5.50E-32	Down	ZNF132
ZNF697	339.484084	1.38135277	0.11543938	11.9660443	5.35E-33	5.56E-32	Up	ZNF697
PRDX4	4795.11818	1.2497833	0.10447076	11.9629957	5.55E-33	5.76E-32	Up	PRDX4
SP6	905.76412	2.03365357	0.17006018	11.9584346	5.87E-33	6.08E-32	Up	SP6
SAPCD1	29.0229292	2.13803319	0.17880861	11.9571043	5.96E-33	6.18E-32	Up	SAPCD1
TAX1BP3	1020.91582	-1.0511231	0.08791566	-11.9560395	6.04E-33	6.25E-32	Down	TAX1BP3
MOGAT2	952.972536	-2.7580688	0.23070385	-11.9550185	6.11E-33	6.33E-32	Down	MOGAT2
PA2G4	6769.68294	0.81168302	0.06791133	11.9521005	6.33E-33	6.55E-32	Up	PA2G4
THRB	324.917665	-2.40626871	0.20138035	-11.9488756	6.58E-33	6.80E-32	Down	THRB
ECE2	760.792602	1.45729998	0.12197835	11.9472021	6.71E-33	6.94E-32	Up	ECE2
MMS22L	600.486674	1.32926691	0.11131531	11.9414567	7.20E-33	7.43E-32	Up	MMS22L
ZNF280A	6.34203753	5.2399621	0.43885179	11.9401635	7.31E-33	7.54E-32	Up	ZNF280A
EIF4EBP2	6716.62161	-0.77243666	0.06471165	-11.936594	7.63E-33	7.87E-32	Down	EIF4EBP2
PGAM5	3355.88029	1.04725696	0.08773933	11.9360043	7.68E-33	7.92E-32	Up	PGAM5
GOLM1	9979.95299	-1.25690268	0.10533113	-11.9328699	7.98E-33	8.22E-32	Down	GOLM1
RBM19	1139.39472	0.71666563	0.06006398	11.9317032	8.09E-33	8.33E-32	Up	RBM19
LRRC75A	839.767126	-1.19792433	0.10041293	-11.9299803	8.26E-33	8.50E-32	Down	LRRC75A
NSUN5	1419.0268	1.04769623	0.08782635	11.9291782	8.34E-33	8.58E-32	Up	NSUN5
NMNAT1	549.85563	-0.8938098	0.07493665	-11.9275392	8.51E-33	8.74E-32	Down	NMNAT1
DIO2	391.954007	2.06951286	0.17351752	11.9268234	8.58E-33	8.81E-32	Up	DIO2
PROSER1	4117.64151	1.28175294	0.10747611	11.9259338	8.67E-33	8.90E-32	Up	PROSER1
LSM8	1163.31583	0.89742598	0.07525842	11.9245919	8.81E-33	9.04E-32	Up	LSM8

ATL1	195.964546	-1.27973518	0.10733437	-11.9228833	8.99E-33	9.22E-32	Down	ATL1
ITGA2	3071.32156	1.31171017	0.11001947	11.9225276	9.03E-33	9.26E-32	Up	ITGA2
PKP4	3176.5605	0.69051992	0.05792527	11.9208759	9.21E-33	9.44E-32	Up	PKP4
FABP2	401.934539	-3.15178009	0.26445207	-11.9181523	9.52E-33	9.75E-32	Down	FABP2
OGN	225.129638	-3.95446842	0.33192272	-11.9138225	1.00E-32	1.03E-31	Down	OGN
DMD	546.377372	-2.24343119	0.18832623	-11.9124736	1.02E-32	1.04E-31	Down	DMD
SEC14L4	24.2991381	4.89752513	0.41125905	11.9086135	1.07E-32	1.09E-31	Up	SEC14L4
MSI2	3267.68123	1.05861569	0.08892146	11.9050642	1.11E-32	1.14E-31	Up	MSI2
KNSTRN	693.638277	1.04775863	0.08801182	11.9047484	1.12E-32	1.14E-31	Up	KNSTRN
POLM	874.778434	0.94180173	0.07913171	11.9016982	1.16E-32	1.18E-31	Up	POLM
IGSF23	26.7606382	2.92433841	0.24576	11.8991633	1.20E-32	1.22E-31	Up	IGSF23
PMEPA1	6940.98343	1.88291186	0.1582586	11.8976903	1.22E-32	1.24E-31	Up	PMEPA1
POLR1D	5346.3185	1.12499831	0.0945652	11.8965365	1.23E-32	1.26E-31	Up	POLR1D
ARHGEF19	662.793811	1.40839233	0.11840848	11.8943533	1.27E-32	1.29E-31	Up	ARHGEF19
REG3A	3532.5991	5.30305012	0.44590154	11.8928725	1.29E-32	1.31E-31	Up	REG3A
TMEM59L	23.6582332	-2.88712436	0.2427777	-11.8920491	1.30E-32	1.32E-31	Down	TMEM59L
TFDP1	5520.25491	0.90080334	0.07580709	11.8828384	1.45E-32	1.48E-31	Up	TFDP1
ACO2	4391.6829	-1.05980249	0.08919908	-11.8813157	1.48E-32	1.50E-31	Down	ACO2
TGFB1I1	1114.64117	-1.54514557	0.13009952	-11.8766429	1.57E-32	1.59E-31	Down	TGFB1I1
PPIL1	1423.9892	1.08183299	0.09110604	11.874438	1.61E-32	1.63E-31	Up	PPIL1
ONECUT2	181.368296	2.70044363	0.22742963	11.8737547	1.62E-32	1.64E-31	Up	ONECUT2
TRIM65	1213.79266	0.86329401	0.07276054	11.864865	1.80E-32	1.83E-31	Up	TRIM65
ST7	950.114829	0.68996248	0.05815661	11.8638696	1.82E-32	1.85E-31	Up	ST7
RND2	24.8313428	-2.29578607	0.1935135	-11.8636996	1.83E-32	1.85E-31	Down	RND2
SH2D5	16.4716874	3.2416906	0.27336804	11.858338	1.95E-32	1.97E-31	Up	SH2D5
CGRRF1	262.326339	-1.12422583	0.09482435	-11.8558768	2.01E-32	2.03E-31	Down	CGRRF1
AP3M2	678.625617	0.99379236	0.08382879	11.8550244	2.03E-32	2.05E-31	Up	AP3M2
TXLNG	1368.4137	1.08712459	0.09170804	11.8541903	2.05E-32	2.07E-31	Up	TXLNG
SUN2	3831.67574	-0.99970885	0.08433951	-11.8533864	2.07E-32	2.09E-31	Down	SUN2
PODXL2	2033.60524	1.58139663	0.13341511	11.853205	2.07E-32	2.09E-31	Up	PODXL2
CHEK2	665.74619	0.91847125	0.07750322	11.8507495	2.13E-32	2.15E-31	Up	CHEK2
LIMK1	1728.93562	1.03424045	0.08727373	11.8505357	2.14E-32	2.15E-31	Up	LIMK1
HYAL3	189.030952	1.36947884	0.11560044	11.8466575	2.24E-32	2.26E-31	Up	HYAL3
SFXN3	1732.56115	1.00727823	0.08505489	11.8426844	2.35E-32	2.36E-31	Up	SFXN3
EDAR	625.587994	2.88495966	0.24363377	11.8413786	2.38E-32	2.40E-31	Up	EDAR
NFE2L1	8202.10832	-0.81647496	0.06897368	-11.8374859	2.50E-32	2.51E-31	Down	NFE2L1
COL9A3	969.74688	3.1135537	0.26303368	11.8370915	2.51E-32	2.52E-31	Up	COL9A3
ARFGAP1	4549.01991	1.28904211	0.10892033	11.8347241	2.58E-32	2.59E-31	Up	ARFGAP1
ZFYVE1	561.055113	-0.87332131	0.07379515	-11.8343998	2.59E-32	2.60E-31	Down	ZFYVE1
FGFR2	664.755557	-2.27103298	0.191938	-11.8321174	2.66E-32	2.67E-31	Down	FGFR2
MYO1D	14369.6551	-1.08974644	0.09212333	-11.8292131	2.76E-32	2.76E-31	Down	MYO1D
SEC14L5	22.8297353	-2.4335193	0.20579524	-11.8249545	2.90E-32	2.91E-31	Down	SEC14L5
OLFML2A	634.639327	-1.48093679	0.12523955	-11.8248328	2.90E-32	2.91E-31	Down	OLFML2A
CCT5	8911.47901	0.78920058	0.0667421	11.8246291	2.91E-32	2.91E-31	Up	CCT5
SMCO2	8.00454133	2.54005261	0.21488231	11.8206687	3.05E-32	3.05E-31	Up	SMCO2
STAB2	32.9958685	-2.11623031	0.17904476	-11.8195602	3.09E-32	3.09E-31	Down	STAB2
BCL2	346.951726	-1.68235985	0.14241745	-11.8128775	3.35E-32	3.35E-31	Down	BCL2
SPRR1A	19.149551	6.88170008	0.58256338	11.812792	3.35E-32	3.35E-31	Up	SPRR1A
PARM1	6655.27045	-1.55844661	0.13193116	-11.8125742	3.36E-32	3.35E-31	Down	PARM1
SSC4D	38.5637228	1.33956806	0.11340871	11.8118625	3.39E-32	3.38E-31	Up	SSC4D
PER1	919.109048	-1.59000615	0.13461064	-11.8118908	3.39E-32	3.38E-31	Down	PER1
STIP1	6434.35665	0.79305889	0.06714197	11.8116722	3.40E-32	3.39E-31	Up	STIP1
SYT7	2397.49927	1.83017688	0.15495055	11.8113611	3.41E-32	3.40E-31	Up	SYT7
CDH19	41.0665515	-4.26493668	0.36111956	-11.8103176	3.45E-32	3.44E-31	Down	CDH19
MAPK10	70.5256753	-1.98909431	0.16842535	-11.8099463	3.47E-32	3.45E-31	Down	MAPK10
KLHL31	60.0797475	1.82568821	0.15458904	11.8099461	3.47E-32	3.45E-31	Up	KLHL31
PGR	47.5594786	-2.67972103	0.22703569	-11.8030829	3.76E-32	3.74E-31	Down	PGR
KIF5A	62.9505725	-2.55284513	0.21636596	-11.7987372	3.96E-32	3.93E-31	Down	KIF5A

TGDS	761.196794	1.16042159	0.0983629	11.7973501	4.03E-32	4.00E-31	Up	TGDS
CTU2	767.298074	1.20437823	0.10209619	11.7965046	4.07E-32	4.04E-31	Up	CTU2
TPH1	73.7120754	-3.24900574	0.27545482	-11.7950585	4.14E-32	4.10E-31	Down	TPH1
GAP43	38.8918843	-3.18904341	0.27037499	-11.7948904	4.15E-32	4.11E-31	Down	GAP43
LRCH2	56.5381948	-2.30313	0.19527369	-11.794369	4.17E-32	4.13E-31	Down	LRCH2
TP73	167.637112	2.19977924	0.18657682	11.7902067	4.38E-32	4.34E-31	Up	TP73
MFN2	4025.46463	-0.79806317	0.06770364	-11.787597	4.52E-32	4.47E-31	Down	MFN2
FCER1A	30.5859637	-2.61117594	0.22151894	-11.787597	4.52E-32	4.47E-31	Down	FCER1A
BUB3	4098.47071	0.7070722	0.05998805	11.7868851	4.56E-32	4.51E-31	Up	BUB3
ACD	902.763846	1.05825732	0.0897879	11.786191	4.60E-32	4.54E-31	Up	ACD
PDLIM3	979.986185	-2.19316358	0.186149	-11.7817643	4.85E-32	4.78E-31	Down	PDLIM3
KY	8.07174878	-2.6077823	0.22134113	-11.7817342	4.85E-32	4.78E-31	Down	KY
SEMA3G	224.491576	-1.95805301	0.16623629	-11.7787341	5.02E-32	4.95E-31	Down	SEMA3G
BRAT1	2798.32311	1.11498632	0.09466421	11.7783301	5.05E-32	4.97E-31	Up	BRAT1
FAM107B	1683.3966	-1.50760103	0.12801738	-11.7765342	5.16E-32	5.08E-31	Down	FAM107B
RPIA	2103.05135	0.87937985	0.0746822	11.7749583	5.25E-32	5.17E-31	Up	RPIA
RIOK1	968.853104	0.72877467	0.06191522	11.7705251	5.54E-32	5.45E-31	Up	RIOK1
CASP2	2170.83695	0.73541904	0.0624816	11.77017	5.56E-32	5.47E-31	Up	CASP2
CXCL17	34.9890475	4.61767547	0.39246613	11.7657934	5.86E-32	5.76E-31	Up	CXCL17
DCAF11	2933.52748	-0.72052046	0.06125586	-11.7624738	6.09E-32	5.98E-31	Down	DCAF11
C14orf132	217.601437	-2.07264485	0.17621046	-11.7623258	6.10E-32	5.99E-31	Down	C14orf132
GLA	1186.02712	1.02653359	0.08727641	11.7618674	6.14E-32	6.02E-31	Up	GLA
GPATCH4	1492.64929	0.87647177	0.07452488	11.7607938	6.21E-32	6.09E-31	Up	GPATCH4
CD276	3094.64378	1.05560794	0.08977923	11.7578187	6.44E-32	6.31E-31	Up	CD276
PAQR8	3186.90839	-1.4309489	0.12170321	-11.757692	6.45E-32	6.31E-31	Down	PAQR8
POLR3E	1850.23903	0.80550101	0.0685133	11.7568564	6.51E-32	6.37E-31	Up	POLR3E
YDJC	1529.69647	1.51446175	0.12884332	11.7542904	6.71E-32	6.57E-31	Up	YDJC
GNPTAB	2102.20052	-0.90068722	0.07663882	-11.7523627	6.87E-32	6.72E-31	Down	GNPTAB
CLDN18	326.409981	5.63783694	0.4799072	11.7477648	7.25E-32	7.09E-31	Up	CLDN18
SLC44A4	10968.4883	-1.44903659	0.12335559	-11.7468256	7.33E-32	7.16E-31	Down	SLC44A4
SLC35F1	25.5789357	-2.13365566	0.18165487	-11.7456565	7.43E-32	7.26E-31	Down	SLC35F1
COL27A1	869.239218	2.02888192	0.17280442	11.7409145	7.86E-32	7.67E-31	Up	COL27A1
BCL2L12	912.765007	1.18953284	0.10134328	11.7376584	8.17E-32	7.97E-31	Up	BCL2L12
C2	1973.84151	1.66672016	0.14206161	11.7323752	8.70E-32	8.48E-31	Up	C2
ARHGEF9	780.747022	-0.90956238	0.07752793	-11.7320607	8.73E-32	8.50E-31	Down	ARHGEF9
AHSG	31.2200491	5.85958808	0.49950804	11.7307182	8.87E-32	8.64E-31	Up	AHSG
FOLR2	260.110585	-2.36018657	0.20120214	-11.7304249	8.90E-32	8.66E-31	Down	FOLR2
ADCY2	53.6211078	-2.21945163	0.1892227	-11.7293094	9.02E-32	8.77E-31	Down	ADCY2
PAX9	75.2687874	2.75145456	0.23460201	11.7281797	9.14E-32	8.88E-31	Up	PAX9
RGMA	331.23391	-2.42689126	0.2069526	-11.7267975	9.29E-32	9.03E-31	Down	RGMA
GRIN3A	39.5371476	-1.8295809	0.15603997	-11.7250786	9.48E-32	9.21E-31	Down	GRIN3A
TMEM241	298.310616	0.87471291	0.07461777	11.722581	9.77E-32	9.48E-31	Up	TMEM241
HDAC2	4536.8709	0.74513098	0.06359095	11.7175641	1.04E-31	1.00E-30	Up	HDAC2
TST	6003.38306	-1.40786126	0.12015096	-11.7174363	1.04E-31	1.01E-30	Down	TST
TRIP4	733.89828	-0.6716992	0.05732633	-11.717114	1.04E-31	1.01E-30	Down	TRIP4
SH3BGRL2	4253.5744	-1.03039053	0.08795429	-11.7150691	1.07E-31	1.03E-30	Down	SH3BGRL2
CCT7	11950.3848	0.87360515	0.07459517	11.7112822	1.12E-31	1.08E-30	Up	CCT7
RSL1D1	7037.03275	0.87079212	0.07435869	11.710697	1.12E-31	1.09E-30	Up	RSL1D1
LIME1	146.852836	1.74796133	0.14927506	11.7096672	1.14E-31	1.10E-30	Up	LIME1
SPRR1B	25.5288788	6.73251148	0.57508568	11.7069711	1.17E-31	1.13E-30	Up	SPRR1B
ANGPT2	284.224911	1.47991154	0.12643698	11.7047367	1.21E-31	1.16E-30	Up	ANGPT2
CASR	3.26448091	-3.38723566	0.28955686	-11.6979984	1.30E-31	1.26E-30	Down	CASR
SIK3	1130.31119	-0.70343907	0.06013516	-11.6976331	1.31E-31	1.26E-30	Down	SIK3
DPH7	894.29406	0.98755827	0.08442462	11.6975149	1.31E-31	1.27E-30	Up	DPH7
LRRFIP2	1737.39328	-0.79102587	0.06762591	-11.6970831	1.32E-31	1.27E-30	Down	LRRFIP2
PTRH2	912.990651	0.910682	0.07786253	11.6960231	1.34E-31	1.29E-30	Up	PTRH2
CDC42SE2	3741.00632	-0.87399669	0.07475906	-11.6908472	1.42E-31	1.37E-30	Down	CDC42SE2
HCFC2	290.151072	-1.21263862	0.10372905	-11.6904433	1.43E-31	1.37E-30	Down	HCFC2

KIF1C	6614.91976	-0.95460551	0.08169884	-11.6844444	1.53E-31	1.47E-30	Down	KIF1C
TAS2R38	11.3566937	2.91030753	0.24909739	11.6834126	1.55E-31	1.49E-30	Up	TAS2R38
DTNB	381.072205	1.22952854	0.1052627	11.6805716	1.60E-31	1.54E-30	Up	DTNB
GDE1	3926.15518	-0.73504433	0.06293726	-11.679001	1.63E-31	1.57E-30	Down	GDE1
MASTL	560.25943	0.92248438	0.07898822	11.6787588	1.64E-31	1.57E-30	Up	MASTL
ATAD3B	1096.18375	1.36119302	0.11656198	11.677847	1.65E-31	1.59E-30	Up	ATAD3B
AVPR1B	1.48859129	-3.78616033	0.32429211	-11.6751542	1.71E-31	1.64E-30	Down	AVPR1B
ACTL10	307.611872	1.79800903	0.15403591	11.6726618	1.76E-31	1.68E-30	Up	ACTL10
KREMEN2	82.3616517	2.26296287	0.19404636	11.6619703	1.99E-31	1.91E-30	Up	KREMEN2
ANO5	82.5188399	-3.15471258	0.27056825	-11.6595818	2.05E-31	1.96E-30	Down	ANO5
BMP4	2726.62071	1.94722536	0.167018	11.6587754	2.07E-31	1.98E-30	Up	BMP4
CASQ1	8.25569303	-2.50279507	0.21470841	-11.6567167	2.12E-31	2.03E-30	Down	CASQ1
GGCT	1975.82793	1.07498814	0.0922441	11.6537336	2.20E-31	2.10E-30	Up	GGCT
PDGFD	208.74057	-1.64259628	0.14097773	-11.6514589	2.26E-31	2.15E-30	Down	PDGFD
B3GALT1	93.629311	-3.29461597	0.28277887	-11.6508564	2.27E-31	2.17E-30	Down	B3GALT1
FGF20	31.1053832	6.07245427	0.52122636	11.6503207	2.29E-31	2.18E-30	Up	FGF20
RANGAP1	5005.25666	1.02488939	0.08798222	11.6488242	2.33E-31	2.22E-30	Up	RANGAP1
SLC38A7	802.48946	0.7654843	0.06572034	11.6476013	2.36E-31	2.25E-30	Up	SLC38A7
RERG	147.087809	-2.33153437	0.20020223	-11.6458959	2.41E-31	2.29E-30	Down	RERG
COL21A1	44.5545335	-2.23220872	0.19170886	-11.6437429	2.47E-31	2.35E-30	Down	COL21A1
USP36	2248.13536	0.81234075	0.06978105	11.6412804	2.54E-31	2.42E-30	Up	USP36
GYPC	338.542697	-1.72378415	0.14809931	-11.63938	2.60E-31	2.47E-30	Down	GYPC
ABHD6	559.059431	-0.92819639	0.07975867	-11.6375607	2.66E-31	2.52E-30	Down	ABHD6
DHX37	1543.30413	0.91372037	0.07851925	11.6368957	2.68E-31	2.54E-30	Up	DHX37
GSTM2	146.691973	-1.51941462	0.13061682	-11.6326106	2.81E-31	2.67E-30	Down	GSTM2
ADNP	4717.72675	0.79847073	0.06864267	11.6322789	2.82E-31	2.68E-30	Up	ADNP
SCNN1G	54.9541239	-4.24386324	0.36485787	-11.6315519	2.85E-31	2.70E-30	Down	SCNN1G
PLXNA2	2147.90192	-1.1121246	0.09562197	-11.6304299	2.89E-31	2.74E-30	Down	PLXNA2
CUL4A	4813.79737	0.85412638	0.07344367	11.6296802	2.91E-31	2.76E-30	Up	CUL4A
MAGEE2	1.37502643	-3.3722822	0.28998752	-11.6290599	2.93E-31	2.78E-30	Down	MAGEE2
KDM1A	4066.81908	0.81280863	0.0698983	11.6284468	2.95E-31	2.80E-30	Up	KDM1A
GIMAP8	239.733083	-1.56404133	0.13450907	-11.6277756	2.98E-31	2.82E-30	Down	GIMAP8
GINS4	341.021458	1.26376742	0.10871287	11.6248188	3.08E-31	2.91E-30	Up	GINS4
PER3	339.283012	-1.77098823	0.15234819	-11.6246096	3.09E-31	2.92E-30	Down	PER3
CD36	386.392292	-2.49954238	0.21509918	-11.6204182	3.25E-31	3.06E-30	Down	CD36
UTP18	1547.43758	0.83160484	0.07157465	11.6187066	3.31E-31	3.12E-30	Up	UTP18
SHROOM3	4462.82897	-0.99980685	0.08605391	-11.6183776	3.32E-31	3.14E-30	Down	SHROOM3
CD79B	123.550358	-2.25369904	0.1939881	-11.6177181	3.35E-31	3.16E-30	Down	CD79B
AARD	7.64541337	-2.54430203	0.21900197	-11.6177129	3.35E-31	3.16E-30	Down	AARD
ESPL1	975.324311	1.11210358	0.09573033	11.6170453	3.38E-31	3.18E-30	Up	ESPL1
ATP2B3	3.61769124	-3.48116719	0.2997196	-11.6147466	3.47E-31	3.26E-30	Down	ATP2B3
MRPS23	1838.65439	0.99512257	0.08568075	11.6143068	3.49E-31	3.28E-30	Up	MRPS23
GPR37L1	89.3303963	-1.60492898	0.13824	-11.6097295	3.68E-31	3.46E-30	Down	GPR37L1
UMPS	1564.43576	0.60841755	0.05240631	11.6096244	3.68E-31	3.46E-30	Up	UMPS
GPA33	9861.71666	-1.79830173	0.15491667	-11.6081873	3.74E-31	3.52E-30	Down	GPA33
SSBP1	2947.10276	0.8533831	0.07352343	11.6069548	3.80E-31	3.57E-30	Up	SSBP1
SLC25A32	1192.04433	1.01645096	0.08757627	11.606466	3.82E-31	3.58E-30	Up	SLC25A32
LRRN4	13.0478326	3.02819882	0.26091227	11.6061954	3.83E-31	3.59E-30	Up	LRRN4
RAB8A	1751.42377	-0.52177392	0.04496226	-11.6047079	3.90E-31	3.66E-30	Down	RAB8A
CES3	1673.83044	-2.16585935	0.18664278	-11.6043031	3.92E-31	3.67E-30	Down	CES3
WDR72	553.203582	3.12663448	0.26948155	11.6024064	4.01E-31	3.75E-30	Up	WDR72
ZNF251	594.966649	1.09417977	0.09433113	11.5993495	4.15E-31	3.89E-30	Up	ZNF251
CHAT	2.55402297	-4.61938443	0.39842656	-11.5940675	4.42E-31	4.13E-30	Down	CHAT
LMBR1	2064.74585	0.83604325	0.07211831	11.5926626	4.49E-31	4.20E-30	Up	LMBR1
EIF2S3	9245.3954	0.95465496	0.08235247	11.5923049	4.51E-31	4.21E-30	Up	EIF2S3
MAGEA6	126.334732	9.83966802	0.8490533	11.5889874	4.69E-31	4.38E-30	Up	MAGEA6
RFX8	8.98967877	2.82753953	0.24399359	11.5885812	4.71E-31	4.39E-30	Up	RFX8
ATG4A	481.299569	-0.9214405	0.07951848	-11.5877531	4.75E-31	4.44E-30	Down	ATG4A

SLC23A3	68.7451293	-1.76278108	0.15212853	-11.5874457	4.77E-31	4.45E-30	Down	SLC23A3
DNTTIP1	2632.37741	1.31011033	0.11306906	11.5868155	4.81E-31	4.48E-30	Up	DNTTIP1
FAM163A	16.5944989	-2.11853557	0.18286097	-11.5854991	4.88E-31	4.55E-30	Down	FAM163A
FEZ2	1100.48922	-0.88813516	0.07668263	-11.5819601	5.09E-31	4.74E-30	Down	FEZ2
PSMA7	11383.5299	1.29125971	0.1115082	11.5799525	5.21E-31	4.85E-30	Up	PSMA7
VIP	305.944352	-3.35065373	0.28935434	-11.5797597	5.22E-31	4.85E-30	Down	VIP
FAM98A	1601.42189	0.52201141	0.04508264	11.5789888	5.27E-31	4.89E-30	Up	FAM98A
SLC25A42	248.981181	-1.11087047	0.0959466	-11.5780071	5.33E-31	4.95E-30	Down	SLC25A42
EFTUD2	4865.14241	0.68171672	0.05889064	11.575977	5.45E-31	5.06E-30	Up	EFTUD2
NAT1	367.616581	-1.46072153	0.12618727	-11.575823	5.46E-31	5.07E-30	Down	NAT1
ATP2B1	2053.68582	-1.06971182	0.09245214	-11.5704391	5.82E-31	5.40E-30	Down	ATP2B1
HCAR1	88.3100529	3.8331869	0.33129229	11.5704077	5.82E-31	5.40E-30	Up	HCAR1
EARS2	1349.20235	0.684451	0.05915624	11.5702243	5.83E-31	5.40E-30	Up	EARS2
KLRB1	96.0306517	-1.91073826	0.1651509	-11.5696511	5.87E-31	5.44E-30	Down	KLRB1
TSPEAR	60.7872433	3.19222681	0.27593185	11.5688958	5.92E-31	5.48E-30	Up	TSPEAR
MMP8	15.2911053	4.56652539	0.39485533	11.5650595	6.19E-31	5.73E-30	Up	MMP8
NEU3	590.71419	0.81421195	0.07041854	11.5624652	6.38E-31	5.90E-30	Up	NEU3
PLA2G5	41.4846292	-2.5018454	0.21639043	-11.5617194	6.44E-31	5.95E-30	Down	PLA2G5
HEATR5A	773.005294	-0.95549331	0.08264533	-11.5613712	6.47E-31	5.97E-30	Down	HEATR5A
ZNF835	12.7678041	-2.09083751	0.18088288	-11.5590682	6.64E-31	6.13E-30	Down	ZNF835
TCF3	4554.64149	1.00421052	0.08688157	11.558384	6.70E-31	6.18E-30	Up	TCF3
PRKDC	9219.95349	1.15983804	0.10034959	11.5579755	6.73E-31	6.20E-30	Up	PRKDC
FAM13C	52.4394643	-1.86099613	0.161074	-11.5536719	7.07E-31	6.52E-30	Down	FAM13C
SERPINH1	7496.75905	1.20468251	0.10427102	11.5533782	7.10E-31	6.54E-30	Up	SERPINH1
HMCN2	645.494162	-2.25875703	0.19552012	-11.5525555	7.17E-31	6.60E-30	Down	HMCN2
PALM3	134.475848	2.94124273	0.25461365	11.5517873	7.23E-31	6.65E-30	Up	PALM3
GFRA3	42.8590042	-2.8721204	0.24867502	-11.549694	7.41E-31	6.81E-30	Down	GFRA3
CPSF4	1200.44015	0.93561087	0.08102371	11.547371	7.61E-31	7.00E-30	Up	CPSF4
FGF18	74.9824985	2.02085158	0.17501941	11.5464428	7.69E-31	7.07E-30	Up	FGF18
PROX1	1357.30897	1.91060585	0.16547698	11.5460522	7.73E-31	7.10E-30	Up	PROX1
POLG2	316.677912	0.91321458	0.07910055	11.5449846	7.83E-31	7.18E-30	Up	POLG2
MYBPHL	21.3360573	5.79336924	0.5018528	11.5439612	7.92E-31	7.27E-30	Up	MYBPHL
ATP6V1D	1508.0046	-0.7749281	0.06717567	-11.5358439	8.70E-31	7.98E-30	Down	ATP6V1D
CMBL	2895.52107	-1.73617633	0.15052316	-11.53428	8.86E-31	8.12E-30	Down	CMBL
IFITM3	17784.0567	1.58086262	0.13707604	11.5327424	9.02E-31	8.27E-30	Up	IFITM3
BICD1	391.887664	1.3195124	0.11442787	11.5313898	9.17E-31	8.39E-30	Up	BICD1
CYP4B1	16.48912	-2.77403619	0.24059606	-11.529849	9.33E-31	8.54E-30	Down	CYP4B1
PFDN2	1709.45984	1.0717515	0.0929675	11.5282387	9.51E-31	8.70E-30	Up	PFDN2
CWH43	202.119404	-3.86272633	0.33510099	-11.5270514	9.64E-31	8.81E-30	Down	CWH43
LXN	570.401547	-1.32206652	0.11470851	-11.5254442	9.82E-31	8.97E-30	Down	LXN
ATP8A2	23.2705561	-1.84870275	0.16040148	-11.5254717	9.82E-31	8.97E-30	Down	ATP8A2
NPR1	162.67036	-1.73558646	0.15063594	-11.5217291	1.03E-30	9.36E-30	Down	NPR1
MYBBP1A	2841.81914	1.04822981	0.09098161	11.5213366	1.03E-30	9.40E-30	Up	MYBBP1A
CXXC5	2923.5736	1.06933607	0.09284024	11.5180238	1.07E-30	9.76E-30	Up	CXXC5
CCDC158	9.79215446	-2.04826495	0.17784946	-11.5168464	1.09E-30	9.89E-30	Down	CCDC158
SPC24	544.285895	1.28246255	0.11135741	11.5166344	1.09E-30	9.91E-30	Up	SPC24
CARS2	2713.93962	1.02911739	0.08936065	11.5164487	1.09E-30	9.93E-30	Up	CARS2
NLGN1	13.5401136	-3.74982123	0.32561973	-11.5159523	1.10E-30	9.98E-30	Down	NLGN1
SLC9A2	878.438829	-2.21800211	0.19266004	-11.5125175	1.14E-30	1.04E-29	Down	SLC9A2
TDRD10	10.3768324	-1.70447917	0.14806695	-11.5115434	1.15E-30	1.05E-29	Down	TDRD10
PIP5K1B	1744.4566	-1.08214892	0.09401005	-11.5109922	1.16E-30	1.06E-29	Down	PIP5K1B
TCEAL6	1.74613387	-4.05371235	0.35224107	-11.5083466	1.20E-30	1.09E-29	Down	TCEAL6
FKBP10	3675.86602	1.90701091	0.16576602	11.5042326	1.26E-30	1.14E-29	Up	FKBP10
PRR5L	1691.80968	-1.06223923	0.09233827	-11.5037812	1.26E-30	1.15E-29	Down	PRR5L
TRIM71	29.8016665	5.9114727	0.51406684	11.4994244	1.33E-30	1.20E-29	Up	TRIM71
NIP7	1557.68664	0.75503387	0.06568427	11.4948968	1.40E-30	1.27E-29	Up	NIP7
SFPQ	10096.0017	0.53262531	0.04635338	11.4905382	1.47E-30	1.33E-29	Up	SFPQ
GPRASP1	148.858027	-1.88360357	0.1639354	-11.4899137	1.48E-30	1.34E-29	Down	GPRASP1

ACSM5	9.89668614	-2.73237315	0.23781129	-11.4896699	1.49E-30	1.35E-29	Down	ACSM5
DMRTA1	15.7755938	-3.25138519	0.28310838	-11.4845953	1.58E-30	1.43E-29	Down	DMRTA1
MADCAM1	59.3306517	-2.32838568	0.20276415	-11.4832216	1.60E-30	1.45E-29	Down	MADCAM1
SLC44A1	7226.42896	-0.78590874	0.06844364	-11.4825683	1.61E-30	1.46E-29	Down	SLC44A1
SRPRB	3170.24959	0.77948492	0.06790064	11.4797874	1.67E-30	1.51E-29	Up	SRPRB
SKP2	1154.72244	1.05117748	0.0915902	11.4769651	1.72E-30	1.55E-29	Up	SKP2
FCRL4	6.94584602	-3.25939541	0.28404374	-11.4749772	1.76E-30	1.59E-29	Down	FCRL4
OBP2B	9.98585328	5.77707595	0.50383321	11.4662469	1.95E-30	1.76E-29	Up	OBP2B
PTP4A1	7374.28308	-1.04961003	0.09161699	-11.4564993	2.18E-30	1.97E-29	Down	PTP4A1
MTG2	1682.57542	1.09599114	0.0956726	11.4556425	2.20E-30	1.98E-29	Up	MTG2
RNF32	163.506967	1.32893895	0.11603401	11.4530127	2.27E-30	2.04E-29	Up	RNF32
MASP1	214.092134	-3.27808655	0.28629508	-11.4500275	2.35E-30	2.12E-29	Down	MASP1
VENTX	158.823359	3.6446515	0.31833845	11.4489828	2.38E-30	2.14E-29	Up	VENTX
TAB2	2483.51873	-0.74535743	0.06510287	-11.4489177	2.38E-30	2.14E-29	Down	TAB2
NAA25	1121.27752	0.97957506	0.08556215	11.4486968	2.39E-30	2.14E-29	Up	NAA25
NR3C1	574.372909	-1.80501651	0.15773415	-11.4434097	2.54E-30	2.28E-29	Down	NR3C1
STMN2	168.788832	-3.06552785	0.26793173	-11.4414514	2.60E-30	2.33E-29	Down	STMN2
PXYLP1	631.663658	0.78449127	0.06857899	11.4392358	2.66E-30	2.39E-29	Up	PXYLP1
WDR5	2389.65898	0.75769336	0.06624488	11.4377657	2.71E-30	2.43E-29	Up	WDR5
MRTO4	2099.8764	0.97318269	0.08508776	11.437399	2.72E-30	2.44E-29	Up	MRTO4
EPHA6	8.78637945	-4.263557	0.37284241	-11.4352791	2.79E-30	2.50E-29	Down	EPHA6
P2RY8	155.556362	-1.70088431	0.14874231	-11.4351075	2.79E-30	2.50E-29	Down	P2RY8
SPNS3	118.405715	1.68938472	0.14775307	11.433838	2.83E-30	2.54E-29	Up	SPNS3
ALG3	2871.56908	1.09071961	0.09540469	11.432558	2.88E-30	2.57E-29	Up	ALG3
IRF2BP2	7878.32169	0.74872938	0.06549881	11.4311908	2.92E-30	2.61E-29	Up	IRF2BP2
MAF	741.541376	-1.66935869	0.14607587	-11.4280247	3.03E-30	2.71E-29	Down	MAF
RGS2	801.896692	-1.92483332	0.16843207	-11.4279499	3.03E-30	2.71E-29	Down	RGS2
CTSG	48.0230875	-3.32930019	0.29134834	-11.4272154	3.06E-30	2.73E-29	Down	CTSG
NAA10	2010.99421	1.3903705	0.12168224	11.4262401	3.09E-30	2.76E-29	Up	NAA10
DCTD	2925.55023	0.68605661	0.0600489	11.4249656	3.14E-30	2.80E-29	Up	DCTD
C1QTNF7	61.8587067	-2.69825639	0.23623663	-11.4218374	3.25E-30	2.90E-29	Down	C1QTNF7
SRCIN1	411.415025	1.86834826	0.16361298	11.4193153	3.35E-30	2.98E-29	Up	SRCIN1
RECK	209.40821	-1.42528627	0.12483985	-11.4169179	3.44E-30	3.07E-29	Down	RECK
ZNF746	913.856373	0.61949203	0.0542692	11.4151679	3.51E-30	3.13E-29	Up	ZNF746
RAB15	3209.86621	1.19548899	0.10473331	11.4146018	3.54E-30	3.15E-29	Up	RAB15
SPRR2A	18.1685486	6.18108907	0.54155853	11.41352	3.58E-30	3.18E-29	Up	SPRR2A
PCSK7	1878.82435	-1.12165275	0.09827834	-11.4130205	3.60E-30	3.20E-29	Down	PCSK7
TP53I3	1126.31052	-1.08809924	0.09535438	-11.4111086	3.68E-30	3.27E-29	Down	TP53I3
ACP1	3603.9123	0.62722536	0.05496683	11.4109798	3.69E-30	3.27E-29	Up	ACP1
IGF2	29481.5107	4.66505849	0.40884508	11.4103329	3.71E-30	3.30E-29	Up	IGF2
MATN3	113.654711	2.70847973	0.23739124	11.4093498	3.76E-30	3.33E-29	Up	MATN3
FERMT2	858.653293	-1.88864657	0.16559352	-11.4053172	3.93E-30	3.49E-29	Down	FERMT2
CLDN7	11884.1484	-1.09876083	0.09638739	-11.3994256	4.21E-30	3.73E-29	Down	CLDN7
KIF9	389.498652	1.00489618	0.08816699	11.3976467	4.30E-30	3.80E-29	Up	KIF9
CCNJ	470.775614	0.87883695	0.07711141	11.3969761	4.33E-30	3.83E-29	Up	CCNJ
UGGT2	907.655324	1.22999886	0.10794787	11.3943788	4.46E-30	3.95E-29	Up	UGGT2
FOSL1	564.047669	2.26443647	0.19873835	11.3940587	4.48E-30	3.96E-29	Up	FOSL1
NMU	181.896849	2.86989092	0.25198734	11.3890283	4.74E-30	4.19E-29	Up	NMU
PRPF3	1461.20169	0.68287454	0.05999895	11.3814406	5.17E-30	4.57E-29	Up	PRPF3
DNMT1	3590.30594	0.91774495	0.08064058	11.3806833	5.22E-30	4.61E-29	Up	DNMT1
PDPN	901.991333	1.75195363	0.15394872	11.3801114	5.25E-30	4.64E-29	Up	PDPN
ARF3	7643.44703	-0.64845931	0.05701305	-11.3738753	5.64E-30	4.98E-29	Down	ARF3
FOXS1	72.4890803	1.70913624	0.15028879	11.3723466	5.74E-30	5.06E-29	Up	FOXS1
WDR7	458.976789	-1.08518984	0.09543771	-11.3706612	5.85E-30	5.16E-29	Down	WDR7
RNF114	5502.43862	0.81306274	0.07151256	11.3695093	5.93E-30	5.23E-29	Up	RNF114
NEB	128.408979	2.84237833	0.25003119	11.368095	6.03E-30	5.31E-29	Up	NEB
GPR15	58.7623849	-3.48301469	0.30643302	-11.3663163	6.15E-30	5.41E-29	Down	GPR15
PCK1	2534.4103	-2.72539316	0.23980209	-11.3651767	6.23E-30	5.48E-29	Down	PCK1

GNG8	3.5840719	-2.50528512	0.22051275	-11.3611802	6.53E-30	5.74E-29	Down	GNG8
TP53RK	1416.66089	1.19877312	0.10555785	11.3565514	6.88E-30	6.05E-29	Up	TP53RK
SRMS	222.719317	2.21028449	0.19462811	11.3564504	6.89E-30	6.05E-29	Up	SRMS
REM2	37.7083907	1.73825886	0.15307935	11.35528	6.98E-30	6.13E-29	Up	REM2
F13A1	654.80884	-2.59075239	0.22823601	-11.351199	7.32E-30	6.42E-29	Down	F13A1
LCN12	160.519738	2.20235183	0.1940353	11.3502639	7.39E-30	6.48E-29	Up	LCN12
SLC22A17	204.42688	-1.74843976	0.15405659	-11.3493344	7.47E-30	6.55E-29	Down	SLC22A17
TMEM120B	532.033761	0.83815085	0.07385002	11.3493659	7.47E-30	6.55E-29	Up	TMEM120B
TTF2	1167.81354	0.81726825	0.07202908	11.3463646	7.73E-30	6.77E-29	Up	TTF2
SFRP4	1463.45003	3.08829828	0.27222842	11.3445109	7.90E-30	6.91E-29	Up	SFRP4
ASXL1	2967.57056	0.91916221	0.08102967	11.3435258	7.99E-30	6.99E-29	Up	ASXL1
ZMIZ2	5834.01941	1.01769638	0.08976807	11.3369531	8.61E-30	7.53E-29	Up	ZMIZ2
EVX1	248.901117	3.30165486	0.29125189	11.3360804	8.70E-30	7.60E-29	Up	EVX1
USP31	573.884579	0.95490118	0.08426488	11.3321369	9.10E-30	7.95E-29	Up	USP31
EEF1E1	378.248012	1.03746433	0.0915571	11.3313365	9.18E-30	8.01E-29	Up	EEF1E1
CHD7	2134.85509	1.11039197	0.09799311	11.3313271	9.18E-30	8.01E-29	Up	CHD7
FOXD1	75.700828	3.82268579	0.33740338	11.32972	9.35E-30	8.16E-29	Up	FOXD1
BLM	437.932648	1.1044459	0.09748263	11.3296685	9.36E-30	8.16E-29	Up	BLM
PBXIP1	2921.76475	-1.09181549	0.09637071	-11.3293287	9.39E-30	8.18E-29	Down	PBXIP1
APOBEC3A	35.2511723	-2.54932413	0.22502274	-11.3291845	9.41E-30	8.19E-29	Down	APOBEC3A
CDKN3	672.79366	1.19781248	0.10575323	11.3264863	9.70E-30	8.45E-29	Up	CDKN3
PHF14	1620.73331	0.84677252	0.07477199	11.3247283	9.90E-30	8.61E-29	Up	PHF14
ANKRD13D	939.836318	1.00476273	0.08876745	11.3190442	1.06E-29	9.19E-29	Up	ANKRD13D
SPNS2	2487.19469	1.7595398	0.15552783	11.3133434	1.13E-29	9.80E-29	Up	SPNS2
GINS3	370.853377	0.96040547	0.08489523	11.3128318	1.13E-29	9.85E-29	Up	GINS3
MFAP4	1803.38566	-2.16815526	0.19169928	-11.3101899	1.17E-29	1.01E-28	Down	MFAP4
STAP1	19.5156879	-2.51875188	0.22278407	-11.3057987	1.23E-29	1.07E-28	Down	STAP1
CD101	77.1912102	-1.3295466	0.11762518	-11.3032478	1.26E-29	1.10E-28	Down	CD101
MSTO1	300.304157	0.86409804	0.07645337	11.3022882	1.28E-29	1.11E-28	Up	MSTO1
PHOX2A	4.43452971	-3.51651046	0.31113191	-11.3023137	1.28E-29	1.11E-28	Down	PHOX2A
PRIM2	619.194271	0.77863205	0.06889695	11.3014016	1.29E-29	1.12E-28	Up	PRIM2
GRIA4	13.2613258	-2.66072423	0.23549275	-11.2985397	1.33E-29	1.16E-28	Down	GRIA4
TMEM130	28.1441643	-2.47703393	0.21924994	-11.2977634	1.35E-29	1.16E-28	Down	TMEM130
CMA1	13.5876862	-3.95921598	0.35047126	-11.296835	1.36E-29	1.18E-28	Down	CMA1
C2orf74	10.7069336	-1.97483978	0.17481408	-11.2968004	1.36E-29	1.18E-28	Down	C2orf74
DDX39A	4011.13244	1.15818641	0.10252607	11.296506	1.37E-29	1.18E-28	Up	DDX39A
VMA21	1769.41002	1.11314826	0.09854187	11.2961963	1.37E-29	1.18E-28	Up	VMA21
PODNL1	247.060268	1.79206854	0.15872452	11.2904331	1.46E-29	1.26E-28	Up	PODNL1
FNIP2	1415.34499	-1.12031573	0.09923612	-11.2893942	1.48E-29	1.28E-28	Down	FNIP2
LSG1	1787.02018	0.51973146	0.04605416	11.2852228	1.55E-29	1.34E-28	Up	LSG1
PWP1	1823.6084	0.5636043	0.0499475	11.2839341	1.58E-29	1.36E-28	Up	PWP1
NPEPL1	579.436783	1.30799728	0.11594091	11.2815852	1.62E-29	1.39E-28	Up	NPEPL1
DDX27	3750.26178	1.16641272	0.10340532	11.2800074	1.65E-29	1.42E-28	Up	DDX27
BDKRB2	702.929022	-1.2323832	0.10927641	-11.2776691	1.69E-29	1.46E-28	Down	BDKRB2
CAMTA2	1462.3325	-0.9417348	0.08350804	-11.2771756	1.70E-29	1.46E-28	Down	CAMTA2
UCKL1	2378.73159	1.35060532	0.11977932	11.2757809	1.73E-29	1.49E-28	Up	UCKL1
CYSLTR1	58.1482609	-1.79172952	0.15892666	-11.2739392	1.76E-29	1.52E-28	Down	CYSLTR1
AMOTL1	634.695057	-1.98395865	0.17598349	-11.2735498	1.77E-29	1.52E-28	Down	AMOTL1
SULT1C2	444.725766	2.56128385	0.22719659	11.2734258	1.78E-29	1.52E-28	Up	SULT1C2
HRCT1	310.214955	-2.27308101	0.20163877	-11.2730356	1.78E-29	1.53E-28	Down	HRCT1
ADCY3	1665.04458	1.06959321	0.0949008	11.2706451	1.83E-29	1.57E-28	Up	ADCY3
CNTNAP3	14.8892022	-2.72512948	0.24184761	-11.2679612	1.89E-29	1.62E-28	Down	CNTNAP3
SPINK2	10.9063283	-3.0647117	0.27205671	-11.2649737	1.95E-29	1.67E-28	Down	SPINK2
CD46	16187.1328	1.0500239	0.09322705	11.2630819	2.00E-29	1.71E-28	Up	CD46
PPP1R14B	2640.2658	0.91750774	0.08147505	11.2612117	2.04E-29	1.75E-28	Up	PPP1R14B
CNRIP1	163.792093	-1.46104334	0.12974441	-11.260935	2.05E-29	1.75E-28	Down	CNRIP1
LZTS1	188.891419	1.64935629	0.14648252	11.2597485	2.07E-29	1.77E-28	Up	LZTS1
MPP3	126.290786	1.40078011	0.12444779	11.2559661	2.16E-29	1.85E-28	Up	MPP3

DTYMK	1630.83053	1.0618319	0.09434933	11.2542597	2.21E-29	1.89E-28	Up		DTYMK
SLC11A2	3419.34172	1.14425086	0.10169067	11.2522704	2.26E-29	1.93E-28	Up		SLC11A2
DSG1	16.4326455	2.34406685	0.2083358	11.2513877	2.28E-29	1.95E-28	Up		DSG1
CCL15	186.336659	-1.49923317	0.13326749	-11.2498044	2.32E-29	1.98E-28	Down		CCL15
BDKRB1	66.1055904	-1.59876239	0.14214202	-11.2476408	2.38E-29	2.03E-28	Down		BDKRB1
MGST3	3618.52935	-0.86730296	0.07712545	-11.2453532	2.44E-29	2.08E-28	Down		MGST3
DPH2	1614.68323	0.95428433	0.08486609	11.2445888	2.46E-29	2.10E-28	Up		DPH2
MAT1A	128.087932	3.17733677	0.28265676	11.2409724	2.57E-29	2.19E-28	Up		MAT1A
PRKAR2B	278.717405	-1.99209054	0.17726144	-11.2381491	2.65E-29	2.25E-28	Down		PRKAR2B
RFT1	987.139141	0.6608391	0.05880336	11.2381179	2.65E-29	2.25E-28	Up		RFT1
SIX4	53.6983366	2.5150983	0.22380675	11.2378125	2.66E-29	2.26E-28	Up		SIX4
TMEM74	5.5314226	-2.4329705	0.21651097	-11.2371698	2.68E-29	2.28E-28	Down		TMEM74
IRF4	209.748785	-2.37779888	0.21169128	-11.2323892	2.83E-29	2.40E-28	Down		IRF4
SSPN	395.190821	-1.77633475	0.15816389	-11.2309752	2.87E-29	2.44E-28	Down		SSPN
DEPDC1B	546.411681	1.11046326	0.09887556	11.2309171	2.87E-29	2.44E-28	Up		DEPDC1B
GCSAML	10.3465406	-2.51293609	0.22380217	-11.2283811	2.96E-29	2.51E-28	Down		GCSAML
CIT	1125.13917	1.01365107	0.09028333	11.2274448	2.99E-29	2.54E-28	Up		CIT
FAM13B	645.480606	-1.04902562	0.09343484	-11.2273499	2.99E-29	2.54E-28	Down		FAM13B
EHHADH	726.225631	-1.14831036	0.1023306	-11.2215741	3.20E-29	2.71E-28	Down		EHHADH
FAM131C	19.1096136	2.64016553	0.23528676	11.2210545	3.21E-29	2.72E-28	Up		FAM131C
EID1	2733.33272	-0.94062759	0.08383109	-11.2205095	3.23E-29	2.74E-28	Down		EID1
CASP5	248.987774	-2.10612831	0.18773336	-11.2187219	3.30E-29	2.79E-28	Down		CASP5
ICA1	2329.09583	0.95565488	0.08519691	11.2170134	3.36E-29	2.85E-28	Up		ICA1
SLC25A29	961.799521	1.32327986	0.11800632	11.2136359	3.50E-29	2.95E-28	Up		SLC25A29
MBNL1	3947.419	-0.94289093	0.08410011	-11.2115298	3.58E-29	3.02E-28	Down		MBNL1
BAAT	23.4239727	3.9923406	0.35627861	11.2056702	3.82E-29	3.23E-28	Up		BAAT
FASN	12716.467	1.44158583	0.12866454	11.2042201	3.89E-29	3.28E-28	Up		FASN
ASPM	956.209238	1.38293039	0.12346823	11.200698	4.05E-29	3.41E-28	Up		ASPM
CCL28	1170.69944	-2.13184934	0.19035922	-11.1990862	4.12E-29	3.47E-28	Down		CCL28
PAXIP1	846.033558	0.67170135	0.05998251	11.1982866	4.16E-29	3.50E-28	Up		PAXIP1
ZNF670	13.3812244	1.81353642	0.16197651	11.1962928	4.25E-29	3.58E-28	Up		ZNF670
PPP1R16B	295.424459	-1.74536475	0.15592159	-11.1938622	4.37E-29	3.68E-28	Down		PPP1R16B
KRTAP5-4	4.74912225	4.14009345	0.36985519	11.1938227	4.37E-29	3.68E-28	Up		KRTAP5-4
RXRA	2838.33168	-0.95055286	0.08492426	-11.1929482	4.42E-29	3.71E-28	Down		RXRA
CHAC1	341.8428	1.70850422	0.15264258	11.1928419	4.42E-29	3.72E-28	Up		CHAC1
LIG1	2029.72171	0.90712251	0.08105321	11.191691	4.48E-29	3.76E-28	Up		LIG1
PARVA	2814.72479	-0.74852997	0.06688929	-11.1905799	4.53E-29	3.81E-28	Down		PARVA
GHITM	9311.41041	-0.75872116	0.06780676	-11.1894627	4.59E-29	3.86E-28	Down		GHITM
RCBTB1	925.562811	1.02366494	0.09149917	11.1876963	4.68E-29	3.93E-28	Up		RCBTB1
NUSAP1	1688.85991	1.0831613	0.09682276	11.1870524	4.72E-29	3.96E-28	Up		NUSAP1
TRMT112	3828.05325	0.95334478	0.08522634	11.1860339	4.77E-29	4.00E-28	Up		TRMT112
CYP4X1	366.461311	2.90637422	0.25983099	11.1856333	4.79E-29	4.02E-28	Up		CYP4X1
FANCG	874.258331	0.95860992	0.08572275	11.1826774	4.96E-29	4.15E-28	Up		FANCG
MEF2D	2185.05758	-0.91105253	0.08147511	-11.181973	5.00E-29	4.18E-28	Down		MEF2D
CLNS1A	3299.82636	0.66253548	0.05925846	11.1804372	5.08E-29	4.26E-28	Up		CLNS1A
ECI2	979.925223	-1.41411498	0.12648815	-11.1798215	5.12E-29	4.28E-28	Down		ECI2
WDR46	2127.5685	0.85257638	0.07627836	11.177172	5.27E-29	4.41E-28	Up		WDR46
MPND	945.909897	-1.06750034	0.09551331	-11.1764568	5.32E-29	4.44E-28	Down		MPND
CPXM2	428.24954	-2.45601245	0.21978042	-11.1748466	5.41E-29	4.52E-28	Down		CPXM2
IGSF11	8.23130967	-3.24410194	0.29031499	-11.174421	5.44E-29	4.54E-28	Down		IGSF11
RPL22L1	2045.55128	1.90115285	0.17014229	11.1738996	5.47E-29	4.57E-28	Up		RPL22L1
DMPK	1020.48657	-1.35407943	0.12120009	-11.172264	5.57E-29	4.65E-28	Down		DMPK
PDCD2	2273.26965	0.70882898	0.06344823	11.1717682	5.61E-29	4.67E-28	Up		PDCD2
ZNF783	666.820183	0.99376368	0.08895535	11.1714892	5.62E-29	4.69E-28	Up		ZNF783
ISM2	98.5190933	3.85591738	0.34525528	11.1683081	5.83E-29	4.86E-28	Up		ISM2
UBXN11	639.363591	1.18893354	0.10649197	11.1645372	6.08E-29	5.06E-28	Up		UBXN11
POLA1	1113.29265	0.96732226	0.08664484	11.164223	6.10E-29	5.08E-28	Up		POLA1
HK2	5729.6494	-1.25463967	0.11238951	-11.1633161	6.16E-29	5.13E-28	Down		HK2

ADRB2	30.2210424	-1.95666747	0.17528361	-11.1628658	6.20E-29	5.15E-28	Down	ADRB2
DDX51	1041.2008	0.90667946	0.08123095	11.1617487	6.27E-29	5.22E-28	Up	DDX51
MAGEA12	61.6945677	9.41332816	0.84353749	11.1593477	6.45E-29	5.36E-28	Up	MAGEA12
RAB5B	5652.39059	-0.63929254	0.05730412	-11.1561352	6.68E-29	5.55E-28	Down	RAB5B
IRAK1	8589.37525	1.11811605	0.10025883	11.1522946	6.98E-29	5.79E-28	Up	IRAK1
SLC52A1	51.220722	-2.54081741	0.22789692	-11.1489766	7.24E-29	6.01E-28	Down	SLC52A1
FOXA2	1624.49572	1.44907884	0.12999019	11.1476013	7.36E-29	6.10E-28	Up	FOXA2
FBL	8394.23466	1.15632892	0.10375791	11.1444895	7.62E-29	6.31E-28	Up	FBL
EPHB3	7021.85607	1.96826216	0.17664661	11.1423717	7.80E-29	6.46E-28	Up	EPHB3
KLK11	376.928017	3.24800962	0.29155753	11.1402015	7.99E-29	6.62E-28	Up	KLK11
CEP164	818.751806	0.78249957	0.07024431	11.1396857	8.04E-29	6.66E-28	Up	CEP164
DDIT4	3033.5441	1.50288419	0.13491618	11.1393917	8.07E-29	6.67E-28	Up	DDIT4
ZNF229	18.1803937	-2.11165063	0.18956659	-11.1393606	8.07E-29	6.67E-28	Down	ZNF229
CORO2B	51.2286424	-2.17372458	0.19516717	-11.1377571	8.22E-29	6.79E-28	Down	CORO2B
SLC2A1	9718.36179	1.79553335	0.16121891	11.1372376	8.26E-29	6.83E-28	Up	SLC2A1
OSBPL11	654.093416	-0.68654112	0.06166939	-11.1326079	8.71E-29	7.19E-28	Down	OSBPL11
AFG3L2	2281.37488	-0.87094568	0.07823762	-11.1320576	8.76E-29	7.23E-28	Down	AFG3L2
OLA1	4169.15351	0.86076296	0.07733096	11.1308972	8.87E-29	7.32E-28	Up	OLA1
SNRK	963.173207	-0.84339834	0.0757878	-11.1284186	9.12E-29	7.52E-28	Down	SNRK
PPP1R3C	101.64177	-2.32087947	0.20860469	-11.1257302	9.40E-29	7.75E-28	Down	PPP1R3C
UTP6	1680.32621	0.66426354	0.05971011	11.1248078	9.50E-29	7.83E-28	Up	UTP6
ATCAY	9.07841675	-3.20346448	0.2881084	-11.1189553	1.01E-28	8.36E-28	Down	ATCAY
KLF3	4285.27288	-0.77332519	0.06958169	-11.1139174	1.07E-28	8.84E-28	Down	KLF3
PRSS41	10.4331508	3.95318696	0.35574552	11.1124013	1.09E-28	8.98E-28	Up	PRSS41
SIGLEC8	31.1690338	-2.31504479	0.208363	-11.1106331	1.11E-28	9.16E-28	Down	SIGLEC8
NKAPL	5.23163197	-2.17753273	0.19598884	-11.1104934	1.12E-28	9.17E-28	Down	NKAPL
EIF5A2	357.992507	1.17891027	0.10613218	11.1079437	1.15E-28	9.43E-28	Up	EIF5A2
FCRLA	42.7718277	-2.93206972	0.26397542	-11.1073589	1.16E-28	9.49E-28	Down	FCRLA
KCTD14	623.025479	1.15837326	0.10430348	11.1057968	1.18E-28	9.65E-28	Up	KCTD14
ARHGAP39	852.850855	1.21709159	0.1096036	11.1044855	1.19E-28	9.79E-28	Up	ARHGAP39
FAM189A2	43.6817888	-2.31481407	0.20848878	-11.1028235	1.22E-28	9.97E-28	Down	FAM189A2
CDON	183.770807	-1.61521486	0.14548768	-11.1020732	1.23E-28	1.00E-27	Down	CDON
CACNA1H	816.478	-1.93358771	0.17416488	-11.1020526	1.23E-28	1.00E-27	Down	CACNA1H
FRMD4B	578.378517	-1.10647154	0.09966406	-11.1020114	1.23E-28	1.00E-27	Down	FRMD4B
FAM163B	12.0075035	-2.95778785	0.26643413	-11.1013852	1.24E-28	1.01E-27	Down	FAM163B
F7	81.1751206	3.6391471	0.32788618	11.098812	1.27E-28	1.04E-27	Up	F7
NCL	26254.9641	0.7995305	0.07206525	11.0945354	1.33E-28	1.09E-27	Up	NCL
CENPO	541.604306	0.71591906	0.06456156	11.0889373	1.42E-28	1.16E-27	Up	CENPO
IGF2BP3	150.808267	4.01436174	0.36203466	11.0883353	1.43E-28	1.17E-27	Up	IGF2BP3
HVCN1	114.097758	-1.35796947	0.12253641	-11.0821715	1.53E-28	1.25E-27	Down	HVCN1
PTPN9	1614.34993	-0.55799969	0.05035493	-11.0813311	1.55E-28	1.26E-27	Down	PTPN9
SIPA1L2	1373.19939	-1.27858429	0.11539368	-11.0801935	1.57E-28	1.28E-27	Down	SIPA1L2
EREG	1976.63897	2.9228001	0.2637861	11.08019	1.57E-28	1.28E-27	Up	EREG
F2	22.7852647	3.73659854	0.33724023	11.0799312	1.57E-28	1.28E-27	Up	F2
REEP6	881.518071	1.97401026	0.17816849	11.0794579	1.58E-28	1.29E-27	Up	REEP6
TSPAN3	13164.2824	-0.90608649	0.08178965	-11.0782534	1.60E-28	1.30E-27	Down	TSPAN3
SPOP	1839.24374	-0.64688681	0.05839306	-11.078146	1.60E-28	1.30E-27	Down	SPOP
TRMU	1050.55185	0.93225885	0.0841636	11.0767463	1.63E-28	1.32E-27	Up	TRMU
NIPAL3	1442.63057	-0.72798019	0.06572629	-11.0759368	1.64E-28	1.34E-27	Down	NIPAL3
GNAI1	476.562141	-1.51811901	0.13708119	-11.0745974	1.67E-28	1.35E-27	Down	GNAI1
SOX1	76.4116787	4.81466786	0.434752	11.0745158	1.67E-28	1.36E-27	Up	SOX1
HOGA1	26.3479003	2.82892394	0.25547376	11.0732468	1.69E-28	1.37E-27	Up	HOGA1
ATP1B3	6578.32737	-0.82329847	0.07436956	-11.0703697	1.75E-28	1.42E-27	Down	ATP1B3
SOCS6	1122.43346	-1.05183352	0.09502189	-11.0693806	1.77E-28	1.43E-27	Down	SOCS6
ANKRD33B	61.7114134	-1.89338925	0.17115803	-11.0622287	1.91E-28	1.55E-27	Down	ANKRD33B
DFFB	460.266399	0.78818473	0.07125878	11.0608786	1.94E-28	1.57E-27	Up	DFFB
TMEM201	974.380297	1.01192243	0.09149539	11.059819	1.96E-28	1.59E-27	Up	TMEM201
PHF19	1797.74791	0.99336151	0.08982854	11.0584178	2.00E-28	1.62E-27	Up	PHF19

FAM72D	24.3748076	1.75341062	0.15857597	11.0572276	2.02E-28	1.64E-27	Up	FAM72D
RNF8	767.06038	0.60899996	0.05508834	11.0549696	2.07E-28	1.68E-27	Up	RNF8
FEN1	1786.30962	0.94647292	0.08562003	11.0543405	2.09E-28	1.69E-27	Up	FEN1
UAP1	2666.49579	-0.82437604	0.07459714	-11.0510403	2.17E-28	1.75E-27	Down	UAP1
NNAT	96.5239093	-2.3831915	0.21570777	-11.0482413	2.24E-28	1.81E-27	Down	NNAT
CFP	75.7656044	-1.72398672	0.1560574	-11.0471323	2.26E-28	1.83E-27	Down	CFP
ZNRF3	26.1391479	1.66714325	0.15092029	11.0465148	2.28E-28	1.84E-27	Up	ZNRF3
SLC8A1	375.50179	-1.65135551	0.14956528	-11.0410352	2.42E-28	1.96E-27	Down	SLC8A1
SLC1A1	722.467839	-1.84284671	0.16691977	-11.0403144	2.44E-28	1.97E-27	Down	SLC1A1
PLEKHS1	1153.21859	1.77352791	0.16065284	11.0395053	2.46E-28	1.99E-27	Up	PLEKHS1
POLA2	1045.79175	0.88354939	0.08004459	11.0382147	2.50E-28	2.01E-27	Up	POLA2
SPECC1	1010.32679	-1.19853472	0.10860774	-11.0354451	2.58E-28	2.08E-27	Down	SPECC1
DES	11628.8124	-3.75428093	0.34029327	-11.0324867	2.66E-28	2.15E-27	Down	DES
CD160	22.1733935	-2.13500972	0.19352698	-11.0321039	2.68E-28	2.15E-27	Down	CD160
R3HDM1	1418.84267	0.73808663	0.06691163	11.0307672	2.72E-28	2.18E-27	Up	R3HDM1
HAUS6	828.082648	1.01076459	0.09165326	11.028136	2.80E-28	2.25E-27	Up	HAUS6
LRRC3B	1.55765845	-3.39902644	0.30823114	-11.0275244	2.82E-28	2.26E-27	Down	LRRC3B
PPP1R1A	51.1227946	-3.40714182	0.30905227	-11.024484	2.91E-28	2.34E-27	Down	PPP1R1A
RILP	438.122261	-1.37055872	0.12432175	-11.0242875	2.92E-28	2.34E-27	Down	RILP
SLC1A5	9355.29326	1.19544195	0.10843959	11.0240357	2.93E-28	2.35E-27	Up	SLC1A5
ZG16	5619.57142	-4.39333317	0.39852351	-11.0240252	2.93E-28	2.35E-27	Down	ZG16
TLN1	11348.8422	-0.96425378	0.08747597	-11.0230711	2.96E-28	2.37E-27	Down	TLN1
MYL6	28475.3413	-0.99832871	0.09058799	-11.0205413	3.04E-28	2.44E-27	Down	MYL6
PPM1G	6204.37562	0.71852975	0.06520225	11.0200143	3.06E-28	2.45E-27	Up	PPM1G
CLMP	472.190849	-2.00180176	0.18165992	-11.0195018	3.08E-28	2.47E-27	Down	CLMP
NUGGC	37.8058276	-2.49729638	0.22669235	-11.0162357	3.19E-28	2.56E-27	Down	NUGGC
PRDM6	69.4600575	-2.24238111	0.2035732	-11.0151097	3.23E-28	2.59E-27	Down	PRDM6
ASF1B	1659.86286	1.06077162	0.09631589	11.0134643	3.29E-28	2.63E-27	Up	ASF1B
GRK1	16.6758862	3.60332803	0.32729089	11.0095578	3.44E-28	2.75E-27	Up	GRK1
SNX24	202.524171	-1.34916401	0.12256044	-11.0081522	3.49E-28	2.79E-27	Down	SNX24
TNFSF12	410.626958	-1.31193994	0.11918043	-11.0080149	3.50E-28	2.79E-27	Down	TNFSF12
NR1H4	74.0314604	-3.71938222	0.33806752	-11.0018916	3.74E-28	2.99E-27	Down	NR1H4
GSDMA	119.3139	2.31362163	0.21030318	11.0013633	3.76E-28	3.00E-27	Up	GSDMA
SLC5A7	7.84048638	-4.2836068	0.38937505	-11.001236	3.77E-28	3.01E-27	Down	SLC5A7
NEUROG2	14.6271323	5.9872104	0.54424447	11.0009576	3.78E-28	3.01E-27	Up	NEUROG2
CLDN9	74.4240015	2.66014644	0.24182816	11.0001518	3.81E-28	3.04E-27	Up	CLDN9
TBC1D31	393.892906	1.02537562	0.09323442	10.997823	3.91E-28	3.12E-27	Up	TBC1D31
SOWAHB	473.496082	-1.11750123	0.10161146	-10.9977875	3.92E-28	3.12E-27	Down	SOWAHB
KCP	107.450104	1.83672473	0.16701272	10.997514	3.93E-28	3.13E-27	Up	KCP
PSMG4	762.438337	1.17692601	0.10701891	10.997365	3.93E-28	3.13E-27	Up	PSMG4
FOXD3	6.76537757	-3.48823259	0.31733562	-10.9922503	4.16E-28	3.31E-27	Down	FOXD3
PPARD	2159.5249	-0.99101752	0.09018264	-10.9890058	4.32E-28	3.43E-27	Down	PPARD
RDH16	25.0970808	2.2847951	0.20793927	10.9877999	4.37E-28	3.48E-27	Up	RDH16
PRICKLE2	234.796437	-1.75781231	0.15998937	-10.987057	4.41E-28	3.50E-27	Down	PRICKLE2
DAZAP2	10095.0921	-0.5562725	0.05064417	-10.9839403	4.57E-28	3.62E-27	Down	DAZAP2
RGL1	485.501572	-1.30246688	0.11858431	-10.9834674	4.59E-28	3.64E-27	Down	RGL1
ACTL6A	2752.65811	0.77748943	0.0707994	10.9815818	4.69E-28	3.72E-27	Up	ACTL6A
DCAF5	1408.93119	-0.58196415	0.0529949	-10.9815114	4.69E-28	3.72E-27	Down	DCAF5
MEF2C	434.367212	-1.58200155	0.14407381	-10.9804937	4.74E-28	3.76E-27	Down	MEF2C
UNC5D	7.12442533	-2.99055955	0.27237956	-10.9793831	4.80E-28	3.80E-27	Down	UNC5D
CALD1	7776.75025	-1.94819006	0.17746228	-10.9780516	4.87E-28	3.86E-27	Down	CALD1
MEX3D	1224.38043	1.34062738	0.12212935	10.9771105	4.92E-28	3.90E-27	Up	MEX3D
MYZAP	139.57921	-1.31538482	0.11985616	-10.9746949	5.06E-28	4.00E-27	Down	MYZAP
MAST2	3146.96707	-0.93559186	0.08531701	-10.9660641	5.56E-28	4.40E-27	Down	MAST2
KLB	32.6801783	-2.03478654	0.18556082	-10.9656042	5.59E-28	4.42E-27	Down	KLB
ITPR1	483.901723	-1.50865748	0.1376632	-10.9590472	6.01E-28	4.75E-27	Down	ITPR1
DXO	516.175636	0.92804012	0.08468406	10.9588524	6.03E-28	4.76E-27	Up	DXO
SERPIND1	57.9168997	3.64248525	0.33238383	10.9586716	6.04E-28	4.77E-27	Up	SERPIND1

IGSF9	926.009007	-1.56666513	0.14296379	-10.9584747	6.05E-28	4.77E-27	Down	IGSF9
MUC5AC	1438.26654	4.23476176	0.38646973	10.9575509	6.11E-28	4.82E-27	Up	MUC5AC
EYA2	171.380184	-2.7577583	0.25175306	-10.9542196	6.34E-28	5.00E-27	Down	EYA2
SH3BP4	3658.16047	0.95927835	0.08761013	10.9494003	6.69E-28	5.27E-27	Up	SH3BP4
CD48	320.898366	-1.85408132	0.16936439	-10.9472911	6.85E-28	5.39E-27	Down	CD48
SPIN4	613.637244	1.07734818	0.09841552	10.9469333	6.87E-28	5.41E-27	Up	SPIN4
B4GALNT2	703.0245	-4.16472277	0.38050546	-10.945238	7.00E-28	5.51E-27	Down	B4GALNT2
GTF2F2	1683.36468	1.05502945	0.09642792	10.9411197	7.33E-28	5.76E-27	Up	GTF2F2
TSPYL5	140.821518	-1.73524379	0.15859908	-10.9410709	7.33E-28	5.76E-27	Down	TSPYL5
LGALS3	28967.6492	-1.17289426	0.10720923	-10.9402362	7.40E-28	5.82E-27	Down	LGALS3
PCOLCE2	73.3705789	-3.27614912	0.29950812	-10.9384317	7.55E-28	5.93E-27	Down	PCOLCE2
NAP1L1	10972.384	0.96816247	0.08857677	10.930207	8.27E-28	6.49E-27	Up	NAP1L1
GRAMD4	2198.97685	-0.98296183	0.08993407	-10.9298045	8.30E-28	6.52E-27	Down	GRAMD4
LONRF2	47.0213604	-3.68752613	0.33743089	-10.928241	8.45E-28	6.63E-27	Down	LONRF2
ENGASE	2988.1083	1.50555235	0.13777422	10.9276779	8.50E-28	6.66E-27	Up	ENGASE
CCNA2	1722.41014	1.05978036	0.09699232	10.9264359	8.62E-28	6.75E-27	Up	CCNA2
LRRC20	699.223914	0.96324935	0.08816114	10.9260083	8.66E-28	6.78E-27	Up	LRRC20
SKIDA1	11.3625431	-1.92741386	0.17642211	-10.9250131	8.75E-28	6.85E-27	Down	SKIDA1
SLC34A3	20.0578622	2.82865564	0.25892651	10.9245502	8.80E-28	6.89E-27	Up	SLC34A3
CITED1	53.1995622	2.65774778	0.2432872	10.9243222	8.82E-28	6.90E-27	Up	CITED1
SLC46A3	1057.3002	-1.30665371	0.11964267	-10.9213016	9.12E-28	7.13E-27	Down	SLC46A3
AQP7	116.737437	-1.91917887	0.17577636	-10.9182994	9.42E-28	7.37E-27	Down	AQP7
IGSF10	61.0065966	-2.66922194	0.24452325	-10.9160252	9.66E-28	7.55E-27	Down	IGSF10
BIN2	249.82482	-1.17128222	0.10730486	-10.915463	9.72E-28	7.59E-27	Down	BIN2
WLS	2449.65292	-0.93126903	0.08531802	-10.9152674	9.74E-28	7.60E-27	Down	WLS
MZT1	1209.13852	1.29481504	0.11862433	10.9152568	9.75E-28	7.60E-27	Up	MZT1
SERPINB7	23.7278006	4.42835697	0.40586927	10.9107965	1.02E-27	7.98E-27	Up	SERPINB7
APOA2	32.8239363	5.92710494	0.54324008	10.9106547	1.03E-27	7.99E-27	Up	APOA2
GIMAP6	385.32737	-1.50307848	0.13778397	-10.9089501	1.04E-27	8.14E-27	Down	GIMAP6
NAP1L3	43.1747334	-2.0915254	0.19173298	-10.9085325	1.05E-27	8.17E-27	Down	NAP1L3
LGALS2	377.980405	-2.64062355	0.2422002	-10.9026479	1.12E-27	8.72E-27	Down	LGALS2
FBF1	130.685533	1.13100342	0.1037409	10.902194	1.13E-27	8.76E-27	Up	FBF1
HDHD2	531.630015	-0.72995398	0.06696286	-10.9008778	1.14E-27	8.88E-27	Down	HDHD2
AURKB	1112.12221	1.23460736	0.11326244	10.9004125	1.15E-27	8.92E-27	Up	AURKB
CKMT2	555.920975	3.45611833	0.31708563	10.8996373	1.16E-27	8.99E-27	Up	CKMT2
FAM53A	65.0492108	1.58955491	0.14587222	10.8968993	1.19E-27	9.27E-27	Up	FAM53A
MTMR3	223.531906	-0.73577053	0.06752904	-10.8956168	1.21E-27	9.39E-27	Down	MTMR3
CCDC57	894.07093	0.95325791	0.08750968	10.893171	1.24E-27	9.64E-27	Up	CCDC57
SLC35E4	750.689999	1.50365179	0.13806469	10.890922	1.27E-27	9.88E-27	Up	SLC35E4
POLR3G	187.855095	1.48868432	0.13670724	10.8895795	1.29E-27	1.00E-26	Up	POLR3G
NUMB	2693.5368	-0.63104373	0.05794936	-10.8895725	1.29E-27	1.00E-26	Down	NUMB
FAM71E1	111.727884	1.43584693	0.13191259	10.8848364	1.36E-27	1.05E-26	Up	FAM71E1
PLA2G3	77.4831728	4.37580861	0.40205509	10.8836047	1.38E-27	1.07E-26	Up	PLA2G3
TBL2	2443.10621	0.69904934	0.06423428	10.8828077	1.39E-27	1.08E-26	Up	TBL2
TBRG4	5212.70543	0.97181527	0.08929863	10.8827572	1.39E-27	1.08E-26	Up	TBRG4
C16orf46	40.78374	1.01432167	0.09322151	10.8807686	1.42E-27	1.10E-26	Up	C16orf46
LIPG	1320.94441	1.32668773	0.12193813	10.8800072	1.44E-27	1.11E-26	Up	LIPG
NLGN4X	51.4720938	-2.09987954	0.19301047	-10.8796146	1.44E-27	1.11E-26	Down	NLGN4X
DLX6	35.8076969	4.05073778	0.37233426	10.8793046	1.45E-27	1.12E-26	Up	DLX6
SCG2	146.0374	-2.35588346	0.21656975	-10.878174	1.46E-27	1.13E-26	Down	SCG2
MSLN	2744.30839	2.98208951	0.27415072	10.877555	1.47E-27	1.14E-26	Up	MSLN
OCEL1	484.94709	-1.06380914	0.09780557	-10.8767745	1.49E-27	1.15E-26	Down	OCEL1
ATR	1126.1121	0.9186763	0.08446349	10.8766087	1.49E-27	1.15E-26	Up	ATR
TMEM170A	1313.56231	-0.8049948	0.07401213	-10.876526	1.49E-27	1.15E-26	Down	TMEM170A
SCN3A	25.7160079	-2.1848819	0.20094577	-10.872993	1.55E-27	1.19E-26	Down	SCN3A
SPATS2L	3970.70819	-0.69876268	0.06431199	-10.8652009	1.69E-27	1.30E-26	Down	SPATS2L
CTU1	306.655155	1.90653115	0.17555294	10.8601492	1.78E-27	1.37E-26	Up	CTU1
IGFL2	95.8478732	3.37764445	0.31103215	10.8594703	1.80E-27	1.38E-26	Up	IGFL2

GCNT4	72.4717242	-2.15524462	0.19848752	-10.8583379	1.82E-27	1.40E-26	Down	GCNT4
ZNF367	493.174138	1.03283073	0.09512904	10.8571551	1.84E-27	1.42E-26	Up	ZNF367
SCUBE1	129.152113	-1.9092405	0.17585717	-10.8567679	1.85E-27	1.42E-26	Down	SCUBE1
CCNI2	535.651806	1.31547245	0.12119431	10.8542428	1.90E-27	1.46E-26	Up	CCNI2
ORM1	73.4735803	5.74821771	0.52961826	10.8535113	1.92E-27	1.47E-26	Up	ORM1
DOK7	112.558324	2.10392595	0.19385089	10.853321	1.92E-27	1.48E-26	Up	DOK7
TACC3	2281.0826	1.0434699	0.09616901	10.8503759	1.99E-27	1.52E-26	Up	TACC3
FAM120AOS	1611.3389	-0.66032105	0.06085723	-10.8503299	1.99E-27	1.52E-26	Down	FAM120AOS
NUP35	528.433455	0.78222012	0.07213474	10.8438751	2.13E-27	1.63E-26	Up	NUP35
PPP1R13L	1396.42815	1.27837143	0.11794158	10.8390227	2.25E-27	1.72E-26	Up	PPP1R13L
EDNRA	477.444	1.85413151	0.17108485	10.8374965	2.29E-27	1.75E-26	Up	EDNRA
FAXDC2	579.946361	-1.78032637	0.16440703	-10.8287728	2.52E-27	1.93E-26	Down	FAXDC2
FRMD3	285.700471	-1.97288828	0.18223166	-10.8262653	2.58E-27	1.98E-26	Down	FRMD3
CCDC148	16.044952	2.14825179	0.19855354	10.8195087	2.78E-27	2.13E-26	Up	CCDC148
NUP188	2952.69748	0.82532539	0.07629489	10.817571	2.84E-27	2.17E-26	Up	NUP188
HNRNPA1	25382.9569	0.66038366	0.06107241	10.8131267	2.98E-27	2.28E-26	Up	HNRNPA1
LY9	60.2303674	-2.09794679	0.19402762	-10.8126192	3.00E-27	2.29E-26	Down	LY9
BRSK2	225.651957	2.74803306	0.25424336	10.8086721	3.13E-27	2.39E-26	Up	BRSK2
TAPT1	632.706724	-0.72873715	0.0674446	-10.8049738	3.26E-27	2.49E-26	Down	TAPT1
VWA5A	1435.85758	-1.43108516	0.13245949	-10.8039458	3.30E-27	2.52E-26	Down	VWA5A
HIPK4	3.92292979	-2.05857022	0.19059106	-10.8009803	3.41E-27	2.60E-26	Down	HIPK4
MAN1A1	2246.66501	-1.34641834	0.12471535	-10.795931	3.60E-27	2.74E-26	Down	MAN1A1
CCDC85B	1376.99803	1.94661296	0.18043929	10.788188	3.91E-27	2.98E-26	Up	CCDC85B
HDAC8	644.226136	0.74656309	0.06920401	10.7878591	3.93E-27	2.99E-26	Up	HDAC8
MALSU1	902.880113	0.86504588	0.08019019	10.7874273	3.95E-27	3.00E-26	Up	MALSU1
FLNB	16008.4011	-1.01422852	0.09404834	-10.7841199	4.09E-27	3.11E-26	Down	FLNB
C2CD2L	804.467739	-0.81973591	0.07601402	-10.7840099	4.10E-27	3.12E-26	Down	C2CD2L
TREM1	157.587789	2.63698527	0.24472469	10.7753134	4.50E-27	3.42E-26	Up	TREM1
PPP2R2B	29.0842841	-2.12000939	0.19680281	-10.7722516	4.65E-27	3.54E-26	Down	PPP2R2B
BMP5	90.6544594	-2.57775147	0.23932609	-10.7708754	4.72E-27	3.59E-26	Down	BMP5
HS3ST6	4.96040383	-4.14384841	0.38475703	-10.7700396	4.77E-27	3.62E-26	Down	HS3ST6
RASGRP2	131.026757	-1.66180889	0.15433674	-10.7674223	4.91E-27	3.72E-26	Down	RASGRP2
ZEB1	664.655975	-1.82092737	0.16912122	-10.7669951	4.93E-27	3.74E-26	Down	ZEB1
RIC3	17.2772651	-2.91156725	0.27045978	-10.7652503	5.02E-27	3.81E-26	Down	RIC3
PTTG1IP	9998.51808	-0.69037762	0.0641432	-10.7630673	5.14E-27	3.90E-26	Down	PTTG1IP
APOBEC3B	316.601096	-1.57839439	0.14668207	-10.7606497	5.28E-27	4.00E-26	Down	APOBEC3B
AGTRAP	1336.16791	1.0933404	0.10160799	10.7603785	5.30E-27	4.01E-26	Up	AGTRAP
DLGAP5	812.739494	1.12331809	0.1044071	10.7590204	5.37E-27	4.07E-26	Up	DLGAP5
ODC1	8186.43414	1.02625978	0.09539594	10.7578983	5.44E-27	4.12E-26	Up	ODC1
DPM2	1868.97809	0.9230258	0.08580789	10.756887	5.50E-27	4.16E-26	Up	DPM2
MYO1C	8069.43914	-0.90420637	0.08407918	-10.7542246	5.66E-27	4.28E-26	Down	MYO1C
ZNF469	307.559388	1.94869826	0.18132314	10.7471017	6.12E-27	4.62E-26	Up	ZNF469
CCNL2	3098.85494	1.05385993	0.09812565	10.7399026	6.61E-27	5.00E-26	Up	CCNL2
TMEM39A	1216.80106	0.60120311	0.05598071	10.7394686	6.64E-27	5.02E-26	Up	TMEM39A
LY6E	5644.94235	1.95575902	0.18212101	10.7387883	6.69E-27	5.05E-26	Up	LY6E
ZNF7	701.770653	0.79471916	0.07401072	10.7378931	6.76E-27	5.10E-26	Up	ZNF7
CHRDL1	434.210334	-3.16606736	0.29489873	-10.7361174	6.89E-27	5.20E-26	Down	CHRDL1
NEXN	394.513503	-2.19178969	0.20419243	-10.7339422	7.05E-27	5.32E-26	Down	NEXN
ALAD	1763.63085	-0.73892267	0.06884363	-10.7333481	7.10E-27	5.35E-26	Down	ALAD
NHSL1	1959.01747	-1.01351253	0.09442834	-10.7331398	7.11E-27	5.36E-26	Down	NHSL1
PPP1R35	811.39135	1.27145036	0.11846942	10.7323089	7.18E-27	5.40E-26	Up	PPP1R35
TMTC4	1428.16126	1.02673488	0.09568583	10.7302715	7.34E-27	5.52E-26	Up	TMTC4
PREB	3082.23415	0.72131018	0.06724089	10.7272556	7.58E-27	5.70E-26	Up	PREB
EPHX2	1654.61208	-1.47657804	0.13766238	-10.7260828	7.68E-27	5.77E-26	Down	EPHX2
RIPK3	717.647627	-0.88039746	0.08208863	-10.7249618	7.77E-27	5.84E-26	Down	RIPK3
MLXIP	8229.41728	-0.92284958	0.08607095	-10.7219633	8.03E-27	6.03E-26	Down	MLXIP
CNTFR	94.8860738	-3.66020275	0.34139841	-10.7212062	8.09E-27	6.08E-26	Down	CNTFR
UTP20	1377.94696	0.94223004	0.08789431	10.720035	8.20E-27	6.15E-26	Up	UTP20

DUSP15	110.109406	2.49113606	0.232506	10.7142872	8.72E-27	6.55E-26	Up	DUSP15
TNS2	1208.60882	-1.19302003	0.11135003	-10.7141416	8.74E-27	6.55E-26	Down	TNS2
NPW	68.9831642	2.99390061	0.27944941	10.7135695	8.79E-27	6.59E-26	Up	NPW
CORO6	28.1073788	-1.76933691	0.1651687	-10.7123017	8.91E-27	6.68E-26	Down	CORO6
PTCD3	3133.10093	0.67721641	0.06323522	10.7094808	9.19E-27	6.88E-26	Up	PTCD3
IQGAP2	1649.27732	-1.64861136	0.15394082	-10.7093837	9.20E-27	6.89E-26	Down	IQGAP2
MARCKSL1	11855.1934	0.87781405	0.08196735	10.709313	9.20E-27	6.89E-26	Up	MARCKSL1
VIPR1	1452.2935	-1.53312557	0.14317578	-10.707995	9.34E-27	6.99E-26	Down	VIPR1
SPAG5	1292.74676	0.93735093	0.08754112	10.7075507	9.38E-27	7.02E-26	Up	SPAG5
EIF2AK3	815.998174	-0.73232578	0.06839802	-10.706827	9.45E-27	7.07E-26	Down	EIF2AK3
TM4SF19	9.17563803	2.86256322	0.26740004	10.7051713	9.63E-27	7.19E-26	Up	TM4SF19
SS18L1	862.160167	0.95850519	0.08957303	10.7008231	1.01E-26	7.54E-26	Up	SS18L1
ATRNL1	27.3773711	-2.9129435	0.27224492	-10.6997165	1.02E-26	7.62E-26	Down	ATRNL1
SYTL2	2166.52899	-1.32784284	0.12410496	-10.6993533	1.02E-26	7.65E-26	Down	SYTL2
KIAA0895	231.880628	1.37243238	0.12827478	10.6991598	1.03E-26	7.66E-26	Up	KIAA0895
KCNK3	42.6950904	-2.92413713	0.27332976	-10.6982027	1.04E-26	7.74E-26	Down	KCNK3
SH3RF1	1624.90002	-0.80940384	0.07565974	-10.6979461	1.04E-26	7.76E-26	Down	SH3RF1
APLP2	17514.7029	-0.68119207	0.06368696	-10.6959425	1.06E-26	7.92E-26	Down	APLP2
RNF14	1451.39526	-0.69805399	0.0652815	-10.692984	1.10E-26	8.18E-26	Down	RNF14
ABCB6	176.990357	1.22451381	0.1145629	10.6885723	1.15E-26	8.57E-26	Up	ABCB6
TUBGCP3	1397.66451	0.77502039	0.07251784	10.6873061	1.17E-26	8.69E-26	Up	TUBGCP3
TTPAL	956.710394	0.92933212	0.08696557	10.686208	1.18E-26	8.79E-26	Up	TTPAL
LIPH	2224.80384	-1.27937866	0.11974897	-10.6838385	1.21E-26	9.01E-26	Down	LIPH
MME	807.23548	2.72417959	0.25498467	10.6836995	1.21E-26	9.02E-26	Up	MME
PARPBP	534.740363	1.22428617	0.11462538	10.6807601	1.25E-26	9.31E-26	Up	PARPBP
PPP2CB	2540.33654	-0.83506745	0.07819301	-10.679567	1.27E-26	9.42E-26	Down	PPP2CB
SLC17A5	1325.21702	-0.89920615	0.08423155	-10.6754084	1.33E-26	9.85E-26	Down	SLC17A5
BEST2	185.694139	-3.59558667	0.33684627	-10.6742661	1.34E-26	9.96E-26	Down	BEST2
SNAPC4	849.471231	0.94145715	0.08819851	10.6742981	1.34E-26	9.96E-26	Up	SNAPC4
MFAP5	357.202988	-2.66711756	0.24987235	-10.6739203	1.35E-26	1.00E-25	Down	MFAP5
ANGPTL7	20.2374983	-4.20913868	0.39442496	-10.6715829	1.38E-26	1.02E-25	Down	ANGPTL7
FOXF1	622.897911	-1.45084611	0.13597663	-10.6698193	1.41E-26	1.04E-25	Down	FOXF1
ANKRD16	328.136623	0.89923762	0.08427937	10.6697247	1.41E-26	1.04E-25	Up	ANKRD16
CADPS	746.504937	2.22226395	0.20840141	10.6633823	1.51E-26	1.12E-25	Up	CADPS
DENND2A	330.749953	-1.36300719	0.12782711	-10.6628964	1.52E-26	1.12E-25	Down	DENND2A
TMEM40	8.48431003	3.9521668	0.37076691	10.6594377	1.58E-26	1.17E-25	Up	TMEM40
TIMM50	2800.99605	0.93270444	0.08750799	10.6585061	1.59E-26	1.18E-25	Up	TIMM50
BHMT2	33.1448538	-2.23505556	0.20969778	-10.6584608	1.59E-26	1.18E-25	Down	BHMT2
LY6D	30.3988272	4.53393161	0.42541274	10.6577242	1.60E-26	1.19E-25	Up	LY6D
COA1	1279.91508	0.81156114	0.07617439	10.6539893	1.67E-26	1.23E-25	Up	COA1
BHLHE40	6217.25124	1.18060096	0.1108161	10.6536956	1.68E-26	1.24E-25	Up	BHLHE40
NEO1	4342.61212	-1.07940399	0.10134794	-10.6504774	1.73E-26	1.28E-25	Down	NEO1
LMO2	204.984605	-1.23113252	0.11562208	-10.6479019	1.78E-26	1.32E-25	Down	LMO2
PES1	3794.62216	0.88608022	0.08322053	10.6473756	1.79E-26	1.32E-25	Up	PES1
LTB4R	270.776478	1.55862606	0.14641377	10.6453517	1.83E-26	1.35E-25	Up	LTB4R
ZNF671	62.181686	-1.51791979	0.14259554	-10.644932	1.84E-26	1.36E-25	Down	ZNF671
CILP2	66.0538462	2.69418027	0.25309609	10.644891	1.84E-26	1.36E-25	Up	CILP2
SLBP	2092.28585	0.72467232	0.06809095	10.6427107	1.89E-26	1.39E-25	Up	SLBP
PIGZ	1253.27746	-1.66510914	0.15649022	-10.6403396	1.93E-26	1.42E-25	Down	PIGZ
CAMK2D	1882.31344	-0.97331882	0.09148776	-10.6387874	1.97E-26	1.45E-25	Down	CAMK2D
MEP1A	4829.17914	-2.31854317	0.21797216	-10.6368773	2.01E-26	1.48E-25	Down	MEP1A
UBA2	4067.35605	0.77257807	0.07263526	10.6364056	2.02E-26	1.48E-25	Up	UBA2
ZBTB16	38.845255	-3.26380156	0.30686092	-10.6360937	2.02E-26	1.49E-25	Down	ZBTB16
ST3GAL3	170.903806	-0.99072687	0.09316579	-10.6340197	2.07E-26	1.52E-25	Down	ST3GAL3
MORN5	18.1060889	-4.90890474	0.46170215	-10.6321895	2.11E-26	1.55E-25	Down	MORN5
EFHC2	19.6805004	-2.42101572	0.22771299	-10.6318738	2.12E-26	1.55E-25	Down	EFHC2
NPY2R	6.03375375	-4.51802838	0.42495167	-10.6318639	2.12E-26	1.55E-25	Down	NPY2R
PTGR1	1113.50198	-1.06057424	0.09976461	-10.6307658	2.14E-26	1.57E-25	Down	PTGR1

MYH15	52.8647045	-1.74612878	0.16432946	-10.6257807	2.26E-26	1.66E-25	Down	MYH15
EXOC6B	651.645001	-0.7943681	0.07476418	-10.624982	2.28E-26	1.67E-25	Down	EXOC6B
NUDT1	819.85015	1.15130834	0.1083689	10.6239735	2.31E-26	1.69E-25	Up	NUDT1
PRR9	20.5895223	6.93432526	0.65271416	10.6238315	2.31E-26	1.69E-25	Up	PRR9
TMEM255B	330.10836	1.75885314	0.16556012	10.6236524	2.31E-26	1.69E-25	Up	TMEM255B
PLXNA3	2154.3482	1.21913331	0.1147719	10.6222281	2.35E-26	1.72E-25	Up	PLXNA3
LGI4	180.009002	-2.0620877	0.19414076	-10.6216114	2.36E-26	1.73E-25	Down	LGI4
UGT2B17	2419.85613	-4.07000898	0.38331066	-10.6180427	2.46E-26	1.79E-25	Down	UGT2B17
VGLL1	9.59367719	5.8894725	0.55474684	10.6165047	2.50E-26	1.82E-25	Up	VGLL1
MAP1A	499.584669	-1.77462935	0.16717087	-10.6156617	2.52E-26	1.84E-25	Down	MAP1A
OTOP3	8.79125361	-6.05046886	0.57009943	-10.6130063	2.59E-26	1.89E-25	Down	OTOP3
CD22	129.431621	-2.58940818	0.24404938	-10.6101814	2.67E-26	1.95E-25	Down	CD22
SLC25A14	281.62395	0.85599838	0.08068668	10.6089187	2.71E-26	1.97E-25	Up	SLC25A14
SLC25A22	1469.76177	1.12670553	0.10620711	10.6085692	2.72E-26	1.98E-25	Up	SLC25A22
STRADB	632.098712	-0.91397118	0.08616917	-10.6067079	2.77E-26	2.02E-25	Down	STRADB
DPCD	628.418364	1.06567258	0.10052522	10.6010474	2.95E-26	2.15E-25	Up	DPCD
STK36	735.181383	0.90702875	0.085582	10.5983594	3.03E-26	2.21E-25	Up	STK36
UBE2I	4424.15369	0.6329602	0.05972779	10.5974161	3.06E-26	2.23E-25	Up	UBE2I
RELT	343.914526	1.09228573	0.10307358	10.5971454	3.07E-26	2.23E-25	Up	RELT
CCNG2	1210.10569	-1.01253748	0.09559483	-10.5919688	3.25E-26	2.36E-25	Down	CCNG2
BCL10	1382.74454	-0.94610301	0.08932427	-10.5917805	3.25E-26	2.36E-25	Down	BCL10
ZNF781	13.8953963	-1.73384363	0.16369729	-10.5917676	3.25E-26	2.36E-25	Down	ZNF781
AGFG2	1531.15492	-1.04987982	0.09915909	-10.5878327	3.39E-26	2.46E-25	Down	AGFG2
TLR10	35.0046504	-2.38121439	0.22500311	-10.5830289	3.57E-26	2.59E-25	Down	TLR10
PPY	3.4759512	-4.41443379	0.41717936	-10.5816208	3.63E-26	2.63E-25	Down	PPY
BID	2068.71089	1.05750375	0.09994672	10.5806754	3.66E-26	2.66E-25	Up	BID
TMEM147	3380.77294	1.12334053	0.10617356	10.5802284	3.68E-26	2.67E-25	Up	TMEM147
B3GALT5	1451.85353	-2.64233755	0.24975554	-10.5796954	3.70E-26	2.68E-25	Down	B3GALT5
HSD17B7	448.11945	0.93917628	0.08878191	10.5784641	3.75E-26	2.72E-25	Up	HSD17B7
SLC35B3	1132.51259	-0.71570874	0.06768356	-10.5743369	3.92E-26	2.84E-25	Down	SLC35B3
ABCE1	3036.29221	0.92515312	0.08755397	10.5666614	4.25E-26	3.08E-25	Up	ABCE1
PPIA	17025.275	0.80992054	0.07666275	10.5647207	4.34E-26	3.14E-25	Up	PPIA
BEND4	8.89545668	-2.57334131	0.24365191	-10.5615478	4.49E-26	3.25E-25	Down	BEND4
YPEL5	2198.43635	-0.83993508	0.0795301	-10.5612224	4.51E-26	3.26E-25	Down	YPEL5
USPL1	955.360237	1.01901186	0.09649432	10.5603302	4.55E-26	3.29E-25	Up	USPL1
TRIM9	41.7099737	-2.31485562	0.21925822	-10.5576688	4.68E-26	3.38E-25	Down	TRIM9
SMYD3	232.936697	0.97360302	0.09222208	10.5571576	4.71E-26	3.40E-25	Up	SMYD3
RASGEF1C	6.93043548	-3.04368038	0.28831211	-10.556894	4.72E-26	3.41E-25	Down	RASGEF1C
CCR2	82.2346185	-2.13653871	0.20240239	-10.5558964	4.77E-26	3.44E-25	Down	CCR2
DTD1	1495.60208	1.07926064	0.10228282	10.5517298	4.99E-26	3.60E-25	Up	DTD1
BEND3	510.407178	0.79617785	0.07546516	10.5502708	5.07E-26	3.65E-25	Up	BEND3
SECISBP2L	1030.51635	-1.07145092	0.10156642	-10.5492633	5.12E-26	3.69E-25	Down	SECISBP2L
ADAMTS8	75.1066887	-2.11730052	0.20072133	-10.548458	5.16E-26	3.72E-25	Down	ADAMTS8
FAM83H	7038.84048	1.1035759	0.10462946	10.5474684	5.22E-26	3.76E-25	Up	FAM83H
CYP2S1	6794.15101	1.36063005	0.12901228	10.5465158	5.27E-26	3.79E-25	Up	CYP2S1
RASD2	378.896825	-1.97092158	0.18690334	-10.5451386	5.35E-26	3.85E-25	Down	RASD2
PDZK1	52.1659082	-2.33817045	0.22173152	-10.5450524	5.35E-26	3.85E-25	Down	PDZK1
POLR3A	1186.56294	0.60952982	0.05781012	10.5436531	5.43E-26	3.90E-25	Up	POLR3A
PROB1	169.069502	-1.05686845	0.100244	-10.5429593	5.47E-26	3.93E-25	Down	PROB1
MATN2	1544.81492	-1.8137397	0.17205534	-10.5416064	5.55E-26	3.99E-25	Down	MATN2
NDUFA4L2	698.552433	2.29861864	0.21808172	10.5401712	5.64E-26	4.05E-25	Up	NDUFA4L2
RELL1	149.554414	-1.49347494	0.14169904	-10.5397675	5.66E-26	4.06E-25	Down	RELL1
GALK1	1091.06612	1.40347211	0.1331636	10.5394572	5.68E-26	4.07E-25	Up	GALK1
GJA3	20.9352221	3.11733202	0.29587048	10.5361372	5.89E-26	4.22E-25	Up	GJA3
ERICH2	30.0332158	2.34891829	0.22296424	10.5349552	5.96E-26	4.27E-25	Up	ERICH2
FEV	13.288349	-3.87030833	0.3673993	-10.5343377	6.00E-26	4.30E-25	Down	FEV
ERP27	254.783914	2.71718365	0.25800524	10.5315055	6.18E-26	4.43E-25	Up	ERP27
BZW2	4875.51731	0.80651727	0.07658295	10.5312908	6.20E-26	4.43E-25	Up	BZW2

DOCK6	2951.34532	0.95207635	0.09040496	10.5312403	6.20E-26	4.44E-25	Up	DOCK6
CENPE	601.232305	1.30423086	0.12386876	10.5291349	6.34E-26	4.53E-25	Up	CENPE
NACA2	33.9685912	2.71882115	0.25825135	10.5278098	6.43E-26	4.60E-25	Up	NACA2
RCCD1	758.453426	0.92490569	0.0878946	10.5228949	6.78E-26	4.84E-25	Up	RCCD1
ISX	920.698812	-2.24443039	0.21333051	-10.5209069	6.92E-26	4.94E-25	Down	ISX
UQCRFS1	3127.07028	-0.8137292	0.0773742	-10.5168023	7.23E-26	5.16E-25	Down	UQCRFS1
SLC8A2	40.9574095	-2.89093647	0.27508352	-10.5093043	7.83E-26	5.58E-25	Down	SLC8A2
DNAH14	243.52014	1.92691432	0.18338129	10.5076932	7.96E-26	5.68E-25	Up	DNAH14
ASPG	91.3397511	-2.7077109	0.25772129	-10.5063532	8.08E-26	5.76E-25	Down	ASPG
APBB1	314.875463	-1.59596807	0.15194856	-10.5033444	8.34E-26	5.94E-25	Down	APBB1
EXOSC4	1372.05034	1.48086038	0.14099424	10.502985	8.37E-26	5.96E-25	Up	EXOSC4
C2CD4B	290.551266	2.56815188	0.24464569	10.4974334	8.88E-26	6.32E-25	Up	C2CD4B
FOXP4	5219.1516	1.06017029	0.10100702	10.4960062	9.01E-26	6.41E-25	Up	FOXP4
NR0B2	138.725097	2.53273912	0.24131968	10.4953693	9.07E-26	6.46E-25	Up	NR0B2
BMP6	77.49149	-1.89039973	0.18013436	-10.4943872	9.17E-26	6.52E-25	Down	BMP6
PROKR1	2.16364817	-2.83442403	0.27010356	-10.4938417	9.22E-26	6.56E-25	Down	PROKR1
NUP210	3717.37254	1.3447564	0.12818922	10.4904016	9.56E-26	6.80E-25	Up	NUP210
RBM24	52.4112234	-2.26844064	0.21628639	-10.4881338	9.79E-26	6.96E-25	Down	RBM24
TIMM8A	415.491616	0.9034193	0.08614767	10.4868682	9.93E-26	7.05E-25	Up	TIMM8A
CRHBP	6.76401637	-2.4133748	0.23014259	-10.4864327	9.97E-26	7.08E-25	Down	CRHBP
KIFC2	855.955836	1.65660513	0.15800449	10.4845446	1.02E-25	7.22E-25	Up	KIFC2
TRMT6	921.406643	0.99299315	0.09471498	10.4840135	1.02E-25	7.26E-25	Up	TRMT6
UGT2B15	231.167847	-3.02516982	0.28856297	-10.4835691	1.03E-25	7.29E-25	Down	UGT2B15
MT1E	1698.04125	-2.4187078	0.23073217	-10.4827504	1.04E-25	7.35E-25	Down	MT1E
PCNA	6223.83738	1.09384747	0.10435185	10.4823003	1.04E-25	7.38E-25	Up	PCNA
PLCG1	3110.05917	1.04841255	0.10006755	10.4770485	1.10E-25	7.80E-25	Up	PLCG1
OTUD7A	71.8428134	-1.55760588	0.14868405	-10.4759444	1.11E-25	7.89E-25	Down	OTUD7A
CENPW	730.758503	1.12579517	0.1074662	10.4758074	1.12E-25	7.90E-25	Up	CENPW
LRAT	10.4417151	-3.35425374	0.32026096	-10.4735019	1.14E-25	8.09E-25	Down	LRAT
CLIP4	164.997255	-2.02848175	0.19371333	-10.471565	1.17E-25	8.25E-25	Down	CLIP4
DHX33	1029.17851	0.72174995	0.06893013	10.4707469	1.18E-25	8.32E-25	Up	DHX33
BCL2L1	5214.97638	0.99530298	0.09505752	10.4705341	1.18E-25	8.33E-25	Up	BCL2L1
CACNA2D1	184.56631	-2.05768371	0.1965257	-10.4703033	1.18E-25	8.35E-25	Down	CACNA2D1
OPTN	3198.37904	-0.84401828	0.0806148	-10.4697677	1.19E-25	8.40E-25	Down	OPTN
FPGS	2578.74398	1.07227039	0.10242033	10.4693116	1.20E-25	8.43E-25	Up	FPGS
ADH1B	679.912895	-3.86793884	0.3694796	-10.4686126	1.20E-25	8.49E-25	Down	ADH1B
TEP1	1652.54689	-0.89832969	0.08582287	-10.4672525	1.22E-25	8.61E-25	Down	TEP1
ACSF2	1303.46186	-1.34958809	0.1289387	-10.4668967	1.23E-25	8.64E-25	Down	ACSF2
RIN1	925.756032	1.1705722	0.11183941	10.4665446	1.23E-25	8.67E-25	Up	RIN1
NSDHL	1542.46065	1.04975223	0.10030134	10.4659839	1.24E-25	8.72E-25	Up	NSDHL
SLCO5A1	139.289476	1.83161358	0.17504447	10.4637046	1.27E-25	8.93E-25	Up	SLCO5A1
GBA	1414.1238	-0.82291824	0.07867938	-10.4591342	1.33E-25	9.36E-25	Down	GBA
F12	763.487466	1.67389623	0.16009443	10.4556809	1.38E-25	9.71E-25	Up	F12
TLCD1	561.664542	1.40132626	0.13408509	10.451022	1.45E-25	1.02E-24	Up	TLCD1
EPHA1	1332.11881	1.2232436	0.11708416	10.4475586	1.50E-25	1.06E-24	Up	EPHA1
KLF13	3919.06233	-0.84387269	0.08077684	-10.4469633	1.51E-25	1.06E-24	Down	KLF13
CAT	3556.32657	-0.8607722	0.08239694	-10.4466519	1.52E-25	1.07E-24	Down	CAT
RASSF10	250.815697	2.3693787	0.22683051	10.4455911	1.53E-25	1.08E-24	Up	RASSF10
PLD1	1099.01367	-1.12084921	0.10730986	-10.4449784	1.54E-25	1.08E-24	Down	PLD1
ZNF316	1543.17377	1.048091	0.10036869	10.4424097	1.59E-25	1.11E-24	Up	ZNF316
FHL5	32.9644979	-1.72902268	0.16560813	-10.440446	1.62E-25	1.14E-24	Down	FHL5
TSC22D3	1739.59842	-1.32455133	0.12691327	-10.4366652	1.69E-25	1.18E-24	Down	TSC22D3
EMC8	1447.57448	0.82009299	0.07858478	10.4357742	1.70E-25	1.19E-24	Up	EMC8
NT5DC4	19.141589	2.49862867	0.23948872	10.4331791	1.75E-25	1.23E-24	Up	NT5DC4
NCS1	1207.0253	-1.33545558	0.12800766	-10.4326227	1.76E-25	1.23E-24	Down	NCS1
ZNF761	623.55407	0.93298752	0.08944297	10.4310878	1.79E-25	1.25E-24	Up	ZNF761
EHMT2	3220.07207	0.89738413	0.08604035	10.4298059	1.81E-25	1.27E-24	Up	EHMT2
SLC35C2	3062.53434	0.8234911	0.07899271	10.4248995	1.91E-25	1.33E-24	Up	SLC35C2

UGT2A3	711.891712	-3.2122546	0.30818021	-10.4232994	1.94E-25	1.36E-24	Down	UGT2A3
NXPH1	3.96504629	4.5832611	0.43974851	10.4224598	1.96E-25	1.37E-24	Up	NXPH1
AUH	564.417222	-0.70033659	0.06721034	-10.4200721	2.01E-25	1.40E-24	Down	AUH
WDR27	509.945247	1.24362415	0.1193531	10.4197054	2.02E-25	1.41E-24	Up	WDR27
APOBR	1934.74344	-1.73999255	0.16699818	-10.4192303	2.03E-25	1.41E-24	Down	APOBR
MTAP	1435.97511	0.91277606	0.08763749	10.4153608	2.11E-25	1.47E-24	Up	MTAP
PABPC3	86.4771298	2.03049664	0.19495889	10.4149988	2.12E-25	1.48E-24	Up	PABPC3
ATP13A4	45.1862166	-2.47509677	0.23767224	-10.4139077	2.14E-25	1.49E-24	Down	ATP13A4
IL1A	96.9112132	2.64760282	0.25424237	10.4136963	2.15E-25	1.50E-24	Up	IL1A
POLE2	284.798617	1.07251804	0.10299201	10.4136045	2.15E-25	1.50E-24	Up	POLE2
MEIS1	327.32706	-1.87938541	0.18048264	-10.413109	2.16E-25	1.50E-24	Down	MEIS1
IMPA1	992.067502	-1.09852861	0.10553751	-10.4088923	2.26E-25	1.57E-24	Down	IMPA1
CDK5	737.818991	0.89724573	0.08621167	10.4074737	2.29E-25	1.59E-24	Up	CDK5
KIAA1671	2973.42356	-0.88583379	0.08513125	-10.4055061	2.34E-25	1.63E-24	Down	KIAA1671
CLCN4	329.360658	1.74510196	0.1677218	10.4047413	2.36E-25	1.64E-24	Up	CLCN4
AGTR1	42.7595231	-3.49796696	0.33624711	-10.402965	2.40E-25	1.67E-24	Down	AGTR1
IMPDH2	9004.2044	0.88706467	0.08528734	10.4008949	2.46E-25	1.71E-24	Up	IMPDH2
TNNT2	39.2478674	2.40669494	0.23148836	10.3966131	2.57E-25	1.78E-24	Up	TNNT2
RFC2	1266.91706	0.80684057	0.07761502	10.3954177	2.60E-25	1.81E-24	Up	RFC2
SOCS2	372.322111	-1.19766796	0.11521536	-10.3950376	2.61E-25	1.81E-24	Down	SOCS2
STX16	2764.71718	1.00737386	0.0969308	10.3927123	2.68E-25	1.86E-24	Up	STX16
CLU	2938.43943	-2.24927489	0.21661695	-10.3836514	2.94E-25	2.04E-24	Down	CLU
CENPJ	590.253596	1.091602	0.10512833	10.3835186	2.95E-25	2.04E-24	Up	CENPJ
HBB	790.94325	-2.56661585	0.2471998	-10.3827586	2.97E-25	2.06E-24	Down	HBB
NTAN1	543.499851	-0.76551986	0.07374679	-10.380382	3.05E-25	2.11E-24	Down	NTAN1
BEST3	15.3643361	3.97075134	0.38254312	10.3798792	3.06E-25	2.12E-24	Up	BEST3
PSMG1	1685.44733	1.09698733	0.10573594	10.374782	3.23E-25	2.23E-24	Up	PSMG1
CTPS2	1501.01123	0.81690467	0.0787464	10.3738674	3.26E-25	2.26E-24	Up	CTPS2
PRMT1	7159.59984	1.28741263	0.12414239	10.3704511	3.38E-25	2.34E-24	Up	PRMT1
MOCS3	806.512229	1.05811053	0.10204244	10.3693181	3.42E-25	2.36E-24	Up	MOCS3
TNFRSF1A	4284.71088	-0.75450384	0.07277131	-10.3681503	3.46E-25	2.39E-24	Down	TNFRSF1A
NOC4L	1243.91211	1.07926907	0.10409681	10.3679356	3.47E-25	2.40E-24	Up	NOC4L
MST1	219.979668	1.79512934	0.17316196	10.3667649	3.51E-25	2.42E-24	Up	MST1
ST3GAL2	1302.33685	0.85711193	0.08269076	10.365268	3.57E-25	2.46E-24	Up	ST3GAL2
NPIPB5	173.248661	2.10984625	0.20356261	10.3646059	3.59E-25	2.48E-24	Up	NPIPB5
FBXO5	454.990358	0.90566504	0.0873839	10.3642093	3.61E-25	2.49E-24	Up	FBXO5
CDC16	2751.94753	0.79413993	0.07662583	10.3638674	3.62E-25	2.50E-24	Up	CDC16
CCNB2	1499.7202	0.99241641	0.09580023	10.3592279	3.80E-25	2.62E-24	Up	CCNB2
GPR89A	125.098195	0.72413603	0.06990402	10.3590039	3.81E-25	2.62E-24	Up	GPR89A
BACE2	6348.69171	1.20595201	0.11643689	10.3571303	3.88E-25	2.67E-24	Up	BACE2
INSL5	166.715613	-5.78731004	0.5587898	-10.3568641	3.90E-25	2.68E-24	Down	INSL5
EMD	2561.24238	0.9371856	0.09049788	10.3558843	3.94E-25	2.71E-24	Up	EMD
EXT1	1987.5814	-0.65210452	0.06299013	-10.3524869	4.08E-25	2.80E-24	Down	EXT1
LAPTM4B	6288.28025	1.50812832	0.14570176	10.3507899	4.15E-25	2.85E-24	Up	LAPTM4B
RBM39	7889.83991	0.85394256	0.08252167	10.3481001	4.27E-25	2.93E-24	Up	RBM39
PHKA1	621.931216	1.28054215	0.12375541	10.3473632	4.30E-25	2.95E-24	Up	PHKA1
SLC38A5	3054.00748	2.09560263	0.20253049	10.3470969	4.31E-25	2.96E-24	Up	SLC38A5
STK17B	1413.57605	-1.1034373	0.1066629	-10.3450902	4.41E-25	3.02E-24	Down	STK17B
MIOX	12.4904559	3.3436199	0.32324478	10.3439254	4.46E-25	3.06E-24	Up	MIOX
SYNC	73.1269099	-1.95192911	0.18872006	-10.342987	4.50E-25	3.09E-24	Down	SYNC
PTDSS1	4182.58841	0.8134408	0.07866151	10.3410265	4.60E-25	3.15E-24	Up	PTDSS1
FKBP5	1756.51974	-1.11853356	0.10817976	-10.339583	4.67E-25	3.20E-24	Down	FKBP5
ACLY	6943.7342	0.64961615	0.06286617	10.3333182	4.98E-25	3.41E-24	Up	ACLY
DLG2	35.5287166	-1.86723861	0.18071878	-10.3322886	5.03E-25	3.45E-24	Down	DLG2
PLK4	466.704016	0.93524243	0.09051787	10.3321309	5.04E-25	3.45E-24	Up	PLK4
TDP1	741.180459	0.63643478	0.06160551	10.3308088	5.11E-25	3.50E-24	Up	TDP1
CKS1B	1038.08871	0.99825281	0.09663719	10.329903	5.16E-25	3.53E-24	Up	CKS1B
POLB	684.803511	1.01852721	0.09860395	10.3294772	5.18E-25	3.54E-24	Up	POLB

GAS7	702.348958	-1.61111711	0.15597963	-10.3290228	5.21E-25	3.56E-24	Down	GAS7
SNRPA1	1250.52357	0.74649764	0.07228513	10.3271255	5.31E-25	3.63E-24	Up	SNRPA1
SNRPB	10388.2005	1.16238955	0.1125585	10.326981	5.32E-25	3.63E-24	Up	SNRPB
MYOM3	819.521154	2.0287898	0.19645649	10.3269164	5.32E-25	3.64E-24	Up	MYOM3
TSPAN11	381.283448	-1.62308947	0.15722781	-10.3231702	5.54E-25	3.78E-24	Down	TSPAN11
PALM	280.667961	-1.87359958	0.18152335	-10.3215351	5.63E-25	3.84E-24	Down	PALM
SLC25A15	501.681666	0.91589913	0.08875733	10.3191378	5.77E-25	3.94E-24	Up	SLC25A15
CD27	129.459558	-1.85992497	0.18025558	-10.3182655	5.83E-25	3.97E-24	Down	CD27
TBC1D10A	507.078516	-0.79616191	0.07716444	-10.3177312	5.86E-25	3.99E-24	Down	TBC1D10A
TEX30	306.814561	1.2434772	0.12052482	10.3171879	5.89E-25	4.01E-24	Up	TEX30
FMNL2	1445.35637	0.95214259	0.0923083	10.3148105	6.04E-25	4.11E-24	Up	FMNL2
ANTXR2	2402.91823	-1.24179308	0.12040419	-10.3135374	6.12E-25	4.17E-24	Down	ANTXR2
BTC	283.09303	-1.20832396	0.11717076	-10.3125039	6.19E-25	4.21E-24	Down	BTC
ZCCHC7	925.510733	0.76978542	0.07465083	10.3118128	6.23E-25	4.24E-24	Up	ZCCHC7
THEM6	2814.92616	1.33140864	0.12912349	10.3111266	6.28E-25	4.27E-24	Up	THEM6
FAM136A	2200.33318	0.58365856	0.05660736	10.3106473	6.31E-25	4.29E-24	Up	FAM136A
TRIM36	298.14147	-1.51744229	0.14717679	-10.310337	6.33E-25	4.30E-24	Down	TRIM36
KRT15	300.641667	1.66503145	0.16151729	10.3086887	6.44E-25	4.37E-24	Up	KRT15
AQP11	130.429016	-1.1247263	0.10912836	-10.3064525	6.59E-25	4.47E-24	Down	AQP11
CAMSAP1	1110.5221	0.63399598	0.0615167	10.3060792	6.61E-25	4.49E-24	Up	CAMSAP1
ZNF677	56.4395447	-1.68826861	0.16383948	-10.3044066	6.73E-25	4.57E-24	Down	ZNF677
ADAM33	139.147571	-2.11811836	0.20557071	-10.3036002	6.79E-25	4.60E-24	Down	ADAM33
ZFYVE28	456.567341	-1.201922	0.11665446	-10.3032663	6.81E-25	4.62E-24	Down	ZFYVE28
ARHGAP40	9.26268485	4.69708409	0.45593341	10.3021274	6.89E-25	4.67E-24	Up	ARHGAP40
KDM4A	1675.47958	-0.59872077	0.05812665	-10.3002796	7.03E-25	4.76E-24	Down	KDM4A
ANKLE2	2573.97186	0.55292045	0.05368731	10.2989039	7.13E-25	4.82E-24	Up	ANKLE2
GNAQ	2412.93651	-0.98001791	0.09515822	-10.298826	7.13E-25	4.83E-24	Down	GNAQ
SLC25A4	1264.9563	-0.94095446	0.09137851	-10.2973281	7.24E-25	4.90E-24	Down	SLC25A4
TNIP1	6058.81366	-0.69568515	0.06756188	-10.2970071	7.27E-25	4.92E-24	Down	TNIP1
GINS2	1056.61936	1.11073573	0.10789916	10.2942021	7.48E-25	5.06E-24	Up	GINS2
HAPLN3	544.330243	1.70765343	0.16594158	10.2906902	7.76E-25	5.24E-24	Up	HAPLN3
GPR180	787.642786	0.99362056	0.09657459	10.288634	7.93E-25	5.36E-24	Up	GPR180
NOS1	35.7306778	-3.38940234	0.3294702	-10.2874323	8.03E-25	5.42E-24	Down	NOS1
GAS8	556.919562	0.70611508	0.06864789	10.2860423	8.15E-25	5.50E-24	Up	GAS8
INTS8	1850.53707	0.80770013	0.07852399	10.2860297	8.15E-25	5.50E-24	Up	INTS8
PDE3A	916.144428	-1.98249639	0.19276357	-10.2846008	8.27E-25	5.58E-24	Down	PDE3A
CPQ	657.22312	-1.27340298	0.12382648	-10.2837697	8.34E-25	5.62E-24	Down	CPQ
C1orf198	2049.39548	0.72081313	0.07009842	10.2828732	8.42E-25	5.67E-24	Up	C1orf198
LAIR2	21.8876647	2.59975609	0.25283707	10.2823377	8.46E-25	5.70E-24	Up	LAIR2
GHR	140.912283	-2.26042008	0.21987018	-10.2807032	8.61E-25	5.80E-24	Down	GHR
GCOM1	32.5918434	-1.46133669	0.14214599	-10.280534	8.62E-25	5.81E-24	Down	GCOM1
LGALSL	750.41902	-0.86149468	0.08382003	-10.2779091	8.86E-25	5.96E-24	Down	LGALSL
RABEPK	1181.78815	0.89259956	0.08689956	10.2716234	9.46E-25	6.36E-24	Up	RABEPK
KCNN4	3482.27401	1.63455478	0.15918952	10.2679797	9.82E-25	6.61E-24	Up	KCNN4
WDR5B	303.625665	0.81803222	0.07967112	10.2676134	9.86E-25	6.63E-24	Up	WDR5B
TMA16	705.722971	0.74805426	0.07285813	10.2672727	9.90E-25	6.65E-24	Up	TMA16
SIGLEC6	11.7647292	-2.28295988	0.22236942	-10.2665189	9.97E-25	6.70E-24	Down	SIGLEC6
ATP6V0A4	7.97155639	4.6450968	0.45250841	10.2652166	1.01E-24	6.79E-24	Up	ATP6V0A4
LRRC43	26.1710984	1.99809042	0.19466204	10.2644068	1.02E-24	6.84E-24	Up	LRRC43
TMTC1	274.368825	-1.79435289	0.17483646	-10.2630358	1.03E-24	6.94E-24	Down	TMTC1
MCEMP1	48.6563805	2.88092833	0.28071888	10.2626812	1.04E-24	6.96E-24	Up	MCEMP1
HOXC11	51.3236007	5.03855866	0.49111625	10.2594013	1.07E-24	7.20E-24	Up	HOXC11
UTP15	598.358211	0.79685784	0.07767181	10.2592928	1.07E-24	7.20E-24	Up	UTP15
CACNA1D	577.422648	1.4101517	0.13746996	10.2578895	1.09E-24	7.31E-24	Up	CACNA1D
MCM8	802.276346	1.10339952	0.10758557	10.2560173	1.11E-24	7.45E-24	Up	MCM8
HSPB2	17.5656037	-1.69585683	0.16537532	-10.2545943	1.13E-24	7.55E-24	Down	HSPB2
DLGAP4	3714.40577	0.71779053	0.07001663	10.2517157	1.16E-24	7.78E-24	Up	DLGAP4
USP39	2301.87442	0.52463587	0.05118345	10.2501077	1.18E-24	7.91E-24	Up	USP39

IRX3	43.6246493	3.12591233	0.30496759	10.2499821	1.18E-24	7.91E-24	Up	IRX3
HSPB7	286.452944	-2.66353763	0.25988857	-10.2487679	1.20E-24	8.01E-24	Down	HSPB7
CEP250	1963.07406	1.03492522	0.10098395	10.2484128	1.20E-24	8.04E-24	Up	CEP250
TMEM158	451.024121	1.59535196	0.15570732	10.2458378	1.24E-24	8.25E-24	Up	TMEM158
ATAD3A	1629.51789	1.21036684	0.11817981	10.2417399	1.29E-24	8.61E-24	Up	ATAD3A
IGFBP6	406.416834	-1.93591645	0.18904567	-10.2404697	1.31E-24	8.72E-24	Down	IGFBP6
FBXL5	2011.52051	-0.8289498	0.08097246	-10.2374288	1.35E-24	8.99E-24	Down	FBXL5
APCDD1	3586.80776	2.51847413	0.24602397	10.2367023	1.36E-24	9.06E-24	Up	APCDD1
TDRD7	1117.9664	-0.70169601	0.06855173	-10.2360076	1.37E-24	9.12E-24	Down	TDRD7
TRIT1	752.490979	0.74071179	0.07238384	10.23311	1.41E-24	9.39E-24	Up	TRIT1
BAHD1	1237.38061	-0.7143669	0.06982352	-10.231036	1.44E-24	9.59E-24	Down	BAHD1
PRPS1	1279.98521	0.84401204	0.0824953	10.2310316	1.44E-24	9.59E-24	Up	PRPS1
CDC20	2719.3348	1.21186033	0.11850409	10.2263162	1.51E-24	1.01E-23	Up	CDC20
NOL8	1370.55721	0.79368433	0.07763147	10.2237445	1.55E-24	1.03E-23	Up	NOL8
CHMP3	3645.97329	-0.59991848	0.0586898	-10.2218534	1.58E-24	1.05E-23	Down	CHMP3
C9orf50	11.0498601	2.23857235	0.21902199	10.2207653	1.60E-24	1.06E-23	Up	C9orf50
MID1IP1	2620.62996	0.95646062	0.09361407	10.2170603	1.66E-24	1.11E-23	Up	MID1IP1
PRDM13	15.9539439	4.23038135	0.41410772	10.2156545	1.69E-24	1.12E-23	Up	PRDM13
NPHP4	553.226863	0.90477903	0.08856759	10.2156899	1.69E-24	1.12E-23	Up	NPHP4
GPER1	122.519583	-2.0739324	0.20307553	-10.2126158	1.74E-24	1.16E-23	Down	GPER1
PLS3	1742.51427	1.07021217	0.1047988	10.2120653	1.75E-24	1.16E-23	Up	PLS3
ORC5	858.579956	0.70933933	0.06949381	10.2072299	1.84E-24	1.22E-23	Up	ORC5
CD79A	388.761233	-2.47276316	0.2423102	-10.2049485	1.88E-24	1.25E-23	Down	CD79A
FMN2	34.3023095	-3.40407871	0.33365133	-10.202503	1.93E-24	1.28E-23	Down	FMN2
SYNJ2BP	1337.3604	-0.89239488	0.0874837	-10.2006992	1.97E-24	1.30E-23	Down	SYNJ2BP
SMG9	1293.33878	0.66627735	0.06531965	10.2002583	1.98E-24	1.31E-23	Up	SMG9
SIGLEC15	76.6468421	2.47360372	0.24252559	10.1993512	2.00E-24	1.32E-23	Up	SIGLEC15
KNOP1	838.75887	0.64388506	0.06313181	10.1990591	2.00E-24	1.32E-23	Up	KNOP1
UBQLN4	1966.03211	0.63333394	0.0620988	10.1988119	2.01E-24	1.33E-23	Up	UBQLN4
ATP6V1F	3207.80563	0.94778342	0.09293112	10.198773	2.01E-24	1.33E-23	Up	ATP6V1F
UPK2	14.8341636	4.10856358	0.40300522	10.1948149	2.09E-24	1.38E-23	Up	UPK2
TLL1	30.9978362	-2.36807926	0.23232982	-10.1927476	2.14E-24	1.41E-23	Down	TLL1
KCNH4	27.9835392	2.17334268	0.21324659	10.1916878	2.16E-24	1.43E-23	Up	KCNH4
RELN	70.4989472	-2.6389547	0.2589568	-10.1907141	2.18E-24	1.44E-23	Down	RELN
FCHO1	622.778601	1.56679053	0.15375802	10.1899758	2.20E-24	1.45E-23	Up	FCHO1
MAGEA11	65.2552747	6.53507624	0.64168684	10.1842143	2.33E-24	1.54E-23	Up	MAGEA11
HSD17B6	97.0157316	-1.56786285	0.15397516	-10.1825698	2.37E-24	1.56E-23	Down	HSD17B6
SVEP1	418.124774	-1.75166482	0.17205096	-10.1810815	2.41E-24	1.59E-23	Down	SVEP1
FAS	591.999488	-1.36037045	0.13362828	-10.1802589	2.43E-24	1.60E-23	Down	FAS
B3GNT8	886.621801	-1.4356333	0.14105033	-10.178163	2.48E-24	1.64E-23	Down	B3GNT8
MAGOHB	582.784522	0.95380301	0.09375225	10.1736546	2.60E-24	1.71E-23	Up	MAGOHB
COX19	820.652919	0.71574864	0.07036609	10.171783	2.65E-24	1.74E-23	Up	COX19
ATAD5	341.057087	1.22295178	0.12023544	10.1713083	2.66E-24	1.75E-23	Up	ATAD5
SLC29A1	3387.70016	1.04974887	0.10326158	10.165919	2.81E-24	1.85E-23	Up	SLC29A1
LAGE3	924.863679	1.26193784	0.12417711	10.162403	2.92E-24	1.92E-23	Up	LAGE3
CHST8	2.90329401	-2.47571866	0.24365085	-10.1609276	2.96E-24	1.95E-23	Down	CHST8
MAPT	78.5094801	-2.38602531	0.23484351	-10.1600651	2.99E-24	1.96E-23	Down	MAPT
LGALS9C	165.157789	-2.64781461	0.26062621	-10.1594335	3.01E-24	1.98E-23	Down	LGALS9C
LARP1	8695.08666	0.62710618	0.06181395	10.1450596	3.49E-24	2.29E-23	Up	LARP1
METAP1D	346.9109	0.79189782	0.07806607	10.1439432	3.53E-24	2.31E-23	Up	METAP1D
CCSAP	421.043838	1.01016259	0.09959781	10.1424177	3.58E-24	2.35E-23	Up	CCSAP
WNT11	874.340603	2.7572212	0.27186741	10.1417863	3.60E-24	2.36E-23	Up	WNT11
TSPAN5	822.600904	1.23870242	0.12216261	10.1397836	3.68E-24	2.41E-23	Up	TSPAN5
PRKACA	2111.41062	-0.57172036	0.05638685	-10.1392499	3.70E-24	2.42E-23	Down	PRKACA
RAD51AP1	564.338733	1.17270184	0.115673	10.1380777	3.74E-24	2.45E-23	Up	RAD51AP1
RAB6B	161.955385	-1.56220112	0.15411321	-10.1367111	3.80E-24	2.49E-23	Down	RAB6B
RNF182	56.2299302	3.5044829	0.34577839	10.1350546	3.86E-24	2.53E-23	Up	RNF182
VPREB3	29.328273	-2.42465543	0.23924292	-10.1347007	3.88E-24	2.54E-23	Down	VPREB3

FZD5	3092.46731	-0.86788769	0.08563723	-10.1344667	3.88E-24	2.54E-23	Down	FZD5
PSMG3	1400.6632	1.00198114	0.09886903	10.1344285	3.89E-24	2.54E-23	Up	PSMG3
ZHX1-C8orf7(	30.1294292	1.15140553	0.11362542	10.1333448	3.93E-24	2.57E-23	Up	ZHX1-C8orf76
COX7A1	164.346348	-1.56718412	0.15465829	-10.133205	3.94E-24	2.57E-23	Down	COX7A1
FABP1	19709.5048	-2.64336837	0.26088484	-10.1323189	3.97E-24	2.59E-23	Down	FABP1
PRR5-ARHG/	18.7092797	1.64978927	0.16282497	10.1322865	3.97E-24	2.59E-23	Up	PRR5-ARHGAP8
LUC7L	1229.49704	0.88482486	0.08733811	10.1310285	4.02E-24	2.63E-23	Up	LUC7L
RTN1	105.937175	-1.78445121	0.17616478	-10.1294434	4.09E-24	2.67E-23	Down	RTN1
HAUS7	117.637908	1.26025326	0.12443692	10.1276473	4.17E-24	2.72E-23	Up	HAUS7
CEP83	464.554376	0.92893085	0.09174068	10.1256154	4.25E-24	2.77E-23	Up	CEP83
RAB37	174.361265	-1.51754848	0.14990299	-10.1235374	4.34E-24	2.83E-23	Down	RAB37
HLCS	927.358926	0.62338207	0.06157846	10.1233782	4.35E-24	2.84E-23	Up	HLCS
ITGA11	1073.44291	1.90833751	0.18853406	10.1219774	4.41E-24	2.87E-23	Up	ITGA11
CACNG4	168.219184	3.53807352	0.34958898	10.1206667	4.47E-24	2.91E-23	Up	CACNG4
BMF	589.217826	-1.15029248	0.11366207	-10.1202842	4.49E-24	2.92E-23	Down	BMF
GPC2	60.3952044	1.45969892	0.14423813	10.1200624	4.50E-24	2.93E-23	Up	GPC2
ABCA12	74.1381806	3.59353414	0.35525627	10.1153291	4.72E-24	3.07E-23	Up	ABCA12
SYPL2	20.832296	-1.93803071	0.19167854	-10.1108384	4.95E-24	3.22E-23	Down	SYPL2
PAM16	356.572171	0.9021883	0.08923204	10.1105864	4.96E-24	3.22E-23	Up	PAM16
SOX15	26.4779014	-1.54757042	0.15306918	-10.1102681	4.97E-24	3.23E-23	Down	SOX15
B3GALT6	897.575868	0.97913069	0.09684583	10.1101999	4.98E-24	3.23E-23	Up	B3GALT6
BSG	28696.3617	-0.91675385	0.09069536	-10.1080565	5.09E-24	3.30E-23	Down	BSG
PAK1IP1	838.704104	0.79442948	0.07861415	10.1054255	5.23E-24	3.39E-23	Up	PAK1IP1
VILL	2516.37091	-1.40581841	0.13912327	-10.1048401	5.26E-24	3.41E-23	Down	VILL
FAM111B	742.391905	1.1191831	0.11075691	10.1048606	5.26E-24	3.41E-23	Up	FAM111B
XPO4	1717.3612	1.05000298	0.10393365	10.1026282	5.38E-24	3.49E-23	Up	XPO4
NWD2	15.5467521	-2.56733748	0.25420097	-10.0996369	5.54E-24	3.59E-23	Down	NWD2
ZNF792	291.508883	0.79829002	0.07904736	10.0988825	5.59E-24	3.62E-23	Up	ZNF792
ST8SIA1	63.3827514	-1.71534383	0.16986385	-10.0983455	5.62E-24	3.64E-23	Down	ST8SIA1
ARHGAP35	2997.66426	-0.74723996	0.07400099	-10.0977029	5.66E-24	3.66E-23	Down	ARHGAP35
WRAP73	1051.73779	0.78405492	0.07766833	10.094912	5.82E-24	3.76E-23	Up	WRAP73
TRIM28	15001.7334	0.99837294	0.09890017	10.0947545	5.83E-24	3.77E-23	Up	TRIM28
GAS2	83.700555	1.90244419	0.18848038	10.0935925	5.90E-24	3.81E-23	Up	GAS2
ORM2	12.2383516	3.21091629	0.31814243	10.0927006	5.95E-24	3.85E-23	Up	ORM2
ERVMER34-1	109.356121	2.52134096	0.24982146	10.0925714	5.96E-24	3.85E-23	Up	ERVMER34-1
HOXD1	22.3509258	-2.31917729	0.22979358	-10.0924373	5.97E-24	3.85E-23	Down	HOXD1
ASTN1	9.60428232	-3.50910098	0.34773396	-10.0913382	6.03E-24	3.90E-23	Down	ASTN1
CASZ1	841.290014	-0.92068265	0.09123893	-10.0908973	6.06E-24	3.91E-23	Down	CASZ1
DCN	7536.37725	-1.85298005	0.18363558	-10.0905287	6.08E-24	3.93E-23	Down	DCN
AMMECR1	926.814171	0.86632333	0.08588104	10.0874803	6.28E-24	4.05E-23	Up	AMMECR1
AKAP8L	1160.37096	0.65792591	0.0652231	10.0873143	6.29E-24	4.05E-23	Up	AKAP8L
NEFH	21.6183531	-1.77215196	0.17568801	-10.0869258	6.31E-24	4.07E-23	Down	NEFH
FOXK2	2168.28176	0.60136381	0.05961893	10.0867923	6.32E-24	4.07E-23	Up	FOXK2
BCS1L	1048.53563	0.77628154	0.07696224	10.0865246	6.34E-24	4.08E-23	Up	BCS1L
CDH10	1.72212539	-3.93377354	0.39004795	-10.0853589	6.41E-24	4.13E-23	Down	CDH10
ALYREF	3316.43998	0.89643307	0.08888584	10.0852183	6.42E-24	4.13E-23	Up	ALYREF
SLC52A2	4923.69291	1.2468389	0.12368061	10.081119	6.70E-24	4.31E-23	Up	SLC52A2
DHODH	573.312836	0.69032868	0.06849538	10.0784714	6.88E-24	4.42E-23	Up	DHODH
ZNF26	469.441248	0.97021303	0.09627146	10.077888	6.92E-24	4.45E-23	Up	ZNF26
MEIS2	201.582915	-2.00169246	0.19870109	-10.0738875	7.21E-24	4.63E-23	Down	MEIS2
DDR2	972.971153	-2.00325532	0.19887146	-10.073116	7.26E-24	4.67E-23	Down	DDR2
NBEA	121.808652	-2.34604522	0.23298542	-10.0694939	7.54E-24	4.84E-23	Down	NBEA
ZBTB7C	854.156642	-2.13521084	0.2120604	-10.0688805	7.58E-24	4.87E-23	Down	ZBTB7C
PPME1	2094.5987	0.53935818	0.05357742	10.0668937	7.74E-24	4.97E-23	Up	PPME1
INHBB	331.455276	2.36915288	0.23535672	10.0662217	7.79E-24	5.00E-23	Up	INHBB
SRSF5	4561.03909	-0.66052514	0.06564814	-10.0615964	8.17E-24	5.24E-23	Down	SRSF5
CALCA	42.9412258	3.85042661	0.38280315	10.0585031	8.43E-24	5.40E-23	Up	CALCA
PDE1A	124.201146	-1.67832188	0.16688414	-10.056809	8.57E-24	5.49E-23	Down	PDE1A

HIF3A	100.745121	-2.25338672	0.22410364	-10.0551097	8.72E-24	5.59E-23	Down	HIF3A
CDKL2	17.5347197	-1.92850803	0.19183596	-10.0529017	8.92E-24	5.71E-23	Down	CDKL2
IL6ST	2357.53926	-1.20036641	0.119407	-10.0527303	8.94E-24	5.72E-23	Down	IL6ST
NKX3-2	51.6002212	-1.93211431	0.19222684	-10.0512205	9.07E-24	5.81E-23	Down	NKX3-2
NPM1	20989.4874	0.94817975	0.0943534	10.0492373	9.26E-24	5.92E-23	Up	NPM1
CCDC74A	147.568949	1.22014707	0.12149117	10.0430927	9.85E-24	6.30E-23	Up	CCDC74A
AATF	2609.48089	0.70512089	0.07021976	10.0416307	1.00E-23	6.39E-23	Up	AATF
YARS2	829.330098	0.7512285	0.07482702	10.0395355	1.02E-23	6.53E-23	Up	YARS2
TCEAL5	3.50722792	-3.4662002	0.34556558	-10.0305135	1.12E-23	7.15E-23	Down	TCEAL5
MC1R	129.957812	1.51125456	0.15069734	10.0284087	1.14E-23	7.30E-23	Up	MC1R
CPXM1	518.893945	1.70677797	0.17019829	10.0281734	1.15E-23	7.32E-23	Up	CPXM1
NDST1	2214.64287	-0.80183415	0.07996157	-10.0277435	1.15E-23	7.34E-23	Down	NDST1
NAA15	2133.71505	0.79013504	0.07879929	10.0271843	1.16E-23	7.38E-23	Up	NAA15
FOSL2	5107.22297	-0.90029119	0.0898234	-10.0229026	1.21E-23	7.71E-23	Down	FOSL2
PMP2	6.94753501	-4.02282564	0.40140761	-10.021797	1.22E-23	7.79E-23	Down	PMP2
MLKL	918.596662	0.66912275	0.0668074	10.0156976	1.30E-23	8.29E-23	Up	MLKL
SEC23A	2464.78547	-0.82138445	0.08201441	-10.0151237	1.31E-23	8.33E-23	Down	SEC23A
RAD51D	568.981918	0.71556246	0.07145952	10.0135355	1.33E-23	8.46E-23	Up	RAD51D
PBX1	1585.93101	-1.3847566	0.13830602	-10.0122657	1.35E-23	8.57E-23	Down	PBX1
TNFRSF10C	98.1750337	1.64076903	0.16389901	10.0108536	1.37E-23	8.69E-23	Up	TNFRSF10C
GGT6	2606.79488	-1.74886023	0.17481997	-10.0037783	1.47E-23	9.33E-23	Down	GGT6
COL12A1	7826.77906	1.8228978	0.18223313	10.0031085	1.48E-23	9.39E-23	Up	COL12A1
LMBRD1	1754.294	-0.86818278	0.08680409	-10.0016343	1.50E-23	9.53E-23	Down	LMBRD1
CELF4	9.24602534	-2.57219562	0.25717964	-10.0015524	1.50E-23	9.53E-23	Down	CELF4
BOD1	1637.8259	0.81737121	0.08172548	10.0014249	1.50E-23	9.54E-23	Up	BOD1
KRT83	11.0584181	3.77745484	0.3776909	10.0014451	1.50E-23	9.54E-23	Up	KRT83
ANKRD53	11.0482811	-1.55252905	0.15524836	-10.0002928	1.52E-23	9.64E-23	Down	ANKRD53
COL5A2	6030.61812	1.72888623	0.17288794	10.0000397	1.52E-23	9.66E-23	Up	COL5A2
KIF5C	77.3250034	-2.06365774	0.20643813	-9.99649485	1.58E-23	1.00E-22	Down	KIF5C
THOC3	496.016754	0.8730921	0.0873478	9.99558252	1.59E-23	1.01E-22	Up	THOC3
UPF3B	680.227235	0.88442789	0.08848162	9.99561101	1.59E-23	1.01E-22	Up	UPF3B
SH3BGRL	2525.87493	-1.17248016	0.11732322	-9.9935899	1.63E-23	1.03E-22	Down	SH3BGRL
CLCN2	1461.03069	-1.25913992	0.12599949	-9.99321458	1.63E-23	1.03E-22	Down	CLCN2
CAPN12	215.227191	1.86095972	0.18631031	9.98849586	1.71E-23	1.08E-22	Up	CAPN12
BEX4	311.403741	-1.81696542	0.18191595	-9.98793882	1.72E-23	1.09E-22	Down	BEX4
GZMB	460.43902	2.2387284	0.22416419	9.98700281	1.74E-23	1.10E-22	Up	GZMB
NOL10	1370.0454	0.57850757	0.05793623	9.98524671	1.77E-23	1.12E-22	Up	NOL10
KIAA1522	6789.75645	-0.85599584	0.08573755	-9.98390773	1.79E-23	1.13E-22	Down	KIAA1522
CD180	118.524461	-1.68670058	0.16894901	-9.98348923	1.80E-23	1.14E-22	Down	CD180
GPR143	292.439295	2.32138967	0.23253184	9.98310462	1.81E-23	1.14E-22	Up	GPR143
SRPK1	5525.71082	0.86958751	0.08713381	9.97990932	1.87E-23	1.18E-22	Up	SRPK1
MAGI2	105.771896	-1.32346053	0.1326377	-9.97801159	1.90E-23	1.20E-22	Down	MAGI2
DDX60	1292.51489	-1.50622154	0.15097274	-9.97677792	1.93E-23	1.22E-22	Down	DDX60
PRSS51	29.0759985	3.08749704	0.30953006	9.97478909	1.97E-23	1.24E-22	Up	PRSS51
CAP1	13626.5831	-0.60009153	0.06016594	-9.97394035	1.98E-23	1.25E-22	Down	CAP1
CYTH1	1796.38784	-0.65159928	0.06534623	-9.9714893	2.03E-23	1.28E-22	Down	CYTH1
EBNA1BP2	2650.50444	0.76073214	0.07629446	9.97100109	2.04E-23	1.29E-22	Up	EBNA1BP2
LANCL3	82.3429904	-1.64762226	0.16525187	-9.9703696	2.05E-23	1.29E-22	Down	LANCL3
CHAF1A	1268.91352	0.8853001	0.08881097	9.96836383	2.10E-23	1.32E-22	Up	CHAF1A
PLAGL2	3453.15043	1.25255334	0.12568126	9.96611095	2.14E-23	1.35E-22	Up	PLAGL2
BORA	453.978546	0.96011483	0.09634095	9.96580154	2.15E-23	1.35E-22	Up	BORA
PEX19	1800.05737	-0.56677906	0.05687536	-9.96528264	2.16E-23	1.36E-22	Down	PEX19
RBBP8NL	580.716485	1.4051353	0.14101041	9.96476317	2.17E-23	1.37E-22	Up	RBBP8NL
DAP3	3790.53272	0.55813139	0.05601161	9.9645658	2.18E-23	1.37E-22	Up	DAP3
CSTL1	6.06533123	3.39340373	0.34057044	9.96388214	2.19E-23	1.38E-22	Up	CSTL1
ADAMTSL5	247.990488	1.3182379	0.13231853	9.96261005	2.22E-23	1.40E-22	Up	ADAMTSL5
LSP1	1173.65977	-1.4312117	0.14366061	-9.96245025	2.23E-23	1.40E-22	Down	LSP1
KCNC1	4.76875982	-2.07273792	0.20808016	-9.96124727	2.25E-23	1.41E-22	Down	KCNC1

CREBZF	1977.59845	0.7512106	0.07541388	9.96117114	2.25E-23	1.41E-22	Up	CREBZF
ZNF598	2507.29447	0.98808189	0.09919709	9.96079509	2.26E-23	1.42E-22	Up	ZNF598
LETM1	3016.76495	-0.85165689	0.08551184	-9.95952068	2.29E-23	1.44E-22	Down	LETM1
PSORS1C2	16.5153133	3.09393184	0.31068798	9.95832481	2.32E-23	1.45E-22	Up	PSORS1C2
CDHR2	2063.41051	-2.13099525	0.21400653	-9.95761786	2.34E-23	1.46E-22	Down	CDHR2
CLSPN	292.076928	1.17000767	0.11751045	9.95662668	2.36E-23	1.48E-22	Up	CLSPN
BTG1	3821.69663	-0.81051064	0.08141259	-9.95559343	2.38E-23	1.49E-22	Down	BTG1
TUB	74.1015666	-1.91231453	0.19210112	-9.95472861	2.40E-23	1.51E-22	Down	TUB
STON1	213.273689	-1.84762618	0.18564759	-9.95233081	2.46E-23	1.54E-22	Down	STON1
SNX2	1776.31319	-0.59405004	0.05969167	-9.95197575	2.47E-23	1.55E-22	Down	SNX2
ABL1	3960.50906	-0.61130095	0.06144577	-9.9486249	2.56E-23	1.60E-22	Down	ABL1
NQO2	1000.1521	0.92234707	0.09271253	9.94846226	2.56E-23	1.60E-22	Up	NQO2
MAPK6	1894.11995	-0.93244756	0.09378535	-9.94235787	2.72E-23	1.70E-22	Down	MAPK6
PPM1N	80.2123783	1.62899199	0.16387795	9.94027581	2.78E-23	1.74E-22	Up	PPM1N
EPHA10	594.279337	-1.26010368	0.12678201	-9.9391364	2.81E-23	1.76E-22	Down	EPHA10
LRRC46	24.9799824	1.38985937	0.13988011	9.93607551	2.90E-23	1.81E-22	Up	LRRC46
MTHFD1	3551.00199	0.67972925	0.06841605	9.93523094	2.92E-23	1.83E-22	Up	MTHFD1
GALM	1804.97726	-0.86615507	0.08718224	-9.93499451	2.93E-23	1.83E-22	Down	GALM
C17orf78	32.4707937	4.22725174	0.42549154	9.93498427	2.93E-23	1.83E-22	Up	C17orf78
WFDC10B	11.0100791	2.52942645	0.25459891	9.93494622	2.93E-23	1.83E-22	Up	WFDC10B
RPS2	58801.8282	1.16416481	0.11719665	9.93343073	2.98E-23	1.86E-22	Up	RPS2
TEK	221.361591	-1.35055963	0.13599176	-9.93118704	3.05E-23	1.90E-22	Down	TEK
PPP1R12C	2292.52317	-0.78021654	0.07856551	-9.9307764	3.06E-23	1.90E-22	Down	PPP1R12C
PRSS1	36.3919311	3.77589125	0.38022209	9.93075202	3.06E-23	1.90E-22	Up	PRSS1
SPHK1	474.006561	1.89296499	0.190639	9.92957905	3.10E-23	1.93E-22	Up	SPHK1
PPARGC1A	496.125015	-1.81204541	0.18249449	-9.92931599	3.10E-23	1.93E-22	Down	PPARGC1A
PDZRN3	607.572548	-1.52829438	0.15392793	-9.92863599	3.13E-23	1.94E-22	Down	PDZRN3
RAD51AP2	11.0762315	2.22094637	0.22369954	9.92825649	3.14E-23	1.95E-22	Up	RAD51AP2
DIS3	2125.20923	0.79273193	0.0798491	9.92787539	3.15E-23	1.96E-22	Up	DIS3
GOLGA6L2	6.0102522	5.31618795	0.53561529	9.92538502	3.23E-23	2.01E-22	Up	GOLGA6L2
TRHDE	80.6281674	-2.25017208	0.22675719	-9.92326669	3.30E-23	2.05E-22	Down	TRHDE
ANGEL1	1233.81684	0.7248529	0.07306754	9.92031278	3.40E-23	2.11E-22	Up	ANGEL1
THBS2	4832.95858	2.28245464	0.230134	9.91793757	3.48E-23	2.16E-22	Up	THBS2
B2M	84240.4942	-1.07428437	0.10832917	-9.91685243	3.52E-23	2.18E-22	Down	B2M
TMEM200B	96.3868817	-1.68050695	0.16951797	-9.91344455	3.64E-23	2.26E-22	Down	TMEM200B
CRK	2628.8444	-0.62401394	0.06295128	-9.91264841	3.67E-23	2.27E-22	Down	CRK
BHLHA9	7.00861893	5.30410183	0.53516126	9.91122166	3.72E-23	2.31E-22	Up	BHLHA9
RTN4R	317.228391	1.56077413	0.15751478	9.90874733	3.81E-23	2.36E-22	Up	RTN4R
RBMX2	821.502603	0.74675539	0.07537632	9.90702838	3.88E-23	2.40E-22	Up	RBMX2
TRPC7	4.53275992	-2.24636919	0.22676439	-9.90618156	3.91E-23	2.42E-22	Down	TRPC7
VSIG2	1326.73064	-2.81584736	0.28428022	-9.90518213	3.95E-23	2.45E-22	Down	VSIG2
P3H2	493.360642	-1.83126865	0.18489868	-9.90417359	3.99E-23	2.47E-22	Down	P3H2
FGF10	28.4002013	-2.2714244	0.22936098	-9.90327308	4.03E-23	2.49E-22	Down	FGF10
TRPM2	1032.98903	1.64265182	0.16588014	9.90264298	4.05E-23	2.51E-22	Up	TRPM2
HECTD3	2928.55493	-0.83966367	0.08479453	-9.90233252	4.07E-23	2.51E-22	Down	HECTD3
NEU4	463.67011	-2.13752783	0.21591364	-9.89992043	4.17E-23	2.57E-22	Down	NEU4
PMS1	509.724423	0.75625263	0.07640657	9.89774339	4.26E-23	2.63E-22	Up	PMS1
CDC123	2696.93749	0.58529109	0.05914343	9.89613097	4.33E-23	2.67E-22	Up	CDC123
TTC28	324.441468	-1.39288504	0.14075846	-9.89556921	4.35E-23	2.69E-22	Down	TTC28
PIGS	2261.29127	-0.74800027	0.07560684	-9.89328833	4.45E-23	2.75E-22	Down	PIGS
PGF	366.987685	1.46794595	0.1485365	9.88272906	4.95E-23	3.05E-22	Up	PGF
NOD2	235.001729	1.47229249	0.14900154	9.88105539	5.03E-23	3.10E-22	Up	NOD2
CCR10	26.4702277	-1.7998008	0.18216436	-9.88009288	5.08E-23	3.13E-22	Down	CCR10
CCNJL	365.776152	-1.5594299	0.15789654	-9.87627628	5.28E-23	3.25E-22	Down	CCNJL
GAR1	995.272493	0.75474046	0.0764424	9.87332224	5.43E-23	3.35E-22	Up	GAR1
ADRM1	5919.38424	1.0527625	0.10665007	9.87118398	5.55E-23	3.42E-22	Up	ADRM1
CORO2A	2375.10056	-0.96343334	0.09760993	-9.8702392	5.60E-23	3.45E-22	Down	CORO2A
CCDC85C	1368.32616	-0.86328975	0.08748377	-9.86799904	5.73E-23	3.52E-22	Down	CCDC85C

ILF2	6847.10851	0.63566274	0.06441771	9.86782591	5.74E-23	3.53E-22	Up	ILF2
EDNRB	364.579986	-1.36072374	0.13790932	-9.86680025	5.80E-23	3.56E-22	Down	EDNRB
POLR3C	1022.2323	0.58193781	0.0589863	9.86564275	5.87E-23	3.60E-22	Up	POLR3C
KLHDC1	40.580019	-1.32389635	0.13422265	-9.86343442	6.00E-23	3.68E-22	Down	KLHDC1
HTR4	36.2798366	-2.79976042	0.28385939	-9.86319453	6.01E-23	3.69E-22	Down	HTR4
NXPE4	1385.66636	-2.86452297	0.29043636	-9.86282482	6.03E-23	3.70E-22	Down	NXPE4
ZBTB39	503.17573	0.6534041	0.06627332	9.85923356	6.25E-23	3.84E-22	Up	ZBTB39
UBE2O	1621.74546	0.70279787	0.07129697	9.85733199	6.37E-23	3.91E-22	Up	UBE2O
AFP	11.7917837	4.0079955	0.40666409	9.85578917	6.47E-23	3.97E-22	Up	AFP
ITGB3	94.6205116	-1.58051469	0.16038549	-9.85447446	6.56E-23	4.02E-22	Down	ITGB3
OSBPL10	944.471286	0.70373517	0.07144135	9.85053006	6.82E-23	4.18E-22	Up	OSBPL10
WFDC1	395.344705	-1.47762703	0.15003827	-9.84833414	6.97E-23	4.27E-22	Down	WFDC1
USP30	688.525312	-0.57619847	0.05851222	-9.84748908	7.03E-23	4.30E-22	Down	USP30
NSUN2	3121.18445	0.52524302	0.05334273	9.84657133	7.09E-23	4.34E-22	Up	NSUN2
B4GALNT4	394.449823	2.77579204	0.28192932	9.84570178	7.15E-23	4.38E-22	Up	B4GALNT4
KIF21B	1194.39209	1.1856838	0.12043223	9.84523665	7.19E-23	4.40E-22	Up	KIF21B
MPI	1234.83532	-0.86359254	0.08771898	-9.84499082	7.20E-23	4.41E-22	Down	MPI
SSBP4	2035.15397	1.06427126	0.10810481	9.8448094	7.22E-23	4.41E-22	Up	SSBP4
SPATA24	83.2832566	-0.79553494	0.08083943	-9.84092744	7.50E-23	4.58E-22	Down	SPATA24
ALOXE3	9.51536334	2.92245641	0.29703756	9.83867649	7.67E-23	4.69E-22	Up	ALOXE3
PNMA1	823.255924	-1.20336363	0.12231197	-9.83847783	7.69E-23	4.69E-22	Down	PNMA1
WNT8B	7.86523063	2.49924343	0.25420419	9.83163739	8.23E-23	5.02E-22	Up	WNT8B
TMEM50A	3813.32289	-0.60111082	0.06114496	-9.83091289	8.29E-23	5.06E-22	Down	TMEM50A
ADO	1013.347	0.50588909	0.05146267	9.83021404	8.34E-23	5.09E-22	Up	ADO
IL11RA	228.246598	-1.13349929	0.11531676	-9.82944127	8.41E-23	5.13E-22	Down	IL11RA
GALR1	3.50242235	-3.64328186	0.37072937	-9.82733529	8.59E-23	5.23E-22	Down	GALR1
ZNF3	1577.45102	0.62801147	0.06390879	9.82668373	8.64E-23	5.27E-22	Up	ZNF3
DRD2	163.92706	3.28255736	0.33406752	9.8260297	8.70E-23	5.30E-22	Up	DRD2
SLC4A10	34.0372135	-3.02587414	0.30802021	-9.82362209	8.91E-23	5.43E-22	Down	SLC4A10
SMPD3	1457.83736	-1.31093816	0.13345479	-9.82308824	8.96E-23	5.45E-22	Down	SMPD3
GNG4	1527.35196	2.69364219	0.27431981	9.81934979	9.29E-23	5.66E-22	Up	GNG4
TMEM251	377.387927	-0.72818368	0.07416341	-9.81863756	9.36E-23	5.70E-22	Down	TMEM251
RTN4RL2	51.6296701	1.76626129	0.18000072	9.8125237	9.94E-23	6.05E-22	Up	RTN4RL2
CNIH3	112.7957	1.32840392	0.13542304	9.80929034	1.03E-22	6.24E-22	Up	CNIH3
PRELP	784.749823	-2.55008898	0.25997399	-9.80901581	1.03E-22	6.26E-22	Down	PRELP
RALB	2886.39509	-0.57220023	0.05835123	-9.80613878	1.06E-22	6.44E-22	Down	RALB
B9D1	347.434735	1.20336021	0.12274055	9.80409687	1.08E-22	6.57E-22	Up	B9D1
PITPNM3	561.786167	-1.4368162	0.14663726	-9.79843882	1.14E-22	6.94E-22	Down	PITPNM3
B3GNT5	1132.604	-1.00132229	0.10219205	-9.79843665	1.14E-22	6.94E-22	Down	B3GNT5
AKAP4	5.71535452	4.79733118	0.48961142	9.79824205	1.15E-22	6.95E-22	Up	AKAP4
USP6NL	1465.58153	0.73600849	0.07512355	9.79730711	1.16E-22	7.01E-22	Up	USP6NL
ADCY6	2605.86491	-0.81536365	0.08323466	-9.79596326	1.17E-22	7.11E-22	Down	ADCY6
SKA1	356.228865	1.11352153	0.11368154	9.79509505	1.18E-22	7.16E-22	Up	SKA1
SLC6A9	695.918586	-1.330072	0.13581116	-9.79353994	1.20E-22	7.27E-22	Down	SLC6A9
NUDT16	1757.70191	-0.68914668	0.07037854	-9.79200071	1.22E-22	7.38E-22	Down	NUDT16
PIK3CG	120.633282	-1.79276026	0.18311396	-9.79040729	1.24E-22	7.49E-22	Down	PIK3CG
DCSTAMP	14.6308483	3.02239942	0.30871254	9.79033573	1.24E-22	7.50E-22	Up	DCSTAMP
HMMR	987.121863	1.2220555	0.12482577	9.79008998	1.24E-22	7.51E-22	Up	HMMR
ZGRF1	196.73922	1.1439468	0.11685182	9.78972153	1.25E-22	7.54E-22	Up	ZGRF1
CLDN11	63.0215979	-2.08055355	0.21255007	-9.78853384	1.26E-22	7.62E-22	Down	CLDN11
CAST	9204.18761	-0.84337389	0.08619027	-9.78502475	1.31E-22	7.89E-22	Down	CAST
NCK1	887.548717	-0.67135605	0.06861203	-9.78481563	1.31E-22	7.90E-22	Down	NCK1
METTL3	1039.18302	0.64279195	0.0656926	9.78484506	1.31E-22	7.90E-22	Up	METTL3
NBR1	5777.2013	-0.63802011	0.06520817	-9.78435855	1.31E-22	7.93E-22	Down	NBR1
PTPRF	15341.7635	-0.93690575	0.09576297	-9.78359148	1.32E-22	7.99E-22	Down	PTPRF
RPL12	33511.0923	0.90624526	0.09262924	9.78357676	1.32E-22	7.99E-22	Up	RPL12
WDR37	633.811216	-0.60234601	0.06156813	-9.7834057	1.33E-22	8.00E-22	Down	WDR37
MTMR10	765.190654	-0.82247469	0.08407788	-9.78229574	1.34E-22	8.09E-22	Down	MTMR10

FAM124A	77.3615212	-1.75057273	0.17896279	-9.78176927	1.35E-22	8.13E-22	Down	FAM124A
WDHD1	532.351053	0.95240396	0.09736866	9.78142222	1.35E-22	8.15E-22	Up	WDHD1
UBAP2	2365.49652	0.67930243	0.06945737	9.780134	1.37E-22	8.25E-22	Up	UBAP2
GSTP1	22273.5945	1.24757522	0.12756318	9.78005774	1.37E-22	8.26E-22	Up	GSTP1
PDE4D	638.211734	-1.38130034	0.141245	-9.77946335	1.38E-22	8.30E-22	Down	PDE4D
PDE1C	66.8091265	-1.51601932	0.15506971	-9.77637319	1.42E-22	8.56E-22	Down	PDE1C
SLC9A7	442.482631	1.59969597	0.16364176	9.77559749	1.43E-22	8.62E-22	Up	SLC9A7
WFDC10A	4.0214577	3.84008195	0.39300564	9.77106055	1.50E-22	9.01E-22	Up	WFDC10A
TLR7	76.1932836	-1.81314181	0.18556886	-9.77072208	1.50E-22	9.04E-22	Down	TLR7
SNRNP70	6033.32689	0.77577201	0.07945029	9.76424366	1.60E-22	9.63E-22	Up	SNRNP70
PMP22	1740.19705	-1.38332978	0.14168531	-9.76339566	1.62E-22	9.71E-22	Down	PMP22
THADA	1337.89326	0.58439095	0.05985545	9.7633714	1.62E-22	9.71E-22	Up	THADA
FUS	9791.58589	0.634954	0.06503777	9.76285044	1.63E-22	9.76E-22	Up	FUS
LRFN5	14.6976062	-2.1740756	0.2227332	-9.76089604	1.66E-22	9.94E-22	Down	LRFN5
SOX12	898.580601	1.23858574	0.12689896	9.76040918	1.66E-22	9.99E-22	Up	SOX12
SMPD4	2608.21476	0.59942446	0.06143168	9.75757946	1.71E-22	1.03E-21	Up	SMPD4
NDUFS1	4301.82625	-0.56062374	0.05747163	-9.75479111	1.76E-22	1.05E-21	Down	NDUFS1
ENOX2	705.037064	0.79170423	0.08116096	9.75474247	1.76E-22	1.05E-21	Up	ENOX2
MSANTD3	508.750495	0.71836571	0.07364482	9.75446315	1.77E-22	1.06E-21	Up	MSANTD3
CFD	1117.64103	-2.29930911	0.235743	-9.75345643	1.78E-22	1.07E-21	Down	CFD
MAOB	373.509594	-2.35846581	0.24182372	-9.75283087	1.79E-22	1.07E-21	Down	MAOB
GTF3C3	1366.82758	0.6013043	0.06167031	9.75030468	1.84E-22	1.10E-21	Up	GTF3C3
TOMM40L	558.351206	-0.78894776	0.080918	-9.74996622	1.85E-22	1.10E-21	Down	TOMM40L
GPR162	54.8505295	-1.26961883	0.13022852	-9.74916132	1.86E-22	1.11E-21	Down	GPR162
TCEAL2	32.0074547	-3.90479265	0.40055183	-9.74853292	1.87E-22	1.12E-21	Down	TCEAL2
BTK	141.348211	-1.5801914	0.16215245	-9.74509706	1.94E-22	1.16E-21	Down	BTK
RANBP17	193.268541	1.51531277	0.15559634	9.73874325	2.06E-22	1.23E-21	Up	RANBP17
CCDC138	235.735515	0.91768833	0.09423285	9.7385182	2.07E-22	1.23E-21	Up	CCDC138
PHLDA2	1697.57012	1.47044657	0.15106953	9.73357459	2.17E-22	1.29E-21	Up	PHLDA2
ZNF696	331.500442	0.93802648	0.09637272	9.73331905	2.17E-22	1.30E-21	Up	ZNF696
ZNF684	79.0193688	-0.77883209	0.08004478	-9.72995465	2.25E-22	1.34E-21	Down	ZNF684
BRIP1	357.406029	1.07110863	0.11014397	9.72462323	2.37E-22	1.41E-21	Up	BRIP1
RNF224	16.5791353	2.23858878	0.23022809	9.72335213	2.40E-22	1.43E-21	Up	RNF224
FRMD5	250.119951	1.69656265	0.1744934	9.72278965	2.41E-22	1.44E-21	Up	FRMD5
CCR8	14.4696728	2.07995163	0.21396238	9.72110899	2.45E-22	1.46E-21	Up	CCR8
BMX	73.2589563	-2.0684553	0.21278857	-9.72070682	2.46E-22	1.47E-21	Down	BMX
SYNGR1	139.789252	-1.97835394	0.20352172	-9.72060341	2.46E-22	1.47E-21	Down	SYNGR1
YY2	35.0977841	1.19813857	0.12329535	9.71763002	2.54E-22	1.51E-21	Up	YY2
SMAD7	851.671511	-0.92547198	0.09527423	-9.71377016	2.63E-22	1.57E-21	Down	SMAD7
WDR54	393.912323	1.17108591	0.12057618	9.71241525	2.67E-22	1.59E-21	Up	WDR54
NMT2	548.185644	-0.71241368	0.07335146	-9.71233097	2.67E-22	1.59E-21	Down	NMT2
WDR59	1419.89156	0.53006944	0.054588	9.7103662	2.72E-22	1.62E-21	Up	WDR59
GID8	4124.61171	0.68649525	0.0706981	9.71023687	2.73E-22	1.62E-21	Up	GID8
CD19	59.6655566	-2.09976341	0.21625561	-9.70963674	2.74E-22	1.63E-21	Down	CD19
RHBDL2	357.419836	-1.7808456	0.18341885	-9.70917449	2.76E-22	1.64E-21	Down	RHBDL2
SLC30A4	322.966309	-1.35471113	0.13954802	-9.70784903	2.79E-22	1.66E-21	Down	SLC30A4
TARS2	1288.69775	0.61923771	0.06381684	9.70335939	2.92E-22	1.73E-21	Up	TARS2
GRHL2	1971.00057	0.65102871	0.0670988	9.70253814	2.94E-22	1.74E-21	Up	GRHL2
SGCA	72.6236805	-2.36873849	0.24416874	-9.70123566	2.98E-22	1.77E-21	Down	SGCA
MSH6	1806.83033	0.64065831	0.06605065	9.69949985	3.03E-22	1.80E-21	Up	MSH6
GALNT15	107.686174	-2.04118831	0.21044911	-9.69920165	3.04E-22	1.80E-21	Down	GALNT15
TNPO2	3053.52564	0.66368331	0.06842831	9.69895847	3.05E-22	1.80E-21	Up	TNPO2
RNF125	342.689277	-1.47815925	0.15241205	-9.69844097	3.06E-22	1.81E-21	Down	RNF125
CPAMD8	51.6945845	-1.44365985	0.14886605	-9.69771031	3.08E-22	1.83E-21	Down	CPAMD8
ELAC1	184.176576	-0.78880866	0.08135051	-9.6964196	3.12E-22	1.85E-21	Down	ELAC1
ZNF703	8521.04748	1.07507792	0.11087978	9.69588767	3.14E-22	1.86E-21	Up	ZNF703
EPHA5	3.88533366	-2.82897202	0.29180073	-9.6948764	3.17E-22	1.88E-21	Down	EPHA5
CXCL11	523.091326	2.56234254	0.26434286	9.69325431	3.22E-22	1.90E-21	Up	CXCL11

VIPR2	28.3400601	-1.99564425	0.20589454	-9.69255539	3.24E-22	1.92E-21	Down	VIPR2
UNC79	14.0752019	-1.29617455	0.13374646	-9.69128118	3.28E-22	1.94E-21	Down	UNC79
WNT5A	877.388961	1.48976576	0.15380643	9.68597847	3.46E-22	2.04E-21	Up	WNT5A
RGS10	582.767938	-1.0084751	0.10412085	-9.68562099	3.47E-22	2.05E-21	Down	RGS10
KLRF1	6.14120528	-2.04614237	0.21126348	-9.68526319	3.48E-22	2.06E-21	Down	KLRF1
ZCWPW2	15.5552806	-1.171426	0.12095107	-9.68512275	3.49E-22	2.06E-21	Down	ZCWPW2
GABARAP	1078.62943	-1.20765577	0.12469944	-9.68453238	3.51E-22	2.07E-21	Down	GABARAP
TCEA3	2124.45437	-1.40531855	0.14513436	-9.68287942	3.57E-22	2.10E-21	Down	TCEA3
GNL2	2006.04785	0.60958593	0.06297807	9.67933717	3.69E-22	2.18E-21	Up	GNL2
SH2D2A	278.542392	1.07949281	0.1115339	9.6786076	3.72E-22	2.19E-21	Up	SH2D2A
LIG3	1216.85393	0.6263579	0.06473803	9.67527024	3.84E-22	2.26E-21	Up	LIG3
RBM26	2138.361	0.86141195	0.08904973	9.67338049	3.91E-22	2.30E-21	Up	RBM26
SEMA3E	30.6151993	-3.66727818	0.37913928	-9.67264117	3.94E-22	2.32E-21	Down	SEMA3E
LUZP1	1857.48139	-0.72874405	0.07534702	-9.67183611	3.97E-22	2.34E-21	Down	LUZP1
FXYD3	19858.9735	-1.47964962	0.15299449	-9.67126072	3.99E-22	2.35E-21	Down	FXYD3
POC5	340.522555	0.65950771	0.06819396	9.67105789	4.00E-22	2.35E-21	Up	POC5
SGK2	948.780537	-1.60155847	0.16561517	-9.67036094	4.03E-22	2.37E-21	Down	SGK2
MYPN	17.2368657	-2.42905164	0.25125808	-9.66755627	4.14E-22	2.43E-21	Down	MYPN
VIT	20.2507671	-2.56378601	0.2652524	-9.66545831	4.23E-22	2.48E-21	Down	VIT
PATZ1	1667.46713	0.76539359	0.07919022	9.66525361	4.24E-22	2.49E-21	Up	PATZ1
BTBD9	836.59037	-0.76445397	0.07909838	-9.66459672	4.26E-22	2.50E-21	Down	BTBD9
CAMKMT	367.230543	0.79990541	0.08278946	9.66192372	4.38E-22	2.57E-21	Up	CAMKMT
FGFR4	4263.56025	1.4265913	0.14765442	9.6616907	4.39E-22	2.57E-21	Up	FGFR4
SMARCD1	2434.29708	0.52694425	0.0545569	9.6586182	4.52E-22	2.65E-21	Up	SMARCD1
ACSS2	5650.13365	-0.93958848	0.09729176	-9.65743121	4.57E-22	2.68E-21	Down	ACSS2
PTCHD1	37.5899396	-3.09492695	0.32047032	-9.65745282	4.57E-22	2.68E-21	Down	PTCHD1
NOTCH3	2749.52346	1.37795486	0.14271394	9.65536254	4.67E-22	2.73E-21	Up	NOTCH3
HMGCL	1811.7325	-0.82175179	0.08511688	-9.65439286	4.71E-22	2.76E-21	Down	HMGCL
HELZ2	3451.91432	1.15865364	0.12004193	9.65207463	4.82E-22	2.82E-21	Up	HELZ2
TELO2	2057.07881	1.00134328	0.10374898	9.65159631	4.84E-22	2.83E-21	Up	TELO2
SMAD1	596.758415	-0.63518211	0.06585612	-9.64499692	5.16E-22	3.02E-21	Down	SMAD1
SCGB2A1	30.7225441	-2.74702726	0.28484886	-9.64380628	5.22E-22	3.05E-21	Down	SCGB2A1
LPCAT1	2223.19001	1.12079481	0.11623227	9.64271601	5.28E-22	3.09E-21	Up	LPCAT1
CD1C	45.3046223	-1.78545973	0.18521552	-9.6399034	5.42E-22	3.17E-21	Down	CD1C
MRPL36	852.227143	0.83807551	0.08696742	9.63666088	5.60E-22	3.27E-21	Up	MRPL36
ATP2A3	5060.14507	-1.59705497	0.16573282	-9.63632272	5.62E-22	3.28E-21	Down	ATP2A3
KCNJ11	148.062568	1.45472918	0.1509641	9.63625886	5.62E-22	3.28E-21	Up	KCNJ11
CNR2	13.2933095	-2.34480566	0.24341031	-9.63314015	5.79E-22	3.38E-21	Down	CNR2
DUSP2	517.893099	1.39878329	0.14520922	9.63288211	5.81E-22	3.39E-21	Up	DUSP2
PHF20	1903.29546	0.7073481	0.07344157	9.63144013	5.89E-22	3.44E-21	Up	PHF20
TOPBP1	1701.86078	0.65097678	0.06760646	9.62891355	6.04E-22	3.52E-21	Up	TOPBP1
HADH	3991.31726	-0.88099145	0.09151004	-9.62726513	6.13E-22	3.58E-21	Down	HADH
SNRPA	2859.20158	0.74318211	0.07719821	9.62693504	6.15E-22	3.59E-21	Up	SNRPA
C1orf35	703.963746	0.86385327	0.08973455	9.62676338	6.16E-22	3.59E-21	Up	C1orf35
ZNF750	30.9813149	3.15417241	0.32772914	9.62432696	6.31E-22	3.68E-21	Up	ZNF750
TMEM67	251.763915	1.1048734	0.11480699	9.62374671	6.35E-22	3.69E-21	Up	TMEM67
POU2F2	173.845926	-1.50827687	0.15673615	-9.62303123	6.39E-22	3.72E-21	Down	POU2F2
PDIA4	14031.451	0.73824696	0.0767182	9.62284056	6.40E-22	3.72E-21	Up	PDIA4
SSTR3	6.97664439	-2.23266425	0.23203989	-9.62189854	6.46E-22	3.76E-21	Down	SSTR3
NAT16	8.5243963	3.3221122	0.34547024	9.61620376	6.83E-22	3.97E-21	Up	NAT16
FAM8A1	1546.36702	-0.83061784	0.08640885	-9.61264782	7.07E-22	4.11E-21	Down	FAM8A1
KCNK9	30.4657081	2.73141998	0.28418651	9.61136397	7.16E-22	4.16E-21	Up	KCNK9
MICAL2	5170.97521	-0.75837179	0.07891036	-9.61054737	7.22E-22	4.19E-21	Down	MICAL2
PRPF19	6357.96912	0.67078179	0.06979976	9.61008722	7.25E-22	4.21E-21	Up	PRPF19
CNTN3	64.9266492	-2.56952345	0.2674034	-9.6091651	7.31E-22	4.24E-21	Down	CNTN3
SELENBP1	13251.1523	-1.90046773	0.19786434	-9.60490284	7.62E-22	4.42E-21	Down	SELENBP1
SBSN	7.15205466	3.42564598	0.35678178	9.60151606	7.88E-22	4.57E-21	Up	SBSN
ELFN1	91.0912545	1.42771417	0.148737	9.59891742	8.08E-22	4.68E-21	Up	ELFN1

CILP	255.017559	-2.85050017	0.29710875	-9.59413058	8.46E-22	4.91E-21	Down	CILP
MPPED2	20.0425161	-2.01669175	0.21022004	-9.59324217	8.54E-22	4.95E-21	Down	MPPED2
NAA16	571.731078	0.9381545	0.09780861	9.59173758	8.66E-22	5.02E-21	Up	NAA16
OSM	208.135531	2.27474617	0.2371592	9.59164219	8.67E-22	5.02E-21	Up	OSM
MIPEP	1568.97545	0.8272854	0.08625536	9.59111897	8.71E-22	5.04E-21	Up	MIPEP
CLIC2	202.64215	-1.44872266	0.15107888	-9.58918073	8.88E-22	5.14E-21	Down	CLIC2
ARHGAP42	384.831658	-1.10793523	0.11554725	-9.58859012	8.93E-22	5.17E-21	Down	ARHGAP42
TAF4	1421.09833	0.83788182	0.08738681	9.588196	8.96E-22	5.18E-21	Up	TAF4
FAM117A	365.23285	-0.90168429	0.09405245	-9.58703658	9.07E-22	5.24E-21	Down	FAM117A
MAGI1	988.709331	-0.78718507	0.08211583	-9.58627693	9.13E-22	5.28E-21	Down	MAGI1
TTC27	768.042584	0.60545247	0.06316893	9.58465588	9.28E-22	5.36E-21	Up	TTC27
WRNIP1	2418.40345	0.59904174	0.06250721	9.58356261	9.38E-22	5.41E-21	Up	WRNIP1
TRPV4	88.1537604	1.7023342	0.1776559	9.58219927	9.50E-22	5.48E-21	Up	TRPV4
PCDH10	8.76654018	-2.96975672	0.30996752	-9.58086407	9.62E-22	5.55E-21	Down	PCDH10
DDX18	3894.02685	0.62904426	0.06567562	9.57804809	9.89E-22	5.71E-21	Up	DDX18
ZNF511	1014.27737	0.97763194	0.10207851	9.57725479	9.97E-22	5.75E-21	Up	ZNF511
ZDHHC15	18.2602576	-1.95000873	0.20362669	-9.57639057	1.00E-21	5.79E-21	Down	ZDHHC15
TPRG1L	3296.3756	-0.73435127	0.07669578	-9.57485935	1.02E-21	5.88E-21	Down	TPRG1L
NOC2L	4136.90293	0.82699375	0.08638194	9.57368763	1.03E-21	5.94E-21	Up	NOC2L
PLEKHA8	706.927472	0.83044156	0.0867469	9.57315599	1.04E-21	5.97E-21	Up	PLEKHA8
PABPC4	5584.42696	0.61996563	0.06476611	9.5723771	1.04E-21	6.02E-21	Up	PABPC4
CDH24	419.978333	1.13008077	0.11806488	9.57169285	1.05E-21	6.05E-21	Up	CDH24
SLC25A12	744.182778	-0.63282948	0.06612883	-9.56964623	1.07E-21	6.17E-21	Down	SLC25A12
FLI1	299.245423	-1.2735462	0.13310839	-9.56773806	1.09E-21	6.29E-21	Down	FLI1
LSM14B	2092.60775	0.72723314	0.07601655	9.56677349	1.10E-21	6.34E-21	Up	LSM14B
ABCA6	61.3939753	-2.30106812	0.24058669	-9.56440306	1.13E-21	6.49E-21	Down	ABCA6
BCL7A	759.965366	0.72195532	0.07548791	9.56385414	1.13E-21	6.52E-21	Up	BCL7A
NRSN1	4.08266186	-3.3073354	0.34587258	-9.56229419	1.15E-21	6.62E-21	Down	NRSN1
ZNF195	946.372785	0.80012516	0.08367926	9.56180964	1.16E-21	6.65E-21	Up	ZNF195
DIP2C	604.492365	-1.17589362	0.12298071	-9.56161032	1.16E-21	6.66E-21	Down	DIP2C
NCAPD2	4459.45211	0.69336516	0.07252123	9.56085733	1.17E-21	6.70E-21	Up	NCAPD2
RNF138	924.092117	-0.85309296	0.08923308	-9.56027642	1.17E-21	6.74E-21	Down	RNF138
ZNF343	416.421054	0.85175407	0.08909755	9.5597923	1.18E-21	6.77E-21	Up	ZNF343
KRT9	2.66348482	-3.06783192	0.32091274	-9.55970751	1.18E-21	6.77E-21	Down	KRT9
POLR3K	855.865521	0.92930456	0.09721966	9.5588131	1.19E-21	6.83E-21	Up	POLR3K
KISS1R	11.5953631	3.10448251	0.32479755	9.55820786	1.20E-21	6.86E-21	Up	KISS1R
LSM7	3242.45121	1.29969765	0.13597717	9.55820498	1.20E-21	6.86E-21	Up	LSM7
OSR1	56.0305808	-2.32625244	0.24345151	-9.55530081	1.23E-21	7.06E-21	Down	OSR1
RPUSD1	1585.79703	1.12069354	0.11728613	9.55520935	1.23E-21	7.06E-21	Up	RPUSD1
FARSB	2770.08437	0.63798937	0.06676971	9.55507122	1.23E-21	7.07E-21	Up	FARSB
PGRMC2	2295.41741	-0.5614508	0.05879678	-9.54900532	1.31E-21	7.49E-21	Down	PGRMC2
HES4	321.758441	1.97742378	0.207144	9.54613127	1.35E-21	7.70E-21	Up	HES4
GCNT3	4305.6041	-2.10259248	0.22025987	-9.54596262	1.35E-21	7.71E-21	Down	GCNT3
MYOC	27.7773941	-4.88022915	0.5112459	-9.54575708	1.35E-21	7.72E-21	Down	MYOC
FBXL7	196.802318	-1.39396598	0.14603359	-9.54551625	1.35E-21	7.74E-21	Down	FBXL7
GNG11	419.244138	-1.22985671	0.12885546	-9.54446699	1.37E-21	7.82E-21	Down	GNG11
UBE2QL1	46.1163788	-1.98379976	0.20787861	-9.54306837	1.39E-21	7.92E-21	Down	UBE2QL1
CLDN8	238.403588	-5.05986089	0.53024292	-9.54253366	1.39E-21	7.96E-21	Down	CLDN8
TWIST1	104.387139	1.85643302	0.19454945	9.54221676	1.40E-21	7.98E-21	Up	TWIST1
C6orf136	997.759089	-0.75381254	0.07900023	-9.5419036	1.40E-21	8.00E-21	Down	C6orf136
CENPL	286.390193	0.7558125	0.07921862	9.54084358	1.42E-21	8.08E-21	Up	CENPL
OGT	6047.00768	1.02087419	0.1070054	9.54039889	1.42E-21	8.11E-21	Up	OGT
ZNF579	494.756226	1.3020429	0.1364842	9.53987979	1.43E-21	8.15E-21	Up	ZNF579
SLC31A2	39.4339338	-1.27299199	0.13347426	-9.53735935	1.47E-21	8.35E-21	Down	SLC31A2
TAOK3	1796.78547	-0.60272969	0.06320025	-9.5368252	1.47E-21	8.39E-21	Down	TAOK3
P4HA3	90.2511719	1.79224607	0.18794203	9.53616443	1.48E-21	8.44E-21	Up	P4HA3
EIF3M	5558.89481	0.63824637	0.06693662	9.53508586	1.50E-21	8.53E-21	Up	EIF3M
COMT	5088.27521	1.18528261	0.12431055	9.53485103	1.50E-21	8.54E-21	Up	COMT

KCNA3	25.7027475	-2.13910496	0.22435812	-9.53433286	1.51E-21	8.58E-21	Down	KCNA3
NPM2	76.2619288	1.66647209	0.17480406	9.53337174	1.52E-21	8.66E-21	Up	NPM2
COL4A6	47.3796131	-2.14636567	0.22519713	-9.53105269	1.56E-21	8.85E-21	Down	COL4A6
PTRH1	7.27434281	1.8945357	0.19877751	9.53093611	1.56E-21	8.86E-21	Up	PTRH1
TARBP2	1166.33979	0.95408517	0.10011397	9.52999059	1.57E-21	8.94E-21	Up	TARBP2
SLC27A5	386.0803	1.78402286	0.18725214	9.5273829	1.61E-21	9.16E-21	Up	SLC27A5
ZNF135	35.0354894	-1.61494532	0.16953169	-9.52592012	1.64E-21	9.29E-21	Down	ZNF135
S1PR5	23.4657146	1.34770798	0.14150409	9.52416271	1.66E-21	9.44E-21	Up	S1PR5
TMEM9	3366.70075	0.99631738	0.10467953	9.51778619	1.77E-21	1.00E-20	Up	TMEM9
SLC7A14	11.4361933	-3.71561818	0.39039053	-9.51769544	1.77E-21	1.00E-20	Down	SLC7A14
C3orf18	103.483375	-1.19405047	0.12550328	-9.51409776	1.83E-21	1.04E-20	Down	C3orf18
CLK2	1181.98269	0.71837368	0.07550866	9.51379226	1.84E-21	1.04E-20	Up	CLK2
ATAD3C	117.037684	2.10079582	0.22097415	9.50697563	1.96E-21	1.11E-20	Up	ATAD3C
MMP20	6.54195044	3.81741503	0.40155766	9.50651782	1.97E-21	1.12E-20	Up	MMP20
LUZP2	28.0718049	-2.24057298	0.23571557	-9.50540928	1.99E-21	1.13E-20	Down	LUZP2
NLGN3	45.6794656	-1.37317482	0.14447993	-9.50425995	2.01E-21	1.14E-20	Down	NLGN3
MMP27	1.44552977	-3.97315767	0.41806322	-9.5037246	2.03E-21	1.15E-20	Down	MMP27
C11orf54	1255.44492	-0.95567684	0.10057484	-9.50214632	2.06E-21	1.16E-20	Down	C11orf54
DLL3	21.3341892	2.74086726	0.28855801	9.49849656	2.13E-21	1.20E-20	Up	DLL3
MPV17	1339.28174	0.63488274	0.06684724	9.497516	2.15E-21	1.22E-20	Up	MPV17
CNTN1	65.7136364	-2.81782845	0.2967081	-9.49697175	2.16E-21	1.22E-20	Down	CNTN1
TSTD2	382.877646	-0.6531797	0.06878844	-9.49548677	2.19E-21	1.24E-20	Down	TSTD2
NOL3	760.528438	0.9052164	0.09533364	9.49524611	2.20E-21	1.24E-20	Up	NOL3
TMEM243	733.909853	0.79004177	0.0832086	9.49471242	2.21E-21	1.25E-20	Up	TMEM243
SNAP91	10.3051971	-3.12167716	0.32880734	-9.49394002	2.22E-21	1.26E-20	Down	SNAP91
DMRT3	7.98848172	3.91962674	0.41314467	9.48729843	2.37E-21	1.34E-20	Up	DMRT3
GABRP	235.207356	3.03840323	0.32034953	9.48465011	2.43E-21	1.37E-20	Up	GABRP
ARMC2	129.592559	1.34803305	0.14213288	9.48431552	2.44E-21	1.38E-20	Up	ARMC2
LRRC31	492.277971	-1.51345931	0.15960001	-9.48282738	2.47E-21	1.40E-20	Down	LRRC31
ORC1	537.920484	0.9898682	0.10439661	9.4818043	2.50E-21	1.41E-20	Up	ORC1
LCN6	4.09358492	-2.82459605	0.29794272	-9.48033241	2.53E-21	1.43E-20	Down	LCN6
DZIP1	157.561507	-1.39720933	0.1473859	-9.47993881	2.54E-21	1.43E-20	Down	DZIP1
A2M	10124.5145	-1.28504061	0.13555801	-9.47963616	2.55E-21	1.44E-20	Down	A2M
ZMIZ1	3489.33617	-0.80320058	0.08474155	-9.47823787	2.59E-21	1.46E-20	Down	ZMIZ1
CST6	25.3817464	2.97316318	0.31368371	9.47821998	2.59E-21	1.46E-20	Up	CST6
PSMC3IP	140.62015	0.86968618	0.09181886	9.47176009	2.75E-21	1.55E-20	Up	PSMC3IP
PHYH	1443.74077	-1.00134839	0.10572001	-9.47170127	2.75E-21	1.55E-20	Down	PHYH
BLK	40.6012796	-2.51128739	0.26520083	-9.4693798	2.82E-21	1.58E-20	Down	BLK
ZNF396	56.7529477	-1.03937533	0.10976335	-9.46923815	2.82E-21	1.58E-20	Down	ZNF396
PILRB	247.454899	1.64758912	0.17405907	9.46568937	2.92E-21	1.64E-20	Up	PILRB
FLVCR1	979.005745	0.81665359	0.08627692	9.46549325	2.92E-21	1.64E-20	Up	FLVCR1
OLFML1	193.295293	-1.35414804	0.14306234	-9.46544063	2.92E-21	1.64E-20	Down	OLFML1
ABCD2	21.1313528	-1.97127353	0.20827505	-9.46476076	2.94E-21	1.65E-20	Down	ABCD2
CLCN5	863.779922	1.03783006	0.1096796	9.46238052	3.01E-21	1.69E-20	Up	CLCN5
PSMB2	4716.98937	0.6130563	0.06481091	9.45915254	3.10E-21	1.74E-20	Up	PSMB2
LRRC55	16.7163639	-1.89942912	0.20082298	-9.45822575	3.13E-21	1.76E-20	Down	LRRC55
RHBDL1	195.413474	1.73775167	0.18377094	9.45607419	3.20E-21	1.79E-20	Up	RHBDL1
ALG8	1254.79008	0.66374081	0.07020194	9.45473665	3.24E-21	1.82E-20	Up	ALG8
SCARB1	4288.99788	1.01774587	0.10770804	9.44911706	3.42E-21	1.91E-20	Up	SCARB1
KBTBD11	1139.84148	-1.12488318	0.11904866	-9.44893612	3.42E-21	1.92E-20	Down	KBTBD11
WDR17	14.4532871	-2.67806129	0.28345593	-9.4478931	3.46E-21	1.94E-20	Down	WDR17
KRT27	0.72942314	-3.20777104	0.33956974	-9.44657497	3.50E-21	1.96E-20	Down	KRT27
RASL10B	108.18856	2.28443509	0.24184547	9.44584616	3.53E-21	1.97E-20	Up	RASL10B
DUSP3	2677.89323	-0.52528214	0.05561441	-9.44507311	3.55E-21	1.99E-20	Down	DUSP3
CNKSR2	14.3870281	-2.14338631	0.22693794	-9.4448125	3.56E-21	1.99E-20	Down	CNKSR2
CLDN5	349.654175	-1.76563921	0.18695072	-9.44440969	3.57E-21	2.00E-20	Down	CLDN5
SERPINE1	1599.55263	2.01210754	0.21308187	9.44288482	3.63E-21	2.03E-20	Up	SERPINE1
POMP	5452.50587	0.94139275	0.09970912	9.44139039	3.68E-21	2.06E-20	Up	POMP

KRCC1	1428.78525	-1.07327531	0.1137029	-9.43929574	3.75E-21	2.10E-20	Down	KRCC1
RCOR2	198.170366	1.48725044	0.15757095	9.43860816	3.78E-21	2.11E-20	Up	RCOR2
KLK12	191.273793	3.11480497	0.33001662	9.43832746	3.79E-21	2.11E-20	Up	KLK12
POU4F1	17.255758	2.80462184	0.29718341	9.43734311	3.82E-21	2.13E-20	Up	POU4F1
RRAGC	630.95897	-0.62349264	0.06612171	-9.42946983	4.12E-21	2.30E-20	Down	RRAGC
CALY	10.0448797	-2.65810181	0.28190572	-9.42904538	4.14E-21	2.31E-20	Down	CALY
SEMA4G	2250.97059	-1.02965717	0.10920395	-9.42875365	4.15E-21	2.31E-20	Down	SEMA4G
SLC43A1	1254.68215	1.19473799	0.12672191	9.42802997	4.18E-21	2.33E-20	Up	SLC43A1
CRYZL1	516.11122	-0.63721872	0.0676094	-9.42500164	4.30E-21	2.40E-20	Down	CRYZL1
C10orf105	1.73767561	-2.28798477	0.24283112	-9.42212328	4.42E-21	2.46E-20	Down	C10orf105
STK32B	32.7148434	-1.53615384	0.16307465	-9.4199425	4.51E-21	2.51E-20	Down	STK32B
MAS1L	1.23805505	-4.01618998	0.4263638	-9.41963179	4.53E-21	2.52E-20	Down	MAS1L
RPH3A	4.86810685	-2.40212053	0.2550449	-9.41842208	4.58E-21	2.55E-20	Down	RPH3A
STAB1	1721.67581	-1.41851534	0.15063084	-9.41716383	4.63E-21	2.58E-20	Down	STAB1
AGO2	3024.85167	0.91080098	0.09672507	9.41639023	4.67E-21	2.60E-20	Up	AGO2
DUS3L	1398.12582	1.06061403	0.11264016	9.41594919	4.69E-21	2.61E-20	Up	DUS3L
SPON2	2583.77512	1.31181446	0.1393708	9.41240505	4.85E-21	2.70E-20	Up	SPON2
NEK6	2630.98622	0.77415767	0.08226029	9.41107417	4.91E-21	2.73E-20	Up	NEK6
NAE1	1999.64468	0.6416807	0.06818922	9.41029575	4.95E-21	2.75E-20	Up	NAE1
MAST3	911.571704	-0.84752097	0.09006572	-9.41002857	4.96E-21	2.75E-20	Down	MAST3
ETS2	18899.6499	0.91881947	0.09765339	9.40898704	5.01E-21	2.78E-20	Up	ETS2
PDF	185.077026	1.55688405	0.16547323	9.40867615	5.02E-21	2.79E-20	Up	PDF
ARVCF	645.858773	1.05769421	0.11242646	9.40787581	5.06E-21	2.81E-20	Up	ARVCF
FCER2	28.3102709	-2.99393798	0.31824545	-9.40763809	5.07E-21	2.81E-20	Down	FCER2
DPY19L1	1667.48785	0.82811952	0.08805657	9.40440374	5.23E-21	2.90E-20	Up	DPY19L1
KLF10	2284.02908	-0.79690374	0.08479901	-9.39755951	5.58E-21	3.09E-20	Down	KLF10
TULP3	770.411743	0.73606425	0.07837414	9.39167266	5.91E-21	3.27E-20	Up	TULP3
DOCK3	35.6583046	-1.75729903	0.18712771	-9.39090753	5.95E-21	3.29E-20	Down	DOCK3
MED9	503.604912	-0.55462175	0.05906182	-9.39052952	5.97E-21	3.31E-20	Down	MED9
MIOS	1138.12926	0.53578085	0.05706534	9.38890217	6.06E-21	3.36E-20	Up	MIOS
CDK7	971.719567	0.71603679	0.0762651	9.38878685	6.07E-21	3.36E-20	Up	CDK7
KLHDC4	1463.17831	0.78555902	0.08367573	9.38813492	6.11E-21	3.38E-20	Up	KLHDC4
ZNF786	292.019708	0.6673816	0.07108801	9.38810291	6.11E-21	3.38E-20	Up	ZNF786
RNF103	1545.62765	-0.75868583	0.0808251	-9.38676049	6.19E-21	3.42E-20	Down	RNF103
TSPAN2	377.102058	-1.90816984	0.20333816	-9.38421886	6.34E-21	3.50E-20	Down	TSPAN2
PID1	784.900385	-1.61515407	0.17214078	-9.38275079	6.43E-21	3.55E-20	Down	PID1
TUT1	471.866867	0.73246048	0.07806962	9.38214515	6.46E-21	3.57E-20	Up	TUT1
LRFN4	1434.25733	1.16049746	0.12369183	9.38216778	6.46E-21	3.57E-20	Up	LRFN4
CXCL6	112.872215	2.36033772	0.25165508	9.3792571	6.64E-21	3.67E-20	Up	CXCL6
CCDC137	1121.69447	0.77929923	0.08308792	9.37921195	6.65E-21	3.67E-20	Up	CCDC137
PIGU	1934.73271	0.89565332	0.09550166	9.37840558	6.70E-21	3.69E-20	Up	PIGU
TMEM156	30.2049424	-1.6498122	0.1759157	-9.37842519	6.70E-21	3.69E-20	Down	TMEM156
SLC19A2	681.573727	0.88388881	0.09426536	9.37660204	6.81E-21	3.76E-20	Up	SLC19A2
GNL3L	576.919868	0.89908093	0.0959092	9.37429309	6.96E-21	3.84E-20	Up	GNL3L
EEPD1	1250.54445	1.03650398	0.11059199	9.37232447	7.10E-21	3.91E-20	Up	EEPD1
GIMAP4	434.248019	-1.30516685	0.13926857	-9.37158236	7.15E-21	3.93E-20	Down	GIMAP4
PCMTD2	2323.05802	1.04044202	0.11105903	9.36836945	7.37E-21	4.05E-20	Up	PCMTD2
LRRC61	937.586314	1.05722005	0.11286345	9.36724934	7.44E-21	4.10E-20	Up	LRRC61
PTH1R	22.1932115	-1.92250494	0.20528342	-9.36512507	7.60E-21	4.18E-20	Down	PTH1R
ACAA1	2040.81007	-0.84413938	0.0901481	-9.36391758	7.68E-21	4.23E-20	Down	ACAA1
DNER	29.5424882	-2.59515141	0.27714737	-9.36379586	7.69E-21	4.23E-20	Down	DNER
UCHL3	602.245399	0.99216551	0.105966	9.36305482	7.75E-21	4.26E-20	Up	UCHL3
C14orf28	123.895327	-0.8615114	0.09201393	-9.36283724	7.76E-21	4.27E-20	Down	C14orf28
TJP3	3679.61251	-1.09550895	0.11701394	-9.36220866	7.81E-21	4.29E-20	Down	TJP3
SPATA6L	117.391267	1.33868436	0.14300428	9.36114899	7.89E-21	4.33E-20	Up	SPATA6L
SLC19A1	1386.34798	0.98389009	0.10510806	9.3607483	7.92E-21	4.35E-20	Up	SLC19A1
HDAC9	181.96245	-1.74558595	0.18649392	-9.36001556	7.97E-21	4.38E-20	Down	HDAC9
PLAC1	19.2212663	2.78791628	0.29786806	9.35956759	8.01E-21	4.39E-20	Up	PLAC1

STX6	1405.97462	0.52145073	0.05571428	9.35937283	8.02E-21	4.40E-20	Up	STX6
CLCN1	22.8559333	2.21434397	0.23663533	9.35762193	8.16E-21	4.47E-20	Up	CLCN1
CYB561D1	540.214838	-0.78314307	0.08369152	-9.35749567	8.16E-21	4.48E-20	Down	CYB561D1
PLA2G4E	24.0498679	1.7735708	0.18957397	9.35556102	8.32E-21	4.56E-20	Up	PLA2G4E
ETNK1	2980.25594	-1.04786532	0.11203151	-9.35330885	8.49E-21	4.65E-20	Down	ETNK1
SCARB2	7245.50705	-0.63227099	0.06760043	-9.35306213	8.51E-21	4.66E-20	Down	SCARB2
RHOC	7793.55446	-0.89193211	0.09538877	-9.35049382	8.72E-21	4.78E-20	Down	RHOC
RBCK1	5893.19403	0.968562	0.10358966	9.34998716	8.77E-21	4.80E-20	Up	RBCK1
SLC27A6	7.05464887	-2.8432872	0.30423289	-9.34575886	9.12E-21	4.99E-20	Down	SLC27A6
CHRNA1	41.8908342	-1.71438677	0.18344284	-9.34561855	9.14E-21	5.00E-20	Down	CHRNA1
MIIP	992.836886	0.96579747	0.10336701	9.3433822	9.33E-21	5.10E-20	Up	MIIP
TESPA1	51.1486717	-1.55695278	0.16664655	-9.34284414	9.38E-21	5.12E-20	Down	TESPA1
KCTD2	965.09008	-0.53269175	0.05701594	-9.34285609	9.38E-21	5.12E-20	Down	KCTD2
ITM2B	12358.0482	-0.79956871	0.08559399	-9.34141222	9.51E-21	5.19E-20	Down	ITM2B
CCT8	7367.12153	0.69723802	0.07464645	9.34053884	9.58E-21	5.23E-20	Up	CCT8
AGRN	7547.06702	1.11933485	0.1198401	9.34023616	9.61E-21	5.25E-20	Up	AGRN
MYCT1	123.843496	-1.24455677	0.13324847	-9.34012056	9.62E-21	5.25E-20	Down	MYCT1
TTC16	15.2059272	2.17367251	0.23272498	9.34009128	9.63E-21	5.25E-20	Up	TTC16
RNF39	187.514905	1.4012718	0.15003848	9.33941616	9.69E-21	5.28E-20	Up	RNF39
PTBP1	13363.21	0.52913037	0.05666041	9.33862534	9.76E-21	5.32E-20	Up	PTBP1
COL5A1	8766.8219	1.54947505	0.16597674	9.33549535	1.01E-20	5.48E-20	Up	COL5A1
CUX2	4.13603351	-2.47479835	0.26513382	-9.33414816	1.02E-20	5.55E-20	Down	CUX2
CXCR5	5.6893196	-2.79265995	0.2992394	-9.33252752	1.03E-20	5.63E-20	Down	CXCR5
ITFG1	2046.60845	-0.60625398	0.06496703	-9.33171828	1.04E-20	5.67E-20	Down	ITFG1
RUVBL2	5090.77498	1.07154008	0.11484605	9.33022998	1.06E-20	5.75E-20	Up	RUVBL2
ZFP64	1131.75877	0.6429674	0.06894023	9.3264469	1.09E-20	5.96E-20	Up	ZFP64
KITLG	951.828739	-1.24011728	0.13296877	-9.32638026	1.10E-20	5.96E-20	Down	KITLG
RHCG	88.4338919	2.51205009	0.26935135	9.32629487	1.10E-20	5.96E-20	Up	RHCG
BIRC7	33.2584088	2.42129548	0.25967151	9.32445554	1.12E-20	6.07E-20	Up	BIRC7
SNCA	64.4195297	-1.94749586	0.20887568	-9.32370812	1.12E-20	6.11E-20	Down	SNCA
FCRL2	34.1256488	-2.36316946	0.25347093	-9.32323663	1.13E-20	6.13E-20	Down	FCRL2
TRAPPC8	1171.18064	-0.73968506	0.07935655	-9.32103289	1.15E-20	6.26E-20	Down	TRAPPC8
PSMC2	3344.57734	0.56895275	0.06105	9.3194557	1.17E-20	6.35E-20	Up	PSMC2
FNBP4	1831.91564	0.68485121	0.07350254	9.31738139	1.19E-20	6.47E-20	Up	FNBP4
IL1R2	466.400916	-2.05511032	0.22058073	-9.31681699	1.20E-20	6.50E-20	Down	IL1R2
SYT8	78.8533244	2.18390545	0.2344041	9.31683983	1.20E-20	6.50E-20	Up	SYT8
FOXP3	134.955867	1.4253385	0.15299703	9.31611873	1.21E-20	6.55E-20	Up	FOXP3
PRICKLE3	578.286561	0.87046266	0.09345361	9.31438272	1.23E-20	6.65E-20	Up	PRICKLE3
DNAJA3	3476.50281	0.67956244	0.07297149	9.31271198	1.25E-20	6.76E-20	Up	DNAJA3
NDRG1	13654.6153	-1.18135553	0.12686709	-9.31175741	1.26E-20	6.81E-20	Down	NDRG1
RPS6KA1	4609.98702	-0.81833572	0.08789937	-9.30991613	1.28E-20	6.93E-20	Down	RPS6KA1
ARHGAP15	134.946619	-1.30979878	0.1407057	-9.30878261	1.29E-20	7.00E-20	Down	ARHGAP15
PACSIN1	20.0450904	-1.71353211	0.18409537	-9.30784994	1.30E-20	7.06E-20	Down	PACSIN1
INCA1	84.6997971	-0.86774936	0.09324573	-9.30604938	1.33E-20	7.18E-20	Down	INCA1
ADK	1216.34958	0.85283928	0.09164505	9.30589523	1.33E-20	7.19E-20	Up	ADK
PPP3CB	1332.29806	-0.62119967	0.06676422	-9.30438001	1.35E-20	7.29E-20	Down	PPP3CB
ADAMTS14	321.549011	1.31353638	0.14117849	9.30408264	1.35E-20	7.31E-20	Up	ADAMTS14
IL4R	2997.19845	-0.64573623	0.06940802	-9.30348083	1.36E-20	7.35E-20	Down	IL4R
SLC46A1	223.272151	-1.1473466	0.12332673	-9.30330808	1.36E-20	7.36E-20	Down	SLC46A1
GLRX3	2456.34063	0.76933761	0.08271154	9.30145427	1.39E-20	7.49E-20	Up	GLRX3
ST6GALNAC	74.8303925	-1.39034773	0.14951031	-9.29934369	1.41E-20	7.63E-20	Down	ST6GALNAC3
ELOVL4	25.3172536	-1.55299594	0.16701506	-9.29853859	1.42E-20	7.69E-20	Down	ELOVL4
MS4A2	49.7008161	-2.10314494	0.22619764	-9.2978199	1.43E-20	7.74E-20	Down	MS4A2
ARNT	1546.33017	-0.5182313	0.05574282	-9.29682616	1.45E-20	7.81E-20	Down	ARNT
DNAJB4	356.179222	-1.11884908	0.12034895	-9.29670848	1.45E-20	7.81E-20	Down	DNAJB4
MYCN	97.439132	1.96306704	0.21116685	9.29628426	1.45E-20	7.84E-20	Up	MYCN
OLFM1	196.617873	-1.75289981	0.18857845	-9.29533486	1.47E-20	7.91E-20	Down	OLFM1
SLC44A2	4734.44814	-0.62687335	0.06744607	-9.29443859	1.48E-20	7.97E-20	Down	SLC44A2

CCL8	72.6240873	-2.34342162	0.25221991	-9.29118421	1.53E-20	8.22E-20	Down	CCL8
SLC8A3	7.42196522	-1.97405122	0.21247864	-9.29058646	1.53E-20	8.26E-20	Down	SLC8A3
RFK	1888.10644	-0.89796288	0.09665977	-9.28993372	1.54E-20	8.31E-20	Down	RFK
BEX1	16.698587	-2.74407802	0.29542501	-9.2885772	1.56E-20	8.42E-20	Down	BEX1
SERPINE2	3103.204	1.38202883	0.14880892	9.28727161	1.58E-20	8.52E-20	Up	SERPINE2
NTHL1	1328.57585	1.17107595	0.12611413	9.28584225	1.60E-20	8.63E-20	Up	NTHL1
PCED1A	886.109257	1.13549204	0.12229869	9.28458051	1.62E-20	8.73E-20	Up	PCED1A
TTC23	457.396294	-0.5840599	0.06292954	-9.28117261	1.68E-20	9.01E-20	Down	TTC23
ZNF593	206.004097	1.17567681	0.12667588	9.28098363	1.68E-20	9.02E-20	Up	ZNF593
NANS	3465.05672	-0.81026808	0.08730571	-9.28081468	1.68E-20	9.04E-20	Down	NANS
PTF1A	11.7868721	6.07208832	0.65429507	9.28035169	1.69E-20	9.07E-20	Up	PTF1A
SSRP1	7512.80582	0.58089962	0.06260796	9.2783673	1.72E-20	9.24E-20	Up	SSRP1
PCBD1	3680.94367	0.71077587	0.07661533	9.27720229	1.74E-20	9.34E-20	Up	PCBD1
CSPP1	593.892717	0.88758771	0.09572615	9.27215463	1.82E-20	9.79E-20	Up	CSPP1
CDH11	1794.65414	1.52812131	0.16482715	9.27105352	1.84E-20	9.89E-20	Up	CDH11
SLC28A3	269.842479	2.25491154	0.24325981	9.26956073	1.87E-20	1.00E-19	Up	SLC28A3
ZRANB3	193.076057	0.8429172	0.09093836	9.26910527	1.88E-20	1.01E-19	Up	ZRANB3
IFITM2	4297.15935	1.47176211	0.15879423	9.26836025	1.89E-20	1.01E-19	Up	IFITM2
FBXL6	1933.03364	1.2385984	0.13364757	9.26764614	1.90E-20	1.02E-19	Up	FBXL6
RLIM	2014.02326	0.86803886	0.09366401	9.26758135	1.90E-20	1.02E-19	Up	RLIM
SLC9A5	50.8211005	1.19676647	0.12913448	9.26759817	1.90E-20	1.02E-19	Up	SLC9A5
RGL2	1570.87538	0.83163211	0.08973734	9.26740295	1.91E-20	1.02E-19	Up	RGL2
ENTPD3	45.4032182	-2.21681211	0.23921529	-9.2670169	1.91E-20	1.02E-19	Down	ENTPD3
PKIG	1367.51864	-0.97442845	0.10516316	-9.26587263	1.93E-20	1.04E-19	Down	PKIG
BTLA	23.910966	-1.77808905	0.19198367	-9.26166805	2.01E-20	1.08E-19	Down	BTLA
GRWD1	1841.34522	0.66266345	0.07155896	9.26038416	2.04E-20	1.09E-19	Up	GRWD1
FOXP2	216.56427	-2.29336789	0.24768709	-9.25913381	2.06E-20	1.10E-19	Down	FOXP2
ZNF491	24.6195582	-1.10450285	0.11929444	-9.25862813	2.07E-20	1.11E-19	Down	ZNF491
SYP	134.359509	-1.67033216	0.18045099	-9.2564311	2.11E-20	1.13E-19	Down	SYP
CPEB2	591.603567	-1.00260436	0.1083422	-9.25405201	2.16E-20	1.15E-19	Down	CPEB2
FOXK1	2814.38479	0.77668877	0.08394285	9.25259052	2.19E-20	1.17E-19	Up	FOXK1
SHISA3	65.3738637	-2.34938926	0.25412888	-9.24487329	2.36E-20	1.26E-19	Down	SHISA3
KIF11	1426.04186	0.89600669	0.09692774	9.24406899	2.37E-20	1.27E-19	Up	KIF11
CNPY2	860.906076	0.65017426	0.07033671	9.24374028	2.38E-20	1.27E-19	Up	CNPY2
SPICE1	663.84484	0.8135535	0.08801929	9.24290014	2.40E-20	1.28E-19	Up	SPICE1
TBCD	3287.06292	0.78397398	0.08482215	9.24256186	2.41E-20	1.28E-19	Up	TBCD
ZFPM1	576.423267	1.38362299	0.14973904	9.24022865	2.46E-20	1.31E-19	Up	ZFPM1
PTMA	39683.7029	0.59689323	0.06459956	9.23989675	2.47E-20	1.31E-19	Up	PTMA
NUP160	2144.89001	0.55896883	0.06050815	9.23791012	2.51E-20	1.34E-19	Up	NUP160
HPS4	1399.26051	0.60621702	0.06562441	9.23767603	2.52E-20	1.34E-19	Up	HPS4
LCN2	22911.4767	2.32005124	0.25120951	9.23552303	2.57E-20	1.37E-19	Up	LCN2
UCHL1	195.03934	-2.07933418	0.22516424	-9.23474437	2.59E-20	1.38E-19	Down	UCHL1
KIF12	1189.23814	1.61900931	0.17537635	9.23162871	2.67E-20	1.42E-19	Up	KIF12
AP5M1	1542.9754	-0.78224947	0.08474115	-9.23104632	2.68E-20	1.43E-19	Down	AP5M1
TSEN54	1809.40598	0.75453045	0.08174474	9.23032453	2.70E-20	1.43E-19	Up	TSEN54
MSRA	435.240838	-0.90898228	0.09848634	-9.2295261	2.72E-20	1.44E-19	Down	MSRA
EPB41L4A	103.426642	-1.68594989	0.18267353	-9.22930608	2.72E-20	1.45E-19	Down	EPB41L4A
CDC42	7337.95562	-0.7192182	0.07793539	-9.22839096	2.75E-20	1.46E-19	Down	CDC42
ABCC10	1418.10072	0.7709463	0.08354266	9.22817544	2.75E-20	1.46E-19	Up	ABCC10
POP7	1248.61755	0.81259368	0.08806391	9.22731813	2.77E-20	1.47E-19	Up	POP7
KIAA0232	1549.34969	-0.7381281	0.08000721	-9.22576984	2.82E-20	1.49E-19	Down	KIAA0232
CX3CR1	39.0491779	-1.57856481	0.17111735	-9.22504238	2.83E-20	1.50E-19	Down	CX3CR1
CPLX1	263.747045	1.69647756	0.18390159	9.22492068	2.84E-20	1.50E-19	Up	CPLX1
CALML4	835.343076	-0.86656639	0.09393979	-9.22469971	2.84E-20	1.51E-19	Down	CALML4
IQCB1	916.143697	0.6846469	0.07421997	9.2245647	2.85E-20	1.51E-19	Up	IQCB1
ACKR1	283.961155	-2.20561647	0.23910432	-9.22449462	2.85E-20	1.51E-19	Down	ACKR1
PLEKHG5	1090.63692	1.0143494	0.10996648	9.22416868	2.86E-20	1.51E-19	Up	PLEKHG5
MKI67	6070.46562	1.01469171	0.11000431	9.22410862	2.86E-20	1.51E-19	Up	MKI67

MTHFR	876.346103	-0.78543354	0.08515675	-9.22338547	2.88E-20	1.52E-19	Down	MTHFR
ROR1	110.327537	-1.85381057	0.20100069	-9.22290654	2.89E-20	1.53E-19	Down	ROR1
GPR4	199.67857	1.15239702	0.12495214	9.2227073	2.90E-20	1.53E-19	Up	GPR4
TSR1	1636.32245	0.64136056	0.06955231	9.22126843	2.94E-20	1.55E-19	Up	TSR1
DPEP3	3.81146304	-2.38579079	0.25872999	-9.2211607	2.94E-20	1.55E-19	Down	DPEP3
LEPR	235.138031	-1.40593094	0.15252414	-9.2177599	3.03E-20	1.60E-19	Down	LEPR
SRSF7	4649.59428	0.52391429	0.05685814	9.21441114	3.13E-20	1.65E-19	Up	SRSF7
TREX2	21.6329071	1.38487163	0.15030276	9.21387998	3.15E-20	1.66E-19	Up	TREX2
HECW2	198.985306	1.37531122	0.14927062	9.21354279	3.16E-20	1.67E-19	Up	HECW2
PABPC5	13.9816121	-1.6943966	0.18391053	-9.21315707	3.17E-20	1.67E-19	Down	PABPC5
PHF7	146.734577	-0.86071345	0.09342553	-9.21282911	3.18E-20	1.68E-19	Down	PHF7
R3HDML	64.5278467	2.04907615	0.22244964	9.21141579	3.22E-20	1.70E-19	Up	R3HDML
GATC	1042.53743	0.56428359	0.06126027	9.21124938	3.22E-20	1.70E-19	Up	GATC
EIF3H	10638.3612	0.68865524	0.07481529	9.20473893	3.43E-20	1.81E-19	Up	EIF3H
VGF	122.13198	2.14388376	0.23295509	9.20299157	3.48E-20	1.84E-19	Up	VGF
ZNF860	64.8661345	1.04820285	0.11394251	9.19940136	3.60E-20	1.90E-19	Up	ZNF860
LTBP4	4926.27745	-1.29963918	0.14131563	-9.19671219	3.69E-20	1.94E-19	Down	LTBP4
ZBTB18	1627.7836	0.82772785	0.09000728	9.1962321	3.71E-20	1.95E-19	Up	ZBTB18
HTR3E	9.58580521	-3.25522209	0.3540992	-9.19296652	3.82E-20	2.01E-19	Down	HTR3E
DPYSL5	7.33624325	-2.862594	0.31150211	-9.18964571	3.94E-20	2.07E-19	Down	DPYSL5
CDC7	332.597884	1.00769474	0.10966195	9.1891012	3.96E-20	2.08E-19	Up	CDC7
SLC37A2	416.504438	-1.95492653	0.21282497	-9.18560679	4.09E-20	2.15E-19	Down	SLC37A2
GNGT1	12.8325075	3.48889505	0.37982391	9.18555942	4.09E-20	2.15E-19	Up	GNGT1
TMEM45B	5471.96381	-1.05706894	0.11509255	-9.1845126	4.13E-20	2.17E-19	Down	TMEM45B
TNFRSF13C	82.8201716	-1.78289609	0.19413787	-9.18365928	4.17E-20	2.19E-19	Down	TNFRSF13C
CHMP6	714.703446	-0.7286125	0.07936823	-9.18015273	4.30E-20	2.26E-19	Down	CHMP6
PRIM1	433.583823	0.84276854	0.09180695	9.17978999	4.32E-20	2.27E-19	Up	PRIM1
SHOC2	1438.93252	-0.75799281	0.08257591	-9.17934563	4.34E-20	2.28E-19	Down	SHOC2
BCAP31	13061.6814	0.81958775	0.08931598	9.17627218	4.46E-20	2.34E-19	Up	BCAP31
ZFP91	2453.93076	-0.67963493	0.07406492	-9.17620616	4.47E-20	2.34E-19	Down	ZFP91
HMGXB3	1891.36332	0.5212641	0.0568159	9.17461734	4.53E-20	2.38E-19	Up	HMGXB3
ANKS3	512.458983	0.83945767	0.0915072	9.17367853	4.57E-20	2.40E-19	Up	ANKS3
ZCCHC12	11.8680416	-1.66950546	0.18201022	-9.17259199	4.62E-20	2.42E-19	Down	ZCCHC12
IFITM5	3.6030869	4.19762238	0.45763074	9.17250957	4.62E-20	2.42E-19	Up	IFITM5
KIF15	610.983963	0.88671225	0.09668776	9.17088422	4.69E-20	2.46E-19	Up	KIF15
OTUD6B	594.694475	1.06492978	0.1161263	9.17044406	4.71E-20	2.47E-19	Up	OTUD6B
FZD10	270.656601	3.29802481	0.35972189	9.1682627	4.81E-20	2.52E-19	Up	FZD10
WSB2	3203.21198	-0.5765163	0.06288656	-9.16755947	4.84E-20	2.53E-19	Down	WSB2
CTSH	4867.17687	1.07619804	0.11740179	9.16679406	4.87E-20	2.55E-19	Up	CTSH
SPRR3	20.2931524	5.24586954	0.57249311	9.16320114	5.04E-20	2.64E-19	Up	SPRR3
RNASEH2B	1334.6801	0.81228117	0.08866983	9.16073851	5.15E-20	2.70E-19	Up	RNASEH2B
SLC9A3R1	4362.32597	-0.98515392	0.10754943	-9.16001045	5.19E-20	2.71E-19	Down	SLC9A3R1
NAT2	200.650015	-1.71566353	0.18734822	-9.15761873	5.31E-20	2.77E-19	Down	NAT2
CFAP20	1113.86581	0.56242782	0.06141688	9.1575446	5.31E-20	2.78E-19	Up	CFAP20
FECH	519.568294	-0.90611321	0.09896091	-9.15627423	5.37E-20	2.81E-19	Down	FECH
UPK1A	11.1286595	3.58300743	0.39131877	9.15623717	5.37E-20	2.81E-19	Up	UPK1A
LHFPL2	1543.26325	-0.85739376	0.09366869	-9.15347214	5.51E-20	2.88E-19	Down	LHFPL2
RPS21	31497.3016	1.43340112	0.15662557	9.15176929	5.60E-20	2.92E-19	Up	RPS21
ZNF182	283.364163	0.82125631	0.08973963	9.15154521	5.61E-20	2.93E-19	Up	ZNF182
CHST5	199.129727	-2.52495902	0.27596289	-9.14963235	5.71E-20	2.98E-19	Down	CHST5
TTC9	327.738526	1.54174042	0.16854486	9.14735964	5.83E-20	3.04E-19	Up	TTC9
MUC16	42.6883039	3.53659666	0.38683489	9.14239319	6.11E-20	3.19E-19	Up	MUC16
EEF2K	1458.22891	-0.61313578	0.06707674	-9.14081077	6.20E-20	3.23E-19	Down	EEF2K
PPP1CB	7313.74526	-0.73904604	0.08086867	-9.13884275	6.31E-20	3.29E-19	Down	PPP1CB
UBTD1	299.94783	1.1685436	0.12786828	9.13865105	6.32E-20	3.29E-19	Up	UBTD1
KLF6	7022.50538	-0.89983637	0.09846997	-9.13818024	6.35E-20	3.31E-19	Down	KLF6
FXN	462.029119	0.72926378	0.07980404	9.13818085	6.35E-20	3.31E-19	Up	FXN
CYP2C18	116.042686	-2.04289501	0.22356976	-9.13761776	6.38E-20	3.32E-19	Down	CYP2C18

ZBTB22	571.632941	-0.68817309	0.07531809	-9.13688984	6.43E-20	3.35E-19	Down	ZBTB22
FOXRED2	882.493982	1.02701803	0.11244386	9.13360739	6.63E-20	3.45E-19	Up	FOXRED2
KRTAP13-2	12.6820247	-5.20975726	0.57042304	-9.13314669	6.65E-20	3.46E-19	Down	KRTAP13-2
FAM222A	411.149348	1.4784517	0.16190047	9.13185541	6.73E-20	3.50E-19	Up	FAM222A
AREG	3338.11774	1.79781757	0.19688117	9.1314855	6.76E-20	3.51E-19	Up	AREG
ENOX1	50.3812201	-1.45870697	0.15976167	-9.13051904	6.82E-20	3.54E-19	Down	ENOX1
ARHGEF6	371.365342	-1.27577401	0.13972638	-9.13051659	6.82E-20	3.54E-19	Down	ARHGEF6
ARNTL	305.592165	-0.75986782	0.08322668	-9.13009918	6.84E-20	3.55E-19	Down	ARNTL
PRKCD	2560.01676	-0.73117698	0.08012966	-9.1249236	7.18E-20	3.73E-19	Down	PRKCD
SNX9	3474.30821	-0.54765343	0.0600331	-9.12252513	7.34E-20	3.81E-19	Down	SNX9
SLCO1B1	4.84742061	4.74148588	0.51977266	9.12223029	7.36E-20	3.82E-19	Up	SLCO1B1
MARS2	804.673716	0.68583401	0.07518509	9.12194199	7.38E-20	3.83E-19	Up	MARS2
TBC1D9	521.451408	-1.24609235	0.13663508	-9.11985647	7.52E-20	3.90E-19	Down	TBC1D9
WNT2B	239.455719	-1.22741316	0.13459265	-9.11946661	7.55E-20	3.91E-19	Down	WNT2B
ASB5	20.640306	-4.74530935	0.52035972	-9.11928652	7.56E-20	3.92E-19	Down	ASB5
SCARA3	329.190139	-1.60760231	0.17631027	-9.11802984	7.65E-20	3.96E-19	Down	SCARA3
SNRPD1	2007.12973	0.7459304	0.08181417	9.11737445	7.70E-20	3.98E-19	Up	SNRPD1
DUS1L	5544.07826	0.90598043	0.09936858	9.11737308	7.70E-20	3.98E-19	Up	DUS1L
DPYD	358.861897	-1.69881605	0.18633486	-9.11700623	7.72E-20	4.00E-19	Down	DPYD
MBOAT1	549.483578	-0.95265731	0.10450486	-9.11591438	7.80E-20	4.04E-19	Down	MBOAT1
SI	807.823844	-3.12553177	0.34290314	-9.11491152	7.87E-20	4.07E-19	Down	SI
NCBP2	3494.5466	0.66062508	0.07248622	9.11380264	7.95E-20	4.11E-19	Up	NCBP2
CSTF3	953.514229	0.60665265	0.06656782	9.11330227	7.99E-20	4.13E-19	Up	CSTF3
C3orf70	387.208349	-1.40917055	0.15465952	-9.11143745	8.13E-20	4.20E-19	Down	C3orf70
R3HDM4	3689.38122	0.67207342	0.07376221	9.11135151	8.14E-20	4.20E-19	Up	R3HDM4
FOXI3	6.85379865	5.32842499	0.58483306	9.11101877	8.16E-20	4.22E-19	Up	FOXI3
LURAP1	11.4363681	-1.36449413	0.14976502	-9.11090034	8.17E-20	4.22E-19	Down	LURAP1
SIX2	51.0324414	3.00669769	0.33003804	9.11015493	8.23E-20	4.25E-19	Up	SIX2
PTCD2	376.11694	0.6158719	0.06760697	9.1095923	8.27E-20	4.27E-19	Up	PTCD2
CCL25	47.5095881	4.19476366	0.46064136	9.10635482	8.52E-20	4.40E-19	Up	CCL25
TRIM24	1603.50234	0.70991371	0.07796635	9.10538558	8.60E-20	4.43E-19	Up	TRIM24
S100A3	64.2124174	1.88964845	0.20756687	9.10380561	8.72E-20	4.50E-19	Up	S100A3
NELFA	1809.31481	0.67627777	0.07430641	9.10120381	8.93E-20	4.61E-19	Up	NELFA
ACADSB	974.089414	-0.87816227	0.09649539	-9.10056175	8.99E-20	4.63E-19	Down	ACADSB
HDGF	14592.6665	0.59028242	0.06489047	9.09659677	9.32E-20	4.80E-19	Up	HDGF
TSHZ3	218.055799	-1.37624587	0.15131056	-9.09550463	9.41E-20	4.85E-19	Down	TSHZ3
PNOC	20.91829	-2.09378667	0.23020435	-9.09533922	9.43E-20	4.85E-19	Down	PNOC
AKAP9	2124.19381	-1.08746029	0.11957011	-9.09474994	9.48E-20	4.88E-19	Down	AKAP9
DCLK2	109.440133	-1.33904266	0.14727389	-9.0921929	9.71E-20	4.99E-19	Down	DCLK2
CACNB1	236.335531	-1.11669666	0.12282881	-9.09148836	9.77E-20	5.03E-19	Down	CACNB1
POLD1	1648.67165	1.03377602	0.11372705	9.08997512	9.91E-20	5.09E-19	Up	POLD1
POGK	2420.16723	0.53396802	0.05875464	9.0880991	1.01E-19	5.18E-19	Up	POGK
ELOVL6	1287.23742	-0.94297383	0.10380693	-9.08392018	1.05E-19	5.38E-19	Down	ELOVL6
GSG1L	3.59971643	-2.80437738	0.30879337	-9.08172793	1.07E-19	5.49E-19	Down	GSG1L
CDK5RAP3	3138.49664	0.78395854	0.08633544	9.08037921	1.08E-19	5.56E-19	Up	CDK5RAP3
DAB2IP	4095.5884	-0.8269539	0.09107605	-9.07981791	1.09E-19	5.59E-19	Down	DAB2IP
KRT6C	4.81239012	4.60782328	0.50749819	9.07948717	1.09E-19	5.60E-19	Up	KRT6C
CDK8	1404.04985	0.74642834	0.08221845	9.07859889	1.10E-19	5.64E-19	Up	CDK8
IL17C	13.7938271	2.92856515	0.32260836	9.07777203	1.11E-19	5.69E-19	Up	IL17C
TSNARE1	699.798626	1.00357823	0.11057035	9.07637755	1.12E-19	5.76E-19	Up	TSNARE1
HHIP	156.288629	-1.92914225	0.21255989	-9.07575877	1.13E-19	5.79E-19	Down	HHIP
CNPPD1	3431.03663	-0.64865907	0.07148487	-9.07407549	1.15E-19	5.88E-19	Down	CNPPD1
RAB1B	5585.40297	-0.67821801	0.07474679	-9.07354032	1.15E-19	5.90E-19	Down	RAB1B
PDP1	1277.64039	0.93756764	0.10333273	9.07328798	1.15E-19	5.92E-19	Up	PDP1
RPS6KA5	452.985872	-1.10531686	0.12182198	-9.07321393	1.16E-19	5.92E-19	Down	RPS6KA5
ANGPT1	97.4827696	-1.57275041	0.17334145	-9.07313503	1.16E-19	5.92E-19	Down	ANGPT1
PSPC1	1437.62158	0.5400064	0.05953207	9.07084812	1.18E-19	6.05E-19	Up	PSPC1
RPL36A	3681.43861	0.98189023	0.10830281	9.0661563	1.23E-19	6.31E-19	Up	RPL36A

INA	24.5020076	-2.71435443	0.29947429	-9.06373113	1.26E-19	6.45E-19	Down	INA
SLAMF7	561.710111	-1.78916178	0.1974107	-9.06314484	1.27E-19	6.48E-19	Down	SLAMF7
NUP62	2861.0729	0.54600112	0.06025018	9.06223269	1.28E-19	6.54E-19	Up	NUP62
CCDC34	745.72008	0.86031799	0.09493519	9.06216081	1.28E-19	6.54E-19	Up	CCDC34
ALKBH6	91.0634887	0.94724316	0.10452815	9.06208663	1.28E-19	6.54E-19	Up	ALKBH6
SYT10	3.27710272	-3.53211874	0.38989678	-9.05911228	1.32E-19	6.72E-19	Down	SYT10
SERPINB2	26.4076532	3.59978099	0.39744149	9.05738593	1.34E-19	6.82E-19	Up	SERPINB2
GIT1	3711.7821	0.81567592	0.09006922	9.05610062	1.35E-19	6.90E-19	Up	GIT1
MEI1	98.770849	-1.48662305	0.16421943	-9.0526624	1.40E-19	7.12E-19	Down	MEI1
SH3BP1	1773.3761	-0.93599962	0.1034205	-9.0504262	1.42E-19	7.27E-19	Down	SH3BP1
AOX1	124.233337	-1.87882163	0.20760275	-9.05008061	1.43E-19	7.29E-19	Down	AOX1
TUSC3	251.051573	-1.42102608	0.15704302	-9.04864218	1.45E-19	7.38E-19	Down	TUSC3
OAF	3209.9681	-0.84255792	0.09312183	-9.04790993	1.46E-19	7.43E-19	Down	OAF
MKS1	529.995598	0.58853992	0.06505137	9.04731	1.47E-19	7.47E-19	Up	MKS1
CAMK4	89.5806197	-1.4389025	0.15907652	-9.0453479	1.49E-19	7.60E-19	Down	CAMK4
ALDH6A1	944.413323	-0.95129834	0.10518591	-9.04397123	1.51E-19	7.70E-19	Down	ALDH6A1
FBLN5	563.005217	-1.34865414	0.14918402	-9.04020522	1.56E-19	7.96E-19	Down	FBLN5
KCNIP3	47.7033665	-1.62951348	0.18028844	-9.03836912	1.59E-19	8.10E-19	Down	KCNIP3
CHSY3	64.6260015	1.38593891	0.15333982	9.03834985	1.59E-19	8.10E-19	Up	CHSY3
ITSN1	958.39216	-0.74186114	0.08210475	-9.03554492	1.63E-19	8.30E-19	Down	ITSN1
SNAP23	1707.14229	-0.62396779	0.06906653	-9.0343005	1.65E-19	8.40E-19	Down	SNAP23
CR2	198.67699	-2.92694783	0.32400966	-9.03352013	1.66E-19	8.45E-19	Down	CR2
ACTA2	7322.62403	-1.55685662	0.17249372	-9.02558435	1.79E-19	9.09E-19	Down	ACTA2
THY1	3323.63552	1.29330566	0.14329525	9.02546077	1.79E-19	9.10E-19	Up	THY1
BMS1	2035.16255	0.56537408	0.0626544	9.02369273	1.82E-19	9.24E-19	Up	BMS1
PTCD1	348.598705	0.81725142	0.09059115	9.02131643	1.86E-19	9.44E-19	Up	PTCD1
DTX4	2997.40929	-0.6465431	0.07166855	-9.02129465	1.86E-19	9.44E-19	Down	DTX4
CTNNBL1	2329.76991	0.79531884	0.08816347	9.02095687	1.86E-19	9.47E-19	Up	CTNNBL1
RHBG	5.3608441	3.37804422	0.37447236	9.02081049	1.87E-19	9.48E-19	Up	RHBG
APBA1	234.999838	-1.28462649	0.14240955	-9.02064866	1.87E-19	9.49E-19	Down	APBA1
ITGBL1	179.662557	2.32229196	0.25744273	9.02061569	1.87E-19	9.49E-19	Up	ITGBL1
RTEL1	52.7976998	1.03361852	0.11458386	9.02062928	1.87E-19	9.49E-19	Up	RTEL1
KCNA1	5.50060956	-3.85634894	0.42758261	-9.0189565	1.90E-19	9.63E-19	Down	KCNA1
AADAC	23.1951753	2.71778976	0.30151597	9.0137505	1.99E-19	1.01E-18	Up	AADAC
ABCF2	2762.95294	0.57863122	0.06419897	9.01309257	2.00E-19	1.01E-18	Up	ABCF2
RRP15	830.74186	0.77642945	0.08614616	9.01293144	2.01E-19	1.02E-18	Up	RRP15
SMS	3501.75836	0.69036683	0.07660599	9.01191725	2.02E-19	1.03E-18	Up	SMS
SH3GLB1	3741.59341	-0.57654946	0.06398285	-9.0109993	2.04E-19	1.03E-18	Down	SH3GLB1
TMEM165	2995.9218	0.74464343	0.08264489	9.01015674	2.06E-19	1.04E-18	Up	TMEM165
SLC20A2	2186.37591	-0.80122783	0.08896943	-9.00565278	2.14E-19	1.08E-18	Down	SLC20A2
MGME1	1000.24399	0.79776386	0.08860105	9.00399962	2.18E-19	1.10E-18	Up	MGME1
DNAAF3	34.097716	1.94709816	0.21627074	9.00305868	2.20E-19	1.11E-18	Up	DNAAF3
PTX3	29.9272689	-1.75405765	0.19487141	-9.00110329	2.23E-19	1.13E-18	Down	PTX3
SASH1	664.485547	-0.89712427	0.09967513	-9.00048222	2.25E-19	1.14E-18	Down	SASH1
NACC2	1277.66271	-0.87371503	0.09707871	-9.00006848	2.26E-19	1.14E-18	Down	NACC2
MS4A6A	723.813601	-1.4409592	0.16016923	-8.99647977	2.33E-19	1.18E-18	Down	MS4A6A
PAN3	2024.27244	0.99930574	0.11108698	8.99570547	2.35E-19	1.18E-18	Up	PAN3
HSPE1	4668.90676	0.87924649	0.09776036	8.99389579	2.39E-19	1.20E-18	Up	HSPE1
ZNF540	25.351706	-1.35375909	0.15052732	-8.99344477	2.40E-19	1.21E-18	Down	ZNF540
MZT2A	1693.32264	1.06962078	0.11896574	8.99099849	2.45E-19	1.24E-18	Up	MZT2A
PKP2	4486.94239	-0.78566392	0.08739742	-8.98955536	2.48E-19	1.25E-18	Down	PKP2
MIXL1	10.9910269	-1.93916611	0.2157185	-8.98933608	2.49E-19	1.25E-18	Down	MIXL1
FAM110D	36.2400739	-1.24077004	0.13807255	-8.98636261	2.56E-19	1.29E-18	Down	FAM110D
RFC5	823.84593	0.6480573	0.07211708	8.98618361	2.56E-19	1.29E-18	Up	RFC5
PFAS	1140.64191	0.75308519	0.0838257	8.98394201	2.61E-19	1.32E-18	Up	PFAS
TNFRSF11B	638.491993	1.97196079	0.21952889	8.98269394	2.64E-19	1.33E-18	Up	TNFRSF11B
NIT1	975.189843	-0.63545068	0.07074831	-8.98184966	2.66E-19	1.34E-18	Down	NIT1
UBE2J1	3343.46118	-0.577606	0.06432123	-8.98002117	2.71E-19	1.36E-18	Down	UBE2J1

MORF4L2	7494.19611	0.62620264	0.06974299	8.97871797	2.74E-19	1.38E-18	Up	MORF4L2
TFB1M	516.842078	0.66041248	0.07355659	8.9782908	2.75E-19	1.38E-18	Up	TFB1M
DSTN	14252.5826	-0.84801503	0.09446942	-8.97660886	2.79E-19	1.40E-18	Down	DSTN
MRPL57	2516.43703	0.89932313	0.10019199	8.97599825	2.81E-19	1.41E-18	Up	MRPL57
FAM214B	805.379939	-0.89767525	0.1000152	-8.97538831	2.82E-19	1.42E-18	Down	FAM214B
FARSA	3599.29981	0.76242759	0.08494889	8.97513277	2.83E-19	1.42E-18	Up	FARSA
PKM	41926.8881	0.90419467	0.1007456	8.97502862	2.83E-19	1.42E-18	Up	PKM
LPP	5064.30905	-1.31231125	0.14621808	-8.97502749	2.83E-19	1.42E-18	Down	LPP
PRX	104.022134	-1.14114398	0.12715287	-8.97458315	2.84E-19	1.43E-18	Down	PRX
NSMF	3629.4297	1.17509345	0.13094181	8.97416556	2.86E-19	1.43E-18	Up	NSMF
RIMS4	15.0334373	-3.391763	0.3779532	-8.97402912	2.86E-19	1.43E-18	Down	RIMS4
QPCTL	635.629531	0.87130968	0.09711572	8.97187051	2.92E-19	1.46E-18	Up	QPCTL
WNT9A	91.259712	-1.67883403	0.18714112	-8.97095213	2.94E-19	1.47E-18	Down	WNT9A
GNAL	179.241539	-1.25738095	0.14017165	-8.97029406	2.96E-19	1.48E-18	Down	GNAL
F2RL2	237.261345	1.54604275	0.17236029	8.96983128	2.97E-19	1.49E-18	Up	F2RL2
GRAMD1C	234.147864	-1.32098012	0.14728258	-8.96901793	2.99E-19	1.50E-18	Down	GRAMD1C
PDLIM2	577.20894	-1.08354346	0.12081091	-8.96892023	2.99E-19	1.50E-18	Down	PDLIM2
PPAN	298.658663	0.92975779	0.10368567	8.96708087	3.04E-19	1.52E-18	Up	PPAN
SEL1L	3553.06158	-0.7759667	0.08653782	-8.9667925	3.05E-19	1.53E-18	Down	SEL1L
ZWILCH	790.442713	0.80986028	0.09037016	8.96158934	3.20E-19	1.60E-18	Up	ZWILCH
PFDN4	869.736625	1.12997501	0.12612674	8.95904377	3.28E-19	1.64E-18	Up	PFDN4
INCENP	1279.93564	0.70115935	0.07826505	8.95877985	3.28E-19	1.64E-18	Up	INCENP
EBF1	115.141208	-1.43264641	0.15992731	-8.95811	3.30E-19	1.65E-18	Down	EBF1
CALM2	17398.4032	-0.68878714	0.07689206	-8.95784448	3.31E-19	1.65E-18	Down	CALM2
IZUMO1	6.63001305	2.37304553	0.26493608	8.95704941	3.33E-19	1.67E-18	Up	IZUMO1
TERT	90.4141684	1.71633829	0.19163408	8.95633132	3.36E-19	1.68E-18	Up	TERT
PRR33	13.1660916	-1.71381401	0.19137638	-8.95520154	3.39E-19	1.69E-18	Down	PRR33
CLIC3	275.979148	2.02700425	0.22636752	8.95448367	3.41E-19	1.70E-18	Up	CLIC3
CCL21	659.494161	-2.20114532	0.24585007	-8.95320205	3.45E-19	1.72E-18	Down	CCL21
SAR1B	1780.63832	-0.67491419	0.07539781	-8.95137608	3.51E-19	1.75E-18	Down	SAR1B
ZBTB4	1845.74906	-0.80799897	0.09028474	-8.94945257	3.57E-19	1.78E-18	Down	ZBTB4
ENPP2	639.267552	-1.46723788	0.16395419	-8.94907235	3.58E-19	1.79E-18	Down	ENPP2
ALB	55.6716275	5.16284694	0.576983	8.94800538	3.62E-19	1.80E-18	Up	ALB
LCN10	2.66665881	-2.66175797	0.29749291	-8.94729875	3.64E-19	1.82E-18	Down	LCN10
SRRM5	25.370369	1.18823643	0.13280949	8.94692424	3.66E-19	1.82E-18	Up	SRRM5
VASH2	95.5040363	1.31415053	0.14690263	8.94572498	3.70E-19	1.84E-18	Up	VASH2
KLRG2	41.5707997	3.33328573	0.37270541	8.9434863	3.77E-19	1.88E-18	Up	KLRG2
SMCR8	1159.0118	-0.67658338	0.07567571	-8.94056201	3.87E-19	1.93E-18	Down	SMCR8
ANKRD10	3669.29507	0.86263254	0.09649903	8.93928677	3.92E-19	1.95E-18	Up	ANKRD10
MAPK7	642.718118	-0.74186446	0.08299619	-8.93853593	3.94E-19	1.96E-18	Down	MAPK7
PRKCE	335.221279	-0.77261965	0.08644206	-8.93800587	3.96E-19	1.97E-18	Down	PRKCE
ROPN1	2.48614064	-3.26795297	0.36567394	-8.93679479	4.01E-19	1.99E-18	Down	ROPN1
ATP6V0D1	4588.93728	-0.84830807	0.09492376	-8.93673012	4.01E-19	1.99E-18	Down	ATP6V0D1
PMFBP1	126.605524	1.52661608	0.17085193	8.93531651	4.06E-19	2.02E-18	Up	PMFBP1
DYNC1I1	59.6772856	-2.17030452	0.24292563	-8.93402848	4.11E-19	2.04E-18	Down	DYNC1I1
KCTD13	653.899128	0.76932104	0.08613638	8.93142956	4.21E-19	2.09E-18	Up	KCTD13
DLX3	67.0448915	3.23628147	0.36236735	8.93094118	4.22E-19	2.10E-18	Up	DLX3
C22orf23	30.3669764	-1.11881988	0.1252762	-8.9308258	4.23E-19	2.10E-18	Down	C22orf23
MCOLN2	382.200626	-1.75059589	0.19605867	-8.92893881	4.30E-19	2.13E-18	Down	MCOLN2
HPRT1	1627.65082	0.763253	0.08550881	8.92601644	4.42E-19	2.19E-18	Up	HPRT1
TMEM25	157.563049	-1.47203313	0.16494605	-8.92433084	4.48E-19	2.22E-18	Down	TMEM25
KIAA0408	2.27724277	-3.31465731	0.37156938	-8.92069564	4.63E-19	2.30E-18	Down	KIAA0408
GAPT	32.7232523	-1.81829496	0.20384061	-8.92018001	4.66E-19	2.31E-18	Down	GAPT
PNPO	1242.44379	0.679391	0.07618225	8.91796947	4.75E-19	2.35E-18	Up	PNPO
LGR6	925.644803	2.10141508	0.2356765	8.91652357	4.81E-19	2.38E-18	Up	LGR6
CEP78	781.028491	0.7729342	0.08669798	8.91525001	4.87E-19	2.41E-18	Up	CEP78
SIPA1L3	2967.43426	-0.7492929	0.08404972	-8.91487639	4.88E-19	2.42E-18	Down	SIPA1L3
TUBG1	1700.08949	0.84388757	0.0946648	8.91448122	4.90E-19	2.43E-18	Up	TUBG1

CSPG4	838.868256	-1.37668699	0.15448892	-8.91123457	5.05E-19	2.50E-18	Down	CSPG4
NUTM2D	15.1437851	-1.18373451	0.1328568	-8.90985283	5.11E-19	2.53E-18	Down	NUTM2D
HENMT1	610.628859	1.18219377	0.13269651	8.90900418	5.15E-19	2.55E-18	Up	HENMT1
AMBP	52.4733022	2.13989446	0.24022274	8.90795965	5.20E-19	2.57E-18	Up	AMBP
FXYD4	18.5514289	2.53997752	0.28517927	8.9065995	5.26E-19	2.60E-18	Up	FXYD4
METTL5	1202.94432	0.60572628	0.06802945	8.90388385	5.39E-19	2.66E-18	Up	METTL5
SLC4A2	4228.31531	0.78067989	0.08768042	8.9036967	5.40E-19	2.67E-18	Up	SLC4A2
MTPAP	969.725119	0.57775732	0.06489486	8.90297565	5.44E-19	2.68E-18	Up	MTPAP
OR51E2	51.1505258	-1.84302157	0.20701707	-8.90275154	5.45E-19	2.69E-18	Down	OR51E2
CAMK2N1	4977.49518	-1.07256699	0.12052091	-8.89942632	5.61E-19	2.77E-18	Down	CAMK2N1
GPRASP2	160.846332	-0.89211097	0.10028391	-8.89585361	5.80E-19	2.86E-18	Down	GPRASP2
MSH2	1350.95594	0.68762822	0.07730068	8.89550062	5.82E-19	2.87E-18	Up	MSH2
SH2D1B	14.8113863	-1.75594965	0.19752821	-8.88961473	6.13E-19	3.02E-18	Down	SH2D1B
CDC42EP3	930.491648	-1.0191896	0.11466666	-8.88828189	6.21E-19	3.06E-18	Down	CDC42EP3
HCRT	4.54217503	3.45196427	0.38837421	8.88824288	6.21E-19	3.06E-18	Up	HCRT
HMGB3	2466.79358	0.76826496	0.08644177	8.88765851	6.24E-19	3.08E-18	Up	HMGB3
ENTPD8	944.397979	-2.15786441	0.24280302	-8.88730455	6.26E-19	3.08E-18	Down	ENTPD8
SUGP2	1475.6159	0.6593671	0.07419877	8.88649647	6.31E-19	3.11E-18	Up	SUGP2
NCR2	1.30966432	-2.7901278	0.31400229	-8.88569239	6.35E-19	3.13E-18	Down	NCR2
CREG2	43.0981237	2.48078664	0.27920255	8.88525784	6.38E-19	3.14E-18	Up	CREG2
KIT	333.276331	-1.7191098	0.19353379	-8.88273722	6.52E-19	3.21E-18	Down	KIT
CPA3	304.112031	-1.9057661	0.2145988	-8.88060005	6.65E-19	3.27E-18	Down	CPA3
CHRNA3	89.1373489	-2.05434206	0.23134682	-8.87992361	6.69E-19	3.29E-18	Down	CHRNA3
SERTAD4	164.954674	-1.72059092	0.19377316	-8.87940765	6.72E-19	3.30E-18	Down	SERTAD4
SUCLG1	4902.1855	-0.73030725	0.08228542	-8.87529301	6.97E-19	3.43E-18	Down	SUCLG1
DOLPP1	1008.31313	-0.8060158	0.09083868	-8.87304615	7.12E-19	3.50E-18	Down	DOLPP1
FAAH2	363.107837	0.83759931	0.09441521	8.87144481	7.22E-19	3.55E-18	Up	FAAH2
RSPH14	22.0054281	1.62327729	0.18307496	8.86673583	7.53E-19	3.70E-18	Up	RSPH14
KIAA2012	10.9594225	1.98695794	0.22412256	8.86549707	7.62E-19	3.74E-18	Up	KIAA2012
ZWINT	1785.08907	0.75650317	0.08534108	8.86446727	7.69E-19	3.77E-18	Up	ZWINT
ZNF514	538.261441	0.92121186	0.10395445	8.86168744	7.88E-19	3.87E-18	Up	ZNF514
MEGF6	690.734976	1.60914768	0.18165493	8.85826569	8.13E-19	3.99E-18	Up	MEGF6
IFFO2	839.372014	0.94001559	0.10613706	8.85661962	8.25E-19	4.04E-18	Up	IFFO2
GPIHBP1	21.4168763	-2.07442804	0.23425902	-8.85527488	8.35E-19	4.09E-18	Down	GPIHBP1
DSCAML1	29.1410393	-2.29851571	0.25970366	-8.85053269	8.71E-19	4.27E-18	Down	DSCAML1
BEAN1	44.5757131	2.17853672	0.24616378	8.84994823	8.76E-19	4.29E-18	Up	BEAN1
MTF1	714.068783	-0.65530943	0.07407316	-8.84678633	9.01E-19	4.41E-18	Down	MTF1
LY6H	19.9086996	-2.13869204	0.2418015	-8.84482525	9.17E-19	4.49E-18	Down	LY6H
ELK1	1693.13273	0.57226917	0.06474312	8.83907262	9.65E-19	4.73E-18	Up	ELK1
ASCC3	1903.40491	0.67021162	0.07583666	8.83756768	9.78E-19	4.79E-18	Up	ASCC3
SLAIN2	1764.59929	-0.55529231	0.06284723	-8.83558909	9.96E-19	4.87E-18	Down	SLAIN2
FLAD1	1750.66841	0.74054785	0.08383673	8.83321534	1.02E-18	4.98E-18	Up	FLAD1
MAP4K1	171.76442	-1.37696923	0.15590874	-8.8318927	1.03E-18	5.03E-18	Down	MAP4K1
KRI1	1106.60084	0.65099718	0.07373054	8.82940971	1.05E-18	5.15E-18	Up	KRI1
SBK1	245.995378	1.88239544	0.21321456	8.82864394	1.06E-18	5.18E-18	Up	SBK1
UBAC2	4353.76794	0.73214455	0.08296358	8.82489103	1.10E-18	5.35E-18	Up	UBAC2
SNTG2	3.54929888	-2.41119973	0.27324253	-8.82439387	1.10E-18	5.38E-18	Down	SNTG2
USP51	30.8218167	-1.3468197	0.15263706	-8.82367437	1.11E-18	5.41E-18	Down	USP51
G3BP1	7062.21302	0.58589339	0.06640403	8.8231601	1.11E-18	5.43E-18	Up	G3BP1
PDE7A	817.979398	0.82594898	0.09361284	8.82303094	1.11E-18	5.44E-18	Up	PDE7A
MPDZ	251.318084	-1.60699125	0.18215667	-8.82202824	1.12E-18	5.48E-18	Down	MPDZ
SMARCD2	5656.97883	0.61343333	0.06954432	8.82075381	1.14E-18	5.55E-18	Up	SMARCD2
FAXC	68.4257424	-1.93362793	0.21921862	-8.82054613	1.14E-18	5.55E-18	Down	FAXC
ZMAT4	3.13604809	-3.06196083	0.34716379	-8.81993157	1.15E-18	5.58E-18	Down	ZMAT4
TCEAL3	221.001942	-1.19378918	0.13536803	-8.81884107	1.16E-18	5.64E-18	Down	TCEAL3
SLC15A2	103.708314	-1.06604502	0.12092501	-8.81575271	1.19E-18	5.79E-18	Down	SLC15A2
RP1L1	10.2548894	2.20118319	0.24970512	8.81513046	1.20E-18	5.82E-18	Up	RP1L1
MBTPS2	869.139623	0.75850633	0.08604628	8.81509719	1.20E-18	5.82E-18	Up	MBTPS2

TTLL9	10.5518226	1.89520252	0.21500127	8.81484335	1.20E-18	5.83E-18	Up	TTLL9
TCERG1	1932.82305	0.64842833	0.07357076	8.81366878	1.21E-18	5.89E-18	Up	TCERG1
FBXO2	339.238226	2.41120565	0.27358833	8.81326209	1.22E-18	5.91E-18	Up	FBXO2
TMEM198	171.830097	1.4186091	0.16096915	8.81292535	1.22E-18	5.93E-18	Up	TMEM198
CLPSL2	1.93878598	3.78736078	0.42984088	8.8110763	1.24E-18	6.03E-18	Up	CLPSL2
ACTL6B	1.85589381	-2.78266003	0.31583915	-8.81037077	1.25E-18	6.06E-18	Down	ACTL6B
ASAP2	999.154309	-0.73807207	0.08379179	-8.80840515	1.27E-18	6.17E-18	Down	ASAP2
EME2	472.178661	1.11649166	0.12675843	8.80802693	1.27E-18	6.19E-18	Up	EME2
GPD2	2136.81429	-0.64634538	0.07338309	-8.807825	1.28E-18	6.20E-18	Down	GPD2
MVP	15312.2485	-0.83125571	0.09437906	-8.80762887	1.28E-18	6.21E-18	Down	MVP
SRL	24.5923097	-1.58782177	0.1802786	-8.80759963	1.28E-18	6.21E-18	Down	SRL
GOT1	2354.14112	-0.76330691	0.08666704	-8.80734903	1.28E-18	6.22E-18	Down	GOT1
GPR119	1.27818359	-3.78773198	0.43007906	-8.80705973	1.28E-18	6.23E-18	Down	GPR119
METTL2B	969.313146	0.57957443	0.06582006	8.80543699	1.30E-18	6.32E-18	Up	METTL2B
SPARC	30927.3858	1.35782113	0.15420341	8.80538952	1.30E-18	6.32E-18	Up	SPARC
MACROD1	1622.4688	1.26697579	0.14390942	8.80398114	1.32E-18	6.40E-18	Up	MACROD1
GPR146	26.829619	-1.23985385	0.14083278	-8.80373037	1.32E-18	6.41E-18	Down	GPR146
STOM	2694.72235	-1.13556017	0.12899996	-8.802795	1.33E-18	6.47E-18	Down	STOM
SLCO1A2	12.9020894	3.88267601	0.44114431	8.80137397	1.35E-18	6.55E-18	Up	SLCO1A2
MSTN	7.51190004	-2.04131426	0.23194229	-8.80095742	1.36E-18	6.57E-18	Down	MSTN
KCNJ15	67.6059451	2.24516835	0.25512337	8.80032412	1.36E-18	6.60E-18	Up	KCNJ15
IL1RN	698.228805	1.88975374	0.21478883	8.79819368	1.39E-18	6.73E-18	Up	IL1RN
GJC1	217.718205	-1.41264711	0.16056371	-8.79804748	1.39E-18	6.73E-18	Down	GJC1
TCP11	21.9039697	3.87346122	0.44026828	8.79795666	1.39E-18	6.74E-18	Up	TCP11
MARK2	3145.84208	-0.55469503	0.0630492	-8.79781216	1.40E-18	6.75E-18	Down	MARK2
MAP2K4	863.265145	-0.64472845	0.07328737	-8.79726569	1.40E-18	6.78E-18	Down	MAP2K4
CPNE9	20.4301086	1.85752713	0.21118641	8.79567569	1.42E-18	6.87E-18	Up	CPNE9
IGFBP1	24.2890996	3.41883438	0.38870257	8.79550244	1.42E-18	6.88E-18	Up	IGFBP1
ANKRD65	64.1842509	-1.59605077	0.18146737	-8.79524928	1.43E-18	6.89E-18	Down	ANKRD65
FZD3	552.624992	1.35260347	0.15378882	8.79520028	1.43E-18	6.89E-18	Up	FZD3
C4orf48	673.994926	2.0770235	0.23627577	8.79067489	1.49E-18	7.18E-18	Up	C4orf48
RBMS3	147.01811	-1.57475216	0.17920962	-8.78720764	1.53E-18	7.40E-18	Down	RBMS3
IRF2	1418.22979	-0.51601319	0.05873405	-8.78558824	1.56E-18	7.50E-18	Down	IRF2
NUDT9	904.32287	-0.52221647	0.05944685	-8.78459469	1.57E-18	7.57E-18	Down	NUDT9
C1orf226	784.123338	-0.7822294	0.08905576	-8.78359139	1.58E-18	7.63E-18	Down	C1orf226
NUDT19	1124.42746	0.66319505	0.07550516	8.78344021	1.59E-18	7.64E-18	Up	NUDT19
FGGY	1355.39049	1.94641686	0.22163053	8.78225957	1.60E-18	7.72E-18	Up	FGGY
ARL6IP5	4103.04258	-0.6247181	0.07114211	-8.78126997	1.62E-18	7.79E-18	Down	ARL6IP5
AVPI1	973.974739	-0.87963529	0.10017332	-8.78113317	1.62E-18	7.80E-18	Down	AVPI1
SNAP25	31.3428394	-2.61436247	0.29775794	-8.78016042	1.63E-18	7.86E-18	Down	SNAP25
STAMBPL1	878.847923	0.70411884	0.08021169	8.77825671	1.66E-18	7.99E-18	Up	STAMBPL1
TCF7L2	2031.74939	-0.71858412	0.08186354	-8.77782875	1.67E-18	8.02E-18	Down	TCF7L2
CTSV	561.046171	1.47434831	0.16797403	8.77723942	1.68E-18	8.06E-18	Up	CTSV
SPOCK3	7.68071989	-3.80714901	0.43379676	-8.77634268	1.69E-18	8.12E-18	Down	SPOCK3
KTN1	6225.84174	-0.76786598	0.08751139	-8.77446934	1.72E-18	8.26E-18	Down	KTN1
REXO2	1786.91494	0.66990771	0.0763575	8.77330547	1.73E-18	8.34E-18	Up	REXO2
NEDD9	2333.49044	-1.01749709	0.1159932	-8.77204078	1.75E-18	8.43E-18	Down	NEDD9
CMKLR1	299.145467	-1.41504219	0.16131799	-8.77175694	1.76E-18	8.45E-18	Down	CMKLR1
SV2B	35.5319452	-2.04098396	0.23267957	-8.77165086	1.76E-18	8.46E-18	Down	SV2B
SLC38A3	44.5838568	2.90115307	0.33076975	8.77091404	1.77E-18	8.51E-18	Up	SLC38A3
PRSS36	98.2536451	-1.14832201	0.13094829	-8.76927815	1.80E-18	8.63E-18	Down	PRSS36
CSAG1	29.6190196	6.3318044	0.72210675	8.76851578	1.81E-18	8.69E-18	Up	CSAG1
NXPE3	244.856491	-1.01467701	0.1157303	-8.76760061	1.83E-18	8.76E-18	Down	NXPE3
SPACA3	37.1431206	2.78792406	0.31801739	8.76657726	1.84E-18	8.84E-18	Up	SPACA3
TMBIM4	1264.5987	-0.84269364	0.09615582	-8.76383414	1.89E-18	9.05E-18	Down	TMBIM4
RNASEH1	650.649507	0.50211738	0.0572973	8.76336962	1.89E-18	9.09E-18	Up	RNASEH1
MS4A1	130.345885	-2.75351939	0.31422355	-8.76293136	1.90E-18	9.12E-18	Down	MS4A1
CDK10	2465.17961	0.91837394	0.10481445	8.76190228	1.92E-18	9.20E-18	Up	CDK10

MB	131.215052	-2.13284872	0.24355655	-8.75709862	2.00E-18	9.60E-18	Down	MB
GCG	268.958928	-4.42024821	0.50497182	-8.75345529	2.07E-18	9.91E-18	Down	GCG
IL10RA	545.26607	-1.37698031	0.15731141	-8.75321324	2.07E-18	9.93E-18	Down	IL10RA
CIDEC	120.349481	-2.23149663	0.25494574	-8.75282965	2.08E-18	9.96E-18	Down	CIDEC
IPO7	7063.1627	0.65278628	0.07459271	8.75134153	2.11E-18	1.01E-17	Up	IPO7
KLLN	47.9576397	-0.7825967	0.0894347	-8.75048181	2.12E-18	1.02E-17	Down	KLLN
TMEM52	219.287588	1.49189272	0.17053843	8.74813236	2.17E-18	1.04E-17	Up	TMEM52
CEP131	619.242046	0.87682262	0.10024704	8.74661876	2.20E-18	1.05E-17	Up	CEP131
CXCL16	3393.64283	0.9103552	0.10408132	8.74657598	2.20E-18	1.05E-17	Up	CXCL16
PANK3	2853.34789	-0.96141244	0.10992955	-8.7457143	2.22E-18	1.06E-17	Down	PANK3
IMP4	3058.61044	0.69238041	0.07917223	8.74524294	2.23E-18	1.06E-17	Up	IMP4
PRRT1	79.9294562	-1.05565855	0.12073375	-8.74369045	2.26E-18	1.08E-17	Down	PRRT1
ADH5	4725.63632	-0.65805265	0.07526983	-8.74258219	2.28E-18	1.09E-17	Down	ADH5
SYNDIG1L	3.43447431	-2.26727721	0.25933942	-8.74250896	2.28E-18	1.09E-17	Down	SYNDIG1L
TRMT2A	1101.65422	0.81225348	0.09291151	8.74222654	2.29E-18	1.09E-17	Up	TRMT2A
MS4A4A	254.68326	-1.5966604	0.182723	-8.73814704	2.37E-18	1.13E-17	Down	MS4A4A
FBXO45	1058.35279	0.59606053	0.06821615	8.73782194	2.38E-18	1.13E-17	Up	FBXO45
MIF	2114.47218	1.37908694	0.15784725	8.73684506	2.40E-18	1.14E-17	Up	MIF
MOGS	3128.37399	0.57615717	0.06595532	8.73556787	2.42E-18	1.16E-17	Up	MOGS
LIMK2	2771.40788	-0.54925611	0.06287605	-8.73553845	2.42E-18	1.16E-17	Down	LIMK2
TM9SF4	6381.72034	0.73406149	0.08405194	8.73342677	2.47E-18	1.18E-17	Up	TM9SF4
MGP	2270.59153	-1.86111802	0.21315138	-8.73143778	2.51E-18	1.20E-17	Down	MGP
HDAC4	892.728395	-0.7049257	0.08074333	-8.73045093	2.54E-18	1.21E-17	Down	HDAC4
LYPD3	145.285039	1.82565239	0.20919509	8.72703272	2.61E-18	1.24E-17	Up	LYPD3
SIGLEC1	302.973249	-1.71601384	0.19668192	-8.72481727	2.67E-18	1.27E-17	Down	SIGLEC1
GPRIN2	318.887207	-1.36219454	0.15625641	-8.71768734	2.84E-18	1.35E-17	Down	GPRIN2
TAF1C	1208.59978	0.76989871	0.0883531	8.71388466	2.94E-18	1.40E-17	Up	TAF1C
OSBP2	370.796963	0.82241429	0.09438095	8.71377382	2.94E-18	1.40E-17	Up	OSBP2
RAB23	465.530212	-1.09099503	0.12521193	-8.71318725	2.95E-18	1.40E-17	Down	RAB23
RHBDF1	1767.32346	0.91576008	0.10510357	8.71293044	2.96E-18	1.41E-17	Up	RHBDF1
SPAG17	10.4734377	2.77845767	0.31889395	8.71279518	2.96E-18	1.41E-17	Up	SPAG17
CCDC77	296.23475	0.67363297	0.07733396	8.71070034	3.02E-18	1.43E-17	Up	CCDC77
MXRA7	1487.37532	-1.19492066	0.13718708	-8.71015448	3.03E-18	1.44E-17	Down	MXRA7
DNAH12	12.3298463	2.06551037	0.23714225	8.7100059	3.04E-18	1.44E-17	Up	DNAH12
SLC35A3	2958.97776	-0.87729856	0.10073432	-8.70903356	3.06E-18	1.45E-17	Down	SLC35A3
SSB	3571.65444	0.82372816	0.09459468	8.7079759	3.09E-18	1.47E-17	Up	SSB
PORCN	337.915236	0.91103708	0.10463538	8.70677821	3.13E-18	1.48E-17	Up	PORCN
AGL	947.794547	-0.82246426	0.09446952	-8.706134	3.14E-18	1.49E-17	Down	AGL
CCL13	101.307622	-2.39390912	0.27498704	-8.70553422	3.16E-18	1.50E-17	Down	CCL13
CACFD1	1723.98358	-0.93158184	0.1070184	-8.70487537	3.18E-18	1.51E-17	Down	CACFD1
NKX2-1	22.9222183	6.05007405	0.69506049	8.70438492	3.19E-18	1.51E-17	Up	NKX2-1
NMD3	1875.45327	0.6426111	0.07384587	8.70205922	3.26E-18	1.54E-17	Up	NMD3
BBC3	802.656708	0.97380532	0.1119112	8.70158926	3.27E-18	1.55E-17	Up	BBC3
LEPROT	2591.57418	-0.62034342	0.07130406	-8.69997388	3.32E-18	1.57E-17	Down	LEPROT
DUSP7	798.424817	0.66564528	0.07651976	8.69899846	3.35E-18	1.58E-17	Up	DUSP7
WWP2	3073.92482	-0.52941622	0.06086335	-8.69844063	3.36E-18	1.59E-17	Down	WWP2
G6PC3	2452.99538	0.81496441	0.09370005	8.69758812	3.39E-18	1.60E-17	Up	G6PC3
GRM8	533.942666	2.18140342	0.25081324	8.69732154	3.40E-18	1.61E-17	Up	GRM8
MICU3	28.7017663	-1.82776331	0.2101696	-8.69661123	3.42E-18	1.62E-17	Down	MICU3
DPF1	23.031279	1.5680855	0.18032248	8.69600679	3.44E-18	1.62E-17	Up	DPF1
PCSK6	759.414525	-0.99111099	0.11399765	-8.69413515	3.49E-18	1.65E-17	Down	PCSK6
INPP5D	2178.8847	1.06948357	0.12303428	8.69256561	3.54E-18	1.67E-17	Up	INPP5D
INPP5E	596.912762	0.773318	0.08897152	8.69174815	3.57E-18	1.69E-17	Up	INPP5E
NXPE1	707.312234	-2.25192943	0.25911376	-8.69089109	3.60E-18	1.70E-17	Down	NXPE1
C19orf53	2950.32462	0.82129541	0.09451128	8.68991972	3.63E-18	1.71E-17	Up	C19orf53
KRT31	5.86714307	5.56933431	0.6409317	8.6894349	3.64E-18	1.72E-17	Up	KRT31
GPR34	154.290871	-1.60415868	0.18465717	-8.68722649	3.71E-18	1.75E-17	Down	GPR34
HSF2BP	17.7322543	1.22106995	0.14058144	8.68585464	3.76E-18	1.77E-17	Up	HSF2BP

ACACA	2698.66826	0.73428866	0.08455605	8.68404649	3.82E-18	1.80E-17	Up	ACACA
CCM2L	53.2765188	-1.04846254	0.12074085	-8.68357769	3.84E-18	1.81E-17	Down	CCM2L
FEM1B	1822.94192	-0.63605936	0.07324997	-8.68340751	3.84E-18	1.81E-17	Down	FEM1B
ZSWIM1	782.749106	0.80022251	0.0921573	8.68322458	3.85E-18	1.81E-17	Up	ZSWIM1
PADI1	89.7794296	2.10168632	0.24204556	8.68301935	3.85E-18	1.82E-17	Up	PADI1
SFRP5	30.3761982	-3.47726594	0.4005028	-8.6822512	3.88E-18	1.83E-17	Down	SFRP5
CHRM4	10.7456596	-2.11489342	0.24363887	-8.6804433	3.94E-18	1.86E-17	Down	CHRM4
TOMM40	3784.12707	0.82733421	0.09531342	8.68014418	3.95E-18	1.86E-17	Up	TOMM40
CLEC17A	10.9006087	-2.38457833	0.27472843	-8.67976536	3.97E-18	1.87E-17	Down	CLEC17A
FLT3LG	34.649009	-0.99015123	0.11408932	-8.67873729	4.00E-18	1.88E-17	Down	FLT3LG
CASP10	1338.97638	-0.84543736	0.09741672	-8.6785656	4.01E-18	1.88E-17	Down	CASP10
SGCB	1247.86053	-0.84833212	0.09775915	-8.67777701	4.04E-18	1.90E-17	Down	SGCB
PPM1J	55.4220803	1.09896672	0.12664213	8.67773402	4.04E-18	1.90E-17	Up	PPM1J
PTPN12	2602.14753	0.72591563	0.08365586	8.67740354	4.05E-18	1.90E-17	Up	PTPN12
CHRNB4	14.3152345	-1.69328142	0.19515012	-8.67681472	4.07E-18	1.91E-17	Down	CHRNB4
RNF5	1752.53623	-0.61088614	0.07041286	-8.67577502	4.11E-18	1.93E-17	Down	RNF5
PHF6	1356.06691	0.81740884	0.09422081	8.67545965	4.12E-18	1.93E-17	Up	PHF6
ZNF626	37.6627049	-1.57962251	0.18210725	-8.67413308	4.17E-18	1.96E-17	Down	ZNF626
TUBA1A	2930.0486	-1.27268873	0.14679119	-8.6700622	4.32E-18	2.03E-17	Down	TUBA1A
ARHGEF38	400.018578	1.05023991	0.12114126	8.66954779	4.34E-18	2.04E-17	Up	ARHGEF38
SEMA4D	1629.63957	0.58729794	0.06774707	8.66897911	4.36E-18	2.04E-17	Up	SEMA4D
MAPK3	5072.07179	-0.9232784	0.10653091	-8.66676519	4.45E-18	2.08E-17	Down	MAPK3
TBC1D4	2077.01396	0.90445169	0.10436732	8.66604344	4.47E-18	2.10E-17	Up	TBC1D4
PODXL	2886.21469	0.93371323	0.10775178	8.66540882	4.50E-18	2.11E-17	Up	PODXL
ME2	1958.38281	-0.87429272	0.10091103	-8.66399526	4.56E-18	2.13E-17	Down	ME2
MMP14	9546.94966	1.15979189	0.13389956	8.66165553	4.65E-18	2.18E-17	Up	MMP14
NAA20	2279.65496	0.8107656	0.09360656	8.66141838	4.66E-18	2.18E-17	Up	NAA20
FGF2	146.872157	-1.74744773	0.20175701	-8.66114998	4.67E-18	2.19E-17	Down	FGF2
CCL24	1300.77108	1.85855597	0.21460249	8.66045843	4.70E-18	2.20E-17	Up	CCL24
AGK	1034.59733	0.58361034	0.06739278	8.65983441	4.72E-18	2.21E-17	Up	AGK
DERA	1574.84556	-0.698085	0.08063044	-8.65783411	4.81E-18	2.25E-17	Down	DERA
PRR13	4947.16914	-0.65008892	0.07509338	-8.65707335	4.84E-18	2.26E-17	Down	PRR13
TIGD2	363.585599	0.65372059	0.07553415	8.65463621	4.94E-18	2.31E-17	Up	TIGD2
EPHB2	9354.96439	1.07221457	0.12389076	8.65451586	4.95E-18	2.31E-17	Up	EPHB2
FAM102A	3657.10519	-0.73791376	0.08527669	-8.65317049	5.01E-18	2.34E-17	Down	FAM102A
F2R	1333.25294	0.99935903	0.11549122	8.65311712	5.01E-18	2.34E-17	Up	F2R
CD302	164.438902	-1.30330441	0.15066993	-8.65006291	5.15E-18	2.40E-17	Down	CD302
NARS2	934.23281	0.63234985	0.07310384	8.65002233	5.15E-18	2.40E-17	Up	NARS2
IKZF1	333.955721	-1.50692898	0.17427384	-8.64690303	5.29E-18	2.47E-17	Down	IKZF1
PDK2	1070.08031	-0.95975087	0.11099911	-8.64647362	5.31E-18	2.48E-17	Down	PDK2
PCDHGA1	42.5025428	1.47753927	0.17090082	8.64559511	5.35E-18	2.50E-17	Up	PCDHGA1
RMND5A	2945.92056	-0.60700966	0.07021201	-8.64538226	5.36E-18	2.50E-17	Down	RMND5A
NMRAL1	1629.85252	0.86860645	0.10049091	8.64363233	5.45E-18	2.54E-17	Up	NMRAL1
GOLT1A	435.021679	1.25006664	0.14465254	8.64185771	5.53E-18	2.58E-17	Up	GOLT1A
PRKG1	328.08258	-1.30775282	0.15134572	-8.64083124	5.58E-18	2.60E-17	Down	PRKG1
C1orf21	1857.86458	-1.06064351	0.12275652	-8.64022132	5.61E-18	2.61E-17	Down	C1orf21
WSCD2	20.0051618	-2.67521539	0.30963514	-8.63989592	5.63E-18	2.62E-17	Down	WSCD2
PINLYP	26.9101061	-1.16038369	0.13430675	-8.63980186	5.63E-18	2.62E-17	Down	PINLYP
TMEM216	608.537006	0.69025621	0.07990189	8.63879653	5.68E-18	2.64E-17	Up	TMEM216
KRTAP3-1	4.20974115	4.91506263	0.56898184	8.63834706	5.70E-18	2.65E-17	Up	KRTAP3-1
TMEM164	3104.28719	-0.67252527	0.07785514	-8.63816169	5.71E-18	2.66E-17	Down	TMEM164
RPRD1B	1969.02028	0.63535535	0.07356178	8.63703023	5.77E-18	2.68E-17	Up	RPRD1B
TSPAN18	514.739609	-1.44812163	0.16768643	-8.6358906	5.83E-18	2.71E-17	Down	TSPAN18
SLC15A1	227.085428	-2.61845106	0.30321233	-8.635701	5.84E-18	2.71E-17	Down	SLC15A1
CYP4F8	12.7289068	2.7122562	0.31410575	8.63485043	5.88E-18	2.73E-17	Up	CYP4F8
VPS13D	1916.42862	-0.8436856	0.09770773	-8.63478826	5.88E-18	2.73E-17	Down	VPS13D
SRRT	4450.76403	0.55485943	0.0642609	8.63447968	5.90E-18	2.74E-17	Up	SRRT
AKAP5	211.767623	-1.42747825	0.16535431	-8.63284571	5.98E-18	2.78E-17	Down	AKAP5

LILRA4	9.21444249	-2.01362464	0.23325648	-8.63266318	5.99E-18	2.78E-17	Down	LILRA4
MAFA	3.59394037	-2.04233944	0.23665664	-8.62996892	6.14E-18	2.85E-17	Down	MAFA
SPINK5	211.728137	-1.85659149	0.21514094	-8.62965238	6.15E-18	2.86E-17	Down	SPINK5
AAAS	1659.16721	0.58355434	0.06763841	8.62755915	6.27E-18	2.91E-17	Up	AAAS
NDRG4	160.243701	-1.17431378	0.13613436	-8.62613825	6.35E-18	2.94E-17	Down	NDRG4
LPCAT4	2577.52687	-0.84431009	0.09788427	-8.62559555	6.38E-18	2.96E-17	Down	LPCAT4
ATP8A1	1190.40428	-1.30606078	0.15144192	-8.62416922	6.46E-18	2.99E-17	Down	ATP8A1
LRRC9	7.69047958	2.39253379	0.27750461	8.6216002	6.60E-18	3.06E-17	Up	LRRC9
PUSL1	584.393001	1.0400195	0.12063488	8.62121719	6.62E-18	3.07E-17	Up	PUSL1
TNFRSF10A	722.692184	0.86480761	0.10031591	8.6208424	6.65E-18	3.08E-17	Up	TNFRSF10A
PRPF6	6959.9831	0.89174867	0.10344245	8.62072279	6.65E-18	3.08E-17	Up	PRPF6
S1PR1	405.142943	-1.11054818	0.12882316	-8.62071849	6.65E-18	3.08E-17	Down	S1PR1
CCDC59	930.357196	0.68192788	0.07910963	8.62003645	6.69E-18	3.10E-17	Up	CCDC59
PKD1L2	9.18448826	-2.14189468	0.24853895	-8.6179436	6.82E-18	3.15E-17	Down	PKD1L2
MAP2K6	794.896739	-0.96646099	0.11220084	-8.61367013	7.08E-18	3.27E-17	Down	MAP2K6
TMEM79	259.660918	0.7356196	0.0854083	8.61297515	7.12E-18	3.29E-17	Up	TMEM79
TMC4	3799.50744	-0.88395993	0.10264464	-8.61184674	7.19E-18	3.32E-17	Down	TMC4
SPRED3	48.8636525	1.40090121	0.16270805	8.60990708	7.31E-18	3.38E-17	Up	SPRED3
TMEM160	565.646082	1.88496847	0.21893658	8.60965512	7.33E-18	3.39E-17	Up	TMEM160
FOXO4	906.254766	-0.84723068	0.09842346	-8.60801577	7.43E-18	3.43E-17	Down	FOXO4
SLITRK2	4.65734222	-2.65284912	0.30820498	-8.607418	7.47E-18	3.45E-17	Down	SLITRK2
ACYP1	271.828606	0.73620445	0.08553263	8.60729316	7.48E-18	3.45E-17	Up	ACYP1
LRWD1	896.055741	0.84133693	0.09774905	8.60711107	7.49E-18	3.46E-17	Up	LRWD1
LBP	23.4753142	2.70980888	0.31490548	8.60514993	7.62E-18	3.52E-17	Up	LBP
SH3KBP1	2865.91786	-0.77345489	0.08989326	-8.6041476	7.69E-18	3.55E-17	Down	SH3KBP1
PRNP	2327.03635	-1.10170029	0.12805914	-8.60305843	7.76E-18	3.58E-17	Down	PRNP
MISP	13794.0106	-0.96210651	0.11183992	-8.60253208	7.80E-18	3.60E-17	Down	MISP
STRAP	4937.04085	0.60691702	0.07055186	8.60242407	7.80E-18	3.60E-17	Up	STRAP
PPIH	1265.11025	0.77773297	0.09043742	8.59968069	7.99E-18	3.68E-17	Up	PPIH
COL19A1	4.95402942	-2.73334456	0.31799923	-8.59544407	8.29E-18	3.82E-17	Down	COL19A1
AGAP6	204.042255	0.938195	0.10916191	8.59452703	8.36E-18	3.85E-17	Up	AGAP6
RASSF6	843.735505	-1.10229017	0.12825953	-8.59421628	8.38E-18	3.86E-17	Down	RASSF6
KIRREL3	22.93138	-1.88603013	0.21947517	-8.59336449	8.45E-18	3.89E-17	Down	KIRREL3
TPSB2	267.814047	-1.89634119	0.22070018	-8.59238618	8.52E-18	3.92E-17	Down	TPSB2
TANGO6	537.990437	0.53921749	0.06276031	8.59169581	8.57E-18	3.94E-17	Up	TANGO6
DKK1	62.7473285	2.9499026	0.3433979	8.59033372	8.67E-18	3.99E-17	Up	DKK1
FGG	11.3585202	5.62039988	0.65449447	8.58739092	8.90E-18	4.09E-17	Up	FGG
TCL1A	41.1808783	-3.31838707	0.3864399	-8.58707166	8.92E-18	4.10E-17	Down	TCL1A
NABP2	1825.98308	0.64376134	0.07496908	8.58702503	8.93E-18	4.10E-17	Up	NABP2
SLITRK3	13.89849	-3.54853695	0.41329589	-8.58594788	9.01E-18	4.14E-17	Down	SLITRK3
PRKAG2	889.746753	-0.64643719	0.07530628	-8.58410692	9.15E-18	4.20E-17	Down	PRKAG2
DPH6	313.904916	0.78578261	0.09153905	8.58412495	9.15E-18	4.20E-17	Up	DPH6
MGAT5	6519.18646	0.92296437	0.10751995	8.5841221	9.15E-18	4.20E-17	Up	MGAT5
DCLRE1B	465.118899	0.54653418	0.06368496	8.58184106	9.34E-18	4.29E-17	Up	DCLRE1B
AKAP12	1075.0188	-1.82518237	0.21269302	-8.58129878	9.38E-18	4.30E-17	Down	AKAP12
ROBO2	198.064554	1.92156468	0.22397729	8.57928347	9.55E-18	4.38E-17	Up	ROBO2
CEP95	908.060934	0.6932878	0.08081213	8.57900648	9.57E-18	4.39E-17	Up	CEP95
ZNF667	21.4527258	-1.45961119	0.17016121	-8.57781392	9.67E-18	4.43E-17	Down	ZNF667
RASSF2	489.01888	-1.25000832	0.14577848	-8.57471112	9.93E-18	4.55E-17	Down	RASSF2
LRRC14	1224.88223	0.80545945	0.09396024	8.57234356	1.01E-17	4.65E-17	Up	LRRC14
RNF24	881.319201	0.85850913	0.10017838	8.5698049	1.04E-17	4.75E-17	Up	RNF24
KCNK10	63.7461197	-1.77489306	0.20715771	-8.56783504	1.05E-17	4.83E-17	Down	KCNK10
CNGA1	122.423355	-1.54119202	0.17990687	-8.56661011	1.07E-17	4.88E-17	Down	CNGA1
SERF1B	12.8179128	-0.99991117	0.11673951	-8.56531906	1.08E-17	4.93E-17	Down	SERF1B
STAM2	1066.4586	-0.63774267	0.0744618	-8.56469603	1.08E-17	4.96E-17	Down	STAM2
SAA2	105.938697	2.49442806	0.29133618	8.56202638	1.11E-17	5.07E-17	Up	SAA2
ABLIM2	875.304929	1.15245374	0.13460243	8.56190909	1.11E-17	5.08E-17	Up	ABLIM2
DYNC1LI2	3145.95622	-0.64522034	0.07536612	-8.56114551	1.12E-17	5.11E-17	Down	DYNC1LI2

MIER2	730.452095	0.63901986	0.07465119	8.56007647	1.13E-17	5.16E-17	Up	MIER2
LRRC37A3	196.620463	1.24630862	0.14559977	8.5598255	1.13E-17	5.17E-17	Up	LRRC37A3
AFF1	2347.70177	-0.85238166	0.09959217	-8.55872179	1.14E-17	5.21E-17	Down	AFF1
CHMP2B	2535.2427	-0.67238478	0.07856428	-8.55840341	1.14E-17	5.23E-17	Down	CHMP2B
DPP7	3674.03603	1.06542949	0.12449127	8.55826694	1.15E-17	5.23E-17	Up	DPP7
ARHGAP25	234.145077	-1.1304584	0.13209509	-8.5579141	1.15E-17	5.25E-17	Down	ARHGAP25
TBX20	14.9627115	3.31868303	0.38784347	8.55675886	1.16E-17	5.30E-17	Up	TBX20
PCBP4	1004.62112	0.89917198	0.10511578	8.55411049	1.19E-17	5.42E-17	Up	PCBP4
SEMA3F	1479.69871	1.12075404	0.13102032	8.55404719	1.19E-17	5.42E-17	Up	SEMA3F
MEF2A	969.805525	-0.71470186	0.0836033	-8.54872784	1.24E-17	5.68E-17	Down	MEF2A
ADAMTSL2	339.559719	1.44873162	0.1694905	8.54756852	1.26E-17	5.73E-17	Up	ADAMTSL2
SULF1	6489.47885	1.73042444	0.20248666	8.54586866	1.28E-17	5.82E-17	Up	SULF1
PPP1R14D	1421.74294	-1.35460634	0.15852304	-8.54517009	1.28E-17	5.85E-17	Down	PPP1R14D
FAM220A	605.690496	0.54436726	0.06370736	8.54480986	1.29E-17	5.87E-17	Up	FAM220A
PRRG3	20.432458	-1.71706893	0.20098757	-8.54316002	1.31E-17	5.95E-17	Down	PRRG3
MOAP1	922.132392	-0.52549062	0.06151182	-8.54292086	1.31E-17	5.96E-17	Down	MOAP1
PTPRZ1	28.2951829	-2.97618455	0.34842684	-8.54177752	1.32E-17	6.02E-17	Down	PTPRZ1
ZNF749	139.247096	0.84655544	0.0991269	8.5401184	1.34E-17	6.10E-17	Up	ZNF749
RSPH3	600.252374	-0.54325124	0.06361463	-8.53972205	1.35E-17	6.12E-17	Down	RSPH3
MRPL30	1325.05098	0.54167117	0.06343006	8.53966108	1.35E-17	6.12E-17	Up	MRPL30
TSEN15	1052.28601	0.76742531	0.08987264	8.53903148	1.35E-17	6.16E-17	Up	TSEN15
PAPPA2	24.6457078	-2.39985616	0.28110725	-8.53715496	1.38E-17	6.25E-17	Down	PAPPA2
MYH4	11.0826074	2.70218182	0.31654762	8.53641495	1.38E-17	6.29E-17	Up	MYH4
BDH2	495.969089	-0.96133925	0.11262625	-8.5356587	1.39E-17	6.33E-17	Down	BDH2
SCO1	1427.5465	-0.60358348	0.07071646	-8.53526142	1.40E-17	6.35E-17	Down	SCO1
MPP4	3.52975956	2.41135404	0.28251885	8.5351969	1.40E-17	6.36E-17	Up	MPP4
LAMTOR3	1330.19238	-0.6386986	0.07483297	-8.53498945	1.40E-17	6.36E-17	Down	LAMTOR3
ZBTB33	1268.46512	0.78055244	0.09145891	8.53446039	1.41E-17	6.39E-17	Up	ZBTB33
MFSD10	3313.05664	0.89323437	0.10466619	8.53412543	1.41E-17	6.41E-17	Up	MFSD10
AQP9	200.507442	2.29940058	0.26944299	8.5339039	1.41E-17	6.42E-17	Up	AQP9
PECR	389.700577	-0.9042278	0.10596904	-8.53294293	1.43E-17	6.47E-17	Down	PECR
TPMT	2409.81157	-0.72823439	0.08534578	-8.53275263	1.43E-17	6.48E-17	Down	TPMT
SAAL1	545.596841	0.57684849	0.06762185	8.53050422	1.46E-17	6.60E-17	Up	SAAL1
BCL2L15	1580.77952	-1.19103048	0.13963118	-8.52983169	1.47E-17	6.64E-17	Down	BCL2L15
CDKN1A	5864.06633	-1.1281628	0.13229124	-8.52787215	1.49E-17	6.75E-17	Down	CDKN1A
SYNJ1	318.890336	-0.75430104	0.088472	-8.52587301	1.52E-17	6.87E-17	Down	SYNJ1
RNASEK	186.646369	-0.69288756	0.08126977	-8.52577214	1.52E-17	6.87E-17	Down	RNASEK
CCDC120	938.172932	0.73965725	0.08676852	8.52448895	1.53E-17	6.95E-17	Up	CCDC120
TLE4	465.724878	-0.97410259	0.11429112	-8.52299438	1.55E-17	7.04E-17	Down	TLE4
CSMD2	68.3323023	1.85205388	0.21730469	8.52284349	1.56E-17	7.04E-17	Up	CSMD2
SH2D6	39.664477	-2.15771438	0.25317906	-8.52248354	1.56E-17	7.06E-17	Down	SH2D6
GPCPD1	2704.35444	1.25150892	0.14687816	8.5207283	1.59E-17	7.17E-17	Up	GPCPD1
SERPINF2	236.021569	1.60908545	0.18889958	8.51820555	1.62E-17	7.32E-17	Up	SERPINF2
CLRN3	1801.29562	-0.92922549	0.10910168	-8.51705952	1.64E-17	7.40E-17	Down	CLRN3
TUBB2A	406.104446	-1.10539889	0.12978967	-8.51684829	1.64E-17	7.41E-17	Down	TUBB2A
OTULIN	970.912837	0.59525024	0.06989236	8.51667142	1.64E-17	7.42E-17	Up	OTULIN
FAM118B	926.214334	-0.692873	0.08135649	-8.51650556	1.64E-17	7.43E-17	Down	FAM118B
CKMT1B	621.402775	-1.10226146	0.12943585	-8.51589015	1.65E-17	7.46E-17	Down	CKMT1B
RAB9A	840.371821	-0.62750509	0.07371544	-8.5125322	1.70E-17	7.68E-17	Down	RAB9A
BAMBI	770.051408	1.74016765	0.20452172	8.50847357	1.76E-17	7.95E-17	Up	BAMBI
CST5	2.42390642	3.64737568	0.42869245	8.50814073	1.77E-17	7.97E-17	Up	CST5
SYT11	394.093888	-1.18963661	0.13989505	-8.50377926	1.84E-17	8.28E-17	Down	SYT11
GAST	3.09658587	3.95093921	0.46471942	8.50177343	1.87E-17	8.42E-17	Up	GAST
ASXL3	47.1692739	-2.05510396	0.24174917	-8.50097613	1.88E-17	8.47E-17	Down	ASXL3
MRI1	741.666393	0.73360997	0.0863211	8.49861668	1.92E-17	8.64E-17	Up	MRI1
NFS1	1332.04245	0.67466042	0.07940314	8.49664627	1.95E-17	8.79E-17	Up	NFS1
DCUN1D2	536.894059	0.79882198	0.09402061	8.49624318	1.96E-17	8.82E-17	Up	DCUN1D2
CAPN13	336.621027	-1.7794256	0.20944002	-8.4961107	1.96E-17	8.83E-17	Down	CAPN13

BRICD5	100.577125	1.1025732	0.12978871	8.49513937	1.98E-17	8.90E-17	Up	BRICD5
LSM5	1611.46928	0.73775854	0.08685055	8.49457525	1.99E-17	8.94E-17	Up	LSM5
TNK1	641.80548	-0.74205164	0.0873743	-8.49279033	2.02E-17	9.08E-17	Down	TNK1
COLEC12	266.941606	-1.78981257	0.21074908	-8.49262331	2.02E-17	9.09E-17	Down	COLEC12
KCNAB1	63.6800935	-1.60001629	0.1884315	-8.49123578	2.04E-17	9.19E-17	Down	KCNAB1
HPN	163.926121	2.94324812	0.34665705	8.49037424	2.06E-17	9.26E-17	Up	HPN
STK3	740.812658	0.82454555	0.09712362	8.48964993	2.07E-17	9.31E-17	Up	STK3
CPSF1	5387.3524	0.9589009	0.11295845	8.48897007	2.08E-17	9.37E-17	Up	CPSF1
SCRG1	27.8827032	-2.96785886	0.34962129	-8.48878185	2.09E-17	9.38E-17	Down	SCRG1
EPHX3	32.8622329	1.97530727	0.23272515	8.48772598	2.11E-17	9.46E-17	Up	EPHX3
RMI2	749.230512	0.83478702	0.09837792	8.48551213	2.15E-17	9.64E-17	Up	RMI2
SMAD6	690.067525	0.97781353	0.11523736	8.48521307	2.15E-17	9.66E-17	Up	SMAD6
PCGF1	506.471084	0.56166682	0.06620666	8.48353961	2.18E-17	9.80E-17	Up	PCGF1
PGLYRP3	4.61270605	4.51570126	0.5323603	8.48241547	2.21E-17	9.89E-17	Up	PGLYRP3
MYO5B	4668.99764	-0.70762585	0.08342733	-8.48194269	2.21E-17	9.93E-17	Down	MYO5B
PLIN4	484.425893	-2.99113354	0.35265544	-8.48174514	2.22E-17	9.94E-17	Down	PLIN4
HNRNPD	7289.26173	0.50599835	0.05965866	8.48155674	2.22E-17	9.96E-17	Up	HNRNPD
LRRC7	18.1845763	-1.456573	0.17174252	-8.48114354	2.23E-17	9.99E-17	Down	LRRC7
MIS18A	650.089173	0.66297627	0.07817741	8.48040711	2.24E-17	1.01E-16	Up	MIS18A
DTX2	2173.53486	0.83744091	0.09875386	8.48008241	2.25E-17	1.01E-16	Up	DTX2
CNPY3	2587.51975	0.73232916	0.0863606	8.47989909	2.25E-17	1.01E-16	Up	CNPY3
DNASE1	934.697345	1.12999604	0.13327299	8.47880777	2.28E-17	1.02E-16	Up	DNASE1
SGPP1	589.232836	-0.93992563	0.11087491	-8.47735174	2.30E-17	1.03E-16	Down	SGPP1
FADS1	928.8311	1.43738536	0.1695581	8.47724399	2.31E-17	1.03E-16	Up	FADS1
HEPHL1	55.0276293	2.59963292	0.30670453	8.47601735	2.33E-17	1.04E-16	Up	HEPHL1
SLIT2	223.087122	-1.93973809	0.22888688	-8.47465822	2.36E-17	1.05E-16	Down	SLIT2
EPHA8	8.08353151	4.58325072	0.54086636	8.47390604	2.37E-17	1.06E-16	Up	EPHA8
SNRPD2	6494.13411	0.88010312	0.10387814	8.47245772	2.40E-17	1.07E-16	Up	SNRPD2
C1orf159	593.538542	0.84064295	0.09922343	8.47222207	2.41E-17	1.08E-16	Up	C1orf159
SMIM10	75.4772835	-1.43530657	0.16943223	-8.47127235	2.43E-17	1.08E-16	Down	SMIM10
BRD9	1392.15284	0.59506155	0.07024538	8.47118383	2.43E-17	1.08E-16	Up	BRD9
GRM2	10.693301	1.53608487	0.18133376	8.47103618	2.43E-17	1.09E-16	Up	GRM2
CPN1	30.5286034	3.52782535	0.41663842	8.46735493	2.51E-17	1.12E-16	Up	CPN1
AQP6	16.9699183	1.89631921	0.22397669	8.46659165	2.53E-17	1.13E-16	Up	AQP6
CDCA2	373.540091	1.05957201	0.12515498	8.46607971	2.54E-17	1.13E-16	Up	CDCA2
SLC18A2	25.222928	-1.28194852	0.15143853	-8.46514111	2.56E-17	1.14E-16	Down	SLC18A2
ICAM3	61.1258062	-1.55624338	0.18385511	-8.4645096	2.57E-17	1.15E-16	Down	ICAM3
TIPIN	249.526678	0.77125751	0.09112359	8.4638618	2.59E-17	1.15E-16	Up	TIPIN
BANK1	109.780505	-1.52952238	0.18076471	-8.46139918	2.64E-17	1.18E-16	Down	BANK1
E2F4	3633.40369	0.59401033	0.07022951	8.45812957	2.72E-17	1.21E-16	Up	E2F4
NNT	2333.31288	-0.64218044	0.07592649	-8.45792355	2.72E-17	1.21E-16	Down	NNT
NFASC	466.111486	-1.35399178	0.16009233	-8.45756811	2.73E-17	1.22E-16	Down	NFASC
SNX6	2103.33698	-0.68351091	0.0808189	-8.45731513	2.74E-17	1.22E-16	Down	SNX6
DMXL1	819.507884	-0.85337069	0.10093165	-8.45493619	2.79E-17	1.24E-16	Down	DMXL1
SLC39A6	1340.17017	0.75082978	0.08880669	8.45465345	2.80E-17	1.24E-16	Up	SLC39A6
RNF149	2384.26289	0.53173603	0.06289823	8.45391092	2.82E-17	1.25E-16	Up	RNF149
ANAPC5	5081.94894	0.50008954	0.0591562	8.45371285	2.82E-17	1.25E-16	Up	ANAPC5
OTUD7B	1021.38027	-0.6038982	0.07143829	-8.4534245	2.83E-17	1.26E-16	Down	OTUD7B
TXLNB	24.2670968	-1.59955838	0.18925352	-8.45193469	2.87E-17	1.27E-16	Down	TXLNB
LYRM4	962.259383	0.71258077	0.08431539	8.45137292	2.88E-17	1.28E-16	Up	LYRM4
MZB1	559.168549	-1.97310295	0.23348982	-8.45048809	2.90E-17	1.29E-16	Down	MZB1
MTMR9	334.574367	-0.91320762	0.10807365	-8.44986409	2.92E-17	1.29E-16	Down	MTMR9
RNF180	47.834806	-1.67747053	0.19854763	-8.44870589	2.95E-17	1.31E-16	Down	RNF180
RPL10L	12.0140008	-2.00572411	0.23743425	-8.44749276	2.98E-17	1.32E-16	Down	RPL10L
THOP1	2723.49678	0.95684273	0.11327364	8.44717897	2.98E-17	1.32E-16	Up	THOP1
DENND5B	363.723269	-1.12017706	0.13262733	-8.44605003	3.01E-17	1.34E-16	Down	DENND5B
ARSA	1481.35034	-0.90543865	0.10723455	-8.44353476	3.08E-17	1.37E-16	Down	ARSA
AKR1C8P	3.89633749	3.89343759	0.46137712	8.43873132	3.21E-17	1.42E-16	Up	AKR1C8P

SLC22A15	162.869013	1.2406538	0.14709115	8.43459152	3.32E-17	1.47E-16	Up	SLC22A15
HTR3A	13.2430786	-2.61094614	0.30955525	-8.4345077	3.33E-17	1.47E-16	Down	HTR3A
ASPSCR1	1555.75831	0.95294334	0.11300087	8.43306228	3.37E-17	1.49E-16	Up	ASPSCR1
RDM1	50.5403591	1.19741213	0.14199014	8.43306526	3.37E-17	1.49E-16	Up	RDM1
RABGGTB	2002.42907	0.68403806	0.08113239	8.43113461	3.42E-17	1.51E-16	Up	RABGGTB
SLC15A4	826.044315	0.51750848	0.06138395	8.43068104	3.44E-17	1.52E-16	Up	SLC15A4
SGSM1	106.431798	-1.48946356	0.17668526	-8.43003857	3.46E-17	1.53E-16	Down	SGSM1
PLXNB1	4683.65237	0.83175425	0.09870555	8.42662068	3.56E-17	1.57E-16	Up	PLXNB1
SMARCA4	6717.76399	0.68120256	0.08084124	8.4264235	3.56E-17	1.58E-16	Up	SMARCA4
SPATA25	44.7511903	1.41247215	0.16762418	8.42642246	3.56E-17	1.58E-16	Up	SPATA25
BAD	1028.0337	-0.77654868	0.09216379	-8.42574564	3.58E-17	1.58E-16	Down	BAD
DPEP2	59.0283816	-1.37582003	0.16332062	-8.42404368	3.64E-17	1.61E-16	Down	DPEP2
LMTK2	2344.38803	-0.65818972	0.07818061	-8.41883576	3.80E-17	1.68E-16	Down	LMTK2
CHRM2	84.3579246	-3.73491328	0.44365831	-8.41844539	3.82E-17	1.68E-16	Down	CHRM2
FAM110B	65.7319007	-1.42532684	0.16935154	-8.41637968	3.88E-17	1.71E-16	Down	FAM110B
SYNE1	709.719691	-1.46854877	0.17451096	-8.4152238	3.92E-17	1.73E-16	Down	SYNE1
FANCE	395.11787	0.65424533	0.07775345	8.41435741	3.95E-17	1.74E-16	Up	FANCE
ZNF385B	14.3750423	-2.18707055	0.25994171	-8.41369604	3.97E-17	1.75E-16	Down	ZNF385B
CSNK1G2	2860.52056	0.65463143	0.07781061	8.41313783	3.99E-17	1.76E-16	Up	CSNK1G2
SLC4A8	144.885074	1.40527577	0.16704535	8.41254064	4.01E-17	1.77E-16	Up	SLC4A8
PENK	26.7536103	-3.30760649	0.3931867	-8.41230504	4.02E-17	1.77E-16	Down	PENK
KIAA0895L	603.690197	0.93023188	0.11058259	8.41210105	4.03E-17	1.77E-16	Up	KIAA0895L
PAK3	17.0311394	-1.82782956	0.21730928	-8.4111897	4.06E-17	1.79E-16	Down	PAK3
ZBTB47	421.492578	-0.99645512	0.11847324	-8.41080312	4.07E-17	1.79E-16	Down	ZBTB47
EVX2	6.01494759	-2.96874503	0.35311669	-8.40726342	4.20E-17	1.85E-16	Down	EVX2
KRT24	14.0467011	-5.9882933	0.7124022	-8.405776	4.25E-17	1.87E-16	Down	KRT24
RAB30	443.51482	-1.07288821	0.12764083	-8.40552508	4.26E-17	1.87E-16	Down	RAB30
ZNF142	972.132796	0.59774007	0.07111434	8.40533782	4.27E-17	1.88E-16	Up	ZNF142
SLC5A2	36.9906817	1.47614987	0.17573776	8.39973082	4.48E-17	1.97E-16	Up	SLC5A2
CAPZB	7330.94235	-0.51764975	0.06167129	-8.39369038	4.71E-17	2.07E-16	Down	CAPZB
CCDC136	83.9668894	-1.54811047	0.18447053	-8.39218303	4.77E-17	2.10E-16	Down	CCDC136
SYNCRIP	6961.85429	0.62887161	0.07493896	8.3917847	4.79E-17	2.10E-16	Up	SYNCRIP
MTFR1	1424.2078	0.75883636	0.09045459	8.3891417	4.90E-17	2.15E-16	Up	MTFR1
NHP2	2154.27125	0.71405308	0.08511982	8.38879919	4.91E-17	2.16E-16	Up	NHP2
SCN3B	28.1469048	-1.89955081	0.22644649	-8.38851953	4.92E-17	2.16E-16	Down	SCN3B
VAMP8	3793.10131	-0.76782114	0.09153815	-8.38799088	4.95E-17	2.17E-16	Down	VAMP8
GGN	13.0211654	1.31305904	0.15655073	8.38743491	4.97E-17	2.18E-16	Up	GGN
CCDC96	41.5048183	-0.83352943	0.0993994	-8.38565857	5.04E-17	2.21E-16	Down	CCDC96
SETSIP	5.99734089	1.64752319	0.19649293	8.38464364	5.09E-17	2.23E-16	Up	SETSIP
FGF3	4.97765927	5.05812396	0.60326873	8.38452868	5.09E-17	2.23E-16	Up	FGF3
NPY	7.59161076	-2.91342386	0.3475317	-8.3831887	5.15E-17	2.26E-16	Down	NPY
SAMD4A	526.112523	-1.18503821	0.14137612	-8.38216673	5.20E-17	2.28E-16	Down	SAMD4A
CSF1	827.522674	-1.00204691	0.11956757	-8.38059106	5.27E-17	2.31E-16	Down	CSF1
LONP1	4630.19555	0.90121006	0.10753896	8.38031216	5.28E-17	2.31E-16	Up	LONP1
DPM1	1808.69348	0.82776018	0.09879644	8.37844177	5.36E-17	2.35E-16	Up	DPM1
EYA1	77.6678344	2.83470361	0.33837731	8.37734551	5.41E-17	2.37E-16	Up	EYA1
PPP1R36	131.076285	-1.26390482	0.15087684	-8.37706335	5.43E-17	2.38E-16	Down	PPP1R36
NSUN6	339.068651	0.58083175	0.06935079	8.3752723	5.51E-17	2.41E-16	Up	NSUN6
FBXO22	979.602367	0.58702874	0.07010292	8.37381246	5.58E-17	2.44E-16	Up	FBXO22
COL1A2	54779.7072	1.53578572	0.18342773	8.37270198	5.63E-17	2.46E-16	Up	COL1A2
RAB39B	17.6053465	-1.58490278	0.1893169	-8.37169222	5.68E-17	2.48E-16	Down	RAB39B
MS4A8	328.621648	-2.02076095	0.24144934	-8.36929586	5.80E-17	2.54E-16	Down	MS4A8
HBA2	163.37406	-1.94900897	0.23288702	-8.36890346	5.82E-17	2.54E-16	Down	HBA2
NRIP1	1556.97658	-0.81845811	0.09780999	-8.36783727	5.87E-17	2.57E-16	Down	NRIP1
MAP1LC3C	3.48452638	-1.76284643	0.21069652	-8.36675642	5.92E-17	2.59E-16	Down	MAP1LC3C
RAB3D	1272.5247	0.74521325	0.08906933	8.36666486	5.93E-17	2.59E-16	Up	RAB3D
RASSF3	2686.69884	-0.73675024	0.08806467	-8.36601362	5.96E-17	2.60E-16	Down	RASSF3
FBLN1	3916.19484	-1.59355187	0.19057022	-8.36201946	6.17E-17	2.69E-16	Down	FBLN1

DCPS	783.082343	0.71799867	0.08587444	8.36102906	6.22E-17	2.71E-16	Up	DCPS
NAP1L5	88.578299	-0.99315268	0.11878738	-8.36075889	6.23E-17	2.72E-16	Down	NAP1L5
FIGN	28.249889	-1.67226748	0.20003026	-8.36007244	6.27E-17	2.73E-16	Down	FIGN
MEN1	1654.60508	0.5963837	0.07135893	8.35752053	6.41E-17	2.79E-16	Up	MEN1
CDS1	1698.57469	-0.71464858	0.08552368	-8.35614887	6.48E-17	2.83E-16	Down	CDS1
FZD1	716.136076	-0.87161912	0.10431502	-8.35564386	6.51E-17	2.84E-16	Down	FZD1
PHGDH	2189.6907	1.66237309	0.19898582	8.35422903	6.59E-17	2.87E-16	Up	PHGDH
GSKIP	2757.6492	-0.98751547	0.11823548	-8.35210802	6.71E-17	2.92E-16	Down	GSKIP
FAM177A1	2133.30588	-0.6622084	0.07931602	-8.34898694	6.89E-17	3.00E-16	Down	FAM177A1
AADAT	271.05151	1.07774339	0.12912854	8.34628313	7.04E-17	3.07E-16	Up	AADAT
SMAP1	593.793945	-0.67616299	0.08101765	-8.34587238	7.07E-17	3.08E-16	Down	SMAP1
ZNF658	43.231159	-0.81412493	0.09755942	-8.3449135	7.13E-17	3.10E-16	Down	ZNF658
FGFR1	1142.27295	-1.241001	0.14873591	-8.34365412	7.20E-17	3.14E-16	Down	FGFR1
DPAGT1	2058.6718	0.53082432	0.06362452	8.34307751	7.24E-17	3.15E-16	Up	DPAGT1
PRPS2	3053.71851	0.63710479	0.07636761	8.34260485	7.27E-17	3.16E-16	Up	PRPS2
PRUNE2	1894.82942	-1.6316225	0.19559503	-8.34184013	7.31E-17	3.18E-16	Down	PRUNE2
C1QA	2418.91576	-1.37084431	0.16440464	-8.33823395	7.54E-17	3.28E-16	Down	C1QA
ANKRD39	390.853711	0.68508838	0.08217834	8.33660538	7.65E-17	3.32E-16	Up	ANKRD39
SAMD13	275.119868	-1.21897084	0.14622133	-8.33647775	7.65E-17	3.33E-16	Down	SAMD13
VTCN1	14.508144	3.3675564	0.40400861	8.33535799	7.73E-17	3.36E-16	Up	VTCN1
ID2	2802.65306	-0.80272875	0.09630605	-8.33518478	7.74E-17	3.36E-16	Down	ID2
RGS13	27.7246201	-2.43023721	0.29164016	-8.33299918	7.88E-17	3.42E-16	Down	RGS13
FAM114A1	1610.82047	-0.81415953	0.09770807	-8.33257232	7.91E-17	3.43E-16	Down	FAM114A1
DECR1	2510.18014	-0.66147089	0.07939261	-8.33164332	7.97E-17	3.46E-16	Down	DECR1
BMPR1A	929.932008	-0.62695604	0.07525128	-8.33150009	7.98E-17	3.46E-16	Down	BMPR1A
TNFAIP8L3	127.993884	-1.45835258	0.17506433	-8.33038119	8.06E-17	3.50E-16	Down	TNFAIP8L3
KIFC3	667.388731	1.15640942	0.13882243	8.33013389	8.08E-17	3.50E-16	Up	KIFC3
ZNHIT2	542.899557	1.00080714	0.12019714	8.32638078	8.34E-17	3.61E-16	Up	ZNHIT2
ENO1	46692.8179	0.78189266	0.09391994	8.32509709	8.43E-17	3.65E-16	Up	ENO1
EGFR	2333.81494	-0.83432677	0.10021912	-8.32502578	8.43E-17	3.65E-16	Down	EGFR
HAND1	72.9303261	-4.12551861	0.49556059	-8.32495294	8.44E-17	3.66E-16	Down	HAND1
PEMT	727.205557	0.91876469	0.1103677	8.32457954	8.46E-17	3.67E-16	Up	PEMT
ZNF25	209.738463	-0.90308687	0.10848584	-8.32446807	8.47E-17	3.67E-16	Down	ZNF25
PLEK2	2268.64632	0.81873824	0.09837543	8.32258865	8.61E-17	3.73E-16	Up	PLEK2
GBA2	2905.64613	-0.68431259	0.08223654	-8.32127167	8.70E-17	3.77E-16	Down	GBA2
FCRL3	45.1869285	-2.0600883	0.24765869	-8.3182555	8.93E-17	3.86E-16	Down	FCRL3
TP53INP1	837.735797	-1.05699327	0.12709958	-8.31626085	9.08E-17	3.93E-16	Down	TP53INP1
SLC25A24	2426.70071	-0.80334626	0.09662731	-8.31386344	9.26E-17	4.01E-16	Down	SLC25A24
SAMD9	777.660945	-1.41488967	0.17020966	-8.31262837	9.36E-17	4.05E-16	Down	SAMD9
GEMIN8	637.000908	0.74946341	0.09016474	8.31215601	9.40E-17	4.06E-16	Up	GEMIN8
COL22A1	57.8934488	1.7657956	0.21245204	8.31150238	9.45E-17	4.08E-16	Up	COL22A1
SLC25A27	200.654308	1.33074266	0.16011172	8.31133812	9.46E-17	4.09E-16	Up	SLC25A27
ZC3H12C	413.282266	-1.39280608	0.16763317	-8.30865445	9.68E-17	4.18E-16	Down	ZC3H12C
SERPINA7	33.1943011	3.33984457	0.40198163	8.30845064	9.70E-17	4.19E-16	Up	SERPINA7
GPX2	23055.701	1.11183112	0.13382268	8.30824159	9.71E-17	4.19E-16	Up	GPX2
STMN1	5625.44001	0.83571165	0.10059325	8.30783011	9.75E-17	4.21E-16	Up	STMN1
TRIM16	707.656278	0.83451675	0.10048496	8.30489199	9.99E-17	4.31E-16	Up	TRIM16
GLI3	191.498792	-1.72257954	0.20743907	-8.30402655	1.01E-16	4.34E-16	Down	GLI3
NLRP7	8.23872959	-2.05481794	0.24745824	-8.30369576	1.01E-16	4.35E-16	Down	NLRP7
PIEZO1	6131.74378	0.88796018	0.10693872	8.30344843	1.01E-16	4.36E-16	Up	PIEZO1
CLEC4G	12.5563392	-2.61231781	0.31468371	-8.30140783	1.03E-16	4.43E-16	Down	CLEC4G
SLC12A9	2607.66242	0.79035537	0.09520846	8.30131479	1.03E-16	4.44E-16	Up	SLC12A9
POU3F2	4.43123449	2.62152098	0.31581891	8.30070931	1.03E-16	4.46E-16	Up	POU3F2
LY86	85.0760661	-1.26765876	0.15276556	-8.29806635	1.06E-16	4.56E-16	Down	LY86
TRIM54	113.297968	2.86686538	0.34551014	8.29748548	1.06E-16	4.58E-16	Up	TRIM54
HES7	5.74851924	3.0475036	0.3673637	8.29560357	1.08E-16	4.65E-16	Up	HES7
TOMM20	8938.37478	0.58857423	0.07097055	8.29321821	1.10E-16	4.74E-16	Up	TOMM20
KIFAP3	1125.24328	-0.6634155	0.08001711	-8.29092027	1.12E-16	4.84E-16	Down	KIFAP3

KRT222	2.9662978	-2.70625259	0.32644372	-8.29010469	1.13E-16	4.87E-16	Down	KRT222
TRPM4	4352.75682	-1.00333532	0.12103325	-8.28974962	1.13E-16	4.88E-16	Down	TRPM4
P3H4	1170.12085	0.93002008	0.11221301	8.28798806	1.15E-16	4.95E-16	Up	P3H4
CCL19	155.655941	-2.32789953	0.28089113	-8.28755091	1.16E-16	4.97E-16	Down	CCL19
BPNT1	1793.5096	-0.72044329	0.08696108	-8.28466365	1.18E-16	5.09E-16	Down	BPNT1
PRKRIP1	772.728913	0.56516044	0.0682248	8.28379777	1.19E-16	5.13E-16	Up	PRKRIP1
RAD1	888.825066	0.56221335	0.06788604	8.28172301	1.21E-16	5.21E-16	Up	RAD1
GATA4	64.9774701	4.63232388	0.55939806	8.28090805	1.22E-16	5.25E-16	Up	GATA4
MUC13	23606.2669	-0.87120962	0.10520851	-8.28079013	1.22E-16	5.25E-16	Down	MUC13
ZNF623	1259.41534	0.67409247	0.08140426	8.28080101	1.22E-16	5.25E-16	Up	ZNF623
DCLRE1A	613.495317	0.59976931	0.07244745	8.2786803	1.25E-16	5.34E-16	Up	DCLRE1A
PCP2	11.8873222	1.73408552	0.20950896	8.27690392	1.26E-16	5.42E-16	Up	PCP2
ZNF878	13.4048454	1.32641718	0.16029113	8.27505042	1.28E-16	5.51E-16	Up	ZNF878
ARHGAP33	313.707883	1.05267269	0.12722821	8.27389342	1.30E-16	5.56E-16	Up	ARHGAP33
IFT22	532.706082	0.68206917	0.08246267	8.27124728	1.33E-16	5.68E-16	Up	IFT22
SPATA17	44.6339969	1.32458518	0.16018775	8.26895426	1.35E-16	5.79E-16	Up	SPATA17
AKAP17A	1829.86441	0.67720243	0.08190293	8.26835419	1.36E-16	5.82E-16	Up	AKAP17A
TRPC4AP	5396.63006	0.62245602	0.07528314	8.26819987	1.36E-16	5.82E-16	Up	TRPC4AP
DIRAS1	34.1610053	-1.86777284	0.22594359	-8.26654485	1.38E-16	5.90E-16	Down	DIRAS1
FLT3	19.4462117	-1.75621056	0.21247282	-8.26557764	1.39E-16	5.95E-16	Down	FLT3
GLOD5	67.5646485	-1.27637928	0.15443346	-8.26491436	1.40E-16	5.98E-16	Down	GLOD5
RBBP7	5721.2849	0.60222839	0.07287511	8.26384198	1.41E-16	6.04E-16	Up	RBBP7
ACVR2B	481.675593	0.88824613	0.107492	8.26336947	1.42E-16	6.06E-16	Up	ACVR2B
MCTS1	1558.40588	0.6810016	0.08241672	8.26290541	1.42E-16	6.08E-16	Up	MCTS1
SCN4A	11.1551055	-1.99526441	0.24149085	-8.26227746	1.43E-16	6.11E-16	Down	SCN4A
MDH1	4453.88723	-0.53603047	0.0648817	-8.26165837	1.44E-16	6.14E-16	Down	MDH1
KIF20B	777.723094	0.93741485	0.11348515	8.26024257	1.45E-16	6.21E-16	Up	KIF20B
SDHA	5948.10589	-0.76715774	0.09288557	-8.25917048	1.47E-16	6.27E-16	Down	SDHA
POU2AF1	379.297687	-1.94212635	0.23514783	-8.25917181	1.47E-16	6.27E-16	Down	POU2AF1
BNIPL	21.2342124	1.35695781	0.16430531	8.25875789	1.47E-16	6.29E-16	Up	BNIPL
ACOT9	1303.021	0.5873587	0.07112377	8.25826152	1.48E-16	6.31E-16	Up	ACOT9
KCTD12	2069.92945	-1.44066549	0.17448915	-8.25647628	1.50E-16	6.40E-16	Down	KCTD12
EHD2	1852.52373	-1.26488666	0.15320258	-8.25630131	1.50E-16	6.41E-16	Down	EHD2
SLC2A12	264.493678	1.69365281	0.20517586	8.25463993	1.52E-16	6.50E-16	Up	SLC2A12
ATP6V1B1	19.1961833	2.09599249	0.25392569	8.2543538	1.53E-16	6.51E-16	Up	ATP6V1B1
MTA1	2031.04228	0.74713802	0.09053377	8.25258917	1.55E-16	6.61E-16	Up	MTA1
PLD5	2.4615419	-3.17951181	0.38529473	-8.25215491	1.56E-16	6.63E-16	Down	PLD5
UBE2G1	1520.8528	-0.53065253	0.06430514	-8.25210136	1.56E-16	6.63E-16	Down	UBE2G1
SNRPC	2933.01583	0.64507877	0.07818611	8.25055463	1.58E-16	6.72E-16	Up	SNRPC
CDR2	1540.72632	0.54902617	0.06654405	8.25056689	1.58E-16	6.72E-16	Up	CDR2
SLC35D2	1939.1197	-0.64267651	0.0778994	-8.25008279	1.58E-16	6.74E-16	Down	SLC35D2
IFI6	3760.55071	1.6412359	0.19896306	8.24894804	1.60E-16	6.80E-16	Up	IFI6
SRD5A1	977.981362	0.68506131	0.08304836	8.24894398	1.60E-16	6.80E-16	Up	SRD5A1
RAB3C	30.3529271	-2.09047674	0.25350895	-8.24616543	1.64E-16	6.96E-16	Down	RAB3C
AP1S1	3976.61095	0.84574533	0.10257244	8.24534665	1.65E-16	7.01E-16	Up	AP1S1
VSTM4	311.091419	-1.23614719	0.14994828	-8.2438237	1.67E-16	7.09E-16	Down	VSTM4
HNRNPA1L2	161.940649	0.84716333	0.10277322	8.24303584	1.68E-16	7.14E-16	Up	HNRNPA1L2
ZNF354B	198.247277	0.8118059	0.0984963	8.24199381	1.69E-16	7.20E-16	Up	ZNF354B
TLN2	1629.11947	-0.91638469	0.11119183	-8.24147511	1.70E-16	7.23E-16	Down	TLN2
SLC2A5	215.755666	-1.56280582	0.18964357	-8.24075291	1.71E-16	7.27E-16	Down	SLC2A5
PTPRR	443.671916	-1.68810769	0.2048491	-8.24073776	1.71E-16	7.27E-16	Down	PTPRR
CDR2L	647.499496	1.13387654	0.13761204	8.23966085	1.73E-16	7.33E-16	Up	CDR2L
CALB1	112.457989	3.37275162	0.40941434	8.23799092	1.75E-16	7.44E-16	Up	CALB1
ORC2	1184.67337	0.5512473	0.06691984	8.23742717	1.76E-16	7.47E-16	Up	ORC2
FBXL17	827.667591	-0.6570692	0.07977445	-8.23658721	1.77E-16	7.52E-16	Down	FBXL17
STAP2	3504.78695	-0.9820131	0.11925077	-8.23485743	1.80E-16	7.63E-16	Down	STAP2
RHOF	218.112475	-1.41359049	0.17167861	-8.23393483	1.81E-16	7.68E-16	Down	RHOF
CYYR1	211.200161	-0.99903862	0.1213549	-8.23237149	1.84E-16	7.78E-16	Down	CYYR1

MYO19	2084.73578	0.64733279	0.07863817	8.23178871	1.84E-16	7.82E-16	Up	MYO19
GRPR	30.5912998	2.70352942	0.32844753	8.23123677	1.85E-16	7.85E-16	Up	GRPR
ANKEF1	660.344154	0.83281532	0.1011916	8.23008332	1.87E-16	7.93E-16	Up	ANKEF1
PELI2	391.171404	-1.20452401	0.14636755	-8.22944734	1.88E-16	7.97E-16	Down	PELI2
LTV1	875.325423	0.69042808	0.08390578	8.22861193	1.89E-16	8.02E-16	Up	LTV1
PYROXD1	516.124541	-0.76015941	0.09239108	-8.22762744	1.91E-16	8.09E-16	Down	PYROXD1
GBF1	3359.73497	-0.55093735	0.06696411	-8.22735304	1.91E-16	8.10E-16	Down	GBF1
CFH	856.52351	-1.33959494	0.16290239	-8.22329814	1.98E-16	8.38E-16	Down	CFH
LRRC15	492.569121	1.73828316	0.21146611	8.22015006	2.03E-16	8.60E-16	Up	LRRC15
MALRD1	103.504635	2.9365382	0.35726803	8.21942612	2.04E-16	8.65E-16	Up	MALRD1
BTBD19	117.557671	1.29998212	0.15817367	8.21870137	2.06E-16	8.70E-16	Up	BTBD19
TMEM217	16.1651615	-0.99208235	0.12075089	-8.21594236	2.11E-16	8.90E-16	Down	TMEM217
BAK1	1709.07511	-0.76189487	0.09274224	-8.21518696	2.12E-16	8.95E-16	Down	BAK1
ENAM	11.6132512	-2.49520439	0.30375862	-8.21443142	2.13E-16	9.01E-16	Down	ENAM
SUSD5	31.0163491	-1.80758335	0.22013011	-8.21143177	2.19E-16	9.23E-16	Down	SUSD5
COL4A1	12790.4602	1.09251189	0.13305133	8.21120617	2.19E-16	9.25E-16	Up	COL4A1
POLR2H	2356.42094	0.64044072	0.07799822	8.21096533	2.19E-16	9.26E-16	Up	POLR2H
REXO4	1403.13627	0.63506454	0.07736714	8.20845345	2.24E-16	9.46E-16	Up	REXO4
TMEM179	13.6222241	-2.61122562	0.31813311	-8.2079656	2.25E-16	9.49E-16	Down	TMEM179
FANCL	542.545226	0.58962777	0.07186177	8.20502761	2.31E-16	9.73E-16	Up	FANCL
ZNF335	1106.39014	0.6955563	0.08479062	8.20322215	2.34E-16	9.87E-16	Up	ZNF335
IL1RAP	320.471706	1.00383209	0.12237469	8.20293855	2.35E-16	9.89E-16	Up	IL1RAP
SMCHD1	1527.22181	-1.05920664	0.12913744	-8.20216551	2.36E-16	9.95E-16	Down	SMCHD1
ETV5	604.504719	1.28827266	0.15706829	8.201991	2.36E-16	9.97E-16	Up	ETV5
S100A5	16.8161271	1.7120619	0.20874119	8.20184029	2.37E-16	9.98E-16	Up	S100A5
AKIRIN2	1635.82448	0.55759603	0.06798808	8.20137909	2.38E-16	1.00E-15	Up	AKIRIN2
SEZ6L2	2035.15263	1.16479231	0.14203323	8.20084376	2.39E-16	1.01E-15	Up	SEZ6L2
ZNF146	2881.50831	0.63425988	0.07736894	8.19786231	2.45E-16	1.03E-15	Up	ZNF146
ORC3	817.9163	0.55076585	0.06719686	8.19630374	2.48E-16	1.04E-15	Up	ORC3
KLHL6	224.59337	-1.47790563	0.1803542	-8.19446214	2.52E-16	1.06E-15	Down	KLHL6
SHISA5	8025.1447	0.76845929	0.09378737	8.19363284	2.53E-16	1.07E-15	Up	SHISA5
RPUSD3	1171.10677	0.59971336	0.0731956	8.19329806	2.54E-16	1.07E-15	Up	RPUSD3
TGFBR3	748.640658	-1.27418316	0.15556255	-8.19080902	2.59E-16	1.09E-15	Down	TGFBR3
GPI	18074.356	0.63633663	0.07775168	8.18421696	2.74E-16	1.15E-15	Up	GPI
TOR1AIP1	1543.83946	-0.63968015	0.07816498	-8.18371794	2.75E-16	1.16E-15	Down	TOR1AIP1
SMTNL2	66.0756669	2.12253037	0.25948278	8.17985051	2.84E-16	1.19E-15	Up	SMTNL2
TRPC6	57.096014	-1.2395308	0.15154323	-8.17938724	2.85E-16	1.20E-15	Down	TRPC6
HIBCH	1156.11247	-0.69158486	0.08457187	-8.1774813	2.90E-16	1.22E-15	Down	HIBCH
FKBP9	4731.3925	0.57431953	0.07024316	8.17616324	2.93E-16	1.23E-15	Up	FKBP9
CFAP74	20.127812	2.0118085	0.24607891	8.17546104	2.95E-16	1.24E-15	Up	CFAP74
TM7SF2	810.56946	1.02699793	0.12564649	8.17370951	2.99E-16	1.25E-15	Up	TM7SF2
RNASE1	4299.57667	-1.18782235	0.14534371	-8.17250623	3.02E-16	1.27E-15	Down	RNASE1
SLC35B2	3069.29222	0.61622905	0.07541489	8.17118559	3.05E-16	1.28E-15	Up	SLC35B2
TMEM120A	1571.79602	-0.85711244	0.10489881	-8.17085024	3.06E-16	1.28E-15	Down	TMEM120A
HEXIM1	1338.15748	-0.67215739	0.08226788	-8.17035019	3.07E-16	1.29E-15	Down	HEXIM1
TMEM47	505.062691	-1.41062858	0.17266717	-8.16963962	3.09E-16	1.30E-15	Down	TMEM47
STAU1	7781.41385	0.56971155	0.06974021	8.16905402	3.11E-16	1.30E-15	Up	STAU1
NTN4	586.959093	-1.01226122	0.12395041	-8.16666318	3.17E-16	1.33E-15	Down	NTN4
HMGCS2	8597.89352	-2.20488534	0.2699983	-8.16629328	3.18E-16	1.33E-15	Down	HMGCS2
CFB	674.065145	1.28220614	0.1570211	8.1658208	3.19E-16	1.34E-15	Up	CFB
VWC2	8.32120499	-2.14120325	0.2622476	-8.16481557	3.22E-16	1.35E-15	Down	VWC2
DCTN1	4300.08959	-0.50833869	0.0622957	-8.16009226	3.35E-16	1.40E-15	Down	DCTN1
EEFSEC	939.373708	0.6782873	0.08315372	8.15702899	3.43E-16	1.44E-15	Up	EEFSEC
PAN2	1199.57428	0.68365623	0.08381267	8.15695509	3.44E-16	1.44E-15	Up	PAN2
PSME4	2609.08694	0.55146548	0.06761457	8.15601536	3.46E-16	1.45E-15	Up	PSME4
ZCCHC10	607.243712	-0.64887374	0.07956372	-8.15539739	3.48E-16	1.46E-15	Down	ZCCHC10
MRPL9	1547.61587	0.52185033	0.06398921	8.1552866	3.48E-16	1.46E-15	Up	MRPL9
ANO9	5108.37296	1.2813903	0.15714026	8.15443668	3.51E-16	1.47E-15	Up	ANO9

RGS5	2680.36094	-1.29010066	0.15821905	-8.15388979	3.52E-16	1.47E-15	Down	RGS5
HYAL2	2197.22868	0.8521967	0.10451572	8.15376539	3.53E-16	1.47E-15	Up	HYAL2
FZD4	496.12971	-0.92063492	0.11291395	-8.15342051	3.54E-16	1.48E-15	Down	FZD4
C20orf27	2209.49524	1.08633723	0.13327539	8.15107125	3.61E-16	1.51E-15	Up	C20orf27
ABCB1	1574.13774	-1.75888864	0.21580347	-8.15041861	3.63E-16	1.51E-15	Down	ABCB1
SKAP1	414.065688	1.33940334	0.16434294	8.15005086	3.64E-16	1.52E-15	Up	SKAP1
SEC14L6	6.52015791	-1.72524177	0.21170103	-8.14942531	3.66E-16	1.53E-15	Down	SEC14L6
SH3PXD2B	1852.32795	0.78274219	0.0960657	8.14798781	3.70E-16	1.54E-15	Up	SH3PXD2B
OLFM3	5.69515927	-3.17387137	0.38956572	-8.14720397	3.72E-16	1.55E-15	Down	OLFM3
DCAF4L1	10.9112572	1.52608296	0.1874037	8.14329145	3.85E-16	1.60E-15	Up	DCAF4L1
CCDC80	1921.88014	-1.79672253	0.22068039	-8.14174086	3.90E-16	1.62E-15	Down	CCDC80
COQ10B	946.904448	-0.56483848	0.06940646	-8.138125	4.01E-16	1.67E-15	Down	COQ10B
ZNF850	128.691548	1.14650761	0.1408849	8.13790263	4.02E-16	1.68E-15	Up	ZNF850
POMGNT1	2236.44935	0.60269689	0.07406775	8.1371027	4.05E-16	1.69E-15	Up	POMGNT1
NCF1	76.7665867	-1.45333981	0.17861914	-8.13652892	4.07E-16	1.69E-15	Down	NCF1
ANKMY1	551.207753	0.82724417	0.10168327	8.13549906	4.10E-16	1.71E-15	Up	ANKMY1
XRCC3	687.580492	0.85759897	0.10541564	8.13540522	4.11E-16	1.71E-15	Up	XRCC3
SLC25A17	673.007635	0.52660665	0.06473089	8.13532246	4.11E-16	1.71E-15	Up	SLC25A17
CYP3A4	37.6123863	-2.34009242	0.28767702	-8.13444321	4.14E-16	1.72E-15	Down	CYP3A4
ATP2A1	57.3158135	1.32737632	0.16319171	8.13384647	4.16E-16	1.73E-15	Up	ATP2A1
NHEJ1	49.6853044	-0.61566728	0.07569976	-8.13301483	4.19E-16	1.74E-15	Down	NHEJ1
HCLS1	776.152204	-1.13848308	0.13999074	-8.1325601	4.20E-16	1.75E-15	Down	HCLS1
VPS37B	2872.65964	-0.67277827	0.08274158	-8.13107853	4.25E-16	1.77E-15	Down	VPS37B
GBP3	1868.03143	-1.27132026	0.15635708	-8.13087734	4.26E-16	1.77E-15	Down	GBP3
TEX19	4.32442279	2.67376348	0.32888493	8.12978406	4.30E-16	1.79E-15	Up	TEX19
ERGIC3	14533.6021	0.89497323	0.11009788	8.12888676	4.33E-16	1.80E-15	Up	ERGIC3
SPX	2.91193238	-2.28910853	0.28167888	-8.12666027	4.41E-16	1.83E-15	Down	SPX
USP26	1.52299386	3.46998507	0.42699831	8.1264609	4.42E-16	1.83E-15	Up	USP26
MS4A15	22.4330555	2.64243016	0.3253007	8.12303863	4.55E-16	1.89E-15	Up	MS4A15
ZNF497	65.6523098	1.00461518	0.12368364	8.12245832	4.57E-16	1.90E-15	Up	ZNF497
F5	289.221109	2.32113062	0.28579216	8.12174361	4.60E-16	1.91E-15	Up	F5
COL5A3	690.080004	1.2437816	0.15317305	8.12010712	4.66E-16	1.93E-15	Up	COL5A3
TMEM132B	22.5048394	-1.69301283	0.20849966	-8.11997903	4.66E-16	1.93E-15	Down	TMEM132B
SNRPE	1723.19897	0.73303545	0.09028258	8.11934512	4.69E-16	1.94E-15	Up	SNRPE
OASL	555.04638	-1.39906725	0.17237337	-8.1164931	4.80E-16	1.99E-15	Down	OASL
HNF4G	1397.5445	-0.86975855	0.10718051	-8.11489436	4.86E-16	2.01E-15	Down	HNF4G
ATXN2L	4737.34683	0.58424402	0.07200303	8.11415849	4.89E-16	2.03E-15	Up	ATXN2L
PHYKPL	1310.58334	-0.75314816	0.09282444	-8.11368353	4.91E-16	2.03E-15	Down	PHYKPL
COL8A1	1393.24883	1.7125917	0.21110257	8.11260477	4.95E-16	2.05E-15	Up	COL8A1
YPEL2	803.908752	-0.71003288	0.08753004	-8.1118767	4.98E-16	2.06E-15	Down	YPEL2
MIA2	5.07816614	-2.05086768	0.25283438	-8.11150647	5.00E-16	2.07E-15	Down	MIA2
DCLK3	23.8678539	1.19102398	0.14688085	8.10877673	5.11E-16	2.12E-15	Up	DCLK3
ITPA	2028.74531	0.7743037	0.09552307	8.10593442	5.23E-16	2.17E-15	Up	ITPA
ABCC8	14.4012957	-2.10184252	0.25931533	-8.10535405	5.26E-16	2.18E-15	Down	ABCC8
FAM111A	1407.20086	0.6689113	0.08253111	8.10495934	5.28E-16	2.18E-15	Up	FAM111A
CBX6	1137.81556	-1.21227689	0.1496339	-8.10161934	5.42E-16	2.24E-15	Down	CBX6
NCKAP1L	628.785152	-1.27344896	0.15723531	-8.09900136	5.54E-16	2.29E-15	Down	NCKAP1L
KLC4	2407.30014	-0.89091801	0.11000898	-8.09859362	5.56E-16	2.30E-15	Down	KLC4
AGAP1	2137.12249	-0.53310378	0.06583097	-8.09807014	5.58E-16	2.31E-15	Down	AGAP1
METTL22	434.983551	0.6357235	0.07851873	8.09645634	5.66E-16	2.34E-15	Up	METTL22
FADS2	1921.83386	1.77337738	0.2190426	8.09603873	5.68E-16	2.34E-15	Up	FADS2
KIF1A	75.9517009	-2.66039173	0.32867855	-8.09420541	5.76E-16	2.38E-15	Down	KIF1A
DUSP18	392.481493	0.7135377	0.08815445	8.09417643	5.77E-16	2.38E-15	Up	DUSP18
CALML3	25.5738799	2.40513775	0.29720532	8.09251241	5.84E-16	2.41E-15	Up	CALML3
CKMT1A	511.102195	-1.03254409	0.12761659	-8.09098608	5.92E-16	2.44E-15	Down	CKMT1A
DNAJA1	6580.00247	0.53369359	0.0659757	8.08924502	6.00E-16	2.48E-15	Up	DNAJA1
CCDC178	3.82745903	-1.88880703	0.23352479	-8.08825079	6.05E-16	2.50E-15	Down	CCDC178
SNCG	96.9663614	-1.7380748	0.21490079	-8.08780084	6.08E-16	2.50E-15	Down	SNCG

CTSO	828.386758	-0.93190409	0.11526787	-8.08468214	6.23E-16	2.57E-15	Down	CTSO
RPS15	18421.4027	1.04466996	0.12922543	8.08408955	6.26E-16	2.58E-15	Up	RPS15
GRIN2A	10.3169974	-2.55135714	0.31561033	-8.08388362	6.27E-16	2.58E-15	Down	GRIN2A
STARD9	244.20328	-1.24445114	0.15396035	-8.08293262	6.32E-16	2.60E-15	Down	STARD9
HMGA2	617.458421	1.60000316	0.19799296	8.08111156	6.42E-16	2.64E-15	Up	HMGA2
NPTX2	856.614323	2.69989357	0.33411396	8.08075661	6.44E-16	2.65E-15	Up	NPTX2
HDAC10	352.67675	0.95744756	0.11849951	8.07975988	6.49E-16	2.67E-15	Up	HDAC10
PPOX	427.427811	0.709905	0.08787183	8.07886903	6.54E-16	2.69E-15	Up	PPOX
DGKZ	3476.24007	0.75417798	0.0933599	8.07817908	6.57E-16	2.70E-15	Up	DGKZ
SPHKAP	1.62477718	-3.60478834	0.44627086	-8.07757954	6.61E-16	2.72E-15	Down	SPHKAP
ADA	415.375527	1.15518268	0.14303525	8.07620983	6.68E-16	2.75E-15	Up	ADA
NRG3	3.01140712	-2.04506157	0.25324192	-8.07552544	6.72E-16	2.76E-15	Down	NRG3
CCL20	2077.79051	1.6318949	0.20212846	8.07355323	6.83E-16	2.81E-15	Up	CCL20
CYP2F1	8.03107584	1.88348965	0.2332951	8.07342122	6.84E-16	2.81E-15	Up	CYP2F1
CD164	17502.8265	-0.63955037	0.07923691	-8.07136938	6.95E-16	2.86E-15	Down	CD164
CACYBP	2763.78441	0.61079501	0.07567896	8.07086982	6.98E-16	2.87E-15	Up	CACYBP
RFXAP	250.596399	0.78172963	0.09687154	8.06975521	7.04E-16	2.89E-15	Up	RFXAP
OGFR	2561.97805	0.76923604	0.09532479	8.06963287	7.05E-16	2.89E-15	Up	OGFR
DUSP10	483.374247	0.93764226	0.11620006	8.06920635	7.08E-16	2.90E-15	Up	DUSP10
TIMM17B	2316.50274	0.91281681	0.1131285	8.06884948	7.10E-16	2.91E-15	Up	TIMM17B
SFTPD	4.62501172	-1.86291261	0.23091551	-8.06750757	7.17E-16	2.94E-15	Down	SFTPD
CATSPERG	49.2002173	-1.09404568	0.13562404	-8.06675315	7.22E-16	2.96E-15	Down	CATSPERG
SEZ6L	12.7765339	-2.44449301	0.30304212	-8.06651237	7.23E-16	2.97E-15	Down	SEZ6L
CD37	380.234599	-1.38464241	0.17171512	-8.06360204	7.41E-16	3.04E-15	Down	CD37
ANXA8L1	9.01222956	-2.29218719	0.28429312	-8.06275992	7.46E-16	3.06E-15	Down	ANXA8L1
HLX	258.830678	-1.00747483	0.12496293	-8.06218943	7.49E-16	3.07E-15	Down	HLX
RIMS3	256.31338	-1.42062569	0.17623281	-8.06107401	7.56E-16	3.10E-15	Down	RIMS3
PNN	3105.35621	0.65385404	0.0811325	8.05908882	7.69E-16	3.15E-15	Up	PNN
L3HYPDH	177.186234	0.82756784	0.1026923	8.05871337	7.71E-16	3.16E-15	Up	L3HYPDH
FASTKD2	1410.70989	0.51000707	0.06330336	8.05655644	7.85E-16	3.21E-15	Up	FASTKD2
EFS	119.8578	-1.19015792	0.14775053	-8.05518531	7.94E-16	3.25E-15	Down	EFS
RASGRP4	52.2060741	-1.35322128	0.16799733	-8.05501645	7.95E-16	3.25E-15	Down	RASGRP4
TDRKH	413.298941	0.6764658	0.0839931	8.05382624	8.02E-16	3.28E-15	Up	TDRKH
PDLIM1	9231.18269	-0.61239404	0.07604619	-8.05292221	8.08E-16	3.31E-15	Down	PDLIM1
CENPU	746.59611	0.93793098	0.11647386	8.05271654	8.10E-16	3.31E-15	Up	CENPU
ASB11	1.22822776	-2.77107679	0.34414388	-8.05208791	8.14E-16	3.33E-15	Down	ASB11
DSEL	60.5330054	-1.47254384	0.18287711	-8.05209484	8.14E-16	3.33E-15	Down	DSEL
AXL	1035.59463	-1.13706573	0.14121885	-8.05179846	8.16E-16	3.33E-15	Down	AXL
CHGB	135.05291	-2.56879297	0.31914105	-8.04908347	8.34E-16	3.41E-15	Down	CHGB
ABCB5	7.97417566	-3.13006619	0.38904598	-8.04549172	8.59E-16	3.51E-15	Down	ABCB5
GPALPP1	1067.19057	0.87823125	0.10916826	8.04474927	8.64E-16	3.53E-15	Up	GPALPP1
KLHL29	424.170181	1.03845795	0.1291014	8.04373886	8.71E-16	3.56E-15	Up	KLHL29
NARF	1876.75934	0.5948237	0.07395087	8.04349844	8.73E-16	3.56E-15	Up	NARF
SLC25A19	390.833088	0.58647067	0.07292228	8.04240679	8.81E-16	3.59E-15	Up	SLC25A19
S100A7	6.20885826	4.26984229	0.53094837	8.04191612	8.84E-16	3.61E-15	Up	S100A7
THNSL1	420.264482	0.70272334	0.08738647	8.04155797	8.87E-16	3.62E-15	Up	THNSL1
GRIA3	12.472323	-1.94720466	0.24215417	-8.04117766	8.90E-16	3.63E-15	Down	GRIA3
PCDH7	382.508136	-1.52523455	0.18972537	-8.03917045	9.04E-16	3.69E-15	Down	PCDH7
TM4SF1	4982.99759	1.03000745	0.12813199	8.03864392	9.08E-16	3.70E-15	Up	TM4SF1
IQCH	57.1941485	1.07500473	0.1337476	8.03756275	9.16E-16	3.73E-15	Up	IQCH
EMCN	182.440412	-1.14469055	0.14245091	-8.03568419	9.31E-16	3.79E-15	Down	EMCN
SAA4	4.32107726	3.74546223	0.46619249	8.03415395	9.42E-16	3.83E-15	Up	SAA4
IL36RN	5.25073705	3.44128902	0.4283773	8.03331326	9.49E-16	3.86E-15	Up	IL36RN
ERICH4	10.8893762	3.00864417	0.3745622	8.03242873	9.56E-16	3.89E-15	Up	ERICH4
DTNA	175.276626	-1.80229229	0.22437808	-8.03239025	9.56E-16	3.89E-15	Down	DTNA
AMOTL2	1390.87653	0.75166297	0.09359471	8.03104139	9.66E-16	3.93E-15	Up	AMOTL2
ANKRD18B	10.571247	3.3030011	0.41136244	8.02941823	9.79E-16	3.98E-15	Up	ANKRD18B
ERCC2	1123.73922	0.7266866	0.09051397	8.02844672	9.87E-16	4.01E-15	Up	ERCC2

IL17A	13.9183813	2.66250219	0.3316567	8.02788593	9.92E-16	4.03E-15	Up	IL17A
GPR142	2.41291154	-1.95062856	0.2429914	-8.02756222	9.94E-16	4.04E-15	Down	GPR142
CPTP	1425.33763	-0.85619147	0.10665795	-8.02745082	9.95E-16	4.04E-15	Down	CPTP
ARG2	158.329639	1.07772838	0.1342591	8.02722781	9.97E-16	4.05E-15	Up	ARG2
NET1	6744.61373	-0.55985009	0.06974574	-8.02701439	9.99E-16	4.05E-15	Down	NET1
DNAJC18	175.821292	-0.71371666	0.088923	-8.0262324	1.01E-15	4.08E-15	Down	DNAJC18
MTUS1	2317.49559	-0.90975374	0.11335096	-8.02599085	1.01E-15	4.09E-15	Down	MTUS1
VSIG10	1942.14753	-0.55550463	0.06921703	-8.02554813	1.01E-15	4.10E-15	Down	VSIG10
ETFA	4328.07016	-0.67704629	0.08436797	-8.02492099	1.02E-15	4.12E-15	Down	ETFA
ZFP2	27.4463227	-1.0080042	0.12566207	-8.02154705	1.04E-15	4.23E-15	Down	ZFP2
DIMT1	1190.15417	0.58801705	0.0733096	8.02101038	1.05E-15	4.25E-15	Up	DIMT1
DLEU7	35.1375774	1.46510799	0.18267072	8.02048624	1.05E-15	4.27E-15	Up	DLEU7
TGFA	856.540982	-0.9202264	0.11474508	-8.01974605	1.06E-15	4.29E-15	Down	TGFA
PRR22	99.462459	1.20757529	0.15057769	8.01961619	1.06E-15	4.30E-15	Up	PRR22
YPEL4	18.2479585	-1.40277451	0.17493099	-8.01901642	1.07E-15	4.32E-15	Down	YPEL4
DEFA6	682.557528	3.34473456	0.41711257	8.01878144	1.07E-15	4.32E-15	Up	DEFA6
CCDC157	53.5458455	0.73096703	0.09117294	8.01736859	1.08E-15	4.37E-15	Up	CCDC157
PRAME	106.780486	2.80443911	0.34981645	8.01688749	1.08E-15	4.39E-15	Up	PRAME
INHBE	23.2571589	1.2462547	0.15547718	8.01567614	1.10E-15	4.43E-15	Up	INHBE
MPP7	963.091784	-0.96666751	0.12063344	-8.01326312	1.12E-15	4.52E-15	Down	MPP7
SLC1A4	2169.04717	0.72501133	0.09048884	8.01216274	1.13E-15	4.56E-15	Up	SLC1A4
NDUFB1	1279.18053	-0.70343004	0.087798	-8.01191426	1.13E-15	4.57E-15	Down	NDUFB1
ZDHHC8	1351.69987	0.89181743	0.11133207	8.01042687	1.14E-15	4.62E-15	Up	ZDHHC8
NT5C3B	1192.52061	0.90478083	0.11295897	8.0098183	1.15E-15	4.64E-15	Up	NT5C3B
PCDHB9	77.657566	1.66905402	0.20837649	8.00980018	1.15E-15	4.64E-15	Up	PCDHB9
FSCN1	2236.87752	1.55332215	0.19394978	8.00888854	1.16E-15	4.68E-15	Up	FSCN1
WASF2	5702.25178	-0.53760301	0.06712786	-8.00864258	1.16E-15	4.68E-15	Down	WASF2
TMEM167B	1345.46806	-0.54413882	0.06794648	-8.00834498	1.16E-15	4.70E-15	Down	TMEM167B
CTNNB1	20353.139	0.53102358	0.06633345	8.00536616	1.19E-15	4.81E-15	Up	CTNNB1
RAB4B	394.843501	-0.69344853	0.08663451	-8.0042992	1.20E-15	4.85E-15	Down	RAB4B
UQCC1	1913.4387	0.65941168	0.0823829	8.00422981	1.20E-15	4.85E-15	Up	UQCC1
ARHGAP10	308.524893	-1.28533594	0.16059174	-8.00374871	1.21E-15	4.87E-15	Down	ARHGAP10
GRIA1	6.06450851	-2.32397082	0.29040621	-8.00248324	1.22E-15	4.92E-15	Down	GRIA1
MYO15B	5455.69694	-1.11596699	0.13946513	-8.00176337	1.23E-15	4.95E-15	Down	MYO15B
RTKN2	291.044241	0.97821628	0.12225317	8.0015614	1.23E-15	4.95E-15	Up	RTKN2
TIFAB	23.5842955	-1.61510086	0.2018562	-8.00124467	1.23E-15	4.97E-15	Down	TIFAB
LHX5	5.20639775	3.77238982	0.4714814	8.00114242	1.23E-15	4.97E-15	Up	LHX5
COL6A5	9.34914006	-2.58086817	0.32263749	-7.99928178	1.25E-15	5.04E-15	Down	COL6A5
ARF6	5796.10772	-0.52985087	0.06623798	-7.99920003	1.25E-15	5.04E-15	Down	ARF6
TUBB	21382.8377	0.51876798	0.0648621	7.99801374	1.26E-15	5.09E-15	Up	TUBB
SYT4	13.307891	-3.18355328	0.39810256	-7.99681684	1.28E-15	5.14E-15	Down	SYT4
RSPO1	10.5600127	-2.05030311	0.25645696	-7.99472585	1.30E-15	5.23E-15	Down	RSPO1
EPS8	4441.29609	-0.62095876	0.07767845	-7.99396451	1.31E-15	5.26E-15	Down	EPS8
CAMK2A	15.786043	-1.73791965	0.21741514	-7.99355395	1.31E-15	5.28E-15	Down	CAMK2A
STT3B	9391.50353	0.62876931	0.07866335	7.99316725	1.32E-15	5.29E-15	Up	STT3B
SLC6A14	550.483199	2.22125382	0.27790672	7.99280368	1.32E-15	5.31E-15	Up	SLC6A14
NME2	32.9881596	1.26275606	0.15801745	7.99124441	1.34E-15	5.37E-15	Up	NME2
CD33	61.2634464	-1.26526662	0.158342	-7.99072017	1.34E-15	5.39E-15	Down	CD33
RPS19	44855.8399	0.86671559	0.10848245	7.98945432	1.36E-15	5.45E-15	Up	RPS19
EVI2B	300.601062	-1.43078894	0.17912047	-7.98785819	1.37E-15	5.52E-15	Down	EVI2B
MRPS36	785.198355	-0.67721937	0.08478681	-7.98731969	1.38E-15	5.54E-15	Down	MRPS36
ZNF556	23.727645	3.23132927	0.40458239	7.98682637	1.38E-15	5.56E-15	Up	ZNF556
LRP2	7.38604572	3.40972512	0.42696292	7.98599822	1.39E-15	5.60E-15	Up	LRP2
ADRA2C	183.158504	1.99378387	0.24977973	7.98216847	1.44E-15	5.77E-15	Up	ADRA2C
PAAF1	856.18211	0.6277108	0.07865356	7.98070444	1.46E-15	5.84E-15	Up	PAAF1
AICDA	4.66950008	-3.31053354	0.41490427	-7.97902986	1.47E-15	5.92E-15	Down	AICDA
MMP12	2145.27157	1.77445798	0.22242078	7.9779325	1.49E-15	5.97E-15	Up	MMP12
ZCCHC24	900.462549	-1.18702425	0.1487954	-7.97756024	1.49E-15	5.99E-15	Down	ZCCHC24

HPDL	733.410684	1.4281902	0.17907272	7.9754761	1.52E-15	6.09E-15	Up	HPDL
POU6F2	78.076602	3.59328796	0.45057402	7.9749116	1.52E-15	6.11E-15	Up	POU6F2
NEK8	265.134635	0.7901966	0.09908793	7.97470054	1.53E-15	6.12E-15	Up	NEK8
DAZAP1	4820.44005	0.50215626	0.06297073	7.97443912	1.53E-15	6.13E-15	Up	DAZAP1
AIF1	376.484307	-1.14556744	0.14365513	-7.97442748	1.53E-15	6.13E-15	Down	AIF1
LRP1B	5.28850052	-2.45055811	0.30741019	-7.97162292	1.57E-15	6.27E-15	Down	LRP1B
BARX2	265.536275	-1.86252923	0.23366812	-7.97083166	1.58E-15	6.31E-15	Down	BARX2
SASH3	416.405449	-1.18783565	0.14902876	-7.97051313	1.58E-15	6.33E-15	Down	SASH3
TNFSF13	556.933448	-0.94718962	0.11883927	-7.97034216	1.58E-15	6.33E-15	Down	TNFSF13
COL14A1	1476.70922	-1.49705721	0.18785231	-7.96933073	1.60E-15	6.38E-15	Down	COL14A1
TRAK1	3176.59457	-0.53210189	0.0668043	-7.96508444	1.65E-15	6.61E-15	Down	TRAK1
ZNHIT6	802.556263	0.53248219	0.06685259	7.96501911	1.65E-15	6.61E-15	Up	ZNHIT6
LGALS7B	5.41516303	2.81559081	0.35350325	7.96482294	1.65E-15	6.62E-15	Up	LGALS7B
FADS3	518.884998	1.02483506	0.12868394	7.96397021	1.67E-15	6.66E-15	Up	FADS3
TMEM237	413.944273	0.68293116	0.08578327	7.96112317	1.70E-15	6.81E-15	Up	TMEM237
MYO7A	390.300125	1.15248284	0.14476675	7.96096361	1.71E-15	6.82E-15	Up	MYO7A
ANKRD18A	73.890826	1.48331519	0.18634557	7.96002395	1.72E-15	6.87E-15	Up	ANKRD18A
CEP85	673.381245	0.57985503	0.07284865	7.95972185	1.72E-15	6.89E-15	Up	CEP85
EMG1	770.519821	0.6696304	0.08413215	7.95926913	1.73E-15	6.91E-15	Up	EMG1
PCSK4	126.648598	1.40306474	0.17635014	7.95613073	1.78E-15	7.09E-15	Up	PCSK4
HRC	19.5662319	-1.02983856	0.12947834	-7.95375173	1.81E-15	7.22E-15	Down	HRC
KCNG3	10.721525	-2.05929595	0.2589803	-7.95155433	1.84E-15	7.35E-15	Down	KCNG3
INPP5K	1060.52231	-0.67400497	0.08476681	-7.95128399	1.85E-15	7.36E-15	Down	INPP5K
VPS72	1564.04372	0.50398064	0.06338936	7.95055581	1.86E-15	7.40E-15	Up	VPS72
OSER1	2037.87036	0.83749772	0.1053406	7.95037944	1.86E-15	7.41E-15	Up	OSER1
ARHGEF11	2344.73677	-0.51241677	0.06445403	-7.95011259	1.86E-15	7.43E-15	Down	ARHGEF11
POC1A	705.575404	0.80974232	0.10185824	7.94969884	1.87E-15	7.45E-15	Up	POC1A
ZYG11B	977.146306	-0.71725587	0.09023747	-7.94853743	1.89E-15	7.52E-15	Down	ZYG11B
ADAMTSL4	291.329572	-1.28061896	0.1611361	-7.94743692	1.90E-15	7.58E-15	Down	ADAMTSL4
FAM219B	318.951633	-0.52770608	0.06641003	-7.9461807	1.92E-15	7.66E-15	Down	FAM219B
APEX1	5362.16598	0.59952009	0.07544877	7.94605518	1.93E-15	7.67E-15	Up	APEX1
CCL26	30.7284124	1.95097229	0.24561289	7.94328136	1.97E-15	7.84E-15	Up	CCL26
DMRT1	4.74649805	4.80092898	0.60445641	7.9425561	1.98E-15	7.88E-15	Up	DMRT1
NR2C1	743.999164	0.5514107	0.06943472	7.94142664	2.00E-15	7.95E-15	Up	NR2C1
RNF208	338.620929	1.20243083	0.15144851	7.9395355	2.03E-15	8.07E-15	Up	RNF208
TUBB6	1111.33597	-1.20941685	0.15234837	-7.93849559	2.05E-15	8.14E-15	Down	TUBB6
EXTL1	6.93273652	-1.74280333	0.21957663	-7.93710758	2.07E-15	8.23E-15	Down	EXTL1
KRT1	10.6664752	-2.39198165	0.30144127	-7.9351499	2.10E-15	8.36E-15	Down	KRT1
TRAF2	1183.81511	0.6609328	0.08329355	7.93498141	2.11E-15	8.37E-15	Up	TRAF2
RAC3	182.077151	1.29348775	0.16302147	7.93446255	2.11E-15	8.40E-15	Up	RAC3
TDO2	193.30363	1.79837004	0.22669747	7.93290725	2.14E-15	8.50E-15	Up	TDO2
GPN3	669.828421	0.58657072	0.0739453	7.9324946	2.15E-15	8.53E-15	Up	GPN3
EFCAB10	14.0842641	1.10699942	0.13957299	7.93132996	2.17E-15	8.61E-15	Up	EFCAB10
NTNG1	14.5979655	-2.82091354	0.3557411	-7.92968135	2.20E-15	8.72E-15	Down	NTNG1
CDC42EP4	2624.75565	-0.5850047	0.07378357	-7.92865857	2.22E-15	8.79E-15	Down	CDC42EP4
TBPL2	1.38710057	-2.18543839	0.27564323	-7.92850385	2.22E-15	8.80E-15	Down	TBPL2
EIF4A3	3895.84621	0.57933227	0.07307719	7.92767555	2.23E-15	8.86E-15	Up	EIF4A3
DCAF15	1427.03038	0.81922164	0.10338026	7.92435298	2.29E-15	9.10E-15	Up	DCAF15
TRAPPC5	104.807281	1.79668818	0.2267402	7.92399471	2.30E-15	9.12E-15	Up	TRAPPC5
SMARCD3	247.855911	-0.8736553	0.11025769	-7.92375832	2.30E-15	9.13E-15	Down	SMARCD3
OGDH	6454.45639	-0.59662162	0.07529809	-7.92346312	2.31E-15	9.15E-15	Down	OGDH
RPL8	101325.346	1.03212168	0.13030739	7.9206687	2.36E-15	9.36E-15	Up	RPL8
ZNF471	59.423912	-1.66265519	0.20992836	-7.92010768	2.37E-15	9.40E-15	Down	ZNF471
SCG3	28.6093351	-2.52977059	0.31941362	-7.92004615	2.37E-15	9.40E-15	Down	SCG3
MEOX1	73.1479134	-1.42885062	0.1804214	-7.91951873	2.38E-15	9.44E-15	Down	MEOX1
SLC10A4	9.8720374	-1.88892619	0.23856016	-7.91802882	2.41E-15	9.55E-15	Down	SLC10A4
RAB18	2028.03675	-0.55811011	0.07049229	-7.91732096	2.43E-15	9.60E-15	Down	RAB18
ZNF676	2.72262707	-2.43700779	0.30781194	-7.91719702	2.43E-15	9.61E-15	Down	ZNF676

EXOSC6	1163.73448	0.52186696	0.06591601	7.91715077	2.43E-15	9.61E-15	Up	EXOSC6
AKR1E2	92.5637126	1.46461057	0.18505522	7.91445152	2.48E-15	9.82E-15	Up	AKR1E2
INO80B	96.3939361	0.71629344	0.0905175	7.91331442	2.51E-15	9.91E-15	Up	INO80B
IMMP2L	369.920934	0.82538474	0.10431656	7.91230753	2.53E-15	9.99E-15	Up	IMMP2L
SCN1B	97.9075439	-1.14906378	0.14522912	-7.91207568	2.53E-15	1.00E-14	Down	SCN1B
GP1BA	31.3483942	-1.23613077	0.15629187	-7.90911752	2.59E-15	1.02E-14	Down	GP1BA
ZNF582	25.2458157	-1.31162526	0.16585457	-7.90828525	2.61E-15	1.03E-14	Down	ZNF582
LFNG	4086.83814	0.93960137	0.11881676	7.90798664	2.62E-15	1.03E-14	Up	LFNG
CACNB2	151.744989	-1.35228828	0.17101615	-7.907372	2.63E-15	1.04E-14	Down	CACNB2
WWTR1	993.086464	-1.27919442	0.16183826	-7.90415315	2.70E-15	1.06E-14	Down	WWTR1
CACNA1A	29.7602376	-1.34911872	0.17072706	-7.90219631	2.74E-15	1.08E-14	Down	CACNA1A
CYP4V2	843.70696	-0.8148329	0.10312186	-7.90165041	2.75E-15	1.09E-14	Down	CYP4V2
CYP4F3	701.673244	1.3375552	0.16928669	7.90112429	2.76E-15	1.09E-14	Up	CYP4F3
CYP26A1	13.3446249	2.65306663	0.3357856	7.90107331	2.77E-15	1.09E-14	Up	CYP26A1
UPF3A	1152.6871	0.75222654	0.09521972	7.89990279	2.79E-15	1.10E-14	Up	UPF3A
SLAMF1	82.272519	-1.22556146	0.15514499	-7.89945906	2.80E-15	1.10E-14	Down	SLAMF1
ATP2B2	4.81086206	-2.0687707	0.26190528	-7.89892719	2.81E-15	1.11E-14	Down	ATP2B2
TEX22	13.1390459	1.29117112	0.16349768	7.89718315	2.85E-15	1.12E-14	Up	TEX22
MEGF10	14.0680291	-1.85418744	0.23481392	-7.8964118	2.87E-15	1.13E-14	Down	MEGF10
DHX35	753.88349	0.60671626	0.07684594	7.89522833	2.90E-15	1.14E-14	Up	DHX35
C6orf47	1041.20536	-0.5000468	0.06336344	-7.89172477	2.98E-15	1.17E-14	Down	C6orf47
DCAF16	1374.69737	0.59122976	0.07493542	7.88985705	3.03E-15	1.19E-14	Up	DCAF16
TTC38	3501.59998	-0.86446664	0.10958065	-7.88886209	3.05E-15	1.20E-14	Down	TTC38
NUDT10	12.6733401	-1.89551334	0.24027703	-7.88886634	3.05E-15	1.20E-14	Down	NUDT10
MNX1	424.743693	0.87957771	0.11150253	7.88841028	3.06E-15	1.20E-14	Up	MNX1
RAD54L2	1003.4891	0.55748354	0.07068072	7.88734954	3.09E-15	1.21E-14	Up	RAD54L2
DCAF6	1927.03463	-0.5041173	0.06391987	-7.88670753	3.10E-15	1.22E-14	Down	DCAF6
ANKFY1	1817.69035	-0.5732495	0.07268652	-7.88660012	3.11E-15	1.22E-14	Down	ANKFY1
BARD1	494.363631	0.72712193	0.09220008	7.88634854	3.11E-15	1.22E-14	Up	BARD1
BOC	307.591948	-1.5761594	0.19986841	-7.88598548	3.12E-15	1.23E-14	Down	BOC
SLC9B2	275.241126	0.95185355	0.12070195	7.88598289	3.12E-15	1.23E-14	Up	SLC9B2
B4GALT1	5398.44029	-0.59324799	0.07523082	-7.88570437	3.13E-15	1.23E-14	Down	B4GALT1
PLK3	580.582552	0.79393516	0.10070703	7.88361204	3.18E-15	1.25E-14	Up	PLK3
C2CD4D	64.9281643	1.16748461	0.1481229	7.88186423	3.23E-15	1.27E-14	Up	C2CD4D
C10orf99	3177.11759	-1.85358378	0.23523094	-7.87984676	3.28E-15	1.29E-14	Down	C10orf99
MIEF2	375.764904	-0.6858393	0.08705309	-7.87840303	3.32E-15	1.30E-14	Down	MIEF2
GSC	4.60160428	2.27499252	0.28888016	7.87521196	3.40E-15	1.33E-14	Up	GSC
TES	4071.75422	-0.55938671	0.07104159	-7.87407367	3.43E-15	1.35E-14	Down	TES
RNF215	280.757442	0.65333552	0.08297473	7.87390993	3.44E-15	1.35E-14	Up	RNF215
LSM2	1774.70369	0.78942824	0.10025943	7.87385519	3.44E-15	1.35E-14	Up	LSM2
LIX1L	488.38722	-0.96958681	0.12316124	-7.87249942	3.48E-15	1.36E-14	Down	LIX1L
SLC24A4	14.2817446	-1.30422196	0.16573169	-7.86947834	3.56E-15	1.39E-14	Down	SLC24A4
PMPCA	2907.29647	0.6771145	0.08605294	7.86858101	3.59E-15	1.40E-14	Up	PMPCA
PSMA1	2432.5475	0.51057717	0.06491113	7.86578764	3.67E-15	1.44E-14	Up	PSMA1
ZMYND15	126.899778	1.0476641	0.13319412	7.86569328	3.67E-15	1.44E-14	Up	ZMYND15
CHRNA5	149.98326	0.91229836	0.11599239	7.86515701	3.69E-15	1.44E-14	Up	CHRNA5
CYP39A1	367.020827	1.22608456	0.15589076	7.86502376	3.69E-15	1.44E-14	Up	CYP39A1
ZNF526	596.302089	0.52943178	0.06732037	7.86436176	3.71E-15	1.45E-14	Up	ZNF526
TCTN2	437.842011	0.60334769	0.07673049	7.86320637	3.74E-15	1.46E-14	Up	TCTN2
NCOR1	4171.14097	-0.65121531	0.0828534	-7.85985052	3.85E-15	1.50E-14	Down	NCOR1
GABARAPL1	933.095876	-0.88367689	0.11249955	-7.8549374	4.00E-15	1.56E-14	Down	GABARAPL1
HSPBAP1	285.584565	0.63936169	0.08143501	7.85118983	4.12E-15	1.61E-14	Up	HSPBAP1
CRLS1	2761.57162	0.7718315	0.09831447	7.85063987	4.14E-15	1.62E-14	Up	CRLS1
CDO1	20.2152068	-1.92245686	0.24491389	-7.84952151	4.18E-15	1.63E-14	Down	CDO1
GLO1	5006.01377	0.72005509	0.09173327	7.84944348	4.18E-15	1.63E-14	Up	GLO1
MAN1C1	260.164495	-1.10661483	0.14099535	-7.84859096	4.21E-15	1.64E-14	Down	MAN1C1
APC	813.525455	-0.91723334	0.1168922	-7.84683128	4.27E-15	1.67E-14	Down	APC
SOBP	155.182729	-1.3005718	0.16574784	-7.84668909	4.27E-15	1.67E-14	Down	SOBP

SNX30	1412.93195	-0.66621937	0.0849109	-7.84609908	4.29E-15	1.67E-14	Down	SNX30
PROZ	12.0846104	1.67028066	0.2128827	7.84601424	4.29E-15	1.68E-14	Up	PROZ
ADRA2A	544.615304	-1.65992303	0.21160647	-7.8443869	4.35E-15	1.70E-14	Down	ADRA2A
DLGAP2	3.08104465	-1.99903089	0.25493629	-7.84129603	4.46E-15	1.74E-14	Down	DLGAP2
PYCR2	1883.4579	0.57162596	0.0729062	7.84056695	4.49E-15	1.75E-14	Up	PYCR2
PGP	1087.00805	0.97888651	0.12485322	7.84029866	4.49E-15	1.75E-14	Up	PGP
MIPOL1	161.905203	-1.36843995	0.17455882	-7.83942041	4.53E-15	1.76E-14	Down	MIPOL1
VSIG8	14.3260542	2.07136827	0.26423187	7.83920675	4.53E-15	1.77E-14	Up	VSIG8
MYO1E	3534.67935	-0.59549847	0.07596867	-7.83873726	4.55E-15	1.77E-14	Down	MYO1E
B4GALNT3	2648.7978	-0.95563165	0.12192766	-7.83769369	4.59E-15	1.79E-14	Down	B4GALNT3
PRADC1	744.337554	-0.90671641	0.11569144	-7.83736822	4.60E-15	1.79E-14	Down	PRADC1
HSD17B10	3820.13427	0.88063007	0.11239796	7.83492922	4.69E-15	1.83E-14	Up	HSD17B10
UBD	529.367877	1.8275532	0.23325968	7.83484407	4.69E-15	1.83E-14	Up	UBD
KCNIP1	4.10804007	-2.02850481	0.25893173	-7.83412997	4.72E-15	1.84E-14	Down	KCNIP1
TCF19	1093.8841	0.62931067	0.08035904	7.83123636	4.83E-15	1.88E-14	Up	TCF19
ACP2	1414.47313	-0.61067087	0.07799104	-7.83001343	4.88E-15	1.90E-14	Down	ACP2
PDE6G	16.6771421	-1.29057895	0.16484219	-7.82917873	4.91E-15	1.91E-14	Down	PDE6G
SIRT4	20.1376902	-0.93115475	0.11894356	-7.82854267	4.94E-15	1.92E-14	Down	SIRT4
KRT37	2.80553191	4.43797841	0.5669351	7.82801845	4.96E-15	1.93E-14	Up	KRT37
ARRDC1	3400.40748	0.84292786	0.10769965	7.82665384	5.01E-15	1.95E-14	Up	ARRDC1
HCAR3	31.8852452	2.73065779	0.34889465	7.82659695	5.01E-15	1.95E-14	Up	HCAR3
MANEAL	742.751595	1.0522274	0.134444	7.82651083	5.02E-15	1.95E-14	Up	MANEAL
ACRV1	11.6014241	1.19779565	0.15304676	7.82633768	5.02E-15	1.95E-14	Up	ACRV1
MGAT4C	5.0951935	-3.10761124	0.39711254	-7.82551769	5.06E-15	1.96E-14	Down	MGAT4C
SLC6A1	33.0251396	1.23760068	0.15818186	7.82391	5.12E-15	1.99E-14	Up	SLC6A1
EBI3	59.7345728	-1.27874243	0.16347035	-7.82247329	5.18E-15	2.01E-14	Down	EBI3
TNK2	1982.80875	0.84010482	0.10739904	7.82227506	5.19E-15	2.01E-14	Up	TNK2
SLC3A1	525.742101	-1.59969702	0.20451649	-7.82184857	5.21E-15	2.02E-14	Down	SLC3A1
VWA3B	25.2230207	-1.47753878	0.18894611	-7.81989511	5.29E-15	2.05E-14	Down	VWA3B
SFXN1	1985.27869	-0.62569892	0.0800147	-7.81979941	5.29E-15	2.05E-14	Down	SFXN1
CLDN12	3151.91799	0.72954162	0.09332727	7.81702536	5.41E-15	2.10E-14	Up	CLDN12
PPM1L	511.548983	-0.96392436	0.12331205	-7.81695159	5.41E-15	2.10E-14	Down	PPM1L
MANF	2765.56652	0.68480556	0.08760962	7.81655682	5.43E-15	2.10E-14	Up	MANF
C15orf62	81.9869861	0.90419638	0.11570477	7.81468569	5.51E-15	2.13E-14	Up	C15orf62
PDGFRA	1154.33118	-1.16624906	0.14924596	-7.81427582	5.53E-15	2.14E-14	Down	PDGFRA
ITGB1BP2	30.3791549	-1.20226602	0.15387022	-7.81350711	5.56E-15	2.15E-14	Down	ITGB1BP2
AKNAD1	15.2899625	1.6740198	0.21425559	7.81319066	5.58E-15	2.16E-14	Up	AKNAD1
THOC6	1331.89935	0.76333476	0.0977003	7.81302409	5.58E-15	2.16E-14	Up	THOC6
RAPGEF1	2775.12744	-0.51348838	0.06572416	-7.81278021	5.59E-15	2.16E-14	Down	RAPGEF1
NUDT11	25.9884554	-1.48535345	0.19021907	-7.80864639	5.78E-15	2.23E-14	Down	NUDT11
PPP1R12A	1958.64383	-0.71689509	0.09181659	-7.807904	5.81E-15	2.25E-14	Down	PPP1R12A
TMCO6	436.384517	0.60766855	0.07783649	7.80698829	5.86E-15	2.26E-14	Up	TMCO6
CYP24A1	41.9953261	2.58728179	0.33147273	7.80541361	5.93E-15	2.29E-14	Up	CYP24A1
SNX29	533.922162	-0.67202225	0.08610521	-7.80466405	5.97E-15	2.30E-14	Down	SNX29
ULK4	152.362891	0.87893107	0.11262029	7.80437613	5.98E-15	2.31E-14	Up	ULK4
TOX	372.088895	-1.54542268	0.19807044	-7.80238935	6.07E-15	2.35E-14	Down	TOX
PRMT5	2247.30493	0.63762176	0.08173034	7.80153086	6.12E-15	2.36E-14	Up	PRMT5
TMEM54	7916.16036	-1.08283759	0.1387977	-7.80155258	6.12E-15	2.36E-14	Down	TMEM54
SIX3	9.91915634	3.71447812	0.47612597	7.80146084	6.12E-15	2.36E-14	Up	SIX3
TFCP2L1	2519.37573	-1.24249426	0.15927188	-7.80109009	6.14E-15	2.37E-14	Down	TFCP2L1
GGH	4765.13228	1.2064792	0.15465925	7.80088632	6.15E-15	2.37E-14	Up	GGH
RPRM	20.4976875	-2.27579199	0.29180436	-7.79903365	6.24E-15	2.41E-14	Down	RPRM
HMBS	1409.37636	0.75374195	0.09665276	7.79845202	6.27E-15	2.42E-14	Up	HMBS
GIGYF1	2253.00395	0.73500066	0.09427571	7.79628894	6.38E-15	2.46E-14	Up	GIGYF1
MDC1	1490.52785	0.62487819	0.08015918	7.79546615	6.42E-15	2.47E-14	Up	MDC1
PROCR	1422.16165	1.05562504	0.13545837	7.79298508	6.54E-15	2.52E-14	Up	PROCR
SNX4	1260.04549	-0.54168288	0.06951509	-7.79230692	6.58E-15	2.53E-14	Down	SNX4
CELSR2	746.117262	0.92559019	0.11880021	7.79114936	6.64E-15	2.56E-14	Up	CELSR2

TMOD2	323.893747	-1.253761	0.16093453	-7.79050316	6.67E-15	2.57E-14	Down	TMOD2
IZUMO2	17.3168665	2.93596976	0.3770123	7.78746415	6.84E-15	2.63E-14	Up	IZUMO2
MAP7D2	397.376069	2.37920869	0.30553376	7.78705663	6.86E-15	2.64E-14	Up	MAP7D2
TRMT61A	1410.60079	0.92060391	0.11822341	7.78698469	6.86E-15	2.64E-14	Up	TRMT61A
LPAR2	846.285741	0.83537892	0.10728852	7.78628418	6.90E-15	2.65E-14	Up	LPAR2
RGS18	44.5450368	-1.43868013	0.184771	-7.78628733	6.90E-15	2.65E-14	Down	RGS18
PTBP2	332.0643	-0.96593168	0.12406092	-7.78594617	6.92E-15	2.66E-14	Down	PTBP2
TMCO1	3632.35977	-0.60690282	0.07794988	-7.78580851	6.93E-15	2.66E-14	Down	TMCO1
C4orf46	428.032952	0.65148777	0.08368701	7.78481381	6.98E-15	2.68E-14	Up	C4orf46
TRAPPC10	983.24382	-0.5671488	0.07287375	-7.782621	7.10E-15	2.73E-14	Down	TRAPPC10
TMEM231	375.821942	0.6824449	0.08771941	7.77986212	7.26E-15	2.79E-14	Up	TMEM231
NPIPA3	9.96396678	2.10830091	0.27107982	7.77741749	7.40E-15	2.84E-14	Up	NPIPA3
NR4A3	205.11511	-1.72178058	0.22143342	-7.7756131	7.51E-15	2.88E-14	Down	NR4A3
PREX2	111.030549	-1.3207908	0.16990851	-7.77354096	7.63E-15	2.93E-14	Down	PREX2
TAGAP	196.10881	-1.32825215	0.17088375	-7.77284053	7.67E-15	2.94E-14	Down	TAGAP
STAC	26.2370366	-1.87668481	0.24146902	-7.77194872	7.73E-15	2.96E-14	Down	STAC
ZNF304	121.873275	-1.38876352	0.1787106	-7.77101917	7.79E-15	2.98E-14	Down	ZNF304
ITPKA	536.608826	-1.34175777	0.17272391	-7.76822276	7.96E-15	3.05E-14	Down	ITPKA
ARHGAP11B	282.836594	0.89298426	0.11498752	7.7659232	8.11E-15	3.11E-14	Up	ARHGAP11B
ADRB3	4.07478836	-2.57237334	0.33127501	-7.76506897	8.16E-15	3.13E-14	Down	ADRB3
KLK15	52.8020211	-1.89728658	0.24435991	-7.76431202	8.21E-15	3.14E-14	Down	KLK15
EIF6	9100.87379	0.86415046	0.11130438	7.76384925	8.24E-15	3.16E-14	Up	EIF6
FKBP1B	76.195415	-1.44196088	0.18573634	-7.76348277	8.26E-15	3.16E-14	Down	FKBP1B
L3MBTL1	273.436567	1.37120062	0.1766439	7.76251336	8.33E-15	3.19E-14	Up	L3MBTL1
FGF8	2.79490036	2.68627718	0.34615942	7.76023142	8.48E-15	3.24E-14	Up	FGF8
RSAD2	408.154076	-1.5631607	0.20143516	-7.76011829	8.49E-15	3.25E-14	Down	RSAD2
ZFP3	273.076101	-0.99514401	0.12826401	-7.75855983	8.59E-15	3.29E-14	Down	ZFP3
VSIG4	434.280481	-1.5770259	0.20327183	-7.7582118	8.61E-15	3.29E-14	Down	VSIG4
EEF2KMT	430.463328	0.62193574	0.08016966	7.75774422	8.65E-15	3.31E-14	Up	EEF2KMT
SLC2A8	1686.00516	1.04040235	0.13411404	7.75759482	8.66E-15	3.31E-14	Up	SLC2A8
USP35	251.302162	0.64250876	0.08285132	7.7549613	8.84E-15	3.38E-14	Up	USP35
C1QTNF3	175.965301	-1.29567402	0.16707917	-7.75485053	8.84E-15	3.38E-14	Down	C1QTNF3
MRPL4	2996.26436	0.83603383	0.1078165	7.75422875	8.89E-15	3.40E-14	Up	MRPL4
THSD4	755.879371	-0.96306227	0.12419796	-7.75425191	8.89E-15	3.40E-14	Down	THSD4
ABCA5	636.506076	-1.14675452	0.1479251	-7.7522646	9.03E-15	3.45E-14	Down	ABCA5
TACR1	19.9346957	-2.15774597	0.2783449	-7.75205862	9.04E-15	3.45E-14	Down	TACR1
PTPDC1	314.935647	0.63488691	0.08191259	7.75078511	9.13E-15	3.49E-14	Up	PTPDC1
ANKRD33	3.79888874	3.03121717	0.39116898	7.74912462	9.25E-15	3.53E-14	Up	ANKRD33
CYP11A1	7.39446286	-1.80133078	0.23247895	-7.7483607	9.31E-15	3.55E-14	Down	CYP11A1
CD52	388.774629	-1.28020084	0.16525477	-7.7468315	9.42E-15	3.59E-14	Down	CD52
REP15	65.5575135	-2.23675471	0.28874794	-7.74639185	9.45E-15	3.61E-14	Down	REP15
GMEB2	1232.86534	0.64977756	0.08388399	7.7461451	9.47E-15	3.61E-14	Up	GMEB2
GPATCH2	539.16844	0.62036743	0.0801244	7.74255298	9.74E-15	3.71E-14	Up	GPATCH2
ANAPC1	968.782817	0.62146458	0.08028373	7.74085296	9.88E-15	3.76E-14	Up	ANAPC1
MRPL37	3896.99159	0.60889871	0.07866229	7.74066882	9.89E-15	3.77E-14	Up	MRPL37
HSP90AA1	39037.6985	0.63473378	0.08200994	7.7397175	9.96E-15	3.80E-14	Up	HSP90AA1
TCTN1	571.676391	0.65620694	0.08479227	7.73899529	1.00E-14	3.82E-14	Up	TCTN1
TNFSF11	119.278044	1.37852441	0.1781301	7.73886263	1.00E-14	3.82E-14	Up	TNFSF11
CLVS2	5.59378049	-3.05960957	0.39546227	-7.73679257	1.02E-14	3.88E-14	Down	CLVS2
MAPKAPK3	2472.41067	0.59777488	0.07726541	7.73664322	1.02E-14	3.89E-14	Up	MAPKAPK3
MAK16	725.987126	0.74214517	0.09592803	7.73647876	1.02E-14	3.89E-14	Up	MAK16
BTBD7	1197.35034	-0.55756706	0.07207185	-7.73626656	1.02E-14	3.90E-14	Down	BTBD7
KBTBD6	501.979087	0.66710218	0.08623455	7.735904	1.03E-14	3.91E-14	Up	KBTBD6
GOLGA8B	277.939041	1.42082645	0.18368818	7.73499098	1.03E-14	3.93E-14	Up	GOLGA8B
CD96	151.245511	-1.31266043	0.16973249	-7.73370147	1.04E-14	3.97E-14	Down	CD96
TBX6	65.1286759	1.04017075	0.13450854	7.73312044	1.05E-14	3.99E-14	Up	TBX6
RHOJ	237.526691	-1.04184188	0.13473881	-7.73230721	1.06E-14	4.01E-14	Down	RHOJ
FBRSL1	3172.30114	0.73725591	0.09534782	7.73227827	1.06E-14	4.01E-14	Up	FBRSL1

F8	170.151412	-1.03230545	0.1335259	-7.73112505	1.07E-14	4.05E-14	Down	F8
RARG	1261.11244	0.87153697	0.11273862	7.73059807	1.07E-14	4.07E-14	Up	RARG
NASP	3491.44078	0.5589195	0.07231917	7.72851116	1.09E-14	4.13E-14	Up	NASP
MYBPC3	9.48457378	1.40985107	0.18251164	7.7247185	1.12E-14	4.25E-14	Up	MYBPC3
RGR	12.3906418	3.60176742	0.46628657	7.7243645	1.12E-14	4.27E-14	Up	RGR
PHYHD1	75.9895867	-1.40084441	0.18136367	-7.72395286	1.13E-14	4.28E-14	Down	PHYHD1
ZFC3H1	1365.89469	0.84855676	0.1098656	7.72358894	1.13E-14	4.29E-14	Up	ZFC3H1
PLGRKT	943.442307	-0.72069744	0.09335456	-7.72000225	1.16E-14	4.41E-14	Down	PLGRKT
XRN2	4082.29212	0.65076641	0.08429696	7.71992773	1.16E-14	4.41E-14	Up	XRN2
IFNE	4.20542947	4.18954301	0.54272712	7.71942823	1.17E-14	4.43E-14	Up	IFNE
WDR18	2003.44799	0.94719279	0.12270662	7.71916633	1.17E-14	4.44E-14	Up	WDR18
HSF1	4539.17017	0.78880641	0.10218927	7.71907305	1.17E-14	4.44E-14	Up	HSF1
NODAL	15.388691	2.06097016	0.26706324	7.71716165	1.19E-14	4.51E-14	Up	NODAL
PLOD1	4465.24958	0.67586705	0.087608	7.7146728	1.21E-14	4.59E-14	Up	PLOD1
FAM189B	1419.76534	0.68006093	0.08818733	7.71154916	1.24E-14	4.71E-14	Up	FAM189B
ARPP21	2.54192606	-3.12903245	0.40577473	-7.71125508	1.25E-14	4.72E-14	Down	ARPP21
USP38	1413.46584	-0.56320237	0.07306886	-7.70782993	1.28E-14	4.84E-14	Down	USP38
ADRA1A	4.608514	-2.42354848	0.31449321	-7.70620281	1.30E-14	4.90E-14	Down	ADRA1A
IGFL3	7.28001537	3.2537733	0.42222778	7.70620374	1.30E-14	4.90E-14	Up	IGFL3
CSF1R	1204.88283	-1.27585681	0.16565779	-7.70176146	1.34E-14	5.07E-14	Down	CSF1R
PBX3	443.554393	-0.8878745	0.115286	-7.7014948	1.34E-14	5.08E-14	Down	PBX3
ZNF581	891.506115	0.84375309	0.10956628	7.70084662	1.35E-14	5.11E-14	Up	ZNF581
ZNF418	19.6787357	-1.41402865	0.18364187	-7.69992529	1.36E-14	5.14E-14	Down	ZNF418
DUOXA2	1196.15034	2.46311247	0.31992213	7.69909999	1.37E-14	5.18E-14	Up	DUOXA2
SLC30A8	2.67829181	-2.5301778	0.32863314	-7.6990952	1.37E-14	5.18E-14	Down	SLC30A8
RDH10	1698.14071	0.71176578	0.09245501	7.69850935	1.38E-14	5.20E-14	Up	RDH10
GSTM4	1110.54847	-0.88444972	0.11488674	-7.69844923	1.38E-14	5.20E-14	Down	GSTM4
AKAP13	3539.54537	-0.76911491	0.09990742	-7.69827597	1.38E-14	5.21E-14	Down	AKAP13
PLA2G4B	15.7651226	1.26821215	0.1647431	7.69812013	1.38E-14	5.21E-14	Up	PLA2G4B
ING1	910.995019	0.53046189	0.06891648	7.69717062	1.39E-14	5.25E-14	Up	ING1
GRIN3B	11.3922324	1.73555372	0.22553659	7.69522027	1.41E-14	5.33E-14	Up	GRIN3B
SYNE4	331.393141	1.70630144	0.22184883	7.6912797	1.46E-14	5.49E-14	Up	SYNE4
MEGF11	39.290027	2.09231845	0.27222138	7.68609145	1.52E-14	5.72E-14	Up	MEGF11
XIRP1	22.8002947	2.03082639	0.26422251	7.68604619	1.52E-14	5.72E-14	Up	XIRP1
TMEM52B	11.4911974	1.79296061	0.23336769	7.6829857	1.55E-14	5.86E-14	Up	TMEM52B
PGK1	23091.9212	0.70859677	0.09223149	7.68280746	1.56E-14	5.86E-14	Up	PGK1
AARSD1	144.703669	0.72954339	0.09496346	7.68235939	1.56E-14	5.88E-14	Up	AARSD1
HSPA2	1048.7758	-1.61238786	0.20998226	-7.67868611	1.61E-14	6.05E-14	Down	HSPA2
RPL37	27961.812	0.70539036	0.09190035	7.67560013	1.65E-14	6.20E-14	Up	RPL37
DNAJC9	735.659013	0.50696161	0.06605353	7.67501124	1.65E-14	6.22E-14	Up	DNAJC9
RPL13	61972.9432	0.93837713	0.12228077	7.67395529	1.67E-14	6.27E-14	Up	RPL13
MPLKIP	1309.47363	0.61059734	0.07958511	7.67225628	1.69E-14	6.35E-14	Up	MPLKIP
PNKP	1282.25229	0.64452867	0.08404376	7.66896472	1.73E-14	6.52E-14	Up	PNKP
RPL28	39099.2118	0.97510066	0.12715649	7.66850856	1.74E-14	6.54E-14	Up	RPL28
LRRC69	26.1329795	1.32414862	0.17274024	7.66554794	1.78E-14	6.69E-14	Up	LRRC69
SIGLEC16	12.4730943	-1.28699153	0.16791195	-7.66468093	1.79E-14	6.74E-14	Down	SIGLEC16
LIN9	353.554721	0.68078538	0.08884546	7.66257922	1.82E-14	6.85E-14	Up	LIN9
PTPRU	623.217055	1.70239608	0.22217596	7.66237764	1.83E-14	6.85E-14	Up	PTPRU
P2RY10	42.2721378	-1.51264124	0.1974141	-7.66227547	1.83E-14	6.86E-14	Down	P2RY10
NPR2	266.406646	-0.74366395	0.09706072	-7.66184244	1.83E-14	6.88E-14	Down	NPR2
POU6F1	150.122784	-0.82184364	0.10726863	-7.66154707	1.84E-14	6.89E-14	Down	POU6F1
CEP41	437.357507	0.64863864	0.08467806	7.66005569	1.86E-14	6.97E-14	Up	CEP41
CP	157.659172	-2.14986691	0.28067927	-7.65951427	1.87E-14	7.00E-14	Down	CP
FEM1C	1345.49833	-0.72400221	0.09454029	-7.65813384	1.89E-14	7.08E-14	Down	FEM1C
MS4A7	394.043126	-1.3937094	0.18199243	-7.65806267	1.89E-14	7.08E-14	Down	MS4A7
PLA2G10	130.090412	-1.39478822	0.18215037	-7.65734509	1.90E-14	7.12E-14	Down	PLA2G10
CPS1	282.390427	2.58491394	0.33764548	7.65570434	1.92E-14	7.21E-14	Up	CPS1
GADD45GIP1	2246.03651	0.9191653	0.12007046	7.65521589	1.93E-14	7.23E-14	Up	GADD45GIP1

GTF2E2	1195.78607	0.69138275	0.09032256	7.65459693	1.94E-14	7.27E-14	Up	GTF2E2
DMKN	610.443952	1.70365579	0.22262799	7.65247782	1.97E-14	7.39E-14	Up	DMKN
BRINP3	66.8690855	-3.33600698	0.43596408	-7.65202251	1.98E-14	7.41E-14	Down	BRINP3
SPINT1	8995.05869	-0.72920706	0.09530078	-7.65163792	1.98E-14	7.43E-14	Down	SPINT1
SCIMP	97.1435135	-1.2933222	0.16904903	-7.65057429	2.00E-14	7.49E-14	Down	SCIMP
TK1	3023.1828	0.92411827	0.1208179	7.64885193	2.03E-14	7.59E-14	Up	TK1
FDCSP	77.374176	-2.8194787	0.36866624	-7.64778104	2.04E-14	7.65E-14	Down	FDCSP
CAMKK1	512.484687	0.72269294	0.09449964	7.64757321	2.05E-14	7.66E-14	Up	CAMKK1
MYO18B	13.3775377	2.45029458	0.32049108	7.64543762	2.08E-14	7.79E-14	Up	MYO18B
MYH7B	171.91931	1.7705529	0.23172494	7.64075247	2.16E-14	8.07E-14	Up	MYH7B
GZMA	220.92956	-1.53376495	0.20078083	-7.63900082	2.19E-14	8.18E-14	Down	GZMA
FBXW9	515.578936	0.85255247	0.11160668	7.63890178	2.19E-14	8.19E-14	Up	FBXW9
ZNF572	120.498692	0.98172954	0.12853668	7.63773866	2.21E-14	8.26E-14	Up	ZNF572
CREBL2	1572.12147	-0.57240168	0.07494421	-7.63770437	2.21E-14	8.26E-14	Down	CREBL2
LGALS9B	123.095739	-1.99638385	0.26146904	-7.63525911	2.25E-14	8.42E-14	Down	LGALS9B
ANKRD23	28.215897	0.96579238	0.12649743	7.63487779	2.26E-14	8.44E-14	Up	ANKRD23
B4GALT7	1262.43361	0.77130254	0.10104662	7.63313516	2.29E-14	8.55E-14	Up	B4GALT7
ILDR1	590.307662	-0.74150026	0.09715054	-7.63248714	2.30E-14	8.59E-14	Down	ILDR1
ZNF454	8.60126894	-1.36047975	0.17825626	-7.63215703	2.31E-14	8.61E-14	Down	ZNF454
MEGF9	671.711545	-0.63748643	0.08353527	-7.63134448	2.32E-14	8.67E-14	Down	MEGF9
ARHGAP11A	1149.38681	0.84067905	0.11016533	7.63106744	2.33E-14	8.68E-14	Up	ARHGAP11A
YEATS4	773.7957	0.68948701	0.09035349	7.63099513	2.33E-14	8.69E-14	Up	YEATS4
FCN3	35.6784915	1.13506904	0.1488008	7.62811125	2.38E-14	8.88E-14	Up	FCN3
EFEMP1	1162.98081	-1.43943827	0.18870513	-7.6279764	2.38E-14	8.89E-14	Down	EFEMP1
ZNF106	2073.21896	-0.62651459	0.08215902	-7.6256335	2.43E-14	9.05E-14	Down	ZNF106
TACC2	3101.28312	-0.57039352	0.07482764	-7.62276471	2.48E-14	9.25E-14	Down	TACC2
ABHD17C	3776.38261	-0.56891012	0.07463462	-7.62260336	2.49E-14	9.26E-14	Down	ABHD17C
CYP19A1	20.2012983	1.83623716	0.24092312	7.62167255	2.50E-14	9.33E-14	Up	CYP19A1
SLC29A2	1330.70228	0.74925043	0.09832939	7.61980127	2.54E-14	9.46E-14	Up	SLC29A2
ZNF8	306.714941	0.73486029	0.09644181	7.61972685	2.54E-14	9.46E-14	Up	ZNF8
FAM110A	661.929315	0.82718087	0.1085669	7.61908889	2.55E-14	9.51E-14	Up	FAM110A
IRX2	216.660934	2.8196057	0.37008196	7.61886821	2.56E-14	9.52E-14	Up	IRX2
IGF1	32.2110304	-1.80672906	0.23714374	-7.61870866	2.56E-14	9.53E-14	Down	IGF1
MAGEH1	265.275108	-1.03481959	0.13583673	-7.61811345	2.57E-14	9.57E-14	Down	MAGEH1
RERE	4480.43116	-0.59624155	0.07826728	-7.61801771	2.58E-14	9.58E-14	Down	RERE
SLC30A2	133.212919	2.35116558	0.30864561	7.61768674	2.58E-14	9.60E-14	Up	SLC30A2
LOXL4	88.7460674	-1.54932887	0.20341614	-7.6165484	2.61E-14	9.68E-14	Down	LOXL4
FAM91A1	2368.0621	0.71460906	0.09383871	7.61529115	2.63E-14	9.77E-14	Up	FAM91A1
MRPS26	2207.73037	0.91700371	0.12043541	7.61407065	2.66E-14	9.86E-14	Up	MRPS26
SLC25A39	8617.02638	0.76630965	0.10065295	7.61338498	2.67E-14	9.91E-14	Up	SLC25A39
UEVLD	944.626781	-0.54705141	0.0718673	-7.61196544	2.70E-14	1.00E-13	Down	UEVLD
EGR4	11.9125544	2.23056178	0.29306372	7.61118348	2.72E-14	1.01E-13	Up	EGR4
HBE1	15.0665588	5.66548342	0.74437479	7.61106304	2.72E-14	1.01E-13	Up	HBE1
ERF	2096.30544	0.63733062	0.08378156	7.60705149	2.80E-14	1.04E-13	Up	ERF
SLC25A18	18.5722163	-0.67313941	0.08849078	-7.60688718	2.81E-14	1.04E-13	Down	SLC25A18
SYN2	32.2098312	-1.75416341	0.23062269	-7.60620487	2.82E-14	1.05E-13	Down	SYN2
PIP5K1C	1743.82052	-0.61168253	0.08042415	-7.60570743	2.83E-14	1.05E-13	Down	PIP5K1C
GPR84	46.3351162	1.50765611	0.19830687	7.60264175	2.90E-14	1.08E-13	Up	GPR84
TMIGD2	25.4147135	-1.39374548	0.1833356	-7.6021541	2.91E-14	1.08E-13	Down	TMIGD2
PDIA2	69.4148088	1.87565021	0.24674603	7.6015417	2.93E-14	1.08E-13	Up	PDIA2
DNAH17	92.9266717	0.96211772	0.12657536	7.60114575	2.94E-14	1.09E-13	Up	DNAH17
TRMT2B	1024.72585	0.55897781	0.07353869	7.60113901	2.94E-14	1.09E-13	Up	TRMT2B
STXBP1	1375.21161	1.10945472	0.1459696	7.60058766	2.95E-14	1.09E-13	Up	STXBP1
LY6G5C	88.3681601	0.77180439	0.10154885	7.6003261	2.95E-14	1.09E-13	Up	LY6G5C
PLCB4	5058.15903	1.51565963	0.19943192	7.59988501	2.96E-14	1.10E-13	Up	PLCB4
ODAM	133.281706	2.23292687	0.29395691	7.59610275	3.05E-14	1.13E-13	Up	ODAM
CIDEB	6.00613906	-1.60436472	0.21125709	-7.59437089	3.09E-14	1.14E-13	Down	CIDEB
GTPBP10	1000.30523	0.63508271	0.0836319	7.59378565	3.11E-14	1.15E-13	Up	GTPBP10

MCF2L2	28.0451871	-1.05169135	0.13850442	-7.59319719	3.12E-14	1.15E-13	Down	MCF2L2
ZNF282	1651.17747	0.53967483	0.07107732	7.59278537	3.13E-14	1.16E-13	Up	ZNF282
LCN15	1272.44752	3.39662421	0.44744906	7.59108576	3.17E-14	1.17E-13	Up	LCN15
B3GNT4	45.8100169	1.26658014	0.16685386	7.59095523	3.18E-14	1.17E-13	Up	B3GNT4
RASSF1	1099.19805	0.60118331	0.07921937	7.5888422	3.23E-14	1.19E-13	Up	RASSF1
OSTM1	755.508914	-0.64837122	0.08544279	-7.5883671	3.24E-14	1.20E-13	Down	OSTM1
ZSCAN18	296.872365	-1.38250116	0.18221424	-7.58722902	3.27E-14	1.21E-13	Down	ZSCAN18
APBB1IP	211.478819	-1.22100012	0.16097125	-7.5852063	3.32E-14	1.23E-13	Down	APBB1IP
GCKR	6.28336624	1.9308709	0.25456904	7.58486133	3.33E-14	1.23E-13	Up	GCKR
REN	87.6890222	2.44369568	0.32219242	7.58458462	3.34E-14	1.23E-13	Up	REN
PLEKHF2	987.507194	-0.65704681	0.08663026	-7.58449586	3.34E-14	1.23E-13	Down	PLEKHF2
TNNT3	4.69726367	-2.39509187	0.31583711	-7.58331359	3.37E-14	1.24E-13	Down	TNNT3
TTC7A	1342.07011	-0.61968625	0.08172973	-7.58213979	3.40E-14	1.25E-13	Down	TTC7A
CYLD	1090.76259	-0.68961621	0.0909609	-7.58145751	3.42E-14	1.26E-13	Down	CYLD
FBXO30	671.152826	-0.63688462	0.08403541	-7.57876536	3.49E-14	1.29E-13	Down	FBXO30
IL5RA	8.27813565	-1.80700359	0.23844538	-7.57827043	3.50E-14	1.29E-13	Down	IL5RA
RPL18	44437.0993	0.84750561	0.11185795	7.57662397	3.55E-14	1.31E-13	Up	RPL18
GOLGA8R	3.40509465	-1.3491046	0.17806487	-7.57647816	3.55E-14	1.31E-13	Down	GOLGA8R
SLC29A3	544.113395	0.64734796	0.08545314	7.57547311	3.58E-14	1.32E-13	Up	SLC29A3
SCRIB	5619.19241	0.86342555	0.11399191	7.57444569	3.61E-14	1.33E-13	Up	SCRIB
RNASE6	318.343967	-0.86710338	0.11448195	-7.57414906	3.61E-14	1.33E-13	Down	RNASE6
OTOA	6.2244601	-1.32983227	0.17557918	-7.57397462	3.62E-14	1.33E-13	Down	OTOA
DCBLD1	871.36875	0.60732472	0.08019789	7.57282668	3.65E-14	1.34E-13	Up	DCBLD1
MNAT1	347.507778	0.66699374	0.0881323	7.56809673	3.79E-14	1.39E-13	Up	MNAT1
SMLR1	4.26663481	2.52520285	0.33379785	7.56506633	3.88E-14	1.43E-13	Up	SMLR1
LACTB2	1063.84439	0.89516027	0.1183327	7.56477501	3.89E-14	1.43E-13	Up	LACTB2
TRIP6	2500.45995	1.17639747	0.15553365	7.56362007	3.92E-14	1.44E-13	Up	TRIP6
SF3A2	3115.61507	0.66709735	0.088208	7.56277623	3.95E-14	1.45E-13	Up	SF3A2
C9orf57	2.16191104	3.20978628	0.4244426	7.56235649	3.96E-14	1.45E-13	Up	C9orf57
KRT5	60.308516	2.80133482	0.37048542	7.5612553	3.99E-14	1.47E-13	Up	KRT5
DHX30	3306.39946	0.61221379	0.08097626	7.56041086	4.02E-14	1.48E-13	Up	DHX30
CNKSR3	699.18957	-0.78773747	0.10421179	-7.55900551	4.06E-14	1.49E-13	Down	CNKSR3
FER1L6	242.579376	-2.38032862	0.31490315	-7.55892276	4.06E-14	1.49E-13	Down	FER1L6
FSTL3	834.709582	1.25143436	0.16557217	7.55824095	4.09E-14	1.50E-13	Up	FSTL3
THEMIS	44.6412123	-1.47053419	0.19459552	-7.55687595	4.13E-14	1.52E-13	Down	THEMIS
PC	705.091736	-0.98369576	0.13018394	-7.55619905	4.15E-14	1.52E-13	Down	PC
UGT1A1	27.3810003	-2.74964093	0.36409134	-7.55206352	4.28E-14	1.57E-13	Down	UGT1A1
MAP4K4	2939.81187	0.64821908	0.08584994	7.55060632	4.33E-14	1.59E-13	Up	MAP4K4
LDLRAD2	49.2972053	-0.98599031	0.13058517	-7.55055334	4.33E-14	1.59E-13	Down	LDLRAD2
MFSD1	1769.9306	-0.58954663	0.07808242	-7.55031208	4.34E-14	1.59E-13	Down	MFSD1
DDX39B	2528.65196	0.74945372	0.09927534	7.54924319	4.38E-14	1.60E-13	Up	DDX39B
AKR1C2	136.200512	-1.54337653	0.20446492	-7.54836825	4.41E-14	1.62E-13	Down	AKR1C2
DDX28	715.795012	0.66587363	0.08826258	7.54423486	4.55E-14	1.67E-13	Up	DDX28
ZNF280B	95.7895505	1.12515306	0.14921026	7.54072168	4.67E-14	1.71E-13	Up	ZNF280B
KRTAP3-2	1.83403265	3.50375996	0.46464739	7.54068572	4.68E-14	1.71E-13	Up	KRTAP3-2
SUPT3H	242.594806	0.63530398	0.08427202	7.53873003	4.75E-14	1.74E-13	Up	SUPT3H
RAB22A	2579.201	0.62839445	0.08336206	7.53813515	4.77E-14	1.75E-13	Up	RAB22A
XYLT1	1076.83249	-0.82619867	0.10961077	-7.53756867	4.79E-14	1.75E-13	Down	XYLT1
KIAA1614	78.385128	-0.9705023	0.12877164	-7.53661541	4.82E-14	1.76E-13	Down	KIAA1614
RNF113A	694.557115	0.69655531	0.0924311	7.53594136	4.85E-14	1.77E-13	Up	RNF113A
NMUR2	19.9385873	3.61635689	0.47991836	7.5353585	4.87E-14	1.78E-13	Up	NMUR2
LASP1	13095.8154	-0.51607215	0.0685124	-7.53253632	4.98E-14	1.82E-13	Down	LASP1
XK	1189.21112	-0.88546708	0.11755789	-7.53217913	4.99E-14	1.82E-13	Down	XK
AMER1	515.382473	0.78221198	0.10385311	7.53190742	5.00E-14	1.83E-13	Up	AMER1
BMP8B	459.601616	-0.80022145	0.1062507	-7.53144658	5.02E-14	1.83E-13	Down	BMP8B
AR	79.206484	-1.76131068	0.23396723	-7.52802304	5.15E-14	1.88E-13	Down	AR
DNAJC10	4128.73539	0.64917864	0.08623595	7.52793527	5.15E-14	1.88E-13	Up	DNAJC10
MAP1B	568.913803	-1.37455327	0.18259742	-7.52778043	5.16E-14	1.89E-13	Down	MAP1B

SLC17A1	4.8226554	-2.22072506	0.29502069	-7.52735375	5.18E-14	1.89E-13	Down	SLC17A1
MYL6B	718.923301	0.77741555	0.10329359	7.52627073	5.22E-14	1.91E-13	Up	MYL6B
PNLIPRP2	133.562255	-2.31853666	0.30812639	-7.52462874	5.29E-14	1.93E-13	Down	PNLIPRP2
BNC2	214.213657	-1.64532091	0.21866034	-7.52455123	5.29E-14	1.93E-13	Down	BNC2
ZC3H15	3205.93599	0.51760949	0.06879875	7.52353076	5.33E-14	1.95E-13	Up	ZC3H15
NDC1	2119.48605	0.62226236	0.08271979	7.52253286	5.37E-14	1.96E-13	Up	NDC1
SNX33	1556.6398	-0.5325393	0.07079723	-7.52203601	5.39E-14	1.97E-13	Down	SNX33
GZMM	38.8298857	-1.46092737	0.1942954	-7.51910408	5.52E-14	2.01E-13	Down	GZMM
UQCRC2	9842.34751	-0.53919638	0.07171196	-7.5189189	5.52E-14	2.01E-13	Down	UQCRC2
LGALS9	4757.6744	-0.84839958	0.11283972	-7.51862547	5.54E-14	2.02E-13	Down	LGALS9
ST8SIA3	4.44708601	-3.1495032	0.41900849	-7.51656182	5.62E-14	2.05E-13	Down	ST8SIA3
KALRN	1521.18694	-0.68539883	0.09120356	-7.51504449	5.69E-14	2.07E-13	Down	KALRN
TMEM123	12141.7905	0.6507205	0.08659711	7.51434446	5.72E-14	2.08E-13	Up	TMEM123
ZNF568	62.973775	-1.27876271	0.17017895	-7.51422394	5.72E-14	2.09E-13	Down	ZNF568
ZNF624	92.1704564	-0.76091277	0.10128391	-7.51267152	5.79E-14	2.11E-13	Down	ZNF624
XCL2	8.14071082	-1.71549628	0.22835011	-7.51257054	5.80E-14	2.11E-13	Down	XCL2
ZBED6CL	504.426512	0.68686305	0.09142823	7.51259294	5.80E-14	2.11E-13	Up	ZBED6CL
EIF3E	14471.2946	0.73470603	0.09782564	7.51036249	5.90E-14	2.15E-13	Up	EIF3E
PLEKHG6	2374.95058	-0.83742956	0.11151534	-7.50954584	5.93E-14	2.16E-13	Down	PLEKHG6
PRPF40B	210.414297	0.71637039	0.0953944	7.50956437	5.93E-14	2.16E-13	Up	PRPF40B
GPR35	3423.46277	0.87213691	0.11614325	7.50914825	5.95E-14	2.16E-13	Up	GPR35
PLP2	8783.76783	0.78746857	0.10487535	7.5086147	5.98E-14	2.17E-13	Up	PLP2
GFI1B	15.9633752	-2.04023481	0.27172629	-7.50841885	5.98E-14	2.18E-13	Down	GFI1B
KIR2DL4	15.1625176	-2.00125549	0.26654651	-7.50809118	6.00E-14	2.18E-13	Down	KIR2DL4
NEK7	1694.96272	-0.77475881	0.10320216	-7.5071954	6.04E-14	2.20E-13	Down	NEK7
CYP26B1	99.2082048	-1.33470651	0.17781858	-7.50600148	6.10E-14	2.21E-13	Down	CYP26B1
OIP5	204.39932	0.8270525	0.11018538	7.50600935	6.10E-14	2.21E-13	Up	OIP5
NOL11	1881.54834	0.52238658	0.06960372	7.5051533	6.14E-14	2.23E-13	Up	NOL11
AHRR	65.796712	-1.18791423	0.15830171	-7.50411475	6.18E-14	2.25E-13	Down	AHRR
GPR88	6.12200645	-2.10653202	0.28073952	-7.50351082	6.21E-14	2.26E-13	Down	GPR88
RHEB	2465.58432	0.5674166	0.07563654	7.50188452	6.29E-14	2.28E-13	Up	RHEB
TTLL7	93.3244471	-1.57139333	0.20952325	-7.49985201	6.39E-14	2.32E-13	Down	TTLL7
WDR36	1406.84955	0.66882936	0.08918632	7.49923701	6.42E-14	2.33E-13	Up	WDR36
MRPL50	1099.02455	0.61666309	0.0822353	7.49876403	6.44E-14	2.34E-13	Up	MRPL50
ZNF707	437.457053	0.69778989	0.09306696	7.49771857	6.49E-14	2.35E-13	Up	ZNF707
PSMD12	2139.95364	0.50254926	0.06703027	7.49734773	6.51E-14	2.36E-13	Up	PSMD12
HEPACAM2	874.285104	-2.38936414	0.31870492	-7.49710458	6.52E-14	2.36E-13	Down	HEPACAM2
DCAF12L2	2.00609649	-2.59783992	0.34652164	-7.49690539	6.53E-14	2.37E-13	Down	DCAF12L2
JMJD4	533.916789	0.6162934	0.08224659	7.49323919	6.72E-14	2.43E-13	Up	JMJD4
ENO3	150.555057	0.90592615	0.12090933	7.49260747	6.75E-14	2.45E-13	Up	ENO3
THOC2	2639.96188	0.72048163	0.09616808	7.49189982	6.79E-14	2.46E-13	Up	THOC2
ADAP2	229.406335	-0.86934998	0.1160481	-7.49129008	6.82E-14	2.47E-13	Down	ADAP2
TINF2	1262.83749	-0.51690899	0.06901154	-7.49018151	6.88E-14	2.49E-13	Down	TINF2
DIRAS2	6.49549716	-2.21877922	0.29623506	-7.48992784	6.89E-14	2.49E-13	Down	DIRAS2
GSTCD	491.393922	0.62050298	0.08285888	7.48867177	6.96E-14	2.52E-13	Up	GSTCD
ADAM28	281.609177	-1.3442821	0.17950991	-7.48862314	6.96E-14	2.52E-13	Down	ADAM28
LMNB1	3303.76175	0.59958474	0.08010387	7.4850905	7.15E-14	2.59E-13	Up	LMNB1
IMPA2	2143.92723	-0.8246676	0.11020862	-7.48278659	7.28E-14	2.63E-13	Down	IMPA2
AKAP7	391.837315	-1.22850492	0.16419546	-7.4819663	7.32E-14	2.65E-13	Down	AKAP7
CYP4F12	872.122009	-1.14250203	0.1527205	-7.48100002	7.38E-14	2.67E-13	Down	CYP4F12
IP6K3	18.2778187	-1.52647563	0.20412887	-7.4779996	7.55E-14	2.73E-13	Down	IP6K3
SMIM2	2.79804245	-1.55143595	0.20746761	-7.47796702	7.55E-14	2.73E-13	Down	SMIM2
TPSG1	269.453761	-2.05336289	0.27462897	-7.47686182	7.61E-14	2.75E-13	Down	TPSG1
A1CF	923.145807	-1.18982176	0.15915963	-7.47565033	7.68E-14	2.77E-13	Down	A1CF
ZNF747	325.696535	-0.52576947	0.0703483	-7.47380456	7.79E-14	2.81E-13	Down	ZNF747
C4orf33	496.81135	-0.83086536	0.11120068	-7.47176529	7.91E-14	2.86E-13	Down	C4orf33
ENPP1	363.795708	-1.17439494	0.15720376	-7.47052706	7.99E-14	2.88E-13	Down	ENPP1
SCX	94.3672136	1.32906113	0.17801606	7.46596187	8.27E-14	2.98E-13	Up	SCX

PANX2	23.0992197	1.83678483	0.24604074	7.46536867	8.31E-14	3.00E-13	Up	PANX2
PRPF40A	4257.81517	0.51474985	0.06895276	7.46525359	8.31E-14	3.00E-13	Up	PRPF40A
PEX5	2113.82549	0.50122219	0.06714368	7.46492024	8.34E-14	3.00E-13	Up	PEX5
JPH3	14.1214466	-1.81799979	0.24354379	-7.4647758	8.34E-14	3.01E-13	Down	JPH3
GPR89B	91.718986	0.69678384	0.09335711	7.46364012	8.42E-14	3.03E-13	Up	GPR89B
MXD3	387.151682	0.82897668	0.11107705	7.46307781	8.45E-14	3.04E-13	Up	MXD3
SLC41A3	1847.12624	0.5783844	0.077502	7.46283216	8.47E-14	3.05E-13	Up	SLC41A3
IRF3	2288.44681	0.5951956	0.07977703	7.46073879	8.60E-14	3.10E-13	Up	IRF3
NPAS2	1960.00022	0.7382576	0.09895341	7.46065881	8.61E-14	3.10E-13	Up	NPAS2
GNGT2	31.7263635	-0.98772166	0.13239826	-7.4602312	8.64E-14	3.11E-13	Down	GNGT2
ITGB8	822.990572	1.15278277	0.15463202	7.45500671	8.99E-14	3.23E-13	Up	ITGB8
LRP10	11757.2042	-0.7553633	0.10133894	-7.45383072	9.07E-14	3.26E-13	Down	LRP10
NTRK3	32.3642225	-2.06995824	0.27770585	-7.45377969	9.07E-14	3.26E-13	Down	NTRK3
FBXL3	1703.03519	-0.65515878	0.08792052	-7.45171656	9.21E-14	3.31E-13	Down	FBXL3
HSPBP1	2627.8777	0.88562903	0.11886772	7.45054297	9.30E-14	3.34E-13	Up	HSPBP1
TMEM145	26.6409008	1.67025278	0.22418972	7.45017547	9.32E-14	3.35E-13	Up	TMEM145
ITGB7	191.623711	-1.30562784	0.17526064	-7.44963517	9.36E-14	3.36E-13	Down	ITGB7
KCNE3	2110.26074	0.76276013	0.10242496	7.44701386	9.55E-14	3.43E-13	Up	KCNE3
TASP1	465.173236	0.66358919	0.08911538	7.4464047	9.59E-14	3.44E-13	Up	TASP1
RPS4X	71185.1225	0.74537798	0.10010047	7.44629887	9.60E-14	3.45E-13	Up	RPS4X
ZNF250	372.315813	0.58169345	0.07814871	7.44341675	9.81E-14	3.52E-13	Up	ZNF250
SLC35A1	567.058667	-0.74594632	0.10021623	-7.44336817	9.82E-14	3.52E-13	Down	SLC35A1
TPM4	19215.7611	0.50497143	0.06785821	7.44156675	9.95E-14	3.57E-13	Up	TPM4
CD320	3424.31496	0.96926513	0.13031353	7.43794693	1.02E-13	3.67E-13	Up	CD320
TEPP	1.47065628	-1.66614403	0.22400989	-7.43781451	1.02E-13	3.67E-13	Down	TEPP
ATP8B2	530.132127	-1.06627293	0.14336774	-7.43732836	1.03E-13	3.68E-13	Down	ATP8B2
MRPS12	2161.79979	0.90831857	0.12216447	7.43521076	1.04E-13	3.74E-13	Up	MRPS12
FAM50A	2590.84475	0.79548658	0.10699135	7.43505505	1.05E-13	3.75E-13	Up	FAM50A
OXLD1	813.984456	0.85670742	0.11524794	7.43360306	1.06E-13	3.79E-13	Up	OXLD1
TNFAIP8L2	88.6125434	-1.03249073	0.13889801	-7.43344486	1.06E-13	3.79E-13	Down	TNFAIP8L2
ARMC6	1710.89397	0.77570039	0.10435555	7.43324535	1.06E-13	3.80E-13	Up	ARMC6
SYT12	30.9097478	1.7579273	0.23651054	7.43276527	1.06E-13	3.81E-13	Up	SYT12
ABHD14A-AC	15.4456984	1.07868246	0.14513697	7.43216854	1.07E-13	3.83E-13	Up	ABHD14A-ACY1
TAL1	47.6578867	-1.08355709	0.14582827	-7.43036392	1.08E-13	3.88E-13	Down	TAL1
SERPINA9	9.03305398	-3.92943778	0.52884813	-7.4301818	1.08E-13	3.88E-13	Down	SERPINA9
NDUFC1	1367.00762	-0.55820323	0.07512888	-7.42994258	1.09E-13	3.89E-13	Down	NDUFC1
ELOVL5	2080.49192	0.90741791	0.12213817	7.42943775	1.09E-13	3.90E-13	Up	ELOVL5
C1orf54	160.9487	-0.72794631	0.09799987	-7.42803331	1.10E-13	3.94E-13	Down	C1orf54
TMEM182	208.16784	0.67389341	0.09073604	7.42696481	1.11E-13	3.97E-13	Up	TMEM182
EFNA4	923.257244	0.6760076	0.09109085	7.42124554	1.16E-13	4.15E-13	Up	EFNA4
CALU	5983.76037	0.6439324	0.08679223	7.41924058	1.18E-13	4.21E-13	Up	CALU
HCN4	2.92049789	-1.88143218	0.25359554	-7.41902721	1.18E-13	4.22E-13	Down	HCN4
PRMT7	1344.19998	0.58864805	0.07935144	7.41824026	1.19E-13	4.24E-13	Up	PRMT7
TPSAB1	273.404944	-1.58591842	0.21382899	-7.4167604	1.20E-13	4.29E-13	Down	TPSAB1
GLMN	271.412462	0.70204886	0.09466491	7.41614666	1.21E-13	4.31E-13	Up	GLMN
ALAS1	2728.6445	-0.56197987	0.07578373	-7.41557381	1.21E-13	4.32E-13	Down	ALAS1
MTRF1	423.697328	0.7033471	0.09487573	7.41335133	1.23E-13	4.40E-13	Up	MTRF1
GSTA1	63.2043802	-2.23478186	0.30147599	-7.41280217	1.24E-13	4.41E-13	Down	GSTA1
IL17B	8.91533275	-1.22560141	0.16533837	-7.41268589	1.24E-13	4.42E-13	Down	IL17B
RIPPLY1	5.65244514	2.10278875	0.28367388	7.41269773	1.24E-13	4.42E-13	Up	RIPPLY1
MDM1	273.641392	-0.65143338	0.08788489	-7.4123481	1.24E-13	4.43E-13	Down	MDM1
SEC61A2	197.196407	0.67003031	0.09039575	7.41218825	1.24E-13	4.43E-13	Up	SEC61A2
C17orf99	9.62481221	2.08169075	0.28085165	7.41206512	1.24E-13	4.43E-13	Up	C17orf99
TPO	25.1179856	-1.79543527	0.24228299	-7.41048835	1.26E-13	4.49E-13	Down	TPO
FAM181B	9.25810898	-2.14648139	0.28972592	-7.40866188	1.28E-13	4.55E-13	Down	FAM181B
TMEM234	423.333978	0.60360321	0.08153152	7.40331108	1.33E-13	4.73E-13	Up	TMEM234
MKNK2	6525.59971	-0.63210913	0.08540614	-7.40121384	1.35E-13	4.81E-13	Down	MKNK2
NDC80	502.730589	0.77453627	0.1046553	7.4008318	1.35E-13	4.82E-13	Up	NDC80

EIF3D	9855.64063	0.53672964	0.0725608	7.39696435	1.39E-13	4.96E-13	Up	EIF3D
CDC42BPG	1279.29781	-0.76529918	0.10347727	-7.39581925	1.41E-13	5.01E-13	Down	CDC42BPG
AGTPBP1	608.911959	0.59585304	0.08058066	7.39449181	1.42E-13	5.05E-13	Up	AGTPBP1
WIPF2	2584.84866	-0.61899503	0.08371193	-7.39434699	1.42E-13	5.06E-13	Down	WIPF2
CDC42BPA	1900.05875	-0.74828008	0.10121918	-7.39267082	1.44E-13	5.12E-13	Down	CDC42BPA
C2orf72	937.920424	-1.12265413	0.15190338	-7.39058051	1.46E-13	5.20E-13	Down	C2orf72
MRPS2	2783.29859	0.82529552	0.11171595	7.38744545	1.50E-13	5.32E-13	Up	MRPS2
KIAA1549L	155.103741	2.06336044	0.27937041	7.38575156	1.52E-13	5.39E-13	Up	KIAA1549L
DEFB1	135.848973	-1.89088948	0.25606712	-7.38435081	1.53E-13	5.45E-13	Down	DEFB1
ACVR1C	229.026584	-1.26436399	0.17123982	-7.38358631	1.54E-13	5.48E-13	Down	ACVR1C
ABHD1	19.784339	1.30643256	0.17694782	7.38315147	1.55E-13	5.49E-13	Up	ABHD1
KRT32	3.31165512	4.44344877	0.60207701	7.38020004	1.58E-13	5.61E-13	Up	KRT32
ABCA10	43.0289738	-1.46827223	0.19896715	-7.37947048	1.59E-13	5.64E-13	Down	ABCA10
KDELR3	1324.30784	0.88316984	0.11972374	7.37673123	1.62E-13	5.76E-13	Up	KDELR3
THEM4	503.92835	0.61680721	0.08361612	7.37665471	1.62E-13	5.76E-13	Up	THEM4
RPA3	1014.4128	0.68181237	0.09244546	7.37529353	1.64E-13	5.82E-13	Up	RPA3
ZDHHC1	580.903662	0.77288514	0.10479353	7.37531374	1.64E-13	5.82E-13	Up	ZDHHC1
TJAP1	1008.53628	0.55399401	0.07512826	7.37397635	1.66E-13	5.87E-13	Up	TJAP1
ZDHHC22	3.1046769	-3.16347853	0.42900625	-7.37396839	1.66E-13	5.87E-13	Down	ZDHHC22
CALML6	7.9752949	1.75995045	0.23868363	7.37357009	1.66E-13	5.89E-13	Up	CALML6
DOCK2	446.171235	-1.2564629	0.17042623	-7.37247352	1.67E-13	5.94E-13	Down	DOCK2
PSKH1	1446.43217	-0.61122127	0.0829067	-7.37239905	1.68E-13	5.94E-13	Down	PSKH1
NGFR	119.921455	-1.80474284	0.2448454	-7.37094867	1.69E-13	6.00E-13	Down	NGFR
ZNF728	1.30356591	-2.6610257	0.36103212	-7.37060658	1.70E-13	6.02E-13	Down	ZNF728
GPLD1	24.446606	-1.06454837	0.14443857	-7.37025019	1.70E-13	6.03E-13	Down	GPLD1
PDLIM7	1962.06983	-1.01873102	0.13822383	-7.37015496	1.70E-13	6.04E-13	Down	PDLIM7
ACSBG1	14.6909909	-1.01712736	0.13803092	-7.36883695	1.72E-13	6.10E-13	Down	ACSBG1
XPO1	5887.40092	0.54403441	0.07383702	7.368044	1.73E-13	6.13E-13	Up	XPO1
PRSS53	35.3259295	1.07092149	0.1453646	7.36714113	1.74E-13	6.17E-13	Up	PRSS53
CDK20	218.323944	-0.85702325	0.11633875	-7.36661908	1.75E-13	6.19E-13	Down	CDK20
TBL3	2289.61898	0.82720132	0.11233212	7.36388973	1.79E-13	6.32E-13	Up	TBL3
TTL	1227.91452	0.51190866	0.06958316	7.35678964	1.88E-13	6.66E-13	Up	TTL
BFSP2	5.09197345	-1.71446567	0.23312206	-7.35436899	1.92E-13	6.78E-13	Down	BFSP2
UNC119	938.377458	0.59761768	0.08127557	7.35298069	1.94E-13	6.85E-13	Up	UNC119
BMP2K	386.655706	-0.68762566	0.09353078	-7.35186502	1.95E-13	6.91E-13	Down	BMP2K
CLSTN1	10366.9036	-0.58728689	0.0798908	-7.35111994	1.97E-13	6.95E-13	Down	CLSTN1
MINK1	5447.8492	-0.6179371	0.08407576	-7.34976494	1.99E-13	7.02E-13	Down	MINK1
FZD6	962.622168	0.66549243	0.09055777	7.34881657	2.00E-13	7.06E-13	Up	FZD6
THBS4	458.912816	-2.33444645	0.31767509	-7.34853487	2.00E-13	7.08E-13	Down	THBS4
HRK	8.15788614	-2.5212477	0.34320364	-7.34621492	2.04E-13	7.20E-13	Down	HRK
FAM216A	164.993294	0.86152836	0.11728453	7.34562689	2.05E-13	7.23E-13	Up	FAM216A
CHID1	2970.14417	0.60216283	0.0819866	7.34464941	2.06E-13	7.28E-13	Up	CHID1
FANCF	991.402357	0.54261116	0.07390703	7.34180701	2.11E-13	7.44E-13	Up	FANCF
ZNF205	594.081628	0.73013684	0.09945835	7.34113131	2.12E-13	7.47E-13	Up	ZNF205
FITM2	1334.16026	0.89014905	0.12127434	7.33996191	2.14E-13	7.53E-13	Up	FITM2
GALNT7	3041.7346	-0.80296469	0.10939679	-7.33992951	2.14E-13	7.53E-13	Down	GALNT7
HBQ1	6.06941001	3.21358114	0.43786979	7.339125	2.15E-13	7.58E-13	Up	HBQ1
TCF20	2768.86131	0.54968758	0.07489853	7.33909689	2.15E-13	7.58E-13	Up	TCF20
FAM126B	699.017941	-0.8846607	0.12054469	-7.33886096	2.15E-13	7.59E-13	Down	FAM126B
ZNF738	190.934607	0.89312947	0.12171652	7.3377833	2.17E-13	7.65E-13	Up	ZNF738
P4HA1	2157.89112	0.82179163	0.11199754	7.33758616	2.17E-13	7.66E-13	Up	P4HA1
C19orf81	5.1917217	3.0490202	0.41565356	7.33548439	2.21E-13	7.78E-13	Up	C19orf81
MBP	1528.61714	-0.61592452	0.08398893	-7.33340151	2.24E-13	7.90E-13	Down	MBP
KCTD19	8.57781926	1.80597505	0.24629417	7.33259366	2.26E-13	7.94E-13	Up	KCTD19
TMEM213	3.75633035	2.93302147	0.40001896	7.33220621	2.26E-13	7.97E-13	Up	TMEM213
PGLYRP4	7.0827251	2.548234	0.34761676	7.33058435	2.29E-13	8.06E-13	Up	PGLYRP4
FAM83D	1267.97284	0.74593906	0.10178147	7.32882998	2.32E-13	8.17E-13	Up	FAM83D
HCAR2	75.8337788	2.22394266	0.30345843	7.32865681	2.32E-13	8.17E-13	Up	HCAR2

MYT1L	3.00285334	-2.89477444	0.39500403	-7.32846812	2.33E-13	8.18E-13	Down	MYT1L
CXCL10	872.817433	1.78820236	0.24409349	7.3258913	2.37E-13	8.34E-13	Up	CXCL10
MAP3K3	740.300994	-0.59269356	0.08091242	-7.32512414	2.39E-13	8.39E-13	Down	MAP3K3
PIK3CD	351.20509	-1.03627328	0.14147575	-7.32474133	2.39E-13	8.41E-13	Down	PIK3CD
FCGBP	33929.8244	-2.28359487	0.3117748	-7.32450123	2.40E-13	8.42E-13	Down	FCGBP
NCOA7	3873.11349	0.85592844	0.11685952	7.32442227	2.40E-13	8.43E-13	Up	NCOA7
DOCK10	317.358127	-1.19057165	0.16255604	-7.32406903	2.41E-13	8.45E-13	Down	DOCK10
MPG	1349.01177	0.8423194	0.11500999	7.32388033	2.41E-13	8.46E-13	Up	MPG
KCNQ1	5272.63398	1.03000792	0.14068995	7.3211194	2.46E-13	8.63E-13	Up	KCNQ1
CDH6	166.576928	1.00288316	0.13704227	7.31805708	2.52E-13	8.83E-13	Up	CDH6
CABLES2	705.983367	0.77800604	0.10632085	7.31753056	2.53E-13	8.86E-13	Up	CABLES2
GNAZ	126.923255	-1.14578358	0.15659974	-7.31663788	2.54E-13	8.92E-13	Down	GNAZ
TATDN1	768.407757	0.70900772	0.09690407	7.31659394	2.54E-13	8.92E-13	Up	TATDN1
SORBS3	2673.22246	-0.76155199	0.10408913	-7.31634535	2.55E-13	8.93E-13	Down	SORBS3
FCN1	118.825463	-1.7235198	0.23558463	-7.31592618	2.56E-13	8.96E-13	Down	FCN1
LRRC45	1060.6253	0.77403348	0.10580459	7.31568872	2.56E-13	8.97E-13	Up	LRRC45
NPY1R	68.998028	-2.02695203	0.27708007	-7.3154017	2.57E-13	8.99E-13	Down	NPY1R
NAPEPLD	1013.39446	-0.60861453	0.08324485	-7.31113769	2.65E-13	9.28E-13	Down	NAPEPLD
HPSE	377.776415	-0.99675235	0.13633709	-7.31094046	2.65E-13	9.29E-13	Down	HPSE
MAP3K10	506.288705	0.76529948	0.10468204	7.31070485	2.66E-13	9.31E-13	Up	MAP3K10
ARHGAP28	68.8008814	-1.19446671	0.16348751	-7.30616504	2.75E-13	9.62E-13	Down	ARHGAP28
LYSMD3	723.241973	-0.72236406	0.09892374	-7.30223162	2.83E-13	9.91E-13	Down	LYSMD3
DLST	4057.43311	-0.51575374	0.07064188	-7.30096247	2.86E-13	1.00E-12	Down	DLST
CLEC4F	6.37486715	-1.78301418	0.24431024	-7.2981557	2.92E-13	1.02E-12	Down	CLEC4F
CA10	5.27433622	-2.27072748	0.31126069	-7.29525951	2.98E-13	1.04E-12	Down	CA10
RNF225	1.49819443	3.3474763	0.45893232	7.29405228	3.01E-13	1.05E-12	Up	RNF225
PPARA	1917.60018	-0.58453748	0.0801536	-7.29271694	3.04E-13	1.06E-12	Down	PPARA
HECW1	41.6454472	1.47292863	0.20200893	7.29140362	3.07E-13	1.07E-12	Up	HECW1
LMLN	316.597595	0.66036397	0.09061928	7.28723516	3.16E-13	1.11E-12	Up	LMLN
ZEB2	806.003201	-1.32539643	0.18190458	-7.28621818	3.19E-13	1.11E-12	Down	ZEB2
HSPA6	159.571897	1.48289255	0.20351957	7.28624076	3.19E-13	1.11E-12	Up	HSPA6
PLOD2	2585.1939	-0.97162567	0.13343386	-7.28170265	3.30E-13	1.15E-12	Down	PLOD2
SPIN3	132.286484	1.16154852	0.15953165	7.2809913	3.31E-13	1.16E-12	Up	SPIN3
FBXO44	422.624555	0.97999465	0.13460617	7.28045844	3.33E-13	1.16E-12	Up	FBXO44
MOK	250.920085	1.07025994	0.14704568	7.2784183	3.38E-13	1.18E-12	Up	MOK
EIF3I	8632.99566	0.57930116	0.07962093	7.27573963	3.45E-13	1.20E-12	Up	EIF3I
UNC5A	31.6696908	2.10556845	0.28940783	7.2754371	3.45E-13	1.20E-12	Up	UNC5A
KIAA1958	465.445486	0.64345426	0.0884869	7.27174627	3.55E-13	1.24E-12	Up	KIAA1958
E4F1	1196.18162	0.78321115	0.10771487	7.27115148	3.56E-13	1.24E-12	Up	E4F1
ACADVL	9204.60679	-0.67096773	0.09228163	-7.27086982	3.57E-13	1.25E-12	Down	ACADVL
ALK	7.2787827	-2.48114994	0.34127135	-7.27031425	3.59E-13	1.25E-12	Down	ALK
LIPC	30.1815132	-2.07470385	0.28539121	-7.26968381	3.60E-13	1.26E-12	Down	LIPC
SP9	5.59835288	4.99860761	0.68765994	7.26901093	3.62E-13	1.26E-12	Up	SP9
VCAN	3776.15198	1.38107135	0.19003867	7.26731749	3.67E-13	1.28E-12	Up	VCAN
NUDT14	1171.07426	0.93594578	0.12882423	7.26529301	3.72E-13	1.30E-12	Up	NUDT14
FBXL13	26.2198368	1.41363358	0.19463254	7.26308961	3.78E-13	1.32E-12	Up	FBXL13
EFHD1	91.9913452	-1.12267644	0.15457417	-7.26302738	3.79E-13	1.32E-12	Down	EFHD1
FUT8	1550.42418	0.84976157	0.11701312	7.26210497	3.81E-13	1.33E-12	Up	FUT8
DNM2	10346.3769	-0.56805403	0.07822556	-7.26174439	3.82E-13	1.33E-12	Down	DNM2
TAGLN2	24008.9575	0.56450561	0.07774131	7.26133358	3.83E-13	1.33E-12	Up	TAGLN2
IER3	6038.75827	0.99784076	0.13742926	7.26075899	3.85E-13	1.34E-12	Up	IER3
PIGL	341.326756	0.60640682	0.08354324	7.25859791	3.91E-13	1.36E-12	Up	PIGL
GOLPH3L	2031.70551	-0.54223434	0.07470598	-7.25824519	3.92E-13	1.36E-12	Down	GOLPH3L
CITED4	346.088849	1.39145443	0.19172405	7.25758936	3.94E-13	1.37E-12	Up	CITED4
SMG6	619.883571	-0.63446869	0.08744366	-7.25574227	3.99E-13	1.39E-12	Down	SMG6
TSPO2	16.7669057	1.48276381	0.20436434	7.25549198	4.00E-13	1.39E-12	Up	TSPO2
ALDH3A2	2721.42363	-0.65809459	0.09071704	-7.25436539	4.04E-13	1.40E-12	Down	ALDH3A2
SEPHS2	4698.00159	-0.65099982	0.08974792	-7.25364821	4.06E-13	1.41E-12	Down	SEPHS2

TMEM184B	3186.32914	0.58141242	0.08017544	7.25175204	4.11E-13	1.43E-12	Up	TMEM184B
APMAP	5286.65553	0.70707043	0.09750694	7.2514886	4.12E-13	1.43E-12	Up	APMAP
C17orf75	622.448613	0.54120273	0.07468079	7.24687967	4.26E-13	1.48E-12	Up	C17orf75
BACH2	62.2375778	-1.52732152	0.21078421	-7.24590097	4.30E-13	1.49E-12	Down	BACH2
FCHO2	1233.09509	-0.70484156	0.09731191	-7.24311706	4.38E-13	1.52E-12	Down	FCHO2
RNF187	4054.6364	0.5930558	0.08188298	7.24272372	4.40E-13	1.53E-12	Up	RNF187
TYSND1	1384.65723	0.72224646	0.09977087	7.23905109	4.52E-13	1.57E-12	Up	TYSND1
HTRA4	13.4844766	1.62160735	0.22401746	7.23875435	4.53E-13	1.57E-12	Up	HTRA4
GJB3	1090.21109	1.20112318	0.16598076	7.23652049	4.60E-13	1.60E-12	Up	GJB3
ZNF133	481.025775	0.68710665	0.09495447	7.23616945	4.62E-13	1.60E-12	Up	ZNF133
CCDC18	183.031373	0.91070936	0.12586051	7.23586238	4.63E-13	1.60E-12	Up	CCDC18
AMN	2155.73318	-1.36838804	0.18916142	-7.23397002	4.69E-13	1.62E-12	Down	AMN
HOXC12	7.34280671	5.16097965	0.71369771	7.23132437	4.78E-13	1.66E-12	Up	HOXC12
CD200R1	39.6829036	-1.42478804	0.19711948	-7.22804293	4.90E-13	1.70E-12	Down	CD200R1
HPCAL4	4.95221808	-1.84063861	0.25469742	-7.22676577	4.95E-13	1.71E-12	Down	HPCAL4
TUBA4A	5251.28048	0.79372529	0.10985121	7.22545787	4.99E-13	1.73E-12	Up	TUBA4A
YRDC	861.079931	0.51554438	0.07136187	7.22436756	5.03E-13	1.74E-12	Up	YRDC
DVL1	3102.0667	0.81231443	0.11244317	7.22422215	5.04E-13	1.74E-12	Up	DVL1
CUL7	1540.11221	0.61035796	0.08450012	7.22316125	5.08E-13	1.76E-12	Up	CUL7
ZFYVE9	623.319618	-0.52436088	0.0726153	-7.22107964	5.16E-13	1.78E-12	Down	ZFYVE9
POFUT1	6146.27896	0.83569406	0.11573498	7.220756	5.17E-13	1.79E-12	Up	POFUT1
MICALL1	1242.79936	-0.54221149	0.07509894	-7.21996178	5.20E-13	1.80E-12	Down	MICALL1
RALYL	2.04645859	-3.18227773	0.44080587	-7.21922728	5.23E-13	1.81E-12	Down	RALYL
RPL36	24563.4883	0.86069808	0.11923792	7.21832521	5.26E-13	1.82E-12	Up	RPL36
SAC3D1	628.17221	1.02934355	0.14264474	7.21613381	5.35E-13	1.85E-12	Up	SAC3D1
ZFAND1	1507.70414	0.68709344	0.09522186	7.21571151	5.37E-13	1.85E-12	Up	ZFAND1
NUDT7	107.669361	-0.66543686	0.09223164	-7.21484341	5.40E-13	1.86E-12	Down	NUDT7
MMRN2	503.272177	-0.90184224	0.12500113	-7.21467249	5.41E-13	1.87E-12	Down	MMRN2
RABGEF1	138.15084	-0.57505752	0.07971007	-7.21436441	5.42E-13	1.87E-12	Down	RABGEF1
DSG2	13120.5497	-0.59637237	0.08268757	-7.21235782	5.50E-13	1.90E-12	Down	DSG2
SVOP	9.77668593	-2.12642705	0.29485991	-7.21165202	5.53E-13	1.91E-12	Down	SVOP
BDH1	2920.32912	-0.72719616	0.10085853	-7.21006128	5.59E-13	1.93E-12	Down	BDH1
POC1B	1752.23844	0.74351011	0.10317604	7.20622869	5.75E-13	1.98E-12	Up	POC1B
PUS7L	1030.91956	0.83956278	0.11653339	7.20448245	5.83E-13	2.01E-12	Up	PUS7L
ZNF443	215.229345	0.64238096	0.08916796	7.20416753	5.84E-13	2.01E-12	Up	ZNF443
SMC2	1499.59905	0.6485651	0.09002903	7.2039554	5.85E-13	2.01E-12	Up	SMC2
FRMD1	414.14321	-1.5706087	0.21802835	-7.20369029	5.86E-13	2.02E-12	Down	FRMD1
ASIP	5.00594048	1.55302162	0.21559673	7.20336359	5.87E-13	2.02E-12	Up	ASIP
SBSPON	511.394887	1.9122929	0.26548703	7.20296155	5.89E-13	2.03E-12	Up	SBSPON
AK3	3420.85862	-0.545126	0.07568476	-7.20258595	5.91E-13	2.03E-12	Down	AK3
RPL7	27898.5148	0.69825304	0.09696873	7.2008063	5.99E-13	2.06E-12	Up	RPL7
MED10	971.412593	0.52287235	0.07261908	7.20020612	6.01E-13	2.07E-12	Up	MED10
ANP32B	7384.75624	0.50815109	0.07061549	7.19602846	6.20E-13	2.13E-12	Up	ANP32B
PSMB7	4444.33869	0.54044839	0.07512271	7.19420787	6.28E-13	2.16E-12	Up	PSMB7
GEMIN4	1471.59712	0.63120415	0.08779035	7.1899036	6.48E-13	2.23E-12	Up	GEMIN4
SSTR1	261.069922	-1.3462	0.18730002	-7.18739895	6.60E-13	2.27E-12	Down	SSTR1
CLASRP	1233.15029	0.62331448	0.08672818	7.18698938	6.62E-13	2.28E-12	Up	CLASRP
MCMDC2	79.2518279	1.2861051	0.17898203	7.185666	6.69E-13	2.30E-12	Up	MCMDC2
DDX54	3517.56131	0.62510841	0.08699963	7.18518479	6.71E-13	2.30E-12	Up	DDX54
CDHR3	176.216826	-0.78329246	0.10902358	-7.18461481	6.74E-13	2.31E-12	Down	CDHR3
SBNO2	3551.87301	0.73027454	0.10164673	7.18443737	6.75E-13	2.32E-12	Up	SBNO2
IQSEC2	706.496352	-0.61857937	0.08610437	-7.1840651	6.77E-13	2.32E-12	Down	IQSEC2
ZNF841	361.728287	0.88037811	0.12255618	7.18346578	6.80E-13	2.33E-12	Up	ZNF841
CREBRF	603.824676	-0.85667333	0.11929997	-7.18083434	6.93E-13	2.38E-12	Down	CREBRF
WAS	246.166063	-1.04255629	0.14518783	-7.18074159	6.93E-13	2.38E-12	Down	WAS
CENPK	408.286983	0.88732321	0.1235938	7.17935027	7.00E-13	2.40E-12	Up	CENPK
GPR78	9.63401587	1.50492549	0.2096201	7.17929939	7.01E-13	2.40E-12	Up	GPR78
COPG2	1447.27091	0.52770699	0.07350904	7.17880435	7.03E-13	2.41E-12	Up	COPG2

METTL25	146.669992	-0.64098072	0.08931216	-7.17685814	7.13E-13	2.44E-12	Down	METTL25
NXPH3	124.036885	-1.68901561	0.23535732	-7.17638864	7.16E-13	2.45E-12	Down	NXPH3
SOWAHA	666.516283	-1.15293945	0.16065776	-7.17636938	7.16E-13	2.45E-12	Down	SOWAHA
POU3F4	1.43304423	-4.25894391	0.59387421	-7.17145788	7.42E-13	2.54E-12	Down	POU3F4
SERPINA10	78.0708149	2.44478329	0.34093067	7.17091032	7.45E-13	2.55E-12	Up	SERPINA10
MRGPRE	1.41327948	-2.3791064	0.33178812	-7.17055938	7.47E-13	2.55E-12	Down	MRGPRE
TBX4	18.4570111	2.54494026	0.35492163	7.17042869	7.48E-13	2.56E-12	Up	TBX4
WWC1	1502.72567	0.52095656	0.07266106	7.16968048	7.52E-13	2.57E-12	Up	WWC1
PRPH2	19.3464878	-1.14894697	0.16028606	-7.16810308	7.60E-13	2.60E-12	Down	PRPH2
FAM83A	70.2043788	2.03458496	0.28386313	7.16748579	7.64E-13	2.61E-12	Up	FAM83A
SDHB	3253.57972	-0.56927392	0.07942987	-7.16700057	7.67E-13	2.62E-12	Down	SDHB
RAB44	8.99969389	-1.38116174	0.19273874	-7.16597877	7.72E-13	2.64E-12	Down	RAB44
ACSL4	2026.40844	0.83869854	0.11706352	7.16447373	7.81E-13	2.67E-12	Up	ACSL4
ZMYND8	3256.354	0.69539129	0.09706474	7.16420113	7.82E-13	2.67E-12	Up	ZMYND8
HCN1	40.0075438	2.36939302	0.33080269	7.16255672	7.92E-13	2.70E-12	Up	HCN1
FRAT1	209.469081	-0.61333083	0.08563247	-7.16236261	7.93E-13	2.71E-12	Down	FRAT1
PLCXD3	15.8176557	-2.71327539	0.37895668	-7.1598563	8.08E-13	2.76E-12	Down	PLCXD3
CDH23	67.2635964	-1.23167667	0.17203232	-7.15956538	8.09E-13	2.76E-12	Down	CDH23
C11orf1	629.188264	0.67227918	0.09390569	7.15908897	8.12E-13	2.77E-12	Up	C11orf1
SPATA4	0.84654424	-2.58458154	0.36103667	-7.15877836	8.14E-13	2.78E-12	Down	SPATA4
SYNRG	1331.2297	-0.5089438	0.07109677	-7.15846599	8.16E-13	2.78E-12	Down	SYNRG
MYLPF	14.3302517	1.42956615	0.19975693	7.15652825	8.27E-13	2.82E-12	Up	MYLPF
NCAN	2.14069418	-2.02651389	0.2833223	-7.15268057	8.51E-13	2.90E-12	Down	NCAN
BST1	174.016737	-1.15766456	0.16185767	-7.15236162	8.53E-13	2.90E-12	Down	BST1
IGLL5	1751.95177	-1.97287581	0.27587033	-7.15146071	8.59E-13	2.92E-12	Down	IGLL5
KLC2	1058.24085	0.65785303	0.09200043	7.15054301	8.64E-13	2.94E-12	Up	KLC2
RAB12	569.654374	-0.64917573	0.09081955	-7.14797368	8.81E-13	3.00E-12	Down	RAB12
ADAMTS7	301.188303	1.02454766	0.14335185	7.14708371	8.86E-13	3.02E-12	Up	ADAMTS7
CTSD	21729.7584	-0.74678133	0.10449092	-7.14685411	8.88E-13	3.02E-12	Down	CTSD
CHRNA6	3.07939984	2.19507234	0.30718038	7.14587421	8.94E-13	3.04E-12	Up	CHRNA6
SEMA3D	57.0327764	-2.15683905	0.30184077	-7.1456188	8.96E-13	3.05E-12	Down	SEMA3D
CD2AP	3723.2272	-0.76099359	0.10652022	-7.14412357	9.06E-13	3.08E-12	Down	CD2AP
TMEM88	58.2111057	-0.88360404	0.12369337	-7.1435036	9.10E-13	3.09E-12	Down	TMEM88
NEFL	53.4565714	-2.60321529	0.36449821	-7.14191519	9.20E-13	3.13E-12	Down	NEFL
HOXD12	6.48685978	-2.51895051	0.3528316	-7.13924287	9.38E-13	3.19E-12	Down	HOXD12
MUCL1	10.9458916	3.10474754	0.43492524	7.1385776	9.43E-13	3.20E-12	Up	MUCL1
CHST15	580.241958	-1.1109022	0.15563982	-7.13764778	9.49E-13	3.22E-12	Down	CHST15
HSBP1L1	471.579625	-0.67828044	0.09504174	-7.13665841	9.56E-13	3.25E-12	Down	HSBP1L1
FNDC9	4.53698315	-2.02934169	0.28436219	-7.13646817	9.58E-13	3.25E-12	Down	FNDC9
SPZ1	1.43250542	3.15105108	0.44162723	7.13509229	9.67E-13	3.28E-12	Up	SPZ1
PARD6A	280.948335	0.96917147	0.13583266	7.13504023	9.68E-13	3.28E-12	Up	PARD6A
CERK	1759.16337	-0.75978554	0.10650945	-7.13350328	9.78E-13	3.32E-12	Down	CERK
DUSP1	3971.43421	-1.13844655	0.15959432	-7.13337742	9.79E-13	3.32E-12	Down	DUSP1
CACNG7	1.91590943	-2.15601966	0.302294	-7.13219459	9.88E-13	3.35E-12	Down	CACNG7
VLDLR	273.244873	-1.19585421	0.16767598	-7.13193493	9.90E-13	3.36E-12	Down	VLDLR
TIMM9	836.119918	0.60463622	0.08481977	7.12848257	1.01E-12	3.44E-12	Up	TIMM9
EIF2B3	770.631706	0.52637532	0.07386282	7.1263905	1.03E-12	3.49E-12	Up	EIF2B3
PIBF1	727.24832	0.75501809	0.10600734	7.12231902	1.06E-12	3.60E-12	Up	PIBF1
GMFG	320.418265	-0.90772303	0.12745615	-7.12184569	1.06E-12	3.61E-12	Down	GMFG
COX7C	8034.36789	-0.56647786	0.07954801	-7.12120753	1.07E-12	3.62E-12	Down	COX7C
DENND1B	665.68796	-0.79977635	0.11231202	-7.12102196	1.07E-12	3.63E-12	Down	DENND1B
GRP	43.8675715	1.95332785	0.27432077	7.12059764	1.07E-12	3.64E-12	Up	GRP
TRIM10	116.394242	-0.82217821	0.11546897	-7.12033916	1.08E-12	3.64E-12	Down	TRIM10
TMBIM1	10734.1132	-0.61773254	0.08677911	-7.11844755	1.09E-12	3.69E-12	Down	TMBIM1
UGCG	1429.89738	-0.6254819	0.08787609	-7.11777135	1.10E-12	3.71E-12	Down	UGCG
CALHM3	6.9878834	2.81624063	0.39574163	7.11636177	1.11E-12	3.75E-12	Up	CALHM3
CSNK1G3	1281.99996	-0.53287223	0.07490628	-7.11385239	1.13E-12	3.82E-12	Down	CSNK1G3
GPR156	7.65381168	-1.46133058	0.20542917	-7.1135496	1.13E-12	3.82E-12	Down	GPR156

KLK5	5.27701735	4.3248058	0.60802405	7.11288609	1.14E-12	3.84E-12	Up	KLK5
POLE3	3108.68656	0.53306707	0.07494743	7.11254623	1.14E-12	3.85E-12	Up	POLE3
TRIM25	2819.10011	-0.5247152	0.07377787	-7.11209437	1.14E-12	3.86E-12	Down	TRIM25
UST	113.867228	-1.52987165	0.2151453	-7.11087646	1.15E-12	3.90E-12	Down	UST
CLCN7	2279.89958	0.70444736	0.09910253	7.10826824	1.18E-12	3.97E-12	Up	CLCN7
ZFPM2	74.5062015	-1.37996087	0.19425476	-7.10387147	1.21E-12	4.10E-12	Down	ZFPM2
ITGA6	14001.3084	0.6501557	0.09153207	7.10303729	1.22E-12	4.12E-12	Up	ITGA6
TBC1D7	663.51183	0.58124388	0.08187422	7.09922947	1.25E-12	4.23E-12	Up	TBC1D7
SUPT20H	1662.79214	0.636409	0.08969476	7.09527491	1.29E-12	4.36E-12	Up	SUPT20H
CHORDC1	990.689147	0.87814943	0.12376955	7.09503591	1.29E-12	4.36E-12	Up	CHORDC1
STK32C	417.725853	1.01300593	0.14280806	7.09347895	1.31E-12	4.41E-12	Up	STK32C
CCDC51	836.921414	0.54907774	0.07740787	7.09330681	1.31E-12	4.41E-12	Up	CCDC51
POLR3D	931.956008	0.53778048	0.07582015	7.09284422	1.31E-12	4.43E-12	Up	POLR3D
RHBDD3	720.415445	0.68675774	0.09682804	7.09254985	1.32E-12	4.44E-12	Up	RHBDD3
NDUFA5	1930.59301	-0.70899293	0.09996591	-7.09234671	1.32E-12	4.44E-12	Down	NDUFA5
MZF1	836.893046	0.92752643	0.13078698	7.09188685	1.32E-12	4.46E-12	Up	MZF1
FGD2	216.92118	-0.98285563	0.13863469	-7.08953607	1.35E-12	4.53E-12	Down	FGD2
UBL3	2187.13776	-0.71625158	0.10102978	-7.08950958	1.35E-12	4.53E-12	Down	UBL3
SARAF	6378.66107	-0.63108877	0.08902389	-7.0889824	1.35E-12	4.55E-12	Down	SARAF
MAML3	594.972527	-0.59635719	0.08413073	-7.08845878	1.36E-12	4.56E-12	Down	MAML3
RAP1B	1681.04133	-0.54412544	0.0767833	-7.08650741	1.38E-12	4.63E-12	Down	RAP1B
AAR2	2153.978	0.56853818	0.0802519	7.08441981	1.40E-12	4.69E-12	Up	AAR2
MED30	518.700811	0.65771017	0.09284348	7.084075	1.40E-12	4.71E-12	Up	MED30
NEIL2	596.746635	0.73648755	0.10397323	7.08343464	1.41E-12	4.73E-12	Up	NEIL2
KAT6B	872.946908	-0.59500608	0.08400266	-7.08318159	1.41E-12	4.73E-12	Down	KAT6B
KCNT2	15.298417	-1.62940989	0.2301679	-7.07922289	1.45E-12	4.87E-12	Down	KCNT2
TMEM92	424.132137	1.08575594	0.15337644	7.07902697	1.45E-12	4.88E-12	Up	TMEM92
PLD6	166.198598	0.74777796	0.10564383	7.07829285	1.46E-12	4.90E-12	Up	PLD6
COL3A1	59241.9412	1.30130076	0.18386257	7.0775729	1.47E-12	4.93E-12	Up	COL3A1
CACNA2D3	22.3393109	-1.25538851	0.17740103	-7.07655704	1.48E-12	4.96E-12	Down	CACNA2D3
POT1	713.732322	0.60231518	0.08512453	7.07569483	1.49E-12	4.99E-12	Up	POT1
KCNV2	4.45735903	2.15550294	0.30476164	7.07275008	1.52E-12	5.10E-12	Up	KCNV2
C7orf50	2581.69975	0.74079854	0.10474365	7.07249116	1.52E-12	5.11E-12	Up	C7orf50
NFE2	45.3714125	1.85534577	0.26236066	7.07173771	1.53E-12	5.13E-12	Up	NFE2
ACOT11	1124.98059	-0.82486137	0.11664944	-7.07128439	1.54E-12	5.15E-12	Down	ACOT11
C22orf39	748.212122	-0.55415768	0.07838252	-7.06991426	1.55E-12	5.20E-12	Down	C22orf39
PPP2R3C	606.869783	-0.56101787	0.07938602	-7.06696014	1.58E-12	5.31E-12	Down	PPP2R3C
MYADM	4341.57196	-0.8392435	0.11883457	-7.06228409	1.64E-12	5.49E-12	Down	MYADM
ARSH	7.81751914	1.77285997	0.25104546	7.06190816	1.64E-12	5.50E-12	Up	ARSH
MKRN2OS	106.423474	-0.84576601	0.11977561	-7.06125404	1.65E-12	5.53E-12	Down	MKRN2OS
COL28A1	134.153489	-1.50022934	0.21248907	-7.0602658	1.66E-12	5.57E-12	Down	COL28A1
UTS2B	5.0641857	-1.83936998	0.26052869	-7.06014364	1.66E-12	5.57E-12	Down	UTS2B
GNA15	418.507402	1.08801531	0.15415518	7.05792246	1.69E-12	5.66E-12	Up	GNA15
FKBP4	7954.57147	0.69058383	0.09785551	7.05717848	1.70E-12	5.69E-12	Up	FKBP4
ITGAL	447.251364	-1.16647894	0.16529761	-7.05684092	1.70E-12	5.70E-12	Down	ITGAL
IL2	0.94878377	-2.04697304	0.2901506	-7.05486406	1.73E-12	5.78E-12	Down	IL2
PTPRM	630.228012	-0.89740542	0.12724098	-7.05280156	1.75E-12	5.87E-12	Down	PTPRM
PF4	83.0466474	1.90274318	0.26988291	7.05025448	1.79E-12	5.97E-12	Up	PF4
LDOC1	113.213314	-1.11358382	0.15799567	-7.04819223	1.81E-12	6.06E-12	Down	LDOC1
GNMT	8.05585805	1.1848218	0.16811061	7.04787064	1.82E-12	6.07E-12	Up	GNMT
KRT35	3.20639614	4.49570564	0.63796058	7.04699595	1.83E-12	6.11E-12	Up	KRT35
SLC35A4	3454.49958	-0.52491159	0.07449692	-7.04608421	1.84E-12	6.15E-12	Down	SLC35A4
LMCD1	643.408897	0.8919071	0.12663533	7.04311405	1.88E-12	6.28E-12	Up	LMCD1
WIF1	158.69116	3.33815878	0.4739753	7.04289604	1.88E-12	6.29E-12	Up	WIF1
RIBC2	62.9889566	1.17411286	0.1667384	7.04164621	1.90E-12	6.34E-12	Up	RIBC2
RPS3	75632.6011	0.78582226	0.11165515	7.03794019	1.95E-12	6.51E-12	Up	RPS3
TRABD	2588.78119	0.8686892	0.12344083	7.03729249	1.96E-12	6.54E-12	Up	TRABD
DRAM1	984.347375	0.73706055	0.10474098	7.03698376	1.96E-12	6.56E-12	Up	DRAM1

RAB42	67.4695002	-1.01969339	0.14492271	-7.03611895	1.98E-12	6.60E-12	Down	RAB42
PDE4DIP	1140.48557	-0.5541314	0.07876312	-7.03541735	1.99E-12	6.63E-12	Down	PDE4DIP
ST6GALNAC	5086.57458	-1.45174818	0.20640216	-7.03359001	2.01E-12	6.71E-12	Down	ST6GALNAC1
DDX52	1501.50617	0.50268824	0.07148219	7.03235637	2.03E-12	6.77E-12	Up	DDX52
PRELID2	581.345871	-0.73059218	0.10389232	-7.03220607	2.03E-12	6.78E-12	Down	PRELID2
CLEC14A	329.533622	-0.83801172	0.11918618	-7.03111474	2.05E-12	6.83E-12	Down	CLEC14A
COBL	1743.15418	-0.73481983	0.1045173	-7.03060472	2.06E-12	6.85E-12	Down	COBL
ECH1	5188.54442	-0.76198346	0.10838248	-7.03050395	2.06E-12	6.85E-12	Down	ECH1
ZFAND4	182.138553	-0.6040316	0.08591584	-7.03050333	2.06E-12	6.85E-12	Down	ZFAND4
BCAP29	1438.31812	-0.5718915	0.08135889	-7.02924389	2.08E-12	6.92E-12	Down	BCAP29
RAPGEFL1	3280.62115	-0.91427638	0.130078	-7.02867817	2.08E-12	6.94E-12	Down	RAPGEFL1
ERICH3	2.63504732	-2.63392419	0.37496284	-7.02449398	2.15E-12	7.15E-12	Down	ERICH3
NCR3LG1	595.172538	1.05215299	0.1497994	7.02374622	2.16E-12	7.19E-12	Up	NCR3LG1
ARGLU1	2663.67044	0.70693874	0.10066375	7.02277402	2.18E-12	7.24E-12	Up	ARGLU1
FCRL1	19.3887429	-2.32131958	0.33057295	-7.02210987	2.19E-12	7.27E-12	Down	FCRL1
ARHGEF1	3937.34862	0.59986508	0.08542663	7.0219916	2.19E-12	7.28E-12	Up	ARHGEF1
CD40LG	32.0653417	-1.21705481	0.17337518	-7.01977519	2.22E-12	7.39E-12	Down	CD40LG
CDH15	10.9113582	1.58571903	0.2259147	7.01910508	2.23E-12	7.42E-12	Up	CDH15
MRPL55	1226.59262	0.99347798	0.14154346	7.01888994	2.24E-12	7.43E-12	Up	MRPL55
SNRPB2	2215.97325	0.718663	0.10239534	7.01851257	2.24E-12	7.45E-12	Up	SNRPB2
WDR24	641.573734	0.97952856	0.13956975	7.01820117	2.25E-12	7.47E-12	Up	WDR24
TRPC1	71.8876418	-1.19084857	0.16974034	-7.0157076	2.29E-12	7.60E-12	Down	TRPC1
GIMAP5	33.3217983	-1.36626547	0.19474371	-7.01571031	2.29E-12	7.60E-12	Down	GIMAP5
DLX5	18.7530191	2.24336369	0.31977717	7.01539661	2.29E-12	7.61E-12	Up	DLX5
LTK	245.35351	-1.66287027	0.23707722	-7.01404485	2.32E-12	7.69E-12	Down	LTK
MAP6D1	69.576262	0.83437546	0.11896914	7.01337699	2.33E-12	7.72E-12	Up	MAP6D1
UGT1A6	48.9909122	1.79058411	0.25544453	7.0096787	2.39E-12	7.93E-12	Up	UGT1A6
NDUFB3	1375.31825	-0.5435617	0.07754658	-7.00948612	2.39E-12	7.94E-12	Down	NDUFB3
POLR2G	1967.92197	0.70075303	0.09998317	7.00870971	2.41E-12	7.98E-12	Up	POLR2G
ALS2	694.324279	0.52328385	0.07467257	7.0077119	2.42E-12	8.03E-12	Up	ALS2
SENP8	92.5167551	-0.64420406	0.09194747	-7.00621862	2.45E-12	8.12E-12	Down	SENP8
FNTA	1963.91437	0.57949476	0.08271318	7.00607502	2.45E-12	8.13E-12	Up	FNTA
UQCC2	1287.42483	0.72962271	0.10415099	7.00543244	2.46E-12	8.16E-12	Up	UQCC2
SEMA4F	312.437303	0.66654693	0.09515838	7.00460574	2.48E-12	8.21E-12	Up	SEMA4F
FOXD4	10.5261214	1.46064183	0.20856302	7.00335948	2.50E-12	8.28E-12	Up	FOXD4
PLEKHB1	1559.0093	1.23525625	0.17638782	7.00306984	2.50E-12	8.30E-12	Up	PLEKHB1
TNFRSF11A	1397.05813	-1.04767509	0.14961919	-7.00227738	2.52E-12	8.34E-12	Down	TNFRSF11A
LSM4	5053.2633	0.59129193	0.08446416	7.00050684	2.55E-12	8.45E-12	Up	LSM4
AFF2	9.97125255	-1.81672237	0.25955993	-6.99924062	2.57E-12	8.52E-12	Down	AFF2
RIMKLB	179.669441	-1.26516562	0.1807686	-6.99881316	2.58E-12	8.55E-12	Down	RIMKLB
GPR183	260.378152	-1.22609549	0.17523806	-6.99674194	2.62E-12	8.67E-12	Down	GPR183
CTSF	593.408108	-1.10114683	0.15738853	-6.99636002	2.63E-12	8.69E-12	Down	CTSF
ZNF267	568.993582	0.69297664	0.09904806	6.99636785	2.63E-12	8.69E-12	Up	ZNF267
CXCL13	243.395702	-1.9100775	0.27307783	-6.99462674	2.66E-12	8.80E-12	Down	CXCL13
PLK5	8.7088921	2.16491193	0.30957829	6.99309997	2.69E-12	8.89E-12	Up	PLK5
OR51E1	128.464152	1.42840691	0.20428474	6.99223489	2.71E-12	8.95E-12	Up	OR51E1
INHA	7.08241883	2.44862367	0.35023031	6.99146705	2.72E-12	8.99E-12	Up	INHA
FOXI2	2.72384091	-1.82387929	0.26096001	-6.98911405	2.77E-12	9.14E-12	Down	FOXI2
CA5A	3.43133632	2.08780434	0.2987422	6.98864878	2.78E-12	9.17E-12	Up	CA5A
ECHDC1	2179.70997	-0.55923788	0.08002887	-6.98795128	2.79E-12	9.21E-12	Down	ECHDC1
RPL7A	54217.8618	0.68597781	0.09818226	6.98677931	2.81E-12	9.29E-12	Up	RPL7A
DOCK1	2130.71986	-0.53993567	0.07731839	-6.98327628	2.88E-12	9.52E-12	Down	DOCK1
ZNRF2	1039.89553	-0.61325932	0.08783811	-6.98169963	2.92E-12	9.63E-12	Down	ZNRF2
AQP2	2.64208736	3.19389531	0.45757161	6.98009939	2.95E-12	9.74E-12	Up	AQP2
CCDC40	94.05158	0.92635884	0.13272129	6.97973031	2.96E-12	9.76E-12	Up	CCDC40
KCNH1	7.4787	-1.28424363	0.18404877	-6.97773544	3.00E-12	9.90E-12	Down	KCNH1
DST	3985.20646	-1.00847205	0.14455857	-6.97621772	3.03E-12	1.00E-11	Down	DST
UNC5B	1421.92683	-0.90768527	0.13011904	-6.9758066	3.04E-12	1.00E-11	Down	UNC5B

SYCE2	26.1658683	0.98934944	0.14187497	6.97338952	3.09E-12	1.02E-11	Up	SYCE2
RBP2	189.525633	2.08275483	0.29879714	6.9704643	3.16E-12	1.04E-11	Up	RBP2
FARP1	3389.57105	0.89623479	0.1285814	6.97017469	3.17E-12	1.04E-11	Up	FARP1
SGPP2	1598.90309	-0.79175778	0.11363842	-6.96734256	3.23E-12	1.06E-11	Down	SGPP2
KLHL10	1.82422527	-1.61659837	0.23206941	-6.96601222	3.26E-12	1.07E-11	Down	KLHL10
CRTAC1	26.278982	-2.042276	0.29325917	-6.96406532	3.31E-12	1.09E-11	Down	CRTAC1
DNAJC13	1783.00279	-0.5082153	0.0729893	-6.96287407	3.33E-12	1.10E-11	Down	DNAJC13
PLCL1	94.9940833	-1.1511985	0.16533573	-6.9627932	3.34E-12	1.10E-11	Down	PLCL1
CCAR1	2339.82648	0.50077108	0.07192882	6.96203634	3.35E-12	1.10E-11	Up	CCAR1
CD207	22.4411682	-1.42435404	0.20459979	-6.96165931	3.36E-12	1.11E-11	Down	CD207
CYB5B	6428.78072	0.6452643	0.09269855	6.96088876	3.38E-12	1.11E-11	Up	CYB5B
DMGDH	17.7822541	-1.15348811	0.16575555	-6.95897135	3.43E-12	1.13E-11	Down	DMGDH
ATP7B	1293.99219	0.82681916	0.1188191	6.95863849	3.44E-12	1.13E-11	Up	ATP7B
TMC6	2918.75221	0.79976512	0.11495075	6.95745886	3.46E-12	1.14E-11	Up	TMC6
PCDHB14	256.729716	0.93993953	0.13510701	6.95700027	3.48E-12	1.14E-11	Up	PCDHB14
IRGQ	1092.65893	0.52847839	0.07596554	6.95681774	3.48E-12	1.14E-11	Up	IRGQ
RDH12	12.7449965	1.33760329	0.19229954	6.95583204	3.50E-12	1.15E-11	Up	RDH12
FRRS1	174.231636	-0.96040236	0.13811071	-6.95385853	3.55E-12	1.17E-11	Down	FRRS1
MOB2	1165.15671	-0.60425596	0.08690141	-6.95335072	3.57E-12	1.17E-11	Down	MOB2
SLC16A1	4233.94167	-0.76174604	0.10957675	-6.95171244	3.61E-12	1.19E-11	Down	SLC16A1
CNTNAP1	305.63979	-1.13194823	0.16283258	-6.9516076	3.61E-12	1.19E-11	Down	CNTNAP1
FAM219A	606.409456	0.60424375	0.08696161	6.94839615	3.69E-12	1.21E-11	Up	FAM219A
IL17RB	933.564315	0.8270232	0.11903586	6.94768124	3.71E-12	1.22E-11	Up	IL17RB
LRCH1	1481.11794	0.65964372	0.09495388	6.94699082	3.73E-12	1.23E-11	Up	LRCH1
ACTRT3	68.4168561	0.63406245	0.09128395	6.94604553	3.76E-12	1.23E-11	Up	ACTRT3
G6PD	1998.03581	0.71775497	0.10338821	6.94232876	3.86E-12	1.27E-11	Up	G6PD
KRT18	48947.9002	0.78015452	0.11242021	6.93962899	3.93E-12	1.29E-11	Up	KRT18
FRYL	3657.11217	-0.8275631	0.11927897	-6.93804724	3.98E-12	1.30E-11	Down	FRYL
PML	2903.65538	0.69599321	0.10033337	6.93680699	4.01E-12	1.31E-11	Up	PML
EIF4A1	269.034375	0.79074724	0.11405916	6.93278131	4.13E-12	1.35E-11	Up	EIF4A1
CCDC107	192.413647	-0.75021112	0.10823095	-6.93157636	4.16E-12	1.36E-11	Down	CCDC107
SMARCA2	2700.12043	-0.58911129	0.08499033	-6.93150981	4.16E-12	1.36E-11	Down	SMARCA2
SARS2	217.584472	0.63611738	0.09177774	6.93106411	4.18E-12	1.37E-11	Up	SARS2
FOXO1	1004.37626	-0.81174875	0.11712333	-6.9307179	4.19E-12	1.37E-11	Down	FOXO1
VRK1	728.874495	0.62412448	0.09006403	6.92978629	4.21E-12	1.38E-11	Up	VRK1
ME1	1035.77989	0.9723398	0.14032542	6.92917772	4.23E-12	1.39E-11	Up	ME1
GBP7	5.17067043	2.34017388	0.33782734	6.92712984	4.29E-12	1.41E-11	Up	GBP7
CD99L2	1076.62199	-0.58980329	0.08515179	-6.92649331	4.31E-12	1.41E-11	Down	CD99L2
MGAM	45.0989626	-1.56641219	0.22621118	-6.92455704	4.37E-12	1.43E-11	Down	MGAM
PDE1B	69.5997759	-1.24429354	0.17977764	-6.92129195	4.48E-12	1.46E-11	Down	PDE1B
SETD9	269.581096	-0.71610261	0.10348965	-6.91955766	4.53E-12	1.48E-11	Down	SETD9
NRG4	17.0203755	-1.46171815	0.21133809	-6.91649167	4.63E-12	1.51E-11	Down	NRG4
CD4	1198.83586	-1.00941958	0.14595026	-6.91618891	4.64E-12	1.52E-11	Down	CD4
WDFY4	251.232752	-1.20743071	0.17458644	-6.91594771	4.65E-12	1.52E-11	Down	WDFY4
CDIP1	537.625982	-1.07861784	0.15596374	-6.91582461	4.65E-12	1.52E-11	Down	CDIP1
SYT9	2.50903398	-2.41477304	0.34925087	-6.91415029	4.71E-12	1.54E-11	Down	SYT9
GRB10	1302.41902	0.72575864	0.10501149	6.91123042	4.80E-12	1.57E-11	Up	GRB10
RUNX1T1	132.724541	-1.36440985	0.19743706	-6.91060653	4.83E-12	1.58E-11	Down	RUNX1T1
SOX17	62.70663	-1.03636928	0.1499969	-6.90927148	4.87E-12	1.59E-11	Down	SOX17
GJB6	8.37713186	2.49657226	0.36137981	6.90844425	4.90E-12	1.60E-11	Up	GJB6
NRAP	17.0849421	-2.16878718	0.31395693	-6.90791309	4.92E-12	1.60E-11	Down	NRAP
NFKBIA	2991.62528	-0.61800833	0.08946603	-6.90774273	4.92E-12	1.61E-11	Down	NFKBIA
BDNF	30.890553	1.22848932	0.17784366	6.90769258	4.93E-12	1.61E-11	Up	BDNF
CABYR	29.5231011	0.99665027	0.14433771	6.90498895	5.02E-12	1.64E-11	Up	CABYR
DUSP19	87.5062319	-0.79048972	0.11449467	-6.90416153	5.05E-12	1.65E-11	Down	DUSP19
TWIST2	52.3241435	-1.34017965	0.19415368	-6.90267446	5.10E-12	1.66E-11	Down	TWIST2
UROC1	3.01829501	2.8405538	0.41152064	6.9025791	5.11E-12	1.66E-11	Up	UROC1
MRPS34	3818.9044	0.8555536	0.12396997	6.90129706	5.15E-12	1.68E-11	Up	MRPS34

CYP2J2	1151.48847	-0.76077274	0.11024163	-6.90095695	5.17E-12	1.68E-11	Down	CYP2J2
SPATA31E1	0.78003705	-2.64481233	0.3832938	-6.90022206	5.19E-12	1.69E-11	Down	SPATA31E1
IL27RA	505.709811	0.81835047	0.11860907	6.89956076	5.22E-12	1.70E-11	Up	IL27RA
CLN6	2421.12841	0.59705559	0.08656951	6.89683442	5.32E-12	1.73E-11	Up	CLN6
FAM131A	473.670155	-0.57069577	0.08278444	-6.89375654	5.43E-12	1.77E-11	Down	FAM131A
MORN3	28.101101	1.54052802	0.22352776	6.89188671	5.51E-12	1.79E-11	Up	MORN3
IFT81	290.026123	0.58991047	0.08560858	6.89078669	5.55E-12	1.80E-11	Up	IFT81
AASDHPPT	1067.38267	0.52736426	0.07653197	6.89077052	5.55E-12	1.80E-11	Up	AASDHPPT
SHOX2	18.8101716	1.86624238	0.2708313	6.89079295	5.55E-12	1.80E-11	Up	SHOX2
CCL5	692.824436	-1.32083133	0.19172587	-6.88916577	5.61E-12	1.82E-11	Down	CCL5
DNAH10	57.5419923	1.32410563	0.19222393	6.88834959	5.64E-12	1.83E-11	Up	DNAH10
TAS1R1	4.26939321	-1.8926497	0.27485501	-6.88599322	5.74E-12	1.86E-11	Down	TAS1R1
TMED3	4270.01934	0.65230267	0.09473068	6.88586499	5.74E-12	1.87E-11	Up	TMED3
AHSA1	4475.61405	0.50837826	0.07383458	6.88536842	5.76E-12	1.87E-11	Up	AHSA1
CSF2RB	465.863217	-1.21856679	0.17699396	-6.8847929	5.79E-12	1.88E-11	Down	CSF2RB
NBPF9	224.483928	0.60694154	0.08816634	6.8840504	5.82E-12	1.89E-11	Up	NBPF9
CALB2	137.144444	-2.00426749	0.29121538	-6.88242329	5.88E-12	1.91E-11	Down	CALB2
SCAPER	396.789082	-0.59371952	0.08629983	-6.87973018	6.00E-12	1.95E-11	Down	SCAPER
PROCA1	68.234054	0.96968776	0.14096256	6.87904474	6.03E-12	1.95E-11	Up	PROCA1
FGF17	6.72517031	1.81097576	0.26330246	6.87792959	6.07E-12	1.97E-11	Up	FGF17
ARMC3	4.87770473	2.26950382	0.32997198	6.87786836	6.08E-12	1.97E-11	Up	ARMC3
CR1	74.9411818	-1.6476318	0.23957095	-6.87742734	6.09E-12	1.98E-11	Down	CR1
TRIOBP	953.438141	-0.51402907	0.07474825	-6.87680444	6.12E-12	1.98E-11	Down	TRIOBP
RCN3	1007.24883	0.97951613	0.14246748	6.87536659	6.18E-12	2.00E-11	Up	RCN3
MESP2	22.3003156	-1.31693068	0.19155301	-6.87501933	6.20E-12	2.01E-11	Down	MESP2
BPIFB2	4.73602687	4.61126622	0.67082249	6.87404836	6.24E-12	2.02E-11	Up	BPIFB2
C12orf75	1939.35998	0.70385241	0.10239922	6.87361078	6.26E-12	2.03E-11	Up	C12orf75
PIGN	923.971137	-0.56680665	0.08247725	-6.87227885	6.32E-12	2.05E-11	Down	PIGN
CCR7	108.923168	-1.32295975	0.19250774	-6.872242	6.32E-12	2.05E-11	Down	CCR7
GEMIN6	481.888529	0.53326207	0.07760732	6.87128555	6.36E-12	2.06E-11	Up	GEMIN6
SLA2	69.6523581	-1.04924314	0.15278362	-6.86751044	6.53E-12	2.11E-11	Down	SLA2
MED11	411.197859	-0.59396736	0.08649258	-6.8672641	6.54E-12	2.12E-11	Down	MED11
NQO1	7178.95095	0.97955634	0.14265927	6.86640518	6.58E-12	2.13E-11	Up	NQO1
TPP2	2169.2494	0.56158762	0.08179244	6.86600862	6.60E-12	2.13E-11	Up	TPP2
CD55	5659.78198	1.12818444	0.16436743	6.86379567	6.71E-12	2.17E-11	Up	CD55
CYSLTR2	27.5370789	-1.31022758	0.1909233	-6.86258623	6.76E-12	2.19E-11	Down	CYSLTR2
HDC	40.9089082	-1.3961415	0.20345236	-6.86225252	6.78E-12	2.19E-11	Down	HDC
SHOX	4.95579449	-1.68944138	0.24619859	-6.86210825	6.79E-12	2.19E-11	Down	SHOX
CBWD5	149.908594	0.79687851	0.11613934	6.86140063	6.82E-12	2.20E-11	Up	CBWD5
TINAG	285.737748	-1.37710877	0.20070817	-6.86124933	6.83E-12	2.20E-11	Down	TINAG
PROSER3	438.907303	0.53748799	0.07833698	6.86122893	6.83E-12	2.20E-11	Up	PROSER3
ENKD1	471.046178	0.70686203	0.1030293	6.86078677	6.85E-12	2.21E-11	Up	ENKD1
APLP1	63.6204402	-1.36140864	0.19845762	-6.85994654	6.89E-12	2.22E-11	Down	APLP1
ADRA1B	12.0046694	-1.49011957	0.21733605	-6.85629282	7.07E-12	2.28E-11	Down	ADRA1B
TMOD3	2803.95715	-0.58011052	0.0846114	-6.85617474	7.07E-12	2.28E-11	Down	TMOD3
ADAM8	624.050672	1.17207914	0.17101202	6.85378235	7.19E-12	2.32E-11	Up	ADAM8
NCR3	13.3605725	-1.32085493	0.19272581	-6.85354465	7.20E-12	2.32E-11	Down	NCR3
CATIP	5.81749744	1.4259654	0.20811973	6.85165884	7.30E-12	2.35E-11	Up	CATIP
CCDC85A	10.9553916	-1.5602343	0.22784927	-6.84765995	7.51E-12	2.42E-11	Down	CCDC85A
GZF1	737.735215	0.58991534	0.08616271	6.84652732	7.57E-12	2.44E-11	Up	GZF1
SCTR	9.33296063	-1.71093506	0.24990481	-6.84634706	7.58E-12	2.44E-11	Down	SCTR
SORCS3	2.98191221	-2.75454694	0.4024116	-6.84509827	7.64E-12	2.46E-11	Down	SORCS3
ERV3-1	344.151712	1.09000272	0.15924803	6.84468574	7.66E-12	2.47E-11	Up	ERV3-1
FAM83G	1647.42832	-0.59599421	0.08708439	-6.84386924	7.71E-12	2.48E-11	Down	FAM83G
TALDO1	7008.34672	0.55941818	0.08176378	6.84188222	7.82E-12	2.52E-11	Up	TALDO1
CENPT	936.035397	0.60306259	0.08818714	6.83844175	8.01E-12	2.58E-11	Up	CENPT
MFSD3	1346.4918	1.00390465	0.14682843	6.83726328	8.07E-12	2.60E-11	Up	MFSD3
TTYH2	139.456588	-0.92968681	0.13599453	-6.8362076	8.13E-12	2.62E-11	Down	TTYH2

NEK1	309.244664	-0.70028568	0.10247364	-6.83381316	8.27E-12	2.66E-11	Down	NEK1
MLST8	2214.17086	0.77845562	0.11391293	6.8337778	8.27E-12	2.66E-11	Up	MLST8
ADCK5	943.281805	0.90163575	0.13197123	6.83206309	8.37E-12	2.69E-11	Up	ADCK5
LHB	3.1650982	2.13209395	0.31207872	6.83191069	8.38E-12	2.69E-11	Up	LHB
TLX3	3.60236623	4.04798456	0.59260336	6.83084978	8.44E-12	2.71E-11	Up	TLX3
CSTF2	1120.80986	0.53110954	0.07775334	6.8306976	8.45E-12	2.72E-11	Up	CSTF2
GIPC1	7166.36269	-0.72955325	0.10681604	-6.82999689	8.49E-12	2.73E-11	Down	GIPC1
ACOT13	1110.59924	-0.5981206	0.08757748	-6.82961678	8.51E-12	2.74E-11	Down	ACOT13
P2RY2	266.978143	-0.91803964	0.13441999	-6.82963631	8.51E-12	2.74E-11	Down	P2RY2
C12orf29	636.818636	0.62218302	0.09112589	6.82773065	8.63E-12	2.77E-11	Up	C12orf29
ACTR10	1387.74177	-0.50020156	0.07328654	-6.82528514	8.78E-12	2.82E-11	Down	ACTR10
PPTC7	1452.82659	-0.51399029	0.07535201	-6.82118888	9.03E-12	2.90E-11	Down	PPTC7
SOCS7	944.444767	0.64289038	0.09428119	6.81886131	9.18E-12	2.94E-11	Up	SOCS7
MAGEA4	16.3313064	6.24891816	0.91658971	6.81757396	9.26E-12	2.97E-11	Up	MAGEA4
UTP23	792.002742	0.6176676	0.09061164	6.81664746	9.32E-12	2.99E-11	Up	UTP23
ZNF275	942.354012	0.56380406	0.08272978	6.81500701	9.43E-12	3.02E-11	Up	ZNF275
ZNF415	98.2477068	-1.3705358	0.2011055	-6.81500911	9.43E-12	3.02E-11	Down	ZNF415
NRROS	128.912997	-0.91030632	0.13357512	-6.81493917	9.43E-12	3.02E-11	Down	NRROS
RPL35	23021.0339	0.8216281	0.12061647	6.81190636	9.63E-12	3.09E-11	Up	RPL35
KCNK5	1788.06191	-0.83625783	0.12277306	-6.81141144	9.66E-12	3.10E-11	Down	KCNK5
ICAM5	26.8564711	1.48999611	0.21882645	6.80903102	9.83E-12	3.15E-11	Up	ICAM5
RGS20	7.62923835	1.9492529	0.2862869	6.80873943	9.85E-12	3.15E-11	Up	RGS20
GAPDH	115169.587	0.73097392	0.10736706	6.80817646	9.88E-12	3.16E-11	Up	GAPDH
EVI2A	160.27637	-1.33873718	0.19665714	-6.80746791	9.93E-12	3.18E-11	Down	EVI2A
CCR9	7.66635618	-1.57306851	0.23109603	-6.80699056	9.97E-12	3.19E-11	Down	CCR9
COL23A1	36.4444732	-1.07258405	0.15759056	-6.80614414	1.00E-11	3.21E-11	Down	COL23A1
ARHGEF28	654.40729	0.71949371	0.10572917	6.8050637	1.01E-11	3.23E-11	Up	ARHGEF28
RPL26L1	594.712661	0.60122676	0.08835356	6.80478285	1.01E-11	3.24E-11	Up	RPL26L1
ZNF587	1085.05843	0.67581554	0.09931616	6.80468841	1.01E-11	3.24E-11	Up	ZNF587
WDTC1	2620.8755	-0.59067749	0.08681079	-6.80419438	1.02E-11	3.25E-11	Down	WDTC1
PFDN6	1324.65843	0.62566005	0.09198115	6.80204674	1.03E-11	3.29E-11	Up	PFDN6
UNC80	5.35349671	-2.18200384	0.3207997	-6.80176391	1.03E-11	3.30E-11	Down	UNC80
FAM189A1	172.641988	-1.92636075	0.28325623	-6.80077105	1.04E-11	3.32E-11	Down	FAM189A1
PKD1L3	13.1917302	1.70372492	0.25053201	6.80042803	1.04E-11	3.33E-11	Up	PKD1L3
RAD51	572.324234	0.65086668	0.09572913	6.79904485	1.05E-11	3.36E-11	Up	RAD51
EIF2A	3930.27397	0.5117814	0.07529741	6.79679941	1.07E-11	3.41E-11	Up	EIF2A
KCNQ5	12.276359	-2.02572041	0.29804921	-6.7965971	1.07E-11	3.42E-11	Down	KCNQ5
JMJD7	7.07365134	1.27555186	0.18771359	6.79520238	1.08E-11	3.45E-11	Up	JMJD7
E2F8	456.66384	0.62072376	0.09134974	6.79502521	1.08E-11	3.45E-11	Up	E2F8
HSPA8	50380.8828	0.54675148	0.08047903	6.79371345	1.09E-11	3.48E-11	Up	HSPA8
FGF23	4.00479813	4.06196219	0.59798175	6.79278625	1.10E-11	3.51E-11	Up	FGF23
PIWIL4	375.968621	0.77971344	0.11478834	6.792619	1.10E-11	3.51E-11	Up	PIWIL4
SNCB	2.48178718	-2.11299387	0.31109099	-6.79220524	1.10E-11	3.52E-11	Down	SNCB
COX5A	5741.6912	-0.64539338	0.09502066	-6.79213717	1.10E-11	3.52E-11	Down	COX5A
PHKG1	42.311013	-0.91529824	0.13477527	-6.79129199	1.11E-11	3.54E-11	Down	PHKG1
KATNB1	1253.83431	0.62117321	0.09148223	6.79009662	1.12E-11	3.57E-11	Up	KATNB1
CCDC167	481.226624	0.72757664	0.10715815	6.78974623	1.12E-11	3.58E-11	Up	CCDC167
CD300LG	10.1403674	-2.79507646	0.41177599	-6.78785679	1.14E-11	3.62E-11	Down	CD300LG
SEC23B	3691.10805	0.56912642	0.0838489	6.78752363	1.14E-11	3.63E-11	Up	SEC23B
PCK2	3967.75186	-0.74001147	0.10902915	-6.78728097	1.14E-11	3.63E-11	Down	PCK2
C16orf96	1.89541594	-1.44900807	0.21353766	-6.78572624	1.16E-11	3.67E-11	Down	C16orf96
ARHGAP30	536.098516	-1.01030097	0.1489178	-6.78428632	1.17E-11	3.71E-11	Down	ARHGAP30
MCU	2326.59341	-0.57381827	0.08458281	-6.78409992	1.17E-11	3.71E-11	Down	MCU
TNFSF4	161.90004	1.2527581	0.18467154	6.78370954	1.17E-11	3.72E-11	Up	TNFSF4
SIRT6	1321.50479	-0.70064091	0.10328773	-6.78338957	1.17E-11	3.73E-11	Down	SIRT6
UBB	16255.28	-0.54206904	0.07991134	-6.78338036	1.17E-11	3.73E-11	Down	UBB
NRG1	98.9041516	-1.40227466	0.20678729	-6.78124215	1.19E-11	3.78E-11	Down	NRG1
SEMG1	40.2190741	2.88972996	0.4263202	6.77830884	1.22E-11	3.86E-11	Up	SEMG1

CAMK1D	1416.34641	-1.01094933	0.14918416	-6.77651912	1.23E-11	3.91E-11	Down	CAMK1D
CROCC	688.717685	0.70558823	0.10412406	6.77641882	1.23E-11	3.91E-11	Up	CROCC
P4HTM	1829.9151	0.67833276	0.10010711	6.77606969	1.23E-11	3.92E-11	Up	P4HTM
WDR31	206.270887	0.58286794	0.08603545	6.77474169	1.25E-11	3.95E-11	Up	WDR31
MTDH	5868.75714	0.51349477	0.07580394	6.77398524	1.25E-11	3.97E-11	Up	MTDH
WDR35	468.245733	0.79980191	0.118151	6.76931967	1.29E-11	4.10E-11	Up	WDR35
ZNF444	1207.64776	0.64656442	0.0955425	6.76729668	1.31E-11	4.16E-11	Up	ZNF444
ZSCAN31	239.483746	-0.6447378	0.0952738	-6.76720969	1.31E-11	4.16E-11	Down	ZSCAN31
NOS3	551.7401	0.90168014	0.13326841	6.7658953	1.32E-11	4.20E-11	Up	NOS3
THTPA	162.636035	-0.56379957	0.08332947	-6.76590836	1.32E-11	4.20E-11	Down	THTPA
TRIM7	242.872475	1.84764129	0.27309968	6.76544652	1.33E-11	4.21E-11	Up	TRIM7
NRP2	1249.92485	-1.13061508	0.16713116	-6.76483719	1.33E-11	4.22E-11	Down	NRP2
NUDC	4157.5708	0.54928132	0.08120607	6.76404292	1.34E-11	4.25E-11	Up	NUDC
COPS6	3864.1211	0.60165168	0.08896891	6.76249337	1.36E-11	4.29E-11	Up	COPS6
LYL1	113.089615	-0.88141285	0.13035667	-6.76154763	1.37E-11	4.32E-11	Down	LYL1
AMPH	42.2771441	-1.11334225	0.16470967	-6.75942244	1.39E-11	4.38E-11	Down	AMPH
PBK	762.680231	0.94754426	0.14023171	6.75699023	1.41E-11	4.46E-11	Up	PBK
PRRX1	681.466034	1.41922829	0.21008399	6.75552801	1.42E-11	4.50E-11	Up	PRRX1
CNTNAP3B	10.3701539	-1.88055561	0.27838179	-6.75531116	1.43E-11	4.51E-11	Down	CNTNAP3B
IQCC	141.810992	0.53315935	0.07893501	6.75440913	1.43E-11	4.53E-11	Up	IQCC
LRRC4B	44.1685158	-1.09264901	0.16177931	-6.75394787	1.44E-11	4.55E-11	Down	LRRC4B
FAM186B	20.9385566	0.95768606	0.14185888	6.750977	1.47E-11	4.64E-11	Up	FAM186B
LRRC4C	14.306631	-1.87951996	0.27847419	-6.74935064	1.49E-11	4.69E-11	Down	LRRC4C
RPA4	6.8239804	1.63052265	0.24158682	6.74922023	1.49E-11	4.69E-11	Up	RPA4
CFAP97	1503.53251	0.64972643	0.09630149	6.74679534	1.51E-11	4.77E-11	Up	CFAP97
ANKMY2	743.178358	-0.51342479	0.07611036	-6.74579407	1.52E-11	4.80E-11	Down	ANKMY2
EPDR1	1131.18094	1.05173443	0.15593896	6.74452654	1.54E-11	4.85E-11	Up	EPDR1
PPP2R2C	187.413134	1.97359752	0.29263845	6.74414966	1.54E-11	4.86E-11	Up	PPP2R2C
ARHGAP36	1.94520542	-2.54924646	0.37811793	-6.74193495	1.56E-11	4.93E-11	Down	ARHGAP36
CD200R1L	2.19692334	3.4292962	0.50865377	6.74190659	1.56E-11	4.93E-11	Up	CD200R1L
KIF25	20.545086	1.73989165	0.25808883	6.74144493	1.57E-11	4.95E-11	Up	KIF25
ANKRD9	1729.28272	-0.91264614	0.13539818	-6.74046085	1.58E-11	4.98E-11	Down	ANKRD9
EIF3G	5838.49872	0.60445559	0.08968115	6.74005148	1.58E-11	4.99E-11	Up	EIF3G
DLC1	629.949718	-0.90863897	0.13486738	-6.73727767	1.61E-11	5.09E-11	Down	DLC1
CTSW	103.785345	-1.37696918	0.20439477	-6.73681233	1.62E-11	5.10E-11	Down	CTSW
CEP70	499.387606	-0.76593577	0.11372053	-6.73524604	1.64E-11	5.16E-11	Down	CEP70
MPDU1	2257.22506	-0.63138742	0.09375506	-6.73443563	1.65E-11	5.18E-11	Down	MPDU1
CPB2	2.34034839	2.7960025	0.41520361	6.73405157	1.65E-11	5.20E-11	Up	CPB2
BHLHE41	696.075486	-1.14910199	0.17071051	-6.73129015	1.68E-11	5.29E-11	Down	BHLHE41
CNPY4	259.229014	-0.55094452	0.08186282	-6.73009432	1.70E-11	5.34E-11	Down	CNPY4
OLFML3	773.682552	-1.1226456	0.16683883	-6.72892274	1.71E-11	5.38E-11	Down	OLFML3
LAX1	69.0976162	-1.37674338	0.20460386	-6.72882406	1.71E-11	5.38E-11	Down	LAX1
PLA2G2F	41.5948973	2.56129116	0.38069479	6.72793858	1.72E-11	5.41E-11	Up	PLA2G2F
AANAT	7.43166475	1.51378793	0.22500736	6.72772642	1.72E-11	5.42E-11	Up	AANAT
CD247	152.551471	-1.00133605	0.14888124	-6.72573699	1.75E-11	5.49E-11	Down	CD247
DERL3	607.857974	-1.16447761	0.1731484	-6.7253154	1.75E-11	5.51E-11	Down	DERL3
GCM1	5.15159234	2.47197149	0.36776077	6.72168345	1.80E-11	5.65E-11	Up	GCM1
RPAP3	1005.74652	0.51241285	0.07623611	6.72139295	1.80E-11	5.66E-11	Up	RPAP3
PTGDS	620.806443	-1.35827486	0.20208754	-6.72122033	1.80E-11	5.66E-11	Down	PTGDS
CDC34	2669.48589	0.6521329	0.09703196	6.72080494	1.81E-11	5.68E-11	Up	CDC34
TIAM1	148.03617	-1.23732816	0.18415771	-6.71885063	1.83E-11	5.75E-11	Down	TIAM1
ADH1A	2.42217377	-2.38355046	0.35480681	-6.71788247	1.84E-11	5.79E-11	Down	ADH1A
AKNA	1624.79156	-0.54798058	0.0815918	-6.71612333	1.87E-11	5.86E-11	Down	AKNA
TMIE	9.10433221	-1.30011441	0.19360267	-6.71537439	1.88E-11	5.89E-11	Down	TMIE
FSIP2	207.944842	-1.90384674	0.2835452	-6.71443825	1.89E-11	5.92E-11	Down	FSIP2
MTG1	249.222618	0.63787996	0.09501363	6.71356273	1.90E-11	5.96E-11	Up	MTG1
GPR108	2190.77843	-0.57540197	0.08570905	-6.71343321	1.90E-11	5.96E-11	Down	GPR108
PGGT1B	920.842444	-0.67202164	0.10011672	-6.71238191	1.91E-11	6.00E-11	Down	PGGT1B

STON1-GTF2	2.43199746	-2.32855486	0.34693944	-6.71170408	1.92E-11	6.03E-11	Down	STON1-GTF2A1L
SAMD15	18.5817783	0.92845386	0.1383347	6.71164852	1.92E-11	6.03E-11	Up	SAMD15
CELA3B	4.87178468	-2.57514152	0.38378863	-6.70979099	1.95E-11	6.11E-11	Down	CELA3B
FANCD2OS	2.57645405	2.04643858	0.30500483	6.70952837	1.95E-11	6.12E-11	Up	FANCD2OS
LEAP2	64.5015187	-1.06839014	0.15923634	-6.70946186	1.95E-11	6.12E-11	Down	LEAP2
LRRC10B	102.308342	1.3830457	0.20615596	6.70873491	1.96E-11	6.15E-11	Up	LRRC10B
SLC10A2	3.17860159	-4.64381132	0.69232633	-6.70754691	1.98E-11	6.20E-11	Down	SLC10A2
KCNT1	33.1661339	2.03188158	0.30298392	6.7062358	2.00E-11	6.25E-11	Up	KCNT1
EPHA3	147.422902	-1.27486124	0.1901774	-6.70353718	2.03E-11	6.37E-11	Down	EPHA3
SKAP2	1647.40697	-0.70752445	0.10555371	-6.70298065	2.04E-11	6.39E-11	Down	SKAP2
ANKS4B	1787.9563	-0.68286286	0.10189843	-6.70140679	2.06E-11	6.46E-11	Down	ANKS4B
DTX3	303.431332	-0.9707258	0.14485729	-6.70125602	2.07E-11	6.46E-11	Down	DTX3
TMPO	5268.07146	0.55082866	0.08221269	6.70004388	2.08E-11	6.52E-11	Up	TMPO
TMEM98	3014.61157	-0.69824229	0.10422274	-6.69951936	2.09E-11	6.54E-11	Down	TMEM98
MYBPC2	10.5561659	-1.48468877	0.22170812	-6.69659186	2.13E-11	6.67E-11	Down	MYBPC2
ZNF48	588.027931	0.50878849	0.07598741	6.69569489	2.15E-11	6.71E-11	Up	ZNF48
SPATA13	2179.08029	0.66126332	0.0987717	6.69486612	2.16E-11	6.75E-11	Up	SPATA13
LIPM	89.4552911	0.94737315	0.14150899	6.69479132	2.16E-11	6.75E-11	Up	LIPM
MYO3B	22.3700277	1.6019088	0.23943717	6.69030969	2.23E-11	6.96E-11	Up	MYO3B
PCSK1	903.521075	2.41542451	0.36105032	6.68999418	2.23E-11	6.97E-11	Up	PCSK1
ANXA10	28.660165	3.13511179	0.46864343	6.6897594	2.24E-11	6.98E-11	Up	ANXA10
STARD8	150.238679	-0.85048486	0.12714615	-6.68903372	2.25E-11	7.02E-11	Down	STARD8
C1QTNF6	634.676354	0.76031091	0.11367976	6.68818159	2.26E-11	7.05E-11	Up	C1QTNF6
ZDHHC24	914.615986	0.61210709	0.09153713	6.68698161	2.28E-11	7.11E-11	Up	ZDHHC24
DLK2	44.8736415	0.88328924	0.13210077	6.68648088	2.29E-11	7.13E-11	Up	DLK2
SEC16B	22.1868193	1.20615735	0.18040179	6.68594981	2.29E-11	7.16E-11	Up	SEC16B
PDZD8	3177.15385	0.66607544	0.09962482	6.68583854	2.30E-11	7.16E-11	Up	PDZD8
SOX5	35.3668309	-1.31363797	0.1965377	-6.68389808	2.33E-11	7.25E-11	Down	SOX5
KIF4B	3.39659004	1.76031573	0.26342673	6.68237324	2.35E-11	7.33E-11	Up	KIF4B
TBC1D12	551.525115	-0.52639831	0.07878141	-6.68175748	2.36E-11	7.36E-11	Down	TBC1D12
IL13RA2	37.4579807	1.4435761	0.21606023	6.68135975	2.37E-11	7.38E-11	Up	IL13RA2
ANKRD12	857.203745	-0.87385861	0.13084266	-6.6786981	2.41E-11	7.51E-11	Down	ANKRD12
SERPINB3	6.16496898	4.68415949	0.70161684	6.67623579	2.45E-11	7.63E-11	Up	SERPINB3
PPFIA4	55.5758057	1.28824732	0.1929606	6.67621949	2.45E-11	7.63E-11	Up	PPFIA4
CYB5R2	285.969798	0.88119622	0.13199617	6.67592258	2.46E-11	7.65E-11	Up	CYB5R2
SYTL1	721.156478	1.18643423	0.17772697	6.67560022	2.46E-11	7.66E-11	Up	SYTL1
SUGT1	2445.54859	0.65364033	0.09792965	6.67459086	2.48E-11	7.71E-11	Up	SUGT1
FAM156A	7.40426391	0.8683358	0.13010771	6.67397628	2.49E-11	7.75E-11	Up	FAM156A
MS4A4E	5.22811868	-1.54444186	0.23141528	-6.67389737	2.49E-11	7.75E-11	Down	MS4A4E
SLC13A2	437.478702	-2.11177075	0.31645967	-6.67311167	2.50E-11	7.79E-11	Down	SLC13A2
PDE3B	818.992917	-0.73952936	0.11084119	-6.67197224	2.52E-11	7.85E-11	Down	PDE3B
GMFB	2402.13856	-0.69517315	0.10419433	-6.67189045	2.53E-11	7.85E-11	Down	GMFB
PTEN	2506.9253	-0.61360179	0.09198091	-6.67096924	2.54E-11	7.90E-11	Down	PTEN
FAIM	325.93822	0.58208924	0.0872672	6.6701951	2.55E-11	7.94E-11	Up	FAIM
C3orf80	76.5598042	-1.22552273	0.18379217	-6.66798121	2.59E-11	8.06E-11	Down	C3orf80
EPHX1	2038.68634	-0.78315477	0.1174624	-6.66728074	2.61E-11	8.09E-11	Down	EPHX1
CDK6	2313.4475	0.73388471	0.11008718	6.66639558	2.62E-11	8.14E-11	Up	CDK6
GUCD1	4198.1629	-0.50000504	0.0750125	-6.66562268	2.64E-11	8.18E-11	Down	GUCD1
MOCOS	618.770025	0.7852875	0.11781788	6.66526608	2.64E-11	8.20E-11	Up	MOCOS
LRRC73	41.6943184	1.27357905	0.19110297	6.66436022	2.66E-11	8.25E-11	Up	LRRC73
AP5Z1	1912.49886	0.69193517	0.10384746	6.66299558	2.68E-11	8.32E-11	Up	AP5Z1
LYZL4	1.24019723	2.98789963	0.44852262	6.66164763	2.71E-11	8.40E-11	Up	LYZL4
SPON1	1729.69774	-1.50068534	0.22530008	-6.66082925	2.72E-11	8.44E-11	Down	SPON1
PSMB5	4055.59241	0.5495384	0.08250799	6.66042635	2.73E-11	8.46E-11	Up	PSMB5
TGM1	28.5162603	0.96387442	0.14478777	6.6571537	2.79E-11	8.65E-11	Up	TGM1
HLA-DOB	60.3843706	-1.16358958	0.17479069	-6.65704551	2.79E-11	8.66E-11	Down	HLA-DOB
ZNF831	32.8375543	-1.38108314	0.20746514	-6.65694062	2.80E-11	8.66E-11	Down	ZNF831
TAS2R5	18.787441	1.04443049	0.15690453	6.65647142	2.80E-11	8.69E-11	Up	TAS2R5

ANO8	415.003106	0.72611248	0.1091201	6.65425039	2.85E-11	8.82E-11	Up	ANO8
PRKAB2	852.908425	-0.61370236	0.09223132	-6.65394774	2.85E-11	8.83E-11	Down	PRKAB2
KIF1B	2108.58107	-0.576234	0.08660106	-6.65389067	2.85E-11	8.84E-11	Down	KIF1B
TMEM81	56.5417886	0.57965094	0.08712074	6.65342109	2.86E-11	8.86E-11	Up	TMEM81
CTTNBP2	413.985207	1.31051367	0.19696935	6.65338887	2.86E-11	8.86E-11	Up	CTTNBP2
TIA1	2099.30761	0.59159034	0.08892964	6.65234179	2.88E-11	8.93E-11	Up	TIA1
C1QB	3198.03462	-1.17185398	0.17622475	-6.64976953	2.94E-11	9.08E-11	Down	C1QB
FBXL19	1131.56156	0.60849697	0.0915372	6.64753714	2.98E-11	9.22E-11	Up	FBXL19
SLC27A2	1064.9158	-0.73836618	0.11112578	-6.64441811	3.04E-11	9.41E-11	Down	SLC27A2
CHRNG	5.5637049	1.90917641	0.28741474	6.64258358	3.08E-11	9.53E-11	Up	CHRNG
DUSP21	0.46797189	-4.02934979	0.60660958	-6.64241039	3.09E-11	9.53E-11	Down	DUSP21
P2RY13	93.9989147	-1.31022966	0.19729679	-6.64090725	3.12E-11	9.63E-11	Down	P2RY13
DNAJB1	7453.76234	0.66604906	0.10029818	6.64068945	3.12E-11	9.64E-11	Up	DNAJB1
VAV1	306.340227	-0.94378681	0.1421705	-6.63841538	3.17E-11	9.79E-11	Down	VAV1
RPGR	386.427411	0.65798653	0.09912361	6.63804059	3.18E-11	9.81E-11	Up	RPGR
PDZD3	301.312866	-1.22342677	0.18434261	-6.63670076	3.21E-11	9.90E-11	Down	PDZD3
RAET1G	15.5719499	1.20386076	0.18139461	6.63669521	3.21E-11	9.90E-11	Up	RAET1G
DPY19L3	833.144721	0.65786031	0.09914019	6.63565727	3.23E-11	9.97E-11	Up	DPY19L3
DDIT3	631.798207	0.75299899	0.11351252	6.63362075	3.28E-11	1.01E-10	Up	DDIT3
FGD4	966.25085	-0.81131141	0.12233939	-6.63164513	3.32E-11	1.02E-10	Down	FGD4
LPCAT2	1488.72528	0.81799646	0.12335603	6.63118334	3.33E-11	1.03E-10	Up	LPCAT2
ASAP1	1245.50339	0.74542671	0.11242184	6.6306217	3.34E-11	1.03E-10	Up	ASAP1
CMTM4	4443.8092	-0.55105078	0.08310703	-6.63061565	3.34E-11	1.03E-10	Down	CMTM4
ELF4	2509.58976	-0.57018902	0.08600253	-6.62990977	3.36E-11	1.03E-10	Down	ELF4
LIX1	5.20931773	-2.43286951	0.36695895	-6.62981382	3.36E-11	1.04E-10	Down	LIX1
PIGW	411.567463	0.59932132	0.09040414	6.62935679	3.37E-11	1.04E-10	Up	PIGW
SDK1	222.826819	-1.24067937	0.18717019	-6.62861625	3.39E-11	1.04E-10	Down	SDK1
PGD	5166.32791	0.58988049	0.08899858	6.62797662	3.40E-11	1.05E-10	Up	PGD
SEMA5B	53.8240635	0.89702858	0.13534117	6.62790604	3.40E-11	1.05E-10	Up	SEMA5B
POLR3GL	575.105903	-0.54902946	0.08285073	-6.62673031	3.43E-11	1.06E-10	Down	POLR3GL
GPR31	1.87911637	-1.57219311	0.2372522	-6.6266746	3.43E-11	1.06E-10	Down	GPR31
NPY4R	13.4206728	-1.47385386	0.22248858	-6.62440237	3.49E-11	1.07E-10	Down	NPY4R
TMEM150A	561.508833	0.61218067	0.09242095	6.62383003	3.50E-11	1.08E-10	Up	TMEM150A
PTPRN	68.0321587	-1.57820569	0.23826819	-6.62365243	3.50E-11	1.08E-10	Down	PTPRN
C1QC	3194.12023	-1.16122963	0.17531531	-6.62366342	3.50E-11	1.08E-10	Down	C1QC
NIPSNAP3A	1125.36972	-0.65348385	0.09866586	-6.62320109	3.52E-11	1.08E-10	Down	NIPSNAP3A
RBP5	109.372061	-0.98968994	0.14943165	-6.62302775	3.52E-11	1.08E-10	Down	RBP5
ARHGAP8	131.997757	0.88835179	0.13413835	6.62265355	3.53E-11	1.08E-10	Up	ARHGAP8
PTPRT	12.0065431	-1.64827249	0.24891815	-6.621745	3.55E-11	1.09E-10	Down	PTPRT
RARRES2	1734.67521	-1.06208126	0.16044231	-6.61970797	3.60E-11	1.11E-10	Down	RARRES2
LYG1	26.8787135	1.17721378	0.17786933	6.61841907	3.63E-11	1.11E-10	Up	LYG1
CCDC88B	2046.45372	1.30760415	0.19757883	6.61813905	3.64E-11	1.12E-10	Up	CCDC88B
SFI1	970.865514	0.54995115	0.08311628	6.61664746	3.67E-11	1.13E-10	Up	SFI1
EZR	16041.3884	-0.56170036	0.08494103	-6.61282708	3.77E-11	1.16E-10	Down	EZR
TTC12	476.009546	0.50499391	0.07636758	6.61267372	3.77E-11	1.16E-10	Up	TTC12
SMAD4	1607.67061	-0.59765388	0.09039369	-6.611677	3.80E-11	1.17E-10	Down	SMAD4
LRRK1	1425.11202	-0.50044397	0.07569233	-6.61155503	3.80E-11	1.17E-10	Down	LRRK1
AADACL2	2.27826982	-3.7839362	0.57234715	-6.6112607	3.81E-11	1.17E-10	Down	AADACL2
SV2A	107.949936	-0.99245828	0.15014515	-6.60999237	3.84E-11	1.18E-10	Down	SV2A
U2SURP	3232.73169	0.64276179	0.09726074	6.60864623	3.88E-11	1.19E-10	Up	U2SURP
GET4	235.665327	0.66582273	0.1007536	6.60842592	3.88E-11	1.19E-10	Up	GET4
SERTAD2	947.365107	-0.53894424	0.08155594	-6.60827665	3.89E-11	1.19E-10	Down	SERTAD2
CSRNP1	1219.04479	-0.69461655	0.1051146	-6.60818316	3.89E-11	1.19E-10	Down	CSRNP1
DISP1	183.211157	-0.66307458	0.10034452	-6.60798025	3.90E-11	1.19E-10	Down	DISP1
RHOD	587.766869	1.13192029	0.17130255	6.6077257	3.90E-11	1.20E-10	Up	RHOD
KRT34	1.97946633	3.72470325	0.56374616	6.6070574	3.92E-11	1.20E-10	Up	KRT34
MITF	240.150287	-1.18041277	0.17868411	-6.606143	3.94E-11	1.21E-10	Down	MITF
CARM1	3188.61685	0.58165556	0.08806125	6.60512523	3.97E-11	1.22E-10	Up	CARM1

RPL38	13896.371	0.59471617	0.0900707	6.60277048	4.04E-11	1.23E-10	Up	RPL38
NHSL2	23.7086056	-1.52392983	0.23083109	-6.60192625	4.06E-11	1.24E-10	Down	NHSL2
CALM3	11076.2293	-0.51373036	0.07781576	-6.60188025	4.06E-11	1.24E-10	Down	CALM3
JAKMIP3	13.3658265	-1.23835492	0.1875979	-6.60111294	4.08E-11	1.25E-10	Down	JAKMIP3
SGIP1	133.309972	1.17866735	0.178561	6.60092274	4.09E-11	1.25E-10	Up	SGIP1
KRAS	2033.96812	-0.64549379	0.09779206	-6.6006768	4.09E-11	1.25E-10	Down	KRAS
CMAS	2505.97638	-0.51219606	0.07764251	-6.59685117	4.20E-11	1.28E-10	Down	CMAS
PDZK1IP1	4176.86338	1.11731505	0.16939681	6.59584472	4.23E-11	1.29E-10	Up	PDZK1IP1
HTRA3	1694.45492	1.16729473	0.17702299	6.5940291	4.28E-11	1.31E-10	Up	HTRA3
SERINC2	9163.27937	-0.87472546	0.1326606	-6.59370949	4.29E-11	1.31E-10	Down	SERINC2
ECEL1	31.4208153	1.93041608	0.29291469	6.59036969	4.39E-11	1.34E-10	Up	ECEL1
OMD	49.0727298	-2.33019119	0.35368427	-6.58833707	4.45E-11	1.36E-10	Down	OMD
TAF4B	349.719853	0.63263297	0.0960249	6.58821763	4.45E-11	1.36E-10	Up	TAF4B
FAM118A	1083.39273	0.67541924	0.10253704	6.58707585	4.49E-11	1.37E-10	Up	FAM118A
UGT1A9	1.78377226	-2.11032335	0.32038127	-6.58691241	4.49E-11	1.37E-10	Down	UGT1A9
ACAP3	1477.63463	0.76946844	0.11683441	6.58597452	4.52E-11	1.38E-10	Up	ACAP3
GPX1	7666.55474	0.77238675	0.11727893	6.58589545	4.52E-11	1.38E-10	Up	GPX1
LTB4R2	52.9436061	0.98296183	0.14925442	6.5858139	4.52E-11	1.38E-10	Up	LTB4R2
LENG8	5577.00568	0.81577374	0.12389119	6.58459867	4.56E-11	1.39E-10	Up	LENG8
POLR3F	438.517131	0.60652771	0.09211809	6.58424133	4.57E-11	1.39E-10	Up	POLR3F
KL	57.3233878	-1.48576801	0.22574272	-6.58168734	4.65E-11	1.42E-10	Down	KL
HECTD2	135.81291	-1.02785894	0.15620098	-6.58036177	4.69E-11	1.43E-10	Down	HECTD2
GABRG2	4.92474732	-3.08953887	0.46952852	-6.58008784	4.70E-11	1.43E-10	Down	GABRG2
MAPRE2	1399.96725	-0.55751965	0.08473275	-6.57974179	4.71E-11	1.43E-10	Down	MAPRE2
ANKRD36B	65.2529509	1.30844191	0.1988655	6.57953195	4.72E-11	1.44E-10	Up	ANKRD36B
BANP	541.153617	0.5605345	0.08519426	6.57948659	4.72E-11	1.44E-10	Up	BANP
ANAPC11	3524.3196	0.99857246	0.15185868	6.57566912	4.84E-11	1.47E-10	Up	ANAPC11
CPEB4	778.764062	-0.80279037	0.12209282	-6.5752466	4.86E-11	1.48E-10	Down	CPEB4
CCDC50	1730.82886	-0.52220132	0.0794212	-6.57508736	4.86E-11	1.48E-10	Down	CCDC50
ADAM11	16.5507281	-1.34434718	0.2045044	-6.57368332	4.91E-11	1.49E-10	Down	ADAM11
LDHB	12772.1087	0.90752343	0.13808091	6.57240316	4.95E-11	1.50E-10	Up	LDHB
STX19	259.539287	-0.94875984	0.14435483	-6.57241507	4.95E-11	1.50E-10	Down	STX19
ZSCAN1	1.25726998	-1.42573283	0.21698125	-6.57076517	5.01E-11	1.52E-10	Down	ZSCAN1
JDP2	738.333621	-0.55842693	0.08500625	-6.5692454	5.06E-11	1.54E-10	Down	JDP2
CCDC73	11.8907557	1.09327635	0.16646809	6.56748289	5.12E-11	1.55E-10	Up	CCDC73
SAA1	363.7402	1.9282348	0.29362122	6.56708261	5.13E-11	1.56E-10	Up	SAA1
GPR17	22.0803047	-1.53177489	0.23326623	-6.56663785	5.15E-11	1.56E-10	Down	GPR17
KHK	678.118543	0.85932404	0.13086497	6.56649396	5.15E-11	1.56E-10	Up	KHK
ARPC5L	2036.58507	0.50322898	0.07664623	6.56560644	5.18E-11	1.57E-10	Up	ARPC5L
KCNS2	4.69793763	-1.93329937	0.29450603	-6.56454941	5.22E-11	1.58E-10	Down	KCNS2
FAM78A	194.0694	-0.88806437	0.13528354	-6.56446743	5.22E-11	1.58E-10	Down	FAM78A
MYL12B	13936.2241	-0.54655112	0.08327756	-6.56300591	5.27E-11	1.60E-10	Down	MYL12B
PHACTR3	101.194089	1.67020602	0.25453487	6.56179658	5.32E-11	1.61E-10	Up	PHACTR3
SLC14A2	21.4790606	-2.47795141	0.37765859	-6.56135323	5.33E-11	1.62E-10	Down	SLC14A2
ZNF429	144.040043	-0.75601823	0.11528055	-6.55807286	5.45E-11	1.65E-10	Down	ZNF429
TMEM254	728.420244	-0.60084731	0.09162358	-6.55778031	5.46E-11	1.66E-10	Down	TMEM254
DGKA	1061.26882	-0.81372619	0.12410246	-6.55689016	5.49E-11	1.66E-10	Down	DGKA
AVEN	581.525334	0.50379127	0.07685464	6.55511836	5.56E-11	1.68E-10	Up	AVEN
MCM5	3016.47921	0.72755618	0.11099157	6.55505821	5.56E-11	1.68E-10	Up	MCM5
KLF8	78.3525538	-1.09646038	0.16727755	-6.5547371	5.57E-11	1.69E-10	Down	KLF8
PGPEP1	2049.00204	-0.51084179	0.07796471	-6.55221804	5.67E-11	1.72E-10	Down	PGPEP1
LY96	80.0612959	-1.13024192	0.17250375	-6.55198478	5.68E-11	1.72E-10	Down	LY96
SPRN	128.779285	0.70918556	0.10824569	6.5516283	5.69E-11	1.72E-10	Up	SPRN
TBX5	4.41633136	4.05594724	0.61918408	6.55047081	5.74E-11	1.74E-10	Up	TBX5
FOXC1	216.348919	1.35297308	0.20656616	6.54982939	5.76E-11	1.74E-10	Up	FOXC1
UBALD1	988.362128	0.69737765	0.10649168	6.54865874	5.81E-11	1.76E-10	Up	UBALD1
BAIAP2	1347.22897	0.79046271	0.12073729	6.54696405	5.87E-11	1.78E-10	Up	BAIAP2
EPGN	2.52712514	2.96097672	0.45228338	6.54672895	5.88E-11	1.78E-10	Up	EPGN

NEK3	1336.78554	0.78916264	0.12054998	6.54635255	5.90E-11	1.78E-10	Up	NEK3
CYP4Z1	3.93881754	2.63061585	0.40185541	6.54617497	5.90E-11	1.78E-10	Up	CYP4Z1
ACVR2A	503.078656	-0.56414235	0.08618821	-6.54546982	5.93E-11	1.79E-10	Down	ACVR2A
KRT4	9.42850944	2.74374672	0.41919879	6.54521625	5.94E-11	1.80E-10	Up	KRT4
RPS5	41175.3282	0.93911555	0.14352403	6.54326345	6.02E-11	1.82E-10	Up	RPS5
ROR2	351.031334	-1.262341	0.19292913	-6.54302941	6.03E-11	1.82E-10	Down	ROR2
CMTM1	39.0958594	0.68863748	0.10528376	6.54077611	6.12E-11	1.85E-10	Up	CMTM1
OBP2A	3.12861356	2.7718968	0.42381072	6.54041214	6.13E-11	1.85E-10	Up	OBP2A
SYT5	16.9096065	-1.63043405	0.24943345	-6.53654942	6.30E-11	1.90E-10	Down	SYT5
RHOH	161.906961	-1.11746414	0.17097741	-6.53574149	6.33E-11	1.91E-10	Down	RHOH
DHDH	47.8735473	1.26543037	0.19363389	6.53517007	6.35E-11	1.92E-10	Up	DHDH
CD34	1032.01205	-0.76192375	0.1166299	-6.53283359	6.45E-11	1.95E-10	Down	CD34
ZHX1	1049.05561	-0.58508471	0.08957607	-6.53170813	6.50E-11	1.96E-10	Down	ZHX1
MCTP2	1073.89443	-0.69450134	0.10637682	-6.52869052	6.63E-11	2.00E-10	Down	MCTP2
GNG13	14.325949	-2.09154673	0.32038807	-6.52816673	6.66E-11	2.01E-10	Down	GNG13
APLF	137.694569	0.92689316	0.1420391	6.52561973	6.77E-11	2.04E-10	Up	APLF
SYMPK	4131.27269	0.50632116	0.07760455	6.52437461	6.83E-11	2.06E-10	Up	SYMPK
MAP2	69.4064939	-1.43564175	0.22004545	-6.52429638	6.83E-11	2.06E-10	Down	MAP2
DPP8	1179.55918	-0.55468452	0.0850447	-6.5222699	6.93E-11	2.09E-10	Down	DPP8
OTUD1	593.133457	-0.57760095	0.0885682	-6.52153841	6.96E-11	2.10E-10	Down	OTUD1
SLC12A6	718.367513	-0.55982029	0.08586864	-6.51949662	7.05E-11	2.12E-10	Down	SLC12A6
DENND4B	1664.4329	0.50484156	0.07745146	6.51816734	7.12E-11	2.14E-10	Up	DENND4B
NDP	27.288219	1.85080495	0.2839691	6.51762807	7.14E-11	2.15E-10	Up	NDP
PRCD	15.9939132	-0.79817675	0.12252381	-6.51446228	7.30E-11	2.20E-10	Down	PRCD
TTC33	357.757599	-0.61459619	0.09434445	-6.51438619	7.30E-11	2.20E-10	Down	TTC33
KLF16	1408.84047	0.61709282	0.09473467	6.51390668	7.32E-11	2.20E-10	Up	KLF16
PRR4	30.066205	1.28961668	0.19809586	6.51006386	7.51E-11	2.26E-10	Up	PRR4
FAM83E	1872.30007	-0.84139976	0.12925472	-6.50962492	7.53E-11	2.27E-10	Down	FAM83E
IL36B	3.79368523	3.78616177	0.58177448	6.50795439	7.62E-11	2.29E-10	Up	IL36B
ACER3	1050.97135	-0.56767352	0.08723019	-6.50776457	7.63E-11	2.29E-10	Down	ACER3
C21orf58	241.888596	0.71738876	0.11026212	6.50621247	7.71E-11	2.32E-10	Up	C21orf58
IQCJ-SCHIP1	40.3676943	-1.056756	0.16244557	-6.50529295	7.75E-11	2.33E-10	Down	IQCJ-SCHIP1
MUC21	1.73836259	3.5444333	0.54501074	6.5034192	7.85E-11	2.36E-10	Up	MUC21
PCDHGC3	323.467449	-1.11735538	0.17184017	-6.50229461	7.91E-11	2.37E-10	Down	PCDHGC3
TPD52L1	1094.3347	0.88338853	0.13588474	6.50101351	7.98E-11	2.39E-10	Up	TPD52L1
JRK	752.7594	0.60322451	0.09283014	6.49815328	8.13E-11	2.44E-10	Up	JRK
SPRR2E	4.51551769	4.85402077	0.7471221	6.49695777	8.20E-11	2.46E-10	Up	SPRR2E
NDUFS4	1194.71558	-0.52296912	0.0805002	-6.4964947	8.22E-11	2.47E-10	Down	NDUFS4
GDF11	448.576258	0.71054464	0.10943336	6.49294376	8.42E-11	2.52E-10	Up	GDF11
ZNF117	444.730466	1.06825366	0.1645552	6.49176482	8.48E-11	2.54E-10	Up	ZNF117
NAALAD2	21.7683823	-1.18074034	0.18195382	-6.48923073	8.63E-11	2.59E-10	Down	NAALAD2
CDH17	20595.6745	-0.75321013	0.11607725	-6.48886967	8.65E-11	2.59E-10	Down	CDH17
ASPH	7527.18072	0.67603783	0.10420508	6.48757081	8.72E-11	2.61E-10	Up	ASPH
IL18	870.494716	-0.84391524	0.13008857	-6.48723609	8.74E-11	2.62E-10	Down	IL18
SLC25A35	199.702874	-0.63921717	0.09855625	-6.48581064	8.83E-11	2.64E-10	Down	SLC25A35
PHLDB1	1642.7008	-0.66801885	0.103051	-6.4824101	9.03E-11	2.70E-10	Down	PHLDB1
GBP2	2370.2676	-0.85027644	0.13119706	-6.48091084	9.12E-11	2.73E-10	Down	GBP2
OAS3	3565.17592	0.83004953	0.12808127	6.48064729	9.13E-11	2.73E-10	Up	OAS3
ASIC4	5.28116714	1.81946682	0.28076184	6.4804634	9.14E-11	2.74E-10	Up	ASIC4
MPHOSPH10	1254.03407	0.52281537	0.08068001	6.48011007	9.17E-11	2.74E-10	Up	MPHOSPH10
AP4S1	193.556335	-0.64847573	0.10007656	-6.4797963	9.18E-11	2.75E-10	Down	AP4S1
CIART	47.4068007	-1.15544782	0.17832992	-6.4792707	9.22E-11	2.76E-10	Down	CIART
CYP2U1	258.36798	-0.70105347	0.1082185	-6.47812973	9.29E-11	2.78E-10	Down	CYP2U1
EIF5B	4744.70028	0.59146439	0.09131661	6.47707358	9.35E-11	2.80E-10	Up	EIF5B
ZNF600	518.27147	0.52384323	0.08087875	6.47689564	9.36E-11	2.80E-10	Up	ZNF600
FBXW12	4.7447437	1.5990181	0.24692547	6.47571137	9.44E-11	2.82E-10	Up	FBXW12
DDX49	2068.12432	0.58460778	0.09028755	6.47495482	9.48E-11	2.83E-10	Up	DDX49
AKT3	458.766331	-1.09276649	0.16878632	-6.47425978	9.53E-11	2.85E-10	Down	AKT3

PCDH11Y	0.93809251	-3.56568561	0.55075326	-6.47419788	9.53E-11	2.85E-10	Down	PCDH11Y
MED27	589.180817	0.52176874	0.08060773	6.47293659	9.61E-11	2.87E-10	Up	MED27
MAP7D1	2232.7154	-0.55198315	0.0852959	-6.47139158	9.71E-11	2.90E-10	Down	MAP7D1
SH2B3	1055.79834	-0.62222597	0.09615099	-6.47134233	9.71E-11	2.90E-10	Down	SH2B3
CDC42EP1	4768.85231	0.80214539	0.12398427	6.46973521	9.82E-11	2.93E-10	Up	CDC42EP1
CARD16	256.700912	-0.79418374	0.12277732	-6.46848892	9.90E-11	2.95E-10	Down	CARD16
TSLP	18.6938879	-1.12616337	0.17410685	-6.46823117	9.92E-11	2.96E-10	Down	TSLP
WEE1	1603.00851	0.59696555	0.09229452	6.46804968	9.93E-11	2.96E-10	Up	WEE1
NBPF14	95.5813184	-0.59986802	0.09274669	-6.46781065	9.94E-11	2.97E-10	Down	NBPF14
QPCT	1197.17583	1.2212278	0.18892393	6.4641246	1.02E-10	3.04E-10	Up	QPCT
PIDD1	736.053367	0.73056202	0.11302498	6.46372147	1.02E-10	3.05E-10	Up	PIDD1
UFSP1	90.2502258	0.86437235	0.13377139	6.46156359	1.04E-10	3.09E-10	Up	UFSP1
PTGES2	3704.38373	0.75571637	0.11696367	6.46112076	1.04E-10	3.10E-10	Up	PTGES2
AZIN1	6944.23949	0.5335836	0.08262923	6.45756457	1.06E-10	3.17E-10	Up	AZIN1
FTCDNL1	95.0807686	0.82176978	0.12727114	6.45684331	1.07E-10	3.18E-10	Up	FTCDNL1
TRIM50	2.80559639	-1.60858279	0.24913809	-6.45659107	1.07E-10	3.19E-10	Down	TRIM50
RCN2	2020.65526	0.52361611	0.08110185	6.45627811	1.07E-10	3.19E-10	Up	RCN2
TAF2	1179.0762	0.52510778	0.081336	6.45603146	1.07E-10	3.20E-10	Up	TAF2
SMC4	1690.67568	0.7007568	0.10854226	6.45607329	1.07E-10	3.20E-10	Up	SMC4
NLN	1375.33632	0.51135833	0.07920616	6.45604272	1.07E-10	3.20E-10	Up	NLN
NUDT12	584.695479	-0.97065947	0.15038063	-6.45468415	1.08E-10	3.23E-10	Down	NUDT12
STMN3	1310.56533	1.36507733	0.21154217	6.45297969	1.10E-10	3.26E-10	Up	STMN3
PHLDB2	428.989836	-1.20758248	0.18715805	-6.45220715	1.10E-10	3.28E-10	Down	PHLDB2
CFAP43	22.5232909	1.17640341	0.18236648	6.45076569	1.11E-10	3.31E-10	Up	CFAP43
FDXR	899.744281	0.83719427	0.12978684	6.45053268	1.11E-10	3.31E-10	Up	FDXR
SMIM3	1170.10956	0.74424632	0.11540067	6.4492375	1.12E-10	3.34E-10	Up	SMIM3
MFSD2B	24.7869774	0.92789401	0.14392409	6.44710714	1.14E-10	3.39E-10	Up	MFSD2B
ANKRD1	5.04594423	2.25029766	0.34905528	6.44682321	1.14E-10	3.39E-10	Up	ANKRD1
CDK15	8.63995493	-1.58587577	0.24607238	-6.44475336	1.16E-10	3.44E-10	Down	CDK15
IGSF5	4.61260554	-1.52282051	0.23635917	-6.44282393	1.17E-10	3.48E-10	Down	IGSF5
JSRP1	163.354552	1.43632954	0.22300033	6.44093003	1.19E-10	3.52E-10	Up	JSRP1
LCN1	1.81915305	2.57433402	0.39981632	6.43879169	1.20E-10	3.57E-10	Up	LCN1
HHAT	174.448935	-0.7133078	0.11084385	-6.43524931	1.23E-10	3.66E-10	Down	HHAT
TRIM34	13.8793531	-0.73034897	0.11353663	-6.43271659	1.25E-10	3.72E-10	Down	TRIM34
C4BPB	314.534448	1.24645219	0.19381771	6.43105415	1.27E-10	3.76E-10	Up	C4BPB
PEAK1	1502.69979	-0.62340521	0.09694184	-6.43071367	1.27E-10	3.76E-10	Down	PEAK1
TMEM132D	6.98623149	-1.69746253	0.26403568	-6.42891332	1.29E-10	3.81E-10	Down	TMEM132D
CHMP4C	1441.7287	0.66655006	0.10368381	6.42868023	1.29E-10	3.81E-10	Up	CHMP4C
STOX1	197.150257	1.09370424	0.17016497	6.42731731	1.30E-10	3.85E-10	Up	STOX1
C8orf33	3716.00806	0.75653249	0.11771245	6.42695402	1.30E-10	3.86E-10	Up	C8orf33
C15orf65	46.9837236	-0.77784549	0.12103992	-6.42635502	1.31E-10	3.87E-10	Down	C15orf65
IL10	17.9037783	-1.24595835	0.19388631	-6.42623163	1.31E-10	3.87E-10	Down	IL10
MAN2B1	2504.23159	-0.5385637	0.08384586	-6.42325948	1.33E-10	3.95E-10	Down	MAN2B1
SERPINB4	3.05665893	4.35155543	0.67747532	6.42319399	1.33E-10	3.95E-10	Up	SERPINB4
NSMCE1	2120.44761	0.60787223	0.0946516	6.42220779	1.34E-10	3.97E-10	Up	NSMCE1
CLDN6	7.10530129	2.19453217	0.34181452	6.42024274	1.36E-10	4.02E-10	Up	CLDN6
NXPE2	105.457548	-1.54651273	0.2408996	-6.41973973	1.37E-10	4.03E-10	Down	NXPE2
CYCS	7991.50703	-0.64904108	0.10110301	-6.41960161	1.37E-10	4.04E-10	Down	CYCS
FAM217B	720.052897	0.75647296	0.11788061	6.4172807	1.39E-10	4.10E-10	Up	FAM217B
ABCC3	5736.3168	-0.72242833	0.11259204	-6.4163355	1.40E-10	4.12E-10	Down	ABCC3
FEZF2	2.43878773	3.85611703	0.60102074	6.41594668	1.40E-10	4.13E-10	Up	FEZF2
TIGD3	32.6043702	0.88791741	0.13845557	6.41301313	1.43E-10	4.21E-10	Up	TIGD3
TRPC4	25.2014318	-1.0323609	0.16098188	-6.41290115	1.43E-10	4.22E-10	Down	TRPC4
KLHL5	511.638719	-0.92796712	0.14470962	-6.41261524	1.43E-10	4.22E-10	Down	KLHL5
RRAS2	632.818771	-0.7590718	0.11838831	-6.41171235	1.44E-10	4.25E-10	Down	RRAS2
ALPK3	707.484342	1.55990975	0.24329175	6.4116837	1.44E-10	4.25E-10	Up	ALPK3
SCARF2	360.019731	1.15521472	0.18020168	6.41067657	1.45E-10	4.27E-10	Up	SCARF2
CCDC71L	1168.53328	0.5584013	0.08710587	6.41060477	1.45E-10	4.28E-10	Up	CCDC71L

TRIM61	2.979705	-1.32372341	0.206565	-6.40826586	1.47E-10	4.34E-10	Down	TRIM61
ULK3	1349.87407	-0.5245221	0.0818808	-6.40592269	1.49E-10	4.41E-10	Down	ULK3
TPRA1	1416.81913	0.53576195	0.08364336	6.40531374	1.50E-10	4.42E-10	Up	TPRA1
FMO3	37.9091565	1.06077473	0.16561369	6.40511488	1.50E-10	4.43E-10	Up	FMO3
SSX2IP	1045.05062	0.59211666	0.09245063	6.40467919	1.51E-10	4.44E-10	Up	SSX2IP
KIF3A	353.173786	0.62272143	0.09731572	6.39898052	1.56E-10	4.61E-10	Up	KIF3A
GRAP2	91.6420574	-0.97987769	0.15313868	-6.39862953	1.57E-10	4.62E-10	Down	GRAP2
ZNF844	381.200769	-0.90976231	0.1421852	-6.39843199	1.57E-10	4.62E-10	Down	ZNF844
LTB	251.750913	-1.03000364	0.16097733	-6.39843913	1.57E-10	4.62E-10	Down	LTB
INSIG1	3387.22803	0.71446798	0.11168863	6.39696269	1.58E-10	4.67E-10	Up	INSIG1
TBXAS1	1122.04174	0.94681003	0.14802494	6.39628727	1.59E-10	4.69E-10	Up	TBXAS1
CLEC11A	412.862236	0.96407359	0.15074359	6.39545347	1.60E-10	4.71E-10	Up	CLEC11A
GPRIN1	278.450127	0.75517415	0.11808979	6.3949148	1.61E-10	4.73E-10	Up	GPRIN1
BLNK	446.447241	-0.81305631	0.12718275	-6.39281887	1.63E-10	4.79E-10	Down	BLNK
POLR2J3	83.0959363	0.78337421	0.12255489	6.39202715	1.64E-10	4.81E-10	Up	POLR2J3
PTDSS2	1233.38589	0.70909881	0.11098937	6.38888939	1.67E-10	4.91E-10	Up	PTDSS2
ABHD4	894.387832	-0.59835628	0.09366899	-6.38798712	1.68E-10	4.94E-10	Down	ABHD4
RLN2	17.8228695	1.40699808	0.22027495	6.38746297	1.69E-10	4.96E-10	Up	RLN2
ARHGAP21	2091.31097	-0.53066961	0.08310446	-6.38557312	1.71E-10	5.02E-10	Down	ARHGAP21
RBAK	672.165849	0.58058297	0.09093953	6.38427512	1.72E-10	5.06E-10	Up	RBAK
DMP1	1.27962344	3.10932536	0.48705023	6.38399321	1.73E-10	5.07E-10	Up	DMP1
UBA6	1679.92746	0.6084752	0.09532839	6.38293773	1.74E-10	5.10E-10	Up	UBA6
SASS6	283.423197	0.6590331	0.10326545	6.38193201	1.75E-10	5.14E-10	Up	SASS6
POLR2I	1061.14019	0.88943321	0.13938467	6.38114065	1.76E-10	5.16E-10	Up	POLR2I
USP53	1753.87625	-0.73889828	0.11579521	-6.38107828	1.76E-10	5.16E-10	Down	USP53
IVL	3.45366012	4.65358025	0.72930599	6.38083368	1.76E-10	5.17E-10	Up	IVL
HS2ST1	1504.55822	0.53610888	0.08404682	6.37869335	1.79E-10	5.24E-10	Up	HS2ST1
HS3ST1	500.42791	0.74454306	0.11676183	6.3765965	1.81E-10	5.31E-10	Up	HS3ST1
SMPX	36.0823715	-2.46491568	0.38656341	-6.37648469	1.81E-10	5.31E-10	Down	SMPX
HELB	315.274434	0.74379434	0.11665883	6.3758086	1.82E-10	5.34E-10	Up	HELB
HOXC13	4.00931485	4.29487137	0.67367422	6.37529419	1.83E-10	5.35E-10	Up	HOXC13
ZNF503	953.7209	0.91297908	0.1432375	6.37388295	1.84E-10	5.40E-10	Up	ZNF503
PCGF5	2431.66813	-0.50565842	0.07933361	-6.37382316	1.84E-10	5.40E-10	Down	PCGF5
FOXL2	3.32337865	3.43915714	0.53959598	6.37357811	1.85E-10	5.41E-10	Up	FOXL2
CYGB	631.812998	-0.77940211	0.12232889	-6.37136599	1.87E-10	5.49E-10	Down	CYGB
C19orf33	1648.06949	-0.99715353	0.15653267	-6.37025824	1.89E-10	5.53E-10	Down	C19orf33
KCNS3	322.348778	-1.01238813	0.15894012	-6.36961994	1.89E-10	5.55E-10	Down	KCNS3
CAMP	2.95929908	-1.61870507	0.25413446	-6.36948269	1.90E-10	5.55E-10	Down	CAMP
PMM1	1074.99146	-0.67781575	0.10642921	-6.36870043	1.91E-10	5.58E-10	Down	PMM1
FOSB	1441.08555	-1.64483693	0.25828217	-6.36837189	1.91E-10	5.59E-10	Down	FOSB
DYNLRB1	4662.73315	0.63318556	0.09944443	6.36722991	1.92E-10	5.63E-10	Up	DYNLRB1
CHRND	2.38334605	3.51817411	0.55256058	6.36703777	1.93E-10	5.64E-10	Up	CHRND
SPNS1	125.729022	0.6054936	0.09512846	6.36501013	1.95E-10	5.71E-10	Up	SPNS1
RPL35A	19664.4655	0.54867825	0.08620966	6.36446387	1.96E-10	5.73E-10	Up	RPL35A
NR1D1	914.67611	0.73769583	0.11592183	6.36373524	1.97E-10	5.76E-10	Up	NR1D1
TMEM63B	2248.3529	-0.5958454	0.0936451	-6.36280385	1.98E-10	5.79E-10	Down	TMEM63B
MAP1LC3B2	36.484509	-0.71066717	0.11169318	-6.36267268	1.98E-10	5.79E-10	Down	MAP1LC3B2
MUTYH	695.816874	0.59962041	0.09425052	6.36198527	1.99E-10	5.82E-10	Up	MUTYH
IL17F	4.58923994	2.44285782	0.38400752	6.36148426	2.00E-10	5.84E-10	Up	IL17F
PAX5	123.454033	-1.94723127	0.30609924	-6.36143787	2.00E-10	5.84E-10	Down	PAX5
GAK	5236.47358	-0.50663671	0.07964553	-6.36114446	2.00E-10	5.85E-10	Down	GAK
C6orf141	185.80508	1.1191132	0.17595308	6.36029338	2.01E-10	5.88E-10	Up	C6orf141
MFSD6L	120.254864	-1.19942874	0.1886	-6.3596433	2.02E-10	5.90E-10	Down	MFSD6L
ANKRD44	272.497139	-0.876796	0.13788531	-6.35887918	2.03E-10	5.93E-10	Down	ANKRD44
PRSS12	519.6705	-0.87460946	0.13761585	-6.35544136	2.08E-10	6.07E-10	Down	PRSS12
KRT13	13.4813561	2.21933848	0.34928154	6.35401028	2.10E-10	6.12E-10	Up	KRT13
CALN1	2.90733197	-1.97047665	0.31014159	-6.35347434	2.11E-10	6.14E-10	Down	CALN1
UMODL1	48.8944576	1.63981067	0.25818055	6.35141059	2.13E-10	6.22E-10	Up	UMODL1

ECSCR	52.1049237	-0.86059105	0.13554861	-6.34894783	2.17E-10	6.32E-10	Down	ECSCR
LHX1	2.87763225	3.87784284	0.61085189	6.34825379	2.18E-10	6.35E-10	Up	LHX1
CHI3L2	48.166629	-1.42210043	0.22403692	-6.34761652	2.19E-10	6.37E-10	Down	CHI3L2
CPB1	14.0056259	-3.03376519	0.47794045	-6.34757996	2.19E-10	6.38E-10	Down	CPB1
C20orf96	326.366776	0.61047457	0.09618149	6.34711075	2.19E-10	6.39E-10	Up	C20orf96
MT4	5.64699098	5.17649886	0.81558833	6.34695063	2.20E-10	6.40E-10	Up	MT4
B3GALNT2	446.754677	0.5608246	0.08838677	6.34511902	2.22E-10	6.47E-10	Up	B3GALNT2
PARD3B	703.188375	-0.67509464	0.10640115	-6.34480609	2.23E-10	6.48E-10	Down	PARD3B
WFS1	2915.66229	1.01085076	0.15935245	6.34349065	2.25E-10	6.54E-10	Up	WFS1
LAMC2	6262.04841	0.79326624	0.1250812	6.34200996	2.27E-10	6.60E-10	Up	LAMC2
TMEM135	681.911451	-0.53768693	0.08478626	-6.34167553	2.27E-10	6.61E-10	Down	TMEM135
ATG4D	949.112684	-0.67732445	0.10689032	-6.33663039	2.35E-10	6.83E-10	Down	ATG4D
PRSS8	9975.84469	-0.79570133	0.1255732	-6.33655371	2.35E-10	6.83E-10	Down	PRSS8
ALG5	2006.78768	0.61087898	0.09647924	6.33171405	2.42E-10	7.05E-10	Up	ALG5
ARL6IP4	74.8278635	0.71452762	0.11287998	6.3299765	2.45E-10	7.13E-10	Up	ARL6IP4
GAB2	1190.26036	-0.5311096	0.08391549	-6.32910104	2.47E-10	7.17E-10	Down	GAB2
OLFML2B	895.61828	1.06771387	0.1688049	6.32513566	2.53E-10	7.35E-10	Up	OLFML2B
ZNF431	462.331905	0.63611493	0.10059948	6.32324246	2.56E-10	7.44E-10	Up	ZNF431
TTLL3	579.846176	0.89465756	0.14149198	6.32302646	2.56E-10	7.45E-10	Up	TTLL3
BCL11B	461.662016	0.64357305	0.10178711	6.32273603	2.57E-10	7.46E-10	Up	BCL11B
IGFN1	44.2378877	1.82865552	0.28931665	6.32060249	2.61E-10	7.56E-10	Up	IGFN1
CDK12	2657.89387	0.65069464	0.10297788	6.31878059	2.64E-10	7.65E-10	Up	CDK12
C16orf91	626.730387	0.62474521	0.09888747	6.31773872	2.65E-10	7.70E-10	Up	C16orf91
GCK	7.57632342	-1.01532588	0.16071278	-6.3176424	2.66E-10	7.70E-10	Down	GCK
GPRC6A	5.99920238	3.20064427	0.50676092	6.3158862	2.69E-10	7.79E-10	Up	GPRC6A
TTC29	9.79644617	4.17508581	0.66115269	6.31485874	2.70E-10	7.84E-10	Up	TTC29
GTF3C5	2472.48463	0.51563345	0.08166493	6.31401289	2.72E-10	7.88E-10	Up	GTF3C5
CHURC1	1211.09232	-0.59232169	0.09381281	-6.31386766	2.72E-10	7.89E-10	Down	CHURC1
NXF3	69.1354878	2.43508999	0.38567778	6.31379383	2.72E-10	7.89E-10	Up	NXF3
NMUR1	50.3326997	-0.95185165	0.15076925	-6.31330099	2.73E-10	7.91E-10	Down	NMUR1
CCPG1	489.537526	-0.66378755	0.10514257	-6.31321398	2.73E-10	7.92E-10	Down	CCPG1
ZNF587B	357.154321	0.68260629	0.10813743	6.31239588	2.75E-10	7.96E-10	Up	ZNF587B
MAB21L3	66.7338036	-1.10291174	0.17473622	-6.31186675	2.76E-10	7.98E-10	Down	MAB21L3
MICAL3	1168.6918	-0.66418226	0.10528471	-6.30843981	2.82E-10	8.16E-10	Down	MICAL3
PITPNC1	766.227324	0.63958688	0.10139316	6.30798808	2.83E-10	8.18E-10	Up	PITPNC1
SIDT1	517.982729	-0.98183966	0.15565847	-6.30765329	2.83E-10	8.20E-10	Down	SIDT1
CACNA1C	622.179422	-1.14051262	0.18081543	-6.30760657	2.83E-10	8.20E-10	Down	CACNA1C
UBA52	20616.7739	0.63124433	0.10008809	6.30688747	2.85E-10	8.24E-10	Up	UBA52
UHRF2	1263.9131	0.50887277	0.08069468	6.30615009	2.86E-10	8.27E-10	Up	UHRF2
CSF3	108.448079	1.83265914	0.2906367	6.30567002	2.87E-10	8.30E-10	Up	CSF3
LRSAM1	1049.29561	0.50844328	0.08064448	6.30474981	2.89E-10	8.35E-10	Up	LRSAM1
ITGAX	576.184012	1.11769164	0.17729976	6.30396584	2.90E-10	8.39E-10	Up	ITGAX
RADIL	30.65167	-1.43362964	0.2274752	-6.30235563	2.93E-10	8.47E-10	Down	RADIL
PEX10	720.036952	0.5903268	0.09369295	6.3006537	2.96E-10	8.57E-10	Up	PEX10
CHD6	1606.75511	0.65206295	0.1034944	6.30046621	2.97E-10	8.57E-10	Up	CHD6
TBX10	154.412276	-1.65115775	0.26242707	-6.29187286	3.14E-10	9.06E-10	Down	TBX10
PLD4	60.9320396	-1.16760752	0.18559095	-6.2912955	3.15E-10	9.09E-10	Down	PLD4
MPPED1	1.32904254	-2.21594056	0.35226984	-6.29046344	3.17E-10	9.14E-10	Down	MPPED1
MAN2A1	2481.90938	-0.56022866	0.08906586	-6.29004943	3.17E-10	9.16E-10	Down	MAN2A1
ZNF30	128.729454	0.52282464	0.08314828	6.28785874	3.22E-10	9.29E-10	Up	ZNF30
CLCF1	208.582385	0.76991018	0.12246381	6.28683814	3.24E-10	9.35E-10	Up	CLCF1
EFCAB1	4.85711395	-1.45828591	0.23197159	-6.28648507	3.25E-10	9.37E-10	Down	EFCAB1
CKLF-CMTM1	13.1295469	0.6791994	0.10806556	6.28506779	3.28E-10	9.45E-10	Up	CKLF-CMTM1
FCRLB	29.5071109	1.06108447	0.16885091	6.28415014	3.30E-10	9.50E-10	Up	FCRLB
NCLN	6235.50551	0.67960529	0.10815813	6.28344154	3.31E-10	9.54E-10	Up	NCLN
HSP90B1	31999.9916	0.53478843	0.08511141	6.2833927	3.31E-10	9.54E-10	Up	HSP90B1
ANGPTL3	7.96904915	2.54828214	0.40560571	6.28265844	3.33E-10	9.59E-10	Up	ANGPTL3
SURF2	522.117383	0.65118663	0.103654	6.28231083	3.34E-10	9.61E-10	Up	SURF2

EIF4G3	2429.78641	-0.52718094	0.08391811	-6.28208814	3.34E-10	9.62E-10	Down	EIF4G3
PCDH17	238.815095	0.89620638	0.14266545	6.2818742	3.35E-10	9.63E-10	Up	PCDH17
FLRT1	46.2069561	-1.25804702	0.20029206	-6.28106284	3.36E-10	9.68E-10	Down	FLRT1
RAI2	272.206332	-1.14137845	0.18178149	-6.27884857	3.41E-10	9.81E-10	Down	RAI2
RALGAPA1	525.30291	-0.73631743	0.11728139	-6.27821177	3.42E-10	9.85E-10	Down	RALGAPA1
CEACAM3	33.261136	-1.19654023	0.19060746	-6.27751	3.44E-10	9.90E-10	Down	CEACAM3
POLE	2259.98274	0.51222577	0.08161127	6.27640952	3.46E-10	9.96E-10	Up	POLE
CATSPER1	15.2695686	1.21618503	0.19389122	6.27251205	3.55E-10	1.02E-09	Up	CATSPER1
SPDYA	21.5315694	1.24723024	0.19885414	6.27208586	3.56E-10	1.02E-09	Up	SPDYA
C10orf95	66.9998062	0.80484138	0.12832851	6.27172674	3.57E-10	1.03E-09	Up	C10orf95
MMP9	1751.9017	1.35476157	0.21605215	6.27053036	3.60E-10	1.03E-09	Up	MMP9
TRIM55	1.65693564	-1.83778011	0.29314757	-6.26912967	3.63E-10	1.04E-09	Down	TRIM55
BTBD11	112.038182	1.37537372	0.21940266	6.26871932	3.64E-10	1.05E-09	Up	BTBD11
INTS1	6183.7038	0.67239219	0.10728157	6.26754617	3.67E-10	1.05E-09	Up	INTS1
LUC7L3	3126.49739	0.56644417	0.09039717	6.26617131	3.70E-10	1.06E-09	Up	LUC7L3
ZNF276	690.441555	0.5425967	0.08660569	6.26513945	3.72E-10	1.07E-09	Up	ZNF276
MRM1	490.818723	0.61670499	0.09848326	6.26202879	3.80E-10	1.09E-09	Up	MRM1
GCSH	155.006782	0.66058383	0.10549992	6.26146316	3.81E-10	1.09E-09	Up	GCSH
ACBD4	397.344673	-0.55596247	0.08883471	-6.25839196	3.89E-10	1.12E-09	Down	ACBD4
POU5F2	4.68988539	3.37054999	0.53861332	6.25782888	3.90E-10	1.12E-09	Up	POU5F2
ANKRD61	22.6341519	1.1327962	0.18102547	6.25766207	3.91E-10	1.12E-09	Up	ANKRD61
HNF1A	724.552972	0.67544613	0.10795089	6.25697576	3.93E-10	1.13E-09	Up	HNF1A
SLC16A8	16.8433924	1.26333313	0.20195647	6.25547256	3.96E-10	1.14E-09	Up	SLC16A8
SLC6A3	4.27693428	2.15468467	0.34451686	6.25422119	4.00E-10	1.14E-09	Up	SLC6A3
SYT15	3.89106813	-1.42798882	0.22834438	-6.25366312	4.01E-10	1.15E-09	Down	SYT15
KCTD8	2.54804072	-2.72906569	0.43641266	-6.25340637	4.02E-10	1.15E-09	Down	KCTD8
PNMA5	9.47917366	2.90816284	0.46511382	6.25258319	4.04E-10	1.16E-09	Up	PNMA5
REG4	19516.8257	2.36624725	0.37847098	6.25212334	4.05E-10	1.16E-09	Up	REG4
TRAF3IP3	135.593614	-1.013371	0.162087	-6.25201886	4.05E-10	1.16E-09	Down	TRAF3IP3
EML5	10.164304	-1.56153412	0.24976307	-6.25206181	4.05E-10	1.16E-09	Down	EML5
PRR25	2.85587289	1.85528793	0.29674889	6.25204684	4.05E-10	1.16E-09	Up	PRR25
FOXL2NB	2.91400779	3.74576148	0.59924769	6.25077328	4.08E-10	1.17E-09	Up	FOXL2NB
RGS14	644.780016	0.68249579	0.10920401	6.24973195	4.11E-10	1.18E-09	Up	RGS14
SYBU	1260.41285	0.6688332	0.10702685	6.24920971	4.13E-10	1.18E-09	Up	SYBU
HAMP	14.9942185	1.54763484	0.24767239	6.24871767	4.14E-10	1.18E-09	Up	HAMP
NKX2-5	2.29825172	3.39720108	0.54367012	6.24864406	4.14E-10	1.18E-09	Up	NKX2-5
RPL29	27070.3697	0.62541623	0.10010008	6.24790915	4.16E-10	1.19E-09	Up	RPL29
B4GALT2	2064.88049	0.517846	0.08288717	6.24760142	4.17E-10	1.19E-09	Up	B4GALT2
C1QTNF1	1449.46595	0.76455554	0.12240818	6.24595117	4.21E-10	1.20E-09	Up	C1QTNF1
KCNK2	11.0970607	-1.95774463	0.31344563	-6.24588262	4.21E-10	1.20E-09	Down	KCNK2
EIF3C	348.50632	0.55543483	0.08893297	6.24554494	4.22E-10	1.21E-09	Up	EIF3C
CASP12	3.68310217	-1.29905888	0.2080004	-6.24546335	4.23E-10	1.21E-09	Down	CASP12
C17orf97	115.318058	-0.92884875	0.14872483	-6.24541802	4.23E-10	1.21E-09	Down	C17orf97
TTC30A	258.729687	-0.68352313	0.10945972	-6.24451765	4.25E-10	1.21E-09	Down	TTC30A
UHRF1BP1L	531.249458	-0.59308591	0.09500358	-6.24277454	4.30E-10	1.23E-09	Down	UHRF1BP1L
CD3E	388.966931	-1.01887569	0.16322494	-6.24215693	4.32E-10	1.23E-09	Down	CD3E
TUBA4B	4.46458948	1.56955694	0.25149993	6.24078488	4.35E-10	1.24E-09	Up	TUBA4B
CD47	4269.09883	0.51388696	0.08234398	6.24073503	4.36E-10	1.24E-09	Up	CD47
PTPRO	2028.02783	1.43243493	0.22953339	6.24063852	4.36E-10	1.24E-09	Up	PTPRO
FAT4	391.444544	-1.10232138	0.17668181	-6.23902035	4.40E-10	1.26E-09	Down	FAT4
NBPF15	280.41634	0.69944319	0.11211288	6.23874054	4.41E-10	1.26E-09	Up	NBPF15
FAM53B	1545.14811	-0.54002811	0.08657671	-6.23756785	4.44E-10	1.27E-09	Down	FAM53B
GREB1	54.4959048	1.01976817	0.16349181	6.23742672	4.45E-10	1.27E-09	Up	GREB1
ASB4	20.4834807	2.03621611	0.32651039	6.23629801	4.48E-10	1.28E-09	Up	ASB4
ZNF114	19.6718854	1.49480519	0.23978651	6.23390033	4.55E-10	1.30E-09	Up	ZNF114
ST8SIA4	245.32119	-0.95736238	0.15357886	-6.23368603	4.56E-10	1.30E-09	Down	ST8SIA4
CD59	7078.64176	-0.55481057	0.08902763	-6.23189187	4.61E-10	1.31E-09	Down	CD59
CBR1	2000.07168	-0.66043247	0.10597744	-6.23182117	4.61E-10	1.31E-09	Down	CBR1

FAM43B	7.83751233	-1.53231782	0.24593716	-6.23052585	4.65E-10	1.32E-09	Down	FAM43B
SRD5A3	924.778298	0.70147091	0.11258804	6.23042104	4.65E-10	1.32E-09	Up	SRD5A3
ASIC3	35.0328508	1.16681523	0.18727726	6.23041582	4.65E-10	1.32E-09	Up	ASIC3
HS3ST4	9.20796892	2.99316483	0.48050464	6.2292111	4.69E-10	1.33E-09	Up	HS3ST4
MYO5A	401.331691	-0.96850609	0.15548835	-6.22880166	4.70E-10	1.34E-09	Down	MYO5A
APRT	4716.82069	0.6506986	0.1044757	6.22822912	4.72E-10	1.34E-09	Up	APRT
TNFSF8	37.9456656	-1.12618605	0.18092113	-6.22473471	4.82E-10	1.37E-09	Down	TNFSF8
PSTK	116.399458	0.50536417	0.08120238	6.22351444	4.86E-10	1.38E-09	Up	PSTK
PAQR7	334.92228	0.56158045	0.09025333	6.22226828	4.90E-10	1.39E-09	Up	PAQR7
GNPNAT1	1909.84961	0.59474978	0.09560565	6.22086457	4.94E-10	1.41E-09	Up	GNPNAT1
SPAG4	233.227275	0.84828946	0.13645724	6.21652206	5.08E-10	1.44E-09	Up	SPAG4
FOXD2	530.223665	-0.93900741	0.1510583	-6.21619229	5.09E-10	1.45E-09	Down	FOXD2
TNFRSF4	134.409079	0.96701447	0.15556332	6.21621142	5.09E-10	1.45E-09	Up	TNFRSF4
CTLA4	73.2783697	1.09785137	0.17664389	6.21505443	5.13E-10	1.46E-09	Up	CTLA4
ZNF517	383.445016	0.66229331	0.10657653	6.21425097	5.16E-10	1.46E-09	Up	ZNF517
PIK3R2	46.1218102	0.6427325	0.10343282	6.21400913	5.16E-10	1.47E-09	Up	PIK3R2
PIK3C3	789.944886	-0.51870092	0.08349179	-6.21259811	5.21E-10	1.48E-09	Down	PIK3C3
KMT2B	2582.84091	0.51532047	0.08295061	6.21237728	5.22E-10	1.48E-09	Up	KMT2B
IDNK	168.982942	-0.61166642	0.09849261	-6.21027735	5.29E-10	1.50E-09	Down	IDNK
ACTBL2	2.72199706	2.91962779	0.47015571	6.20991662	5.30E-10	1.50E-09	Up	ACTBL2
KRT38	2.00541532	3.83098996	0.61696432	6.20941901	5.32E-10	1.51E-09	Up	KRT38
CECR2	79.7705106	1.85424387	0.29862877	6.20919372	5.33E-10	1.51E-09	Up	CECR2
AQP1	2254.85825	-1.05413532	0.16977052	-6.20917751	5.33E-10	1.51E-09	Down	AQP1
CAPG	5780.45415	0.72998665	0.1175664	6.20914331	5.33E-10	1.51E-09	Up	CAPG
ADM2	406.728853	0.86904098	0.13996266	6.20909143	5.33E-10	1.51E-09	Up	ADM2
KCTD16	29.961658	1.99181234	0.3208267	6.20837463	5.35E-10	1.52E-09	Up	KCTD16
COPRS	1042.79532	0.54883024	0.0884079	6.20793245	5.37E-10	1.52E-09	Up	COPRS
MUC19	2.15902187	3.15672844	0.50851425	6.20774827	5.37E-10	1.52E-09	Up	MUC19
MLH3	696.121034	0.51983932	0.08374929	6.20708931	5.40E-10	1.53E-09	Up	MLH3
RASAL2	1048.44456	0.60167154	0.09694661	6.20621507	5.43E-10	1.54E-09	Up	RASAL2
RAD51B	205.453229	0.64637233	0.10416249	6.20542347	5.45E-10	1.54E-09	Up	RAD51B
SPN	300.201833	-0.94734589	0.15267078	-6.20515513	5.46E-10	1.55E-09	Down	SPN
KATNAL1	238.665284	-0.91058652	0.14683681	-6.20135027	5.60E-10	1.58E-09	Down	KATNAL1
TOX2	116.31903	-0.88152912	0.14221853	-6.19841275	5.70E-10	1.61E-09	Down	TOX2
MTUS2	14.4079247	-1.44201209	0.23267821	-6.19745219	5.74E-10	1.62E-09	Down	MTUS2
PHLPP1	822.675615	-0.53431232	0.08626743	-6.1936741	5.88E-10	1.66E-09	Down	PHLPP1
SAMD1	1947.41116	0.51189928	0.0826892	6.19064281	5.99E-10	1.69E-09	Up	SAMD1
MAK	27.0946724	1.31262737	0.21205314	6.190087	6.01E-10	1.70E-09	Up	MAK
STX10	1697.22036	0.56020328	0.09055363	6.18642517	6.15E-10	1.74E-09	Up	STX10
ANKRD36	107.520551	1.25280917	0.20253807	6.18554916	6.19E-10	1.75E-09	Up	ANKRD36
CAMK1	142.016842	-0.566448	0.09164066	-6.1811867	6.36E-10	1.80E-09	Down	CAMK1
COCH	136.725941	1.35874921	0.21985016	6.18034216	6.40E-10	1.81E-09	Up	COCH
RBP1	772.548515	1.53148764	0.24782383	6.17974316	6.42E-10	1.81E-09	Up	RBP1
CD70	54.4590274	1.35521621	0.21932918	6.17891423	6.45E-10	1.82E-09	Up	CD70
PJA1	824.880781	0.62568214	0.10130364	6.1763047	6.56E-10	1.85E-09	Up	PJA1
PRKAA2	115.617409	-1.92705421	0.31208171	-6.17483864	6.62E-10	1.87E-09	Down	PRKAA2
INPP5J	621.767781	-0.85708062	0.13880855	-6.17455228	6.64E-10	1.87E-09	Down	INPP5J
GCAT	758.022361	0.81988812	0.13281006	6.17338854	6.68E-10	1.88E-09	Up	GCAT
CENPQ	252.771382	0.67375502	0.10915539	6.17243922	6.72E-10	1.90E-09	Up	CENPQ
YIF1B	1381.40741	0.66798123	0.108245	6.17101213	6.79E-10	1.91E-09	Up	YIF1B
ZSCAN30	270.560422	-0.59386643	0.09623514	-6.17099334	6.79E-10	1.91E-09	Down	ZSCAN30
LRRC34	62.4997031	1.04639184	0.16960968	6.16941094	6.85E-10	1.93E-09	Up	LRRC34
MAGEB5	0.94424396	2.94427475	0.47748168	6.16625701	6.99E-10	1.97E-09	Up	MAGEB5
KCNAB2	1067.41241	0.76350916	0.12384535	6.16502085	7.05E-10	1.98E-09	Up	KCNAB2
REG1A	11476.7372	2.67064192	0.43320412	6.16485802	7.05E-10	1.99E-09	Up	REG1A
SLC39A5	3213.77866	-1.15921811	0.18804072	-6.16471851	7.06E-10	1.99E-09	Down	SLC39A5
RGS22	6.81914062	-1.72818767	0.28036376	-6.16409089	7.09E-10	1.99E-09	Down	RGS22
RPLP0	84889.3056	0.6706867	0.10882922	6.16274478	7.15E-10	2.01E-09	Up	RPLP0

RPL14	23158.4603	0.50118693	0.08132757	6.16257075	7.16E-10	2.01E-09	Up	RPL14
BTN1A1	20.4065311	1.64273306	0.26673091	6.15876532	7.33E-10	2.06E-09	Up	BTN1A1
ZC3H12A	1807.57478	0.91200384	0.14809349	6.15829816	7.35E-10	2.07E-09	Up	ZC3H12A
HERC1	1461.05798	-0.58771787	0.09545515	-6.15700507	7.41E-10	2.08E-09	Down	HERC1
MYO7B	5240.56317	-0.74567387	0.12112166	-6.15640384	7.44E-10	2.09E-09	Down	MYO7B
SLC22A31	35.8783203	2.03107973	0.32991363	6.15639827	7.44E-10	2.09E-09	Up	SLC22A31
RNF126	2186.49195	0.69180923	0.11238345	6.15579289	7.47E-10	2.10E-09	Up	RNF126
CANT1	8315.24047	-0.54156932	0.08797769	-6.15575758	7.47E-10	2.10E-09	Down	CANT1
SLC35A5	891.634421	-0.5017124	0.08151379	-6.15493913	7.51E-10	2.11E-09	Down	SLC35A5
REPIN1	8587.14097	0.57227308	0.09298823	6.15425274	7.54E-10	2.12E-09	Up	REPIN1
ACKR4	28.1477206	-1.47508622	0.23969598	-6.15398807	7.56E-10	2.12E-09	Down	ACKR4
CGB7	2.25607133	2.56932834	0.41750629	6.15398721	7.56E-10	2.12E-09	Up	CGB7
CLCA1	12924.5111	-2.52458348	0.41028662	-6.15321917	7.59E-10	2.13E-09	Down	CLCA1
PTPRJ	2038.29868	-0.55131582	0.08962046	-6.1516734	7.67E-10	2.15E-09	Down	PTPRJ
PLEKHG4B	16.0290165	-1.69331096	0.27528327	-6.15115826	7.69E-10	2.16E-09	Down	PLEKHG4B
WFIKKN2	5.70951657	-1.73074192	0.28138502	-6.15079621	7.71E-10	2.16E-09	Down	WFIKKN2
CD3G	102.881993	-1.02663661	0.16694207	-6.14965769	7.77E-10	2.18E-09	Down	CD3G
PUF60	7212.20794	0.69569895	0.1131403	6.14899337	7.80E-10	2.19E-09	Up	PUF60
MDGA1	106.15794	-1.12180456	0.18245676	-6.14833098	7.83E-10	2.20E-09	Down	MDGA1
CLPP	2535.83694	0.70142759	0.1140948	6.14776103	7.86E-10	2.20E-09	Up	CLPP
SLC18A3	4.21159107	-2.79027524	0.45391342	-6.14715299	7.89E-10	2.21E-09	Down	SLC18A3
BTNL9	287.895167	1.11996306	0.18219867	6.14693335	7.90E-10	2.21E-09	Up	BTNL9
COL9A1	175.579502	1.53105886	0.24916618	6.14472993	8.01E-10	2.24E-09	Up	COL9A1
ADSL	2355.26012	0.55038156	0.08957523	6.14434995	8.03E-10	2.25E-09	Up	ADSL
LRRC17	66.2532511	-0.97949449	0.15943852	-6.14339912	8.08E-10	2.26E-09	Down	LRRC17
LOX	735.315876	1.10081666	0.17922515	6.14208816	8.14E-10	2.28E-09	Up	LOX
MBOAT4	4.34139868	-1.02502749	0.16689674	-6.14168679	8.16E-10	2.29E-09	Down	MBOAT4
ANKUB1	3.00921801	2.92039647	0.47550941	6.14161662	8.17E-10	2.29E-09	Up	ANKUB1
PSMA8	0.90238012	-2.06774865	0.33668128	-6.14156114	8.17E-10	2.29E-09	Down	PSMA8
ACSM1	27.232368	-1.09531057	0.17835976	-6.14101827	8.20E-10	2.29E-09	Down	ACSM1
MLLT11	140.652219	-0.87925088	0.14319221	-6.14035405	8.23E-10	2.30E-09	Down	MLLT11
PRKCA	1524.37708	-0.6208619	0.10113321	-6.1390504	8.30E-10	2.32E-09	Down	PRKCA
STARD5	236.782898	-0.61087538	0.09951632	-6.13844439	8.33E-10	2.33E-09	Down	STARD5
ZNF337	88.6230526	0.83556566	0.13612702	6.13813235	8.35E-10	2.33E-09	Up	ZNF337
CAPS	635.956296	1.13997672	0.18577566	6.13630821	8.45E-10	2.36E-09	Up	CAPS
CYP8B1	2.5871742	-1.58739814	0.25871209	-6.13577106	8.47E-10	2.37E-09	Down	CYP8B1
C19orf71	58.8423396	0.73232869	0.11935857	6.13553498	8.49E-10	2.37E-09	Up	C19orf71
RETN	7.08618703	1.94906783	0.31769245	6.13507761	8.51E-10	2.38E-09	Up	RETN
TMEM61	56.2408641	-1.4300781	0.23310383	-6.13494029	8.52E-10	2.38E-09	Down	TMEM61
TPK1	285.748909	-0.68416114	0.11152726	-6.13447463	8.54E-10	2.39E-09	Down	TPK1
CBWD3	27.3165531	1.01974029	0.16623747	6.13423843	8.56E-10	2.39E-09	Up	CBWD3
SHC4	25.6761846	-1.07396667	0.17515664	-6.13146432	8.71E-10	2.43E-09	Down	SHC4
LIPA	2595.59151	-0.6371052	0.10391763	-6.13086738	8.74E-10	2.44E-09	Down	LIPA
ELFN2	41.6403166	1.76634594	0.28816104	6.1297182	8.80E-10	2.46E-09	Up	ELFN2
TOMM5	916.702922	0.52245596	0.08526889	6.12715808	8.95E-10	2.50E-09	Up	TOMM5
MDFIC	475.758253	-0.96361334	0.15728451	-6.12656231	8.98E-10	2.50E-09	Down	MDFIC
EVL	699.973709	-0.80618074	0.13161802	-6.1251546	9.06E-10	2.53E-09	Down	EVL
MOSPD1	486.956419	0.63724304	0.10404193	6.12486736	9.08E-10	2.53E-09	Up	MOSPD1
IFT140	868.40489	0.58120437	0.09489781	6.12452872	9.10E-10	2.53E-09	Up	IFT140
PDK3	509.921608	0.59776914	0.09763675	6.12237835	9.22E-10	2.57E-09	Up	PDK3
ADORA2A	6.57557376	-1.018059	0.16630588	-6.12160545	9.26E-10	2.58E-09	Down	ADORA2A
C16orf54	110.489824	-1.35570958	0.22151351	-6.12021159	9.35E-10	2.60E-09	Down	C16orf54
FBXO39	6.21210972	1.64876393	0.26944295	6.11915777	9.41E-10	2.62E-09	Up	FBXO39
TMEM196	0.94981471	-2.79628786	0.45697884	-6.11907513	9.41E-10	2.62E-09	Down	TMEM196
AGMAT	1461.55176	0.66549326	0.10875965	6.11893529	9.42E-10	2.62E-09	Up	AGMAT
EFNA5	161.954375	-1.37351818	0.22452125	-6.11754196	9.50E-10	2.65E-09	Down	EFNA5
GHDC	1781.71469	0.56362043	0.0921487	6.11642289	9.57E-10	2.66E-09	Up	GHDC
PARD6B	639.710037	0.70135386	0.11466984	6.11628892	9.58E-10	2.67E-09	Up	PARD6B

DEFB4A	2.18898906	3.92042689	0.64099664	6.11614266	9.59E-10	2.67E-09	Up	DEFB4A
PLEKHA6	4405.30078	-0.54805373	0.08961099	-6.11592111	9.60E-10	2.67E-09	Down	PLEKHA6
FAM3D	8778.40904	-1.14677187	0.18750952	-6.11580603	9.61E-10	2.67E-09	Down	FAM3D
RBM43	202.928332	-0.69639802	0.1138956	-6.11435397	9.69E-10	2.70E-09	Down	RBM43
INTS2	611.142407	0.51437167	0.08412674	6.11424665	9.70E-10	2.70E-09	Up	INTS2
FBLN2	1674.51821	-1.29932108	0.21252758	-6.11365877	9.74E-10	2.71E-09	Down	FBLN2
GYPE	4.05206266	-1.30743679	0.21386903	-6.11325919	9.76E-10	2.71E-09	Down	GYPE
DOK4	3635.37665	-0.64619933	0.10571125	-6.11287188	9.79E-10	2.72E-09	Down	DOK4
NPAS3	18.4035376	-1.48792665	0.24342496	-6.11246532	9.81E-10	2.73E-09	Down	NPAS3
NLRC3	140.740234	-0.80746078	0.1321231	-6.1114278	9.87E-10	2.74E-09	Down	NLRC3
CHCHD2	9708.55367	0.56344611	0.09219974	6.11114653	9.89E-10	2.75E-09	Up	CHCHD2
CD14	1890.47329	-0.97390916	0.15940735	-6.10956264	9.99E-10	2.77E-09	Down	CD14
CA6	5.78133554	3.22269563	0.52749923	6.10938448	1.00E-09	2.78E-09	Up	CA6
SH3GL1	3402.85151	0.50772126	0.08313088	6.10749289	1.01E-09	2.81E-09	Up	SH3GL1
CTSA	10785.5616	-0.65150822	0.10673052	-6.10423547	1.03E-09	2.87E-09	Down	CTSA
PTGIS	386.285122	-1.50710417	0.24691576	-6.10371791	1.04E-09	2.87E-09	Down	PTGIS
RPL37A	34050.1179	0.56266433	0.09218406	6.10370552	1.04E-09	2.87E-09	Up	RPL37A
SLC39A3	826.74816	0.72772077	0.1192498	6.10249063	1.04E-09	2.90E-09	Up	SLC39A3
MROH1	2785.07913	0.64730449	0.10607877	6.10211185	1.05E-09	2.90E-09	Up	MROH1
IGFBP3	4401.13243	0.86341316	0.14153614	6.10030158	1.06E-09	2.93E-09	Up	IGFBP3
SMC1B	12.8815907	1.61914616	0.26555094	6.0973091	1.08E-09	2.99E-09	Up	SMC1B
MGAT2	161.407764	-0.66176933	0.10858628	-6.09441041	1.10E-09	3.04E-09	Down	MGAT2
METTL7B	1122.18165	-0.70293518	0.11538199	-6.09224327	1.11E-09	3.08E-09	Down	METTL7B
PCDH11X	1.11797465	-2.62711376	0.43123215	-6.09211012	1.11E-09	3.09E-09	Down	PCDH11X
DOK2	224.124583	-0.95708937	0.15710993	-6.09184528	1.12E-09	3.09E-09	Down	DOK2
HEYL	403.530257	0.76010022	0.12481764	6.08968608	1.13E-09	3.13E-09	Up	HEYL
WNT7A	6.86911976	2.67095785	0.43890347	6.08552463	1.16E-09	3.21E-09	Up	WNT7A
TCTA	1292.96005	-0.5133395	0.08435868	-6.08520076	1.16E-09	3.22E-09	Down	TCTA
EBP	4243.37716	0.67431453	0.11092361	6.0790893	1.21E-09	3.34E-09	Up	EBP
SLCO1C1	8.74927047	-1.27846791	0.2103118	-6.07891673	1.21E-09	3.35E-09	Down	SLCO1C1
CLYBL	511.839086	-0.66681382	0.10969568	-6.07876073	1.21E-09	3.35E-09	Down	CLYBL
SDC4	10174.3239	0.59606296	0.09807255	6.07777582	1.22E-09	3.37E-09	Up	SDC4
NHS	426.897242	1.00117353	0.16473245	6.07757341	1.22E-09	3.37E-09	Up	NHS
IZUMO4	52.8249073	0.90699259	0.1492529	6.07688419	1.23E-09	3.39E-09	Up	IZUMO4
LDB2	229.594987	-0.76385369	0.1257236	-6.07565886	1.23E-09	3.41E-09	Down	LDB2
AGR3	1395.70079	-1.41937132	0.23363732	-6.07510529	1.24E-09	3.42E-09	Down	AGR3
AMER2	1.89488591	-2.66174739	0.43815198	-6.07494098	1.24E-09	3.42E-09	Down	AMER2
SMPDL3B	1104.44631	-0.74077751	0.12194814	-6.07452881	1.24E-09	3.43E-09	Down	SMPDL3B
PDGFRB	3205.2735	0.8767805	0.14439446	6.07211991	1.26E-09	3.48E-09	Up	PDGFRB
SLC41A1	724.615625	0.59633185	0.09827871	6.06776208	1.30E-09	3.58E-09	Up	SLC41A1
TMEM144	816.573412	-0.59369759	0.09784612	-6.0676662	1.30E-09	3.58E-09	Down	TMEM144
KRT12	19.0541161	-1.3491162	0.22239776	-6.06623105	1.31E-09	3.61E-09	Down	KRT12
PAX2	4.30701949	2.42482178	0.39975326	6.06579617	1.31E-09	3.62E-09	Up	PAX2
ZNF366	39.0803097	-0.95262281	0.15707467	-6.06477666	1.32E-09	3.64E-09	Down	ZNF366
ZNF107	367.355087	0.6920128	0.1141348	6.06311839	1.34E-09	3.68E-09	Up	ZNF107
SLC30A1	1910.67471	-0.5281264	0.08713573	-6.06096257	1.35E-09	3.73E-09	Down	SLC30A1
NDNF	206.579509	-1.18289199	0.19519022	-6.06020106	1.36E-09	3.74E-09	Down	NDNF
ITIH6	7.2753465	2.30804202	0.38091316	6.05923411	1.37E-09	3.76E-09	Up	ITIH6
EPSTI1	1838.2371	0.90633701	0.14960146	6.05834344	1.38E-09	3.78E-09	Up	EPSTI1
ESCO2	444.593271	0.83628763	0.13807779	6.05664125	1.39E-09	3.82E-09	Up	ESCO2
CEP152	279.016114	0.68631036	0.11339674	6.05229333	1.43E-09	3.93E-09	Up	CEP152
LYPD6B	157.301446	1.18188224	0.19527963	6.05225578	1.43E-09	3.93E-09	Up	LYPD6B
PCDHA3	6.34237524	-1.48952028	0.24615492	-6.05114984	1.44E-09	3.95E-09	Down	PCDHA3
SYCE3	6.92097866	1.31037786	0.2166146	6.04935144	1.45E-09	4.00E-09	Up	SYCE3
CTBS	697.798302	-0.56050691	0.09265771	-6.0492206	1.46E-09	4.00E-09	Down	CTBS
FSCN2	21.0193213	1.01016464	0.16699259	6.04915847	1.46E-09	4.00E-09	Up	FSCN2
WFDC3	84.5626121	1.24238331	0.20541469	6.04817178	1.46E-09	4.03E-09	Up	WFDC3
MON1A	616.733026	0.58880462	0.09737589	6.04671896	1.48E-09	4.06E-09	Up	MON1A

ABI3	319.686912	-0.78681559	0.1301451	-6.04567986	1.49E-09	4.09E-09	Down	ABI3
BLOC1S1	1340.35582	-0.62378251	0.10320193	-6.04429117	1.50E-09	4.12E-09	Down	BLOC1S1
SYNGR2	13508.1571	0.6666905	0.11033168	6.0426024	1.52E-09	4.16E-09	Up	SYNGR2
KLF1	4.94556237	1.2689573	0.21000439	6.04252735	1.52E-09	4.16E-09	Up	KLF1
WIPF1	1155.13127	-0.81252787	0.13449529	-6.04131075	1.53E-09	4.20E-09	Down	WIPF1
SLC16A13	286.101489	0.61413792	0.10167438	6.04024278	1.54E-09	4.22E-09	Up	SLC16A13
ACOT4	140.661618	-0.81876418	0.13555537	-6.040072	1.54E-09	4.23E-09	Down	ACOT4
KLHL33	3.75485298	-1.24853334	0.20672512	-6.03958216	1.55E-09	4.24E-09	Down	KLHL33
TIMM10	1034.24224	0.69933773	0.1158149	6.03840901	1.56E-09	4.27E-09	Up	TIMM10
ZMYND12	41.5567587	-0.91905072	0.15224019	-6.03684688	1.57E-09	4.31E-09	Down	ZMYND12
RIIAD1	31.709395	1.69077326	0.28019458	6.03428257	1.60E-09	4.38E-09	Up	RIIAD1
IDUA	493.927561	0.88267084	0.1462813	6.03406465	1.60E-09	4.38E-09	Up	IDUA
ODF3L2	12.1960239	1.34049638	0.22226029	6.03120044	1.63E-09	4.46E-09	Up	ODF3L2
FMO2	41.3431211	-1.82269545	0.30236501	-6.02812954	1.66E-09	4.54E-09	Down	FMO2
FTH1	17223.7909	-0.72659079	0.12053583	-6.02800675	1.66E-09	4.55E-09	Down	FTH1
PDE4C	427.277842	-1.17711892	0.19527761	-6.0279257	1.66E-09	4.55E-09	Down	PDE4C
CA11	111.251355	-0.82864203	0.13750029	-6.02647463	1.68E-09	4.59E-09	Down	CA11
OR5K2	0.64900135	-1.80836209	0.30009948	-6.02587554	1.68E-09	4.61E-09	Down	OR5K2
PRND	25.0645316	-1.77544109	0.29468556	-6.02486626	1.69E-09	4.63E-09	Down	PRND
NBEAL1	439.653223	-1.07200338	0.17794675	-6.02429307	1.70E-09	4.65E-09	Down	NBEAL1
CFI	639.65413	1.07842163	0.17904274	6.0232635	1.71E-09	4.68E-09	Up	CFI
TAF1B	339.408682	0.51166213	0.08498948	6.02029938	1.74E-09	4.76E-09	Up	TAF1B
HHATL	1.06244137	-2.21987092	0.36874821	-6.02001818	1.74E-09	4.77E-09	Down	HHATL
ARHGAP31	599.619329	-0.77718432	0.12913052	-6.01859522	1.76E-09	4.81E-09	Down	ARHGAP31
CAPN14	16.072098	1.83451233	0.30482561	6.0182355	1.76E-09	4.82E-09	Up	CAPN14
FAM161A	104.909954	0.68162701	0.1132701	6.0177137	1.77E-09	4.84E-09	Up	FAM161A
RASSF8	259.617022	-1.17278963	0.19490636	-6.01719525	1.77E-09	4.85E-09	Down	RASSF8
SSC5D	519.447108	-1.33354668	0.22173354	-6.01418569	1.81E-09	4.94E-09	Down	SSC5D
RSPO4	17.9129016	2.45329676	0.40794071	6.01385614	1.81E-09	4.95E-09	Up	RSPO4
ROGDI	763.109619	-0.60121374	0.09999897	-6.01219933	1.83E-09	5.00E-09	Down	ROGDI
PCDHB4	39.9788893	-1.14191762	0.18993907	-6.0120206	1.83E-09	5.01E-09	Down	PCDHB4
LGI3	8.37745956	-1.84688206	0.30726584	-6.01069767	1.85E-09	5.05E-09	Down	LGI3
ITGB3BP	482.81509	0.62332139	0.10370941	6.01026834	1.85E-09	5.06E-09	Up	ITGB3BP
PRRG4	975.06531	-0.61621748	0.10255275	-6.00878535	1.87E-09	5.10E-09	Down	PRRG4
ABHD2	8018.86149	0.79273128	0.13195504	6.00758616	1.88E-09	5.14E-09	Up	ABHD2
ZC3H3	1335.657	0.58736307	0.09777314	6.00740715	1.89E-09	5.14E-09	Up	ZC3H3
EGLN3	1986.69743	-0.96567236	0.16076448	-6.00675206	1.89E-09	5.16E-09	Down	EGLN3
GREB1L	44.4206909	2.10457061	0.35042146	6.0058268	1.90E-09	5.19E-09	Up	GREB1L
AK5	26.4207664	-1.38378709	0.23042219	-6.0054419	1.91E-09	5.20E-09	Down	AK5
FUNDC1	488.794729	0.51404147	0.08560003	6.00515549	1.91E-09	5.21E-09	Up	FUNDC1
RASSF5	498.397989	-0.82624669	0.13762135	-6.00376832	1.93E-09	5.26E-09	Down	RASSF5
TRAT1	23.2488511	-1.26785419	0.21122695	-6.00233156	1.95E-09	5.30E-09	Down	TRAT1
BAG2	470.761575	-1.12372881	0.18724326	-6.00143806	1.96E-09	5.33E-09	Down	BAG2
FKBP14	495.238398	0.56950932	0.09491702	6.00007581	1.97E-09	5.37E-09	Up	FKBP14
C1QBP	5674.87594	0.67360529	0.11229022	5.99878848	1.99E-09	5.41E-09	Up	C1QBP
CDKN2C	383.549263	-0.61953648	0.10328929	-5.99807065	2.00E-09	5.44E-09	Down	CDKN2C
SLC39A13	1176.03506	0.52022457	0.08673648	5.99775947	2.00E-09	5.45E-09	Up	SLC39A13
CGB5	2.51320049	4.20298605	0.70089015	5.99664022	2.01E-09	5.48E-09	Up	CGB5
TBX3	1283.1723	0.93971216	0.15676384	5.99444458	2.04E-09	5.56E-09	Up	TBX3
FAR2	1801.63681	-0.55211832	0.09211955	-5.99349771	2.05E-09	5.59E-09	Down	FAR2
HECA	1481.54568	-0.50668178	0.08456078	-5.99192387	2.07E-09	5.64E-09	Down	HECA
ARHGAP9	257.069067	-0.88751758	0.14819123	-5.98900213	2.11E-09	5.74E-09	Down	ARHGAP9
STOML2	5307.89543	0.55725174	0.09304957	5.98876216	2.11E-09	5.75E-09	Up	STOML2
MRC1	592.752222	-1.28987778	0.21539202	-5.98851236	2.12E-09	5.76E-09	Down	MRC1
CAPN9	371.03707	-1.52565527	0.25477431	-5.98826184	2.12E-09	5.77E-09	Down	CAPN9
KLHL4	21.2500319	-1.23321485	0.20594805	-5.98799009	2.12E-09	5.78E-09	Down	KLHL4
KLHL34	24.6301865	-1.88036189	0.31404799	-5.98749848	2.13E-09	5.79E-09	Down	KLHL34
LCE1E	1.96585899	3.99057025	0.66673834	5.98521189	2.16E-09	5.87E-09	Up	LCE1E

ZNF175	181.18915	-0.66493983	0.11111242	-5.98438775	2.17E-09	5.90E-09	Down	ZNF175
FGF7	223.967264	-1.38188766	0.23093221	-5.98395377	2.18E-09	5.92E-09	Down	FGF7
SYTL3	107.898919	-0.61500333	0.10280708	-5.9821108	2.20E-09	5.98E-09	Down	SYTL3
ZFP41	433.37493	0.7063166	0.11808063	5.98164676	2.21E-09	6.00E-09	Up	ZFP41
ZNF134	255.916802	-0.95291705	0.15931771	-5.98123729	2.21E-09	6.01E-09	Down	ZNF134
CCL11	190.370092	-1.30031562	0.21740873	-5.98097245	2.22E-09	6.02E-09	Down	CCL11
VIM	9786.07281	-0.78580583	0.13139019	-5.9807041	2.22E-09	6.03E-09	Down	VIM
TRABD2B	63.9398736	-1.08989496	0.18223499	-5.9807119	2.22E-09	6.03E-09	Down	TRABD2B
SPAG7	1086.51786	-0.50257264	0.08403446	-5.98055433	2.22E-09	6.03E-09	Down	SPAG7
CRYBG3	467.274783	-0.90325258	0.15103647	-5.9803607	2.23E-09	6.04E-09	Down	CRYBG3
BHLHA15	37.921993	-1.10337962	0.1845026	-5.9802932	2.23E-09	6.04E-09	Down	BHLHA15
RXFP3	0.74206244	2.26604344	0.37893533	5.98002678	2.23E-09	6.05E-09	Up	RXFP3
DEPDC1	514.451004	0.74270061	0.12420097	5.97982944	2.23E-09	6.06E-09	Up	DEPDC1
RDH11	2209.25499	0.50362767	0.08422216	5.97975252	2.23E-09	6.06E-09	Up	RDH11
OCIAD2	3357.76559	0.56279232	0.09412844	5.97898305	2.25E-09	6.09E-09	Up	OCIAD2
PNPLA1	28.3136621	1.29869019	0.21723871	5.9781711	2.26E-09	6.11E-09	Up	PNPLA1
HGD	364.471418	-1.07807553	0.18034149	-5.9779674	2.26E-09	6.12E-09	Down	HGD
UAP1L1	660.148309	0.75176746	0.1257708	5.97728138	2.27E-09	6.15E-09	Up	UAP1L1
PLIN3	3379.71628	-0.57186849	0.09568155	-5.97678985	2.28E-09	6.16E-09	Down	PLIN3
NCAM2	49.8079659	-1.34079575	0.22436325	-5.97600439	2.29E-09	6.19E-09	Down	NCAM2
FGF13	108.615047	-1.26788585	0.21217033	-5.9757923	2.29E-09	6.20E-09	Down	FGF13
RPS16	38799.3266	0.73561429	0.12310611	5.97544889	2.29E-09	6.21E-09	Up	RPS16
EID3	21.9961709	-0.92888317	0.15548672	-5.97403522	2.31E-09	6.26E-09	Down	EID3
CYB5A	1946.67273	-0.56282922	0.09422344	-5.9733458	2.32E-09	6.29E-09	Down	CYB5A
PCDH1	4488.01163	-0.59893478	0.10028397	-5.97238834	2.34E-09	6.32E-09	Down	PCDH1
DHRS1	928.003216	-0.62111459	0.10400692	-5.9718584	2.35E-09	6.34E-09	Down	DHRS1
SEZ6	9.6098827	-1.91221066	0.32024075	-5.97116599	2.36E-09	6.37E-09	Down	SEZ6
NIPAL4	28.3456971	-1.39002408	0.23287442	-5.96898573	2.39E-09	6.46E-09	Down	NIPAL4
SPTLC3	204.490014	-1.1058545	0.18527122	-5.96884134	2.39E-09	6.46E-09	Down	SPTLC3
SHISA7	2.04215325	-1.31530686	0.22037129	-5.96859442	2.39E-09	6.47E-09	Down	SHISA7
LPIN2	1845.08558	-0.55616505	0.09321592	-5.96641705	2.43E-09	6.55E-09	Down	LPIN2
NRIP2	140.611395	-0.62140996	0.10415709	-5.96608415	2.43E-09	6.57E-09	Down	NRIP2
CPSF4L	5.95344038	1.17777422	0.19744523	5.96506794	2.45E-09	6.61E-09	Up	CPSF4L
STK4	2363.34293	0.52499738	0.08801649	5.96476131	2.45E-09	6.62E-09	Up	STK4
IL36G	4.3677221	1.67498395	0.28081709	5.96467948	2.45E-09	6.62E-09	Up	IL36G
A2ML1	10.2267321	2.18995356	0.36716654	5.96446932	2.45E-09	6.63E-09	Up	A2ML1
SRR	149.010866	-0.51466908	0.08631834	-5.96245362	2.48E-09	6.71E-09	Down	SRR
PIGR	121308.032	-1.66731785	0.2797145	-5.96078443	2.51E-09	6.77E-09	Down	PIGR
RPL30	24767.1911	0.54906052	0.09212465	5.95997391	2.52E-09	6.81E-09	Up	RPL30
SLX4IP	472.911908	0.63690851	0.10688707	5.95870512	2.54E-09	6.86E-09	Up	SLX4IP
ITGA4	366.981539	-0.91022724	0.15278327	-5.95763669	2.56E-09	6.90E-09	Down	ITGA4
EBF4	177.568641	-0.94618627	0.15894307	-5.95298859	2.63E-09	7.10E-09	Down	EBF4
IL3RA	322.505454	-0.70137456	0.11785353	-5.95123913	2.66E-09	7.17E-09	Down	IL3RA
PRRG2	391.955769	-0.57365126	0.09641098	-5.95006177	2.68E-09	7.22E-09	Down	PRRG2
BATF	173.96236	1.13184706	0.19024499	5.9494184	2.69E-09	7.25E-09	Up	BATF
FBXO16	63.186169	1.01474403	0.17056664	5.94925278	2.69E-09	7.26E-09	Up	FBXO16
CYHR1	2133.19323	0.62045901	0.10430864	5.94829934	2.71E-09	7.30E-09	Up	CYHR1
USH2A	11.9335072	1.41915385	0.23862134	5.94730484	2.73E-09	7.34E-09	Up	USH2A
CPNE2	2093.99047	-0.60786748	0.10225246	-5.94477103	2.77E-09	7.45E-09	Down	CPNE2
NFIC	2375.73229	-0.66984289	0.11268259	-5.94451121	2.77E-09	7.46E-09	Down	NFIC
ABCG1	1364.27901	-0.71337691	0.12005143	-5.94226096	2.81E-09	7.57E-09	Down	ABCG1
CLEC4M	2.17046928	-2.70463635	0.45519068	-5.94176563	2.82E-09	7.59E-09	Down	CLEC4M
FAM169A	141.654862	1.2026115	0.2024267	5.94097265	2.83E-09	7.62E-09	Up	FAM169A
ITPRIP	863.190848	0.60432124	0.10174865	5.93935373	2.86E-09	7.70E-09	Up	ITPRIP
LTF	190.542701	-1.27576748	0.21493048	-5.93572165	2.93E-09	7.87E-09	Down	LTF
MFNG	331.097533	-0.80359745	0.13542702	-5.93380456	2.96E-09	7.96E-09	Down	MFNG
EMP3	729.902072	-0.86794091	0.14629296	-5.93289587	2.98E-09	8.00E-09	Down	EMP3
SLC5A1	3422.06663	0.80570229	0.13580706	5.93269797	2.98E-09	8.01E-09	Up	SLC5A1

NXN	584.192416	-1.0077755	0.1700316	-5.92698951	3.09E-09	8.29E-09	Down	NXN
EVC2	49.7867323	-0.97818307	0.16506362	-5.92609724	3.10E-09	8.33E-09	Down	EVC2
ADAMTS18	15.7912307	1.47406748	0.24875749	5.92572093	3.11E-09	8.35E-09	Up	ADAMTS18
PARVB	1137.60306	0.95232304	0.160722	5.92528114	3.12E-09	8.37E-09	Up	PARVB
TRIM46	41.4268187	0.91528246	0.15448879	5.92458826	3.13E-09	8.40E-09	Up	TRIM46
SYNJ2BP-CC	5.620339	-0.72074161	0.1216773	-5.92338583	3.15E-09	8.46E-09	Down	SYNJ2BP-COX16
MYDGF	4430.89925	0.62881533	0.1061637	5.92307259	3.16E-09	8.48E-09	Up	MYDGF
FOXG1	10.1767271	3.68581434	0.62231452	5.92275162	3.17E-09	8.49E-09	Up	FOXG1
WWOX	356.611633	0.53914036	0.09103485	5.92235143	3.17E-09	8.51E-09	Up	WWOX
P2RX7	102.00282	-1.01640052	0.17164772	-5.92143319	3.19E-09	8.56E-09	Down	P2RX7
GYG2	681.568025	0.97432719	0.16459984	5.91936906	3.23E-09	8.67E-09	Up	GYG2
USP27X	169.943474	0.61756087	0.10434586	5.91840323	3.25E-09	8.71E-09	Up	USP27X
PMCH	1.58209741	1.73154503	0.29268443	5.91608189	3.30E-09	8.84E-09	Up	PMCH
UGT1A5	0.80384156	-3.77849547	0.63876448	-5.91531867	3.31E-09	8.88E-09	Down	UGT1A5
INSR	2681.83727	-0.52003949	0.08793322	-5.91402782	3.34E-09	8.95E-09	Down	INSR
TVP23A	27.3209919	-0.64609985	0.1092543	-5.91372469	3.34E-09	8.96E-09	Down	TVP23A
ERVV-1	2.35930087	2.89318441	0.48926997	5.91326786	3.35E-09	8.98E-09	Up	ERVV-1
ZNF804A	9.66876168	-1.27203213	0.21515243	-5.91223696	3.37E-09	9.04E-09	Down	ZNF804A
GNE	2671.66709	-0.95793204	0.16204302	-5.91159083	3.39E-09	9.07E-09	Down	GNE
SOHLH1	2.33553135	3.56105059	0.60247545	5.91069828	3.41E-09	9.12E-09	Up	SOHLH1
ELMO1	384.314573	-0.81792347	0.13842075	-5.90896578	3.44E-09	9.21E-09	Down	ELMO1
ERVV-2	1.85254361	3.7578054	0.63596993	5.90877838	3.45E-09	9.22E-09	Up	ERVV-2
NUBP2	2103.39632	0.62824663	0.10633416	5.90822966	3.46E-09	9.25E-09	Up	NUBP2
ERAP1	3354.17523	-0.55883103	0.09459695	-5.90749511	3.47E-09	9.29E-09	Down	ERAP1
OR2W3	8.55063973	-1.89148671	0.3202851	-5.90563441	3.51E-09	9.40E-09	Down	OR2W3
CHST1	98.6403211	0.94465389	0.15998293	5.90471677	3.53E-09	9.45E-09	Up	CHST1
MEIG1	5.72711611	1.27288841	0.21558682	5.904296	3.54E-09	9.47E-09	Up	MEIG1
GPR12	1.50981416	-2.75816949	0.46748441	-5.90002454	3.63E-09	9.71E-09	Down	GPR12
TECTA	12.5072007	-0.86090446	0.1459802	-5.8974058	3.69E-09	9.87E-09	Down	TECTA
TRMT10C	1127.82559	0.50099273	0.08500337	5.89379854	3.77E-09	1.01E-08	Up	TRMT10C
NAT14	279.297739	0.82591076	0.14015358	5.89289794	3.79E-09	1.01E-08	Up	NAT14
PTPRC	1073.13608	-1.11415665	0.18907232	-5.8927538	3.80E-09	1.01E-08	Down	PTPRC
DRD4	35.1583505	1.07489939	0.18244043	5.8917829	3.82E-09	1.02E-08	Up	DRD4
CCDC33	5.09878968	1.68291458	0.28565673	5.89138783	3.83E-09	1.02E-08	Up	CCDC33
MAPK8IP3	1880.41898	0.71944516	0.12214126	5.89027134	3.86E-09	1.03E-08	Up	MAPK8IP3
DTX1	141.598416	-0.91880105	0.15602272	-5.88889248	3.89E-09	1.04E-08	Down	DTX1
GJB7	12.4057179	2.78314834	0.47271137	5.88762731	3.92E-09	1.04E-08	Up	GJB7
POTEF	7.8190808	1.59531182	0.27099014	5.88697375	3.93E-09	1.05E-08	Up	POTEF
CDKL3	17.0418794	0.72218391	0.12271862	5.88487638	3.98E-09	1.06E-08	Up	CDKL3
GALNT1	3275.20547	-0.55491336	0.0942984	-5.88465298	3.99E-09	1.06E-08	Down	GALNT1
ADH6	322.524934	-1.28054958	0.21761817	-5.88438718	4.00E-09	1.06E-08	Down	ADH6
FBXL16	260.587383	1.23159912	0.20933564	5.88337039	4.02E-09	1.07E-08	Up	FBXL16
GPR27	25.2578582	-1.58957384	0.27045197	-5.87747195	4.17E-09	1.11E-08	Down	GPR27
SMDT1	1386.06408	-0.52428411	0.08921968	-5.87632781	4.19E-09	1.12E-08	Down	SMDT1
MDP1	34.9283447	-0.59769682	0.10171327	-5.87629138	4.20E-09	1.12E-08	Down	MDP1
NYAP1	18.2467955	-1.12369783	0.19127605	-5.87474411	4.23E-09	1.13E-08	Down	NYAP1
VNN1	385.689995	1.68029075	0.28613206	5.87243086	4.29E-09	1.14E-08	Up	VNN1
ARL4A	1190.48603	-0.59867926	0.10195587	-5.87194493	4.31E-09	1.15E-08	Down	ARL4A
NENF	2080.71716	0.66383286	0.11305687	5.87167197	4.31E-09	1.15E-08	Up	NENF
NCKAP5L	515.676063	0.56835968	0.09684091	5.8690036	4.38E-09	1.17E-08	Up	NCKAP5L
TMED6	58.4349449	-0.83114	0.14162472	-5.8686083	4.39E-09	1.17E-08	Down	TMED6
HHEX	128.070879	-0.87113743	0.14846303	-5.86770595	4.42E-09	1.17E-08	Down	HHEX
ZGLP1	26.6660831	0.7996773	0.13634336	5.86517213	4.49E-09	1.19E-08	Up	ZGLP1
FCHSD1	576.709607	0.50372329	0.08588758	5.86491452	4.49E-09	1.19E-08	Up	FCHSD1
MEGF8	1472.55847	-0.57146352	0.09748173	-5.8622628	4.57E-09	1.21E-08	Down	MEGF8
GOLGA8A	417.735469	1.17245343	0.20001126	5.8619371	4.57E-09	1.21E-08	Up	GOLGA8A
FBXL8	314.49127	0.69429733	0.11845389	5.86133006	4.59E-09	1.22E-08	Up	FBXL8
RPP25	1480.02863	0.6617588	0.11290318	5.86129472	4.59E-09	1.22E-08	Up	RPP25

NGF	11.4391611	-1.14286898	0.1949881	-5.86122418	4.59E-09	1.22E-08	Down	NGF
PAX7	8.56285215	4.42045651	0.75426219	5.86063648	4.61E-09	1.22E-08	Up	PAX7
SYN3	104.405461	1.7434815	0.29755399	5.85937867	4.65E-09	1.23E-08	Up	SYN3
MYLK2	11.0425685	0.80859392	0.13804537	5.85745057	4.70E-09	1.25E-08	Up	MYLK2
NUTM2A	7.50509928	-1.10776727	0.18912477	-5.85733575	4.70E-09	1.25E-08	Down	NUTM2A
CHD5	22.7500281	-1.19161391	0.20345287	-5.85695302	4.71E-09	1.25E-08	Down	CHD5
DPYSL3	2863.98657	-1.04185773	0.17809953	-5.84986248	4.92E-09	1.30E-08	Down	DPYSL3
ATP1A1	53160.3575	-0.54593031	0.09332973	-5.84947903	4.93E-09	1.31E-08	Down	ATP1A1
ALCAM	1843.86358	0.74339221	0.1270979	5.84897321	4.95E-09	1.31E-08	Up	ALCAM
VWCE	56.3814749	1.4034347	0.24001858	5.84719194	5.00E-09	1.32E-08	Up	VWCE
ZDHHC11	79.238833	1.3353138	0.22838369	5.84680007	5.01E-09	1.33E-08	Up	ZDHHC11
CCL16	1.97320039	-1.25629592	0.21488365	-5.84640067	5.02E-09	1.33E-08	Down	CCL16
UNKL	415.450433	0.57584357	0.09850586	5.84577995	5.04E-09	1.33E-08	Up	UNKL
TNNC2	570.589896	1.53936959	0.26344421	5.84324697	5.12E-09	1.35E-08	Up	TNNC2
PSMB3	5124.8699	0.66209398	0.11331048	5.84318391	5.12E-09	1.35E-08	Up	PSMB3
IRS2	2803.69939	0.78510862	0.13444433	5.83965588	5.23E-09	1.38E-08	Up	IRS2
SLC13A5	11.0733589	1.81083193	0.31009965	5.83951624	5.24E-09	1.38E-08	Up	SLC13A5
IFT52	1167.41615	0.56103857	0.09612664	5.83645267	5.33E-09	1.41E-08	Up	IFT52
SWI5	462.305176	0.51679402	0.088565	5.83519474	5.37E-09	1.42E-08	Up	SWI5
KRBA2	10.2300979	-0.79732097	0.13664603	-5.8349368	5.38E-09	1.42E-08	Down	KRBA2
TIMD4	15.5514521	-1.56473713	0.26818567	-5.8345293	5.39E-09	1.42E-08	Down	TIMD4
GIPC2	1255.1579	-0.67913595	0.11640588	-5.83420678	5.40E-09	1.43E-08	Down	GIPC2
ZNF596	69.3470938	-0.65654216	0.11255966	-5.83283735	5.45E-09	1.44E-08	Down	ZNF596
PQBP1	2515.12289	0.61883373	0.10611779	5.83157374	5.49E-09	1.45E-08	Up	PQBP1
PAOX	171.155026	-0.63267257	0.10849099	-5.83156795	5.49E-09	1.45E-08	Down	PAOX
ADARB1	449.655208	-0.66022864	0.11328516	-5.82802423	5.61E-09	1.48E-08	Down	ADARB1
SYNGR4	15.1117534	1.5289489	0.26249729	5.82462734	5.72E-09	1.51E-08	Up	SYNGR4
NRM	589.029908	0.56262362	0.09659882	5.82433231	5.73E-09	1.51E-08	Up	NRM
JAKMIP2	24.5211876	-1.16581271	0.20021124	-5.82291336	5.78E-09	1.52E-08	Down	JAKMIP2
CPVL	666.69375	-0.99245871	0.1704647	-5.82207758	5.81E-09	1.53E-08	Down	CPVL
TMEM17	62.5672874	0.69865286	0.12000689	5.82177269	5.82E-09	1.53E-08	Up	TMEM17
TRIM23	232.712548	-0.54018059	0.09279748	-5.82106954	5.85E-09	1.54E-08	Down	TRIM23
OBSCN	596.270817	0.99698088	0.17127773	5.82084345	5.86E-09	1.54E-08	Up	OBSCN
PRSS50	5.68823323	2.24739437	0.38610499	5.82068208	5.86E-09	1.54E-08	Up	PRSS50
ANKRD22	1190.67482	0.7209717	0.12386486	5.82063143	5.86E-09	1.54E-08	Up	ANKRD22
HRG	2.25523574	3.16040731	0.54317465	5.81839991	5.94E-09	1.56E-08	Up	HRG
FIBCD1	537.245642	1.78003699	0.30597225	5.81764188	5.97E-09	1.57E-08	Up	FIBCD1
SATB2	3805.94956	-0.86365392	0.14854642	-5.81403378	6.10E-09	1.60E-08	Down	SATB2
PHGR1	8742.00505	-1.32660123	0.22818379	-5.8137399	6.11E-09	1.61E-08	Down	PHGR1
SPEF1	6.25096272	1.38522667	0.23838909	5.81078059	6.22E-09	1.64E-08	Up	SPEF1
USHBP1	45.5492035	-0.66703495	0.11480291	-5.81026165	6.24E-09	1.64E-08	Down	USHBP1
TPGS1	249.194222	1.11372489	0.19170091	5.80970054	6.26E-09	1.65E-08	Up	TPGS1
CARTPT	17.5632879	-2.47621096	0.42628903	-5.8087607	6.29E-09	1.65E-08	Down	CARTPT
APOC2	4.64509634	1.35867438	0.23397277	5.8069766	6.36E-09	1.67E-08	Up	APOC2
PRKAR1B	1668.88382	0.65474214	0.11277732	5.80561901	6.41E-09	1.68E-08	Up	PRKAR1B
RGCC	456.682075	0.84321248	0.14531593	5.8026157	6.53E-09	1.71E-08	Up	RGCC
CYTH4	349.729221	-0.90321744	0.1556775	-5.80184971	6.56E-09	1.72E-08	Down	CYTH4
ETV7	482.534818	0.85176216	0.1468118	5.8017282	6.56E-09	1.72E-08	Up	ETV7
HOXB6	1283.13906	1.06421103	0.18345426	5.80096127	6.59E-09	1.73E-08	Up	HOXB6
DDT	832.920392	-0.71401875	0.12310131	-5.80025296	6.62E-09	1.74E-08	Down	DDT
CKB	23887.3169	-1.39966014	0.24144616	-5.79698658	6.75E-09	1.77E-08	Down	CKB
ZNF785	340.921903	0.55970877	0.09655218	5.79695614	6.75E-09	1.77E-08	Up	ZNF785
ZNF391	166.250546	0.78470849	0.13537202	5.79668161	6.76E-09	1.77E-08	Up	ZNF391
HEPH	8976.81008	-0.61636683	0.1063414	-5.79611361	6.79E-09	1.78E-08	Down	HEPH
OPN4	2.41150802	-1.50644734	0.25991604	-5.79589981	6.80E-09	1.78E-08	Down	OPN4
PEBP4	1.99041935	-1.41640388	0.24447179	-5.79373132	6.88E-09	1.80E-08	Down	PEBP4
GEM	784.753052	-0.86618168	0.14952206	-5.79300251	6.91E-09	1.81E-08	Down	GEM
KCNK15	54.7139726	1.60177155	0.27652239	5.79255636	6.93E-09	1.82E-08	Up	KCNK15

TNFAIP6	149.223409	1.27593369	0.22031001	5.79153762	6.97E-09	1.83E-08	Up	TNFAIP6
C16orf86	34.0417585	-0.75141367	0.12974292	-5.79155827	6.97E-09	1.83E-08	Down	C16orf86
STAT4	78.100785	-0.8374095	0.14462251	-5.79031238	7.03E-09	1.84E-08	Down	STAT4
ESR2	23.4511148	-1.01559002	0.17541105	-5.78977231	7.05E-09	1.84E-08	Down	ESR2
SP140	99.7964087	-1.11023429	0.19176863	-5.78944671	7.06E-09	1.85E-08	Down	SP140
RAI1	1349.99295	0.51508348	0.08899479	5.7877937	7.13E-09	1.87E-08	Up	RAI1
KIF17	25.4876736	-0.8616485	0.14887374	-5.78778031	7.13E-09	1.87E-08	Down	KIF17
HLA-E	24422.2626	-0.52449483	0.09062508	-5.78752429	7.14E-09	1.87E-08	Down	HLA-E
PAXBP1	1067.55856	0.50915171	0.087978	5.7872618	7.15E-09	1.87E-08	Up	PAXBP1
SEMG2	2.69122874	4.10957418	0.71024414	5.78614301	7.20E-09	1.88E-08	Up	SEMG2
PIK3R5	191.070646	-0.93522727	0.16172991	-5.78264894	7.35E-09	1.92E-08	Down	PIK3R5
SYT6	3.85800968	-1.81558094	0.31408381	-5.78056193	7.45E-09	1.95E-08	Down	SYT6
ZNF84	729.464924	0.57183031	0.09894501	5.77927375	7.50E-09	1.96E-08	Up	ZNF84
WDR64	1.73597764	-1.64130656	0.28400958	-5.77905353	7.51E-09	1.96E-08	Down	WDR64
FAM180A	30.3366348	-1.31246392	0.22711503	-5.778851	7.52E-09	1.96E-08	Down	FAM180A
THAP10	147.798749	0.53312309	0.09228877	5.7766843	7.62E-09	1.99E-08	Up	THAP10
SLC47A1	49.9242277	-1.11951853	0.19387447	-5.77445054	7.72E-09	2.01E-08	Down	SLC47A1
SCAND1	2875.7436	0.88937558	0.154061	5.77287935	7.79E-09	2.03E-08	Up	SCAND1
PLIN1	107.713732	-1.87329069	0.32451437	-5.772597	7.81E-09	2.04E-08	Down	PLIN1
CNTD1	82.5775456	0.80277941	0.13912636	5.77014585	7.92E-09	2.07E-08	Up	CNTD1
RBP3	5.08139554	2.50583114	0.43432708	5.76945634	7.95E-09	2.07E-08	Up	RBP3
BCAN	110.938878	0.7205984	0.12490128	5.76934369	7.96E-09	2.07E-08	Up	BCAN
CCNA1	3.41158768	-1.62253015	0.28128959	-5.76818419	8.01E-09	2.09E-08	Down	CCNA1
MSC	207.586315	0.81269477	0.14090369	5.76773212	8.03E-09	2.09E-08	Up	MSC
TTLL2	28.6210684	1.03260577	0.17907225	5.76641986	8.10E-09	2.11E-08	Up	TTLL2
RRP7A	2384.68321	0.58151885	0.10085455	5.76591595	8.12E-09	2.11E-08	Up	RRP7A
ZMYM2	2798.18487	0.63243786	0.10968624	5.76588164	8.12E-09	2.11E-08	Up	ZMYM2
LDLRAD1	10.4883512	1.65074807	0.28630179	5.76576226	8.13E-09	2.12E-08	Up	LDLRAD1
STARD13	812.759168	-0.64974819	0.11269613	-5.76548796	8.14E-09	2.12E-08	Down	STARD13
GPR171	37.94073	-1.09646042	0.19018942	-5.76509666	8.16E-09	2.12E-08	Down	GPR171
CENPM	758.059742	0.81446972	0.14133672	5.76261945	8.28E-09	2.15E-08	Up	CENPM
KLK1	1718.7493	-1.13797853	0.19748277	-5.76241931	8.29E-09	2.16E-08	Down	KLK1
GSTO2	911.021767	0.78669537	0.13659841	5.7591839	8.45E-09	2.20E-08	Up	GSTO2
LRRK2	150.668573	-1.32798497	0.23085686	-5.75241712	8.80E-09	2.29E-08	Down	LRRK2
C1orf56	213.82239	0.51070287	0.08878512	5.75212201	8.81E-09	2.29E-08	Up	C1orf56
COL4A5	155.709522	-1.17571663	0.20440144	-5.75199765	8.82E-09	2.29E-08	Down	COL4A5
ADAM23	72.3445481	-1.0422869	0.18126192	-5.75017032	8.92E-09	2.32E-08	Down	ADAM23
CNDP2	6057.08596	-0.50855372	0.08846533	-5.74862158	9.00E-09	2.34E-08	Down	CNDP2
NR5A1	2.91056945	2.6990027	0.46955707	5.74797589	9.03E-09	2.34E-08	Up	NR5A1
SAA2-SAA4	17.3814076	2.33865194	0.40688518	5.74769503	9.05E-09	2.35E-08	Up	SAA2-SAA4
AMHR2	2.07705628	-1.48237362	0.25796471	-5.74642011	9.12E-09	2.37E-08	Down	AMHR2
OTOG	4.50474043	2.08538651	0.36292335	5.74607979	9.13E-09	2.37E-08	Up	OTOG
PLA2G4D	31.6883301	1.31842307	0.2294871	5.74508564	9.19E-09	2.38E-08	Up	PLA2G4D
MRPL45	1856.42609	0.52770252	0.09187477	5.74371517	9.26E-09	2.40E-08	Up	MRPL45
BCAS4	357.417441	0.90427534	0.15744692	5.74336623	9.28E-09	2.41E-08	Up	BCAS4
TMEM205	1822.19328	0.54351357	0.09466222	5.74161036	9.38E-09	2.43E-08	Up	TMEM205
FBXO43	12.3170882	1.00942217	0.17580773	5.74162552	9.38E-09	2.43E-08	Up	FBXO43
VWA1	3412.93672	0.75584915	0.13164581	5.74153613	9.38E-09	2.43E-08	Up	VWA1
DHRS4	538.93315	-0.58985134	0.10274043	-5.74118047	9.40E-09	2.44E-08	Down	DHRS4
ZNF92	393.975003	0.6105263	0.10634548	5.74097065	9.41E-09	2.44E-08	Up	ZNF92
PHYHIP	52.0828605	-1.17581408	0.20483895	-5.74018798	9.46E-09	2.45E-08	Down	PHYHIP
EID2B	22.5392284	-0.72012947	0.12548825	-5.73862066	9.55E-09	2.47E-08	Down	EID2B
FCRL5	149.82532	-1.46658558	0.25557729	-5.73832506	9.56E-09	2.48E-08	Down	FCRL5
DNAH6	78.402619	0.86787771	0.1513094	5.73578189	9.71E-09	2.51E-08	Up	DNAH6
PINX1	290.161777	0.57560411	0.10035331	5.73577601	9.71E-09	2.51E-08	Up	PINX1
SCPEP1	1027.70129	-0.63130066	0.11007046	-5.73542319	9.73E-09	2.52E-08	Down	SCPEP1
ACAP2	1718.37156	-0.5481133	0.09556835	-5.73530162	9.73E-09	2.52E-08	Down	ACAP2
LMBRD2	755.412801	-0.61831174	0.10780933	-5.7352341	9.74E-09	2.52E-08	Down	LMBRD2

DDX47	61.4942829	0.78528065	0.13692227	5.73522953	9.74E-09	2.52E-08	Up	DDX47
PFKFB3	2056.57281	0.54532536	0.0950849	5.73514153	9.74E-09	2.52E-08	Up	PFKFB3
PCYOX1L	396.611968	0.54337658	0.094763	5.73405833	9.81E-09	2.54E-08	Up	PCYOX1L
PHF20L1	1466.1968	0.60228304	0.10506341	5.73256727	9.89E-09	2.56E-08	Up	PHF20L1
FLRT2	201.029712	-1.1603702	0.20246662	-5.73116791	9.97E-09	2.58E-08	Down	FLRT2
PIP5KL1	20.8758226	1.10353913	0.19255675	5.73098122	9.99E-09	2.58E-08	Up	PIP5KL1
CRAT	2378.5795	-0.88660765	0.15475142	-5.72923771	1.01E-08	2.61E-08	Down	CRAT
GUK1	8593.4751	0.71971351	0.12563507	5.72860363	1.01E-08	2.61E-08	Up	GUK1
CD300LD	2.32238606	2.58837579	0.45184111	5.72850881	1.01E-08	2.62E-08	Up	CD300LD
LGALS12	10.2131965	-1.67417734	0.29226025	-5.72837852	1.01E-08	2.62E-08	Down	LGALS12
DAAM1	891.420255	-0.59800589	0.10440123	-5.72795835	1.02E-08	2.62E-08	Down	DAAM1
RIF1	1907.68952	0.64763726	0.11309842	5.72631558	1.03E-08	2.65E-08	Up	RIF1
PI15	143.765416	-1.53870382	0.26871986	-5.72605175	1.03E-08	2.65E-08	Down	PI15
ATP8B4	87.4783027	-0.93565263	0.16342306	-5.72534021	1.03E-08	2.66E-08	Down	ATP8B4
ADD2	29.3980346	-1.50846006	0.26354116	-5.72381199	1.04E-08	2.69E-08	Down	ADD2
CLIC4	3672.67361	-0.77592985	0.13556129	-5.72383039	1.04E-08	2.69E-08	Down	CLIC4
CHKB	105.782752	0.62543921	0.10927415	5.72357889	1.04E-08	2.69E-08	Up	CHKB
OR5M11	0.90818843	2.3985258	0.41907494	5.72338165	1.04E-08	2.69E-08	Up	OR5M11
SMOC1	358.568217	1.73710855	0.30364264	5.72089798	1.06E-08	2.73E-08	Up	SMOC1
REEP1	310.443205	-1.42140189	0.24849002	-5.72015689	1.06E-08	2.74E-08	Down	REEP1
ABHD8	197.295566	-0.67314631	0.11770686	-5.71883651	1.07E-08	2.76E-08	Down	ABHD8
SPTBN4	27.8857073	-1.29710275	0.22681218	-5.71884075	1.07E-08	2.76E-08	Down	SPTBN4
PDE6B	29.0002032	-0.9910989	0.17334775	-5.71740285	1.08E-08	2.78E-08	Down	PDE6B
ODF3B	317.97631	0.91646232	0.16030967	5.71682488	1.09E-08	2.79E-08	Up	ODF3B
CYSTM1	3847.27494	-0.61201678	0.10708524	-5.71522999	1.10E-08	2.82E-08	Down	CYSTM1
IPCEF1	58.5400973	-0.93813112	0.16416576	-5.71453571	1.10E-08	2.83E-08	Down	IPCEF1
OR2L13	0.4237172	-2.66652898	0.46680027	-5.7123553	1.11E-08	2.87E-08	Down	OR2L13
CLHC1	94.1266084	0.73204262	0.12818016	5.7110447	1.12E-08	2.89E-08	Up	CLHC1
FCGRT	9414.27209	-0.80592027	0.14113684	-5.7102047	1.13E-08	2.90E-08	Down	FCGRT
FOXO6	51.0224845	1.02642592	0.17975833	5.71003248	1.13E-08	2.90E-08	Up	FOXO6
CCNE2	188.42725	0.6196857	0.10856235	5.70810883	1.14E-08	2.94E-08	Up	CCNE2
GSAP	476.615951	0.62113561	0.10885196	5.70624167	1.15E-08	2.97E-08	Up	GSAP
LCP1	2571.71474	-0.94416086	0.16546495	-5.70610799	1.16E-08	2.97E-08	Down	LCP1
GFOD1	382.386239	-0.60697289	0.10638679	-5.7053406	1.16E-08	2.98E-08	Down	GFOD1
SAT2	971.748512	-0.52256995	0.09161558	-5.70394219	1.17E-08	3.01E-08	Down	SAT2
CDSN	2.82909011	1.73833439	0.30476464	5.70385852	1.17E-08	3.01E-08	Up	CDSN
ZBTB25	400.981096	0.55674732	0.09761574	5.70345866	1.17E-08	3.02E-08	Up	ZBTB25
MCAT	835.07254	0.53955317	0.09460078	5.70347477	1.17E-08	3.02E-08	Up	MCAT
LPIN3	1290.18246	0.67813504	0.11891297	5.70278433	1.18E-08	3.03E-08	Up	LPIN3
BSPRY	1773.93914	0.5905822	0.10358304	5.7015339	1.19E-08	3.05E-08	Up	BSPRY
ATRIP	6.27227089	0.96409333	0.1691291	5.70033972	1.20E-08	3.07E-08	Up	ATRIP
CRYBA2	20.2903666	-1.76804226	0.31024074	-5.6989364	1.21E-08	3.09E-08	Down	CRYBA2
DAPL1	28.160743	1.70331729	0.29889574	5.69870038	1.21E-08	3.10E-08	Up	DAPL1
HEY1	167.767957	-0.57773331	0.10138163	-5.69859935	1.21E-08	3.10E-08	Down	HEY1
JMJD7-PLA2(	119.619116	0.77299319	0.13567039	5.69758201	1.22E-08	3.12E-08	Up	JMJD7-PLA2G4B
KCNMB2	6.26408071	-1.66573571	0.29239495	-5.69686886	1.22E-08	3.13E-08	Down	KCNMB2
TMEM151A	30.2738607	1.61464902	0.2834365	5.69668702	1.22E-08	3.13E-08	Up	TMEM151A
ZNF350	226.904945	-0.71197013	0.12500148	-5.69569366	1.23E-08	3.15E-08	Down	ZNF350
RGL3	218.759525	1.32072593	0.23188736	5.69554944	1.23E-08	3.15E-08	Up	RGL3
HOXA9	1037.66772	1.02248154	0.1795623	5.69429973	1.24E-08	3.17E-08	Up	HOXA9
RNF151	1.33371923	1.97429149	0.34675216	5.69366739	1.24E-08	3.19E-08	Up	RNF151
ZNF829	36.7868308	-1.11227617	0.19537579	-5.69300918	1.25E-08	3.20E-08	Down	ZNF829
PPP1R27	1.97399941	1.92091717	0.33747235	5.69207283	1.26E-08	3.21E-08	Up	PPP1R27
IGIP	287.534415	-0.54729129	0.09620489	-5.68880921	1.28E-08	3.27E-08	Down	IGIP
LPGAT1	2907.89121	0.57550233	0.10116611	5.68868688	1.28E-08	3.28E-08	Up	LPGAT1
FBXO27	32.3587729	-1.22507839	0.21538668	-5.68780929	1.29E-08	3.29E-08	Down	FBXO27
QTRT1	2379.39709	0.78772599	0.13849873	5.68760422	1.29E-08	3.29E-08	Up	QTRT1
ROMO1	3633.58391	0.9350591	0.16446808	5.68535308	1.31E-08	3.34E-08	Up	ROMO1

ZNF185	988.385961	0.9140781	0.16078557	5.68507543	1.31E-08	3.34E-08	Up		ZNF185
REEP3	1670.47501	-0.56405837	0.09922567	-5.68460153	1.31E-08	3.35E-08	Down		REEP3
CCDC28B	175.176064	0.56742175	0.09983229	5.68374948	1.32E-08	3.37E-08	Up		CCDC28B
ACOT2	562.648352	-0.57693286	0.10150669	-5.68369298	1.32E-08	3.37E-08	Down		ACOT2
TMEM154	364.131774	0.79952332	0.14066989	5.68368498	1.32E-08	3.37E-08	Up		TMEM154
NBPF8	51.3392029	0.57112159	0.10048511	5.68364414	1.32E-08	3.37E-08	Up		NBPF8
CLTCL1	170.077883	0.79694757	0.14023858	5.6827982	1.33E-08	3.38E-08	Up		CLTCL1
HOXB13	1432.09626	-1.50109982	0.26426309	-5.68032334	1.34E-08	3.43E-08	Down		HOXB13
CD28	73.5444681	-0.95468258	0.16807614	-5.68006022	1.35E-08	3.44E-08	Down		CD28
MYO9A	735.147894	-0.63128577	0.11116191	-5.67897541	1.36E-08	3.46E-08	Down		MYO9A
FLII	5255.52677	-0.50534232	0.08899154	-5.67854303	1.36E-08	3.47E-08	Down		FLII
ZNF385D	28.1966703	-1.34429796	0.23678751	-5.67723347	1.37E-08	3.49E-08	Down		ZNF385D
C2orf81	152.999468	0.67914614	0.11962775	5.67716205	1.37E-08	3.49E-08	Up		C2orf81
AXIN1	2901.06888	0.54063465	0.09530184	5.67286696	1.40E-08	3.58E-08	Up		AXIN1
ZBED2	35.7912623	1.44829568	0.25531336	5.67262002	1.41E-08	3.59E-08	Up		ZBED2
PODN	694.551425	-1.04595419	0.18442144	-5.67154345	1.42E-08	3.61E-08	Down		PODN
ALDH1B1	5673.53959	0.62705882	0.11056451	5.67142924	1.42E-08	3.61E-08	Up		ALDH1B1
MUC15	4.95976018	3.80156406	0.67030916	5.67135924	1.42E-08	3.61E-08	Up		MUC15
PDE10A	137.080327	1.25172825	0.22073922	5.67062012	1.42E-08	3.63E-08	Up		PDE10A
KCNA10	1.16578731	2.5117565	0.44308321	5.66881444	1.44E-08	3.66E-08	Up		KCNA10
SAMD14	75.6592958	0.66772131	0.11780066	5.66823059	1.44E-08	3.68E-08	Up		SAMD14
KRTCAP3	2592.28676	0.66816023	0.1179027	5.66704758	1.45E-08	3.70E-08	Up		KRTCAP3
FGL1	15.0874738	3.3575541	0.59252596	5.66650972	1.46E-08	3.71E-08	Up		FGL1
RSPO3	133.665684	-1.37800382	0.24327855	-5.66430474	1.48E-08	3.76E-08	Down		RSPO3
CCDC24	446.569602	0.62506932	0.11035999	5.66391256	1.48E-08	3.77E-08	Up		CCDC24
FGF16	0.52640655	-2.11865636	0.37413522	-5.66280919	1.49E-08	3.79E-08	Down		FGF16
KRT74	1.78695479	3.4245175	0.60492181	5.66109109	1.50E-08	3.83E-08	Up		KRT74
GFY	1.61801078	3.06990899	0.54243926	5.65945203	1.52E-08	3.86E-08	Up		GFY
SVOPL	19.2009615	-2.04993562	0.3623071	-5.65800564	1.53E-08	3.90E-08	Down		SVOPL
TBC1D8	662.55803	0.56575889	0.09999973	5.65760394	1.54E-08	3.90E-08	Up		TBC1D8
ZNF287	56.0783376	-0.81172169	0.14348173	-5.65731761	1.54E-08	3.91E-08	Down		ZNF287
TPPP2	2.27045769	-1.42176223	0.25136364	-5.65619688	1.55E-08	3.93E-08	Down		TPPP2
COL26A1	7.79467261	1.47589414	0.2609369	5.65613425	1.55E-08	3.94E-08	Up		COL26A1
TAF13	687.645478	-0.51482301	0.09103421	-5.65526941	1.56E-08	3.95E-08	Down		TAF13
MRPL52	1746.90088	0.52187622	0.09233127	5.6522152	1.58E-08	4.02E-08	Up		MRPL52
COMMD7	2423.67154	0.57982283	0.10259406	5.65162193	1.59E-08	4.04E-08	Up		COMMD7
CCDC121	96.9466636	-0.54345287	0.09616004	-5.65154566	1.59E-08	4.04E-08	Down		CCDC121
VAX1	6.93938293	3.70599865	0.65601323	5.64927422	1.61E-08	4.09E-08	Up		VAX1
ESF1	803.201856	0.69338501	0.12278771	5.64702275	1.63E-08	4.14E-08	Up		ESF1
ZSWIM8	1279.96957	-0.5258104	0.09312836	-5.64608248	1.64E-08	4.16E-08	Down		ZSWIM8
OCA2	58.2666491	1.73402939	0.30720361	5.64456066	1.66E-08	4.20E-08	Up		OCA2
ALS2CL	1458.3191	0.70056988	0.12413472	5.64362541	1.67E-08	4.22E-08	Up		ALS2CL
TLR5	95.1871784	-0.80885849	0.1434702	-5.63781542	1.72E-08	4.37E-08	Down		TLR5
EN1	4.42170361	2.81323561	0.49904803	5.63720416	1.73E-08	4.38E-08	Up		EN1
GABRB1	12.1134038	1.7626768	0.3127234	5.63653634	1.74E-08	4.40E-08	Up		GABRB1
MXRA5	3122.62182	1.07966594	0.19159429	5.63516769	1.75E-08	4.43E-08	Up		MXRA5
SLC38A4	187.699751	-1.56528345	0.27783217	-5.63391716	1.76E-08	4.46E-08	Down		SLC38A4
DOHH	696.346236	0.65213088	0.11581137	5.63097431	1.79E-08	4.54E-08	Up		DOHH
UCN3	51.8625125	-1.82387116	0.32402321	-5.62882876	1.81E-08	4.59E-08	Down		UCN3
ITLN2	32.046318	2.37615291	0.42219299	5.62812025	1.82E-08	4.61E-08	Up		ITLN2
IPMK	665.772739	-0.62823662	0.11163051	-5.6278219	1.82E-08	4.62E-08	Down		IPMK
MEF2B	15.4042468	-0.80541082	0.14311969	-5.62753321	1.83E-08	4.63E-08	Down		MEF2B
MTURN	918.81088	-0.63653283	0.11312028	-5.62704424	1.83E-08	4.64E-08	Down		MTURN
HPCA	34.061001	-0.93347506	0.16590144	-5.62668435	1.84E-08	4.65E-08	Down		HPCA
EVA1B	327.643	0.89240366	0.15864264	5.62524473	1.85E-08	4.69E-08	Up		EVA1B
AQP4	2.34227669	-2.51901213	0.44781378	-5.62513318	1.85E-08	4.69E-08	Down		AQP4
PTPN13	667.341805	1.13043381	0.20105076	5.62262891	1.88E-08	4.75E-08	Up		PTPN13
USP44	10.5881628	-1.12898233	0.20079701	-5.62250575	1.88E-08	4.76E-08	Down		USP44

TMEM240	22.1853137	-0.81522022	0.14501924	-5.62146248	1.89E-08	4.78E-08	Down	TMEM240
NAIP	21.8275506	-1.01326604	0.18025304	-5.62135357	1.89E-08	4.79E-08	Down	NAIP
ADAL	169.580457	-0.54065087	0.09618302	-5.62106378	1.90E-08	4.79E-08	Down	ADAL
HCK	514.03517	-0.87502958	0.15571265	-5.61951519	1.91E-08	4.84E-08	Down	HCK
LRP3	1406.05577	0.93677332	0.16671574	5.61898533	1.92E-08	4.85E-08	Up	LRP3
OPRD1	44.3035666	1.71005706	0.30439216	5.6179406	1.93E-08	4.88E-08	Up	OPRD1
DALRD3	810.110941	0.56275873	0.10017213	5.61791742	1.93E-08	4.88E-08	Up	DALRD3
NAT8B	46.5247193	-1.81422753	0.32301996	-5.61645647	1.95E-08	4.92E-08	Down	NAT8B
WNT3A	2.33085057	1.55391139	0.27671505	5.61556509	1.96E-08	4.94E-08	Up	WNT3A
XKR5	3.44923258	1.33093574	0.23701295	5.61545571	1.96E-08	4.95E-08	Up	XKR5
FFAR1	0.7419861	-1.95451619	0.34810367	-5.61475312	1.97E-08	4.97E-08	Down	FFAR1
NSMCE2	740.870662	0.55415489	0.09871791	5.6135192	1.98E-08	5.00E-08	Up	NSMCE2
HIPK3	2256.98017	-0.54135642	0.09656483	-5.60614461	2.07E-08	5.22E-08	Down	HIPK3
ZNF726	16.6030235	-1.32828339	0.23693625	-5.60607935	2.07E-08	5.22E-08	Down	ZNF726
COL6A6	12.4056571	1.76142268	0.31420765	5.60591919	2.07E-08	5.22E-08	Up	COL6A6
QRFP	17.9501958	-0.81446914	0.14529838	-5.60549379	2.08E-08	5.23E-08	Down	QRFP
CKM	16.4163128	-1.36258104	0.24308211	-5.60543537	2.08E-08	5.24E-08	Down	CKM
POLRMT	2408.28413	0.62212724	0.11099576	5.6049641	2.08E-08	5.25E-08	Up	POLRMT
E2F2	584.996448	-0.62683405	0.11183911	-5.60478368	2.09E-08	5.25E-08	Down	E2F2
LYN	2214.42644	0.53883477	0.09614978	5.60411849	2.09E-08	5.27E-08	Up	LYN
SLC24A2	19.2919897	1.53102295	0.27322351	5.60355494	2.10E-08	5.29E-08	Up	SLC24A2
LRRC74A	4.83820622	1.63926601	0.29254387	5.60348777	2.10E-08	5.29E-08	Up	LRRC74A
ANKRD36C	70.4437337	1.13150927	0.201952	5.6028624	2.11E-08	5.31E-08	Up	ANKRD36C
CETN2	1599.14196	0.52676728	0.09402221	5.60258339	2.11E-08	5.32E-08	Up	CETN2
ABCC9	141.800519	-1.16802992	0.20852603	-5.60136271	2.13E-08	5.35E-08	Down	ABCC9
GAB3	89.8342281	-0.74493508	0.13301741	-5.6002827	2.14E-08	5.38E-08	Down	GAB3
CD163	908.195308	-1.19333117	0.21309785	-5.59992109	2.14E-08	5.39E-08	Down	CD163
ITGA5	2748.58444	-0.9245917	0.16511278	-5.59975854	2.15E-08	5.40E-08	Down	ITGA5
ZDHHC20	4926.88684	0.53730238	0.09595258	5.59966558	2.15E-08	5.40E-08	Up	ZDHHC20
MT1HL1	0.46152062	-2.30478024	0.41166003	-5.59874668	2.16E-08	5.43E-08	Down	MT1HL1
NR6A1	394.171028	0.65383111	0.1168011	5.59781649	2.17E-08	5.46E-08	Up	NR6A1
ZBED8	151.930528	0.57621554	0.10293999	5.59758706	2.17E-08	5.46E-08	Up	ZBED8
WFDC13	4.68747498	1.75271139	0.31321384	5.5958938	2.19E-08	5.52E-08	Up	WFDC13
TNRC18	7904.41426	0.53828426	0.09620154	5.59538112	2.20E-08	5.53E-08	Up	TNRC18
GPR87	1.6473603	3.05306457	0.54567016	5.59507337	2.21E-08	5.54E-08	Up	GPR87
SFMBT2	171.929651	-0.9407716	0.16815929	-5.59452663	2.21E-08	5.56E-08	Down	SFMBT2
SRXN1	81.4020869	0.73197304	0.1308537	5.59382767	2.22E-08	5.58E-08	Up	SRXN1
EMILIN1	3192.53328	-0.90540932	0.16186917	-5.59346376	2.23E-08	5.59E-08	Down	EMILIN1
FMO1	29.4030443	1.11700209	0.19972113	5.59280871	2.23E-08	5.61E-08	Up	FMO1
ARAP3	758.30442	0.70564617	0.12618813	5.5920171	2.24E-08	5.63E-08	Up	ARAP3
SLCO2B1	2270.05188	-0.63340662	0.11327451	-5.59178434	2.25E-08	5.64E-08	Down	SLCO2B1
HYDIN	7.98337152	1.9844112	0.35490273	5.59142279	2.25E-08	5.65E-08	Up	HYDIN
HCST	73.8363625	-0.96824244	0.17319008	-5.59063442	2.26E-08	5.68E-08	Down	HCST
POSTN	6004.04811	1.24496636	0.22270134	5.59029587	2.27E-08	5.69E-08	Up	POSTN
ZNF66	38.7188971	1.05332674	0.18842884	5.59005059	2.27E-08	5.69E-08	Up	ZNF66
NPPC	4.66835674	-1.7394164	0.31129701	-5.58764253	2.30E-08	5.77E-08	Down	NPPC
IL1RAPL1	2.36924938	-1.79747719	0.32169039	-5.58759982	2.30E-08	5.77E-08	Down	IL1RAPL1
NDUFAF2	629.65052	0.52843281	0.094575	5.58744693	2.30E-08	5.77E-08	Up	NDUFAF2
GOLGA8N	12.5906856	-1.15180973	0.20618085	-5.58640495	2.32E-08	5.81E-08	Down	GOLGA8N
HKDC1	2724.16418	0.74841932	0.1339807	5.58602335	2.32E-08	5.82E-08	Up	HKDC1
KCND3	202.217279	-1.18644076	0.21248533	-5.58363604	2.36E-08	5.90E-08	Down	KCND3
ST3GAL4	1662.31217	-1.15453182	0.20679655	-5.58293567	2.36E-08	5.92E-08	Down	ST3GAL4
KRBA1	80.7236293	-0.97065023	0.17391017	-5.58133093	2.39E-08	5.98E-08	Down	KRBA1
RNF144A	574.253576	-0.72985972	0.13084119	-5.57821058	2.43E-08	6.08E-08	Down	RNF144A
TMEM68	944.183365	0.54316811	0.09737988	5.57782703	2.44E-08	6.09E-08	Up	TMEM68
PLA2G2D	82.687709	-1.62136189	0.29081169	-5.57529816	2.47E-08	6.18E-08	Down	PLA2G2D
TTC39C	346.364643	0.56692795	0.10169481	5.57479752	2.48E-08	6.20E-08	Up	TTC39C
NPIPB11	47.2995402	1.01340994	0.18178583	5.57474672	2.48E-08	6.20E-08	Up	NPIPB11

LYRM7	695.072897	-0.51555794	0.09250383	-5.57336848	2.50E-08	6.25E-08	Down	LYRM7
OSCAR	116.795723	0.90692551	0.16272588	5.57333311	2.50E-08	6.25E-08	Up	OSCAR
SLC16A4	251.94656	0.89088421	0.15990843	5.57121463	2.53E-08	6.32E-08	Up	SLC16A4
POMK	84.0205391	0.92323203	0.1657146	5.57121733	2.53E-08	6.32E-08	Up	POMK
SPDYE5	16.6365645	1.12771985	0.20246032	5.57007848	2.55E-08	6.36E-08	Up	SPDYE5
FGF9	36.7817005	-1.39697233	0.25082009	-5.56961905	2.55E-08	6.38E-08	Down	FGF9
ALG1L2	18.2262062	0.83248735	0.1495059	5.56825766	2.57E-08	6.43E-08	Up	ALG1L2
PNPLA7	279.389469	-0.87994153	0.15807388	-5.56664731	2.60E-08	6.49E-08	Down	PNPLA7
TLX2	11.6258974	-1.24554735	0.2237684	-5.5662343	2.60E-08	6.50E-08	Down	TLX2
FAM193B	1012.3655	0.53288311	0.0957445	5.56567857	2.61E-08	6.52E-08	Up	FAM193B
POU3F3	8.15445858	-2.01164033	0.3614948	-5.5647836	2.62E-08	6.55E-08	Down	POU3F3
SNRPN	472.189483	-0.99720538	0.17922717	-5.56391865	2.64E-08	6.58E-08	Down	SNRPN
ABO	2765.2538	-0.85021366	0.15281026	-5.56385201	2.64E-08	6.58E-08	Down	ABO
MMP25	124.391433	-0.92619056	0.16654154	-5.56131867	2.68E-08	6.68E-08	Down	MMP25
SRD5A2	1.9302021	2.25747146	0.40595556	5.56088327	2.68E-08	6.70E-08	Up	SRD5A2
GSS	4182.59783	0.52913974	0.0951682	5.56004786	2.70E-08	6.73E-08	Up	GSS
FGF21	0.85428258	2.48629929	0.44723289	5.55929443	2.71E-08	6.76E-08	Up	FGF21
RBPMS	1486.9988	-0.66921208	0.12038926	-5.55873552	2.72E-08	6.78E-08	Down	RBPMS
TYR	3.32030558	3.92528262	0.70668746	5.55448181	2.78E-08	6.94E-08	Up	TYR
ANKRD31	6.31954408	1.25338083	0.22566989	5.55404553	2.79E-08	6.95E-08	Up	ANKRD31
PTGES	652.114645	0.9761523	0.17575515	5.55404654	2.79E-08	6.95E-08	Up	PTGES
DYRK3	100.412513	0.68526908	0.12340121	5.55317971	2.81E-08	6.99E-08	Up	DYRK3
CEACAM6	47019.8493	0.95873056	0.17267687	5.55216544	2.82E-08	7.03E-08	Up	CEACAM6
PARD6G	137.166261	-0.64310856	0.1158305	-5.55215209	2.82E-08	7.03E-08	Down	PARD6G
SH3BP5	150.156945	-0.70629202	0.12724584	-5.55061008	2.85E-08	7.09E-08	Down	SH3BP5
MOSPD3	631.306671	0.58454482	0.10532205	5.55007065	2.86E-08	7.11E-08	Up	MOSPD3
DGKH	1251.48584	0.91344	0.16458792	5.5498607	2.86E-08	7.12E-08	Up	DGKH
ZNF432	406.442399	0.57234165	0.1031405	5.54914536	2.87E-08	7.14E-08	Up	ZNF432
ZMYM5	452.736676	0.5337149	0.09618602	5.54877821	2.88E-08	7.16E-08	Up	ZMYM5
C11orf65	17.6201844	1.08579977	0.19568866	5.54860844	2.88E-08	7.16E-08	Up	C11orf65
CHML	621.558375	0.69764601	0.12573349	5.54860929	2.88E-08	7.16E-08	Up	CHML
SH3TC1	570.927122	0.59125413	0.10657052	5.54800849	2.89E-08	7.19E-08	Up	SH3TC1
ZNF138	264.651654	0.53256508	0.0960208	5.54635122	2.92E-08	7.25E-08	Up	ZNF138
HSPA1B	4399.77736	0.70692277	0.12752406	5.54344643	2.97E-08	7.37E-08	Up	HSPA1B
MBNL3	1046.31252	-0.65583051	0.11830797	-5.54341786	2.97E-08	7.37E-08	Down	MBNL3
ABCA1	944.397001	-0.85448087	0.15415472	-5.54300826	2.97E-08	7.39E-08	Down	ABCA1
KLRC1	11.951383	-1.31201501	0.23681408	-5.54027438	3.02E-08	7.50E-08	Down	KLRC1
FGF12	19.4531883	-0.99788892	0.18015115	-5.53917583	3.04E-08	7.55E-08	Down	FGF12
COLEC10	5.96477721	1.06592096	0.19246921	5.53813745	3.06E-08	7.59E-08	Up	COLEC10
CYP2C8	7.64471777	-1.15264358	0.20814213	-5.53777154	3.06E-08	7.60E-08	Down	CYP2C8
MPC2	2635.66949	-0.50563958	0.09131531	-5.53729248	3.07E-08	7.62E-08	Down	MPC2
RBP4	606.464638	1.40467026	0.2537339	5.53599765	3.09E-08	7.67E-08	Up	RBP4
SLC39A4	4206.56751	0.89821935	0.16224974	5.53602947	3.09E-08	7.67E-08	Up	SLC39A4
C14orf180	4.67139673	-2.99710304	0.54157894	-5.53400953	3.13E-08	7.76E-08	Down	C14orf180
TULP4	1274.11135	0.55061491	0.09951959	5.53272907	3.15E-08	7.81E-08	Up	TULP4
BIK	729.238305	0.79411805	0.14353787	5.53246349	3.16E-08	7.82E-08	Up	BIK
ABCG5	27.7194186	1.39405743	0.25212322	5.52927024	3.22E-08	7.96E-08	Up	ABCG5
HOXB3	1293.9434	0.85958523	0.15552708	5.52691669	3.26E-08	8.07E-08	Up	HOXB3
SPATA31C2	0.81608851	-1.62462019	0.29397455	-5.5263973	3.27E-08	8.09E-08	Down	SPATA31C2
NEU1	4301.08394	0.64831392	0.11733193	5.52546903	3.29E-08	8.13E-08	Up	NEU1
LAT2	296.524105	-0.66609825	0.12056094	-5.52499226	3.30E-08	8.15E-08	Down	LAT2
NDUFA1	3394.4251	-0.50278254	0.09102793	-5.52338795	3.33E-08	8.22E-08	Down	NDUFA1
MMD2	0.51770505	-2.4196176	0.43814488	-5.52241447	3.34E-08	8.27E-08	Down	MMD2
NOTCH4	464.78444	0.59418428	0.10762974	5.5206329	3.38E-08	8.35E-08	Up	NOTCH4
SLC13A1	2.49827065	-3.38439124	0.61308521	-5.52026242	3.38E-08	8.36E-08	Down	SLC13A1
FCGR2B	133.703474	-1.15391899	0.20910061	-5.51848695	3.42E-08	8.45E-08	Down	FCGR2B
DDX25	2.72963605	-1.6131957	0.29250114	-5.51517756	3.48E-08	8.61E-08	Down	DDX25
SERHL2	52.4967682	0.5055026	0.09170732	5.51212918	3.55E-08	8.75E-08	Up	SERHL2

NECAB2	6.08131234	-1.42053493	0.25778864	-5.51046356	3.58E-08	8.84E-08	Down	NECAB2
THSD7A	73.2203582	-1.12364734	0.20391295	-5.5104266	3.58E-08	8.84E-08	Down	THSD7A
WDR76	472.21978	0.54114632	0.09823183	5.50886956	3.61E-08	8.91E-08	Up	WDR76
UPP1	923.905701	-0.65461382	0.11883042	-5.50880687	3.61E-08	8.91E-08	Down	UPP1
SLC27A4	2338.64985	-0.5583366	0.10135859	-5.50852768	3.62E-08	8.93E-08	Down	SLC27A4
ST8SIA6	30.2799241	-1.28179263	0.23286616	-5.50441781	3.70E-08	9.13E-08	Down	ST8SIA6
YIF1A	3477.28009	0.65000021	0.11808778	5.50438159	3.70E-08	9.14E-08	Up	YIF1A
OXGR1	201.167345	1.32848359	0.24139473	5.50336617	3.73E-08	9.19E-08	Up	OXGR1
ECM1	1507.6924	0.83010517	0.15089649	5.50115617	3.77E-08	9.30E-08	Up	ECM1
CCDC184	31.5178259	-0.8298379	0.15086038	-5.50070152	3.78E-08	9.32E-08	Down	CCDC184
SNX10	431.730967	0.80801678	0.14695161	5.4985228	3.83E-08	9.44E-08	Up	SNX10
KRT40	87.0651726	2.147147	0.39053587	5.49795074	3.84E-08	9.46E-08	Up	KRT40
ISG20	783.750013	-0.77959566	0.14181292	-5.49735283	3.86E-08	9.50E-08	Down	ISG20
CCDC14	1699.63149	0.66888809	0.12170348	5.49604727	3.88E-08	9.56E-08	Up	CCDC14
SPATA21	2.56935519	2.18595339	0.39779636	5.49515684	3.90E-08	9.61E-08	Up	SPATA21
CDK16	4310.88006	0.51106406	0.09301932	5.49417116	3.93E-08	9.66E-08	Up	CDK16
SLC22A12	1.52625423	2.9585868	0.53852484	5.49387252	3.93E-08	9.68E-08	Up	SLC22A12
LRRC58	1676.3456	0.54916779	0.09999001	5.49222667	3.97E-08	9.76E-08	Up	LRRC58
KCNB2	3.16769966	-1.66904154	0.30390537	-5.49197785	3.97E-08	9.78E-08	Down	KCNB2
CCDC154	16.5972361	1.13706529	0.20706163	5.49143412	3.99E-08	9.80E-08	Up	CCDC154
SPATA5	349.897766	0.59264605	0.10795064	5.4899727	4.02E-08	9.88E-08	Up	SPATA5
ITIH2	12.684767	1.65412558	0.30145638	5.48711416	4.09E-08	1.00E-07	Up	ITIH2
IL1R1	1076.5381	-0.83256099	0.15174523	-5.48657088	4.10E-08	1.01E-07	Down	IL1R1
TSSK6	71.9411072	0.66748513	0.12169074	5.4850937	4.13E-08	1.02E-07	Up	TSSK6
SH2B2	194.004174	0.77844436	0.14199142	5.48233394	4.20E-08	1.03E-07	Up	SH2B2
DENND3	898.715156	-0.52440608	0.09566191	-5.48186924	4.21E-08	1.03E-07	Down	DENND3
TMPRSS11E	8.82012722	2.74333135	0.50045533	5.48167079	4.21E-08	1.03E-07	Up	TMPRSS11E
RNF113B	1.56113855	3.3801775	0.61672634	5.48083855	4.23E-08	1.04E-07	Up	RNF113B
DQX1	380.296832	-0.98076479	0.17901544	-5.47866025	4.29E-08	1.05E-07	Down	DQX1
CCDC175	13.7926494	1.31988797	0.2409383	5.478116	4.30E-08	1.05E-07	Up	CCDC175
ITLN1	3592.53773	-2.10549891	0.38437789	-5.47767948	4.31E-08	1.06E-07	Down	ITLN1
SLC28A2	376.7821	-1.91358357	0.3494356	-5.47621245	4.35E-08	1.07E-07	Down	SLC28A2
KLHL38	6.87979862	-1.25013732	0.22838384	-5.47384321	4.40E-08	1.08E-07	Down	KLHL38
CRP	5.38054716	4.4324226	0.8097607	5.47374381	4.41E-08	1.08E-07	Up	CRP
POLR2F	3.86000017	-1.11541668	0.2037993	-5.47311353	4.42E-08	1.08E-07	Down	POLR2F
TSPAN8	23877.5638	-0.7127768	0.13025637	-5.47210687	4.45E-08	1.09E-07	Down	TSPAN8
MCAM	2643.263	-0.66712417	0.12196159	-5.46995288	4.50E-08	1.10E-07	Down	MCAM
DCDC1	3.87931455	1.94609678	0.35591353	5.46789206	4.55E-08	1.12E-07	Up	DCDC1
PALMD	150.28598	-0.96666278	0.17698486	-5.46183874	4.71E-08	1.15E-07	Down	PALMD
CADM1	281.883083	-1.003783	0.18382204	-5.4606239	4.74E-08	1.16E-07	Down	CADM1
ADAM19	1446.7112	0.75776984	0.13882378	5.45850188	4.80E-08	1.18E-07	Up	ADAM19
IL1RL2	86.9383001	1.06532628	0.19517797	5.45823004	4.81E-08	1.18E-07	Up	IL1RL2
KRTAP4-1	26.5624143	1.1604498	0.21270584	5.45565547	4.88E-08	1.19E-07	Up	KRTAP4-1
KIAA1109	1805.03909	-0.65456916	0.11998526	-5.45541303	4.89E-08	1.20E-07	Down	KIAA1109
CHD3	3169.49896	-0.54802651	0.10049218	-5.45342453	4.94E-08	1.21E-07	Down	CHD3
AOC2	19.0615265	0.907353	0.16638343	5.45338544	4.94E-08	1.21E-07	Up	AOC2
EFCAB5	25.6796348	-0.81072729	0.14871035	-5.45172058	4.99E-08	1.22E-07	Down	EFCAB5
AKAP14	3.02652097	1.82745682	0.3352327	5.4513083	5.00E-08	1.22E-07	Up	AKAP14
PPIL6	83.0853777	0.65666426	0.12053701	5.44782284	5.10E-08	1.25E-07	Up	PPIL6
CCT6B	63.8623266	0.67339736	0.12362529	5.44708423	5.12E-08	1.25E-07	Up	CCT6B
TFF2	201.242543	1.79104523	0.3290225	5.44353426	5.22E-08	1.28E-07	Up	TFF2
ZNF597	204.895033	0.54410855	0.09995734	5.4434079	5.23E-08	1.28E-07	Up	ZNF597
RORB	10.4592525	-1.55250718	0.2852415	-5.44278158	5.25E-08	1.28E-07	Down	RORB
ANO1	1241.87924	1.04201737	0.19146357	5.44237932	5.26E-08	1.28E-07	Up	ANO1
FOXC2	35.4234065	1.32418917	0.24335989	5.44127949	5.29E-08	1.29E-07	Up	FOXC2
GPX4	6379.23561	0.64891862	0.11931686	5.43861645	5.37E-08	1.31E-07	Up	GPX4
FANCM	187.520143	0.56851805	0.10453811	5.43838071	5.38E-08	1.31E-07	Up	FANCM
QPRT	2909.54049	1.10009453	0.20230638	5.43776491	5.40E-08	1.32E-07	Up	QPRT

ENTPD2	1242.65415	0.84611342	0.15561777	5.43712592	5.41E-08	1.32E-07	Up	ENTPD2
MYBPH	2.40367039	1.70699857	0.31397588	5.4367189	5.43E-08	1.32E-07	Up	MYBPH
ZNF23	40.5021179	0.60851833	0.11193315	5.43644402	5.44E-08	1.33E-07	Up	ZNF23
EPB41L2	6777.75148	0.58312162	0.1072628	5.43638261	5.44E-08	1.33E-07	Up	EPB41L2
NBPF10	29.1990564	-0.58586884	0.10776921	-5.43632861	5.44E-08	1.33E-07	Down	NBPF10
C6	16.3661893	-1.59257191	0.2931287	-5.43301265	5.54E-08	1.35E-07	Down	C6
ABHD11	3392.86038	0.54611032	0.10055915	5.43073708	5.61E-08	1.37E-07	Up	ABHD11
AHNAK2	1235.18081	-1.20148527	0.22126655	-5.43003573	5.63E-08	1.37E-07	Down	AHNAK2
LRRC23	199.48553	0.50862588	0.09367694	5.42957386	5.65E-08	1.38E-07	Up	LRRC23
TMEM63C	309.05641	-1.20762956	0.222681	-5.42313687	5.86E-08	1.43E-07	Down	TMEM63C
LGMN	4608.93083	-0.51432281	0.09485489	-5.42220663	5.89E-08	1.43E-07	Down	LGMN
SHD	180.852991	-0.91596137	0.16896494	-5.42101448	5.93E-08	1.44E-07	Down	SHD
TREM2	192.043267	1.13210904	0.20887995	5.41990282	5.96E-08	1.45E-07	Up	TREM2
RIBC1	35.5904079	0.69892384	0.12900366	5.41786064	6.03E-08	1.47E-07	Up	RIBC1
LYPD6	247.150815	0.71771946	0.1324903	5.41714705	6.06E-08	1.47E-07	Up	LYPD6
CD8A	258.818439	-1.07195205	0.19792337	-5.4159953	6.09E-08	1.48E-07	Down	CD8A
REG1B	1338.20069	2.56794619	0.47431088	5.41405706	6.16E-08	1.50E-07	Up	REG1B
PTGER3	146.818153	-1.13747245	0.21023283	-5.41053669	6.28E-08	1.53E-07	Down	PTGER3
TDRD5	77.8106525	1.21968988	0.22548135	5.40927167	6.33E-08	1.54E-07	Up	TDRD5
C1QTNF8	1.82089033	2.33815274	0.43226859	5.40902758	6.34E-08	1.54E-07	Up	C1QTNF8
STK32A	23.1276741	-1.27149206	0.23507559	-5.40886471	6.34E-08	1.54E-07	Down	STK32A
XAB2	2344.08649	0.56692508	0.10484349	5.40734636	6.40E-08	1.55E-07	Up	XAB2
KCNE5	3.82921144	1.12069684	0.20727899	5.40670738	6.42E-08	1.56E-07	Up	KCNE5
COL9A2	590.361662	1.06621379	0.19723895	5.40569587	6.46E-08	1.57E-07	Up	COL9A2
TEKT3	7.14946656	-0.96793053	0.17909089	-5.40468868	6.49E-08	1.58E-07	Down	TEKT3
ARHGAP24	170.230283	-0.75780224	0.14023181	-5.40392529	6.52E-08	1.58E-07	Down	ARHGAP24
ARPC4-TTLL	17.3494896	0.89987238	0.16655048	5.40300098	6.55E-08	1.59E-07	Up	ARPC4-TTLL3
TMEM19	1249.48295	-0.55185693	0.10214132	-5.40287655	6.56E-08	1.59E-07	Down	TMEM19
C1orf162	175.811878	-0.80152334	0.1483799	-5.40183225	6.60E-08	1.60E-07	Down	C1orf162
ZNF460	70.9238058	1.15527056	0.21391307	5.40065428	6.64E-08	1.61E-07	Up	ZNF460
PAIP2B	293.642416	-0.59853565	0.11083132	-5.40041975	6.65E-08	1.61E-07	Down	PAIP2B
HLA-DMB	989.543354	-0.94032263	0.17419977	-5.39795554	6.74E-08	1.63E-07	Down	HLA-DMB
RNF19B	1972.26488	-0.50066791	0.09275541	-5.3977222	6.75E-08	1.64E-07	Down	RNF19B
RYR1	35.6702666	-1.05781815	0.19602686	-5.396292	6.80E-08	1.65E-07	Down	RYR1
AP1S2	546.321823	-0.66345777	0.12295802	-5.39580702	6.82E-08	1.65E-07	Down	AP1S2
C2orf66	6.70470372	1.51344748	0.28053736	5.39481616	6.86E-08	1.66E-07	Up	C2orf66
TRAPPC2L	1530.72697	0.58928019	0.10927031	5.39286653	6.93E-08	1.68E-07	Up	TRAPPC2L
HEPACAM	2.46566213	-2.38488355	0.44223715	-5.39277076	6.94E-08	1.68E-07	Down	HEPACAM
RPLP2	34982.1909	0.61318495	0.11372506	5.39181912	6.97E-08	1.69E-07	Up	RPLP2
MAGEA10	2.02543867	3.67452908	0.6815588	5.39136031	6.99E-08	1.69E-07	Up	MAGEA10
SMIM5	89.4506211	-0.97111404	0.18013287	-5.3910983	7.00E-08	1.69E-07	Down	SMIM5
ZNF439	122.088274	-0.93397012	0.17325807	-5.39062979	7.02E-08	1.70E-07	Down	ZNF439
TBCB	2352.19393	0.52490624	0.09739955	5.38920595	7.08E-08	1.71E-07	Up	TBCB
TRNP1	312.372118	1.2646725	0.23468354	5.38884203	7.09E-08	1.72E-07	Up	TRNP1
RNF207	296.740124	0.82297863	0.1527617	5.38733619	7.15E-08	1.73E-07	Up	RNF207
GRIK2	18.2949096	-1.32863264	0.24662093	-5.38734757	7.15E-08	1.73E-07	Down	GRIK2
CCDC89	12.7916006	-0.87773692	0.16296314	-5.38610698	7.20E-08	1.74E-07	Down	CCDC89
AIRE	12.6213679	1.66939094	0.30997145	5.38562814	7.22E-08	1.74E-07	Up	AIRE
HMGB1	10138.8496	0.55188055	0.10247578	5.38547309	7.23E-08	1.75E-07	Up	HMGB1
ADAMTS4	721.07414	0.91854884	0.17058421	5.38472354	7.26E-08	1.75E-07	Up	ADAMTS4
CDH4	25.266431	1.1624579	0.21590821	5.38403759	7.28E-08	1.76E-07	Up	CDH4
CC2D2A	278.286165	-0.83259459	0.15465112	-5.38369587	7.30E-08	1.76E-07	Down	CC2D2A
LTBP2	1643.10332	0.89561136	0.1663694	5.38326963	7.31E-08	1.77E-07	Up	LTBP2
MPV17L2	600.49879	0.51851233	0.09631901	5.38328112	7.31E-08	1.77E-07	Up	MPV17L2
RAB11FIP3	1795.41445	0.51551516	0.09577109	5.38278496	7.33E-08	1.77E-07	Up	RAB11FIP3
TMCC2	48.5213703	-0.73877323	0.13726265	-5.38218672	7.36E-08	1.78E-07	Down	TMCC2
KCNJ5	46.4367653	-1.18410073	0.220065	-5.38068632	7.42E-08	1.79E-07	Down	KCNJ5
RAB40A	15.5809882	0.99954232	0.18576906	5.38056413	7.43E-08	1.79E-07	Up	RAB40A

GLCE	2436.83399	0.53519374	0.0994741	5.38023214	7.44E-08	1.79E-07	Up	GLCE
SLC6A8	4686.76708	-0.97271254	0.18084662	-5.37866021	7.50E-08	1.81E-07	Down	SLC6A8
MMAB	1387.7943	0.54497095	0.10135344	5.37693614	7.58E-08	1.83E-07	Up	MMAB
HTR2C	5.36772867	5.20433936	0.96795641	5.37662573	7.59E-08	1.83E-07	Up	HTR2C
KLHL32	80.0116538	1.18270586	0.21997378	5.37657663	7.59E-08	1.83E-07	Up	KLHL32
SPATA31C1	0.46852103	-2.3489	0.4369194	-5.3760487	7.61E-08	1.84E-07	Down	SPATA31C1
CELA3A	3.01902228	-2.00481686	0.37293499	-5.37578115	7.63E-08	1.84E-07	Down	CELA3A
ENTPD6	8223.59951	0.62255384	0.1158791	5.37244288	7.77E-08	1.87E-07	Up	ENTPD6
BEND6	22.4798288	-1.09472753	0.20383907	-5.3705481	7.85E-08	1.89E-07	Down	BEND6
RPS18	81418.941	0.65618017	0.12219889	5.36977177	7.88E-08	1.90E-07	Up	RPS18
UBE2NL	2.27045514	1.86422153	0.34723202	5.3688065	7.93E-08	1.91E-07	Up	UBE2NL
HOXC6	58.9453217	1.89259095	0.35258524	5.3677543	7.97E-08	1.92E-07	Up	HOXC6
C2orf80	0.55581446	-1.92629695	0.35903796	-5.36516235	8.09E-08	1.95E-07	Down	C2orf80
SCNN1A	6673.72622	-0.77873976	0.14515357	-5.3649372	8.10E-08	1.95E-07	Down	SCNN1A
MOGAT1	2.3608459	1.89480369	0.35331128	5.36298661	8.19E-08	1.97E-07	Up	MOGAT1
KRT19	31743.5759	-0.7001605	0.13064303	-5.3593406	8.35E-08	2.01E-07	Down	KRT19
RPL18A	22504.5013	0.65754921	0.1226988	5.35905184	8.37E-08	2.01E-07	Up	RPL18A
CLSTN3	1178.55526	0.52361667	0.09773394	5.3575723	8.43E-08	2.03E-07	Up	CLSTN3
CMYA5	32.6611833	-0.90310681	0.1685696	-5.35747141	8.44E-08	2.03E-07	Down	CMYA5
MATK	90.96507	-1.04282602	0.19467469	-5.35676216	8.47E-08	2.04E-07	Down	MATK
CYTL1	20.836536	-1.15674486	0.21596245	-5.35623134	8.50E-08	2.04E-07	Down	CYTL1
HS3ST3A1	39.1740199	1.02701725	0.19174932	5.3560412	8.51E-08	2.04E-07	Up	HS3ST3A1
ZNF772	71.9232361	-1.08295517	0.20221373	-5.35549767	8.53E-08	2.05E-07	Down	ZNF772
HTR3B	0.53222904	-2.83674941	0.52979757	-5.35440241	8.58E-08	2.06E-07	Down	HTR3B
CLEC1A	45.3522912	-0.73795792	0.13782926	-5.35414564	8.60E-08	2.06E-07	Down	CLEC1A
BBS5	142.800324	0.62928569	0.11756112	5.35283869	8.66E-08	2.08E-07	Up	BBS5
HSD3B7	719.727171	0.57801467	0.10802628	5.35068581	8.76E-08	2.10E-07	Up	HSD3B7
HUNK	1224.65917	1.0456628	0.19552085	5.34808831	8.89E-08	2.13E-07	Up	HUNK
EIF1AX	3690.40167	0.54262468	0.10147303	5.34747695	8.92E-08	2.14E-07	Up	EIF1AX
BIVM	657.308516	0.51778402	0.09685065	5.3462109	8.98E-08	2.15E-07	Up	BIVM
C20orf85	1.76332663	2.76224542	0.51705603	5.34225551	9.18E-08	2.20E-07	Up	C20orf85
NTF4	2.09379524	2.44042256	0.45690979	5.34114744	9.24E-08	2.21E-07	Up	NTF4
STK26	1306.07444	0.55044283	0.10309114	5.33938062	9.33E-08	2.24E-07	Up	STK26
PTGFR	66.6015783	-1.07190398	0.20079451	-5.33831312	9.38E-08	2.25E-07	Down	PTGFR
TYRP1	40.4592301	-1.25235689	0.23462738	-5.33764168	9.42E-08	2.26E-07	Down	TYRP1
SULT1C3	20.6567844	2.12032494	0.39724835	5.33752994	9.42E-08	2.26E-07	Up	SULT1C3
SPIN2B	130.18045	0.66224597	0.12412452	5.33533551	9.54E-08	2.28E-07	Up	SPIN2B
UBA7	1747.67118	-0.58658771	0.10996042	-5.33453499	9.58E-08	2.29E-07	Down	UBA7
FZD9	33.2695508	1.45731838	0.27318548	5.33453829	9.58E-08	2.29E-07	Up	FZD9
IKZF3	367.277115	-1.02611458	0.19235697	-5.33442891	9.58E-08	2.29E-07	Down	IKZF3
CCDC170	253.51094	1.01611577	0.19049516	5.33407654	9.60E-08	2.30E-07	Up	CCDC170
GAL	409.554653	1.41048634	0.26452499	5.33214783	9.71E-08	2.32E-07	Up	GAL
ZFR2	2.8310162	-1.3631593	0.25581488	-5.32869441	9.89E-08	2.37E-07	Down	ZFR2
HSPA4L	458.486623	1.06480191	0.19985942	5.32775442	9.94E-08	2.38E-07	Up	HSPA4L
QKI	989.695134	-0.85568966	0.16062531	-5.32724067	9.97E-08	2.38E-07	Down	QKI
APOLD1	1639.4889	1.16245984	0.21824404	5.32642194	1.00E-07	2.39E-07	Up	APOLD1
NPIPB3	56.6365966	0.87858561	0.16494964	5.32638684	1.00E-07	2.39E-07	Up	NPIPB3
OLAH	2.67551453	1.67577752	0.3146425	5.32597323	1.00E-07	2.40E-07	Up	OLAH
SNX8	1568.05754	0.52386497	0.09836915	5.32550041	1.01E-07	2.40E-07	Up	SNX8
TRIM58	44.1150161	-1.55690642	0.29247469	-5.32321755	1.02E-07	2.43E-07	Down	TRIM58
PTCRA	3.75977997	-1.16026757	0.21796891	-5.32308746	1.02E-07	2.43E-07	Down	PTCRA
ALPK2	53.087904	1.07182272	0.20140426	5.32174792	1.03E-07	2.45E-07	Up	ALPK2
GMPR	391.048198	1.03701282	0.19495305	5.3192953	1.04E-07	2.48E-07	Up	GMPR
PPIC	1645.63116	-0.51853153	0.09752288	-5.31702463	1.05E-07	2.52E-07	Down	PPIC
RASAL1	524.476988	1.07763284	0.20270368	5.31629626	1.06E-07	2.52E-07	Up	RASAL1
ACTL8	50.7551844	1.51461737	0.28494816	5.31541383	1.06E-07	2.54E-07	Up	ACTL8
CCDC70	0.78885147	2.47392542	0.46546576	5.31494612	1.07E-07	2.54E-07	Up	CCDC70
TBX18	113.35527	1.59855445	0.30078131	5.31467356	1.07E-07	2.55E-07	Up	TBX18

MOV10L1	15.8499687	1.00693358	0.18950138	5.31359499	1.07E-07	2.56E-07	Up	MOV10L1
URGCP-MRF	1.78854237	-0.89310891	0.16808237	-5.31351934	1.08E-07	2.56E-07	Down	URGCP-MRPS24
OR1J4	1.25002837	2.77740412	0.52282751	5.31227618	1.08E-07	2.58E-07	Up	OR1J4
PKN3	466.751741	0.66836149	0.12589508	5.30887698	1.10E-07	2.63E-07	Up	PKN3
HGFAC	3.69072816	1.63926748	0.30887037	5.30729928	1.11E-07	2.65E-07	Up	HGFAC
PSTPIP2	464.679331	0.74346499	0.14009015	5.30704678	1.11E-07	2.65E-07	Up	PSTPIP2
SESN1	2014.97968	0.8446154	0.15919466	5.30555103	1.12E-07	2.67E-07	Up	SESN1
PARP10	2555.9375	0.61318593	0.115624	5.30327558	1.14E-07	2.71E-07	Up	PARP10
LRIT3	9.33304911	1.04481134	0.19714858	5.2996138	1.16E-07	2.76E-07	Up	LRIT3
CASP4	1394.49229	0.53666219	0.10127975	5.29881037	1.17E-07	2.77E-07	Up	CASP4
STXBP5	1217.44751	-0.55556115	0.10491579	-5.29530539	1.19E-07	2.82E-07	Down	STXBP5
LARP4	1956.90564	0.5142098	0.09711796	5.29469301	1.19E-07	2.83E-07	Up	LARP4
SIGLEC10	216.655365	-0.86258583	0.16292604	-5.29433971	1.19E-07	2.84E-07	Down	SIGLEC10
POLN	27.2531871	0.90698347	0.17132348	5.29398214	1.20E-07	2.84E-07	Up	POLN
CD244	41.9461781	-1.01520723	0.19176804	-5.29393328	1.20E-07	2.84E-07	Down	CD244
ANKK1	12.6169253	1.1740167	0.2217866	5.29345186	1.20E-07	2.85E-07	Up	ANKK1
CNBD2	8.62810263	0.94271414	0.17810787	5.29293941	1.20E-07	2.86E-07	Up	CNBD2
NABP1	732.035359	0.72468167	0.13694736	5.29168043	1.21E-07	2.88E-07	Up	NABP1
COL17A1	5233.05073	-1.03319111	0.19525966	-5.29137011	1.21E-07	2.88E-07	Down	COL17A1
SYCP2	55.1733691	1.22843935	0.23216113	5.29132215	1.21E-07	2.88E-07	Up	SYCP2
DCDC2	149.656348	1.53917986	0.29089445	5.29119704	1.22E-07	2.88E-07	Up	DCDC2
ABL2	898.297295	0.52735456	0.09967444	5.29077003	1.22E-07	2.89E-07	Up	ABL2
GABRG1	0.75377265	-3.01489891	0.57011691	-5.28821171	1.24E-07	2.93E-07	Down	GABRG1
FRMD4A	307.247795	-0.60613348	0.11462571	-5.28793632	1.24E-07	2.93E-07	Down	FRMD4A
NFAT5	1684.36999	0.82960134	0.15698635	5.28454446	1.26E-07	2.99E-07	Up	NFAT5
SPOCK2	781.637107	-0.81283013	0.15383083	-5.28392199	1.26E-07	3.00E-07	Down	SPOCK2
LAMA2	486.368813	-0.99881646	0.18910795	-5.28172651	1.28E-07	3.03E-07	Down	LAMA2
FAM83B	387.384784	-0.6677609	0.12645023	-5.28081992	1.29E-07	3.05E-07	Down	FAM83B
GRM4	23.7889096	1.70839426	0.32351087	5.28079397	1.29E-07	3.05E-07	Up	GRM4
FAM24B	47.1578114	0.82770744	0.15674054	5.28074904	1.29E-07	3.05E-07	Up	FAM24B
FAM120C	211.366618	0.64968207	0.12306309	5.27926006	1.30E-07	3.07E-07	Up	FAM120C
ZAR1	2.42763215	2.28399878	0.43267122	5.27883229	1.30E-07	3.08E-07	Up	ZAR1
MYOZ3	12.1090976	-1.03538167	0.19618267	-5.2776408	1.31E-07	3.10E-07	Down	MYOZ3
TRPA1	302.779659	-1.17173025	0.22212784	-5.27502665	1.33E-07	3.14E-07	Down	TRPA1
ZNF528	154.328779	-1.00688277	0.19088346	-5.27485591	1.33E-07	3.15E-07	Down	ZNF528
GSX1	1.32201087	3.19040577	0.60486797	5.27454907	1.33E-07	3.15E-07	Up	GSX1
CAMK2N2	15.745656	1.33026839	0.25241463	5.27017153	1.36E-07	3.23E-07	Up	CAMK2N2
CRISPLD2	1454.51337	-0.82517406	0.15657746	-5.2700691	1.36E-07	3.23E-07	Down	CRISPLD2
NAB1	1660.35053	0.5363081	0.10180141	5.26817929	1.38E-07	3.26E-07	Up	NAB1
NEURL1	482.953965	-1.27107957	0.24138465	-5.2657847	1.40E-07	3.30E-07	Down	NEURL1
TRIM3	614.430594	-0.54606711	0.10371907	-5.26486717	1.40E-07	3.32E-07	Down	TRIM3
LSAMP	348.927006	-0.97037209	0.18431795	-5.264664	1.40E-07	3.32E-07	Down	LSAMP
ROCK1	1385.77838	-0.55960594	0.10633089	-5.26287297	1.42E-07	3.35E-07	Down	ROCK1
PAX3	3.89642735	3.18851232	0.60588436	5.26257572	1.42E-07	3.36E-07	Up	PAX3
ISM1	89.9502174	1.34509374	0.25560426	5.26240741	1.42E-07	3.36E-07	Up	ISM1
CCRL2	507.112439	-0.51251997	0.09743362	-5.26019634	1.44E-07	3.40E-07	Down	CCRL2
TLR1	126.858803	-0.97595989	0.18556534	-5.25938678	1.45E-07	3.41E-07	Down	TLR1
LBH	1879.99702	0.66188971	0.12585064	5.25932741	1.45E-07	3.41E-07	Up	LBH
HCN3	374.839051	0.58932559	0.11205482	5.25926127	1.45E-07	3.41E-07	Up	HCN3
NRXN2	211.794042	-1.15537928	0.21971052	-5.2586435	1.45E-07	3.43E-07	Down	NRXN2
IDS	2184.07585	-0.54953619	0.10459405	-5.25399107	1.49E-07	3.51E-07	Down	IDS
TP53	3957.56015	0.71585406	0.13625477	5.25379087	1.49E-07	3.52E-07	Up	TP53
GLRB	34.7237472	-1.01540922	0.19328931	-5.25331275	1.49E-07	3.53E-07	Down	GLRB
FGFBP1	404.783972	-1.09787943	0.20906587	-5.25135658	1.51E-07	3.56E-07	Down	FGFBP1
SCHIP1	18.0210094	-1.09684557	0.20888419	-5.25097463	1.51E-07	3.57E-07	Down	SCHIP1
PLXDC1	377.130392	0.64730245	0.12329047	5.25022298	1.52E-07	3.58E-07	Up	PLXDC1
KRT8	101343.765	-0.58152195	0.11077584	-5.24953771	1.52E-07	3.59E-07	Down	KRT8
SLN	9.56138835	1.69613027	0.32336413	5.24526409	1.56E-07	3.68E-07	Up	SLN

SALL1	96.6783527	-0.87600544	0.16704263	-5.24420293	1.57E-07	3.70E-07	Down	SALL1
SELL	227.376628	-1.04442724	0.19932494	-5.23982218	1.61E-07	3.79E-07	Down	SELL
DHH	9.44409553	-0.91629907	0.17489145	-5.23924439	1.61E-07	3.80E-07	Down	DHH
NEURL2	35.0830032	0.71449583	0.13640558	5.23802494	1.62E-07	3.82E-07	Up	NEURL2
CD82	2196.0741	0.56783494	0.10841081	5.23780759	1.62E-07	3.83E-07	Up	CD82
SNCAIP	214.379325	1.16710831	0.22284142	5.23739387	1.63E-07	3.83E-07	Up	SNCAIP
MAST4	595.962982	-0.55202122	0.10545304	-5.23475874	1.65E-07	3.89E-07	Down	MAST4
RBP7	41.2806963	-0.97092053	0.18547869	-5.23467425	1.65E-07	3.89E-07	Down	RBP7
MMP15	4971.05133	-0.55602585	0.1062225	-5.23453927	1.65E-07	3.89E-07	Down	MMP15
ZFP82	56.4285608	-0.93867318	0.17934423	-5.23391891	1.66E-07	3.90E-07	Down	ZFP82
C1QL4	3.20936983	1.78216818	0.34055008	5.23320445	1.67E-07	3.92E-07	Up	C1QL4
KCNRG	17.0881831	0.97851757	0.18703165	5.23182872	1.68E-07	3.95E-07	Up	KCNRG
ETS1	2092.28305	-0.67617619	0.12925287	-5.23142095	1.68E-07	3.95E-07	Down	ETS1
FFAR4	659.414324	-1.2306762	0.23526449	-5.23103252	1.69E-07	3.96E-07	Down	FFAR4
GH1	0.57618198	-1.70976119	0.32690935	-5.2300774	1.69E-07	3.98E-07	Down	GH1
KIF16B	1591.10591	-0.53496392	0.10230152	-5.22928614	1.70E-07	4.00E-07	Down	KIF16B
RDX	759.580377	-0.86131717	0.16471025	-5.22928706	1.70E-07	4.00E-07	Down	RDX
LHX3	1.21585886	2.66367052	0.50938136	5.22922657	1.70E-07	4.00E-07	Up	LHX3
ARFRP1	2046.33891	0.55596682	0.10632533	5.22892187	1.71E-07	4.01E-07	Up	ARFRP1
UQCR10	2776.01272	-0.55309347	0.10581453	-5.22700883	1.72E-07	4.05E-07	Down	UQCR10
RBM12B	532.827065	0.55741463	0.10664826	5.22666429	1.73E-07	4.05E-07	Up	RBM12B
LYZ	17834.4532	1.34587563	0.25757434	5.22519303	1.74E-07	4.08E-07	Up	LYZ
CD69	142.320445	-1.05978288	0.20284966	-5.22447444	1.75E-07	4.10E-07	Down	CD69
SLC39A12	0.71161842	-1.80011295	0.34458503	-5.22400216	1.75E-07	4.11E-07	Down	SLC39A12
NDUFV2	341.077549	-0.58384711	0.11178071	-5.22314712	1.76E-07	4.13E-07	Down	NDUFV2
PPP1R3D	566.213181	0.5585991	0.10697344	5.22184839	1.77E-07	4.16E-07	Up	PPP1R3D
DOCK8	634.069918	-0.85098722	0.16298958	-5.22111432	1.78E-07	4.17E-07	Down	DOCK8
CBWD1	353.345456	0.5043693	0.09661008	5.22066942	1.78E-07	4.18E-07	Up	CBWD1
CLDN20	11.1075549	1.35390118	0.2593507	5.22034897	1.79E-07	4.19E-07	Up	CLDN20
VCAM1	540.847204	-0.80349163	0.15391762	-5.22027052	1.79E-07	4.19E-07	Down	VCAM1
FAM229B	82.8061233	-0.77211904	0.14792448	-5.21968398	1.79E-07	4.20E-07	Down	FAM229B
SNRPG	1673.95296	0.51057524	0.09787442	5.21663597	1.82E-07	4.27E-07	Up	SNRPG
TNR	7.35080742	-1.32116969	0.25334345	-5.21493525	1.84E-07	4.31E-07	Down	TNR
TCP11L2	179.838661	-0.71157203	0.13648495	-5.21355657	1.85E-07	4.34E-07	Down	TCP11L2
RIT2	0.4037243	-2.95159094	0.566159	-5.21336042	1.85E-07	4.34E-07	Down	RIT2
TCAP	26.1654162	0.88041558	0.1688992	5.21266884	1.86E-07	4.36E-07	Up	TCAP
GLIPR1L2	10.1762539	-0.92907275	0.17824389	-5.21236786	1.86E-07	4.36E-07	Down	GLIPR1L2
SERTAD1	767.986207	-0.58054902	0.11149732	-5.20684268	1.92E-07	4.49E-07	Down	SERTAD1
AP1G2	1646.08656	0.5061024	0.09720394	5.206604	1.92E-07	4.50E-07	Up	AP1G2
ZNF569	59.1249047	-0.80574565	0.15485412	-5.20325596	1.96E-07	4.58E-07	Down	ZNF569
FAM183A	11.5574083	1.52573672	0.29323663	5.20309045	1.96E-07	4.58E-07	Up	FAM183A
RPS6KA6	271.16578	-1.1814193	0.22706449	-5.20301219	1.96E-07	4.58E-07	Down	RPS6KA6
HOXD4	9.49204613	-1.15539884	0.2221628	-5.20068538	1.99E-07	4.64E-07	Down	HOXD4
GSTZ1	615.589926	-0.64353736	0.1237421	-5.20063375	1.99E-07	4.64E-07	Down	GSTZ1
PCDHGB7	82.4810503	-1.0350432	0.19902382	-5.20059967	1.99E-07	4.64E-07	Down	PCDHGB7
SDC2	1314.87364	-0.74227286	0.14277374	-5.19894519	2.00E-07	4.68E-07	Down	SDC2
HSPB3	20.4529781	-2.17854279	0.41911876	-5.19791286	2.02E-07	4.71E-07	Down	HSPB3
GRIA2	2.3622238	-1.94161419	0.37362082	-5.1967505	2.03E-07	4.74E-07	Down	GRIA2
ZNF208	9.14059374	-1.18609211	0.22827277	-5.19594216	2.04E-07	4.76E-07	Down	ZNF208
PFN4	7.97540241	0.88759773	0.17085578	5.19501152	2.05E-07	4.78E-07	Up	PFN4
C17orf107	38.7268099	-0.711677	0.13701019	-5.19433638	2.05E-07	4.79E-07	Down	C17orf107
ISYNA1	645.306287	0.94394977	0.18180334	5.19214761	2.08E-07	4.85E-07	Up	ISYNA1
GABPA	840.8961	-0.53020893	0.10213754	-5.19112666	2.09E-07	4.87E-07	Down	GABPA
DNLZ	5.52136795	0.9753998	0.18801494	5.18788466	2.13E-07	4.96E-07	Up	DNLZ
TUBB2B	101.845934	-1.25488077	0.24196384	-5.18623271	2.15E-07	5.00E-07	Down	TUBB2B
CERS1	5.72468214	-1.30808773	0.25223541	-5.18597984	2.15E-07	5.01E-07	Down	CERS1
SLC45A1	39.113495	-0.75029928	0.14468469	-5.1857544	2.15E-07	5.01E-07	Down	SLC45A1
FAM166A	3.00771603	1.36295922	0.26283649	5.18557837	2.15E-07	5.02E-07	Up	FAM166A

MUC12	8193.28862	-1.33662998	0.25777309	-5.18529686	2.16E-07	5.02E-07	Down	MUC12
SMARCA1	370.345236	-0.89727938	0.17313556	-5.18252506	2.19E-07	5.10E-07	Down	SMARCA1
FAM171B	106.214115	-0.87038314	0.16794907	-5.18242322	2.19E-07	5.10E-07	Down	FAM171B
KLRG1	24.6480328	-0.85817294	0.16563589	-5.18108076	2.21E-07	5.13E-07	Down	KLRG1
CCDC183	122.158167	0.78950906	0.15240722	5.18026019	2.22E-07	5.16E-07	Up	CCDC183
C1orf167	0.73491155	-1.40695436	0.27160153	-5.18021519	2.22E-07	5.16E-07	Down	C1orf167
DLL4	940.43225	0.55414561	0.10699471	5.17918691	2.23E-07	5.18E-07	Up	DLL4
LYRM1	1078.31916	0.51092595	0.09866357	5.17846589	2.24E-07	5.20E-07	Up	LYRM1
VSIG1	35.9231826	1.47587771	0.28525066	5.17396765	2.29E-07	5.33E-07	Up	VSIG1
CLEC2B	150.485022	-0.87643121	0.16939195	-5.17398393	2.29E-07	5.33E-07	Down	CLEC2B
MDM4	1251.8759	0.63630096	0.12300684	5.17289085	2.30E-07	5.36E-07	Up	MDM4
MGARP	4.37710526	-0.99517506	0.1923861	-5.17280131	2.31E-07	5.36E-07	Down	MGARP
OTP	1.1561658	2.28367749	0.44161648	5.17117815	2.33E-07	5.40E-07	Up	OTP
DTWD2	261.812384	-0.51578693	0.0998119	-5.16758959	2.37E-07	5.51E-07	Down	DTWD2
ZGPAT	357.14769	0.55537749	0.10747519	5.16749467	2.37E-07	5.51E-07	Up	ZGPAT
NTNG2	61.5378543	0.77908913	0.15077407	5.16726202	2.38E-07	5.52E-07	Up	NTNG2
LIN28A	3.55234959	3.17856596	0.61517071	5.16696571	2.38E-07	5.52E-07	Up	LIN28A
PIR	388.093629	0.61055776	0.11823083	5.16411614	2.42E-07	5.60E-07	Up	PIR
NKX2-8	2.15609033	3.78940888	0.73408235	5.16210326	2.44E-07	5.66E-07	Up	NKX2-8
LMOD3	14.540537	1.13845501	0.22055836	5.16169508	2.45E-07	5.68E-07	Up	LMOD3
COL18A1	4150.919	0.74844005	0.14499889	5.16169496	2.45E-07	5.68E-07	Up	COL18A1
PPARG	2381.53865	-0.59126359	0.11455421	-5.16143067	2.45E-07	5.68E-07	Down	PPARG
L1CAM	571.224363	-1.49751197	0.29022339	-5.1598596	2.47E-07	5.73E-07	Down	L1CAM
TIMM13	3432.43137	0.63876215	0.12380299	5.15950501	2.48E-07	5.74E-07	Up	TIMM13
PIK3C2G	2.55242199	-2.50900458	0.48629498	-5.1594293	2.48E-07	5.74E-07	Down	PIK3C2G
SKIL	2320.22074	0.6889674	0.13357098	5.15806204	2.50E-07	5.78E-07	Up	SKIL
LPIN1	1308.60145	0.51719435	0.10027679	5.15766767	2.50E-07	5.79E-07	Up	LPIN1
POU2F1	1062.24178	0.5391699	0.10455082	5.15701265	2.51E-07	5.81E-07	Up	POU2F1
MAGEA1	3.98169682	4.77504826	0.92598232	5.15673801	2.51E-07	5.82E-07	Up	MAGEA1
ZNF93	159.257936	0.80225853	0.15559917	5.15593066	2.52E-07	5.84E-07	Up	ZNF93
CDRT1	6.29475616	1.18580842	0.23016305	5.15203646	2.58E-07	5.97E-07	Up	CDRT1
ZNF717	223.588921	0.67173834	0.13041913	5.1506121	2.60E-07	6.01E-07	Up	ZNF717
GSTM3	652.717822	-0.97926328	0.19015554	-5.14980135	2.61E-07	6.03E-07	Down	GSTM3
MAGED2	5216.64106	0.50100956	0.09728882	5.14971367	2.61E-07	6.03E-07	Up	MAGED2
CRABP2	266.626876	1.22421303	0.23775213	5.14911498	2.62E-07	6.05E-07	Up	CRABP2
NOXO1	2.47736266	2.35341227	0.45706657	5.14894863	2.62E-07	6.06E-07	Up	NOXO1
CYP27A1	1686.63967	-0.80652621	0.15671444	-5.14647036	2.65E-07	6.14E-07	Down	CYP27A1
EPHA4	281.151238	-1.08691968	0.21120723	-5.14622378	2.66E-07	6.14E-07	Down	EPHA4
CSAG3	4.92166834	4.70071547	0.9138025	5.14412626	2.69E-07	6.21E-07	Up	CSAG3
ZNF525	359.676576	0.76294276	0.14832799	5.1436196	2.69E-07	6.23E-07	Up	ZNF525
KLHL1	0.64591843	-3.08296443	0.5994202	-5.14324413	2.70E-07	6.24E-07	Down	KLHL1
MAP3K8	417.400876	0.5310101	0.10326862	5.14202777	2.72E-07	6.28E-07	Up	MAP3K8
TMEM262	38.3350878	0.54918968	0.10681028	5.14173042	2.72E-07	6.29E-07	Up	TMEM262
ZDBF2	104.184483	-1.11340024	0.21687807	-5.13376132	2.84E-07	6.55E-07	Down	ZDBF2
SMN1	159.001695	0.5396513	0.10518317	5.13058622	2.89E-07	6.66E-07	Up	SMN1
FITM1	13.3833455	-0.70291742	0.13705437	-5.12874873	2.92E-07	6.73E-07	Down	FITM1
FBLN7	43.9042623	-0.73997411	0.14447538	-5.12180056	3.03E-07	6.98E-07	Down	FBLN7
TEX29	5.57475301	0.88883957	0.17354042	5.12180143	3.03E-07	6.98E-07	Up	TEX29
MCOLN1	650.552271	-0.50796265	0.09927126	-5.11691565	3.11E-07	7.16E-07	Down	MCOLN1
ENPP4	1026.58385	-0.50311453	0.0983331	-5.11643093	3.11E-07	7.17E-07	Down	ENPP4
BTG2	4934.52451	-0.57856257	0.11310703	-5.11517783	3.13E-07	7.22E-07	Down	BTG2
SGMS2	1102.47476	-0.68437234	0.13383849	-5.11341939	3.16E-07	7.29E-07	Down	SGMS2
ASB18	0.88708864	2.52818661	0.49448907	5.11272497	3.18E-07	7.31E-07	Up	ASB18
RPEL1	3.16596728	1.35766244	0.26554567	5.11272673	3.18E-07	7.31E-07	Up	RPEL1
PPP4C	4906.28503	0.57064444	0.11163538	5.11168099	3.19E-07	7.35E-07	Up	PPP4C
PRDM1	841.780794	-0.669061	0.13089003	-5.11162684	3.19E-07	7.35E-07	Down	PRDM1
OR2A7	25.7688647	-0.91211564	0.17844079	-5.11158707	3.19E-07	7.35E-07	Down	OR2A7
CFAP54	11.5698748	-1.15675468	0.22635393	-5.11038035	3.22E-07	7.40E-07	Down	CFAP54

PROM2	1132.46554	-1.16399617	0.22790499	-5.10737463	3.27E-07	7.51E-07	Down	PROM2
ZNF382	34.7897043	-0.88247836	0.17279882	-5.10696972	3.27E-07	7.53E-07	Down	ZNF382
ACAP1	298.709455	-0.72593155	0.14215587	-5.10658858	3.28E-07	7.54E-07	Down	ACAP1
PTGDR	149.58948	-1.65245904	0.32369481	-5.10499091	3.31E-07	7.60E-07	Down	PTGDR
LINGO1	193.858671	0.97059586	0.19018442	5.10344573	3.34E-07	7.67E-07	Up	LINGO1
ZAN	1.71647166	2.31626832	0.45388668	5.10318636	3.34E-07	7.67E-07	Up	ZAN
RPL36A-HNF	2.30401774	1.21312217	0.23775403	5.10242536	3.35E-07	7.70E-07	Up	RPL36A-HNRNPH2
COPZ2	196.230932	-0.77684146	0.15227149	-5.10168678	3.37E-07	7.73E-07	Down	COPZ2
NOTO	0.58570473	-1.95840619	0.38403911	-5.0994967	3.41E-07	7.82E-07	Down	NOTO
FAM162B	15.6627608	-0.81074828	0.15898769	-5.09944068	3.41E-07	7.82E-07	Down	FAM162B
SLC25A10	1315.89471	0.63794689	0.12510218	5.09940657	3.41E-07	7.82E-07	Up	SLC25A10
SLC26A4	13.7672677	-0.9157506	0.17958493	-5.09926184	3.41E-07	7.83E-07	Down	SLC26A4
PSD3	874.559462	-0.68359458	0.13409821	-5.09771584	3.44E-07	7.89E-07	Down	PSD3
CLEC6A	4.61063395	1.664105	0.32647937	5.09712146	3.45E-07	7.91E-07	Up	CLEC6A
GDF5	5.69794593	-1.63286172	0.32037854	-5.09666387	3.46E-07	7.93E-07	Down	GDF5
KRT78	1.29224623	2.46687945	0.48408553	5.09595789	3.47E-07	7.96E-07	Up	KRT78
GDAP1	285.439348	0.68351326	0.13413582	5.0956803	3.47E-07	7.97E-07	Up	GDAP1
CHDH	2437.95756	0.53750604	0.10554477	5.09268302	3.53E-07	8.09E-07	Up	CHDH
AUTS2	2331.26554	0.55713919	0.10944107	5.09076898	3.57E-07	8.17E-07	Up	AUTS2
SLX1A	1.2516054	1.8365253	0.360964	5.08783502	3.62E-07	8.30E-07	Up	SLX1A
SUSD3	273.035064	1.00118703	0.1967885	5.08762986	3.63E-07	8.31E-07	Up	SUSD3
ZNF564	30.5543048	-0.63363839	0.12457045	-5.08658685	3.65E-07	8.35E-07	Down	ZNF564
ZNF549	103.99429	-0.95885125	0.18858096	-5.08456037	3.68E-07	8.44E-07	Down	ZNF549
SLITRK1	0.67153529	-2.36146192	0.46461518	-5.08261899	3.72E-07	8.52E-07	Down	SLITRK1
TBX21	22.8787727	-0.98629253	0.19405392	-5.0825695	3.72E-07	8.52E-07	Down	TBX21
ATP6V0C	1053.15434	-0.56701356	0.11176091	-5.07345156	3.91E-07	8.93E-07	Down	ATP6V0C
EFCAB13	93.2709262	0.99657735	0.19647127	5.07238222	3.93E-07	8.98E-07	Up	EFCAB13
GUCY2D	7.03520908	1.29871309	0.25617976	5.06953817	3.99E-07	9.11E-07	Up	GUCY2D
MYT1	31.794872	1.37022796	0.27032985	5.06872616	4.00E-07	9.15E-07	Up	MYT1
KCNJ16	8.44224711	-1.70042643	0.33552733	-5.06792232	4.02E-07	9.19E-07	Down	KCNJ16
RPL19	47837.1255	0.54367734	0.10730093	5.06684657	4.04E-07	9.24E-07	Up	RPL19
INSL4	2.53936094	3.52766004	0.69636292	5.06583557	4.07E-07	9.28E-07	Up	INSL4
ADAMTS20	2.89973671	4.23772156	0.83716726	5.0619772	4.15E-07	9.47E-07	Up	ADAMTS20
SUN3	2.50485998	1.44072746	0.2846414	5.06155278	4.16E-07	9.49E-07	Up	SUN3
ZC3H12D	247.451936	-0.61693137	0.12194709	-5.05900865	4.21E-07	9.62E-07	Down	ZC3H12D
FRZB	592.941059	-0.9993262	0.1975374	-5.05892157	4.22E-07	9.62E-07	Down	FRZB
DBNDD2	284.875791	-0.51256931	0.10135701	-5.05706821	4.26E-07	9.71E-07	Down	DBNDD2
SLFNL1	15.3868232	0.99007613	0.1958145	5.05619423	4.28E-07	9.75E-07	Up	SLFNL1
LYRM9	110.440521	-0.50712092	0.10030547	-5.05576515	4.29E-07	9.77E-07	Down	LYRM9
CD300LB	24.9472501	-0.85293976	0.16872015	-5.05535208	4.30E-07	9.79E-07	Down	CD300LB
CD24	34403.412	-0.70927996	0.14038324	-5.0524547	4.36E-07	9.94E-07	Down	CD24
KNG1	32.411506	-1.42928154	0.28301688	-5.05016364	4.41E-07	1.01E-06	Down	KNG1
SLC25A2	2.21992259	1.60287645	0.31762837	5.04638949	4.50E-07	1.03E-06	Up	SLC25A2
PECAM1	2891.13497	-0.61650803	0.12219542	-5.04526302	4.53E-07	1.03E-06	Down	PECAM1
PBOV1	2.66167209	3.07262939	0.6090803	5.04470331	4.54E-07	1.03E-06	Up	PBOV1
ZNF512B	216.377813	-0.7039375	0.13959369	-5.0427603	4.59E-07	1.04E-06	Down	ZNF512B
SLC5A10	9.21695827	-0.80576542	0.15981927	-5.04172887	4.61E-07	1.05E-06	Down	SLC5A10
AKR1C3	2494.80697	-0.89907995	0.17840269	-5.03960972	4.66E-07	1.06E-06	Down	AKR1C3
TFAP2B	0.73374303	-2.95877293	0.58715554	-5.03916376	4.68E-07	1.06E-06	Down	TFAP2B
TAS2R4	21.1718911	1.14942513	0.22818994	5.03714191	4.73E-07	1.07E-06	Up	TAS2R4
TTLL10	15.1277445	1.0721102	0.21284216	5.03711397	4.73E-07	1.07E-06	Up	TTLL10
TRAM1L1	25.12707	-0.8614578	0.17107178	-5.03565104	4.76E-07	1.08E-06	Down	TRAM1L1
FCAR	19.7724861	1.47978364	0.29387322	5.0354491	4.77E-07	1.08E-06	Up	FCAR
LRRC18	3.91595059	-1.16242872	0.2309512	-5.03322221	4.82E-07	1.10E-06	Down	LRRC18
SEMA3B	1877.63082	-0.78542848	0.15613394	-5.03047902	4.89E-07	1.11E-06	Down	SEMA3B
ELMOD1	4.48324333	-1.35922539	0.27022388	-5.02999739	4.90E-07	1.11E-06	Down	ELMOD1
GPC1	1510.58937	0.73636293	0.14655492	5.02448438	5.05E-07	1.15E-06	Up	GPC1
ZNF467	101.096093	-0.74181587	0.14764127	-5.02444805	5.05E-07	1.15E-06	Down	ZNF467

TAS2R19	6.24148589	1.59964313	0.31840295	5.02395833	5.06E-07	1.15E-06	Up	TAS2R19
SLC1A7	280.74694	1.3987582	0.27850485	5.02238363	5.10E-07	1.16E-06	Up	SLC1A7
IRAK1BP1	258.159798	0.53439427	0.10642144	5.02149055	5.13E-07	1.16E-06	Up	IRAK1BP1
GPSM3	583.507232	-0.56340202	0.11221202	-5.02087045	5.14E-07	1.17E-06	Down	GPSM3
TXNDC5	472.466116	-0.55911366	0.11140367	-5.01880825	5.20E-07	1.18E-06	Down	TXNDC5
TRIM6	37.353946	-0.78299475	0.1560152	-5.01870809	5.20E-07	1.18E-06	Down	TRIM6
SHARPIN	2481.60992	0.55206922	0.11003245	5.01733108	5.24E-07	1.19E-06	Up	SHARPIN
FERMT3	838.162199	-0.77554739	0.1545887	-5.01684395	5.25E-07	1.19E-06	Down	FERMT3
ALPP	22.7626866	1.97420055	0.39355945	5.01627016	5.27E-07	1.19E-06	Up	ALPP
MICB	251.313813	0.78073764	0.15571202	5.01398447	5.33E-07	1.21E-06	Up	MICB
CES4A	104.957924	0.82984946	0.16553621	5.0130994	5.36E-07	1.21E-06	Up	CES4A
NPIPA5	32.4022101	1.03214291	0.20589855	5.01287133	5.36E-07	1.21E-06	Up	NPIPA5
FHAD1	49.3283812	0.81327661	0.16231398	5.01051499	5.43E-07	1.23E-06	Up	FHAD1
ADRA1D	15.5766229	-1.2180912	0.24312667	-5.01010938	5.44E-07	1.23E-06	Down	ADRA1D
SCML2	131.677079	0.86268994	0.17219127	5.01006794	5.44E-07	1.23E-06	Up	SCML2
FUT7	24.4643215	0.97807179	0.19535421	5.00665849	5.54E-07	1.25E-06	Up	FUT7
TNFRSF19	219.273271	1.32254946	0.26423758	5.00515287	5.58E-07	1.26E-06	Up	TNFRSF19
LCA5L	45.5919098	0.64333487	0.12855347	5.00441458	5.60E-07	1.27E-06	Up	LCA5L
ATG101	1065.62433	0.5032603	0.10061435	5.00187398	5.68E-07	1.28E-06	Up	ATG101
CYP46A1	7.6242917	-0.91345128	0.18275869	-4.99812761	5.79E-07	1.31E-06	Down	CYP46A1
ASTN2	146.850778	-0.52491205	0.10502139	-4.99814413	5.79E-07	1.31E-06	Down	ASTN2
DAW1	2.13478971	1.83382404	0.36692397	4.99783108	5.80E-07	1.31E-06	Up	DAW1
DHRS4L2	488.383685	-0.54291252	0.1086355	-4.99756102	5.81E-07	1.31E-06	Down	DHRS4L2
GRID1	45.0503819	-0.81048005	0.16227499	-4.99448522	5.90E-07	1.33E-06	Down	GRID1
CEP295	370.370054	0.59209385	0.11861502	4.99172754	5.98E-07	1.35E-06	Up	CEP295
GSG1	1.40211044	-1.94643697	0.38997463	-4.99118877	6.00E-07	1.35E-06	Down	GSG1
CDC42EP2	752.868109	0.59661639	0.11961178	4.98793997	6.10E-07	1.38E-06	Up	CDC42EP2
TGFBR3L	8.95387023	-1.23979231	0.24860242	-4.9870485	6.13E-07	1.38E-06	Down	TGFBR3L
B4GALNT1	53.0138379	-0.7458703	0.14960507	-4.98559494	6.18E-07	1.39E-06	Down	B4GALNT1
ARID3C	6.31836163	1.08430241	0.21750831	4.98510804	6.19E-07	1.40E-06	Up	ARID3C
IL37	15.5203032	1.46287335	0.29360313	4.98248559	6.28E-07	1.41E-06	Up	IL37
CSRNP3	52.7234037	-1.11991524	0.22477233	-4.98244269	6.28E-07	1.41E-06	Down	CSRNP3
SAMSN1	209.3974	-0.86592326	0.17397257	-4.97735511	6.45E-07	1.45E-06	Down	SAMSN1
GPR174	14.8143425	-1.18600581	0.23829173	-4.97711697	6.45E-07	1.45E-06	Down	GPR174
AMZ1	16.8617247	1.12718172	0.22651037	4.97629198	6.48E-07	1.46E-06	Up	AMZ1
KCNS1	8.46848368	1.39411336	0.2801705	4.97594622	6.49E-07	1.46E-06	Up	KCNS1
STX11	85.6392212	-0.83417581	0.16769062	-4.97449309	6.54E-07	1.47E-06	Down	STX11
GIPC3	94.889097	-0.57478876	0.1155625	-4.97383475	6.56E-07	1.48E-06	Down	GIPC3
TLR6	64.3775143	-0.87705079	0.17634503	-4.97349315	6.58E-07	1.48E-06	Down	TLR6
NPDC1	4502.78589	0.71597816	0.14402025	4.97137134	6.65E-07	1.49E-06	Up	NPDC1
AMOT	1387.85055	0.68491396	0.13782402	4.96948167	6.71E-07	1.51E-06	Up	AMOT
ZNF782	141.2814	0.56416833	0.11354111	4.96884627	6.74E-07	1.51E-06	Up	ZNF782
NUTM2B	4.74191976	-1.09384384	0.22018258	-4.96789445	6.77E-07	1.52E-06	Down	NUTM2B
CCDC27	0.7385596	-1.67357158	0.33691977	-4.96727028	6.79E-07	1.53E-06	Down	CCDC27
GRIK1	11.8563136	-1.36103369	0.27400809	-4.96712953	6.80E-07	1.53E-06	Down	GRIK1
NHLRC3	1402.78174	0.51579545	0.10384459	4.96699379	6.80E-07	1.53E-06	Up	NHLRC3
CD53	928.057561	-0.82860702	0.16683003	-4.96677383	6.81E-07	1.53E-06	Down	CD53
CEACAM21	38.128262	-0.74141206	0.149306	-4.96572183	6.84E-07	1.54E-06	Down	CEACAM21
TENM2	21.4615604	-1.29815295	0.26144224	-4.96535284	6.86E-07	1.54E-06	Down	TENM2
DPY19L2	18.5466828	-1.18990021	0.23964625	-4.96523609	6.86E-07	1.54E-06	Down	DPY19L2
LOXL1	569.436605	0.78221175	0.15757042	4.96420417	6.90E-07	1.55E-06	Up	LOXL1
DRD5	10.5738036	-2.61757584	0.52737732	-4.9633834	6.93E-07	1.55E-06	Down	DRD5
ADAM15	7297.36615	0.53456418	0.10773966	4.96162883	6.99E-07	1.57E-06	Up	ADAM15
ARID5A	974.139928	0.50242348	0.10130092	4.95971271	7.06E-07	1.58E-06	Up	ARID5A
MUC17	2948.14494	1.75103905	0.35305624	4.95966035	7.06E-07	1.58E-06	Up	MUC17
DDRGK1	2506.39029	0.51532656	0.10391129	4.95929302	7.08E-07	1.59E-06	Up	DDRGK1
TMEM169	15.9898356	-0.84698126	0.17079053	-4.95918164	7.08E-07	1.59E-06	Down	TMEM169
SLC2A7	1.11411114	-1.2386376	0.24977201	-4.95907295	7.08E-07	1.59E-06	Down	SLC2A7

DDX58	637.088118	-0.57015008	0.11497388	-4.95895296	7.09E-07	1.59E-06	Down	DDX58
NBPF19	114.836795	-0.59278166	0.11955054	-4.95841879	7.11E-07	1.59E-06	Down	NBPF19
LRIF1	543.004051	0.5755242	0.11607281	4.95830354	7.11E-07	1.59E-06	Up	LRIF1
COX8A	5873.061	-0.51233105	0.10343241	-4.95329332	7.30E-07	1.64E-06	Down	COX8A
GPC6	359.821807	-0.90238696	0.18223393	-4.95180534	7.35E-07	1.65E-06	Down	GPC6
CSAD	367.034975	0.58353245	0.11786762	4.95074436	7.39E-07	1.66E-06	Up	CSAD
EPS8L1	1305.06682	0.58562317	0.1183089	4.94995019	7.42E-07	1.66E-06	Up	EPS8L1
MAGEC1	2.92819481	4.40465479	0.89005716	4.94873247	7.47E-07	1.67E-06	Up	MAGEC1
CCDC124	2222.09335	0.52213071	0.10553274	4.94757104	7.51E-07	1.68E-06	Up	CCDC124
FAM186A	15.3335189	0.97631029	0.19734296	4.94727701	7.53E-07	1.68E-06	Up	FAM186A
BTNL2	0.47819361	-2.05056965	0.41452693	-4.94677067	7.55E-07	1.69E-06	Down	BTNL2
PRR15	4192.6938	-0.65379	0.13218932	-4.94586085	7.58E-07	1.70E-06	Down	PRR15
SLC47A2	6.47476144	-0.86386603	0.17467228	-4.94563881	7.59E-07	1.70E-06	Down	SLC47A2
ADAM32	104.281877	1.04089138	0.2105275	4.94420635	7.65E-07	1.71E-06	Up	ADAM32
PRRT2	72.6471672	-0.82728041	0.16732442	-4.94417032	7.65E-07	1.71E-06	Down	PRRT2
APBA3	653.80445	0.52907301	0.10703724	4.94288715	7.70E-07	1.72E-06	Up	APBA3
LAYN	176.352919	-0.76802889	0.15538936	-4.94260933	7.71E-07	1.72E-06	Down	LAYN
LEP	13.444271	-2.18489707	0.44206858	-4.94243919	7.72E-07	1.72E-06	Down	LEP
NFATC1	246.765401	-0.81757057	0.16543112	-4.94206039	7.73E-07	1.73E-06	Down	NFATC1
IL20	1.18680832	1.99141169	0.40315239	4.93960036	7.83E-07	1.75E-06	Up	IL20
IL13	3.27760107	1.60760867	0.32564849	4.93663791	7.95E-07	1.78E-06	Up	IL13
IL4I1	313.117818	0.904666	0.1832703	4.93623898	7.96E-07	1.78E-06	Up	IL4I1
FABP4	194.571639	-1.90532756	0.38624054	-4.93300773	8.10E-07	1.81E-06	Down	FABP4
CCDC168	2.5279595	1.4427691	0.29254216	4.93183312	8.15E-07	1.82E-06	Up	CCDC168
BANF2	1.08572985	2.43908671	0.494577	4.93166227	8.15E-07	1.82E-06	Up	BANF2
HNF1B	1007.45016	-0.56832579	0.11525282	-4.9311226	8.18E-07	1.82E-06	Down	HNF1B
HS6ST3	6.97997902	-1.78911422	0.3628966	-4.93009366	8.22E-07	1.83E-06	Down	HS6ST3
TGM4	2.33053281	-1.0555292	0.21410363	-4.92999202	8.22E-07	1.83E-06	Down	TGM4
FZD7	806.854515	-0.73838423	0.14985065	-4.92746757	8.33E-07	1.86E-06	Down	FZD7
APC2	127.148791	-0.60950928	0.12375725	-4.92503889	8.43E-07	1.88E-06	Down	APC2
IL2RB	452.792876	-0.7937858	0.16125428	-4.92257201	8.54E-07	1.90E-06	Down	IL2RB
SLC24A3	376.188865	-0.80898404	0.16435877	-4.922062	8.56E-07	1.91E-06	Down	SLC24A3
LAMA3	3085.20451	-0.79603283	0.16177393	-4.92064974	8.63E-07	1.92E-06	Down	LAMA3
PIH1D2	31.6406433	0.55399155	0.11267579	4.91668673	8.80E-07	1.96E-06	Up	PIH1D2
PCDH9	43.8432459	-1.46513686	0.2980326	-4.91602889	8.83E-07	1.97E-06	Down	PCDH9
KAAG1	2.7698855	1.74015596	0.35410808	4.91419452	8.91E-07	1.99E-06	Up	KAAG1
PPP1R32	53.178678	0.61237851	0.12463831	4.91324452	8.96E-07	1.99E-06	Up	PPP1R32
HOXB9	4516.84614	0.86207435	0.17548558	4.91250814	8.99E-07	2.00E-06	Up	HOXB9
PCDH18	624.523785	-0.64393301	0.1310828	-4.91241423	9.00E-07	2.00E-06	Down	PCDH18
EFCAB8	7.10336039	1.17794689	0.23981113	4.91197751	9.02E-07	2.01E-06	Up	EFCAB8
CRYGS	58.8467979	0.59481881	0.1211134	4.91125515	9.05E-07	2.01E-06	Up	CRYGS
SLC26A7	11.5731892	-1.15796931	0.23582571	-4.91027592	9.09E-07	2.02E-06	Down	SLC26A7
RPGRIP1	12.8131651	-0.5705469	0.1162003	-4.91002937	9.11E-07	2.03E-06	Down	RPGRIP1
NOTCH1	5160.23643	0.57853133	0.11783669	4.90960255	9.13E-07	2.03E-06	Up	NOTCH1
HMX2	3.57963984	-1.88375347	0.38370225	-4.90941471	9.13E-07	2.03E-06	Down	HMX2
GCNT7	10.0841145	1.13946493	0.23212529	4.90883585	9.16E-07	2.04E-06	Up	GCNT7
IFI44	410.4639	0.90278994	0.18392169	4.90855618	9.17E-07	2.04E-06	Up	IFI44
ESPN	1304.10307	-0.86867797	0.17704676	-4.90648887	9.27E-07	2.06E-06	Down	ESPN
GOLGA6L9	86.4118776	0.63110049	0.12864812	4.90563305	9.31E-07	2.07E-06	Up	GOLGA6L9
KIF27	103.958538	0.50766882	0.10352526	4.9038158	9.40E-07	2.09E-06	Up	KIF27
ZNF385C	59.7137113	0.75843602	0.15469443	4.90280093	9.45E-07	2.10E-06	Up	ZNF385C
NRCAM	167.967394	1.21222864	0.24731521	4.90155314	9.51E-07	2.11E-06	Up	NRCAM
PCDHB5	19.4180912	-1.03001658	0.21016939	-4.90088772	9.54E-07	2.12E-06	Down	PCDHB5
NID2	661.131453	0.71559929	0.14601656	4.90080924	9.54E-07	2.12E-06	Up	NID2
SOX21	1.98511346	2.84122275	0.57981444	4.90022766	9.57E-07	2.12E-06	Up	SOX21
UGT2B4	5.09303108	2.50751284	0.51194443	4.89801762	9.68E-07	2.15E-06	Up	UGT2B4
SYTL4	655.237159	-0.55561267	0.1134702	-4.89655142	9.75E-07	2.16E-06	Down	SYTL4
RPS6	100629.076	0.5418218	0.11066716	4.89595822	9.78E-07	2.17E-06	Up	RPS6

CXorf65	14.4844627	0.87750676	0.1792423	4.8956455	9.80E-07	2.17E-06	Up	CXorf65
RNASE4	92.3940417	-0.66592809	0.13610589	-4.89272052	9.95E-07	2.20E-06	Down	RNASE4
FMN1	950.180098	-0.59751243	0.12222327	-4.88869597	1.02E-06	2.25E-06	Down	FMN1
FCAMR	84.2621452	-0.89046647	0.18215931	-4.88839405	1.02E-06	2.25E-06	Down	FCAMR
PFKFB2	2445.76527	-0.5698665	0.11662759	-4.88620642	1.03E-06	2.28E-06	Down	PFKFB2
REM1	32.662586	-0.81760334	0.16733486	-4.88603124	1.03E-06	2.28E-06	Down	REM1
DNPH1	2127.97586	0.59225597	0.12121749	4.88589517	1.03E-06	2.28E-06	Up	DNPH1
OVOL3	3.60189851	1.14522143	0.23440099	4.88573623	1.03E-06	2.28E-06	Up	OVOL3
MCHR2	0.58091454	-2.94894544	0.60358229	-4.88573885	1.03E-06	2.28E-06	Down	MCHR2
ZNF879	46.8580182	-0.75701095	0.15495258	-4.88543621	1.03E-06	2.28E-06	Down	ZNF879
S100A14	6053.36306	-0.72936143	0.14937253	-4.88283493	1.05E-06	2.31E-06	Down	S100A14
MDN1	2001.78462	0.51756034	0.10602149	4.88165503	1.05E-06	2.33E-06	Up	MDN1
SOAT2	8.06825939	1.93827983	0.39714032	4.88059191	1.06E-06	2.34E-06	Up	SOAT2
CLEC18B	5.06589131	1.17972527	0.2417551	4.87983619	1.06E-06	2.35E-06	Up	CLEC18B
POMGNT2	640.282953	0.52340538	0.10726032	4.87976712	1.06E-06	2.35E-06	Up	POMGNT2
ALAS2	7.12660335	-1.33482015	0.27356124	-4.87941979	1.06E-06	2.35E-06	Down	ALAS2
IQUB	7.35127886	1.05744887	0.21681499	4.87719447	1.08E-06	2.38E-06	Up	IQUB
FAM221A	358.196406	0.68035742	0.1395453	4.87553099	1.09E-06	2.40E-06	Up	FAM221A
CDH7	9.84895995	1.89788556	0.38931514	4.87493382	1.09E-06	2.40E-06	Up	CDH7
GPC4	2419.70382	0.55156291	0.113145	4.87483261	1.09E-06	2.40E-06	Up	GPC4
PLEKHH2	142.979593	-0.89889733	0.18448079	-4.87257954	1.10E-06	2.43E-06	Down	PLEKHH2
ATF7IP2	325.090629	0.74736736	0.15343029	4.87105479	1.11E-06	2.45E-06	Up	ATF7IP2
NUTM2G	9.52672651	0.92754792	0.19048355	4.86943836	1.12E-06	2.47E-06	Up	NUTM2G
MRGPRX2	0.54050181	-3.16574367	0.65015374	-4.86922321	1.12E-06	2.47E-06	Down	MRGPRX2
RHOBTB1	555.996943	0.55126975	0.11324122	4.86810129	1.13E-06	2.48E-06	Up	RHOBTB1
SOCS1	278.227806	0.91348672	0.18769076	4.86697752	1.13E-06	2.50E-06	Up	SOCS1
FXYD7	3.41377603	-0.94359061	0.19388799	-4.86667899	1.13E-06	2.50E-06	Down	FXYD7
FCGR3A	1021.7167	1.0083921	0.20723025	4.8660469	1.14E-06	2.51E-06	Up	FCGR3A
FAM9A	1.22466619	3.27496967	0.67303789	4.8659514	1.14E-06	2.51E-06	Up	FAM9A
B3GNT6	636.575155	-1.77422631	0.36463692	-4.86573419	1.14E-06	2.51E-06	Down	B3GNT6
RPS7	24342.6948	0.52396907	0.10769456	4.86532549	1.14E-06	2.52E-06	Up	RPS7
CRYBB1	12.7655544	-0.92423285	0.18996521	-4.86527437	1.14E-06	2.52E-06	Down	CRYBB1
LIPI	1.78026423	-1.60936917	0.33093455	-4.86310414	1.16E-06	2.54E-06	Down	LIPI
ZMYND10	21.8003104	0.89989449	0.18505466	4.8628577	1.16E-06	2.55E-06	Up	ZMYND10
UBE2V1	377.679279	0.53150276	0.10935563	4.86031452	1.17E-06	2.58E-06	Up	UBE2V1
ORAI1	1080.05621	0.51725121	0.10643126	4.85995575	1.17E-06	2.58E-06	Up	ORAI1
PLAT	2003.55139	-0.64656359	0.13306352	-4.85905968	1.18E-06	2.59E-06	Down	PLAT
SULT1E1	27.9520843	1.80771213	0.37205054	4.85878106	1.18E-06	2.60E-06	Up	SULT1E1
UNC93B1	2412.85379	0.54806008	0.11280662	4.85840332	1.18E-06	2.60E-06	Up	UNC93B1
KCNQ4	145.017623	-0.92554285	0.19063686	-4.85500482	1.20E-06	2.65E-06	Down	KCNQ4
TCHH	14.6751564	1.04458414	0.21517467	4.85458701	1.21E-06	2.65E-06	Up	TCHH
FAM149A	229.152438	-0.86397716	0.17809888	-4.85110942	1.23E-06	2.70E-06	Down	FAM149A
VEPH1	28.5700593	-1.05439438	0.21735536	-4.8510162	1.23E-06	2.70E-06	Down	VEPH1
ZNF655	1219.60128	-0.7100797	0.14640621	-4.85006539	1.23E-06	2.71E-06	Down	ZNF655
CTF1	59.918814	-0.95258502	0.19656094	-4.84625796	1.26E-06	2.76E-06	Down	CTF1
MEOX2	46.6036654	-1.35207465	0.27900504	-4.84605815	1.26E-06	2.77E-06	Down	MEOX2
ACSS1	3105.27871	0.61962432	0.12798404	4.84141854	1.29E-06	2.83E-06	Up	ACSS1
TAS2R3	3.01202965	2.65956613	0.5493832	4.84100376	1.29E-06	2.84E-06	Up	TAS2R3
CLTB	4556.95515	-0.53981408	0.11156181	-4.83869934	1.31E-06	2.87E-06	Down	CLTB
SPINK7	1.78818587	2.45417359	0.5074181	4.83659059	1.32E-06	2.90E-06	Up	SPINK7
ALX1	5.20894869	2.89675533	0.59914533	4.83481254	1.33E-06	2.92E-06	Up	ALX1
ANKRD6	80.7000236	-0.80062057	0.16560679	-4.83446694	1.34E-06	2.93E-06	Down	ANKRD6
GOLGA8S	0.9886054	-1.55404739	0.32153745	-4.83317689	1.34E-06	2.95E-06	Down	GOLGA8S
CDA	623.205395	-0.94642539	0.19594791	-4.82998467	1.37E-06	2.99E-06	Down	CDA
CBLN1	72.7885833	1.4293242	0.29611425	4.82693489	1.39E-06	3.04E-06	Up	CBLN1
PYGO1	82.1283952	-1.03258558	0.21396977	-4.82584797	1.39E-06	3.05E-06	Down	PYGO1
NKX2-2	7.63305174	-1.82554136	0.37833792	-4.82516094	1.40E-06	3.06E-06	Down	NKX2-2
PRB2	2.14088614	2.42698715	0.50303761	4.82466338	1.40E-06	3.07E-06	Up	PRB2

SOD3	3760.1222	1.04648537	0.2171891	4.8183144	1.45E-06	3.17E-06	Up	SOD3
SH2D4B	2.25137451	1.3840145	0.28728403	4.8175825	1.45E-06	3.18E-06	Up	SH2D4B
AQP12A	6.53213777	-1.22867041	0.25506897	-4.81701255	1.46E-06	3.19E-06	Down	AQP12A
CCER2	7.74906061	1.05057402	0.21814081	4.81603603	1.46E-06	3.20E-06	Up	CCER2
S100A9	1298.56438	1.05311682	0.21868052	4.81577793	1.47E-06	3.21E-06	Up	S100A9
SCAMP5	469.799827	-0.78857115	0.16376604	-4.81523007	1.47E-06	3.22E-06	Down	SCAMP5
PRRT4	10.6717206	-1.4023539	0.29135053	-4.81328763	1.48E-06	3.25E-06	Down	PRRT4
C5orf52	40.5937583	-0.99086684	0.20587883	-4.81286413	1.49E-06	3.25E-06	Down	C5orf52
NMRK2	1.34310353	-2.20012795	0.4572625	-4.81152061	1.50E-06	3.27E-06	Down	NMRK2
KYNU	171.577404	0.93279644	0.19387546	4.81131771	1.50E-06	3.28E-06	Up	KYNU
ACSM3	632.910189	-0.74472835	0.15478991	-4.81122022	1.50E-06	3.28E-06	Down	ACSM3
AIF1L	256.117938	-0.88232666	0.18339345	-4.81111336	1.50E-06	3.28E-06	Down	AIF1L
KRT3	1.17762732	2.61398378	0.54339636	4.81045509	1.51E-06	3.29E-06	Up	KRT3
RFX3	398.144203	0.53424858	0.11111686	4.80798838	1.52E-06	3.33E-06	Up	RFX3
UPK3A	73.3947315	1.4004373	0.29136898	4.80640498	1.54E-06	3.36E-06	Up	UPK3A
RAB33A	23.3788886	-0.68429635	0.14242483	-4.80461406	1.55E-06	3.39E-06	Down	RAB33A
CEACAM18	21.7435079	1.99988829	0.41634487	4.8034416	1.56E-06	3.41E-06	Up	CEACAM18
IL1B	805.440266	1.09649932	0.22835517	4.80172758	1.57E-06	3.43E-06	Up	IL1B
FRMPD2	2.91848128	2.29112297	0.47718771	4.80130338	1.58E-06	3.44E-06	Up	FRMPD2
LY75-CD302	2.5462865	-0.92899794	0.1935062	-4.8008691	1.58E-06	3.45E-06	Down	LY75-CD302
TENM4	157.524336	0.93774617	0.19540851	4.79890135	1.60E-06	3.48E-06	Up	TENM4
TTBK2	242.974567	-0.58726642	0.12244746	-4.79606868	1.62E-06	3.53E-06	Down	TTBK2
NKX6-1	2.24949249	2.71449842	0.5659869	4.79604464	1.62E-06	3.53E-06	Up	NKX6-1
AZIN2	180.623077	-0.64315615	0.1341535	-4.794181	1.63E-06	3.56E-06	Down	AZIN2
DZANK1	103.29324	0.57152125	0.11928753	4.79112315	1.66E-06	3.62E-06	Up	DZANK1
ELL3	79.7240628	-0.52071535	0.10873005	-4.78906575	1.68E-06	3.65E-06	Down	ELL3
CNDP1	12.9017252	1.65105941	0.34477866	4.78875173	1.68E-06	3.66E-06	Up	CNDP1
DNAJC15	3175.06344	0.57340522	0.11978593	4.78691614	1.69E-06	3.69E-06	Up	DNAJC15
MSH4	10.4238582	1.40716862	0.29400206	4.78625424	1.70E-06	3.70E-06	Up	MSH4
GULP1	333.558973	-0.9183577	0.19193997	-4.78460891	1.71E-06	3.73E-06	Down	GULP1
XCR1	37.158417	-1.19457861	0.24973267	-4.78342943	1.72E-06	3.75E-06	Down	XCR1
TDRD12	6.97423544	-1.02640302	0.21458015	-4.78330835	1.72E-06	3.75E-06	Down	TDRD12
FGF4	3.21296955	1.67124662	0.34948207	4.78206687	1.74E-06	3.77E-06	Up	FGF4
ERICH6B	13.4165449	0.81281555	0.17003295	4.78034148	1.75E-06	3.81E-06	Up	ERICH6B
DUOX1	166.224666	0.82452293	0.17260447	4.77694996	1.78E-06	3.87E-06	Up	DUOX1
ANKLE1	27.4653958	1.02073341	0.21375167	4.77532361	1.79E-06	3.90E-06	Up	ANKLE1
PCDHGA9	30.6366799	-0.81982396	0.17170306	-4.77466138	1.80E-06	3.91E-06	Down	PCDHGA9
CEP85L	94.7197467	-0.85571721	0.17922821	-4.77445617	1.80E-06	3.91E-06	Down	CEP85L
NRBP2	925.348154	0.56668079	0.11871381	4.77350341	1.81E-06	3.93E-06	Up	NRBP2
TMEM106A	169.473607	-0.60482135	0.12674576	-4.77192551	1.82E-06	3.96E-06	Down	TMEM106A
TMEM221	6.89758596	-0.75631939	0.15849466	-4.77189191	1.83E-06	3.96E-06	Down	TMEM221
FES	322.534471	-0.61240139	0.12835791	-4.77104506	1.83E-06	3.98E-06	Down	FES
HRAS	1083.68795	0.54660913	0.11457766	4.77064328	1.84E-06	3.99E-06	Up	HRAS
CPT1B	59.114045	0.76855918	0.16114228	4.76944458	1.85E-06	4.01E-06	Up	CPT1B
KHDRBS3	534.095041	0.73913062	0.15498167	4.7691487	1.85E-06	4.02E-06	Up	KHDRBS3
LINGO4	5.28000065	-1.04366606	0.21886507	-4.76853654	1.86E-06	4.03E-06	Down	LINGO4
RSRP1	1721.73282	0.50497003	0.10589758	4.76847552	1.86E-06	4.03E-06	Up	RSRP1
CD3D	184.761189	-0.7975661	0.16726267	-4.7683449	1.86E-06	4.03E-06	Down	CD3D
EPHB6	98.1691821	-0.97799989	0.20516209	-4.766962	1.87E-06	4.06E-06	Down	EPHB6
FOS	10506.7252	-0.85877901	0.18016618	-4.76659397	1.87E-06	4.06E-06	Down	FOS
PTPRS	482.239909	-0.84516084	0.17733258	-4.76596479	1.88E-06	4.08E-06	Down	PTPRS
MYH6	1.45829693	2.51154408	0.52702628	4.76550069	1.88E-06	4.08E-06	Up	MYH6
RASGRF1	63.061762	1.0512088	0.22060901	4.76503123	1.89E-06	4.09E-06	Up	RASGRF1
ZDHHC2	823.959188	-0.76599361	0.16077499	-4.764383	1.89E-06	4.11E-06	Down	ZDHHC2
KNDC1	44.8965027	1.16097783	0.24371935	4.76358502	1.90E-06	4.12E-06	Up	KNDC1
TMEM178B	193.978083	1.35023013	0.28349523	4.76279666	1.91E-06	4.14E-06	Up	TMEM178B
LYPLA1	3880.85964	0.56350231	0.11831636	4.76267462	1.91E-06	4.14E-06	Up	LYPLA1
ZNF331	264.90792	-0.8006296	0.16813319	-4.7618771	1.92E-06	4.15E-06	Down	ZNF331

NPTXR	221.451474	-1.12334853	0.23614162	-4.75709681	1.96E-06	4.25E-06	Down	NPTXR
SOSTDC1	238.404676	-1.20833332	0.2540098	-4.75703421	1.96E-06	4.25E-06	Down	SOSTDC1
MYH2	2.22438748	-2.44423901	0.51400126	-4.75531718	1.98E-06	4.29E-06	Down	MYH2
XKR9	91.8559847	1.23712368	0.26020548	4.75441057	1.99E-06	4.31E-06	Up	XKR9
KBTBD13	0.36060344	-1.7077982	0.35925649	-4.75370181	2.00E-06	4.32E-06	Down	KBTBD13
RAB34	519.064672	-0.69191486	0.14555584	-4.75360425	2.00E-06	4.32E-06	Down	RAB34
SAP25	5.32412868	0.9842442	0.20705531	4.75353272	2.00E-06	4.32E-06	Up	SAP25
CRB1	1.82012606	-1.56904975	0.33018606	-4.75201695	2.01E-06	4.36E-06	Down	CRB1
FAM43A	508.087986	-0.63271019	0.13317136	-4.7510979	2.02E-06	4.37E-06	Down	FAM43A
CHCHD10	2319.27177	-0.67105836	0.14126401	-4.75038443	2.03E-06	4.39E-06	Down	CHCHD10
STXBP5L	16.5503321	-1.67263976	0.35218357	-4.74934074	2.04E-06	4.41E-06	Down	STXBP5L
OLFM2	202.878558	0.89972311	0.18947165	4.7485895	2.05E-06	4.43E-06	Up	OLFM2
TP53I13	1400.57618	0.54955203	0.11575627	4.74749241	2.06E-06	4.45E-06	Up	TP53I13
NPBWR1	2.16211566	1.87881292	0.39579086	4.74698406	2.06E-06	4.46E-06	Up	NPBWR1
APBA2	152.879569	0.7456314	0.15707915	4.74685149	2.07E-06	4.46E-06	Up	APBA2
CYP27C1	16.164575	-0.98423247	0.20743951	-4.74467221	2.09E-06	4.51E-06	Down	CYP27C1
LRRC8C	275.752528	-0.64614849	0.13621003	-4.74376591	2.10E-06	4.53E-06	Down	LRRC8C
DUSP5	932.387451	-0.75985184	0.1603148	-4.73974848	2.14E-06	4.62E-06	Down	DUSP5
APOL6	3021.24581	-0.53013657	0.11189286	-4.73789444	2.16E-06	4.66E-06	Down	APOL6
MAS1	1.29243698	2.38960211	0.50438982	4.73760975	2.16E-06	4.67E-06	Up	MAS1
TMEM190	3.73937558	1.20612399	0.25470413	4.73539231	2.19E-06	4.71E-06	Up	TMEM190
GOLGA8K	0.74731174	-1.32532614	0.27993104	-4.73447369	2.20E-06	4.74E-06	Down	GOLGA8K
PLEKHD1	4.31491743	1.1316778	0.23909815	4.7331099	2.21E-06	4.77E-06	Up	PLEKHD1
GPR39	366.609052	-0.54943725	0.1160878	-4.73294576	2.21E-06	4.77E-06	Down	GPR39
OTUD3	535.267726	0.53475322	0.11300073	4.73229896	2.22E-06	4.78E-06	Up	OTUD3
ADAMTS9	491.100319	0.61161106	0.12927937	4.73092556	2.23E-06	4.82E-06	Up	ADAMTS9
TMEM215	2.34540662	1.79630395	0.37977233	4.72994947	2.25E-06	4.84E-06	Up	TMEM215
MAPK12	197.456892	0.84470821	0.17860284	4.72953389	2.25E-06	4.85E-06	Up	MAPK12
THRSP	7.3089265	-1.6976231	0.3590083	-4.72864579	2.26E-06	4.87E-06	Down	THRSP
CNPY1	1.30895618	2.67765761	0.56636316	4.72781035	2.27E-06	4.89E-06	Up	CNPY1
KANSL1L	418.257552	0.55175266	0.11675425	4.72576079	2.29E-06	4.93E-06	Up	KANSL1L
STS	774.110257	-0.64342038	0.13616322	-4.72536124	2.30E-06	4.94E-06	Down	STS
SH3GL3	1.6047745	-2.16375995	0.45794737	-4.72490964	2.30E-06	4.95E-06	Down	SH3GL3
PGPEP1L	1.37017735	-2.06029298	0.43621549	-4.72310828	2.32E-06	4.99E-06	Down	PGPEP1L
UBASH3A	48.2920675	-0.76656223	0.16233742	-4.72203042	2.34E-06	5.02E-06	Down	UBASH3A
ZNF354C	59.1699846	-0.92673472	0.19628488	-4.72137595	2.34E-06	5.03E-06	Down	ZNF354C
KLKB1	13.1658756	-0.9224584	0.1953832	-4.72127794	2.34E-06	5.04E-06	Down	KLKB1
ACOT1	106.699848	-0.67581481	0.14319856	-4.71942453	2.37E-06	5.08E-06	Down	ACOT1
PIM3	3647.08218	0.51642461	0.10942801	4.71930898	2.37E-06	5.08E-06	Up	PIM3
SLC34A1	1.9814596	1.37320637	0.29103923	4.71828622	2.38E-06	5.11E-06	Up	SLC34A1
QRICH2	89.3789229	0.62619107	0.13275736	4.71680871	2.40E-06	5.14E-06	Up	QRICH2
IFIT2	339.523571	-0.78331095	0.16629052	-4.71049678	2.47E-06	5.30E-06	Down	IFIT2
TAF1L	2.04773496	1.39099735	0.29529737	4.71049686	2.47E-06	5.30E-06	Up	TAF1L
EVC	249.900002	-0.86369545	0.18337138	-4.71008872	2.48E-06	5.31E-06	Down	EVC
SMTNL1	10.7834213	1.15785165	0.24582412	4.71008152	2.48E-06	5.31E-06	Up	SMTNL1
OXT	2.86640481	1.45355582	0.30863872	4.70957061	2.48E-06	5.33E-06	Up	OXT
RAB7B	93.3751727	-0.65063572	0.13818626	-4.70839669	2.50E-06	5.35E-06	Down	RAB7B
ZNF219	1508.49952	-0.58277817	0.12377787	-4.70825828	2.50E-06	5.36E-06	Down	ZNF219
ZNF891	189.110677	0.68374694	0.14527584	4.7065427	2.52E-06	5.40E-06	Up	ZNF891
NR2F1	489.880199	0.71374845	0.15167978	4.70562701	2.53E-06	5.42E-06	Up	NR2F1
PFKFB4	532.625914	0.6442789	0.13693392	4.70503517	2.54E-06	5.44E-06	Up	PFKFB4
ALDOB	1316.39241	1.29901087	0.27613963	4.70418131	2.55E-06	5.46E-06	Up	ALDOB
ZNF311	105.735059	0.50812933	0.10805094	4.70268333	2.57E-06	5.50E-06	Up	ZNF311
PHYHIPL	89.92855	1.68674638	0.35884546	4.70048127	2.60E-06	5.56E-06	Up	PHYHIPL
ARNT2	232.679279	-0.95335726	0.20287896	-4.69914316	2.61E-06	5.59E-06	Down	ARNT2
MUC4	3597.93966	-1.18587012	0.25242068	-4.69799116	2.63E-06	5.62E-06	Down	MUC4
MTTP	219.42521	1.87496331	0.39916778	4.69718105	2.64E-06	5.64E-06	Up	MTTP
KLF7	793.88548	0.62918375	0.13395959	4.69681755	2.64E-06	5.65E-06	Up	KLF7

CERKL	190.843304	-0.58351799	0.12424816	-4.6963914	2.65E-06	5.66E-06	Down	CERKL
AKR7A3	1120.58077	-0.68502347	0.145937	-4.69396704	2.68E-06	5.73E-06	Down	AKR7A3
RAET1E	22.4750915	0.92972606	0.19808823	4.69349476	2.69E-06	5.74E-06	Up	RAET1E
C15orf61	330.146678	0.54909827	0.11711289	4.68862388	2.75E-06	5.88E-06	Up	C15orf61
KRT36	12.3026169	1.35093351	0.28813594	4.68852825	2.75E-06	5.88E-06	Up	KRT36
SGCD	274.184453	-0.92530085	0.19735642	-4.6884761	2.75E-06	5.88E-06	Down	SGCD
ZNF75A	282.623907	-0.55052017	0.11742587	-4.68823592	2.76E-06	5.89E-06	Down	ZNF75A
TNFRSF9	102.873927	0.79109214	0.16875143	4.68791374	2.76E-06	5.90E-06	Up	TNFRSF9
LETM2	57.1530849	0.693359	0.14802732	4.68399342	2.81E-06	6.01E-06	Up	LETM2
GABRR1	16.1412023	2.12195645	0.45314379	4.6827442	2.83E-06	6.04E-06	Up	GABRR1
TTC34	16.398874	0.84030344	0.17948691	4.68169763	2.85E-06	6.07E-06	Up	TTC34
RNLS	250.719345	-0.75164413	0.16062843	-4.67939651	2.88E-06	6.14E-06	Down	RNLS
PIK3R6	52.942631	-0.67302098	0.14382651	-4.67939442	2.88E-06	6.14E-06	Down	PIK3R6
ALDOC	1369.408	0.6892056	0.14738262	4.67630164	2.92E-06	6.23E-06	Up	ALDOC
SAGE1	1.96430281	3.71618521	0.79481252	4.67554943	2.93E-06	6.25E-06	Up	SAGE1
GPR26	1.33466477	-2.23634683	0.47841594	-4.67448225	2.95E-06	6.28E-06	Down	GPR26
TIGD5	654.609402	0.5439389	0.11637445	4.67404063	2.95E-06	6.30E-06	Up	TIGD5
BIN1	2677.61758	0.50605254	0.108309	4.67230349	2.98E-06	6.35E-06	Up	BIN1
NNMT	1282.13856	0.84635848	0.18115908	4.67190751	2.98E-06	6.36E-06	Up	NNMT
SNTN	8.10464105	0.92332532	0.19765794	4.67132937	2.99E-06	6.38E-06	Up	SNTN
AVIL	228.298391	-0.66674302	0.14274566	-4.6708463	3.00E-06	6.39E-06	Down	AVIL
CT83	8.35741706	4.88589658	1.04617313	4.67025621	3.01E-06	6.41E-06	Up	CT83
PIPOX	262.38685	1.29658343	0.27764626	4.66991143	3.01E-06	6.42E-06	Up	PIPOX
ENDOU	4.19985952	-0.98462035	0.21093057	-4.66798319	3.04E-06	6.48E-06	Down	ENDOU
ANKRD55	4.52810149	-0.97489536	0.20886856	-4.66750647	3.05E-06	6.49E-06	Down	ANKRD55
MRGPRD	0.52637954	-2.8483475	0.61024986	-4.66751027	3.05E-06	6.49E-06	Down	MRGPRD
RRH	4.84856707	0.9754772	0.20906108	4.66599136	3.07E-06	6.54E-06	Up	RRH
PACSIN3	668.282554	0.82846779	0.17755884	4.66587738	3.07E-06	6.54E-06	Up	PACSIN3
SYT2	21.3245298	-0.92351815	0.19794792	-4.66546013	3.08E-06	6.55E-06	Down	SYT2
ZNF347	184.331292	-0.76326251	0.16362389	-4.66473754	3.09E-06	6.57E-06	Down	ZNF347
KANK4	50.6867405	-1.09797196	0.23539957	-4.66429049	3.10E-06	6.59E-06	Down	KANK4
CD7	271.238859	-0.93404415	0.2003399	-4.66229719	3.13E-06	6.65E-06	Down	CD7
CDC20B	2.01978685	1.91276209	0.41036074	4.66117219	3.14E-06	6.68E-06	Up	CDC20B
FREM1	668.931711	1.24663574	0.26766163	4.65750629	3.20E-06	6.80E-06	Up	FREM1
TENM1	15.8725367	-1.49213925	0.32050271	-4.65562136	3.23E-06	6.86E-06	Down	TENM1
ALDH1A1	2956.6291	-1.04851261	0.22525822	-4.6547141	3.24E-06	6.89E-06	Down	ALDH1A1
GUCA1A	4.52735056	1.35801137	0.29179926	4.65392327	3.26E-06	6.91E-06	Up	GUCA1A
PKN1	3204.72071	0.52212249	0.11221256	4.65297716	3.27E-06	6.94E-06	Up	PKN1
MUSTN1	0.91983649	-1.00734016	0.21654497	-4.65187521	3.29E-06	6.98E-06	Down	MUSTN1
ARTN	70.774564	0.68362302	0.14711176	4.6469639	3.37E-06	7.14E-06	Up	ARTN
ZNF488	114.755484	1.00666116	0.2166393	4.64671525	3.37E-06	7.15E-06	Up	ZNF488
SDF2L1	1528.81935	0.71030173	0.15290768	4.64529784	3.40E-06	7.20E-06	Up	SDF2L1
KCNG1	85.5437324	-1.27375176	0.27425291	-4.64444205	3.41E-06	7.22E-06	Down	KCNG1
SLC40A1	8546.52794	-0.65712325	0.14152743	-4.64308064	3.43E-06	7.27E-06	Down	SLC40A1
CD38	199.583773	-0.97135243	0.20923688	-4.64235776	3.44E-06	7.29E-06	Down	CD38
ZNF771	214.834028	0.67254962	0.14490478	4.64132122	3.46E-06	7.33E-06	Up	ZNF771
HOXD3	14.6083167	-0.97932262	0.21108984	-4.63936414	3.49E-06	7.40E-06	Down	HOXD3
KLF15	58.2690379	-0.9826206	0.21204245	-4.634075	3.59E-06	7.58E-06	Down	KLF15
FETUB	2.08858163	-2.12112167	0.45783189	-4.6329706	3.60E-06	7.62E-06	Down	FETUB
ZPBP	1.13155886	2.47569833	0.5343778	4.63286152	3.61E-06	7.63E-06	Up	ZPBP
TBC1D10C	202.099417	-0.74551572	0.16094939	-4.63198852	3.62E-06	7.66E-06	Down	TBC1D10C
GDF6	12.892202	-1.26318968	0.27274429	-4.63140646	3.63E-06	7.68E-06	Down	GDF6
CABP4	51.8684176	0.96336629	0.20806966	4.63001817	3.66E-06	7.73E-06	Up	CABP4
RAB11FIP1	4549.22881	-0.5225045	0.1128629	-4.62955061	3.66E-06	7.74E-06	Down	RAB11FIP1
MC3R	0.97860758	2.69466922	0.58245038	4.62643565	3.72E-06	7.86E-06	Up	MC3R
PYHIN1	32.9514496	-0.8866177	0.19167504	-4.6256293	3.73E-06	7.89E-06	Down	PYHIN1
SLX1B	1.81506724	1.38343709	0.29909418	4.62542304	3.74E-06	7.89E-06	Up	SLX1B
ZSCAN10	1.55726777	2.05231674	0.44371324	4.62532229	3.74E-06	7.90E-06	Up	ZSCAN10

GSTM1	179.948108	-1.47104993	0.31807689	-4.62482499	3.75E-06	7.91E-06	Down	GSTM1
IRX6	2.05594647	-2.13264288	0.46115031	-4.62461549	3.75E-06	7.92E-06	Down	IRX6
C19orf73	50.4325678	0.55315896	0.11963375	4.62376994	3.77E-06	7.95E-06	Up	C19orf73
KCNA4	0.68340607	-2.52783063	0.54684937	-4.62253551	3.79E-06	8.00E-06	Down	KCNA4
MTRNR2L1	345.238226	-2.07223057	0.448297	-4.62245021	3.79E-06	8.00E-06	Down	MTRNR2L1
WFDC9	0.64742577	2.42078763	0.52371021	4.62238002	3.79E-06	8.00E-06	Up	WFDC9
TEX43	17.6755616	-1.11947936	0.24252087	-4.61601251	3.91E-06	8.24E-06	Down	TEX43
ARHGEF17	870.575588	-0.60853424	0.13185028	-4.61534262	3.92E-06	8.27E-06	Down	ARHGEF17
SLC6A16	16.5004038	-0.95406588	0.20671867	-4.61528639	3.93E-06	8.27E-06	Down	SLC6A16
GPR176	232.573341	0.6736284	0.14596657	4.61494977	3.93E-06	8.28E-06	Up	GPR176
PAK6	8.23814592	0.74210223	0.16080673	4.61487041	3.93E-06	8.28E-06	Up	PAK6
DEFA5	751.939273	2.05872241	0.4461757	4.61415179	3.95E-06	8.31E-06	Up	DEFA5
SUPT20HL1	0.77297687	-1.47405481	0.31951501	-4.61341334	3.96E-06	8.34E-06	Down	SUPT20HL1
CDH18	0.81166241	-2.37685037	0.51522286	-4.61324709	3.96E-06	8.35E-06	Down	CDH18
LMNTD2	123.037169	0.66738094	0.14470685	4.61195116	3.99E-06	8.40E-06	Up	LMNTD2
OR8D1	0.49912284	-3.24101523	0.70282728	-4.61139645	4.00E-06	8.42E-06	Down	OR8D1
ANXA9	297.700796	0.51170522	0.11099314	4.61024187	4.02E-06	8.46E-06	Up	ANXA9
FAM221B	13.2540853	1.00224397	0.21745948	4.60887682	4.05E-06	8.52E-06	Up	FAM221B
ERVH48-1	7.19021897	1.34925773	0.29275693	4.60879858	4.05E-06	8.52E-06	Up	ERVH48-1
MC2R	0.87581298	2.18344668	0.47380655	4.60830831	4.06E-06	8.54E-06	Up	MC2R
ZNF705A	0.95566673	2.04298428	0.4433454	4.60810975	4.06E-06	8.54E-06	Up	ZNF705A
HTR3C	5.80688972	-2.35367442	0.5111612	-4.60456389	4.13E-06	8.69E-06	Down	HTR3C
TSHB	0.53296589	-1.79549773	0.39004724	-4.60328278	4.16E-06	8.74E-06	Down	TSHB
PTPN22	152.018448	-0.68859328	0.14963678	-4.60176497	4.19E-06	8.80E-06	Down	PTPN22
ITK	119.186224	-0.82786562	0.17993134	-4.60100858	4.20E-06	8.83E-06	Down	ITK
MTRNR2L10	4.07956176	-1.09812106	0.23884278	-4.59767329	4.27E-06	8.97E-06	Down	MTRNR2L10
DCST1	17.2893005	0.66776212	0.14529716	4.59583748	4.31E-06	9.05E-06	Up	DCST1
CYTIP	307.747617	-0.74030827	0.1611329	-4.59439541	4.34E-06	9.11E-06	Down	CYTIP
PRODH	131.816113	1.18397948	0.25772219	4.59401456	4.35E-06	9.12E-06	Up	PRODH
SHF	463.039978	0.88436961	0.19251457	4.59378021	4.35E-06	9.13E-06	Up	SHF
ESR1	32.9903879	-1.01319258	0.2207538	-4.58969489	4.44E-06	9.31E-06	Down	ESR1
ZNF449	160.519426	0.50093599	0.10917462	4.58839244	4.47E-06	9.37E-06	Up	ZNF449
GADD45G	350.118574	0.80634312	0.17575174	4.58796673	4.48E-06	9.38E-06	Up	GADD45G
NMNAT3	361.247851	0.56653816	0.12362557	4.58269391	4.59E-06	9.61E-06	Up	NMNAT3
DCDC2B	7.33773179	1.12555891	0.24563854	4.58217557	4.60E-06	9.64E-06	Up	DCDC2B
STOML3	15.9465433	1.18479852	0.25857563	4.58201936	4.61E-06	9.64E-06	Up	STOML3
DRAXIN	23.3200655	0.86837048	0.18954353	4.58137759	4.62E-06	9.67E-06	Up	DRAXIN
PRH2	3.53230881	1.58728081	0.34650293	4.5808583	4.63E-06	9.69E-06	Up	PRH2
PRRX2	81.6983521	0.94994625	0.20740995	4.58004188	4.65E-06	9.73E-06	Up	PRRX2
BIRC3	1516.00371	-0.78368332	0.17111156	-4.57995545	4.65E-06	9.73E-06	Down	BIRC3
MROH2B	1.60769429	-2.57211103	0.56225917	-4.5746004	4.77E-06	9.98E-06	Down	MROH2B
CCDC87	10.6619658	-0.60297649	0.13181482	-4.57442108	4.78E-06	9.99E-06	Down	CCDC87
GCSAM	90.2102168	-0.81222613	0.17756442	-4.57426169	4.78E-06	9.99E-06	Down	GCSAM
ARHGAP6	237.46283	-0.96940129	0.21198871	-4.57289106	4.81E-06	1.01E-05	Down	ARHGAP6
GALNT14	33.777047	1.30506726	0.2854728	4.57159925	4.84E-06	1.01E-05	Up	GALNT14
MAGEE1	57.0355367	-0.91131316	0.1993578	-4.57124393	4.85E-06	1.01E-05	Down	MAGEE1
SYNDIG1	91.3367422	0.84747107	0.18559573	4.56622062	4.97E-06	1.04E-05	Up	SYNDIG1
RASGEF1A	127.856119	1.26432715	0.27703081	4.56385036	5.02E-06	1.05E-05	Up	RASGEF1A
DUSP13	0.89562869	2.14104934	0.46982055	4.5571641	5.18E-06	1.08E-05	Up	DUSP13
SULF2	5969.42368	0.5672861	0.12448888	4.55692199	5.19E-06	1.08E-05	Up	SULF2
KLRF2	0.67339314	-1.94158549	0.42608666	-4.55678547	5.19E-06	1.08E-05	Down	KLRF2
CDKL4	2.22435499	1.57401754	0.34544657	4.55647176	5.20E-06	1.08E-05	Up	CDKL4
INTS6	1352.59325	0.50734745	0.11144419	4.55247999	5.30E-06	1.11E-05	Up	INTS6
ZNF154	67.5242072	-0.83517666	0.18348506	-4.55174194	5.32E-06	1.11E-05	Down	ZNF154
ZNF83	738.643715	0.59851363	0.13157252	4.54892577	5.39E-06	1.12E-05	Up	ZNF83
NEUROD1	20.3760073	-2.36077799	0.51906822	-4.54810737	5.41E-06	1.13E-05	Down	NEUROD1
MAGEC2	3.3302224	4.52801441	0.99578458	4.54718272	5.44E-06	1.13E-05	Up	MAGEC2
APOBEC3D	118.565803	-0.65235101	0.14347246	-4.54687283	5.44E-06	1.13E-05	Down	APOBEC3D

EPHA2	3989.23588	0.5987102	0.13168439	4.54655414	5.45E-06	1.14E-05	Up	EPHA2
PCDHB1	1.16952553	-2.01318465	0.44297412	-4.54470038	5.50E-06	1.15E-05	Down	PCDHB1
HPD	9.78991862	-0.68929125	0.15170107	-4.54374675	5.53E-06	1.15E-05	Down	HPD
NRIP3	65.8148555	0.74514865	0.16401682	4.54312342	5.54E-06	1.15E-05	Up	NRIP3
UBN2	1076.25241	0.55083969	0.12128887	4.54155182	5.58E-06	1.16E-05	Up	UBN2
BRDT	0.91978485	2.12349951	0.4676134	4.5411434	5.59E-06	1.16E-05	Up	BRDT
DIRAS3	23.8739182	-0.83400003	0.18366229	-4.5409432	5.60E-06	1.16E-05	Down	DIRAS3
NRARP	1820.49182	-0.60873907	0.13408814	-4.5398427	5.63E-06	1.17E-05	Down	NRARP
IQSEC3	38.6571837	-0.87478068	0.19269631	-4.53968575	5.63E-06	1.17E-05	Down	IQSEC3
COL16A1	1590.70473	0.72852386	0.16050304	4.53900363	5.65E-06	1.18E-05	Up	COL16A1
LY75	1332.63281	0.51371591	0.11318912	4.5385627	5.66E-06	1.18E-05	Up	LY75
ANO2	20.1340591	-0.71740184	0.15807002	-4.53850662	5.67E-06	1.18E-05	Down	ANO2
KCTD4	11.9148484	-1.83347619	0.40404075	-4.53784962	5.68E-06	1.18E-05	Down	KCTD4
ENPP5	251.124888	0.66157742	0.14581692	4.53704159	5.70E-06	1.19E-05	Up	ENPP5
PRB3	5.11527297	1.08675288	0.23960424	4.53561629	5.74E-06	1.19E-05	Up	PRB3
OSGIN1	289.649298	-0.57698596	0.12722913	-4.53501472	5.76E-06	1.20E-05	Down	OSGIN1
NLRC5	2600.85151	0.56477576	0.12457365	4.53366952	5.80E-06	1.20E-05	Up	NLRC5
NUTM2F	1.20132073	1.69608484	0.37421818	4.53234214	5.83E-06	1.21E-05	Up	NUTM2F
FAM228B	80.6245259	-0.52374043	0.11557851	-4.53146889	5.86E-06	1.22E-05	Down	FAM228B
SLC27A1	598.655543	0.53885905	0.11899214	4.52852627	5.94E-06	1.23E-05	Up	SLC27A1
PIEZO2	117.135924	-0.84369301	0.18634971	-4.52747155	5.97E-06	1.24E-05	Down	PIEZO2
LMX1A	8.46564293	-1.92525319	0.4252551	-4.52729007	5.97E-06	1.24E-05	Down	LMX1A
MYOZ1	5.59789496	-0.86440173	0.19094686	-4.52692302	5.98E-06	1.24E-05	Down	MYOZ1
APCDD1L	24.0219329	-1.24172727	0.27430197	-4.52686236	5.99E-06	1.24E-05	Down	APCDD1L
MAP9	106.80798	-1.02957652	0.22749914	-4.52562813	6.02E-06	1.25E-05	Down	MAP9
ZG16B	1257.41038	0.75033812	0.16581929	4.52503528	6.04E-06	1.25E-05	Up	ZG16B
SLC7A8	2225.73344	0.69714715	0.15408526	4.52442452	6.06E-06	1.26E-05	Up	SLC7A8
OR2C1	1.07700333	-1.13226762	0.25032568	-4.52317798	6.09E-06	1.26E-05	Down	OR2C1
ADRB1	39.3532581	-1.07958502	0.2387553	-4.5217217	6.13E-06	1.27E-05	Down	ADRB1
TIMP2	6898.17897	-0.7103996	0.15711805	-4.52143838	6.14E-06	1.27E-05	Down	TIMP2
CDH16	58.8508203	1.63060016	0.36071507	4.52046587	6.17E-06	1.28E-05	Up	CDH16
PVALB	2.11526874	-1.21236173	0.26822139	-4.52000394	6.18E-06	1.28E-05	Down	PVALB
CAGE1	2.86705037	1.07983101	0.2389075	4.51987061	6.19E-06	1.28E-05	Up	CAGE1
CCDC106	200.935068	-0.62756156	0.13888239	-4.5186546	6.22E-06	1.29E-05	Down	CCDC106
PEAR1	173.025354	-0.59631506	0.13197395	-4.51843016	6.23E-06	1.29E-05	Down	PEAR1
WNT5B	210.45084	-0.77265011	0.17108832	-4.51608907	6.30E-06	1.30E-05	Down	WNT5B
NTN5	9.83054627	0.93535728	0.2073383	4.51126136	6.44E-06	1.33E-05	Up	NTN5
ACHE	439.722363	-0.93041523	0.20626117	-4.51085991	6.46E-06	1.34E-05	Down	ACHE
PNMT	10.8934063	1.14709998	0.25433321	4.51022492	6.48E-06	1.34E-05	Up	PNMT
TICAM2	3.22182324	-0.83803181	0.18592568	-4.50734843	6.56E-06	1.36E-05	Down	TICAM2
CACNG1	1.40890504	2.42558192	0.53824223	4.5064876	6.59E-06	1.36E-05	Up	CACNG1
MROH8	46.098779	-0.50420237	0.11191283	-4.50531347	6.63E-06	1.37E-05	Down	MROH8
TM6SF1	40.5794723	-0.76461095	0.1697622	-4.50401173	6.67E-06	1.38E-05	Down	TM6SF1
BCL2L10	39.6421145	-0.91728859	0.2037478	-4.50207844	6.73E-06	1.39E-05	Down	BCL2L10
APOA5	1.18165055	2.39854491	0.53278861	4.50186972	6.74E-06	1.39E-05	Up	APOA5
SIVA1	2732.98478	0.63163518	0.1403324	4.50099318	6.76E-06	1.40E-05	Up	SIVA1
SDSL	593.684127	-0.51510525	0.11453002	-4.49755678	6.87E-06	1.42E-05	Down	SDSL
SERPINE3	5.61520469	1.04826367	0.23310367	4.49698481	6.89E-06	1.42E-05	Up	SERPINE3
TFRC	14274.4596	0.54175281	0.12048351	4.49648935	6.91E-06	1.43E-05	Up	TFRC
CLDN10	22.4169462	1.99173319	0.44307712	4.49522913	6.95E-06	1.43E-05	Up	CLDN10
ZNF157	1.34895149	-1.29453646	0.2879993	-4.49492924	6.96E-06	1.44E-05	Down	ZNF157
CFAP46	12.1551938	1.38449397	0.30819885	4.49221006	7.05E-06	1.45E-05	Up	CFAP46
RINL	313.460871	0.54258236	0.1208161	4.49097732	7.09E-06	1.46E-05	Up	RINL
BGLAP	16.7308912	-0.71671483	0.15959731	-4.49077	7.10E-06	1.46E-05	Down	BGLAP
ERG	236.717411	-0.61592893	0.13716095	-4.49055601	7.10E-06	1.46E-05	Down	ERG
GLUD2	37.4972659	-0.55818662	0.12432544	-4.48972175	7.13E-06	1.47E-05	Down	GLUD2
CCDC83	1.76082703	2.39489246	0.53343514	4.48956638	7.14E-06	1.47E-05	Up	CCDC83
CTXN2	0.64766638	-1.99972904	0.44546212	-4.48911135	7.15E-06	1.47E-05	Down	CTXN2

IL23R	30.1396642	-0.7262235	0.16182616	-4.48767669	7.20E-06	1.48E-05	Down	IL23R
ACMSD	5.30719011	1.59318372	0.35511816	4.48634824	7.25E-06	1.49E-05	Up	ACMSD
ANGPTL6	14.2080849	-0.63839668	0.14230858	-4.48600271	7.26E-06	1.49E-05	Down	ANGPTL6
FNDC5	30.3345464	-0.92650865	0.20655306	-4.48557221	7.27E-06	1.50E-05	Down	FNDC5
LRRTM2	14.9278057	-0.97971357	0.21844056	-4.48503505	7.29E-06	1.50E-05	Down	LRRTM2
PLCH2	196.650575	0.95823908	0.21368573	4.48433827	7.31E-06	1.51E-05	Up	PLCH2
ENPEP	174.476024	0.63604697	0.14186375	4.4835061	7.34E-06	1.51E-05	Up	ENPEP
CBS	9.87584968	1.35667599	0.30260708	4.48329226	7.35E-06	1.51E-05	Up	CBS
UGT2B11	1.81139433	-1.80183141	0.40197923	-4.48239922	7.38E-06	1.52E-05	Down	UGT2B11
TET2	708.448987	-0.53822691	0.12012529	-4.48054638	7.45E-06	1.53E-05	Down	TET2
TPRG1	43.1220919	0.73767816	0.16464029	4.4805446	7.45E-06	1.53E-05	Up	TPRG1
FAM210B	2936.23626	0.51282695	0.11446775	4.48009994	7.46E-06	1.53E-05	Up	FAM210B
ZNF404	37.0124572	-0.68222258	0.15231766	-4.47894606	7.50E-06	1.54E-05	Down	ZNF404
GRID2IP	53.4278103	0.55076897	0.12297199	4.47881655	7.51E-06	1.54E-05	Up	GRID2IP
OR1J1	1.0886069	2.55887296	0.57141565	4.47812893	7.53E-06	1.55E-05	Up	OR1J1
DRC1	4.46318492	1.22932135	0.27461909	4.47645991	7.59E-06	1.56E-05	Up	DRC1
SLC22A13	3.45303126	0.90962721	0.20321492	4.47618323	7.60E-06	1.56E-05	Up	SLC22A13
CCND2	7151.06438	0.72974738	0.16304444	4.47575754	7.61E-06	1.56E-05	Up	CCND2
ALG10	126.117956	0.53048159	0.11857683	4.47373732	7.69E-06	1.58E-05	Up	ALG10
PLAG1	120.619668	0.94895438	0.21212196	4.47362633	7.69E-06	1.58E-05	Up	PLAG1
PCP4	142.421856	-1.5887301	0.35519719	-4.47281157	7.72E-06	1.58E-05	Down	PCP4
CFAP44	325.152105	0.63071316	0.14104305	4.47177761	7.76E-06	1.59E-05	Up	CFAP44
APOBEC3C	1166.96782	-0.6787088	0.15178761	-4.47143744	7.77E-06	1.59E-05	Down	APOBEC3C
PPP1R1C	22.5032553	0.98078665	0.2193806	4.47070826	7.80E-06	1.60E-05	Up	PPP1R1C
DPP10	82.8330212	-1.73935363	0.38922315	-4.46878259	7.87E-06	1.61E-05	Down	DPP10
LAG3	148.588659	-0.83090433	0.18594782	-4.46848123	7.88E-06	1.61E-05	Down	LAG3
KCTD1	170.144417	-0.68427219	0.15313895	-4.46830943	7.88E-06	1.62E-05	Down	KCTD1
RORC	712.129054	-0.73798394	0.16517917	-4.46777848	7.90E-06	1.62E-05	Down	RORC
GPR75	24.0255579	0.60217191	0.13486459	4.46501134	8.01E-06	1.64E-05	Up	GPR75
OAS2	1411.62667	0.87195878	0.19531277	4.46442269	8.03E-06	1.64E-05	Up	OAS2
NPY5R	4.31804469	-1.70188035	0.38136728	-4.46257568	8.10E-06	1.66E-05	Down	NPY5R
ANKRD20A1	0.80459071	-1.39797695	0.31341359	-4.46048601	8.18E-06	1.67E-05	Down	ANKRD20A1
TREML4	2.71313324	2.05562292	0.46088707	4.46014449	8.19E-06	1.68E-05	Up	TREML4
LAMC3	149.084105	-0.81031519	0.18177347	-4.45782971	8.28E-06	1.69E-05	Down	LAMC3
FOXJ1	270.32761	1.51434834	0.33983246	4.45616154	8.34E-06	1.71E-05	Up	FOXJ1
ATOH1	716.143715	-1.26669672	0.28435599	-4.45461587	8.40E-06	1.72E-05	Down	ATOH1
CCDC122	193.07153	0.64903762	0.1457364	4.45350393	8.45E-06	1.73E-05	Up	CCDC122
CHFR	815.212829	-0.58674454	0.13181474	-4.45128182	8.54E-06	1.74E-05	Down	CHFR
COX6B2	84.652875	-0.92433517	0.20767721	-4.45082618	8.55E-06	1.75E-05	Down	COX6B2
KRTAP1-5	0.91316655	2.10168817	0.47237712	4.44917436	8.62E-06	1.76E-05	Up	KRTAP1-5
PRSS48	3.2039208	2.03972832	0.45848864	4.44880888	8.63E-06	1.76E-05	Up	PRSS48
RAPGEF4	142.531851	0.66468859	0.14949592	4.44619902	8.74E-06	1.78E-05	Up	RAPGEF4
MSMB	5.10277268	2.6996339	0.60728305	4.44542935	8.77E-06	1.79E-05	Up	MSMB
TDRP	94.1715428	-0.87497176	0.19684007	-4.44508973	8.79E-06	1.79E-05	Down	TDRP
PLA1A	164.377836	0.79086546	0.17792864	4.44484625	8.80E-06	1.80E-05	Up	PLA1A
CFAP53	17.8778337	0.61813398	0.13907129	4.44472733	8.80E-06	1.80E-05	Up	CFAP53
PRB1	1.71487935	3.63644917	0.81818591	4.44452675	8.81E-06	1.80E-05	Up	PRB1
HOXC8	10.9156264	1.72902308	0.38913137	4.44328886	8.86E-06	1.81E-05	Up	HOXC8
CYP2E1	28.2055345	0.91731818	0.20645422	4.44320372	8.86E-06	1.81E-05	Up	CYP2E1
IGFBP7	10471.1197	0.63239041	0.14233185	4.44307024	8.87E-06	1.81E-05	Up	IGFBP7
RBM44	13.6272136	0.8274805	0.1862657	4.44247391	8.89E-06	1.81E-05	Up	RBM44
CD1A	22.1266685	0.9262241	0.20849831	4.44235787	8.90E-06	1.81E-05	Up	CD1A
MROH9	1.52728449	3.30197042	0.74356705	4.44071641	8.97E-06	1.83E-05	Up	MROH9
KRT39	7.52012342	1.76676121	0.39787608	4.44048113	8.98E-06	1.83E-05	Up	KRT39
PCDHB7	37.1870057	-0.77170963	0.17389916	-4.43768465	9.09E-06	1.85E-05	Down	PCDHB7
MSN	5375.18391	-0.54955109	0.12384163	-4.43753103	9.10E-06	1.85E-05	Down	MSN
CCDC169-SC	0.31198022	-3.03751724	0.6845289	-4.43738348	9.11E-06	1.85E-05	Down	CCDC169-SOHLH2
CPNE4	10.2494195	-1.57285709	0.35446983	-4.43721008	9.11E-06	1.86E-05	Down	CPNE4

MCC	227.679105	-0.70254424	0.15838884	-4.43556641	9.18E-06	1.87E-05	Down	MCC
CFAP70	52.6523181	0.84520083	0.19070381	4.43200806	9.34E-06	1.90E-05	Up	CFAP70
HNRNPCL1	1.12018484	1.51486707	0.34192052	4.43046551	9.40E-06	1.91E-05	Up	HNRNPCL1
RNF165	13.7945901	-1.14091583	0.25755783	-4.42974626	9.43E-06	1.92E-05	Down	RNF165
DCX	3.70818569	-1.35795748	0.30656397	-4.42960566	9.44E-06	1.92E-05	Down	DCX
CD164L2	13.8849944	1.05508984	0.23829168	4.42772426	9.52E-06	1.94E-05	Up	CD164L2
CRYGN	2.32776223	-1.10084949	0.24864683	-4.42736191	9.54E-06	1.94E-05	Down	CRYGN
PLA2G2E	0.83165866	2.21672503	0.50115925	4.42319487	9.73E-06	1.98E-05	Up	PLA2G2E
KRTAP3-3	1.69211988	2.16503251	0.48949312	4.42300903	9.73E-06	1.98E-05	Up	KRTAP3-3
GRAMD1B	216.654461	0.9185254	0.20774289	4.42145298	9.80E-06	1.99E-05	Up	GRAMD1B
CNIH2	15.541437	-0.61234638	0.13852156	-4.42058547	9.84E-06	2.00E-05	Down	CNIH2
PDC	2.98396966	2.03583813	0.4605841	4.42012252	9.86E-06	2.00E-05	Up	PDC
GFI1	151.125921	-0.85811728	0.19414893	-4.41989178	9.88E-06	2.01E-05	Down	GFI1
NUDT8	587.299261	0.74155645	0.16783888	4.41826393	9.95E-06	2.02E-05	Up	NUDT8
GPR52	2.97051244	2.91768722	0.66055831	4.41700175	1.00E-05	2.03E-05	Up	GPR52
SMIM22	2451.22541	-0.55340446	0.12536561	-4.41432434	1.01E-05	2.06E-05	Down	SMIM22
RBM41	401.317694	0.50084542	0.11346387	4.41414017	1.01E-05	2.06E-05	Up	RBM41
KRTAP1-1	0.90036501	2.65882667	0.60234929	4.41409445	1.01E-05	2.06E-05	Up	KRTAP1-1
DLG4	236.302561	-0.55355667	0.12542341	-4.41350364	1.02E-05	2.06E-05	Down	DLG4
MILR1	63.7048748	-0.72641381	0.16463314	-4.41231834	1.02E-05	2.07E-05	Down	MILR1
ABCG4	7.24817916	-0.69217859	0.15688381	-4.41204609	1.02E-05	2.08E-05	Down	ABCG4
FSTL4	41.1830085	1.30194588	0.29523753	4.40982519	1.03E-05	2.10E-05	Up	FSTL4
HP	17.5897038	1.72848761	0.39213719	4.4078645	1.04E-05	2.11E-05	Up	HP
TFF1	4671.26953	1.28815044	0.29225915	4.40756241	1.05E-05	2.12E-05	Up	TFF1
AMT	583.599793	-0.75945038	0.17246574	-4.40348561	1.07E-05	2.16E-05	Down	AMT
ARMCX2	288.452421	-0.68080667	0.15477092	-4.39880231	1.09E-05	2.20E-05	Down	ARMCX2
OMG	11.4948271	-1.03043459	0.2342782	-4.39833749	1.09E-05	2.21E-05	Down	OMG
C10orf62	0.55895784	1.98716497	0.45196997	4.39667478	1.10E-05	2.22E-05	Up	C10orf62
LTA	20.0735695	-0.73397101	0.16695396	-4.3962481	1.10E-05	2.23E-05	Down	LTA
METRNL	1670.83445	-0.60155265	0.13685835	-4.39543981	1.11E-05	2.24E-05	Down	METRNL
CDH26	24.9118576	0.99799708	0.22720508	4.39249458	1.12E-05	2.27E-05	Up	CDH26
EPPIN	1.86861837	2.51757258	0.57321795	4.39199888	1.12E-05	2.27E-05	Up	EPPIN
RPS14	42150.6565	0.55883014	0.12739624	4.38655126	1.15E-05	2.33E-05	Up	RPS14
TAPBPL	1804.66413	-0.50352432	0.11479952	-4.38611869	1.15E-05	2.33E-05	Down	TAPBPL
GCGR	4.70040981	-1.13644388	0.25913022	-4.38560926	1.16E-05	2.34E-05	Down	GCGR
CLIC6	183.394475	-0.94793178	0.21616038	-4.38531704	1.16E-05	2.34E-05	Down	CLIC6
SLC22A2	11.1607042	1.19041651	0.27157002	4.38346074	1.17E-05	2.36E-05	Up	SLC22A2
SYNE2	3900.87304	-0.51470634	0.11747673	-4.38134715	1.18E-05	2.38E-05	Down	SYNE2
PDGFB	547.02137	0.51876099	0.11840288	4.38132063	1.18E-05	2.38E-05	Up	PDGFB
TLR8	82.6597544	-0.97880515	0.22342641	-4.38088395	1.18E-05	2.39E-05	Down	TLR8
ZNF727	4.66679694	-1.54047179	0.35178705	-4.37898949	1.19E-05	2.41E-05	Down	ZNF727
TREH	68.5125758	-0.93892135	0.21449652	-4.37732676	1.20E-05	2.42E-05	Down	TREH
GPNMB	2839.0099	-0.87082996	0.19895063	-4.37711582	1.20E-05	2.43E-05	Down	GPNMB
CRABP1	24.6558014	-1.42169215	0.3248626	-4.37628752	1.21E-05	2.43E-05	Down	CRABP1
ANO3	6.66126463	-1.68657277	0.3854899	-4.37514124	1.21E-05	2.45E-05	Down	ANO3
GABRA3	31.9124514	2.10335381	0.48086127	4.37413854	1.22E-05	2.46E-05	Up	GABRA3
VSTM2L	166.624474	1.31820432	0.30138535	4.3738169	1.22E-05	2.46E-05	Up	VSTM2L
IFIT1	427.858614	-0.91475549	0.20917318	-4.37319689	1.22E-05	2.47E-05	Down	IFIT1
PYDC1	2.99462019	2.34517575	0.53631875	4.37272748	1.23E-05	2.47E-05	Up	PYDC1
PARP8	741.498099	0.53152082	0.12160603	4.3708425	1.24E-05	2.49E-05	Up	PARP8
SPDYE2	5.79209612	1.01249496	0.23164794	4.3708352	1.24E-05	2.49E-05	Up	SPDYE2
ICA1L	78.2080301	-0.68632666	0.15702517	-4.37080656	1.24E-05	2.49E-05	Down	ICA1L
PKDREJ	27.2350277	-0.58110949	0.13304713	-4.36769672	1.26E-05	2.53E-05	Down	PKDREJ
NMBR	0.57762068	-1.35777867	0.31092193	-4.3669441	1.26E-05	2.53E-05	Down	NMBR
DHRS13	165.059751	0.5069022	0.11608038	4.36682074	1.26E-05	2.54E-05	Up	DHRS13
RPTN	1.90386928	3.58062667	0.82049142	4.36400256	1.28E-05	2.57E-05	Up	RPTN
TXK	27.8623298	-0.65157706	0.14936372	-4.36235145	1.29E-05	2.59E-05	Down	TXK
GRIP2	86.9025073	0.98213021	0.22526943	4.35980239	1.30E-05	2.61E-05	Up	GRIP2

TTLL10-AS1	3.96415776	1.23551871	0.28357095	4.35700033	1.32E-05	2.65E-05	Up	TTLL10-AS1
LY6G5B	76.4217401	0.58447867	0.13415815	4.35663939	1.32E-05	2.65E-05	Up	LY6G5B
TAS2R30	2.60408749	2.70487244	0.62097014	4.35588165	1.33E-05	2.66E-05	Up	TAS2R30
SEMA3C	2571.12309	-0.55365072	0.12720371	-4.35247304	1.35E-05	2.70E-05	Down	SEMA3C
MYLK3	6.83700759	-0.74605214	0.17141956	-4.35219967	1.35E-05	2.71E-05	Down	MYLK3
GATA1	4.16398048	-1.03308901	0.23741311	-4.35144037	1.35E-05	2.71E-05	Down	GATA1
AKR1B1	897.242796	-0.63604437	0.1461844	-4.35097301	1.36E-05	2.72E-05	Down	AKR1B1
RAB40AL	2.11617017	1.79487043	0.41258339	4.35032157	1.36E-05	2.73E-05	Up	RAB40AL
STAC2	8.32607964	-1.26620467	0.2910795	-4.35003044	1.36E-05	2.73E-05	Down	STAC2
S100G	1.43492746	2.82990296	0.65062013	4.34954723	1.36E-05	2.74E-05	Up	S100G
GALNT9	39.2536038	1.41387223	0.3251135	4.34885734	1.37E-05	2.74E-05	Up	GALNT9
PLCXD2	31.1888624	-0.52320274	0.12031088	-4.34875647	1.37E-05	2.75E-05	Down	PLCXD2
ZNF680	284.287298	0.53856659	0.12384964	4.34855189	1.37E-05	2.75E-05	Up	ZNF680
OR2B6	2.9669746	1.24218501	0.28565741	4.34851316	1.37E-05	2.75E-05	Up	OR2B6
HLA-DPB1	4042.31629	-0.76505296	0.17606354	-4.34532306	1.39E-05	2.79E-05	Down	HLA-DPB1
RAC2	1423.34462	-0.53832817	0.12389483	-4.34504159	1.39E-05	2.79E-05	Down	RAC2
P3H3	619.457922	0.63918852	0.14715706	4.34358032	1.40E-05	2.81E-05	Up	P3H3
IL18RAP	33.1244031	-0.7828598	0.18028063	-4.34245096	1.41E-05	2.82E-05	Down	IL18RAP
TMPRSS11D	1.91815925	2.80157766	0.64519769	4.34220039	1.41E-05	2.82E-05	Up	TMPRSS11D
GLYATL3	0.9867994	-2.52852403	0.5824675	-4.34105597	1.42E-05	2.84E-05	Down	GLYATL3
AGTR2	0.76352182	-2.83255744	0.65282502	-4.33892293	1.43E-05	2.87E-05	Down	AGTR2
RBM11	35.9981017	0.9664361	0.22279115	4.33785681	1.44E-05	2.88E-05	Up	RBM11
MTNR1A	14.8787399	-1.07926995	0.24881384	-4.33766034	1.44E-05	2.88E-05	Down	MTNR1A
ZNF423	237.310754	-0.75500498	0.17426807	-4.33243448	1.47E-05	2.95E-05	Down	ZNF423
TCF4	1051.2027	-0.67531411	0.15587424	-4.33242923	1.47E-05	2.95E-05	Down	TCF4
AKR1C1	157.967198	-0.91391351	0.21102497	-4.3308312	1.49E-05	2.97E-05	Down	AKR1C1
FOLH1	58.0790017	0.75287717	0.17384265	4.33079655	1.49E-05	2.97E-05	Up	FOLH1
ITGA3	5122.09262	-0.51519949	0.11899272	-4.32967246	1.49E-05	2.98E-05	Down	ITGA3
POPDC3	15.2699618	-1.26695275	0.29266058	-4.32908574	1.50E-05	2.99E-05	Down	POPDC3
LILRB1	158.116323	-0.71770334	0.16585956	-4.32717506	1.51E-05	3.01E-05	Down	LILRB1
ITGA1	2412.0291	-0.5165468	0.11938584	-4.32670081	1.51E-05	3.02E-05	Down	ITGA1
PROX2	13.8287798	0.98491212	0.22771579	4.32518152	1.52E-05	3.04E-05	Up	PROX2
PKD2	583.652665	-0.56773394	0.13135234	-4.32222163	1.54E-05	3.08E-05	Down	PKD2
PLAUR	2523.58905	0.53390107	0.12359758	4.31967234	1.56E-05	3.12E-05	Up	PLAUR
RPS28	13566.6862	0.56490896	0.13091349	4.31513163	1.60E-05	3.18E-05	Up	RPS28
CCKAR	1.40868422	-2.43683976	0.56477997	-4.31467103	1.60E-05	3.18E-05	Down	CCKAR
PM20D2	1468.49151	0.58128466	0.13474936	4.31382129	1.60E-05	3.19E-05	Up	PM20D2
LRRN1	84.0386618	1.21914694	0.28267148	4.31294642	1.61E-05	3.21E-05	Up	LRRN1
CELF2	1591.09141	-0.51367933	0.11911436	-4.3124887	1.61E-05	3.21E-05	Down	CELF2
PTCHD4	29.8167823	1.29120942	0.29943494	4.31215344	1.62E-05	3.22E-05	Up	PTCHD4
PROSER2	378.881187	0.59856648	0.1388172	4.31190436	1.62E-05	3.22E-05	Up	PROSER2
ADM5	45.7843232	0.7425872	0.17221808	4.31190036	1.62E-05	3.22E-05	Up	ADM5
VPS37D	67.7019049	0.83445471	0.1935415	4.31150283	1.62E-05	3.23E-05	Up	VPS37D
TM4SF4	98.9643444	1.40655504	0.32624935	4.31128839	1.62E-05	3.23E-05	Up	TM4SF4
THBD	533.171484	-0.55070701	0.12778318	-4.3096989	1.63E-05	3.25E-05	Down	THBD
RNASE7	11.9353968	1.20343378	0.27926608	4.30927301	1.64E-05	3.26E-05	Up	RNASE7
SCUBE3	177.389265	1.06247416	0.24661247	4.30827429	1.65E-05	3.27E-05	Up	SCUBE3
SELP	133.160843	-0.91418918	0.21224689	-4.3071971	1.65E-05	3.29E-05	Down	SELP
SLAMF6	140.439494	-0.77172367	0.17917477	-4.30710009	1.65E-05	3.29E-05	Down	SLAMF6
FGFBP3	76.1624367	0.5451816	0.12669741	4.30302082	1.68E-05	3.34E-05	Up	FGFBP3
TMEM187	505.213493	0.54595827	0.12693009	4.30125168	1.70E-05	3.37E-05	Up	TMEM187
FAM83C	12.0991657	1.64586768	0.38283933	4.29910816	1.71E-05	3.40E-05	Up	FAM83C
CER1	1.41564259	2.08755286	0.48591039	4.29616843	1.74E-05	3.45E-05	Up	CER1
BCL11A	667.821553	0.56554809	0.13172105	4.29352855	1.76E-05	3.49E-05	Up	BCL11A
CHRFAM7A	5.97913109	-1.10300962	0.25693624	-4.29293126	1.76E-05	3.50E-05	Down	CHRFAM7A
PMEL	165.862678	0.69817605	0.16278424	4.28896598	1.80E-05	3.56E-05	Up	PMEL
SLC6A17	12.4552312	-1.00039236	0.23329967	-4.28801451	1.80E-05	3.57E-05	Down	SLC6A17
TMEM238	874.230388	0.92085049	0.21482487	4.2865171	1.81E-05	3.60E-05	Up	TMEM238

OR10H1	0.80924542	2.19279868	0.51157688	4.28635217	1.82E-05	3.60E-05	Up	OR10H1
TACC1	4186.84699	-0.5548174	0.12954593	-4.28278515	1.85E-05	3.65E-05	Down	TACC1
CHD9	1237.5818	-0.50157785	0.1171868	-4.28015644	1.87E-05	3.70E-05	Down	CHD9
IL18R1	106.925795	-0.61431992	0.14355902	-4.27921517	1.88E-05	3.71E-05	Down	IL18R1
KRT25	0.36603857	-3.10542789	0.72602647	-4.27729295	1.89E-05	3.74E-05	Down	KRT25
HLA-DPA1	5192.85307	-0.80527252	0.18860927	-4.26952784	1.96E-05	3.87E-05	Down	HLA-DPA1
MYOZ2	1.95039474	-1.1939921	0.27971551	-4.26859453	1.97E-05	3.89E-05	Down	MYOZ2
TNFRSF25	655.060917	0.6063012	0.14205552	4.26805793	1.97E-05	3.90E-05	Up	TNFRSF25
RAB3B	224.09975	-1.0079862	0.23622372	-4.26708284	1.98E-05	3.91E-05	Down	RAB3B
C9orf24	45.7195833	-0.94971677	0.22258226	-4.26681251	1.98E-05	3.92E-05	Down	C9orf24
SOX7	77.9787051	-0.66860307	0.15670662	-4.26659112	1.98E-05	3.92E-05	Down	SOX7
ZNF853	195.108295	-0.79786406	0.18706949	-4.26506788	2.00E-05	3.95E-05	Down	ZNF853
TBX1	19.9700583	-0.9190593	0.21553303	-4.26412281	2.01E-05	3.96E-05	Down	TBX1
KRT85	1.12415184	2.68347998	0.62936881	4.26376381	2.01E-05	3.97E-05	Up	KRT85
SNX32	54.2759522	-0.64020211	0.15020668	-4.26214127	2.02E-05	4.00E-05	Down	SNX32
LILRA1	24.2076934	-0.75228422	0.17656658	-4.26062643	2.04E-05	4.02E-05	Down	LILRA1
AGAP4	54.7255335	0.53042221	0.12449516	4.260585	2.04E-05	4.02E-05	Up	AGAP4
MAMDC4	413.442651	0.74281235	0.17441882	4.25878552	2.06E-05	4.05E-05	Up	MAMDC4
RPL3L	8.58358417	0.69884003	0.16413928	4.25760382	2.07E-05	4.08E-05	Up	RPL3L
DAPK1	753.638091	-0.8130672	0.19098638	-4.25719979	2.07E-05	4.08E-05	Down	DAPK1
CCDC166	0.78466271	1.94367179	0.45693305	4.25373433	2.10E-05	4.15E-05	Up	CCDC166
UCP1	0.64864341	2.0716121	0.48718856	4.25217723	2.12E-05	4.17E-05	Up	UCP1
DNM1	563.96873	0.67765268	0.15952274	4.24800042	2.16E-05	4.25E-05	Up	DNM1
PRL	2.21625179	1.80703696	0.42540031	4.24785058	2.16E-05	4.25E-05	Up	PRL
MTMR7	33.1394071	-0.70696291	0.16644056	-4.24753989	2.16E-05	4.26E-05	Down	MTMR7
NOXA1	730.556271	0.62447504	0.14702872	4.2472996	2.16E-05	4.26E-05	Up	NOXA1
OPRL1	33.163414	-0.71362826	0.16804658	-4.24660981	2.17E-05	4.27E-05	Down	OPRL1
UGT3A2	3.46084356	-1.3039655	0.30706488	-4.24654715	2.17E-05	4.27E-05	Down	UGT3A2
TFAP2E	32.3128156	0.79536548	0.18734009	4.2455701	2.18E-05	4.29E-05	Up	TFAP2E
CFAP69	128.929774	0.57158593	0.13465319	4.24487471	2.19E-05	4.31E-05	Up	CFAP69
MRPL23	1559.0924	0.51084918	0.12035589	4.24448836	2.19E-05	4.31E-05	Up	MRPL23
POMC	14.8819992	-0.84405894	0.19889947	-4.24364603	2.20E-05	4.33E-05	Down	POMC
NLRP6	92.8837987	1.18992609	0.28047437	4.24254843	2.21E-05	4.35E-05	Up	NLRP6
KCNN1	6.03295469	-0.760551	0.1792884	-4.24205368	2.21E-05	4.36E-05	Down	KCNN1
RASAL3	251.955957	-0.6179181	0.14584645	-4.2367717	2.27E-05	4.46E-05	Down	RASAL3
CRACR2B	1670.55803	0.55274046	0.13050362	4.23544148	2.28E-05	4.48E-05	Up	CRACR2B
BMP8A	50.8528742	0.63201275	0.14926557	4.23414962	2.29E-05	4.51E-05	Up	BMP8A
CNNM1	25.3475484	-1.02721705	0.24273353	-4.23187133	2.32E-05	4.55E-05	Down	CNNM1
PCDHGA4	12.5119466	-0.91408207	0.21601972	-4.23147522	2.32E-05	4.56E-05	Down	PCDHGA4
PITX3	0.83259486	1.71562055	0.40551622	4.2307076	2.33E-05	4.58E-05	Up	PITX3
TLR9	1.20552586	-0.91641734	0.21661437	-4.23063954	2.33E-05	4.58E-05	Down	TLR9
SLC5A9	142.11549	0.97198924	0.22986039	4.22860689	2.35E-05	4.62E-05	Up	SLC5A9
ZNF215	169.706957	0.54070592	0.12786841	4.22861211	2.35E-05	4.62E-05	Up	ZNF215
FST	84.8801325	-0.83078959	0.19647869	-4.22839536	2.35E-05	4.62E-05	Down	FST
HSD17B13	11.941253	-0.66839906	0.15819035	-4.22528328	2.39E-05	4.68E-05	Down	HSD17B13
SLC7A9	29.0750748	1.35876239	0.32161216	4.22484766	2.39E-05	4.69E-05	Up	SLC7A9
PTGER2	219.227633	-0.83363896	0.19734882	-4.2241902	2.40E-05	4.70E-05	Down	PTGER2
IL15	164.194177	-0.59797241	0.14157818	-4.22361987	2.40E-05	4.71E-05	Down	IL15
NHLRC4	21.4078826	-0.59588522	0.14124792	-4.21871839	2.46E-05	4.82E-05	Down	NHLRC4
TSPAN6	5523.60813	0.52358271	0.12416243	4.21691752	2.48E-05	4.85E-05	Up	TSPAN6
FASLG	28.7809456	-0.84943044	0.20143888	-4.21681482	2.48E-05	4.86E-05	Down	FASLG
HLA-DQA1	2181.90551	-0.81059973	0.19232212	-4.21480245	2.50E-05	4.90E-05	Down	HLA-DQA1
S100Z	2.99209905	-0.9621901	0.22830852	-4.21442924	2.50E-05	4.91E-05	Down	S100Z
IMPG1	13.1949821	0.8226514	0.19528836	4.21249578	2.53E-05	4.94E-05	Up	IMPG1
LCTL	9.38994306	0.67425731	0.16006504	4.21239588	2.53E-05	4.95E-05	Up	LCTL
MNS1	89.9153689	0.6425074	0.15254034	4.21204898	2.53E-05	4.95E-05	Up	MNS1
NKX6-3	15.4666025	2.45100033	0.58204275	4.21103149	2.54E-05	4.97E-05	Up	NKX6-3
KLK2	4.57778282	1.39943264	0.33235316	4.21067947	2.55E-05	4.98E-05	Up	KLK2

TMEM89	8.86021773	0.93309084	0.22179493	4.20699801	2.59E-05	5.06E-05	Up	TMEM89
SIRPG	65.5739179	-0.72963845	0.17345452	-4.20651166	2.59E-05	5.07E-05	Down	SIRPG
FAM72C	6.97855689	1.01822127	0.24211195	4.20558044	2.60E-05	5.09E-05	Up	FAM72C
NTSR1	133.836867	-1.42848351	0.33972031	-4.20488107	2.61E-05	5.10E-05	Down	NTSR1
SLURP1	0.95272441	2.38265804	0.56669255	4.20449861	2.62E-05	5.11E-05	Up	SLURP1
LAMP3	289.074712	0.58253212	0.1386977	4.20001287	2.67E-05	5.21E-05	Up	LAMP3
RFX4	2.83437832	1.22040968	0.29072672	4.19778985	2.70E-05	5.26E-05	Up	RFX4
PTK6	2712.95648	-0.50511028	0.12034531	-4.19717471	2.70E-05	5.27E-05	Down	PTK6
C1orf87	1.21899145	2.03326562	0.48445864	4.19698498	2.70E-05	5.28E-05	Up	C1orf87
CACNG6	1.51913886	-1.53008145	0.36458397	-4.19678748	2.71E-05	5.28E-05	Down	CACNG6
SLCO3A1	741.033523	0.55444916	0.13216448	4.19514519	2.73E-05	5.32E-05	Up	SLCO3A1
EEF1A2	89.8359391	-1.20031288	0.28618635	-4.19416543	2.74E-05	5.34E-05	Down	EEF1A2
ITGAD	8.14970763	-0.94730066	0.22587166	-4.19397749	2.74E-05	5.34E-05	Down	ITGAD
ADAD2	1.88538614	-0.9748731	0.2325454	-4.19218395	2.76E-05	5.39E-05	Down	ADAD2
C1QL1	33.2484842	-0.89504581	0.21359778	-4.19033297	2.79E-05	5.43E-05	Down	C1QL1
ASCL1	2.66326212	-1.60180269	0.38260664	-4.18655223	2.83E-05	5.52E-05	Down	ASCL1
AXDND1	98.9197377	-1.14406425	0.27329505	-4.18618728	2.84E-05	5.52E-05	Down	AXDND1
C11orf87	2.82022402	1.58723254	0.37945259	4.18295355	2.88E-05	5.60E-05	Up	C11orf87
NPIPB4	74.372091	0.64988905	0.15538353	4.18248337	2.88E-05	5.61E-05	Up	NPIPB4
ST3GAL1	1051.36246	0.67472956	0.16138713	4.18081385	2.90E-05	5.65E-05	Up	ST3GAL1
SLC38A8	1.98395805	1.98977882	0.47618366	4.17859536	2.93E-05	5.70E-05	Up	SLC38A8
CPA5	3.44903179	1.30458478	0.31236967	4.17641304	2.96E-05	5.76E-05	Up	CPA5
LRRC75B	357.446064	0.56610745	0.13555098	4.17634345	2.96E-05	5.76E-05	Up	LRRC75B
ANP32D	1.64818398	1.47153929	0.35249349	4.17465664	2.98E-05	5.80E-05	Up	ANP32D
CCL3L3	88.7359181	1.03750805	0.24864569	4.17263642	3.01E-05	5.85E-05	Up	CCL3L3
C11orf16	3.54321495	1.03528038	0.24815256	4.17195127	3.02E-05	5.87E-05	Up	C11orf16
TAS2R13	2.08535318	2.57879774	0.61820767	4.17141014	3.03E-05	5.88E-05	Up	TAS2R13
ART1	0.96197413	-1.67679405	0.40200143	-4.17111466	3.03E-05	5.89E-05	Down	ART1
SFN	6655.72261	-0.61289508	0.14694616	-4.17088177	3.03E-05	5.89E-05	Down	SFN
LMNTD1	0.95041521	-2.31325337	0.55472913	-4.17005933	3.05E-05	5.91E-05	Down	LMNTD1
FSIP1	36.4396081	-1.02674498	0.24638024	-4.16731862	3.08E-05	5.98E-05	Down	FSIP1
REG3G	4.69386874	2.37509889	0.57048392	4.16330559	3.14E-05	6.09E-05	Up	REG3G
IGFBP2	6518.47571	0.91598623	0.22017339	4.16029495	3.18E-05	6.16E-05	Up	IGFBP2
GADL1	2.85932388	1.50051108	0.36079055	4.1589534	3.20E-05	6.20E-05	Up	GADL1
SH3GL2	14.6626737	-1.46792502	0.35367137	-4.15053393	3.32E-05	6.43E-05	Down	SH3GL2
TRPM8	6.17728425	1.31796166	0.31758336	4.1499708	3.33E-05	6.44E-05	Up	TRPM8
ABCA13	55.9234577	0.99795387	0.24050304	4.14944392	3.33E-05	6.46E-05	Up	ABCA13
CELA1	0.95286415	-1.36088488	0.32799364	-4.14911973	3.34E-05	6.46E-05	Down	CELA1
MX2	659.347988	0.62368209	0.15033929	4.14849692	3.35E-05	6.48E-05	Up	MX2
PRR35	0.88976061	2.13132526	0.51407861	4.14591316	3.38E-05	6.55E-05	Up	PRR35
A1BG	3.31383554	-0.67513346	0.16286298	-4.14540771	3.39E-05	6.56E-05	Down	A1BG
PPP4R4	11.4818693	1.1297119	0.27256291	4.14477487	3.40E-05	6.58E-05	Up	PPP4R4
NCF4	399.895733	-0.62100327	0.14990357	-4.14268493	3.43E-05	6.64E-05	Down	NCF4
MGST1	4111.03174	-0.5104941	0.12324294	-4.14217715	3.44E-05	6.65E-05	Down	MGST1
DNASE2B	8.24425167	-0.76070794	0.18365101	-4.14213864	3.44E-05	6.65E-05	Down	DNASE2B
COMTD1	1328.5046	0.70018687	0.16904507	4.142013	3.44E-05	6.66E-05	Up	COMTD1
FZD2	149.743583	0.67862442	0.16384931	4.1417595	3.45E-05	6.66E-05	Up	FZD2
SLC25A6	39511.4601	0.58043271	0.14015861	4.14125617	3.45E-05	6.68E-05	Up	SLC25A6
N4BP2L2	2609.8677	0.52166267	0.12601694	4.13962335	3.48E-05	6.72E-05	Up	N4BP2L2
SHC2	442.576193	0.75962783	0.18352682	4.13905614	3.49E-05	6.74E-05	Up	SHC2
MYL2	1.36547566	-1.24547051	0.30117066	-4.1354311	3.54E-05	6.84E-05	Down	MYL2
DNHD1	314.864657	0.54336362	0.13142513	4.13439656	3.56E-05	6.87E-05	Up	DNHD1
IQCF1	0.86967197	1.75553226	0.424634	4.13422448	3.56E-05	6.88E-05	Up	IQCF1
TAL2	2.08030741	1.03035906	0.24937437	4.13177615	3.60E-05	6.95E-05	Up	TAL2
EPPK1	1622.84983	0.56747669	0.13735435	4.13147952	3.60E-05	6.96E-05	Up	EPPK1
C12orf50	1.01874851	1.7153381	0.41551484	4.12822341	3.66E-05	7.05E-05	Up	C12orf50
SUSD4	18.7879308	-0.95104122	0.23046617	-4.12659785	3.68E-05	7.10E-05	Down	SUSD4
RHOB	7581.19869	-0.51122324	0.12393445	-4.12494872	3.71E-05	7.15E-05	Down	RHOB

SNPH	179.327235	-0.75746094	0.18379404	-4.12124859	3.77E-05	7.26E-05	Down	SNPH
BST2	2567.1072	0.85707636	0.20797722	4.12101073	3.77E-05	7.27E-05	Up	BST2
ART5	11.8238762	1.02881203	0.24973091	4.11968244	3.79E-05	7.31E-05	Up	ART5
SYCP2L	7.18068094	1.08149367	0.26253959	4.11935454	3.80E-05	7.32E-05	Up	SYCP2L
C8orf48	8.34110062	-0.7559411	0.18358411	-4.11768269	3.83E-05	7.37E-05	Down	C8orf48
CD6	274.431977	-0.56023059	0.13609336	-4.11651672	3.85E-05	7.41E-05	Down	CD6
FBXO47	0.71653601	1.86579043	0.4533116	4.11591149	3.86E-05	7.42E-05	Up	FBXO47
GDF7	26.0696608	-0.88164366	0.21421276	-4.11573825	3.86E-05	7.43E-05	Down	GDF7
RGS1	1112.93245	-0.74637251	0.18140945	-4.11429792	3.88E-05	7.47E-05	Down	RGS1
PAGE2B	0.83570016	1.72306968	0.41886313	4.11368185	3.89E-05	7.49E-05	Up	PAGE2B
DDX60L	725.384678	-0.56719244	0.13796231	-4.11121309	3.94E-05	7.57E-05	Down	DDX60L
FAM71D	4.82257515	0.82603421	0.20093072	4.11103983	3.94E-05	7.57E-05	Up	FAM71D
SFTPA2	81.5391404	-1.1713656	0.28500597	-4.10996863	3.96E-05	7.61E-05	Down	SFTPA2
CHADL	73.957353	-0.6432998	0.15660604	-4.10775857	4.00E-05	7.68E-05	Down	CHADL
ERBB4	3.41236488	-1.65064515	0.40208079	-4.10525746	4.04E-05	7.76E-05	Down	ERBB4
ICAM1	1358.66019	0.56652625	0.13804344	4.10397072	4.06E-05	7.80E-05	Up	ICAM1
CCDC169	9.57961229	-1.56570045	0.38158934	-4.1031032	4.08E-05	7.83E-05	Down	CCDC169
TBR1	1.0719262	2.15729359	0.52579182	4.10294248	4.08E-05	7.83E-05	Up	TBR1
TRPV6	41.3027036	1.58774937	0.38705789	4.10209798	4.09E-05	7.86E-05	Up	TRPV6
LGALS7	1.00100474	2.42361454	0.59087662	4.1017269	4.10E-05	7.87E-05	Up	LGALS7
DBX1	2.47473428	1.76561272	0.43052072	4.10110972	4.11E-05	7.89E-05	Up	DBX1
AQP3	1172.01449	1.01540979	0.24767849	4.09970917	4.14E-05	7.93E-05	Up	AQP3
PCDHB8	22.57785	1.35440896	0.33048549	4.09824035	4.16E-05	7.98E-05	Up	PCDHB8
ANKFN1	17.9349696	1.06826694	0.26089983	4.09454833	4.23E-05	8.10E-05	Up	ANKFN1
FAM171A2	37.3250101	0.69657343	0.17013608	4.09421346	4.24E-05	8.12E-05	Up	FAM171A2
OSTN	0.67706426	-2.48847919	0.60814585	-4.09191185	4.28E-05	8.20E-05	Down	OSTN
COLEC11	41.2658983	-0.83761749	0.2047186	-4.09155538	4.28E-05	8.21E-05	Down	COLEC11
CCR5	184.04866	-0.67905433	0.16602495	-4.09007398	4.31E-05	8.26E-05	Down	CCR5
EBF3	34.4753093	-0.82662233	0.20211365	-4.08988869	4.32E-05	8.26E-05	Down	EBF3
SCNN1D	49.7207392	0.67468337	0.1650415	4.08796205	4.35E-05	8.33E-05	Up	SCNN1D
AGRP	2.24346192	1.14415624	0.27994999	4.08700226	4.37E-05	8.36E-05	Up	AGRP
DNAJB13	4.50860312	0.92798414	0.22718261	4.08474996	4.41E-05	8.44E-05	Up	DNAJB13
CD2	308.42097	-0.66379978	0.16251084	-4.08464919	4.41E-05	8.44E-05	Down	CD2
MLIP	4.68167556	-1.05346513	0.25790935	-4.0846334	4.41E-05	8.44E-05	Down	MLIP
NOX1	6444.85766	0.77552194	0.18997472	4.08223765	4.46E-05	8.53E-05	Up	NOX1
IL22RA2	9.48580041	-1.04445654	0.25610125	-4.0782954	4.54E-05	8.67E-05	Down	IL22RA2
RASL10A	16.5669546	0.6617203	0.16225821	4.07819291	4.54E-05	8.67E-05	Up	RASL10A
AS3MT	3.31884541	-0.93231563	0.22862157	-4.07798626	4.54E-05	8.68E-05	Down	AS3MT
SULT1A3	2.88143443	0.76035662	0.18651319	4.07669091	4.57E-05	8.72E-05	Up	SULT1A3
BOK	1175.65696	0.53274158	0.13082186	4.0722672	4.66E-05	8.89E-05	Up	BOK
RUFY4	13.5977993	0.83290445	0.20457598	4.07136981	4.67E-05	8.92E-05	Up	RUFY4
MAPRE3	632.319648	-0.50860888	0.12500123	-4.06883118	4.72E-05	9.02E-05	Down	MAPRE3
C10orf67	7.13715579	1.20870415	0.29709034	4.06847343	4.73E-05	9.03E-05	Up	C10orf67
SNAI3	40.3639222	-0.63664154	0.15651195	-4.06768648	4.75E-05	9.06E-05	Down	SNAI3
KCNA2	9.11094345	-1.08605132	0.26702617	-4.06720927	4.76E-05	9.07E-05	Down	KCNA2
ZNF628	332.923849	0.51372905	0.12632872	4.06660532	4.77E-05	9.10E-05	Up	ZNF628
ZNF233	52.8026149	-0.58950245	0.14514516	-4.0614683	4.88E-05	9.30E-05	Down	ZNF233
RGL4	34.1640625	-0.53570358	0.13211573	-4.05480555	5.02E-05	9.55E-05	Down	RGL4
FBN3	4.42392974	1.48218065	0.36562306	4.05384893	5.04E-05	9.59E-05	Up	FBN3
SRGAP2C	64.3251408	-0.67738106	0.16717906	-4.05182956	5.08E-05	9.67E-05	Down	SRGAP2C
MIA	37.8673176	1.33325497	0.32936069	4.04800877	5.17E-05	9.83E-05	Up	MIA
CORO1A	1803.40618	-0.5821169	0.14381403	-4.04770586	5.17E-05	9.84E-05	Down	CORO1A
SLC4A9	6.80797511	-0.87146513	0.21538082	-4.04615937	5.21E-05	9.90E-05	Down	SLC4A9
NPR3	117.907774	-1.14523143	0.28308028	-4.04560654	5.22E-05	9.92E-05	Down	NPR3
LEKR1	23.6356111	0.56654162	0.14005305	4.04519307	5.23E-05	9.94E-05	Up	LEKR1
WDR86	45.4695275	-0.59747647	0.14774364	-4.04400798	5.25E-05	9.98E-05	Down	WDR86
ADORA1	75.5712206	0.72531377	0.17952266	4.04023521	5.34E-05	0.00010142	Up	ADORA1
GZMK	45.6753235	-0.95657208	0.23709691	-4.03451945	5.47E-05	0.00010388	Down	GZMK

KIAA1210	1.35509403	1.58016807	0.39183104	4.03277924	5.51E-05	0.00010463	Up	KIAA1210
CHRNA7	29.5093684	-1.19317967	0.29595676	-4.03160139	5.54E-05	0.00010514	Down	CHRNA7
APOF	1.96970892	1.72788102	0.42876128	4.02993719	5.58E-05	0.00010585	Up	APOF
ELOVL3	8.43786956	0.95017788	0.23579564	4.02966689	5.59E-05	0.00010595	Up	ELOVL3
NLRP3	92.5608821	-0.64323077	0.15975759	-4.02629227	5.67E-05	0.00010747	Down	NLRP3
ZNF486	308.867652	0.75394408	0.18730821	4.02515239	5.69E-05	0.00010798	Up	ZNF486
LRGUK	9.97812084	0.86440091	0.2147531	4.02509161	5.70E-05	0.000108	Up	LRGUK
ADAM2	1.35237619	2.77394345	0.68930074	4.0242862	5.71E-05	0.00010836	Up	ADAM2
NPIPB15	121.205087	1.13647565	0.28245837	4.02351559	5.73E-05	0.00010869	Up	NPIPB15
ZDHHC11B	174.417619	0.98163812	0.24410623	4.02135629	5.79E-05	0.00010966	Up	ZDHHC11B
CEBPE	10.2510111	-1.17976878	0.29343169	-4.02059089	5.81E-05	0.00011	Down	CEBPE
ARL13A	10.8344039	0.81632154	0.2030939	4.01942923	5.83E-05	0.0001105	Up	ARL13A
SLC1A2	14.75336	-0.94794115	0.2358609	-4.01906866	5.84E-05	0.00011066	Down	SLC1A2
LOXHD1	6.65408594	0.93488323	0.23273336	4.01697136	5.90E-05	0.00011162	Up	LOXHD1
BOLL	1.50783302	2.05408261	0.5113637	4.01687219	5.90E-05	0.00011166	Up	BOLL
DYSF	661.009355	-0.53366661	0.13289417	-4.01572628	5.93E-05	0.00011219	Down	DYSF
SLC2A3	1092.58798	0.74859357	0.18657946	4.01219702	6.02E-05	0.00011384	Up	SLC2A3
FRMD6	472.358672	-0.70560173	0.17607969	-4.0072863	6.14E-05	0.00011616	Down	FRMD6
MROH5	2.31357159	1.51567424	0.37825538	4.00701307	6.15E-05	0.00011628	Up	MROH5
MYOM2	73.6030657	-0.65098636	0.16249779	-4.00612449	6.17E-05	0.0001167	Down	MYOM2
ATP8B3	37.6075808	0.73985553	0.18468141	4.00611813	6.17E-05	0.0001167	Up	ATP8B3
KIAA1755	230.90847	0.57749867	0.14416999	4.00567875	6.18E-05	0.00011691	Up	KIAA1755
KRTAP2-3	0.7025256	2.40743695	0.60138122	4.0031795	6.25E-05	0.00011812	Up	KRTAP2-3
NAA11	1.34690621	2.62656738	0.65637288	4.00163912	6.29E-05	0.00011883	Up	NAA11
CAPNS2	1.58553395	1.21687783	0.3042878	3.99910166	6.36E-05	0.00012004	Up	CAPNS2
OR1F1	0.73440932	2.35073918	0.58800788	3.99780214	6.39E-05	0.00012066	Up	OR1F1
HSD17B14	90.7312295	-0.57212794	0.14314393	-3.99687175	6.42E-05	0.00012112	Down	HSD17B14
SFTPB	16.5925629	1.52297726	0.38107268	3.99655331	6.43E-05	0.00012127	Up	SFTPB
SUMO4	4.31522065	1.11197394	0.27828525	3.99580629	6.45E-05	0.00012163	Up	SUMO4
GPR65	96.602677	-0.67085691	0.16792843	-3.99489779	6.47E-05	0.00012207	Down	GPR65
AIFM3	1364.75304	-0.75725116	0.18962247	-3.99346752	6.51E-05	0.00012277	Down	AIFM3
FAM156B	0.72893462	1.53734248	0.38504148	3.99266717	6.53E-05	0.00012317	Up	FAM156B
BCO1	131.94767	-0.59808423	0.14980085	-3.99252886	6.54E-05	0.00012322	Down	BCO1
TSKS	3.25232528	-0.9785594	0.24510328	-3.99243696	6.54E-05	0.00012325	Down	TSKS
SYNPR	26.4377003	1.56708547	0.39254463	3.99212054	6.55E-05	0.00012338	Up	SYNPR
TAF7L	7.13144704	0.91625066	0.22954577	3.99158151	6.56E-05	0.00012365	Up	TAF7L
ZNF718	120.684423	-0.61337624	0.1537082	-3.99052392	6.59E-05	0.00012419	Down	ZNF718
ACSBG2	5.02420835	0.83816477	0.21012906	3.98880932	6.64E-05	0.00012506	Up	ACSBG2
KCTD15	544.230091	-0.57318615	0.14373853	-3.9877001	6.67E-05	0.00012561	Down	KCTD15
ARSI	79.8794206	0.79490364	0.19945933	3.98529196	6.74E-05	0.00012685	Up	ARSI
DNMT3L	0.76644699	2.39343804	0.60078053	3.98388085	6.78E-05	0.00012758	Up	DNMT3L
ACKR3	752.384917	-0.55970747	0.14057012	-3.98169583	6.84E-05	0.00012874	Down	ACKR3
NLRC4	51.1219439	-0.5216768	0.13103725	-3.98113353	6.86E-05	0.00012902	Down	NLRC4
ONECUT1	1.45962878	1.75756803	0.44150661	3.98084196	6.87E-05	0.00012916	Up	ONECUT1
TAS2R20	11.8571997	0.84675159	0.21276004	3.9798431	6.90E-05	0.00012969	Up	TAS2R20
MAGEB2	5.79196011	3.53630113	0.88856839	3.97977373	6.90E-05	0.00012972	Up	MAGEB2
PRH1	8.24581467	0.81673437	0.20529602	3.97832532	6.94E-05	0.00013048	Up	PRH1
TGM2	6427.97993	0.58856231	0.14817526	3.97206867	7.13E-05	0.0001339	Up	TGM2
SLC25A47	6.09927337	0.87735527	0.22088738	3.97195733	7.13E-05	0.00013395	Up	SLC25A47
LRP4	3337.64648	0.8544829	0.21522139	3.97025077	7.18E-05	0.00013487	Up	LRP4
HSPA12B	168.631482	-0.540847	0.13639071	-3.96542397	7.33E-05	0.00013747	Down	HSPA12B
ENKUR	26.2737631	-0.73029422	0.18418165	-3.96507583	7.34E-05	0.00013766	Down	ENKUR
SERTM1	1.64862897	-2.32862408	0.58799536	-3.96027626	7.49E-05	0.0001404	Down	SERTM1
ADCYAP1	37.0390029	-1.02985938	0.26007373	-3.95987474	7.50E-05	0.00014062	Down	ADCYAP1
GJA4	238.977282	0.55501984	0.14021414	3.95837286	7.55E-05	0.00014147	Up	GJA4
LMO1	1.22299581	-1.75546935	0.44348608	-3.95834145	7.55E-05	0.00014148	Down	LMO1
ABCA2	2935.58204	0.50615683	0.12787425	3.9582389	7.55E-05	0.00014151	Up	ABCA2
RFPL2	2.17562524	1.49173274	0.37689874	3.95791388	7.56E-05	0.00014169	Up	RFPL2

STKLD1	18.0239611	0.53560579	0.13545588	3.95409769	7.68E-05	0.00014392	Up	STKLD1
CYP4F22	5.41275376	-0.76633378	0.19381077	-3.95403097	7.68E-05	0.00014395	Down	CYP4F22
ZFHX4	97.1010479	-1.08386724	0.27420729	-3.95272945	7.73E-05	0.00014472	Down	ZFHX4
MIB2	1522.39481	0.54604906	0.13814626	3.95268801	7.73E-05	0.00014473	Up	MIB2
UNC45B	2.06425091	-0.91017024	0.23029293	-3.95222822	7.74E-05	0.00014498	Down	UNC45B
DOK6	35.5986347	-0.7105353	0.17980975	-3.95159497	7.76E-05	0.00014533	Down	DOK6
ECM2	156.19722	-0.78966958	0.19992154	-3.94989739	7.82E-05	0.00014633	Down	ECM2
TEKT1	1.5025146	1.83228802	0.46399417	3.94894622	7.85E-05	0.00014687	Up	TEKT1
PLA2G4F	775.828002	-0.6388782	0.16194024	-3.94514778	7.98E-05	0.00014909	Down	PLA2G4F
PSAPL1	4.50794585	2.11052041	0.53511343	3.94406173	8.01E-05	0.00014974	Up	PSAPL1
LAIR1	460.019817	-0.61450099	0.15582839	-3.94344697	8.03E-05	0.00015009	Down	LAIR1
FAM25A	0.92847459	2.489001	0.63155844	3.9410462	8.11E-05	0.00015157	Up	FAM25A
KCNMB3	49.4893008	0.60569183	0.15371125	3.94045206	8.13E-05	0.00015191	Up	KCNMB3
FCRL6	16.9052988	-0.86288094	0.21904701	-3.93924997	8.17E-05	0.00015264	Down	FCRL6
ANKDD1B	111.775459	-0.70108838	0.1780462	-3.93767682	8.23E-05	0.00015358	Down	ANKDD1B
CYP26C1	1.93004923	1.30074825	0.3304716	3.93603644	8.28E-05	0.00015459	Up	CYP26C1
DUOXA1	47.1019221	0.8701938	0.22111014	3.93556704	8.30E-05	0.00015488	Up	DUOXA1
FUT9	3.08286469	-2.85709985	0.72633757	-3.93357024	8.37E-05	0.00015614	Down	FUT9
EPO	1.6521065	1.10752724	0.28162196	3.93267354	8.40E-05	0.00015669	Up	EPO
KCNJ2	350.747691	-0.5834057	0.14846246	-3.92965134	8.51E-05	0.00015859	Down	KCNJ2
CLEC2D	150.419833	-0.60506581	0.15415333	-3.92509069	8.67E-05	0.00016144	Down	CLEC2D
ERVW-1	10.1214201	1.28841819	0.32827544	3.9248084	8.68E-05	0.00016161	Up	ERVW-1
NXPH2	0.52710202	-2.36313283	0.60214216	-3.92454302	8.69E-05	0.00016177	Down	NXPH2
NCKAP5	103.704773	-0.8331971	0.21233397	-3.92399342	8.71E-05	0.00016212	Down	NCKAP5
TNF	41.3573054	-0.6299441	0.16063233	-3.92165193	8.79E-05	0.00016365	Down	TNF
ZFP36	6215.4993	-0.50604011	0.12904828	-3.92132392	8.81E-05	0.00016384	Down	ZFP36
CEACAM19	191.898332	0.60235391	0.1536988	3.91905418	8.89E-05	0.00016529	Up	CEACAM19
AKR1B15	15.4751583	-1.27181184	0.32457648	-3.9183734	8.91E-05	0.00016574	Down	AKR1B15
TRPM1	1.94999734	2.14809323	0.54889649	3.91347597	9.10E-05	0.00016905	Up	TRPM1
CLCA2	20.1221764	-1.43625171	0.36711121	-3.91230693	9.14E-05	0.00016985	Down	CLCA2
IL5	2.93770127	1.13867044	0.29106512	3.91208134	9.15E-05	0.00017	Up	IL5
HORMAD1	16.1463708	1.40075378	0.35832179	3.90920626	9.26E-05	0.00017201	Up	HORMAD1
PURG	3.33362237	-0.90107139	0.23066855	-3.90634693	9.37E-05	0.00017397	Down	PURG
PDLIM4	363.71715	-0.68359787	0.17505173	-3.90511924	9.42E-05	0.00017482	Down	PDLIM4
DCAF12L1	0.42467254	-2.29249422	0.58755174	-3.90177417	9.55E-05	0.00017722	Down	DCAF12L1
AQP12B	9.12169991	-1.00789627	0.25838204	-3.90079848	9.59E-05	0.00017788	Down	AQP12B
CD226	59.4694391	-0.75593969	0.19388598	-3.89888788	9.66E-05	0.00017927	Down	CD226
KCNC3	75.0127613	0.6053859	0.15534019	3.89716219	9.73E-05	0.00018049	Up	KCNC3
AK1	381.301463	-0.50699469	0.13014899	-3.89549471	9.80E-05	0.0001817	Down	AK1
CARD9	147.899175	0.75442711	0.19381645	3.89248234	9.92E-05	0.00018394	Up	CARD9
OOSP1	1.78453841	1.2843955	0.3301243	3.89064206	1.00E-04	0.00018532	Up	OOSP1
MPO	11.4435496	0.82260117	0.21150984	3.88918635	0.00010058	0.00018637	Up	MPO
LRRC25	190.711153	-0.61162785	0.15729893	-3.88831533	0.00010094	0.00018702	Down	LRRC25
MTSS1	819.642722	-0.55208901	0.14198938	-3.88824148	0.00010097	0.00018704	Down	MTSS1
IL9R	10.9463169	-0.74438745	0.19164826	-3.88413355	0.0001027	0.00019014	Down	IL9R
GBP6	3.72416826	1.20653338	0.31099468	3.87959496	0.00010463	0.00019356	Up	GBP6
FPR2	66.4549284	1.10756665	0.28561599	3.87781739	0.0001054	0.00019496	Up	FPR2
STAR	4.61681899	-0.8978205	0.23158108	-3.87691652	0.00010579	0.00019564	Down	STAR
CTRL	1.82018163	1.00841166	0.26012969	3.87657268	0.00010594	0.00019587	Up	CTRL
FAM228A	3.2609431	-0.85326261	0.22013717	-3.87604969	0.00010617	0.00019623	Down	FAM228A
LIPK	0.59020858	2.17407975	0.56099797	3.875379	0.00010646	0.00019675	Up	LIPK
INSC	55.7418095	-0.74572154	0.19243606	-3.87516527	0.00010655	0.00019691	Down	INSC
KRT84	2.0237338	2.094531	0.54055457	3.87478182	0.00010672	0.00019718	Up	KRT84
SAMD5	1232.39428	0.81539671	0.21074821	3.86905643	0.00010926	0.0002018	Up	SAMD5
G0S2	534.084671	0.76515225	0.19791295	3.86610494	0.00011059	0.00020413	Up	G0S2
ZNF541	27.5337387	1.0692779	0.27665178	3.8650679	0.00011106	0.00020496	Up	ZNF541
CALML5	1.53181523	2.47087114	0.63947148	3.86392705	0.00011158	0.00020588	Up	CALML5
AK8	27.9577092	0.60247013	0.1561544	3.85816946	0.00011424	0.00021068	Up	AK8

ZNF793	81.72041	-0.89739866	0.2328119	-3.8546082	0.00011592	0.00021363	Down	ZNF793
PRICKLE1	68.6001658	-0.73488011	0.1906567	-3.85446783	0.00011598	0.00021373	Down	PRICKLE1
ARHGAP4	1822.08653	0.75554394	0.19618722	3.85113733	0.00011757	0.00021662	Up	ARHGAP4
CD80	32.3762633	0.69024915	0.17923417	3.85110251	0.00011759	0.00021663	Up	CD80
UBE2E2	217.733686	-0.61148698	0.15897366	-3.84646721	0.00011983	0.00022065	Down	UBE2E2
CCL3	177.411658	0.78585482	0.20436594	3.84533167	0.00012039	0.00022161	Up	CCL3
PKD2L2	4.69321608	1.15534016	0.30046296	3.8452	0.00012045	0.0002217	Up	PKD2L2
CD300A	286.704881	-0.60307105	0.15701798	-3.84077706	0.00012265	0.00022555	Down	CD300A
LRP2BP	79.1554753	0.65933592	0.17168461	3.84039038	0.00012284	0.00022588	Up	LRP2BP
ADAMTS13	141.437947	0.58675758	0.15280422	3.83993043	0.00012307	0.00022624	Up	ADAMTS13
CDC42EP5	2327.01188	-0.65654503	0.17101683	-3.83906676	0.0001235	0.00022701	Down	CDC42EP5
GNA14	203.691672	-0.55015823	0.14339625	-3.83662928	0.00012473	0.00022925	Down	GNA14
POLR2L	3410.27009	0.54942628	0.14323874	3.83573796	0.00012519	0.00023004	Up	POLR2L
STEAP4	212.168085	-0.88201988	0.22999307	-3.83498457	0.00012557	0.00023069	Down	STEAP4
HEG1	1266.03911	-0.58156822	0.15165687	-3.83476349	0.00012569	0.00023088	Down	HEG1
MDK	5553.02562	0.63432965	0.16546726	3.83356594	0.0001263	0.00023198	Up	MDK
PTPN5	10.7874796	-0.87721064	0.22883935	-3.83330338	0.00012643	0.00023218	Down	PTPN5
KIR3DL2	1.86962914	-1.35152166	0.35327933	-3.82564606	0.00013043	0.00023937	Down	KIR3DL2
FAM71F2	35.8888883	0.64652457	0.16903052	3.82489838	0.00013083	0.00024007	Up	FAM71F2
SLC4A1	2.24030789	-1.27733348	0.33395721	-3.82484178	0.00013086	0.0002401	Down	SLC4A1
CATSPER2	130.786229	0.54856921	0.1434895	3.82306162	0.0001318	0.00024172	Up	CATSPER2
HSPG2	9060.1931	-0.57362904	0.15016733	-3.81993245	0.00013349	0.00024471	Down	HSPG2
SPDEF	943.178462	-0.99796541	0.26125437	-3.81989942	0.00013351	0.00024472	Down	SPDEF
CFAP99	1.27153326	1.41740022	0.37132959	3.81709473	0.00013503	0.00024741	Up	CFAP99
FRMD7	0.68008821	1.99720133	0.52324713	3.81693701	0.00013512	0.00024752	Up	FRMD7
SNED1	326.052044	-0.52904272	0.13873378	-3.81336627	0.00013709	0.000251	Down	SNED1
LRRC2	192.091736	0.71311825	0.18711468	3.81112932	0.00013833	0.00025315	Up	LRRC2
RET	129.250566	-0.95687557	0.25126054	-3.80830027	0.00013993	0.00025588	Down	RET
PCYT1B	11.7307495	-0.98631993	0.25903815	-3.80762425	0.00014031	0.00025655	Down	PCYT1B
KLHDC7A	66.0472725	-0.90138044	0.23674132	-3.80744875	0.00014041	0.00025671	Down	KLHDC7A
HLA-G	309.456697	-0.71533296	0.18790786	-3.80682837	0.00014076	0.00025727	Down	HLA-G
ZNF714	259.221594	0.5299099	0.13920325	3.80673505	0.00014081	0.00025734	Up	ZNF714
DUSP23	1395.56491	0.53610064	0.14093488	3.80388888	0.00014244	0.00026024	Up	DUSP23
NT5DC3	974.029555	-0.51439284	0.13529482	-3.80201439	0.00014352	0.00026211	Down	NT5DC3
HOXD8	181.448231	-0.64508823	0.16969085	-3.80154988	0.00014379	0.00026257	Down	HOXD8
TAP1	7844.22188	0.50851234	0.13377136	3.80135442	0.00014391	0.00026276	Up	TAP1
SIGLEC7	41.6087964	-0.68711865	0.18083343	-3.79973234	0.00014485	0.00026443	Down	SIGLEC7
CMTM2	15.2012147	0.68758233	0.18105064	3.79773478	0.00014602	0.00026651	Up	CMTM2
CSAG2	2.32233547	3.89118865	1.02545581	3.79459417	0.00014789	0.00026983	Up	CSAG2
OR2H2	0.78761876	1.74392863	0.45977534	3.79300165	0.00014884	0.00027142	Up	OR2H2
C2orf50	5.81195303	0.73012536	0.19256017	3.79167373	0.00014964	0.00027285	Up	C2orf50
IL17D	71.9294845	0.52474586	0.13842951	3.79070799	0.00015022	0.00027389	Up	IL17D
HOXA11	498.506227	0.53109929	0.14019925	3.78817499	0.00015176	0.00027658	Up	HOXA11
TUBA8	5.99974519	0.67850307	0.17910984	3.78819545	0.00015175	0.00027658	Up	TUBA8
RAX	0.74065689	2.17746024	0.57515452	3.78587	0.00015317	0.00027901	Up	RAX
ANKRD7	1.08234055	-1.20587076	0.31855182	-3.78547754	0.00015341	0.00027943	Down	ANKRD7
CAV2	646.295672	-0.6022377	0.15918367	-3.78328815	0.00015477	0.00028181	Down	CAV2
SLC2A2	4.05784115	1.87462082	0.49552907	3.78306934	0.00015491	0.00028203	Up	SLC2A2
GLB1L3	5.06933985	-1.27315981	0.33666584	-3.78167207	0.00015578	0.00028353	Down	GLB1L3
LIN28B	3.95620764	3.7253365	0.98594752	3.77843285	0.00015782	0.0002871	Up	LIN28B
KCNA7	1.45498886	1.73994056	0.46049853	3.77838464	0.00015785	0.00028712	Up	KCNA7
C14orf39	1.34203288	-1.64246097	0.43498165	-3.77593163	0.00015941	0.00028985	Down	C14orf39
ZNF843	15.1300575	-0.53592469	0.14193896	-3.77574069	0.00015953	0.00029004	Down	ZNF843
TIGD7	28.9107942	-0.52236098	0.13839921	-3.77430613	0.00016045	0.00029162	Down	TIGD7
OTC	115.815348	-1.25111255	0.33149112	-3.77419625	0.00016052	0.00029172	Down	OTC
PRSS54	0.48537438	1.61892657	0.42895321	3.77413324	0.00016056	0.00029177	Up	PRSS54
PCDHB3	20.8904656	-0.77547232	0.20551478	-3.77331654	0.00016109	0.00029266	Down	PCDHB3
AMIGO3	0.85806518	1.19497092	0.31680899	3.77189712	0.00016201	0.00029418	Up	AMIGO3

PHF21B	1.79330235	-1.16008951	0.30758512	-3.77160474	0.0001622	0.00029447	Down	PHF21B
SPATA22	1.21031938	-1.55540834	0.41253236	-3.77039114	0.00016299	0.00029585	Down	SPATA22
PCDHGC4	4.70493553	-0.99281823	0.26336737	-3.7697085	0.00016344	0.0002966	Down	PCDHGC4
HTR1F	5.8678455	0.89554421	0.23764426	3.76842344	0.00016428	0.0002981	Up	HTR1F
C9	2.70002558	1.09186947	0.28977145	3.76803678	0.00016454	0.00029853	Up	C9
MVD	2305.57371	0.50010586	0.13273317	3.76775351	0.00016472	0.00029884	Up	MVD
KCNK17	25.8907593	0.91768804	0.24396128	3.76161351	0.00016882	0.00030611	Up	KCNK17
CIB2	339.914777	-0.57019582	0.15160314	-3.76110837	0.00016916	0.00030667	Down	CIB2
PKD2L1	9.95730985	-0.74986954	0.19939098	-3.76079964	0.00016937	0.00030702	Down	PKD2L1
TRIM17	21.5281123	0.7680475	0.20428655	3.75965778	0.00017015	0.0003083	Up	TRIM17
GPRC5B	333.346	-0.5173975	0.13761843	-3.75965261	0.00017015	0.0003083	Down	GPRC5B
BCL2A1	171.028597	0.7992953	0.21261534	3.75934915	0.00017036	0.00030865	Up	BCL2A1
TAS2R14	30.1830054	0.6602203	0.17567544	3.75818213	0.00017115	0.00031006	Up	TAS2R14
KCNK13	26.6535703	-0.60560205	0.1612811	-3.7549474	0.00017338	0.00031399	Down	KCNK13
SPAG16	317.3809	-0.64633434	0.17226247	-3.75203229	0.00017541	0.00031748	Down	SPAG16
BATF3	64.3645049	0.53886797	0.14375064	3.74863009	0.0001778	0.00032171	Up	BATF3
SDR42E1	405.319529	-0.62380453	0.16644268	-3.74786392	0.00017835	0.00032263	Down	SDR42E1
GPX8	521.615621	0.54485	0.14538443	3.74765016	0.0001785	0.00032288	Up	GPX8
SH3D21	403.718623	0.58104657	0.15511887	3.74581495	0.00017981	0.00032518	Up	SH3D21
CSMD1	6.52732409	-1.2588103	0.33621537	-3.74405932	0.00018107	0.00032734	Down	CSMD1
KANK3	97.9528815	-0.55048242	0.14718717	-3.74001642	0.00018401	0.00033247	Down	KANK3
CYP1A1	9.06894	1.84457717	0.49336939	3.7387345	0.00018495	0.00033403	Up	CYP1A1
ZNF716	1.74656501	3.51549195	0.94031877	3.73861721	0.00018504	0.00033412	Up	ZNF716
FRY	520.195962	-0.55657817	0.14888827	-3.73822706	0.00018532	0.00033457	Down	FRY
FOXD4L4	0.7845818	1.52873004	0.40919063	3.73598493	0.00018698	0.00033743	Up	FOXD4L4
GJA5	207.033836	-0.5596965	0.14988496	-3.73417379	0.00018833	0.0003398	Down	GJA5
SERPINA5	57.0701101	-0.87260776	0.2336865	-3.73409578	0.00018839	0.00033987	Down	SERPINA5
RCVRN	3.35697027	-0.73740359	0.19755161	-3.73271368	0.00018943	0.00034167	Down	RCVRN
MRAS	303.749014	-0.57020894	0.15276689	-3.73254267	0.00018956	0.00034187	Down	MRAS
LIMD2	439.398893	-0.51698146	0.13854437	-3.73152268	0.00019033	0.00034319	Down	LIMD2
C7orf61	6.06234948	0.67660979	0.18143238	3.72926696	0.00019204	0.00034607	Up	C7orf61
FCGR1A	60.9736783	0.73134064	0.1961376	3.72871203	0.00019246	0.0003468	Up	FCGR1A
SAMD9L	655.800611	-0.64963329	0.17431207	-3.7268406	0.00019389	0.00034917	Down	SAMD9L
MYRFL	27.7922905	1.15090565	0.30891296	3.7256632	0.0001948	0.0003507	Up	MYRFL
ZNF521	168.441103	-0.6005907	0.16125205	-3.72454612	0.00019567	0.00035215	Down	ZNF521
C3AR1	307.254655	-0.63985179	0.17192525	-3.72168585	0.0001979	0.00035594	Down	C3AR1
SATB1	1152.20799	-0.55665424	0.1496198	-3.72045848	0.00019886	0.00035761	Down	SATB1
CLC	73.6942094	-1.38251959	0.37161029	-3.72034799	0.00019895	0.00035773	Down	CLC
IL20RB	43.0497665	0.65659871	0.17662809	3.71740826	0.00020128	0.00036184	Up	IL20RB
HERC5	142.106739	-0.60339853	0.16232398	-3.71724825	0.0002014	0.00036203	Down	HERC5
CNTNAP4	2.0603619	-2.17214803	0.5845418	-3.7159841	0.00020241	0.00036381	Down	CNTNAP4
PRR27	0.86942014	2.41349364	0.64967775	3.71490888	0.00020328	0.00036525	Up	PRR27
CRLF1	52.7913984	-0.54091403	0.14570242	-3.71245754	0.00020526	0.00036862	Down	CRLF1
DIO3	183.750704	0.91504628	0.24653968	3.71155791	0.00020599	0.00036982	Up	DIO3
C1S	6347.5986	-0.60072701	0.16185665	-3.71147564	0.00020605	0.00036991	Down	C1S
NELL2	132.43842	-0.93733704	0.25266585	-3.70978921	0.00020743	0.00037234	Down	NELL2
LRRTM4	5.72412423	-1.29711287	0.34969025	-3.70931946	0.00020782	0.00037296	Down	LRRTM4
BARX1	16.0582112	1.27901618	0.34488134	3.70856884	0.00020843	0.00037403	Up	BARX1
GNB4	434.698068	-0.60807074	0.16407378	-3.70608106	0.00021049	0.00037761	Down	GNB4
CD300LF	99.8114347	-0.6197926	0.1672433	-3.70593386	0.00021061	0.00037775	Down	CD300LF
PLK2	885.303132	0.54770974	0.14788464	3.70362837	0.00021254	0.00038109	Up	PLK2
UNC13C	2.16563105	-1.40199736	0.37890137	-3.70016436	0.00021546	0.00038609	Down	UNC13C
FER1L5	7.48701418	0.91473863	0.24753006	3.69546487	0.00021948	0.00039303	Up	FER1L5
ANTXR1	3728.71986	0.70235335	0.19012205	3.69422348	0.00022056	0.00039483	Up	ANTXR1
KLF12	359.652552	-0.66727307	0.18064268	-3.69388377	0.00022085	0.00039532	Down	KLF12
VASN	431.516952	0.53759249	0.14554021	3.69377301	0.00022095	0.00039546	Up	VASN
NR2E1	0.75931234	2.18177435	0.59105084	3.69134802	0.00022307	0.00039917	Up	NR2E1
ID3	2949.89285	-0.53702635	0.14577446	-3.68395357	0.00022964	0.0004104	Down	ID3

GRK7	2.9016003	0.8255549	0.22420642	3.68211988	0.0002313	0.00041328	Up	GRK7
SLC22A1	11.2277405	0.58005948	0.15755507	3.68163012	0.00023175	0.00041403	Up	SLC22A1
IQCA1	18.6044722	0.74767537	0.2032732	3.67817976	0.0002349	0.00041955	Up	IQCA1
C4orf47	8.8074839	0.74108848	0.20157	3.67658119	0.00023638	0.00042206	Up	C4orf47
FAM153A	3.44806658	-1.08392831	0.2951827	-3.67205901	0.0002406	0.0004293	Down	FAM153A
FOXB1	2.23869456	1.33095855	0.36248519	3.6717598	0.00024089	0.00042976	Up	FOXB1
FAM184A	28.2708594	-0.96721685	0.26345595	-3.67126596	0.00024135	0.0004305	Down	FAM184A
GPBAR1	68.6417553	-0.59769698	0.16294385	-3.66811617	0.00024434	0.00043571	Down	GPBAR1
TNIK	1191.4281	-0.52273082	0.14255135	-3.66696495	0.00024545	0.00043759	Down	TNIK
PSMB9	3036.25175	0.55385336	0.15104402	3.66683418	0.00024557	0.00043777	Up	PSMB9
RASL11B	33.5939712	0.73188907	0.19983646	3.66244004	0.00024982	0.00044521	Up	RASL11B
CNTN6	1.79535056	-1.32138963	0.36099363	-3.66042362	0.0002518	0.00044864	Down	CNTN6
FAM178B	36.197331	0.87193546	0.2384022	3.65741372	0.00025477	0.00045376	Up	FAM178B
TUBB1	13.4149796	0.62350238	0.17070409	3.65253343	0.00025967	0.0004622	Up	TUBB1
KCNAB3	33.4071849	0.52824757	0.14463565	3.65226387	0.00025994	0.00046259	Up	KCNAB3
LRRIQ3	26.217696	0.67015981	0.18364263	3.64926057	0.000263	0.00046794	Up	LRRIQ3
TRIM31	3129.73514	-0.60925053	0.16720527	-3.64372814	0.00026872	0.00047764	Down	TRIM31
GABRA4	32.5514371	1.31446084	0.3607779	3.64340731	0.00026905	0.00047819	Up	GABRA4
GAS1	253.405924	-0.95942032	0.26334523	-3.64320373	0.00026927	0.00047852	Down	GAS1
EDARADD	15.4451502	0.70820708	0.19448339	3.64147839	0.00027108	0.00048164	Up	EDARADD
FAM177B	74.6895095	-1.06712624	0.29323273	-3.63917846	0.00027351	0.00048582	Down	FAM177B
COL11A2	40.5855431	0.77749413	0.21367648	3.63865101	0.00027407	0.00048675	Up	COL11A2
SRPK3	146.153753	0.6958175	0.19158494	3.63190073	0.00028134	0.00049929	Up	SRPK3
LYST	558.727823	-0.50566234	0.13927026	-3.63079925	0.00028254	0.00050132	Down	LYST
FGF1	49.3653562	-0.6897758	0.19008855	-3.62870776	0.00028484	0.00050525	Down	FGF1
CLDN19	1.689349	-1.42336114	0.39237388	-3.62756342	0.00028611	0.00050739	Down	CLDN19
RANBP3L	6.32069736	-1.02291014	0.28199957	-3.62734646	0.00028635	0.00050777	Down	RANBP3L
ANG	687.78268	-0.5164408	0.14252614	-3.62348136	0.00029066	0.00051516	Down	ANG
DCHS2	42.4262714	-1.21574042	0.33561296	-3.62244774	0.00029183	0.00051702	Down	DCHS2
TGM6	2.53376549	2.75362248	0.76039358	3.6213121	0.00029311	0.00051919	Up	TGM6
GNRH2	2.73711749	0.95896168	0.26485933	3.62064533	0.00029387	0.00052048	Up	GNRH2
P2RX6	11.4263317	-0.78123269	0.21578192	-3.62047331	0.00029406	0.00052073	Down	P2RX6
UPK3B	33.2842929	0.7933627	0.21921613	3.61908909	0.00029564	0.00052321	Up	UPK3B
NTF3	19.1977183	-0.59647237	0.16484414	-3.61840204	0.00029643	0.00052455	Down	NTF3
SALL3	1.92277025	3.08525338	0.85291422	3.61730795	0.00029768	0.00052672	Up	SALL3
MSANTD1	10.6365713	0.59284925	0.16407242	3.61333878	0.00030228	0.00053453	Up	MSANTD1
KCNJ13	4.19326328	1.8015079	0.49861869	3.61299713	0.00030268	0.00053502	Up	KCNJ13
VGLL3	232.862053	-0.88207992	0.24438539	-3.60938078	0.00030693	0.00054227	Down	VGLL3
CCDC74B	24.8702448	0.63217445	0.17523663	3.60754729	0.00030911	0.00054606	Up	CCDC74B
MAP3K15	5.47023633	-0.71100512	0.19711881	-3.60698774	0.00030977	0.00054718	Down	MAP3K15
CDK5R2	16.8899872	-1.05269816	0.29186011	-3.6068587	0.00030993	0.0005474	Down	CDK5R2
SYCE1L	62.0553912	0.7513392	0.20832314	3.60660464	0.00031023	0.00054788	Up	SYCE1L
MED12L	9.79672602	-0.83402579	0.23125291	-3.60655269	0.00031029	0.00054794	Down	MED12L
ABCC6	406.016008	-0.54964849	0.15251881	-3.60380793	0.00031359	0.00055358	Down	ABCC6
ST3GAL6	81.2059586	-0.60254133	0.16724864	-3.60266798	0.00031497	0.00055588	Down	ST3GAL6
TAS2R31	4.91554422	1.20777902	0.33524634	3.60266131	0.00031498	0.00055588	Up	TAS2R31
SLC7A3	2.99371857	-1.08576084	0.30138344	-3.60258964	0.00031506	0.00055598	Down	SLC7A3
GOLGA8H	3.44103326	-0.61768175	0.17146935	-3.60228671	0.00031543	0.00055657	Down	GOLGA8H
SRGN	1771.52227	-0.63471952	0.17621296	-3.60200255	0.00031578	0.00055713	Down	SRGN
SACS	409.00073	0.57451349	0.15950817	3.60178106	0.00031604	0.00055755	Up	SACS
PRDM7	1.2283179	1.85498173	0.51586206	3.59588708	0.00032329	0.00057015	Up	PRDM7
ZNF257	19.8879925	-1.0444153	0.29050073	-3.59522433	0.00032411	0.0005715	Down	ZNF257
AP3B2	28.0787084	-1.07603006	0.29942196	-3.59369117	0.00032603	0.00057481	Down	AP3B2
GPR182	5.56854333	-0.76235139	0.21216197	-3.59325186	0.00032658	0.00057561	Down	GPR182
CCL2	497.379891	-0.63929785	0.17794419	-3.59268744	0.00032729	0.00057681	Down	CCL2
RDH8	1.90628964	1.51144786	0.42078946	3.59193371	0.00032823	0.00057825	Up	RDH8
SEC14L3	1.06179263	1.79612633	0.50004449	3.59193305	0.00032823	0.00057825	Up	SEC14L3
MEDAG	152.244043	-0.7485834	0.20849565	-3.59040287	0.00033017	0.00058148	Down	MEDAG

HSPE1-MOB4	2.46248364	0.74551777	0.20776905	3.58820413	0.00033296	0.00058623	Up	HSPE1-MOB4
APOC1	617.243016	0.74138657	0.20664191	3.58778409	0.0003335	0.00058706	Up	APOC1
CALHM1	2.52491828	-0.98168653	0.27369704	-3.58676337	0.00033481	0.00058925	Down	CALHM1
STRC	6.32234864	0.79024139	0.2204207	3.58515049	0.00033688	0.00059267	Up	STRC
RARRES1	983.743133	-0.78344288	0.21879931	-3.58064607	0.00034275	0.0006028	Down	RARRES1
CD1B	10.3092796	0.87849336	0.24535091	3.58055881	0.00034286	0.00060294	Up	CD1B
SELPLG	510.215937	-0.52394559	0.14636044	-3.57983055	0.00034382	0.00060457	Down	SELPLG
GPR82	33.5460221	-0.8622268	0.24091557	-3.57895835	0.00034497	0.00060647	Down	GPR82
FBXW10	1.73707951	1.40791052	0.39347023	3.57818819	0.00034598	0.00060814	Up	FBXW10
HLA-DQA2	371.101825	-0.92606777	0.25888892	-3.57708538	0.00034745	0.00061059	Down	HLA-DQA2
PAGE1	1.70846956	3.25136608	0.90904883	3.57666826	0.000348	0.00061144	Up	PAGE1
PLET1	1.21262455	2.46889894	0.69054338	3.57529885	0.00034983	0.00061459	Up	PLET1
MAP10	51.2583076	-0.78305569	0.21919421	-3.57242864	0.00035369	0.00062094	Down	MAP10
CARD11	607.598798	0.8405028	0.23528434	3.57228537	0.00035388	0.00062122	Up	CARD11
C8orf34	8.08590218	0.74118941	0.20749975	3.57200156	0.00035426	0.00062183	Up	C8orf34
CNGB3	5.42460014	1.16765959	0.32696192	3.57124031	0.00035529	0.00062358	Up	CNGB3
CHST9	8.68325133	-1.9310144	0.54108928	-3.56875373	0.00035868	0.00062934	Down	CHST9
CHRM5	5.865962	-0.83343632	0.23362111	-3.56747008	0.00036044	0.00063225	Down	CHRM5
CGB8	0.77915075	2.6279885	0.73683772	3.56657706	0.00036167	0.00063434	Up	CGB8
SDR16C5	542.858353	0.93658422	0.26274648	3.56459284	0.00036442	0.00063904	Up	SDR16C5
OLFM4	63319.825	1.19541557	0.33536621	3.5645081	0.00036454	0.00063918	Up	OLFM4
CASP14	0.84449459	2.41410714	0.67764199	3.56251114	0.00036732	0.00064394	Up	CASP14
PAPLN	304.883208	-0.53214858	0.1494215	-3.56139239	0.00036889	0.0006465	Down	PAPLN
BPIFB1	3.9716834	2.06915257	0.58108545	3.56084044	0.00036967	0.00064773	Up	BPIFB1
BOLA2B	37.9274738	0.63906563	0.17958538	3.55856161	0.00037289	0.00065318	Up	BOLA2B
ANO7	343.767282	-0.78031122	0.21935912	-3.55723171	0.00037478	0.00065637	Down	ANO7
SFRP2	2353.1506	-1.12047436	0.31515627	-3.55529771	0.00037755	0.00066109	Down	SFRP2
KERA	4.54820046	-1.12033544	0.31512084	-3.55525658	0.00037761	0.00066112	Down	KERA
IL1RL1	41.8208104	-0.75191355	0.21149506	-3.55522985	0.00037765	0.00066113	Down	IL1RL1
AGBL4	11.9951164	1.02993115	0.2897348	3.55473748	0.00037836	0.0006623	Up	AGBL4
FOXA1	920.518017	-0.69205632	0.19479793	-3.55268825	0.00038132	0.00066735	Down	FOXA1
CTAG2	2.92804077	3.59845872	1.01303811	3.55214546	0.0003821	0.0006686	Up	CTAG2
MGAT5B	9.74932959	-0.87346818	0.24596891	-3.55113252	0.00038358	0.00067111	Down	MGAT5B
MMP24	110.477893	0.61047866	0.1719595	3.55013041	0.00038504	0.0006736	Up	MMP24
TFPI	616.114666	-0.57689303	0.16256339	-3.54872662	0.0003871	0.00067714	Down	TFPI
GALR2	10.4520229	-0.77439619	0.21828371	-3.54765906	0.00038867	0.00067969	Down	GALR2
MXRA8	1703.64903	0.55470588	0.15639526	3.54682026	0.00038991	0.00068159	Up	MXRA8
DGCR6	48.3880207	0.58923598	0.16616005	3.54619528	0.00039084	0.00068307	Up	DGCR6
MMP17	115.101605	0.76894005	0.21684185	3.54608699	0.000391	0.00068329	Up	MMP17
PSD2	10.8737822	0.81179966	0.22904249	3.54431905	0.00039363	0.00068755	Up	PSD2
NCR1	5.18522923	-0.88396915	0.2495952	-3.54161122	0.00039769	0.00069424	Down	NCR1
KRTAP10-4	0.79636979	1.60752585	0.45402349	3.54062262	0.00039918	0.00069669	Up	KRTAP10-4
ZNF618	464.913788	0.57209108	0.161679	3.53843769	0.0004025	0.00070229	Up	ZNF618
ANO4	10.5567448	-0.8290479	0.23431166	-3.53822723	0.00040282	0.00070278	Down	ANO4
KLRC4-KLRK1	3.30946019	-0.97041447	0.27437549	-3.53681177	0.00040499	0.00070649	Down	KLRC4-KLRK1
CLEC18C	0.89834422	1.65045094	0.46666589	3.53668645	0.00040518	0.00070676	Up	CLEC18C
HIGD2B	0.89072926	1.21320711	0.34305215	3.53650931	0.00040545	0.00070716	Up	HIGD2B
KRTAP10-2	0.75379397	1.82974118	0.51762203	3.53489818	0.00040793	0.00071128	Up	KRTAP10-2
PRSS55	0.97131551	2.18398258	0.61842137	3.53154451	0.00041314	0.00071987	Up	PRSS55
CD72	94.2982924	-0.51137135	0.14496446	-3.5275636	0.0004194	0.00073062	Down	CD72
COL24A1	59.2658648	0.79508085	0.22539214	3.52754464	0.00041943	0.00073062	Up	COL24A1
FAM124B	27.5859996	-0.59318173	0.16816029	-3.52747813	0.00041954	0.00073073	Down	FAM124B
SH2D1A	49.4753969	-0.69028536	0.19569997	-3.52726357	0.00041988	0.00073125	Down	SH2D1A
CASS4	61.4246656	-0.62060074	0.17610623	-3.52401347	0.00042506	0.00074007	Down	CASS4
MEIKIN	0.4256632	1.68192761	0.47746619	3.52261094	0.00042732	0.00074377	Up	MEIKIN
KCNJ3	110.977922	1.04966729	0.29809755	3.52122074	0.00042956	0.00074754	Up	KCNJ3
SLITRK5	20.3476689	-1.20326642	0.34184122	-3.5199571	0.00043162	0.00075096	Down	SLITRK5
CYP2B6	1139.8198	0.83619136	0.23758825	3.51949793	0.00043236	0.00075212	Up	CYP2B6

HBZ	2.83093848	3.71777365	1.05645461	3.519104	0.00043301	0.00075316	Up	HBZ
CLEC4D	7.8191005	1.11899894	0.31798787	3.5189988	0.00043318	0.00075339	Up	CLEC4D
ZCCHC18	12.6372044	-0.53158611	0.15109625	-3.51819518	0.00043449	0.0007556	Down	ZCCHC18
C5orf58	8.02708911	0.95845558	0.27248495	3.51746248	0.00043569	0.00075754	Up	C5orf58
KRT71	0.73456145	1.89180738	0.53785871	3.51729432	0.00043597	0.00075795	Up	KRT71
APOL1	3857.97839	0.61085486	0.17393011	3.51207074	0.00044463	0.00077255	Up	APOL1
RSPH10B	2.24498608	0.78640465	0.22391673	3.51204064	0.00044468	0.00077256	Up	RSPH10B
CLVS1	7.34370781	0.83135418	0.23674521	3.5115987	0.00044542	0.00077377	Up	CLVS1
KCNH3	20.9704853	0.77397713	0.22067768	3.50727424	0.00045272	0.00078608	Up	KCNH3
CXCR6	165.669347	-0.58339874	0.16643639	-3.5052354	0.0004562	0.00079189	Down	CXCR6
GPC5	10.3164013	-1.1911672	0.33987071	-3.50476569	0.00045701	0.00079321	Down	GPC5
SEC31B	205.414105	0.5191425	0.14818099	3.50343512	0.0004593	0.00079711	Up	SEC31B
C1orf53	107.010713	0.50601475	0.14444488	3.50316854	0.00045976	0.00079775	Up	C1orf53
LRTM2	1.67344571	-1.11426919	0.31814867	-3.50235381	0.00046117	0.00080004	Down	LRTM2
CSNK1A1L	1.58767064	1.40813393	0.40224208	3.50071264	0.00046402	0.00080482	Up	CSNK1A1L
RAG2	0.77461749	1.89338585	0.54089987	3.50043688	0.0004645	0.0008055	Up	RAG2
ACTN3	2.60238749	-0.71587526	0.20462693	-3.49844105	0.00046799	0.00081132	Down	ACTN3
CGA	1.93349163	1.56073873	0.44637617	3.49646518	0.00047147	0.00081703	Up	CGA
SLITRK4	13.001158	-1.07699738	0.3080673	-3.49598087	0.00047232	0.00081844	Down	SLITRK4
ARHGEF4	158.400702	-0.8007088	0.22909424	-3.49510656	0.00047387	0.00082088	Down	ARHGEF4
GATM	597.251209	-0.67177867	0.19225444	-3.4942167	0.00047545	0.00082354	Down	GATM
TMCO2	0.51059917	1.35079105	0.38683458	3.49190875	0.00047958	0.00083021	Up	TMCO2
C17orf50	2.83175594	-0.79747001	0.22838383	-3.49179713	0.00047978	0.00083048	Down	C17orf50
UBQLNL	5.56304891	-0.60382905	0.17299025	-3.49053799	0.00048205	0.00083416	Down	UBQLNL
URAD	259.054905	-1.28548306	0.36848983	-3.48851705	0.00048571	0.00084024	Down	URAD
HFM1	4.98812908	-0.99614165	0.28567173	-3.48701512	0.00048844	0.00084489	Down	HFM1
CRYBA1	1.779977	1.28880105	0.3698463	3.48469369	0.0004927	0.00085209	Up	CRYBA1
RHOV	249.393918	0.65224053	0.18719661	3.484254	0.00049351	0.00085341	Up	RHOV
ZNF256	72.549556	-0.67808334	0.19461779	-3.48417969	0.00049365	0.00085356	Down	ZNF256
HOXC9	13.2687324	1.14529726	0.32903836	3.48074091	0.00050003	0.00086443	Up	HOXC9
LTC4S	0.54523805	-0.93313497	0.26814315	-3.47998807	0.00050144	0.00086661	Down	LTC4S
ZKSCAN7	45.0415518	-0.59031776	0.1696728	-3.47915368	0.000503	0.00086915	Down	ZKSCAN7
HOXB4	218.606362	0.57472208	0.16520214	3.47890213	0.00050347	0.00086988	Up	HOXB4
CACNA1E	32.8502813	0.91332731	0.26297403	3.47307037	0.00051454	0.00088848	Up	CACNA1E
CUZD1	33.1823238	0.55564135	0.16010467	3.47048802	0.00051951	0.00089672	Up	CUZD1
GHRHR	2.28534689	1.1895809	0.34278976	3.47029297	0.00051989	0.00089729	Up	GHRHR
GPR33	0.40370248	-1.41675219	0.40834568	-3.46949231	0.00052144	0.00089979	Down	GPR33
KLF2	742.868159	-0.57111486	0.16472576	-3.46706473	0.00052618	0.00090761	Down	KLF2
CSN1S1	1.29128673	-1.3864386	0.40005315	-3.465636	0.00052898	0.00091218	Down	CSN1S1
MTRNR2L3	11.372343	1.0176796	0.29384636	3.46330516	0.00053358	0.00091985	Up	MTRNR2L3
CHRNB2	16.1782909	-0.86637061	0.250184	-3.4629337	0.00053432	0.00092104	Down	CHRNB2
ROBO3	110.434311	-0.55842033	0.16136175	-3.46067342	0.00053883	0.00092862	Down	ROBO3
NAGS	211.890481	-0.60362508	0.17447987	-3.45956868	0.00054104	0.00093235	Down	NAGS
CAMK2B	20.1270076	-1.00188156	0.28963741	-3.45908889	0.00054201	0.00093392	Down	CAMK2B
IDO1	559.970745	0.92053978	0.26619712	3.45811323	0.00054397	0.00093713	Up	IDO1
SLC1A3	148.635267	0.75833358	0.21930893	3.45783265	0.00054454	0.00093802	Up	SLC1A3
WDR38	2.36440355	1.1955156	0.34622805	3.45297148	0.00055445	0.00095426	Up	WDR38
LSMEM2	2.76184693	-0.83341306	0.24160226	-3.44952504	0.00056157	0.00096605	Down	LSMEM2
PRSS37	2.54647713	1.26249479	0.36617163	3.44782256	0.00056513	0.00097207	Up	PRSS37
SLC25A48	37.7288768	-0.78645365	0.22817819	-3.4466644	0.00056755	0.00097606	Down	SLC25A48
GPR55	41.4939865	-0.85028114	0.24671407	-3.44642338	0.00056806	0.00097674	Down	GPR55
SPIC	0.66812172	-1.15319657	0.33461353	-3.44635366	0.00056821	0.0009769	Down	SPIC
GOLGA6L10	16.2803587	0.55600854	0.16142678	3.44433887	0.00057246	0.00098373	Up	GOLGA6L10
CAPN8	1566.76793	0.56361423	0.16366349	3.44373824	0.00057373	0.00098573	Up	CAPN8
GPR42	0.73809129	-1.04164762	0.30252759	-3.44314912	0.00057498	0.00098769	Down	GPR42
AVPR2	25.133242	-0.80223601	0.23310357	-3.44154327	0.00057841	0.00099338	Down	AVPR2
PHACTR1	131.71002	-0.50500448	0.14680502	-3.43996742	0.00058178	0.0009989	Down	PHACTR1
KRT86	28.127017	0.71847654	0.20891657	3.43905967	0.00058374	0.00100215	Up	KRT86

FAM81B	1.44540542	1.80620289	0.52525819	3.43869537	0.00058452	0.00100331	Up	FAM81B
HLA-DOA	515.344376	-0.71335343	0.20748491	-3.43809799	0.00058582	0.00100524	Down	HLA-DOA
ROS1	22.5905868	-1.12762372	0.32800991	-3.43777336	0.00058652	0.00100635	Down	ROS1
TRPM5	95.3859544	-0.76821175	0.223598	-3.4356826	0.00059106	0.00101395	Down	TRPM5
SLC35F3	3.31322288	-0.96445794	0.28094157	-3.4329485	0.00059706	0.00102393	Down	SLC35F3
CDH22	6.45454789	-1.35889175	0.395884	-3.43255032	0.00059793	0.00102514	Down	CDH22
CLDND2	38.3817261	0.51072717	0.14895932	3.42863517	0.00060662	0.00103974	Up	CLDND2
PCDHGA3	6.59749878	-0.7559485	0.22077368	-3.42408804	0.00061687	0.00105628	Down	PCDHGA3
WFDC8	0.93277644	2.45835148	0.71851955	3.42141209	0.00062297	0.00106612	Up	WFDC8
SLC6A12	51.2190077	-0.67037209	0.1960316	-3.41971448	0.00062687	0.00107247	Down	SLC6A12
CYBB	1274.41871	-0.61957252	0.1813702	-3.41606577	0.00063533	0.00108654	Down	CYBB
OR6A2	0.83539438	2.58210638	0.75621419	3.41451722	0.00063895	0.00109263	Up	OR6A2
TRDN	2.39670426	-2.05886933	0.60309055	-3.41386436	0.00064048	0.00109494	Down	TRDN
IFNG	12.235574	0.94400641	0.27653325	3.41371749	0.00064083	0.00109532	Up	IFNG
CD8B	91.8512379	-0.64027245	0.18771835	-3.41081432	0.00064769	0.00110652	Down	CD8B
C9orf153	1.75376092	1.06970737	0.313757	3.40934984	0.00065118	0.00111226	Up	C9orf153
ANKRD62	0.87676074	-1.23641757	0.3635034	-3.40139203	0.00067044	0.00114395	Down	ANKRD62
KCNH2	639.409054	-0.63854585	0.18776578	-3.40075725	0.00067199	0.00114639	Down	KCNH2
GATA2	204.163214	-0.52834208	0.15538043	-3.4003128	0.00067309	0.00114815	Down	GATA2
ART3	82.0585598	1.0077448	0.29647303	3.3991112	0.00067605	0.00115287	Up	ART3
GSTA2	3.27037969	-0.98071426	0.2887668	-3.39621543	0.00068325	0.00116492	Down	GSTA2
MYRF	1484.11529	0.64901308	0.19116645	3.39501555	0.00068625	0.00116959	Up	MYRF
COL25A1	5.19110314	-1.00203549	0.29515868	-3.39490437	0.00068653	0.00116995	Down	COL25A1
RNF186	801.949996	-0.66623486	0.19626173	-3.39462434	0.00068723	0.00117104	Down	RNF186
CABP2	0.3849124	-2.51252485	0.74021222	-3.39433041	0.00068797	0.00117218	Down	CABP2
GNAT1	0.54463764	1.48589176	0.43814693	3.39130928	0.0006956	0.0011845	Up	GNAT1
GP9	4.19039996	-1.06105457	0.31312455	-3.3886023	0.0007025	0.0011958	Down	GP9
MATN4	6.2720103	-0.68992409	0.20364123	-3.38793911	0.0007042	0.00119858	Down	MATN4
ENTHD1	5.65797668	0.96920832	0.28626915	3.38565404	0.00071009	0.00120838	Up	ENTHD1
DACH2	3.31357023	-1.21366433	0.35849694	-3.3854245	0.00071068	0.00120916	Down	DACH2
NRXN3	259.62681	-0.82184121	0.24291301	-3.38327372	0.00071627	0.0012183	Down	NRXN3
BBOX1	4.65129369	1.14545813	0.33870313	3.38189411	0.00071988	0.0012241	Up	BBOX1
SAG	3.12950848	-0.88960664	0.26361575	-3.37463391	0.00073914	0.00125614	Down	SAG
INSM1	60.2242448	-1.12861278	0.33456723	-3.37335123	0.00074259	0.00126188	Down	INSM1
SYCE1	1.21412144	-0.7841078	0.23251298	-3.37231832	0.00074538	0.00126626	Down	SYCE1
KCNF1	26.2347981	-0.90776703	0.26930153	-3.37082011	0.00074945	0.00127281	Down	KCNF1
GRIP1	87.6269088	-0.60798842	0.18039499	-3.3703177	0.00075082	0.00127501	Down	GRIP1
HTR2A	17.4199676	-0.79974767	0.23745036	-3.3680626	0.00075698	0.00128475	Down	HTR2A
TTC9B	12.7532699	0.6565241	0.19496051	3.36747217	0.00075861	0.00128738	Up	TTC9B
KMO	36.1510381	-0.60041226	0.17840802	-3.3653883	0.00076436	0.00129628	Down	KMO
CFAP57	6.86815438	0.77696088	0.23096696	3.3639481	0.00076836	0.00130257	Up	CFAP57
ZNF736	392.688615	0.55899736	0.16628918	3.36159803	0.00077493	0.00131333	Up	ZNF736
HLA-DRA	14810.622	-0.62355217	0.18572931	-3.35731704	0.00078703	0.00133295	Down	HLA-DRA
CAPN11	15.3604393	-0.60433638	0.18008214	-3.35589289	0.00079109	0.00133958	Down	CAPN11
ARL9	15.3671551	0.74429981	0.22188235	3.35447952	0.00079514	0.00134606	Up	ARL9
SIT1	104.608931	-0.65191354	0.19442608	-3.35301491	0.00079936	0.00135307	Down	SIT1
PKD1L1	64.1296031	0.58601964	0.17488718	3.35084386	0.00080566	0.00136334	Up	PKD1L1
LHFPL1	1.23224137	-0.99848257	0.29829052	-3.34734938	0.00081588	0.00138012	Down	LHFPL1
VWF	3782.03519	-0.51154218	0.15284654	-3.34676969	0.00081759	0.00138288	Down	VWF
SPIN2A	7.08377433	0.9099681	0.27196244	3.34593306	0.00082006	0.00138678	Up	SPIN2A
RGS21	0.95635798	2.632151	0.78689764	3.34497255	0.00082291	0.00139121	Up	RGS21
SYNPO2L	3.57377403	0.85103276	0.2544728	3.34429758	0.00082491	0.00139433	Up	SYNPO2L
DLX2	7.20409148	1.04390734	0.31220319	3.34367933	0.00082675	0.00139705	Up	DLX2
GAMT	94.3071765	-0.61748025	0.18468385	-3.34344471	0.00082745	0.0013981	Down	GAMT
ZNF365	13.7987827	0.85634114	0.25616379	3.3429437	0.00082895	0.00140049	Up	ZNF365
DYNC2H1	120.082706	-0.65846321	0.19705832	-3.34146361	0.00083338	0.00140771	Down	DYNC2H1
TPPP	492.60218	-0.67033105	0.20062136	-3.34127456	0.00083395	0.00140854	Down	TPPP
LCNL1	5.49836446	0.88878982	0.26609136	3.34016794	0.00083728	0.00141363	Up	LCNL1

MYO3A	12.4243122	-1.12409759	0.33657806	-3.33978269	0.00083844	0.00141519	Down	MYO3A
RSPH10B2	0.95757124	1.08244444	0.32417304	3.33909459	0.00084052	0.00141843	Up	RSPH10B2
DAB1	43.6939659	0.83115454	0.24895038	3.33863529	0.00084191	0.00142051	Up	DAB1
SLC38A11	108.270752	0.93216243	0.27932689	3.33717394	0.00084635	0.00142773	Up	SLC38A11
MAGEA9	1.97822045	3.83271801	1.14920973	3.33509011	0.00085272	0.00143793	Up	MAGEA9
OR10A2	1.02719773	2.38036148	0.71400963	3.33379463	0.0008567	0.00144451	Up	OR10A2
LST1	211.487344	-0.51238594	0.15379514	-3.33161326	0.00086344	0.00145574	Down	LST1
NRK	31.3159648	-0.98683426	0.29651676	-3.32808932	0.00087444	0.00147316	Down	NRK
MN1	205.544169	-0.59989034	0.18028551	-3.32744627	0.00087646	0.00147629	Down	MN1
ATF3	1824.31038	-0.54179631	0.16284668	-3.32703317	0.00087776	0.0014782	Down	ATF3
HTR1B	6.3371899	0.70610464	0.21224792	3.32679187	0.00087852	0.0014792	Up	HTR1B
LRRC70	9.90355423	-0.59933718	0.18024809	-3.3250681	0.00088397	0.00148796	Down	LRRC70
BRINP2	4.49706339	-1.15258551	0.34664252	-3.32499744	0.00088419	0.00148819	Down	BRINP2
BTBD18	4.37850694	1.18703919	0.35705833	3.32449655	0.00088578	0.00149073	Up	BTBD18
RD3	1.69656963	-1.11916268	0.33674528	-3.32346955	0.00088905	0.00149609	Down	RD3
XCL1	15.735152	-0.88627509	0.26667638	-3.32341056	0.00088924	0.00149626	Down	XCL1
NINL	272.035298	0.72592402	0.21843965	3.32322462	0.00088983	0.00149712	Up	NINL
MPC1L	0.81650524	1.29373813	0.38932624	3.32301802	0.00089049	0.00149809	Up	MPC1L
ERMN	25.6736716	0.76319547	0.22970465	3.32250767	0.00089212	0.00150069	Up	ERMN
IGSF9B	68.5080985	-0.67053581	0.20189655	-3.32118514	0.00089636	0.00150739	Down	IGSF9B
TAS2R1	0.41495578	1.68417147	0.50714419	3.32089278	0.0008973	0.00150883	Up	TAS2R1
TACR3	0.79285063	-1.5841024	0.47726617	-3.31911728	0.00090302	0.00151803	Down	TACR3
CMPK2	403.886721	-0.53510472	0.16126095	-3.3182535	0.00090582	0.00152259	Down	CMPK2
CATSPER4	0.8259281	1.28395596	0.38721196	3.31589952	0.00091349	0.00153518	Up	CATSPER4
SPINK4	4258.94166	1.20237277	0.3626269	3.31572971	0.00091404	0.00153597	Up	SPINK4
MDGA2	0.9511751	-1.66127404	0.50108226	-3.31537188	0.00091521	0.00153765	Down	MDGA2
IL24	116.377689	0.77090425	0.23259504	3.31436236	0.00091852	0.00154306	Up	IL24
LYPD5	147.928568	0.57575247	0.17376583	3.31338147	0.00092175	0.0015482	Up	LYPD5
CARNS1	36.5998424	-0.62446746	0.18864354	-3.31030402	0.00093195	0.00156488	Down	CARNS1
ZNF518B	379.977798	-0.62530963	0.18897347	-3.30898102	0.00093636	0.00157214	Down	ZNF518B
MLC1	13.9648599	0.71095557	0.21489851	3.30833182	0.00093854	0.00157564	Up	MLC1
ELN	1783.16343	0.59463209	0.17983042	3.30662679	0.00094427	0.00158512	Up	ELN
HRNR	5.39726059	1.07031487	0.32399098	3.30353297	0.00095475	0.00160211	Up	HRNR
PTGER1	27.1036039	0.82262085	0.24905769	3.30293293	0.00095679	0.00160524	Up	PTGER1
CDK14	259.525997	-0.60997128	0.18469725	-3.30254673	0.00095811	0.0016073	Down	CDK14
SORCS2	140.649371	-0.57547421	0.17427502	-3.30210381	0.00095963	0.00160954	Down	SORCS2
DRC7	2.83690502	1.07448342	0.32565513	3.29945182	0.00096874	0.00162436	Up	DRC7
VAMP5	506.13265	-0.50632003	0.15353566	-3.29773583	0.00097468	0.00163402	Down	VAMP5
RSPH6A	0.95211112	1.77509107	0.53879469	3.29455936	0.00098576	0.00165213	Up	RSPH6A
NKG7	187.053828	-0.65373883	0.19849131	-3.29353887	0.00098935	0.00165798	Down	NKG7
TRPS1	203.270944	-0.67608011	0.20538727	-3.29173333	0.00099572	0.00166835	Down	TRPS1
ATP1A4	2.11068402	-0.80951914	0.24604976	-3.29006266	0.00100165	0.00167797	Down	ATP1A4
CPZ	31.1723359	0.61426225	0.18670729	3.28997469	0.00100196	0.00167834	Up	CPZ
OSR2	193.677383	-0.61578122	0.18720937	-3.28926491	0.00100449	0.00168242	Down	OSR2
CTRC	2.26688562	0.83619152	0.25457641	3.28463863	0.00102113	0.00170933	Up	CTRC
TNNI3K	1.68144299	-1.0257587	0.31237066	-3.28378698	0.00102422	0.00171434	Down	TNNI3K
VWA5B1	34.2674683	1.46491236	0.4462099	3.28301176	0.00102704	0.00171874	Up	VWA5B1
MUC7	1.37481031	2.24201355	0.68330031	3.28115402	0.00103383	0.00172994	Up	MUC7
CCL4	236.591709	0.62234543	0.18972223	3.28029795	0.00103698	0.00173483	Up	CCL4
IRX4	0.79627087	1.79761506	0.54825737	3.2787796	0.00104257	0.0017439	Up	IRX4
SMIM1	30.4918078	0.6515841	0.1987564	3.27830505	0.00104432	0.00174667	Up	SMIM1
TCERG1L	1.74154031	-0.78553695	0.23985243	-3.27508443	0.0010563	0.00176621	Down	TCERG1L
RETNLB	643.700999	-1.0807593	0.33029681	-3.27208518	0.00106757	0.0017834	Down	RETNLB
C4orf45	2.18877592	1.58478345	0.48433473	3.27208304	0.00106758	0.0017834	Up	C4orf45
SEMA7A	380.388275	0.51772053	0.15823304	3.2718863	0.00106833	0.00178431	Up	SEMA7A
TRIM74	2.3562487	0.69815162	0.2134565	3.27069742	0.00107283	0.00179149	Up	TRIM74
GJC2	147.106471	0.83189132	0.25435713	3.27056417	0.00107333	0.00179217	Up	GJC2
SLC16A10	193.461815	0.56991758	0.17429343	3.26987412	0.00107595	0.00179638	Up	SLC16A10

WNT1	1.65989476	-0.91026213	0.27838063	-3.26984723	0.00107606	0.00179638	Down	WNT1
RGS17	48.9671273	0.57582941	0.17610539	3.26980007	0.00107624	0.00179651	Up	RGS17
TAS2R50	2.22570756	2.03631873	0.62304781	3.26831856	0.00108189	0.0018051	Up	TAS2R50
GTF2A1L	0.64747192	-1.50952063	0.46200257	-3.26734247	0.00108562	0.00181116	Down	GTF2A1L
SLC16A7	145.554474	-0.81790949	0.2507509	-3.26184075	0.00110691	0.0018453	Down	SLC16A7
TRIM63	3.46940504	-1.03549612	0.3174783	-3.26162801	0.00110774	0.00184634	Down	TRIM63
SLC14A1	233.098816	1.1156811	0.34269077	3.25564968	0.00113133	0.00188475	Up	SLC14A1
TGM3	75.0777423	0.77444608	0.23790864	3.25522462	0.00113303	0.00188707	Up	TGM3
GAL3ST2	314.207848	-0.63527488	0.19542386	-3.25075388	0.00115099	0.00191575	Down	GAL3ST2
KRT81	19.3995466	0.89715183	0.27608262	3.24957743	0.00115577	0.00192333	Up	KRT81
WNT16	8.31072775	-0.93486612	0.28774541	-3.24893499	0.00115838	0.00192732	Down	WNT16
ZNF662	98.8085698	-0.72066908	0.22206384	-3.24532378	0.00117317	0.0019512	Down	ZNF662
RXFP1	10.932048	-0.65743949	0.202617	-3.24474004	0.00117558	0.00195484	Down	RXFP1
SCGB1D2	0.37159832	-2.1593531	0.66610335	-3.24176885	0.0011879	0.00197442	Down	SCGB1D2
C22orf15	4.94781164	-0.6471501	0.19975338	-3.23974542	0.00119636	0.0019878	Down	C22orf15
ABLIM3	310.48674	-0.6035315	0.18629029	-3.23973667	0.0011964	0.0019878	Down	ABLIM3
IGFBP5	10182.3555	-0.58118451	0.17963012	-3.23545138	0.00121451	0.00201751	Down	IGFBP5
TPSD1	52.3695556	-1.11054307	0.34325698	-3.23531092	0.0012151	0.00201831	Down	TPSD1
CLEC3A	3.01096162	1.91632462	0.59260354	3.23373804	0.00122181	0.00202927	Up	CLEC3A
SLC16A14	197.825731	-0.51363122	0.15892842	-3.2318399	0.00122996	0.00204261	Down	SLC16A14
ALLC	1.15336488	1.46355551	0.45291236	3.231432	0.00123172	0.00204514	Up	ALLC
DYDC2	6.84007513	1.49323758	0.46223754	3.23045498	0.00123593	0.00205176	Up	DYDC2
NME9	12.1092574	0.69083789	0.21385833	3.23035298	0.00123637	0.00205231	Up	NME9
ANK1	57.9534631	0.60773722	0.18821382	3.22897238	0.00124236	0.00206205	Up	ANK1
C4B	152.215498	-0.64283076	0.19911776	-3.22839499	0.00124487	0.00206583	Down	C4B
MYO1H	11.9730808	0.70498674	0.21839267	3.22806962	0.00124629	0.00206799	Up	MYO1H
CSF3R	339.20815	0.68754824	0.21305407	3.22710678	0.00125049	0.00207477	Up	CSF3R
MORC1	0.88967037	-1.6342557	0.50660623	-3.22588948	0.00125582	0.00208323	Down	MORC1
ZNF177	0.70912082	1.19335009	0.37022455	3.22331433	0.00126716	0.00210126	Up	ZNF177
C4orf50	2.0745973	-0.89499872	0.27775147	-3.2223006	0.00127166	0.00210852	Down	C4orf50
NBPF6	6.45713437	1.24050238	0.38510634	3.22119437	0.00127658	0.00211589	Up	NBPF6
SOX11	37.2336189	-0.71502644	0.22210776	-3.21927724	0.00128514	0.00212989	Down	SOX11
ACSM4	3.79888606	-0.81622134	0.25357111	-3.21890517	0.00128681	0.00213246	Down	ACSM4
AEBP1	7595.18863	0.61993953	0.19261436	3.21855303	0.00128839	0.00213488	Up	AEBP1
PKHD1	32.1822208	0.97623757	0.30334541	3.21823746	0.00128981	0.00213703	Up	PKHD1
SLC6A18	4.6434552	-0.91930191	0.28592681	-3.21516511	0.0013037	0.00215883	Down	SLC6A18
DDX53	1.66210843	3.56411891	1.10888166	3.21415625	0.00130828	0.00216603	Up	DDX53
INHBC	1.75771779	0.77933721	0.24263331	3.21199595	0.00131816	0.00218218	Up	INHBC
LYG2	3.85115519	0.861094	0.26822533	3.21033818	0.00132579	0.0021942	Up	LYG2
TEKT2	3.93046067	-0.79419624	0.24757601	-3.20788856	0.00133713	0.00221216	Down	TEKT2
SPATC1L	319.430633	0.67769001	0.21125918	3.20786069	0.00133726	0.00221216	Up	SPATC1L
SLC22A14	3.37753712	0.74224384	0.23148168	3.20649066	0.00134365	0.0022221	Up	SLC22A14
SLC6A4	152.122365	0.9425162	0.29395279	3.2063523	0.00134429	0.00222297	Up	SLC6A4
ZNF625	2.39463129	-0.79355152	0.24752791	-3.20590732	0.00134637	0.0022262	Down	ZNF625
HES5	15.603103	-0.8120129	0.2536933	-3.20076606	0.00137063	0.00226546	Down	HES5
KIAA0319	116.853802	0.7234688	0.22603476	3.20069702	0.00137096	0.0022658	Up	KIAA0319
LKAAEAR1	1.46198662	1.46249016	0.45693998	3.20061763	0.00137133	0.00226621	Up	LKAAEAR1
TAS2R46	1.49580352	1.83459119	0.57334161	3.19982214	0.00137512	0.00227206	Up	TAS2R46
PGA5	0.40981865	-1.39190039	0.43508538	-3.19914308	0.00137837	0.00227704	Down	PGA5
DACT1	414.606605	0.53876173	0.16844874	3.19837196	0.00138206	0.00228288	Up	DACT1
RPS10-NUDT	1.30835261	0.98796171	0.30920998	3.19511588	0.00139775	0.00230751	Up	RPS10-NUDT3
XG	26.3091977	0.88442911	0.27688787	3.19417784	0.0014023	0.00231453	Up	XG
PCDHGA10	213.552436	0.79610426	0.24923748	3.19415951	0.00140239	0.00231453	Up	PCDHGA10
CEACAM16	3.54962233	-0.81296244	0.25453044	-3.1939694	0.00140331	0.00231584	Down	CEACAM16
MASP2	33.4966324	-0.53073724	0.16617475	-3.19385005	0.00140389	0.00231658	Down	MASP2
NKAIN4	16.9343383	0.7542258	0.23628344	3.19203828	0.00141273	0.00233095	Up	NKAIN4
CLCNKA	4.18639377	-0.83052188	0.26042134	-3.18914673	0.00142693	0.0023529	Down	CLCNKA
CCDC181	10.552281	-0.64148207	0.20123981	-3.18765002	0.00143434	0.0023642	Down	CCDC181

TYROBP	1024.61621	-0.5434176	0.17047852	-3.18760156	0.00143458	0.00236438	Down	TYROBP
HSPA1A	1315.55358	-0.59948171	0.18821738	-3.1850497	0.00144729	0.00238445	Down	HSPA1A
GNB3	37.9069052	0.50266166	0.15782454	3.18493986	0.00144784	0.00238514	Up	GNB3
ERN2	2880.67978	-0.50677058	0.15921333	-3.18296573	0.00145775	0.00240035	Down	ERN2
EYS	38.5287515	0.62259925	0.19566124	3.18202648	0.00146248	0.0024077	Up	EYS
TMEM88B	0.86775861	1.47957576	0.46588965	3.17580729	0.0014942	0.00245788	Up	TMEM88B
PI3	3281.62654	0.85879004	0.27063583	3.17323111	0.00150752	0.00247911	Up	PI3
ZP2	15.915805	0.9938662	0.3133459	3.17178625	0.00151504	0.00249102	Up	ZP2
ZNF492	7.99054429	-1.13151521	0.3567502	-3.1717297	0.00151534	0.00249127	Down	ZNF492
ASCL4	0.49862135	-1.3134622	0.41417843	-3.17124723	0.00151786	0.00249518	Down	ASCL4
OR2AG2	1.84686538	0.97812586	0.30850437	3.17054139	0.00152155	0.00250079	Up	OR2AG2
UTF1	1.06165336	1.83925567	0.58081692	3.16667025	0.00154195	0.00253315	Up	UTF1
SP7	1.84261525	1.3937122	0.4401734	3.16627991	0.00154402	0.00253609	Up	SP7
CD68	36.789781	-0.56852708	0.17961873	-3.16518813	0.00154983	0.00254539	Down	CD68
SRRM3	71.4522818	0.61305805	0.19369692	3.16503766	0.00155063	0.00254624	Up	SRRM3
GPD1	456.497358	0.73378414	0.23188847	3.16438389	0.00155412	0.0025515	Up	GPD1
PCDH15	2.39700172	-1.37873464	0.43617665	-3.16095475	0.00157253	0.00258125	Down	PCDH15
LRRTM1	29.2944427	-1.15624683	0.36623831	-3.15708874	0.00159353	0.00261452	Down	LRRTM1
RTP3	0.9589258	1.45702577	0.46188538	3.15451804	0.00160763	0.00263669	Up	RTP3
C19orf38	46.219818	-0.5590747	0.17723816	-3.15436972	0.00160845	0.00263779	Down	C19orf38
ITIH3	25.2707143	-0.61039713	0.19352147	-3.1541571	0.00160962	0.00263923	Down	ITIH3
KATNAL2	43.0277	-0.53108693	0.16840743	-3.15358377	0.00161279	0.00264417	Down	KATNAL2
GLRA1	0.82236575	1.64229714	0.52081443	3.15332497	0.00161422	0.00264603	Up	GLRA1
TECTB	2.66982021	1.00131505	0.3176937	3.15182534	0.00162253	0.00265917	Up	TECTB
MT1B	0.44115608	-1.87432835	0.59491918	-3.15055964	0.00162958	0.0026695	Down	MT1B
FSD2	3.08250601	1.08221036	0.34352963	3.1502679	0.00163121	0.00267143	Up	FSD2
TLR2	267.377416	0.5365566	0.1705453	3.14612365	0.0016545	0.00270858	Up	TLR2
TNNC1	98.6946982	0.78825546	0.25064343	3.1449277	0.00166128	0.00271943	Up	TNNC1
MAFB	556.346427	-0.51714968	0.16454473	-3.14291251	0.00167276	0.00273696	Down	MAFB
SLC39A2	169.823906	1.23207931	0.39222909	3.1412237	0.00168243	0.00275179	Up	SLC39A2
FPR1	240.557693	0.71595268	0.22795389	3.14077858	0.00168499	0.00275572	Up	FPR1
PROC	86.3814789	0.53700269	0.17098526	3.14063725	0.00168581	0.0027568	Up	PROC
OTX2	1.05013114	2.76470507	0.88033099	3.14052908	0.00168643	0.00275731	Up	OTX2
VWA3A	3.35244107	-0.75268877	0.23966764	-3.14055229	0.0016863	0.00275731	Down	VWA3A
APOA1	29.6484085	1.10225903	0.35103527	3.14002358	0.00168934	0.00276182	Up	APOA1
MLANA	19.2744408	0.67385085	0.21467748	3.13889862	0.00169584	0.00277168	Up	MLANA
OPCML	14.7528887	0.7254642	0.23116582	3.13828487	0.0016994	0.00277724	Up	OPCML
HUS1B	3.85091714	0.67793688	0.21620854	3.13556942	0.00171521	0.00280205	Up	HUS1B
SLC36A2	1.25864423	1.76019692	0.5614586	3.1350431	0.00171829	0.00280683	Up	SLC36A2
RPRML	4.3940424	1.45947336	0.4656649	3.13417086	0.0017234	0.00281441	Up	RPRML
VAX2	7.9041291	0.84523402	0.26988909	3.13178286	0.00173748	0.00283688	Up	VAX2
CST11	0.60133994	1.55732779	0.49753178	3.13010717	0.00174743	0.00285181	Up	CST11
CERCAM	1202.64353	0.5111672	0.16345706	3.12722622	0.00176464	0.00287885	Up	CERCAM
MEPE	0.48581896	1.77291998	0.56720202	3.12572933	0.00177365	0.00289328	Up	MEPE
CHRNB3	0.41860096	1.4847056	0.47514129	3.12476653	0.00177946	0.00290144	Up	CHRNB3
FRMPD3	43.4764168	0.88356302	0.28303523	3.12174215	0.00179784	0.00293007	Up	FRMPD3
SLC13A4	38.5946019	0.56408884	0.1810156	3.11624433	0.0018317	0.00298335	Up	SLC13A4
C3	6991.87291	-0.69019844	0.2215033	-3.1159736	0.00183339	0.00298542	Down	C3
LYZL2	0.47544867	2.05479392	0.65952147	3.11558305	0.00183582	0.00298896	Up	LYZL2
SERF1A	2.44312753	-0.87723522	0.28172664	-3.11378155	0.00184706	0.00300562	Down	SERF1A
SERPING1	3704.5097	-0.53948673	0.17328267	-3.11333346	0.00184987	0.00300992	Down	SERPING1
UTS2	24.0538801	0.89153203	0.28643289	3.11253369	0.00185489	0.00301781	Up	UTS2
IL33	1446.13578	0.71043211	0.2285368	3.10861139	0.00187969	0.00305704	Up	IL33
TMSB15A	13.4459019	0.51556862	0.16592256	3.10728453	0.00188815	0.00306996	Up	TMSB15A
CELF5	55.737385	0.72909169	0.23467469	3.10681862	0.00189112	0.00307452	Up	CELF5
SIGLEC14	61.0627046	-0.68343153	0.2200274	-3.10612005	0.0018956	0.00308151	Down	SIGLEC14
APOBEC2	5.1800304	-0.76511378	0.24649447	-3.10397947	0.00190937	0.00310305	Down	APOBEC2
LILRA2	38.329883	-0.60228174	0.1942078	-3.10122325	0.00192723	0.00313122	Down	LILRA2

ZBTB9	1.16698005	0.81744792	0.26392082	3.09732267	0.00195277	0.00317099	Up	ZBTB9
SRRM4	2.31974163	-1.18645535	0.3832827	-3.09550984	0.00196475	0.00318986	Down	SRRM4
DLEC1	47.2875127	-0.52919824	0.17104666	-3.09388224	0.00197556	0.00320624	Down	DLEC1
OR2D2	0.9842982	2.79164634	0.9023392	3.09378818	0.00197619	0.00320697	Up	OR2D2
ZSCAN23	7.02989539	-0.97007877	0.3137698	-3.09168944	0.00199021	0.00322884	Down	ZSCAN23
ADAT3	282.249072	0.53824556	0.17424724	3.08897608	0.00200848	0.00325729	Up	ADAT3
PLA2G2A	9007.72458	-0.96681787	0.3130163	-3.08871411	0.00201025	0.00325958	Down	PLA2G2A
FBXO17	122.055035	-0.65816088	0.21310561	-3.08842589	0.0020122	0.00326244	Down	FBXO17
ERICH6	2.21977142	0.70852478	0.22952617	3.086902	0.00202254	0.00327832	Up	ERICH6
TENM3	153.215259	-0.78428927	0.25411212	-3.08639066	0.00202602	0.00328366	Down	TENM3
DDIT4L	34.4040697	-0.87453324	0.28356367	-3.08408067	0.00204182	0.00330867	Down	DDIT4L
SMIM24	1127.77762	0.58842741	0.19089356	3.08248955	0.00205277	0.0033255	Up	SMIM24
GOLGA8M	0.74944583	-0.93628923	0.30411967	-3.0786868	0.00207915	0.00336732	Down	GOLGA8M
KCNIP2	45.8224796	-0.51795629	0.16830777	-3.07743546	0.0020879	0.00338057	Down	KCNIP2
LINGO2	2.09674205	-0.92401402	0.30025931	-3.07738673	0.00208824	0.00338082	Down	LINGO2
FBXL2	119.369179	0.51359657	0.16698414	3.07572065	0.00209994	0.00339946	Up	FBXL2
RLN1	4.8289749	0.66813138	0.21745346	3.07252586	0.00212255	0.00343419	Up	RLN1
MROH7	39.6501569	-0.63765421	0.20765905	-3.07067862	0.00213573	0.00345363	Down	MROH7
DCAF8L2	0.71373992	2.20336365	0.71767718	3.07013195	0.00213964	0.00345901	Up	DCAF8L2
GLYATL2	6.01039123	0.73015856	0.23799004	3.06802147	0.00215481	0.00348259	Up	GLYATL2
CBFA2T3	281.417551	-0.73410133	0.23933481	-3.06725682	0.00216033	0.0034912	Down	CBFA2T3
PRKCQ	143.230693	0.5133283	0.16739501	3.0665687	0.00216531	0.00349861	Up	PRKCQ
C8G	96.1931377	-0.78919352	0.25752113	-3.06457776	0.00217978	0.00352007	Down	C8G
TFEC	118.930443	-0.64285219	0.20981279	-3.06393233	0.00218448	0.00352735	Down	TFEC
RORA	279.087902	-0.55902555	0.18270521	-3.05971316	0.00221549	0.00357645	Down	RORA
HOMER2	81.7472791	-0.80316565	0.26287261	-3.05534176	0.00224804	0.00362801	Down	HOMER2
C9orf131	3.35827873	1.25813148	0.41182713	3.05499901	0.00225061	0.00363183	Up	C9orf131
VCX	0.60862525	1.93166051	0.63258057	3.05361973	0.00226098	0.00364758	Up	VCX
PVRIG	4.85537332	0.73703736	0.24167029	3.04976407	0.00229021	0.0036924	Up	PVRIG
CRYBA4	6.16662864	0.71003736	0.23286889	3.04908632	0.00229539	0.00370013	Up	CRYBA4
PPP1R14C	750.311685	-0.56423731	0.18505156	-3.04908161	0.00229542	0.00370013	Down	PPP1R14C
GRID2	1.51893689	-1.55652495	0.51067856	-3.04795437	0.00230405	0.0037137	Down	GRID2
RTL1	1.17299496	2.08051418	0.68283392	3.0468817	0.00231229	0.00372563	Up	RTL1
ACADL	3.91168861	-1.3655911	0.44820845	-3.0467768	0.00231309	0.00372659	Down	ACADL
SLC7A2	205.086569	-0.74906752	0.24589137	-3.0463352	0.00231649	0.00373106	Down	SLC7A2
CLPSL1	0.8624652	2.48950291	0.81760383	3.04487678	0.00232776	0.00374852	Up	CLPSL1
TNFSF18	13.1789503	0.67997514	0.22352213	3.04209314	0.00234939	0.00378234	Up	TNFSF18
HDX	57.4979012	-0.5223932	0.17176966	-3.04124253	0.00235604	0.0037927	Down	HDX
C4BPA	268.667724	0.78035908	0.25659572	3.04120066	0.00235637	0.00379289	Up	C4BPA
DACT2	89.7765213	-0.85830353	0.28239944	-3.03932442	0.00237109	0.00381521	Down	DACT2
PLXNB3	118.972035	-0.73875448	0.24309614	-3.0389396	0.00237412	0.0038194	Down	PLXNB3
ATP1A3	151.161096	-0.73437325	0.24189048	-3.03597414	0.0023976	0.00385473	Down	ATP1A3
SUSD2	212.545746	-0.58528826	0.19283793	-3.03513036	0.00240432	0.00386428	Down	SUSD2
IL6	167.777593	0.801164	0.26413269	3.03318763	0.00241985	0.00388771	Up	IL6
RIMS1	9.80767587	-0.99607219	0.32841308	-3.03298573	0.00242147	0.00388961	Down	RIMS1
CDHR4	2.60313448	1.02675975	0.33855058	3.03281048	0.00242288	0.00389152	Up	CDHR4
TFAP2C	140.113604	0.67395727	0.2222725	3.03212173	0.00242841	0.00389971	Up	TFAP2C
CACNG5	0.31759508	-1.81857495	0.60028466	-3.02952092	0.00244942	0.00392991	Down	CACNG5
IGLL1	6.39655328	-1.0159023	0.33544958	-3.02847986	0.00245787	0.00394276	Down	IGLL1
CAPSL	1.02877769	1.53059303	0.50570901	3.02662799	0.00247298	0.00396486	Up	CAPSL
CD86	232.635236	-0.51076985	0.16883362	-3.02528521	0.00248399	0.00398143	Down	CD86
FOXD4L5	0.50956304	1.45720427	0.48184699	3.02420543	0.00249287	0.00399459	Up	FOXD4L5
PDCL2	0.37948424	1.59193522	0.52646779	3.02380366	0.00249618	0.00399954	Up	PDCL2
HOXB5	717.684413	0.502833	0.16633406	3.02303085	0.00250257	0.00400883	Up	HOXB5
GP6	10.0770567	0.68219378	0.22585393	3.02050878	0.0025235	0.00404005	Up	GP6
IFNK	2.36650796	3.10977245	1.03006202	3.01901478	0.00253598	0.00405966	Up	IFNK
ART4	11.4558889	-0.9659195	0.31998221	-3.01866627	0.0025389	0.00406397	Down	ART4
CCR6	9.17306268	-0.53740741	0.1780705	-3.01794742	0.00254493	0.00407326	Down	CCR6

NCCRP1	21.818911	-0.89109065	0.29530605	-3.01751573	0.00254856	0.0040787	Down	NCCRP1
C4A	142.161235	-0.60541988	0.20084203	-3.01440828	0.00257481	0.0041196	Down	C4A
KCNK12	12.3205354	-0.65513126	0.21741705	-3.01324702	0.00258468	0.00413503	Down	KCNK12
PRSS21	102.741493	1.2272143	0.40741613	3.01218878	0.00259371	0.00414799	Up	PRSS21
TAS2R43	2.0025358	1.63960022	0.54471837	3.0099962	0.00261251	0.00417618	Up	TAS2R43
TRIML2	0.79633663	1.827211	0.60706676	3.00990126	0.00261333	0.00417711	Up	TRIML2
MFAP3L	214.760719	0.52230596	0.17357618	3.00908784	0.00262033	0.00418756	Up	MFAP3L
CYP4A22	1.5589242	1.97024914	0.65501476	3.00794616	0.0026302	0.00420257	Up	CYP4A22
METRN	727.300129	0.56047762	0.18639091	3.0070008	0.00263839	0.00421415	Up	METRN
TRIM67	8.41070194	-0.533276	0.17740371	-3.00600256	0.00264707	0.00422763	Down	TRIM67
OR1Q1	0.88320507	1.62301095	0.54045894	3.0030236	0.00267312	0.00426771	Up	OR1Q1
KRTAP5-10	2.93102986	0.77927862	0.25954853	3.00243898	0.00267826	0.00427477	Up	KRTAP5-10
OPN5	0.55244976	1.79383414	0.59887831	2.99532329	0.00274154	0.00437108	Up	OPN5
OR56B4	1.44671582	-0.59737926	0.19964019	-2.9922796	0.00276903	0.00441175	Down	OR56B4
HAL	22.1075687	0.56657128	0.18966155	2.98727537	0.00281476	0.00448182	Up	HAL
FLG2	1.35932237	2.430284	0.81356789	2.98719264	0.00281552	0.00448263	Up	FLG2
OR4C6	0.76213252	1.68306006	0.56378787	2.98527185	0.00283326	0.00451007	Up	OR4C6
TREML2	53.951848	0.79125625	0.26511983	2.98452312	0.00284021	0.00452072	Up	TREML2
GPR25	13.9760728	0.82673744	0.27739892	2.98031955	0.00287948	0.0045816	Up	GPR25
ZNF880	138.184479	-0.57920497	0.19445376	-2.97862567	0.00289544	0.00460576	Down	ZNF880
AGMO	77.9129702	-0.81941321	0.27522752	-2.97722126	0.00290874	0.00462568	Down	AGMO
PRB4	1.34760802	2.6971458	0.90602057	2.97691453	0.00291165	0.00462948	Up	PRB4
KLK13	14.9085282	-0.96256546	0.32409024	-2.97005378	0.00297748	0.00473035	Down	KLK13
PLSCR2	10.2541233	0.7621757	0.25668919	2.96925522	0.00298523	0.00474224	Up	PLSCR2
CCDC8	112.801003	-0.52571676	0.17706159	-2.96911805	0.00298656	0.00474394	Down	CCDC8
REC114	1.30890752	1.32986258	0.4484908	2.96519482	0.00302491	0.00480155	Up	REC114
HOXD13	176.173087	-0.95691323	0.32319854	-2.96075971	0.00306881	0.00486679	Down	HOXD13
TRPC5	1.44708452	1.86405404	0.62983204	2.959605	0.00308034	0.0048842	Up	TRPC5
SLC6A15	11.4322193	-1.31701912	0.44599588	-2.95298494	0.00314717	0.00498708	Down	SLC6A15
SCGB3A2	1.66676082	1.46637743	0.49671942	2.95212421	0.00315596	0.00499967	Up	SCGB3A2
SLC43A3	542.955128	0.53518762	0.18129341	2.95205228	0.00315669	0.00500039	Up	SLC43A3
PAGE2	0.49299174	1.69323267	0.57359183	2.95198187	0.00315741	0.00500108	Up	PAGE2
CLEC19A	1.07668177	-0.94327943	0.31987569	-2.94889379	0.00318914	0.00504998	Down	CLEC19A
LIPF	1.12379124	2.47412807	0.83971668	2.94638435	0.00321513	0.00508888	Up	LIPF
OR2T8	0.49378068	-1.25386967	0.42562971	-2.94591667	0.00321999	0.00509613	Down	OR2T8
WNT9B	2.01177873	0.82872947	0.28134301	2.94561953	0.00322309	0.00510058	Up	WNT9B
FOXD4L1	3.00153293	0.70067501	0.23795799	2.94453238	0.00323443	0.00511808	Up	FOXD4L1
GRM1	4.79703366	0.98386379	0.3342133	2.94382001	0.00324188	0.00512942	Up	GRM1
DUOX2	5713.06766	0.84461297	0.28712645	2.94160624	0.00326515	0.00516439	Up	DUOX2
CHST6	109.240977	-0.61513632	0.20913107	-2.94139142	0.00326741	0.00516752	Down	CHST6
PTTG2	1.09832387	-0.75549296	0.25695628	-2.94016143	0.00328041	0.00518716	Down	PTTG2
FAM72B	27.8423958	0.6699821	0.22788243	2.94003408	0.00328176	0.00518883	Up	FAM72B
PACRG	13.6907904	-0.63756648	0.21690686	-2.93935606	0.00328895	0.00519927	Down	PACRG
SULT4A1	28.082728	-1.12834457	0.38398238	-2.93853219	0.0032977	0.00521265	Down	SULT4A1
FAM184B	23.4884451	-0.61950029	0.21091293	-2.93723244	0.00331156	0.00523223	Down	FAM184B
FAM50B	164.842019	-0.60586067	0.20639947	-2.93537897	0.00333141	0.00526126	Down	FAM50B
C12orf54	1.95581399	-0.72027198	0.24541751	-2.93488426	0.00333672	0.00526919	Down	C12orf54
TSPAN16	1.36398463	0.94541165	0.32269587	2.92972961	0.00339257	0.0053536	Up	TSPAN16
DPPA4	3.69953895	1.1388649	0.38906898	2.9271542	0.00342079	0.00539479	Up	DPPA4
GAL3ST3	0.63466621	-1.42049679	0.48567858	-2.92476724	0.00344714	0.00543394	Down	GAL3ST3
ZNF98	0.9584795	-1.03463953	0.35401382	-2.92259642	0.00347126	0.00547148	Down	ZNF98
SLC5A4	6.59111645	0.68015775	0.23319682	2.91666823	0.00353792	0.00557114	Up	SLC5A4
NOL4	13.0227896	-1.05272998	0.36184798	-2.90931564	0.00362221	0.00569784	Down	NOL4
CCL1	0.85999805	1.21866053	0.41889219	2.90924627	0.00362301	0.0056986	Up	CCL1
BPIFB4	0.81873328	2.25164349	0.77443611	2.9074619	0.00364375	0.0057302	Up	BPIFB4
TRAPPC3L	1.61254083	-0.68897333	0.23703511	-2.9066299	0.00365345	0.00574496	Down	TRAPPC3L
ZNF660	37.8070043	-0.57765169	0.19880292	-2.90564987	0.00366491	0.00576247	Down	ZNF660
PLSCR5	0.46971634	-2.37675817	0.81817719	-2.90494308	0.0036732	0.00577448	Down	PLSCR5

VSTM2B	5.50538144	1.63405946	0.56352904	2.89968992	0.00373532	0.00586749	Up	VSTM2B
CATSPERB	63.4144742	0.5083407	0.17531823	2.8995314	0.00373721	0.00586994	Up	CATSPERB
CYP3A43	1.79559411	1.13384512	0.39111163	2.89903197	0.00374317	0.00587826	Up	CYP3A43
OTOGL	12.0631583	-0.72140013	0.24897333	-2.89749959	0.0037615	0.00590602	Down	OTOGL
NHLH2	5.83466725	1.60047857	0.55293278	2.89452647	0.00379731	0.00596014	Up	NHLH2
DPP4	1470.91691	-0.58424838	0.2019771	-2.8926466	0.00382011	0.00599329	Down	DPP4
TNFSF12-TN	0.90640675	-0.75217345	0.2600506	-2.89241188	0.00382296	0.00599671	Down	TNFSF12-TNFSF13
CT55	0.61556475	1.73074364	0.59843225	2.89212965	0.0038264	0.00600158	Up	CT55
GPR37	63.7022054	0.67203455	0.23244477	2.89115792	0.00383825	0.00601911	Up	GPR37
CRTAM	20.8919916	-0.65406965	0.22624536	-2.89097489	0.00384049	0.00602209	Down	CRTAM
MCOLN3	89.0925801	0.71011743	0.24577305	2.88932179	0.00386074	0.00605331	Up	MCOLN3
EMX1	39.7509389	1.05090204	0.36375684	2.88902346	0.0038644	0.00605852	Up	EMX1
CPO	3.99633175	-0.63798824	0.22089472	-2.88820053	0.00387453	0.00607226	Down	CPO
CTAGE4	7.20157951	0.57477726	0.19916716	2.88590385	0.00390291	0.00611567	Up	CTAGE4
TBC1D26	0.78586288	1.55285229	0.53818914	2.88532815	0.00391006	0.00612633	Up	TBC1D26
SHANK1	15.1464857	0.59663733	0.20701684	2.88207156	0.0039507	0.00618621	Up	SHANK1
OTOP1	0.61948015	2.25496005	0.78247292	2.88183781	0.00395363	0.00619025	Up	OTOP1
TRH	0.89530911	1.1137191	0.3864653	2.88180879	0.003954	0.00619028	Up	TRH
PCDHGB3	12.3520566	-0.6395458	0.22207878	-2.87981498	0.00397909	0.00622738	Down	PCDHGB3
LHFPL4	24.593858	-1.20421205	0.41880556	-2.87534877	0.00403581	0.00631394	Down	LHFPL4
CCDC38	2.83484904	0.83269522	0.28971168	2.87422036	0.00405026	0.00633544	Up	CCDC38
MYH3	46.7654543	-0.5081355	0.17684548	-2.87333046	0.00406169	0.00635164	Down	MYH3
HLA-DRB1	8343.91196	-0.51491781	0.17951867	-2.86832455	0.00412652	0.00645019	Down	HLA-DRB1
LRRC38	2.11411997	0.85956604	0.29974776	2.86763129	0.00413557	0.00646378	Up	LRRC38
OR10AC1	0.81022557	1.50719594	0.52560385	2.8675512	0.00413662	0.00646485	Up	OR10AC1
HOXA6	74.3088268	-0.56932234	0.19866164	-2.86578901	0.00415971	0.0064998	Down	HOXA6
GLIPR1L1	9.04782686	0.71060122	0.24847902	2.85980365	0.00423903	0.00661795	Up	GLIPR1L1
ABHD12B	203.924467	1.03080491	0.36094932	2.8558162	0.00429264	0.00669988	Up	ABHD12B
WFDC11	0.39044896	1.50749344	0.52805796	2.85478784	0.00430656	0.00671984	Up	WFDC11
TSHR	8.78646225	-0.76940072	0.26965327	-2.85329645	0.00432682	0.00674969	Down	TSHR
GABBR2	4.97194946	0.69436652	0.24344885	2.85220709	0.00434168	0.00677109	Up	GABBR2
ACR	2.18555404	-0.60512626	0.21217973	-2.85195135	0.00434518	0.00677595	Down	ACR
DOC2A	97.3486932	0.69011227	0.24225582	2.84869226	0.00438993	0.00684276	Up	DOC2A
RNF148	3.80100149	0.6819852	0.23993022	2.84243141	0.00447709	0.00697252	Up	RNF148
LRRD1	1.33013053	0.94482842	0.3326294	2.8404838	0.00450452	0.0070134	Up	LRRD1
ARC	33.9540468	-0.75125135	0.26451785	-2.8400781	0.00451025	0.00702171	Down	ARC
RNASE10	1.14738367	0.84612223	0.29800312	2.83930663	0.00452117	0.0070381	Up	RNASE10
TAS2R42	0.46027044	1.29871029	0.45830633	2.83371669	0.00460101	0.00715552	Up	TAS2R42
MARK1	136.59303	-0.59260756	0.20921142	-2.83257744	0.00461744	0.00717982	Down	MARK1
PKIA	132.165742	-0.66530482	0.23492632	-2.83197231	0.00462619	0.0071928	Down	PKIA
ACTC1	54.3592121	-0.75104934	0.26527664	-2.8311929	0.00463747	0.00720909	Down	ACTC1
OPTC	1.19218766	-1.08297738	0.38333024	-2.82518116	0.00472539	0.00734256	Down	OPTC
IFNL3	0.4389098	1.80307119	0.63824947	2.82502574	0.00472768	0.00734548	Up	IFNL3
SMIM23	0.65918373	1.23609454	0.43777882	2.82355949	0.00474936	0.00737852	Up	SMIM23
RFX6	19.1315485	-1.16765568	0.41385994	-2.82137887	0.00478177	0.00742499	Down	RFX6
KRBOX1	8.86427879	-0.94217434	0.3341435	-2.81966984	0.00480731	0.00746335	Down	KRBOX1
ENPP3	638.162727	-0.77599552	0.27522744	-2.81947007	0.0048103	0.00746735	Down	ENPP3
PRDM8	118.337438	-0.61103907	0.21693321	-2.81671518	0.00485175	0.00752842	Down	PRDM8
FATE1	4.79938834	0.51084383	0.18141784	2.81584124	0.00486497	0.00754827	Up	FATE1
SSTR4	0.26819352	-1.55161386	0.55135853	-2.81416497	0.00489041	0.00758577	Down	SSTR4
C2CD4C	33.7743159	0.6361953	0.22614175	2.81325896	0.00490421	0.00760652	Up	C2CD4C
SLC9C1	17.1863796	0.6587587	0.23425234	2.81217558	0.00492076	0.00763152	Up	SLC9C1
ISG15	1822.56358	0.52306579	0.18620129	2.80914155	0.00496738	0.00770114	Up	ISG15
RNF133	2.21858923	0.83227686	0.29628492	2.80904224	0.00496891	0.00770285	Up	RNF133
ATOH8	187.078482	-0.58745761	0.20949746	-2.80412756	0.00504529	0.00781718	Down	ATOH8
HHLA1	0.58897516	1.64117859	0.58541685	2.80343586	0.00505613	0.00783193	Up	HHLA1
MKRN3	20.8688278	1.29743441	0.4628904	2.80289765	0.00506457	0.00784433	Up	MKRN3
OR6T1	0.66669676	-1.21546511	0.43390201	-2.80124334	0.00509061	0.00788286	Down	OR6T1

LIPJ	1.42708996	-0.80943754	0.28895759	-2.80123303	0.00509077	0.00788286	Down	LIPJ
MS4A3	1.0091775	-1.00138207	0.35802841	-2.79693464	0.005159	0.00798504	Down	MS4A3
PDGFRL	82.3283391	-0.61633772	0.22074398	-2.79209301	0.00523683	0.00809988	Down	PDGFRL
PABPC4L	17.442596	-0.52079998	0.18662446	-2.79063093	0.00526054	0.00813445	Down	PABPC4L
SLC34A2	15.4480791	1.01205274	0.36272393	2.79014603	0.00526843	0.00814593	Up	SLC34A2
FPGT-TNNI3	8.03672117	-0.7331443	0.26281991	-2.78953109	0.00527844	0.0081593	Down	FPGT-TNNI3K
HEMGN	1.69161963	-1.36727018	0.49080526	-2.78576921	0.00534009	0.00824958	Down	HEMGN
VWA5B2	35.0090037	-0.69861392	0.25077869	-2.78577866	0.00533993	0.00824958	Down	VWA5B2
SLC29A4	192.460569	-0.5697862	0.20460336	-2.78483297	0.00535553	0.00827273	Down	SLC29A4
KLRC3	1.44375181	-0.81476962	0.29270063	-2.78362785	0.00537547	0.00830208	Down	KLRC3
TSPYL6	2.25962567	1.90089351	0.68335094	2.78172372	0.0054071	0.00834661	Up	TSPYL6
RNASE2	17.103818	0.55131593	0.1982826	2.78045536	0.00542827	0.00837711	Up	RNASE2
RGS7BP	21.8959537	-0.60036591	0.2160057	-2.77939847	0.00544597	0.00840297	Down	RGS7BP
DKKL1	4.1690323	-0.54024329	0.19467359	-2.77512362	0.00551807	0.00850908	Down	DKKL1
FFAR2	81.3582718	0.66663098	0.24024591	2.77478593	0.00552381	0.00851718	Up	FFAR2
FNDC7	0.88421408	1.08890227	0.39247035	2.77448286	0.00552895	0.00852438	Up	FNDC7
LRTM1	0.54938542	-1.13560372	0.40982325	-2.77095975	0.00558913	0.00861344	Down	LRTM1
CCDC160	2.21609237	-1.12917401	0.40765478	-2.76992705	0.00560688	0.00864005	Down	CCDC160
OR10Q1	1.24223927	1.17391497	0.42384831	2.76965823	0.00561151	0.00864644	Up	OR10Q1
MOG	0.64848015	-1.33861467	0.48341073	-2.76910414	0.00562107	0.00865892	Down	MOG
PRSS35	13.7141115	0.58821762	0.21262829	2.76641287	0.00566767	0.00872845	Up	PRSS35
C4orf51	0.64035416	1.37061477	0.49586353	2.76409674	0.00570806	0.00878686	Up	C4orf51
PIFO	66.6335998	-0.65633278	0.23782796	-2.75969558	0.00578552	0.0089015	Down	PIFO
CLEC2A	0.71568854	2.63707158	0.95721578	2.75493952	0.0058703	0.0090257	Up	CLEC2A
SKOR2	0.94533908	1.76400595	0.64040263	2.75452641	0.00587771	0.00903564	Up	SKOR2
SYT1	188.961021	-0.62342621	0.22639115	-2.75375703	0.00589155	0.00905448	Down	SYT1
PKLR	117.549094	0.88613143	0.32182279	2.7534763	0.0058966	0.00906147	Up	PKLR
C8orf89	2.40898106	0.75694697	0.27501016	2.75243272	0.00591543	0.00908883	Up	C8orf89
CHRM1	51.2049186	-0.70022565	0.25471358	-2.7490708	0.00597645	0.00917547	Down	CHRM1
STK33	60.0698517	-0.77313993	0.28126938	-2.7487526	0.00598225	0.0091828	Down	STK33
CNTNAP2	546.533166	0.63405103	0.230776	2.7474739	0.00600563	0.00921631	Up	CNTNAP2
ZP1	4.17831896	-0.55853903	0.20357166	-2.74369742	0.00607515	0.00931739	Down	ZP1
LY6K	3.86820643	-0.79595959	0.29049252	-2.74003466	0.00614327	0.00941863	Down	LY6K
FCGR3B	123.529663	0.77086591	0.28157532	2.73768995	0.00618724	0.00948115	Up	FCGR3B
ZNF560	0.94024112	1.19365499	0.43673873	2.73310998	0.00627394	0.0096074	Up	ZNF560
U2AF1	31.0556029	0.55538077	0.20324949	2.73250755	0.00628542	0.00962417	Up	U2AF1
TTYH1	48.4406143	-0.59623116	0.21826591	-2.73167337	0.00630136	0.00964691	Down	TTYH1
TRPV5	1.27850594	1.14647507	0.4198459	2.73070445	0.00631991	0.009672	Up	TRPV5
PGK2	0.3937428	1.23376678	0.45286881	2.72433592	0.00644309	0.00985122	Up	PGK2
CRCT1	0.75430917	2.18971769	0.80382632	2.72411793	0.00644735	0.00985688	Up	CRCT1
SPINT4	0.6753152	2.40177911	0.88225069	2.72233182	0.0064823	0.00990862	Up	SPINT4
ISLR	2170.00774	0.50346977	0.18494356	2.72228867	0.00648315	0.00990907	Up	ISLR
RIMBP3	4.32833268	-0.57749043	0.2122007	-2.72143515	0.00649991	0.00993299	Down	RIMBP3
NTSR2	0.67894401	1.58900807	0.58408312	2.72051703	0.00651799	0.00995892	Up	NTSR2
ZNF43	241.560531	-0.60736947	0.2232851	-2.72015231	0.00652519	0.00996905	Down	ZNF43
GABRB3	77.0451663	-0.95478291	0.35168914	-2.71484905	0.0066306	0.01012341	Down	GABRB3
PCDHB16	94.3505064	0.67241841	0.24772988	2.71432104	0.00664118	0.01013758	Up	PCDHB16
TMPRSS11A	0.81341583	2.33219474	0.85968119	2.71286005	0.00667053	0.01018064	Up	TMPRSS11A
SLC22A7	1.39869374	1.09428935	0.40393858	2.70904888	0.00674764	0.01029481	Up	SLC22A7
RBFOX1	20.3396792	-0.97985906	0.36178538	-2.70839875	0.00676087	0.01031236	Down	RBFOX1
SNTG1	0.47401696	-1.46459939	0.54121707	-2.70612195	0.0068074	0.01038155	Down	SNTG1
FAM187B	0.31625574	-0.97538666	0.36047439	-2.70584176	0.00681315	0.01038943	Down	FAM187B
UPP2	2.50376987	0.82223759	0.30398655	2.70484858	0.00683355	0.01041876	Up	UPP2
SLC26A5	4.54040825	0.66328846	0.245325	2.70371323	0.00685694	0.01045264	Up	SLC26A5
PROK2	23.5634028	0.85224259	0.31532258	2.7027642	0.00687655	0.01048163	Up	PROK2
SMOC2	2478.10936	0.60520012	0.22403895	2.70131655	0.00690656	0.01052647	Up	SMOC2
CPE	1247.83058	-0.55182281	0.20439828	-2.69974297	0.00693931	0.01057367	Down	CPE
LGALS14	2.2866374	1.57366144	0.58297731	2.6993528	0.00694745	0.01058518	Up	LGALS14

GRIK4	11.3557814	-0.58422034	0.21651186	-2.69832947	0.00696884	0.01061505	Down	GRIK4
GOLGA8O	0.47412973	-0.84616054	0.31362295	-2.69801859	0.00697535	0.01062407	Down	GOLGA8O
RAD21L1	0.96466841	1.50115647	0.55643955	2.69778896	0.00698017	0.01063049	Up	RAD21L1
POM121L2	2.15914557	-0.70204601	0.26026206	-2.69745819	0.00698711	0.01063924	Down	POM121L2
RBPJL	1.07099275	1.01891204	0.37775444	2.69728676	0.0069907	0.01064381	Up	RBPJL
L1TD1	1023.99155	1.00324257	0.3720361	2.69662693	0.00700457	0.01066401	Up	L1TD1
NLRP4	2.63960196	-1.36425624	0.50647386	-2.69363602	0.00706773	0.0107565	Down	NLRP4
GALR3	1.54589531	0.98137372	0.36459818	2.69165828	0.00710978	0.01081864	Up	GALR3
MLNR	0.68837613	-0.83377272	0.30989097	-2.69053569	0.00713374	0.01085326	Down	MLNR
HMHB1	0.52236725	1.13585506	0.4222579	2.68995571	0.00714615	0.01087029	Up	HMHB1
DGKB	27.8029478	-0.95670106	0.35567241	-2.6898377	0.00714868	0.0108732	Down	DGKB
XPNPEP2	629.391724	-0.76665152	0.2850901	-2.68915519	0.00716331	0.01089453	Down	XPNPEP2
PCDHB6	11.3921046	-0.66007881	0.24558265	-2.68780716	0.00719229	0.01093768	Down	PCDHB6
HLA-DQB2	242.430072	-0.57633322	0.21449003	-2.686993	0.00720985	0.01096157	Down	HLA-DQB2
SHBG	6.80404495	0.54614975	0.203305	2.68635674	0.00722359	0.0109806	Up	SHBG
SPATC1	6.90863861	0.55359251	0.20612386	2.68572746	0.00723721	0.01099942	Up	SPATC1
FGF14	17.0264633	-0.78032038	0.29123677	-2.67933332	0.00737689	0.01120313	Down	FGF14
PCDHA1	2.56439469	-0.96383348	0.35996586	-2.67756913	0.00741585	0.01125943	Down	PCDHA1
WFDC2	1926.45791	-0.7375021	0.27556858	-2.67629237	0.00744417	0.01129953	Down	WFDC2
MTRNR2L9	3.40192082	-0.75956592	0.28412299	-2.67337016	0.00750933	0.01139651	Down	MTRNR2L9
GRIN2C	40.3502472	0.57842418	0.21644023	2.67244298	0.00753012	0.01142611	Up	GRIN2C
MT1A	65.0395534	-0.74423092	0.27850287	-2.67225588	0.00753432	0.01143151	Down	MT1A
THEG	1.56929936	1.10532689	0.41467294	2.66553896	0.0076865	0.01164855	Up	THEG
SYTL5	279.832706	-0.59024107	0.22199252	-2.65883313	0.00784118	0.01187187	Down	SYTL5
DLK1	5.55528642	1.78596311	0.67180612	2.65845019	0.0078501	0.01188436	Up	DLK1
NUPR1	2163.01826	-0.50611424	0.19057894	-2.65566728	0.00791517	0.01197982	Down	NUPR1
RGS6	25.5102125	-0.63777594	0.24052759	-2.65157079	0.00801183	0.01212201	Down	RGS6
CATSPERD	0.85634781	1.30414173	0.49232733	2.64893222	0.00807465	0.01221396	Up	CATSPERD
C10orf82	47.1391693	0.84945813	0.32073908	2.64843977	0.00808643	0.01223056	Up	C10orf82
PRODH2	1.09584234	1.94714583	0.73566846	2.64677085	0.00812644	0.01228604	Up	PRODH2
LECT2	0.45793101	1.76238496	0.66651545	2.64417722	0.00818898	0.0123764	Up	LECT2
MAGEB6	0.84626866	2.78874461	1.05610001	2.64060656	0.00827578	0.01250211	Up	MAGEB6
GLYAT	1.33455399	-1.43405955	0.54327373	-2.63966299	0.00829885	0.01253396	Down	GLYAT
LRRTM3	1.10887142	-1.43746084	0.54467912	-2.63909667	0.00831273	0.01255279	Down	LRRTM3
SHISA8	2.60724377	-0.78863385	0.29895872	-2.63793559	0.00834124	0.01259479	Down	SHISA8
NPAP1	0.85481941	-1.45066079	0.55014341	-2.63687753	0.0083673	0.012632	Down	NPAP1
ELAVL2	44.2716828	0.79825954	0.30294149	2.63502878	0.00841302	0.01269994	Up	ELAVL2
ADAM20	2.40393422	0.93400751	0.35456356	2.63424564	0.00843245	0.01272712	Up	ADAM20
NAV3	48.1990194	-0.6064976	0.23043161	-2.6320069	0.00848821	0.01280804	Down	NAV3
NTM	322.372455	0.54769165	0.2082681	2.62974337	0.00854493	0.01289144	Up	NTM
IFIT1B	0.92064606	1.60090165	0.60977611	2.62539255	0.00865491	0.01305405	Up	IFIT1B
GCM2	0.66833113	2.0561668	0.7836168	2.62394425	0.0086918	0.01310748	Up	GCM2
FNDC1	1254.99976	0.62041418	0.23661419	2.62204971	0.00874027	0.01317611	Up	FNDC1
CA8	350.216948	0.72262298	0.27559336	2.62206233	0.00873994	0.01317611	Up	CA8
HSF5	1.97853107	-0.70339595	0.2683955	-2.62074418	0.00877381	0.01322444	Down	HSF5
GKN2	0.51918862	-1.24649378	0.47567132	-2.62049389	0.00878025	0.0132308	Down	GKN2
LHFPL5	1.92383747	0.83697287	0.31949748	2.61965406	0.0088019	0.01325894	Up	LHFPL5
FAM167A	146.167617	0.50792497	0.19389486	2.61958962	0.00880356	0.01326033	Up	FAM167A
PXT1	2.02916608	0.69590675	0.26577443	2.61841117	0.00883403	0.0133051	Up	PXT1
SOX2	96.151493	1.1547222	0.4410764	2.61796414	0.00884561	0.01332029	Up	SOX2
GPR62	2.14535666	-0.63552252	0.24276165	-2.61788683	0.00884761	0.01332218	Down	GPR62
CCDC7	13.142425	0.57471672	0.21961271	2.61695567	0.00887179	0.01335633	Up	CCDC7
SMIM17	5.75975968	0.5892673	0.22521104	2.61651153	0.00888334	0.01337033	Up	SMIM17
MANSC4	1.77971594	0.74054096	0.28341804	2.61289281	0.00897795	0.01350817	Up	MANSC4
FABP7	3.05475862	1.21025216	0.46355073	2.61082999	0.00903228	0.01358763	Up	FABP7
BNIP3	531.543324	0.51731687	0.19827681	2.6090639	0.00907903	0.01365566	Up	BNIP3
GOLGA8Q	0.28460485	-1.03754953	0.39791486	-2.60746616	0.00912151	0.01371839	Down	GOLGA8Q
IL22	2.95442611	1.06697892	0.40920869	2.60742002	0.00912274	0.01371909	Up	IL22

GAS2L2	2.66888122	-0.83079128	0.31890095	-2.60517028	0.00918286	0.01380717	Down	GAS2L2
AGXT	46.3123181	-0.58992268	0.22660478	-2.60331089	0.00923282	0.01387411	Down	AGXT
FOXN1	15.3551375	-0.71158127	0.27345325	-2.60220444	0.00926266	0.01391309	Down	FOXN1
C19orf84	3.28388782	0.73295606	0.28196644	2.59944428	0.00933748	0.01401958	Up	C19orf84
TCL1B	0.27957562	-2.2775858	0.87641153	-2.59876293	0.00935604	0.01404507	Down	TCL1B
ISLR2	52.3234853	-0.50362905	0.19427412	-2.59236302	0.00953191	0.01429827	Down	ISLR2
CPA2	23.4643078	-0.90589112	0.34957365	-2.59141706	0.00955816	0.01433523	Down	CPA2
CXCR2	69.6700393	0.62887302	0.24277557	2.59034719	0.00958792	0.01437745	Up	CXCR2
CNTNAP5	2.5615626	-1.29899043	0.5018795	-2.58825165	0.00964645	0.01446278	Down	CNTNAP5
VSTM1	5.45681018	-0.77745904	0.30041004	-2.58799288	0.0096537	0.01447122	Down	VSTM1
AMER3	9.67403798	-1.05554835	0.40823795	-2.58562037	0.00972039	0.01456753	Down	AMER3
SOHLH2	4.29502115	-1.37842494	0.53313286	-2.58551863	0.00972326	0.0145706	Down	SOHLH2
GDPD4	3.90739626	0.63061106	0.24394136	2.58509282	0.00973528	0.0145861	Up	GDPD4
ZNF730	9.19311347	0.81667434	0.31605598	2.58395468	0.00976746	0.01462947	Up	ZNF730
HBG2	3.37335598	1.08699956	0.42099018	2.58200689	0.00982276	0.01470859	Up	HBG2
MMP26	0.65528732	1.86700782	0.72310178	2.58194333	0.00982457	0.01471007	Up	MMP26
TKTL1	9.87535528	1.02163904	0.39588208	2.58066501	0.00986102	0.01476093	Up	TKTL1
GPAT2	82.9769757	-0.64576018	0.25034641	-2.57946647	0.00989531	0.01480604	Down	GPAT2
CCL4L1	120.393724	0.5380759	0.20868393	2.57842524	0.00992518	0.01484576	Up	CCL4L1
OR7A5	0.64245235	1.02618917	0.39810194	2.57770449	0.0099459	0.01487426	Up	OR7A5
VRTN	11.9480423	0.83700301	0.32473073	2.5775294	0.00995094	0.01488055	Up	VRTN
C8A	0.59858206	2.099255	0.81472606	2.57663906	0.00997661	0.01491643	Up	C8A
ST6GALNAC	103.29536	0.54690996	0.21233962	2.57563783	0.01000554	0.01495844	Up	ST6GALNAC5
GNRHR	2.7276418	0.89334169	0.34689846	2.57522533	0.01001748	0.01497378	Up	GNRHR
SLC32A1	1.44762571	-1.55845598	0.60557217	-2.57352643	0.0100668	0.01504497	Down	SLC32A1
OR2T10	0.8556548	2.4891059	0.96776178	2.57202335	0.01011061	0.01510792	Up	OR2T10
UGT1A7	2.73291245	-0.80751459	0.31512809	-2.56249638	0.01039227	0.01549895	Down	UGT1A7
VSX1	1.13382008	-0.84016117	0.32787908	-2.56241166	0.0103948	0.01550143	Down	VSX1
FAM216B	6.46686732	-0.91586221	0.35774935	-2.56006672	0.01046521	0.01560382	Down	FAM216B
BOD1L2	0.46317313	1.44622456	0.56611171	2.55466284	0.01062907	0.01583359	Up	BOD1L2
MC4R	0.82022268	-1.03800399	0.40640113	-2.55413654	0.01064515	0.01585622	Down	MC4R
GJD3	4.59689732	0.88387113	0.34621704	2.55293943	0.01068181	0.01590684	Up	GJD3
CCDC141	13.4255047	-0.70120035	0.27479629	-2.55170973	0.01071958	0.01596175	Down	CCDC141
CCL18	738.177993	0.70690563	0.27710835	2.55100807	0.01074118	0.01599259	Up	CCL18
GABRA1	0.42040407	-1.58320999	0.62126049	-2.54838352	0.01082234	0.01610536	Down	GABRA1
GALNTL5	0.51526976	1.91988541	0.75340366	2.54828257	0.01082547	0.01610868	Up	GALNTL5
ZNF483	10.6277933	-0.68988524	0.27083864	-2.54721869	0.01085854	0.01615384	Down	ZNF483
ADIPOQ	89.9229514	-1.99785108	0.78451474	-2.54660743	0.01087757	0.01618081	Down	ADIPOQ
ACTL7B	0.71733913	1.1263521	0.44271312	2.5442031	0.01095274	0.01628855	Up	ACTL7B
DRP2	72.4335593	-0.62786939	0.24679498	-2.54409304	0.01095619	0.01629232	Down	DRP2
IL26	5.33872128	0.64268035	0.2530179	2.5400588	0.01108338	0.0164732	Up	IL26
DEFB126	1.14763678	2.31007896	0.9097201	2.53932936	0.01110652	0.01650075	Up	DEFB126
CFC1	0.44147801	-1.65014424	0.65066447	-2.53609091	0.01120976	0.01664997	Down	CFC1
FCN2	0.31618091	-1.36931377	0.54025247	-2.53458123	0.01125818	0.01671633	Down	FCN2
KLHL40	0.44025891	-1.63932619	0.64680066	-2.53451533	0.0112603	0.01671808	Down	KLHL40
KLK14	14.6713401	0.67223741	0.26552772	2.53170335	0.011351	0.01684573	Up	KLK14
CIB4	2.71829978	0.96899189	0.38307525	2.52950799	0.01142226	0.01694726	Up	CIB4
BPIFA1	1.37557706	2.51498222	0.9946909	2.52840577	0.01145818	0.0169968	Up	BPIFA1
CD1E	23.5543782	-0.5259587	0.20834056	-2.52451422	0.01158583	0.01717567	Down	CD1E
DNAH11	16.8137623	0.61881327	0.24537172	2.52194212	0.01167089	0.01729315	Up	DNAH11
ZDHHC19	4.39593722	-0.55477522	0.22059977	-2.51484946	0.01190832	0.01763325	Down	ZDHHC19
LENEP	1.38534682	0.79839088	0.31760321	2.51379975	0.01194382	0.01768142	Up	LENEP
F11	5.98008559	0.98945428	0.3941583	2.51029671	0.01206298	0.0178504	Up	F11
TEX13B	0.41596052	1.59963513	0.63731629	2.50995487	0.01207466	0.01786621	Up	TEX13B
PTGS2	580.23199	0.62359237	0.24850599	2.50936557	0.01209482	0.01789308	Up	PTGS2
PRAC2	74.0194328	-0.69562867	0.27762471	-2.50564397	0.01222286	0.01807501	Down	PRAC2
AADACL4	0.26420461	-1.50675265	0.60176719	-2.5038797	0.01228398	0.01815335	Down	AADACL4
PCDHA4	44.3552281	0.89624687	0.35806857	2.50300347	0.01231443	0.01819685	Up	PCDHA4

CD300E	73.9587412	0.60866751	0.243617	2.49846076	0.0124734	0.01842107	Up	CD300E
KCNJ9	4.62990604	-0.70251415	0.28161003	-2.49463464	0.01260869	0.01861188	Down	KCNJ9
ZP4	0.63881011	2.09670586	0.84055489	2.49443062	0.01261594	0.0186208	Up	ZP4
CERS4	215.601611	-0.51531223	0.20662766	-2.49391702	0.01263421	0.01864622	Down	CERS4
SPINK1	4930.46387	0.53364124	0.21497956	2.4822883	0.01305416	0.01923261	Up	SPINK1
OR13J1	1.23254773	0.94637081	0.38173493	2.47913078	0.0131703	0.01939411	Up	OR13J1
C12orf40	2.02250116	1.54996611	0.62672199	2.47313183	0.01339347	0.01970973	Up	C12orf40
GALNTL6	36.0879199	0.80729599	0.32692141	2.46938857	0.01353442	0.01990565	Up	GALNTL6
OSBPL6	63.5438941	-0.54256213	0.21977006	-2.46877181	0.01355776	0.01993671	Down	OSBPL6
VANGL2	728.140221	0.50406395	0.20420474	2.46842428	0.01357094	0.01995279	Up	VANGL2
HTN1	0.87411165	2.43857056	0.98830445	2.46742848	0.01360874	0.02000507	Up	HTN1
DNAH8	7.71846258	0.76227519	0.30921293	2.4652112	0.01369325	0.02012267	Up	DNAH8
HBA1	24.0853007	0.89260571	0.36233482	2.46348312	0.01375944	0.02021328	Up	HBA1
TUBA3C	0.62150014	1.67384501	0.6805962	2.4593805	0.0139177	0.02043737	Up	TUBA3C
SPRR2F	1.26187271	1.95137235	0.79459708	2.45580105	0.0140571	0.02063017	Up	SPRR2F
GREM1	3864.89289	0.55179273	0.2247955	2.45464309	0.01410245	0.02069338	Up	GREM1
FNDC8	2.44029958	0.50653967	0.206478	2.45323798	0.01415766	0.02076752	Up	FNDC8
C3orf20	3.81076602	-0.55969856	0.22817192	-2.45296863	0.01416827	0.02078137	Down	C3orf20
VNN2	101.867694	-0.60551003	0.24698761	-2.45158057	0.01422303	0.02085827	Down	VNN2
RAB27B	481.402891	-0.53137925	0.21678134	-2.45122226	0.0142372	0.02087733	Down	RAB27B
DCT	13.515823	0.97580985	0.39817876	2.45068284	0.01425855	0.02090692	Up	DCT
TTR	31.4597516	-0.85049551	0.34733904	-2.44860329	0.01434113	0.02102282	Down	TTR
ACSM6	2.50969179	1.33108455	0.54363511	2.44848894	0.01434568	0.02102777	Up	ACSM6
SLC22A10	0.49457928	1.26804947	0.51821268	2.44696726	0.01440639	0.02111502	Up	SLC22A10
KRTAP10-6	0.52484921	1.66101966	0.67884058	2.4468479	0.01441116	0.02112028	Up	KRTAP10-6
SFTPC	1.75462388	-1.52041936	0.62188942	-2.44483877	0.01449169	0.02123306	Down	SFTPC
CIDEA	7.40372181	-1.84716636	0.75693441	-2.44032552	0.01467403	0.02148435	Down	CIDEA
OTOF	23.4955287	0.65203046	0.26778498	2.43490297	0.01489578	0.0217965	Up	OTOF
G6PC2	1.42198746	-0.95888242	0.39399562	-2.43373878	0.01494378	0.02186314	Down	G6PC2
TTC6	17.8206208	-0.52442648	0.21578226	-2.43035031	0.01508424	0.02205779	Down	TTC6
ALOX15	66.640515	0.58854113	0.24257013	2.42627208	0.01525483	0.02228899	Up	ALOX15
CAV3	1.93076205	-1.06430187	0.43896653	-2.4245627	0.01532684	0.0223887	Down	CAV3
PAGE4	0.53967817	1.93031041	0.79776028	2.41966222	0.01553493	0.02267782	Up	PAGE4
NDUFA7	1.60530111	0.65594572	0.27132421	2.41757171	0.01562446	0.02280478	Up	NDUFA7
VCX3B	0.45508814	1.16720222	0.48286441	2.41724634	0.01563843	0.02282331	Up	VCX3B
PRDM14	0.68932821	1.58723618	0.65695063	2.41606616	0.01568921	0.0228918	Up	PRDM14
MAG	1.55328912	-0.91168696	0.37758528	-2.4145193	0.01575599	0.0229836	Down	MAG
APOD	590.344459	-0.60212099	0.24938254	-2.41444723	0.01575911	0.02298627	Down	APOD
COL4A4	85.1452998	-0.56606011	0.23453508	-2.41354137	0.01579833	0.02303972	Down	COL4A4
ATP13A5	1.17875651	-1.01872258	0.42419981	-2.40151589	0.0163273	0.02378199	Down	ATP13A5
DCC	11.4681767	-0.57996424	0.24157909	-2.400722	0.01636276	0.0238317	Down	DCC
RNF222	2.04624643	0.63633045	0.26519831	2.3994514	0.01641966	0.02391067	Up	RNF222
TAS1R3	153.852511	0.55673875	0.23219211	2.39775057	0.01649609	0.02401218	Up	TAS1R3
SPDYE4	0.35149845	-1.0219192	0.42708911	-2.39275404	0.01672245	0.02432579	Down	SPDYE4
OCSTAMP	1.71685593	0.75362374	0.31511199	2.39160605	0.01677484	0.02439405	Up	OCSTAMP
KRTAP5-2	0.7809396	0.81994088	0.34313415	2.38956359	0.0168684	0.02452611	Up	KRTAP5-2
TMEFF1	0.9011458	-0.7656683	0.32075084	-2.38711235	0.0169813	0.02468021	Down	TMEFF1
XKR6	54.2240679	0.55808031	0.23381673	2.38682799	0.01699444	0.0246973	Up	XKR6
SLC35G6	1.72558975	0.80909477	0.33920425	2.38527312	0.01706645	0.0247979	Up	SLC35G6
CIB3	0.56674612	-0.77675735	0.32574894	-2.38452763	0.01710106	0.02484618	Down	CIB3
TNMD	47.7984619	0.64084721	0.26910128	2.38143497	0.01724534	0.02505171	Up	TNMD
SCRT2	0.85799979	1.48625548	0.62474313	2.37898652	0.01736031	0.02520437	Up	SCRT2
GP2	111.045298	-1.02597005	0.4314302	-2.37806729	0.01740365	0.02526524	Down	GP2
TNC	4448.83392	-0.51762196	0.21768374	-2.37786235	0.01741332	0.02527722	Down	TNC
DGKK	2.48066701	0.86537879	0.36414567	2.37646323	0.0174795	0.02536504	Up	DGKK
PCDHGA5	23.1746451	-0.50391139	0.21222745	-2.37439306	0.01757783	0.0255015	Down	PCDHGA5
AMDHD1	17.9378751	0.51581907	0.21734091	2.37331792	0.01762908	0.02556962	Up	AMDHD1
MS4A18	0.81391462	1.43611066	0.60524095	2.37279164	0.01765422	0.02560192	Up	MS4A18

SERPINC1	3.53083114	0.6404123	0.27038034	2.36856091	0.01785744	0.0258735	Up	SERPINC1
PON1	10.6384788	0.8660927	0.36577536	2.3678268	0.01789291	0.02592279	Up	PON1
LRIT1	0.50941097	1.35845785	0.57425022	2.36562006	0.01799991	0.02606521	Up	LRIT1
RGS8	2.16172722	-0.63870916	0.27064277	-2.35997129	0.01827635	0.02644824	Down	RGS8
DSCAM	3.95551377	-0.6932116	0.29379345	-2.35952029	0.01829858	0.02647612	Down	DSCAM
CRX	0.68535936	0.99919414	0.42363924	2.35859679	0.01834418	0.02653779	Up	CRX
KLHL14	17.9118011	-0.73932656	0.31363834	-2.35725825	0.01841044	0.02662717	Down	KLHL14
CELA2A	0.8079264	1.08861825	0.46196582	2.35649092	0.01844852	0.02668009	Up	CELA2A
COL20A1	2.93221574	0.89348469	0.37919084	2.3562929	0.01845836	0.02668999	Up	COL20A1
PNLIPRP1	8.14701815	-0.56926606	0.24160092	-2.35622471	0.01846175	0.02669273	Down	PNLIPRP1
CALCB	22.0737475	1.06129584	0.45055247	2.35554327	0.01849564	0.02673524	Up	CALCB
HOXD10	153.985108	-0.63329688	0.26907065	-2.35364532	0.01859034	0.02686342	Down	HOXD10
ANKRD30A	0.65946851	1.91207003	0.81277867	2.35251008	0.01864718	0.02693683	Up	ANKRD30A
P2RX3	3.54522996	0.80722725	0.34371797	2.34851631	0.01884837	0.02720984	Up	P2RX3
CSRP3	0.50979871	1.3631789	0.58105222	2.34605231	0.01897344	0.02738597	Up	CSRP3
CLEC4E	56.1822615	0.55866914	0.23843518	2.34306506	0.01912605	0.0275973	Up	CLEC4E
ARSF	0.7527008	-0.72719934	0.31051881	-2.34188502	0.01918663	0.02768247	Down	ARSF
C20orf141	0.27327194	1.19116985	0.50866906	2.34173838	0.01919416	0.02769111	Up	C20orf141
HEATR9	2.97159067	0.73120555	0.31229017	2.34142992	0.01921003	0.02771176	Up	HEATR9
THEGL	0.69123876	-0.94918045	0.40548488	-2.34085285	0.01923975	0.02775239	Down	THEGL
SLC30A3	6.84733391	0.60514338	0.25875944	2.33863307	0.01935443	0.02790879	Up	SLC30A3
LDHAL6B	4.47188589	-0.5878642	0.25197214	-2.33305239	0.0196454	0.02831463	Down	LDHAL6B
A4GNT	0.81165155	1.01193394	0.43442984	2.329338	0.01984117	0.02857141	Up	A4GNT
PCDHGB5	60.6601232	-0.60590623	0.26020646	-2.3285595	0.01988241	0.02862388	Down	PCDHGB5
KRTAP5-7	1.33430592	0.93762936	0.40290188	2.32719034	0.01995514	0.02872394	Up	KRTAP5-7
CCDC110	33.4001785	-0.5231702	0.22493542	-2.3258685	0.02002557	0.02882299	Down	CCDC110
SPDYE2B	0.91573419	1.16631894	0.50200897	2.323303	0.02016288	0.02901361	Up	SPDYE2B
SLC6A11	1.54877463	-0.72702788	0.3131401	-2.32173356	0.02024729	0.02912802	Down	SLC6A11
TCTE1	2.54047822	-0.68175221	0.29397161	-2.31910903	0.02038913	0.02931317	Down	TCTE1
SLC7A4	191.704757	0.51348336	0.22176387	2.31545089	0.02058827	0.02958041	Up	SLC7A4
GMNC	1.00895775	1.53455693	0.66285527	2.31507088	0.02060905	0.0296055	Up	GMNC
ZAR1L	0.72407169	0.80571052	0.34809654	2.31461799	0.02063385	0.02963333	Up	ZAR1L
ACSM2B	1.0537284	-1.12923694	0.48915131	-2.30856364	0.02096781	0.03008696	Down	ACSM2B
HCN2	31.2203618	0.52960003	0.22948549	2.30777128	0.02101186	0.03014532	Up	HCN2
MRAP	1.95007788	-0.83762101	0.36296676	-2.30770723	0.02101543	0.03014559	Down	MRAP
IL17REL	13.8182673	-0.69345122	0.30078146	-2.30549858	0.02113867	0.03031019	Down	IL17REL
OPRM1	0.63214473	-1.27820177	0.55455889	-2.30489818	0.02117228	0.03035351	Down	OPRM1
CXCR1	49.7878638	0.63528896	0.27627208	2.29950475	0.02147629	0.03075724	Up	CXCR1
VTN	15.3152242	0.59227883	0.25757518	2.29944064	0.02147993	0.03075998	Up	VTN
OR11H7	0.90838075	0.7546359	0.32821741	2.29919518	0.02149386	0.03077252	Up	OR11H7
GATA5	11.1340482	-0.94852876	0.41302418	-2.29654534	0.02164472	0.03098105	Down	GATA5
C8B	0.59106773	1.75703647	0.76519523	2.2961937	0.02166481	0.03100483	Up	C8B
C7orf33	0.62545576	1.96458471	0.8556828	2.29592638	0.02168009	0.03102421	Up	C7orf33
DLGAP1	24.7796868	-0.59336046	0.25845827	-2.29576891	0.0216891	0.03103212	Down	DLGAP1
IFNL2	0.3077656	1.30211537	0.56721501	2.29562927	0.02169709	0.03103858	Up	IFNL2
TAS2R10	2.32421585	0.62237641	0.27139099	2.29328321	0.02183171	0.03122614	Up	TAS2R10
LUC7L2	0.78001419	-0.55054179	0.24016171	-2.29237951	0.02188375	0.03129578	Down	LUC7L2
GRM6	2.64190062	-0.5168838	0.22548656	-2.29230424	0.02188809	0.03129927	Down	GRM6
SIAH3	2.4663275	-0.69680145	0.30441954	-2.28895111	0.02208219	0.03156165	Down	SIAH3
SLC6A2	3.60535054	-1.13185133	0.49504419	-2.28636422	0.02223296	0.03177205	Down	SLC6A2
OOEP	9.45697003	1.10870917	0.48494252	2.28626926	0.02223851	0.03177744	Up	OOEP
CSMD3	3.7496423	-0.83797673	0.36768645	-2.2790525	0.02266394	0.03235686	Down	CSMD3
C7orf57	4.15051002	0.68456109	0.30074266	2.27623538	0.02283193	0.03258886	Up	C7orf57
SFTPA1	3.06971446	-1.09954156	0.48346309	-2.274303	0.02294777	0.03274636	Down	SFTPA1
ODF3	1.01564871	1.55030455	0.68226017	2.272307	0.02306797	0.0329021	Up	ODF3
OR8G5	0.40635438	-1.67608297	0.73763438	-2.27224085	0.02307197	0.03290517	Down	OR8G5
NKAIN2	44.559116	0.9313004	0.41036693	2.26943335	0.02324199	0.03313705	Up	NKAIN2
DEFB124	1.01792958	-0.8026758	0.35386962	-2.2682812	0.02331207	0.03322371	Down	DEFB124

EXD1	1.1677081	-0.57331441	0.25381849	-2.2587575	0.02389847	0.03399969	Down	EXD1
WEE2	2.39276297	0.62385062	0.27627936	2.25804281	0.02394299	0.03405488	Up	WEE2
LIN7A	149.198639	0.5550159	0.24587423	2.25731627	0.02398832	0.03411664	Up	LIN7A
C2orf78	0.34519057	1.31464142	0.58277013	2.25584902	0.02408009	0.03423078	Up	C2orf78
SPATA45	1.1130283	-0.5339685	0.23671888	-2.25570728	0.02408897	0.03424068	Down	SPATA45
POU4F3	1.31584475	0.5446862	0.24151758	2.25526522	0.02411669	0.03427735	Up	POU4F3
NPIPA8	0.42412168	1.15037373	0.51009875	2.25519809	0.0241209	0.0342806	Up	NPIPA8
KCNU1	0.91275501	1.48217967	0.65826712	2.25163862	0.02434512	0.03457999	Up	KCNU1
SSUH2	148.355352	0.51140715	0.2271691	2.25121796	0.02437174	0.03461229	Up	SSUH2
INSM2	0.58983227	1.14131344	0.50700345	2.25109598	0.02437946	0.0346205	Up	INSM2
ALX3	0.59952694	2.07634489	0.92378733	2.24764381	0.02459891	0.03491824	Up	ALX3
NPFFR2	2.97374806	-0.58454881	0.26038305	-2.2449572	0.02477088	0.03515676	Down	NPFFR2
LRRC71	2.96450345	0.6363548	0.28359373	2.24389591	0.0248391	0.03524798	Up	LRRC71
FOXI1	14.8312284	0.99747234	0.44458655	2.24359538	0.02485845	0.03527263	Up	FOXI1
VSX2	0.44747079	-0.95643045	0.42663879	-2.24178032	0.02497557	0.03543037	Down	VSX2
AMBN	0.91880434	1.37745415	0.61481036	2.2404537	0.02506148	0.03554377	Up	AMBN
CTXN1	151.025326	-0.53127379	0.23713622	-2.24037387	0.02506666	0.03554642	Down	CTXN1
IGFBPL1	14.3874001	0.63215404	0.28246937	2.23795608	0.02522392	0.03575425	Up	IGFBPL1
CHRNA4	1.16458125	-1.03552106	0.46378101	-2.23278022	0.02556344	0.03621251	Down	CHRNA4
GC	2.1825399	1.63016612	0.73133261	2.2290352	0.02581156	0.03654658	Up	GC
ACSM2A	0.86396396	-1.11933519	0.50270661	-2.22661723	0.02597287	0.03675165	Down	ACSM2A
ASMT	4.25395929	0.51715655	0.23256206	2.2237357	0.02616623	0.03700765	Up	ASMT
DSPP	0.8821008	1.81551911	0.81788015	2.21978624	0.02643328	0.03736093	Up	DSPP
LPA	2.86379384	-0.66805979	0.30115193	-2.21834805	0.02653111	0.03749103	Down	LPA
ALX4	2.8148975	0.74446039	0.33603338	2.21543584	0.02673016	0.03775437	Up	ALX4
ENPP7	3.34637674	0.77080605	0.34796423	2.21518757	0.02674719	0.03777543	Up	ENPP7
OR52N4	0.86948706	-0.83973232	0.38011527	-2.20915176	0.02716409	0.03832782	Down	OR52N4
HAO1	0.60160397	2.18738668	0.99251518	2.20388235	0.02753262	0.03883246	Up	HAO1
GRM5	0.59155156	1.19858301	0.54402092	2.20319288	0.02758116	0.03889785	Up	GRM5
HOXC10	11.7223464	0.87118924	0.39598146	2.20007585	0.02780151	0.03919313	Up	HOXC10
FABP9	0.43774302	1.30926438	0.59614255	2.19622704	0.0280757	0.03957028	Up	FABP9
SAMD7	0.79652635	1.13583985	0.51737215	2.19540201	0.02813477	0.03964728	Up	SAMD7
RFPL4B	0.68992835	1.35273069	0.61631524	2.19486816	0.02817306	0.03969496	Up	RFPL4B
RPE65	4.77727317	0.85296351	0.38955261	2.18959771	0.02855343	0.04020867	Up	RPE65
PNMA2	154.187414	0.51905822	0.23741086	2.18632888	0.02879155	0.0405344	Up	PNMA2
OR1J2	0.42721831	1.43734374	0.65804983	2.18424758	0.02894406	0.04073947	Up	OR1J2
APOL5	0.44868433	0.96665223	0.44294403	2.18233493	0.02908482	0.04093114	Up	APOL5
PCDHA13	1.60607569	-0.86094517	0.39480647	-2.18067647	0.02920735	0.04109061	Down	PCDHA13
GPR151	0.75326881	1.42331223	0.65271614	2.18059909	0.02921308	0.04109543	Up	GPR151
GPR18	43.7557796	-0.60272821	0.27643049	-2.18039704	0.02922804	0.04111324	Down	GPR18
TMPRSS15	1.81824459	1.3156447	0.60451942	2.17634811	0.02952924	0.04150747	Up	TMPRSS15
NELL1	13.2404585	-0.60906809	0.27998542	-2.17535645	0.02960342	0.0416019	Down	NELL1
ST8SIA5	4.66328496	-0.53433055	0.24570668	-2.17466841	0.02965497	0.04166451	Down	ST8SIA5
RASA4B	2.02142767	-0.53540816	0.24629722	-2.17382954	0.02971794	0.04174641	Down	RASA4B
ANXA8	2.92717805	0.88778763	0.40877262	2.1718373	0.02986794	0.04193731	Up	ANXA8
C22orf42	0.47261825	-0.87900078	0.40524752	-2.16904667	0.03007914	0.04222721	Down	C22orf42
STMND1	27.3610926	-0.60093676	0.2770676	-2.16891744	0.03008895	0.04223434	Down	STMND1
SERPINA12	0.37357965	1.26896275	0.58521344	2.16837595	0.03013009	0.04228544	Up	SERPINA12
ANKRD30B	9.64517145	0.93537834	0.43172764	2.16659361	0.03026586	0.04246261	Up	ANKRD30B
CLEC12A	38.613765	-0.50978566	0.23552314	-2.16448225	0.03042736	0.04268249	Down	CLEC12A
MSI1	163.966584	0.53860244	0.24889869	2.16394245	0.03046877	0.04273386	Up	MSI1
GALNT13	17.2647969	-0.81728186	0.37807281	-2.16170495	0.03064093	0.04296181	Down	GALNT13
CXCL9	1206.38847	0.5431131	0.25140152	2.16034133	0.03074626	0.04310611	Up	CXCL9
THSD7B	9.98027062	-0.74500386	0.34712541	-2.14620951	0.03185627	0.04456431	Down	THSD7B
ARHGDIG	96.6435753	0.62918921	0.29319183	2.14599843	0.0318731	0.04458088	Up	ARHGDIG
LBX1	0.53947304	1.810083	0.84456311	2.14321815	0.03209559	0.04486755	Up	LBX1
KRTAP12-1	0.33823885	1.30198942	0.6082908	2.14040623	0.03232195	0.04516974	Up	KRTAP12-1
PRPS1L1	0.39645069	1.29609358	0.60588641	2.13916927	0.03242196	0.04530241	Up	PRPS1L1

CLEC2L	10.0702646	0.67166234	0.31421022	2.13762088	0.03254753	0.04547429	Up	CLEC2L
MTRNR2L2	0.62124101	-0.72477371	0.33917805	-2.13685324	0.03260993	0.04555435	Down	MTRNR2L2
RUNDC3A	46.8855924	-0.5607649	0.26246703	-2.13651553	0.03263742	0.04558918	Down	RUNDC3A
CDH2	91.9313888	-0.50316582	0.23553371	-2.13627944	0.03265664	0.04561247	Down	CDH2
PRR18	3.84878878	0.81490852	0.38155456	2.13575882	0.03269908	0.04566816	Up	PRR18
LHFPL3	36.6088246	-0.61846627	0.28961812	-2.13545433	0.03272392	0.04569725	Down	LHFPL3
IAPP	0.39232657	1.63590417	0.76624209	2.13497039	0.03276343	0.04574371	Up	IAPP
FAM217A	3.6446671	0.69187162	0.32413446	2.1345204	0.03280021	0.0457879	Up	FAM217A
RNASE3	2.06267924	-0.56324748	0.26436631	-2.13055693	0.03312566	0.04622776	Down	RNASE3
SLC10A1	6.06639325	-0.57208593	0.26871735	-2.12895047	0.03325836	0.0463843	Down	SLC10A1
NLRP11	13.2339915	0.53442235	0.25102422	2.12896725	0.03325697	0.0463843	Up	NLRP11
KRTAP5-5	13.1191665	0.60239432	0.28302111	2.12844309	0.03330036	0.04643162	Up	KRTAP5-5
BPIFA2	0.64023099	1.54032514	0.72394108	2.12769406	0.03336246	0.04651093	Up	BPIFA2
HBM	0.35781705	-1.18890092	0.55908215	-2.12652278	0.03345975	0.04663929	Down	HBM
FTCD	8.31162357	0.74169594	0.34937255	2.12293706	0.03375913	0.04703086	Up	FTCD
XAGE3	0.71373806	0.84356944	0.39746589	2.12236941	0.03380673	0.04708983	Up	XAGE3
RS1	2.03228516	0.67229777	0.31682505	2.12198428	0.03383906	0.04713118	Up	RS1
KBTBD12	83.8759997	-0.69412262	0.3271213	-2.12191203	0.03384513	0.04713595	Down	KBTBD12
LEFTY2	44.7941904	0.65426158	0.30837594	2.1216363	0.03386829	0.04716454	Up	LEFTY2
FAM133A	2.95436423	-0.91330657	0.43065007	-2.12076262	0.03394178	0.0472595	Down	FAM133A
RAMP1	609.940476	-0.56554111	0.2672065	-2.11649461	0.03430276	0.04773605	Down	RAMP1
TEDDM1	1.81190899	0.77371517	0.36585148	2.11483409	0.03444409	0.04791778	Up	TEDDM1
HAPLN2	6.80916044	0.61378546	0.29126126	2.10733643	0.03508843	0.04876854	Up	HAPLN2
NYX	0.4101988	-0.8957672	0.42540701	-2.10567096	0.03523294	0.04896178	Down	NYX
