# Supplementary material for: Development of a prognostic risk model for colorectal cancer based on microsatellite stability-associated genes
Source: BMC Cancer. 2025 Oct 1;25:1490. doi: 10.1186/s12885-025-14918-y (PMC12487216; doi:10.1186/s12885-025-14918-y)
Supplement: Supplementary file 11 — Supplementary Material 11. [file 12885_2025_14918_MOESM11_ESM.zip › Table S3.pdf]

	coef	absolute value of coef	Weight proportion
CHGB	0.674679949328963	0.674679949328963	3.71%
FABP4	0.945182257528798	0.945182257528798	5.19%
PLIN4	0.526152842408604	0.526152842408604	2.89%
PLIN1	-0.530178638	0.530178637596166	2.91%
RPRM	3.38842182483815	3.38842182483815	18.62%
C7	-2.446800723	2.44680072272134	13.44%
AQP8	0.217537560043027	0.217537560043027	1.20%
C2CD4A	-1.573011378	1.57301137828441	8.64%
APLP1	-4.624562814	4.62456281448948	25.41%
ADH1B	0.987280700096597	0.987280700096597	5.42%
CD36	2.28622957158893	2.28622957158893	12.56%
